# Computer-aided polyp detection and characterisation systems to support colonoscopy: a systematic review with results stratified by each individual artificial intelligence system

**DOI:** 10.1136/bmjdh-2026-000046

**Published:** 2026-06-22

**Authors:** Nicole Downes, Clare Dadswell, Sophie Ip, Isaac Mackenzie, Victoria Wakefield, Steven J Edwards

**Affiliations:** BMJ Technology Assessment Group (BMJ-TAG), BMJ Group, London, England, UK

**Keywords:** Artificial intelligence, Colorectal Neoplasms, Colorectal Tumors, Colorectal Carcinoma, Decision Making, Computer-Assisted

## Abstract

**Objective:**

To assess the clinical effectiveness and diagnostic accuracy of specific artificial intelligence (AI) systems with computer-aided polyp detection (CADe) and/or computer-aided polyp characterisation (CADx) functionalities.

**Methods and analysis:**

Studies using CADe/CADx systems alongside endoscopist judgement during real-time colonoscopies for any indication were included from searches performed in June 2025. Adenoma detection rate (ADR) and diagnostic accuracy (eg, sensitivity and specificity) were key outcomes, with other polyp detection outcomes, procedural duration and adverse events (AEs) also analysed. For CADe, meta-analyses were stratified by each individual AI system. Meta-analyses were not performed for CADx due to heterogeneity concerns. Subgroup analyses for colonoscopy indication and endoscopist experience were explored.

**Results:**

Overall, 52 studies (43 for CADe and 9 for CADx) covering eight CADe/CADx systems were included. ADR increased with each individual CADe system versus colonoscopy without CADe; results for two systems lacked statistical significance. While more uncertain, some systems also increased the detection of advanced and non-advanced adenomas, different-sized adenomas, sessile serrated lesions (SSLs) and/or hyperplastic/non-neoplastic polyps. Adjunct CADx diagnostic accuracy results were inconsistent and had limitations; comparisons against endoscopist optical diagnoses were often not available, SSLs were often analysed as non-neoplastic and low-confidence diagnoses were often excluded. CADe may increase procedural duration slightly, and no concerns about AEs were identified. No data for the impact of CADe/CADx on long-term outcomes, such as interval colorectal cancer (CRC) or mortality, were available. No robust conclusions about effects across different colonoscopy indications or endoscopist experience subgroups could be made.

**Conclusion:**

The included CADe systems may each increase ADR, with increased detection of other polyp types possible and no major concerns about procedure duration or AEs. Further research on CADx diagnostic accuracy, CADe/CADx impact on long-term outcomes such as CRC incidence and effects across colonoscopy indication and endoscopist experience subgroups is required.

**PROSPERO registration number:**

CRD42024586541.

**WHAT IS ALREADY KNOWN ON THIS TOPIC**

- Colorectal cancer (CRC) is the fourth most common cancer in the UK, accounting for~10% of UK cancer deaths. Colorectal polyp detection during colonoscopies is a key component of current strategies aiming to reduce the risk of CRC. Previous systematic reviews have assessed the evidence for computer-aided polyp detection (CADe) systems during colonoscopy, but these have largely meta-analysed all CADe systems together as a single intervention. Furthermore, while reviews of computer-aided polyp characterisation (CADx) systems during colonoscopy also exist, these have typically permitted the inclusion of study types that are less applicable to clinical practice, such as those where the CADx systems are used autonomously rather than alongside clinician judgement or applied to videos or photos from prior colonoscopies rather than during real-time colonoscopy procedures.

**WHAT THIS STUDY ADDS**

- The inclusion of separate meta-analyses for each individual CADe system from the outset, rather than combining all CADe systems as a single CADe intervention, is a unique strength of this systematic review given the CADe systems have unique algorithms and may have differing impacts on outcomes; different CADe systems may improve adenoma detection rate (ADR) or other detection outcomes to different extents, which may in turn lead to differences in the impact on clinical outcomes such as interval CRC. Furthermore, this review prioritises the most clinically relevant data for CADx, including focusing on those using the CADx system during real-time colonoscopies and as an adjunct to endoscopist judgement. This means that only the most clinically useful information is included, allowing the current gaps and limitations of the evidence base to be more clearly highlighted.

**HOW THIS STUDY MIGHT AFFECT RESEARCH, PRACTICE OR POLICY**

- This research highlights the potential for different CADe systems to increase polyp detection but also highlights key evidence gaps and areas where future research is required. This research was performed as part of the National Institute for Health and Care Excellence (NICE) assessment of artificial intelligence (AI) technologies to help detect or characterise colorectal polyps within the NICE HealthTech Evaluation programme, and has directly informed recommendations made as a result of its assessment.

## Introduction

### Background

Colorectal cancer (CRC) is the fourth most common cancer in the UK, accounting for 10% of cancer-related deaths. Earlier diagnosis improves survival chances.^1 2^ Colorectal polyps, large bowel lesions, can develop into CRC.^1 3 4^ ‘Adenomatous’ colorectal polyps can progress to CRC through the adenoma-carcinoma pathway.^5 6^ Pathways linking other polyp types, including serrated lesions, to CRC also exist.^7^ Collectively, polyps that can progress to CRC are termed ‘neoplastic’ polyps.^8^ Early identification and investigation of colorectal polyps using colonoscopy is a key component of strategies aiming to reduce CRC risk.^1 3^

Artificial intelligence (AI) systems designed to support endoscopists during colonoscopy exist. Functions include computer-aided polyp detection (CADe) and computer-aided polyp characterisation (CADx) to support colorectal polyp detection and optical diagnosis (assessing whether polyps are neoplastic or not), respectively. Functions supporting other aspects of colonoscopy also exist.^9^ Guidance on CADe from health technology appraisal (HTA) groups and professional societies is mixed, and no existing guidance recommending CADx use was identified.^10–17^

This research builds on previous CADe reviews by performing separate meta-analyses for each individual CADe system from the outset, allowing the impact of each CADe system to be assessed rather than CADe systems overall.^11 18–21^ This is important, as the underlying AI algorithms for each CADe system differ, potentially leading to different impacts on colonoscopy outcomes, such as adenoma detection rate (ADR) and downstream clinical outcomes, such as interval CRC.

Furthermore, this research focuses on the most clinically relevant CADx studies, using systems during real-time colonoscopies and alongside clinician judgement. Previous systematic literature reviews (SLRs) of CADx have generally permitted the inclusion of studies less relevant to clinical practice, such as CADx applied to colonoscopy recordings (ex vivo use) or used autonomously without considering endoscopist judgement.^9 22–25^ Neither reflects their intended use; manufacturers advise use alongside clinician judgement, and ex vivo use does not capture conditions during colonoscopies, including interactions between the CADx system and the endoscopist. As for CADe, consideration of each individual CADx system separately was deemed important herein, unlike two recent CADx meta-analyses that make broad conclusions for CADx overall.^24 25^

### Aims of this research

This research was commissioned by the National Institute for Health and Care Research (NIHR) Evidence Synthesis Programme (NIHR 136019) within a National Institute for Health and Care Excellence (NICE) HealthTech Evaluation.^26^ The HealthTech evaluation aimed to compare each AI system against colonoscopy without AI rather than comparing different AI systems; therefore, formal methods of comparing systems were not employed herein, and conclusions about the comparability of results between different systems are not made. This publication focuses on key, publicly available evidence from the clinical SLR within this evaluation. A separate publication on the economic analysis is anticipated.

## Methods

### Patient and public involvement

No patient or public involvement was included in the design, conduct, reporting or dissemination of the results discussed herein; however, some general feedback on colonoscopy from a patient representative was included in the full report delivered for the NICE HealthTech Evaluation.^26^

### Protocol development and search strategies

The protocol was registered on PROSPERO (CRD42024586541; Online supplemental file 1).^27^ Searches of bibliographic databases (MEDLINE, Embase, the Cochrane Database of Systematic Reviews and the Cochrane Central Register of Controlled Trials) and grey literature resources (trial registries, conferences and HTA databases) were performed, and reference lists of relevant papers were reviewed. Searches were performed in September 2024 and updated in June 2025. No study design or language limits were applied, but a date limit of 2010 onwards was used.^11^ Further details, including search strategies, are provided in online supplemental file 2. Six clinical experts (consultant gastroenterologists, consultant surgeons and consultant oncologists) provided advice at various stages of this research, including from protocol development to review of the completed NICE report.

10.1136/bmjdh-2026-000046.supp1 Supplementary data

10.1136/bmjdh-2026-000046.supp2 Supplementary data

### Inclusion and exclusion criteria

Inclusion and exclusion criteria are outlined in online supplemental file 2.^27 28^ Any population undergoing colonoscopy was eligible. The NICE final scope included 11 CADe/CADx systems; however, only systems/studies with publicly available data at the time of writing that had not withdrawn from the UK market and were using the technologies adjunctly are included here.

The relevant comparator was colonoscopy without CADe/CADx, although single-arm diagnostic accuracy studies were eligible. A broad range of outcomes was analysed (online supplemental file 2); those deemed key during the NICE assessment are prioritised herein. Randomised controlled trials (RCTs) were prioritised where outcomes (other than diagnostic accuracy) were reported by at least one RCT. Conference abstracts were included where no other data were available for a particular AI system. Application during real-time colonoscopies (in vivo) was required.

### Study selection, data extraction and quality assessment

Deduplication was performed in the Rayyan software.^29^ Two independent reviewers performed title and abstract (in Rayyan) and full-text (in Microsoft Excel) screening; a single reviewer screened grey literature. One reviewer extracted data and assessed risk of bias, with validation by a second reviewer. The Cochrane risk of bias tool for randomised trials (V.2) and Quality Assessment of Diagnostic Accuracy Studies (QUADAS)-2 were used for RCTs and diagnostic accuracy studies, respectively.^30 31^ Abstracts were assigned a high risk of bias. A third reviewer was available to support screening, data extraction and risk of bias discussions.

### Synthesis and statistical analysis

Separate random effects meta-analyses for each individual CADe system compared with colonoscopy without CADe were performed in V.5.3.5 of Review Manager.^32^ High risk of bias studies were excluded from the main analyses, unless covering a unique outcome or population.^33^ Dichotomous outcomes were analysed as risk ratios (RRs), Peto odds ratios (ORs) or risk differences, depending on the event number; Cochrane highlights Peto ORs as the method associated with the least bias when event rates are <1.0%.^33^ Continuous outcomes were analysed as mean differences, with an incidence rate ratio (IRR) analysis also performed for adenomas per colonoscopy (APC).

Intention-to-treat (ITT) analyses and raw data (rather than reported effect estimates) were prioritised where available. All colonoscopy populations were combined in the main CADe meta-analyses, with subgroup analyses for colonoscopy indications and endoscopist experience explored. CADx meta-analyses were not performed due to heterogeneity. See online supplemental file 2.

## Results

### Search and screening results

Overall, 8092 records were retrieved from database searches; 5815 deduplicated records were screened, and 768 full-text records were sought. From database and grey literature searches, 70 studies (from 72 records) were included in the overall NICE assessment. A Preferred Reporting Items for Systematic reviews and Meta-Analyses (PRISMA) diagram is presented in online supplemental file 2.^26^ This publication focuses on 52 (from 53 records) of these studies.^34–86^

### Included studies

Included studies are summarised in online supplemental file 2. Summary data extraction tables and excluded study tables are provided in online supplemental file 3. Of the 52 studies, 43 covered CADe data,^34–47 53 54 56–82 86^ and 9 covered CADx data.^48–52 55 83–85^ Most covered CAD EYE (n=18 studies)^36–53^ or GI Genius (n=16 studies),^44 71–85^ with fewer for other AI systems. Most included mixed colonoscopy indication populations; higher CRC risk groups (eg, inflammatory bowel disease (IBD), polyposis syndromes or prior CRC) were commonly excluded, although one study each covering Lynch syndrome and IBD populations was included for CAD EYE and GI Genius.^40 52 75 77^ Endoscopist experience ranged from only trainees to only experienced or expert endoscopists.

10.1136/bmjdh-2026-000046.supp3 Supplementary data

### Risk of bias assessment

Risk of bias assessments are presented in online supplemental file 3. Most CADe RCTs had ‘some concerns’, with all studies being unblinded.^30^ Most CADx studies had some limitations, including autonomous use, classifying sessile serrated lesions (SSLs) as non-neoplastic and/or excluding low-confidence endoscopist optical diagnoses. The Hawthorne effect was an additional concern for CADe/CADx,^87^ where the knowledge of being observed in trials may influence endoscopist colonoscopy performance. Furthermore, publication bias may be a concern (online supplemental file 3).

### CADe outcomes

#### Adenoma detection rate

**Figure 1:**
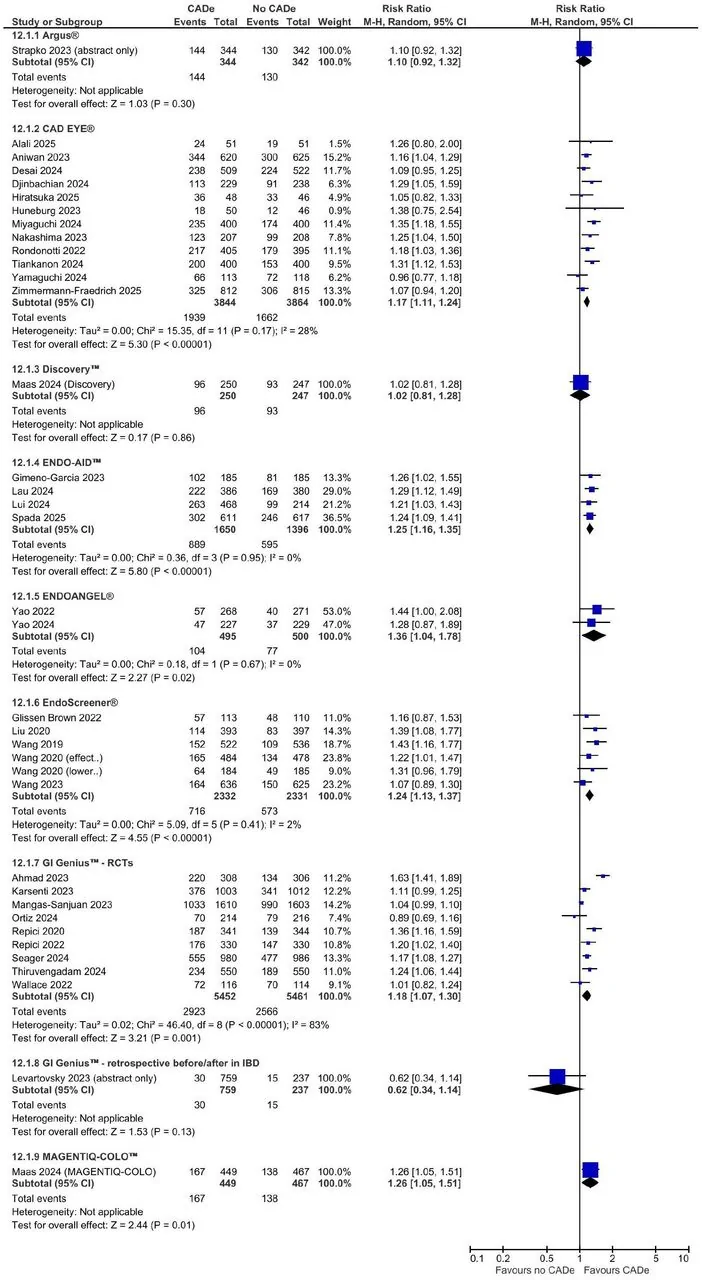
Meta-analyses of overall ADR for each individual CADe system. ADR, adenoma detection rate; CADe, computer-aided polyp detection; IBD, inflammatory bowel disease; M-H, Mantel-Haenszel; RCT, randomised controlled trial.

ADR refers to the proportion of total colonoscopies where at least one adenoma is detected. An increased ADR has been associated with a reduced interval CRC risk^88 89^; therefore, CADe systems may be beneficial if they increase ADR, although any link to reduced interval CRC can only be inferred currently, as this was not assessed within the trials. For overall ADR, each individual CADe system increased ADR versus colonoscopy without CADe (figure 1). The number of colonoscopies analysed ranged between 497 and 10 913, with point estimates from RCTs between 1.02 and 1.36; however, results were not statistically significant for Argus and Discovery. Fewer studies and sample sizes may contribute to this lack of significance, as point estimates are similar to some individual studies within other CADe system meta-analyses. A retrospective GI Genius study in IBD differed notably compared with other results (point estimate 0.62), but study design may be a contributing factor.^75^ Statistical heterogeneity was notable for CAD EYE and GI Genius analyses.

Advanced adenomas may be higher-risk for CRC compared with non-advanced adenomas; however, increased non-advanced adenoma detection is also valuable, as they may develop into advanced adenomas.^90^ The results suggest that CADe systems may impact non-advanced ADR more compared with advanced ADR, as advanced ADR analyses lacked statistical significance (point estimates ranged from 1.00 to 1.35 and 1.31 to 1.45, respectively). However, event rates were lower for non-advanced ADR, given the outcome was reported by very few studies, which is unexpected given non-advanced adenomas are typically more common than advanced adenomas.^91^ Furthermore, statistical heterogeneity is noted for most analyses (online supplemental file 2). The number of colonoscopies analysed ranged between 995 and 9683 (advanced ADR; four of eight CADe systems) and between 312 and 2445 (non-advanced ADR; three of eight CADe systems).

Regarding ADR for specific size categories, the number of colonoscopies analysed varied; larger numbers were generally available for CAD EYE and GI Genius analyses, and no data were available for three CADe systems. The largest impact of CADe was expected to be for smaller polyps, although increased detection of these may be valuable given the potential to develop into larger adenomas and, subsequently, CRC.^92^ Results suggest that the impact of the CADe systems on ADR may be greater for smaller compared with larger adenomas (online supplemental file 2); however, this is uncertain given the same trend was not consistent across interventions, and the event rate drops substantially for the larger size categories, potentially impacting the ability to detect differences. Furthermore, even for the smallest size categories, many analyses were not statistically significant.

#### Adenomas per colonoscopy

**Figure 2:**
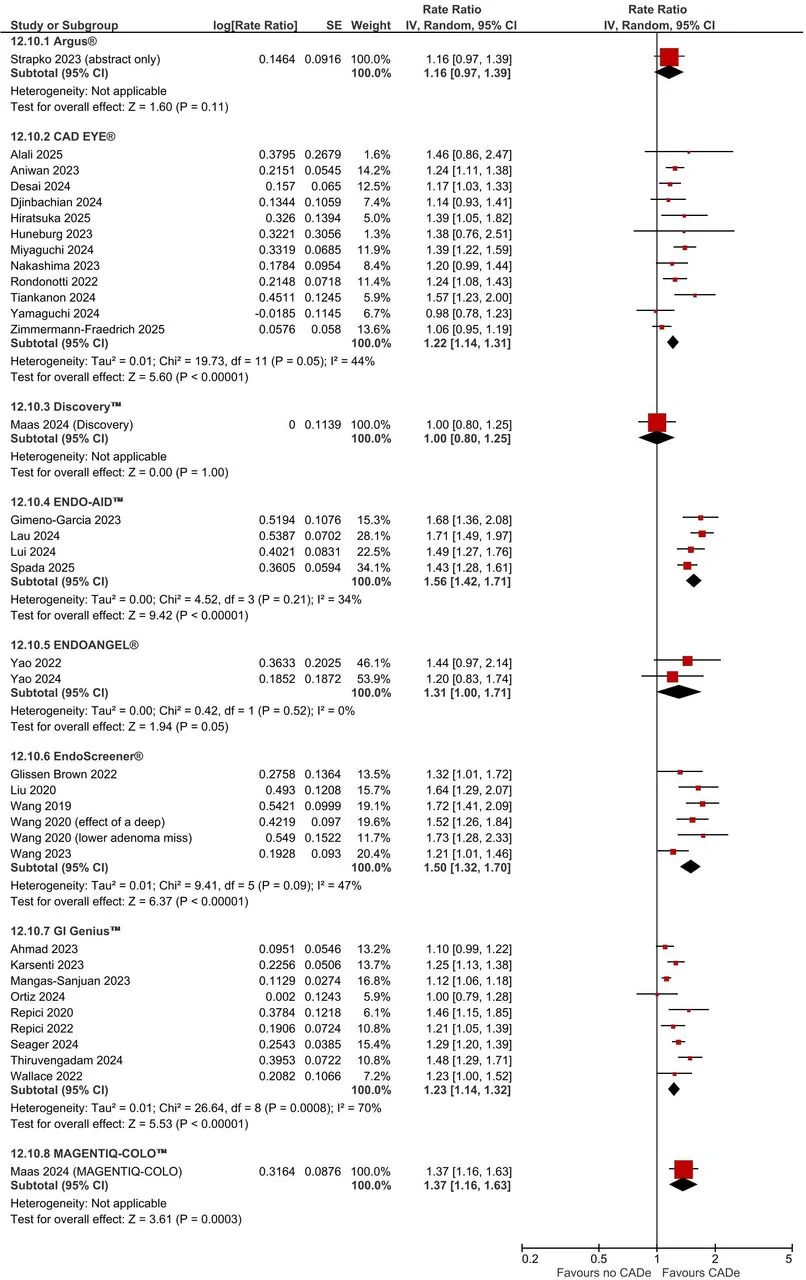
Meta-analyses of overall APC for each individual CADe system—analysed as an IRR. APC, adenomas per colonoscopy; CADe, computer-aided polyp detection; IRR, incidence rate ratio; IV, inverse variance.

APC captures the total number of adenomas detected in all colonoscopies divided by the total number of colonoscopies. It is an alternative adenoma detection measure, with increased detection and removal likely to reduce downstream CRC incidence.^93^ The number of colonoscopies analysed for each individual CADe system was similar to the ADR. Point estimates from IRR analyses indicated an increase in APC with each individual CADe system (statistically significant for five CADe systems; point estimates ranged from 1.00 to 1.56; figure 2). Statistical heterogeneity was notable for most meta-analyses. Results from mean difference analyses were largely consistent with these results (online supplemental file 2).

#### Adenoma miss rate

**Figure 3:**
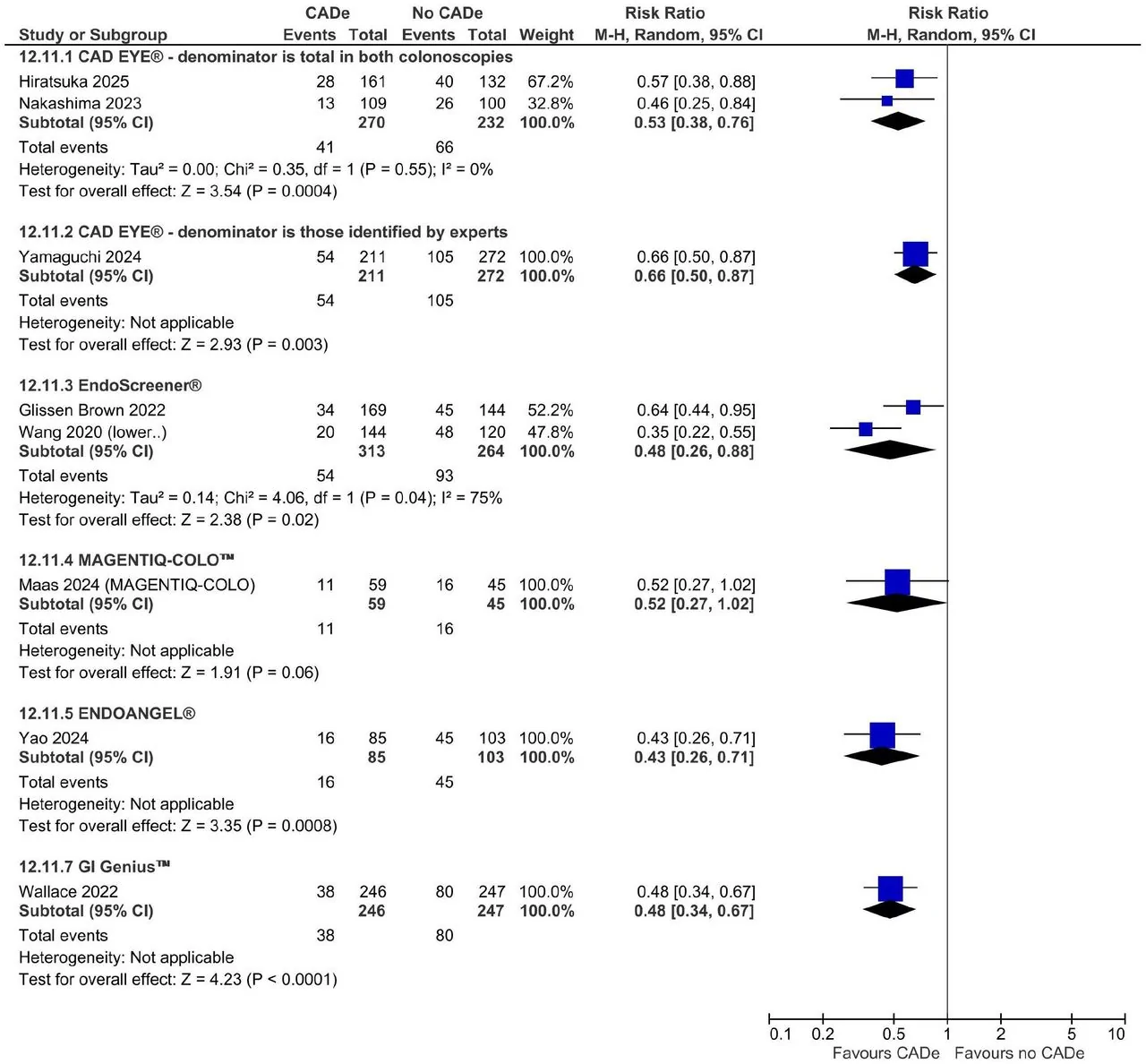
Meta-analyses of AMR for each individual CADe system. Note: the denominator is the total adenomas identified in both colonoscopies, unless stated otherwise. AMR, adenoma miss rate; CADe, computer-aided polyp detection; M-H, Mantel-Haenszel.

A reduced adenoma miss rate (AMR) with CADe would be valuable, given fewer adenomas with the potential for CRC development are missed. Only 1–3 tandem studies for each specific CADe system reported this outcome (data unavailable for three CADe systems). The number of colonoscopies included ranged from 230 to 916. Most defined AMR as the total number of adenomas identified on the second colonoscopy divided by the total number of adenomas identified in both procedures. The five CADe systems with AMR data are likely to reduce AMR versus colonoscopy without CADe (four statistically significant; figure 3). Point estimates ranged from 0.43 to 0.66. Results from three studies reporting per-patient AMR (patients with at least one missed adenoma) were similar (online supplemental file 2).^69 82 86^

#### SSL and significant polyp detection rate

**Figure 4:**
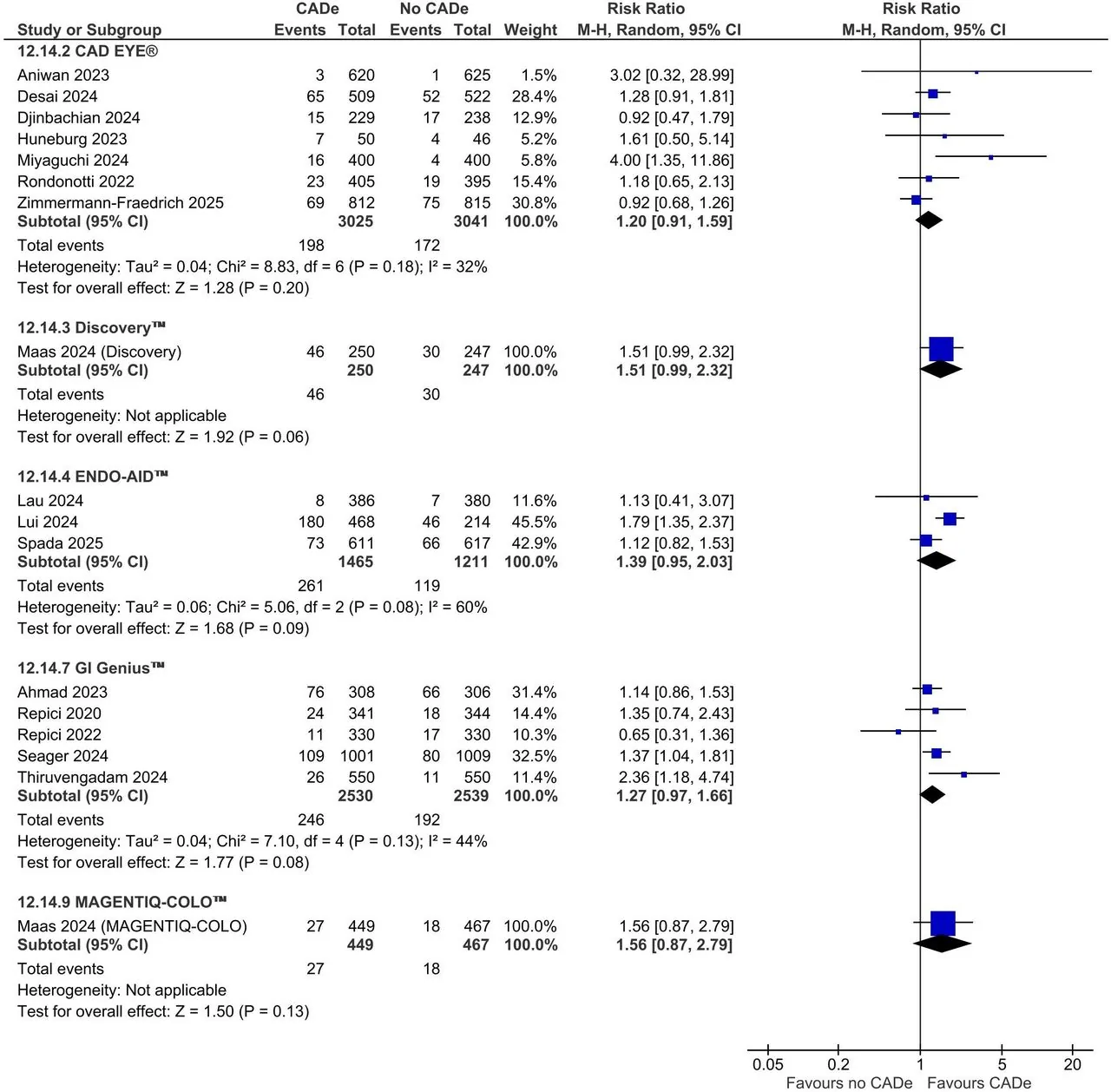
Meta-analyses of SSL DR for each individual CADe system—risk ratio analyses. Note: this figure includes results for analyses performed as a risk ratio, given event rates for the analyses were >1.0%. CADe, computer-aided polyp detection; DR, detection rate; M-H, Mantel-Haenszel; SSL, sessile serrated lesion.

**Figure 5:**
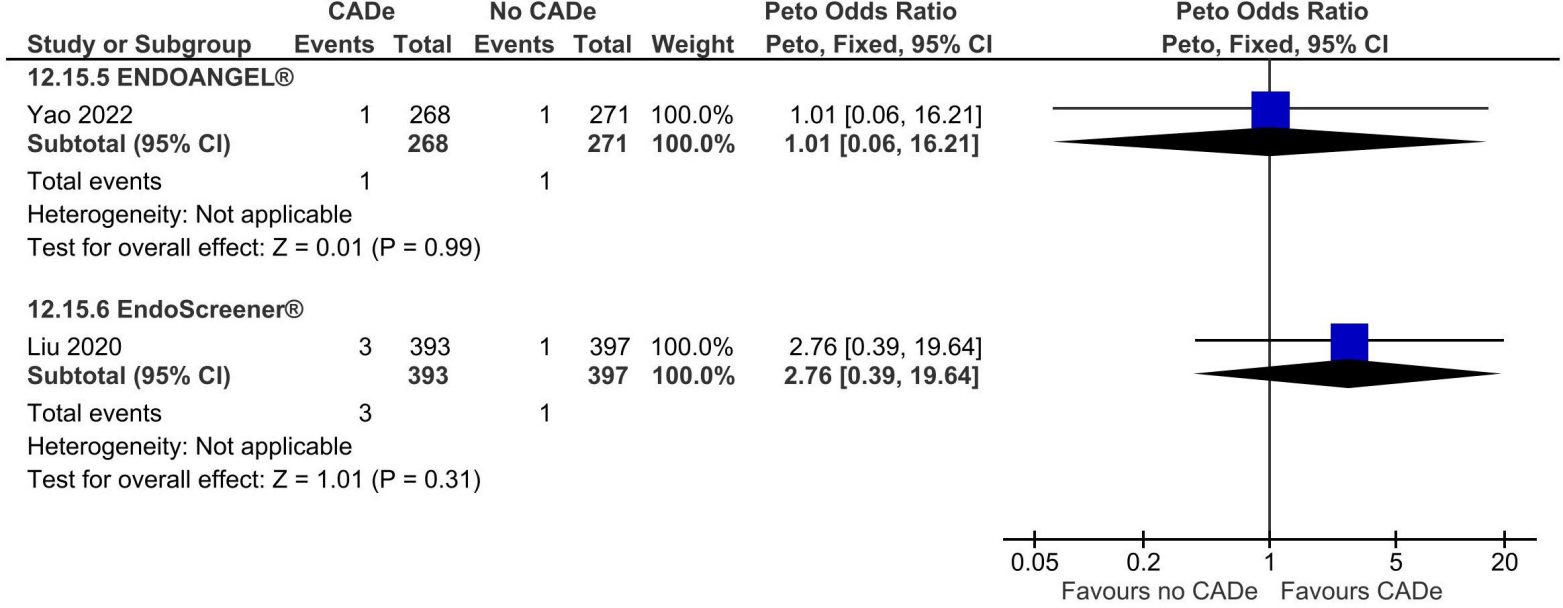
Meta-analyses of SSL DR for each individual CADe system—Peto OR analyses. Note: this figure includes results for SSL DR analyses performed as a Peto OR (ENDOANGEL and EndoScreener), given event rates for the analyses were <1.0%. CADe, computer-aided polyp detection; DR, detection rate; OR, odds ratio; SSL, sessile serrated lesion.

SSLs can also develop into CRC, meaning increased detection and removal of these lesions with CADe would be beneficial.^94^ Figures 4,5 indicate that seven separate CADe systems may improve the SSL detection rate (colonoscopies with at least one SSL detected) versus colonoscopy without CADe, although less so for ENDOANGEL. RR point estimates ranged from 1.20 to 1.56 across individual CADe systems, and Peto OR point estimates for two CADe systems with low event rates were 1.01 and 2.76. The number of colonoscopies analysed ranged between 497 and 6066. Results are more uncertain than ADR results, with considerable statistical heterogeneity across all analyses and no statistically significant differences, potentially linked to lower event rates. Results from one GI Genius study (n=614) reporting a statistically significant polyp detection rate (colonoscopies with at least one adenoma or SSL) were similar to ADR results (online supplemental file 2).

#### Hyperplastic/non-neoplastic polyp detection rate

Hyperplastic/non-neoplastic polyps are not considered to have the same potential for progression to CRC as neoplastic polyps,^95^ and increased detection with CADe may be of limited clinical value. Results for four separate CADe systems indicate that these systems may each increase the detection of non-neoplastic polyps, although not all analyses aligned with this; some lacked statistical significance and fewer studies reported this outcome. Point estimates ranged from 0.90 to 1.51 across different CADe systems and outcome definitions (online supplemental file 2). The number of colonoscopies analysed ranged between 539 and 2523.

### CADx outcomes

Nine studies (for three separate CADx systems) assessing CADx diagnostic accuracy were included. Evidence is limited, and results are mixed; some studies suggest improved sensitivity with CADx versus endoscopist optical diagnosis alone, while others suggest the opposite or no clear difference, and some do not report this comparison. Further limitations include autonomous CADx use, SSLs treated as non-neoplastic polyps and/or the exclusion of low-confidence diagnoses. Results for the assessment of different polyp categories are presented in online supplemental file 2. Sample size ranged between 61 and 630 lesions across the included CADx evidence. Overall, no strong conclusions were considered possible.

### Procedural duration and adverse events

Results for withdrawal time (five CADe systems) and total procedure time (three CADe systems) suggest potential for increased procedure duration with each of these systems, although limited to around 1–2 min per colonoscopy. Withdrawal time and procedural time point estimates ranged from 0.12 to 0.81 and 0.40 to 1.47 min longer with CADe, respectively (online supplemental file 2). CADx studies did not report procedure duration outcomes. No obvious concerns about any CADe or CADx system increasing adverse events (AEs) were identified (online supplemental file 2), but the extent of AE monitoring was often unclear.

### Subgroup analyses

Colonoscopy indication and endoscopist experience subgroup analyses were performed for ADR and APC. However, limitations mean that they are considered to be exploratory and uncertain, with no robust conclusions possible. Differences in definitions existed between trials, and many within-trial subgroup analyses were not stratified at randomisation. Various ways of grouping studies by endoscopist experience were explored based on feedback from clinical experts (online supplemental file 2), including by baseline ADR requirements for trial inclusion, but reporting of this across studies was limited. Furthermore, the Cochrane Handbook for Systematic Reviews of Interventions recommends at least 10 studies for each category for reliable subgroup analysis, a criterion not met here.^33^

Despite some trends within specific trials or analyses, such as larger ADR improvements for less experienced endoscopists or for those indicated due to symptoms compared with screening or surveillance colonoscopy, a consistent pattern across analyses was not observed, and no robust conclusions are considered possible. See online supplemental file 2.

Some Lynch syndrome studies were captured as a separate subgroup in the colonoscopy indication analyses. However, only one study each for two technologies was identified, limiting the conclusions possible; GI Genius analyses highlight Ortiz *et al* as a potential outlier with a negative impact (non-significant) of GI Genius on ADR (online supplemental file 2), but a similar result was not observed for Huneburg *et al* in the CAD EYE analysis (online supplemental file 2).^40 77^

## Discussion

This research assessed the impact of eight CADe/CADx systems on colonoscopy outcomes. Results suggest that each individual CADe system may increase ADR versus colonoscopy without CADe, although results were not statistically significant for two systems. Evidence that some CADe systems may increase the detection of other polyp categories (including advanced and non-advanced adenomas, different-sized adenomas, SSLs and non-neoplastic/hyperplastic polyps) and reduce AMR was also identified. However, these conclusions are more uncertain due to fewer statistically significant differences and reporting by fewer studies. Individual CADe systems may have a small impact on procedure duration, but the impact of CADx systems is unclear. No concerns about AEs with different CADe or CADx systems were identified.

These conclusions broadly align with previous CADe meta-analyses,^11 18–21 96^ but this research adds value with meta-analyses stratified by each individual CADe system from the outset. Unique algorithms for each CADe system mean the impact on detection outcomes and downstream clinical outcomes, such as interval CRC, could vary. While most CADe systems may increase ADR and other detection outcomes and reduce AMR, uncertainty is higher for some systems or for certain outcomes (such as SSLs) based on non-statistically significant differences, which may be partially explained by fewer studies or events for analysis. For example, for ADR, only one study was available for Argus and Discovery, with at least 10 available for CAD EYE and GI Genius.

CADx diagnostic accuracy data were available for three CADx systems, but robust conclusions were not possible given the mixed results observed, limited comparison to endoscopist optical diagnosis alone, frequent classification of SSLs as non-neoplastic and/or exclusion of low-confidence diagnoses. This research may be among the first to focus on the CADx evidence most relevant to clinical practice, including in vivo CADx application and adjunct use. Many earlier CADx reviews include studies applying CADx systems to videos and photographs of colonoscopies (ie, ex vivo use).^9 19 22^ These studies do not reflect their intended use in clinical practice; ex vivo use does not capture conditions during real-time colonoscopies, for example, the interaction between the CADx system and the endoscopist during the procedure. Prioritisation of adjunct CADx use was implemented, given this best reflects its intended use; manufacturers recommend adjunct use and autonomous CADx results may not accurately reflect this.

Two recent CADx meta-analyses for diminutive rectosigmoid polyps concluded no incremental benefits or harms of CADx-assisted colonoscopy compared with colonoscopy without CADx for resect-and-discard or leave in situ strategies.^24 25^ While useful for assessing CADx overall in this context, combining different CADx systems and focusing on high-confidence diagnoses was not appropriate for the aims of this research, informing a NICE HealthTech Evaluation. Algorithm differences mean there is clear potential for different CADx systems to have different accuracies, and excluding low-confidence diagnoses does not allow the overall accuracy of the CADx system to be assessed; insight into whether adjunct CADx use leads to incorrect polyp characterisations, even if made with only low confidence, is important in assessing whether CADx may benefit or hinder endoscopist optical diagnosis, particularly given the confidence assessment is partially subjective.^97^ The proportion of low-confidence diagnoses with CADx may also be important for future cost-effectiveness analyses of CADx, where specific polyp management strategies are implemented, given confidence will impact whether or not histopathological testing is required.^98 99^ These approaches towards CADx allow the current evidence gaps and limitations to be highlighted, which future research may address.

The lack of long-term data with the use of these AI systems, such as on mortality and interval CRC, is a current limitation. Studies focus on within-colonoscopy outcomes and do not follow patients to assess the impact on downstream clinical outcomes. Therefore, conclusions about whether these systems will improve clinical outcomes can only be inferred indirectly through reported links between increased ADR and reduced CRC incidence.^88 89^ Furthermore, the exploratory subgroup analyses herein do not allow a robust assessment of potential differences in effect across colonoscopy indication or endoscopist experience subgroups. Other recent CADe assessments did not identify clear differences for specific subgroups.^17 18 100^

This research only covers CADe/CADx systems available in the UK, given it was performed within a NICE assessment. Furthermore, data for some systems were provided confidentially for the NICE assessment only and could not be reported here. Comparisons between individual CADe/CADx systems are not performed, given this was not an aim of the NICE assessment; this would also have required indirect treatment comparisons, potentially difficult to conduct and interpret given the wide variation in study characteristics. Conclusions about the impact of CADe on advanced compared with non-advanced ADR are limited, given event rates within meta-analyses were unexpectedly lower for non-advanced ADR. This is likely explained by relatively few studies reporting non-advanced ADR, given trials reporting both supported the clinical expectation that non-advanced adenomas are more common.^56 62 78 79 81 91^ Concerns about ongoing software developments for CADe/CADx systems, potentially making earlier trials less representative of the current systems, exist. This was not explored formally herein, as the version number was rarely reported. The authors acknowledge this uncertainty but consider that new updates would most likely improve rather than worsen performance.

Further research from CADx diagnostic accuracy studies addressing the limitations noted herein and RCTs/other comparative studies reporting data on long-term outcomes with CADe/CADx use compared with colonoscopy without CADe/CADx would be of value. For CADx, future studies should use CADx adjunctly alongside endoscopist judgement, categorise SSLs as neoplastic, include a comparison against endoscopist optical diagnosis alone, include an analysis where both high- and low-confidence diagnoses are included and report high-confidence versus low-confidence assessments with and without CADx. Studies with suitable stratification at randomisation and larger sample sizes would provide more robust data to analyse potential differences across colonoscopy indication and endoscopist experience subgroups, and studies focusing on key populations commonly excluded from trials (such as IBD, polyposis syndromes and those with prior CRC) would be beneficial. For endoscopist experience subgroup analyses, clinicians involved in this work highlighted that trials stratifying by baseline ADR ≤40% and >40% may be particularly useful; while a subgroup analysis categorising endoscopist experience using these thresholds based on study inclusion criteria was performed herein, reporting of this was limited across studies. Therefore, future research might explore alternative methods of categorising based on baseline ADR. One method might be based on the within-trial ADR reported for the non-CADe arm; however, analyses based on within-trial performance, rather than prior to the trial, may be limited given the potential for the Hawthorne effect mentioned earlier.^87^

## Conclusions

The individual CADe systems covered herein are likely to increase the detection of adenomas and other polyp types during colonoscopy compared with colonoscopy without CADe, although the evidence is more uncertain for some polyp subtypes, with potential for a small increase in procedure duration and no notable impact on AEs. The results for ADR, a key outcome, were not statistically significant for Argus and Discovery, potentially explained to some extent by fewer studies and reduced sample sizes. No clear evidence of a differential impact across colonoscopy indications and levels of endoscopist experience was identified, but further research assessing this is required. Evidence in specific groups at a higher risk of CRC, such as those with IBD or polyposis syndromes, is also lacking.

The CADx evidence is uncertain, and further research addressing the current limitations (including using the CADx systems alongside clinician judgement, analysing SSLs as neoplastic, comparing against endoscopist optical diagnosis alone and considering both high- and low-confidence diagnoses with and without CADx) is required. No evidence for CADe/CADx impact on long-term outcomes such as interval CRC and mortality is currently available; such data will be key to confirming any downstream effect of the CADe/CADx systems.

## Data Availability

Data are available upon reasonable request. All data relevant to the study are included in the article or uploaded as supplementary information. Additional information on the methods and results of this research has been provided in the online supplemental files.
