# Supplementary material for: Computer-aided polyp detection and characterisation systems to support colonoscopy: a systematic review with results stratified by each individual artificial intelligence system
Source: BMJ Digit Health Ai. 2026 Jun 22;2(1):e000046. doi: 10.1136/bmjdh-2026-000046 (PMC13528337; doi:10.1136/bmjdh-2026-000046)
Supplement: Supplementary file 1 [file bmjdh-2-1-bmjdh-2026-000046-s001.pdf]

Artificial intelligence software to help detect and characterise colorectal polyps [DAP78]

Final Protocol

October 2024

Source of funding

This report was commissioned by the NIHR Evidence Synthesis Programme on behalf of the National Institute for Health and Care Excellence. NIHR Reference No. 136019.

NIHR reference no.	136019
Final amended version date	23/10/2024
Title of the project:	Artificial intelligence software to help detect and characterise colorectal polyps [DAP78]
External assessment group:	BMJ Technology Assessment Group (BMJ-TAG)
Project lead:	Steven J Edwards, Director of Health Technology Assessment, BMJ-TAG, BMJ Group, 5th Floor, 90 Whitfield Street, London W1T 4EZ, UK.
Declared competing interests of the authors	No competing interests were declared which affect the impartiality of this assessment. BMJ Technology Assessment Group (BMJ-TAG) and the editorial team of The BMJ work independently to one another. The views and opinions expressed in this document are those of the BMJ-TAG.
Rider on responsibility for report:	The views expressed in this protocol are those of the authors and not necessarily those of the NIHR Evidence Synthesis Programme. Any errors are the responsibility of the authors.

1 Plain English Summary

Colorectal cancer (CRC), also known as bowel cancer, includes cancer of the colon or rectum.[1] It is the fourth most common cancer in the UK, with over 44,000 new cases each year, and accounts for 10% of all cancer-related deaths in the UK. It is the second most common cause of cancer-related death; the earlier it can be diagnosed, the better the chances of survival.[2-4]

Colorectal polyps are small growths on the lining of the colon or rectum that are usually asymptomatic but may be detected on colonoscopy. Symptoms such as rectal bleeding or a change in bowel habits can occur in some cases. Most colorectal polyps are harmless, but some have the potential to turn into cancer over time if untreated.[5] Colonoscopy is the gold standard for the detection of colorectal polyps. People may undergo colonoscopy through various pathways, as summarised in the National Institute for Health and Care Excellence (NICE) final scope for this assessment.[6]

During a colonoscopy, concerning polyps can be removed. In the UK, all removed polyps are sent for histopathological testing to classify them as neoplastic or non-neoplastic. Neoplastic polyps, such as adenomas and serrated polyps, have a higher risk of developing into cancer. Some polyps may need to be removed during a separate procedure, and endoscopists may choose not to remove certain polyps if they are confident they are not neoplastic. A “resect and discard” strategy, where an optical diagnosis by the endoscopist would suffice without histopathological testing, is in its early stages of use within the Bowel Cancer Screening Programme (BCSP).

In recent years, artificial intelligence (AI)-supported colonoscopy technologies have been developed. These include computer-aided detection (CADe) and computer-aided diagnosis (CADx). CADe aims to reduce missed polyps by flagging areas of interest to for review, while CADx involves characterising detected polyps to suggest whether they are likely neoplastic, supporting decisions about resection. This may reduce resections of non-neoplastic polyps and increase neoplastic polyp resection.

This assessment will review the evidence for the clinical and cost-effectiveness of specific AI-supported colonoscopy technologies, including CADe and CADx. The systematic literature review (SLR) will aim to identify evidence related to clinical effectiveness, diagnostic accuracy, safety and other outcomes. Previous economic evaluations will be reviewed, and a new economic model may be developed to assess whether the technologies represent a good use of NHS resources, where sufficient data are available.

2 Decision problem

2.1 Purpose of the decision to be made

The main research question to be addressed is: "Does the addition of AI-supported colonoscopy technologies to colonoscopy represent a clinically- and cost-effective use of NHS resources?"

To answer this question, an SLR will be performed to collect and synthesise data on the clinical effectiveness, diagnostic accuracy and safety of the AI-supported colonoscopy technologies outlined in Section 2.2 below. A review of existing economic analyses of these technologies will also be performed, with a *de novo* health economic model developed if no existing models that could be used are identified. Sections 3 and 4 provide more details on the methodology that will be used in this assessment.

2.2 Clear definition of the intervention

The following 11 technologies have been identified for inclusion in this assessment as part of the National Institute for Health and Care Excellence (NICE) scope. These technologies are summarised in Supplementary Table 1 below:

- Argus® (Endosoft);
- CAD EYE® (Fujifilm Healthcare UK Ltd.);
- CADDIE™ (Odin Vision);
- Discovery™ (Pentax Medical UK);
- ENDO-AID™ (Olympus Medical Systems Corp.);
- ENDOANGEL® Lower Gastrointestinal Endoscope Image Auxiliary Diagnostic Equipment(Wuhan ENDOANGEL Medical Technology Co. Ltd.);
- Endoscopic Multimedia Information System (EMIS™; EndoMetric Corporation);
- EndoScreener® (Wision AI);
- GI Genius™ (Medtronic);
- MAGNETIQ-COLO™ (MAGNETIQ-EYE);
- WISE VISION® (NEC Corporation).

The External Assessment Group (EAG) will only consider these specific technologies in this assessment and will exclude data on any other technologies that may be identified through the literature search.

It should be noted that some technologies may be excluded from use in certain populations. In addition, it is possible that the skill and experience of the endoscopist may impact the potential benefits associated with AI-supported technologies. The intention is that the technologies are incorporated into the usual colonoscopy procedure, with the final decision about whether or not polyps are removed made by the endoscopist. These factors will be considered in the assessment as outlined in the methods described in Section 3.2.

Supplementary Table 1. Summary of AI technologies included in this assessment as per the NICE scope

Name of technology (manufacturer)	Classification	Function	Intended use
Argus® (Endosoft);	Regulatory approval in process	CADe	Described as a CADe device used in endoscopy to detect abnormal lesions within the GI tract. The device draws attention to images to help with the detection of lesions. It has hardware components that support interfacing with an endoscope.
CAD EYE® (Fujifilm Healthcare UK Ltd.)	CE class IIa	CADe and CADx	The software detects and characterises areas that are suspected to be colonic polyps in an endoscopic video image from an endoscopic video processor. Results in detection or characterisation modes are presented onto the endoscopic video image in real-time. Characterisation mode includes suggestions about whether a suspected colonic polyp is neoplastic or hyperplastic. It is intended for use as a support for diagnosis during colonoscopy under the supervision of medical professionals.
CADDIE™ (Odin Vision)	CE/UKCA marked (based on company website, accessed 23 August 2024)	CADe and CADx	Full statement on intended use not available from the company at the time of protocol development but the product brochure, available on the company's website (accessed 23 August 2024 accessed via the company's website) highlights a role for the product in supporting endoscopists with the detection and characterisation of colorectal polyps in real-time during colonoscopy..
Discovery™ (Pentax Medical UK)	CE class I	CADe	Providing assistance to endoscopists for identification of polyps during colonoscopy; not intended to make or recommend decisions about patient management, diagnosis or therapeutic interventions.
ENDO-AID™ (Olympus Medical Systems Corp.)	CE class I	CADe	Providing assistance to physicians for detection of mucosal abnormalities, such as possible colorectal polyps, during colonoscopy. It is an adjunctive technology and should not be used as a stand-alone method for detection of abnormalities. The system processes signals from the endoscopy video system centre and directs the user's attention to areas of interest for assessment.

ENDOANGEL® Lower Gastrointestinal Endoscope Image Auxiliary Diagnostic Equipment(Wuhan ENDOANGEL Medical Technology Co. Ltd.)	Uncertain	CADe	Full statement on intended use not available from the company at the time of protocol development but the company's website (accessed 28 August 2024) describes ENDOANGEL® as a CADe system for polyps powered by AI. It can be used for polyp identification in the lower digestive tract during endoscopy. It is not intended to replace clinical decision making and results should only be used as a reference..
Endoscopic Multimedia Information System (EMIS™; EndoMetric Corporation);	Regulatory approval in process	CADe	Full statement on intended use not available from the company at the time of protocol development but the company has indicated that the technology has a CADe function.
EndoScreener® (Wision AI)	CE class II	CADe	Full statement on intended use not available from the company at the time of protocol development but the company's website (accessed 23 August 2024) describes it as a CADe device for colorectal polyps. It uses colonoscopy video stream as the input from an endoscopy device and analyses it in real-time. Output from EndoScreener® involves blue boxes being overlaid onto colonoscopy images to highlight potential polyps.
GI Genius™ (Medtronic)	CE class IIb	CADe and CADx	Described as an AI-based medical device which processing colonoscopy images containing regions consistent with colorectal lesions such as polyps, including those with flat (non-polypoid) morphology. It is intended for use by trained clinicians using while-light colonoscopy to highlight regions with visual characteristics consistent with different types of mucosal abnormalities (such as colorectal polyps). The intended target population is any person undergoing a colonoscopy.<br>Characterisation support can be enabled, which enables the software to suggest the possible polyp histology to the user, which includes "adenoma", "non-adenoma" or "no prediction". No prediction is returned when the system is not confident enough to suggest a potential histology.<br>It should be used as an adjunct to colonoscopy and should not replace endoscopist judgement or histopathological assessment.

MAGENTIQ-COLO™ (MAGENTIQ-EYE)	CE class I	CADe	Providing assistance to endoscopists performing colonoscopies by assisting with the detection of lesions by highlighting regions with visual characteristics consistent with different mucosal abnormalities that may be seen during a colonoscopy. Identified lesions should be independently assessed by the endoscopist and action taken according to standard clinical practice. It should be used as an adjunctive tool and should not replace histopathological assessment.
WISE VISION® (NEC Corporation)	CE class IIa and UKCA class IIa	CADe and CADx	The polyp detection function (Ce3.0) involves analysis of video signals from endoscopic equipment to assist endoscopists in identifying potential colorectal polyps. It draws the endoscopist's attention to potential polyps.<br>The polyp characterisation feature (Cx3.0) analyses the polyps identified by the endoscopists and suggests whether they are likely to be neoplastic or non-neoplastic. It is designed to support endoscopists in making an optical diagnosis during colonoscopy.<br>The technology can also assist with polyp sizing, by categorising polyps identified by endoscopists as diminutive (≤5 mm) or non-diminutive (≥6 mm).<br>It is intended for use in any person undergoing colonoscopy, but people with bowel inflammation (UC or GVHD-related bowel inflammation), familial adenomatous polyposis or with a history of chemotherapy or radiation therapy for targeted CRC are excluded.
Abbreviations: AI, artificial intelligence; CADe, computer-aided detection; CADx, computer-aided diagnosis; CRC, colorectal cancer; GHVD, Graft-versus-host disease; GI, gastrointestinal; NICE, National Institute for Health and Care Excellence; UC, ulcerative colitis; UKCA, UK Conformity Assessed.			

2.3 Population and relevant sub-groups

The population of interest in this assessment is people undergoing colonoscopy because they have been referred for investigation via one of the following pathways:

- Colonoscopy through the NHS BCSP;
- Colonoscopy for investigation of symptoms suggestive of CRC;
- Surveillance colonoscopy because of a hereditary risk of CRC;
- Surveillance colonoscopy because of inflammatory bowel disease (IBD);
- Surveillance colonoscopy post-polypectomy or post-CRC resection.

Where data allows, subgroup analyses based on these populations will be explored, as outlined in Section 3.1. Other methods relating to the population in this assessment are also described in Section 3.1.

2.4 Place of the intervention in the treatment pathway

AI-supported colonoscopy technologies will be used in real-time during colonoscopies in the secondary care setting with the aim of increasing the detection of polyps compared to colonoscopy without the use of AI-supported technologies, or assisting with the diagnosis of polyps to aid decisions about whether or not to resect them and send for histological assessment. They are not intended to be used alone but as an adjunct to support endoscopists during colonoscopies; they should not replace the clinical judgement of endoscopists.

2.5 Relevant comparators

The comparator of interest in this assessment is colonoscopy performed without the use of AI-supported colonoscopy technologies. This may be with or without the use of virtual chromoendoscopy (VCE), dye-based chromoendoscopy or Endocuff Vision™. Methods that will be used in this assessment relating to the comparators are described in Section 3.3.

2.6 Outcomes

Relevant outcomes outlined in the NICE final scope include the following, with further details on the methods to be used in this assessment outlined in Sections 3.4, 3.5 and 4.

Intermediate measures:

- Measures of ability or accuracy to detect polyps or cancer;
- Measures of ability to characterise identified polyps;
- Measures related to healthcare resource use (such as time to do colonoscopy, need for repeat colonoscopy to be done, need for a second observer);
- Time to colonoscopy and impact on waiting lists;
- Number of polyp removal procedures;
- Incidences that the technology does not function;
- Impact on decision making;
- Ease of use/acceptability of the technologies to healthcare professionals.

Clinical outcomes:

- Morbidity (including outcomes related to the colonoscopy procedure and cancer, such as incidence of post-colonoscopy CRC);
- Mortality.

Patient-reported outcomes:

- Health-related quality of life (including anxiety);
- Acceptability of tests to patients.

Cost outcomes:

- Costs of AI systems (including any software, hardware, consumables, maintenance, and service costs);
- Cost of training;
- Costs related to colonoscopy and polyp removal;
- Costs of histopathology;
- Cost of treatment for CRC;
- Costs of adverse events (AEs) from the procedure or further diagnostic work up.

2.7 Key factors to be addressed

Other issues for consideration outlined in the NICE final scope are listed below, with details of how these will be accounted for in the assessment report by the EAG. In addition to these, the EAG highlights some other factors that may be important to consider.

Impact of endoscopist skill and experience

There may be differences in the skill and experience level of endoscopists trained through different programmes; for example, for those trained through the BCSP compared to those that are not. Bowel Cancer Screener Accreditation (BSCA) also differs in England and Wales compared to Scotland, meaning skill and experience may differ across the UK nations even within the screening setting. England and Wales also have a programme in place to train suitably qualified registered health professionals to perform colonoscopies (Health Education England and Health Education and Improvement Wales). Clinical expert feedback as part of the scoping workshop for this project highlighted differences in the surveillance programmes for different populations across the UK

nations; for example, people with Lynch syndrome in England have a surveillance programme as part of the BCSP but in other UK nations this may not always be performed by screening-accredited colonoscopists. In addition, in England, there may be variation in terms of whether hereditary high-risk (including people with polyposis) have their colonoscopies performed by screening-accredited colonoscopists.

There is some evidence from the literature that CADe may have less of a benefit in the BCSP setting where procedures are performed by experienced endoscopists.[7] Therefore, the extent that AI is able to improve the detection or characterisation of polyps when added to colonoscopy may vary depending on the skill and experience of the endoscopists. Where data allows, differences in outcomes based on endoscopist skill or experience will be explored using subgroup and sensitivity analyses. Input from the EAG's clinical experts will be used to inform how to divide studies based on endoscopist skill and experience as reported in the trials, which is likely to be defined differently across trials, and criteria outlined by the Joint Advisory Group in Gastrointestinal Endoscopy (JAG) will be considered if studies report these details.

Performance variation by reason for colonoscopy

The NICE scope highlights clinical expert feedback that algorithms included as part of the AI-supported colonoscopy technologies may not have been developed, trained or validated on data from people with IBD or hereditary risk factors, meaning there is some concern about the performance of the technologies in these populations. Furthermore, the amount of evidence available for each population may differ, given some populations such as those with IBD are often excluded from trials. The EAG will comment on any populations within the NICE scope for which there is limited or no evidence and will consider whether generalisation of data from other populations are suitable for these populations, with feedback from its clinical experts considered. It will also explore differences between these populations if data allows using subgroup and sensitivity analyses, as outlined in Section 3.1.

Workforce and capacity issues

Increases in polyp detection may lead to polypectomies being performed more frequently, which would increase the workload of gastroenterologists and histopathologists. This could increase capacity challenges further and increase wait times for colonoscopies. The NICE final scope indicates that, "outputs of modelling should include an indication of the estimated change in numbers of

colonoscopies, as well as other healthcare procedures such as polypectomies and those related to histopathology"; the EAG anticipates that this will be captured as part of its economic modelling. An analysis of the impact of introducing AI technologies on waiting times may also be incorporated within the model, if the change in the number of colonoscopies is considered to be substantial, and if sufficient data are available to inform this. The broader impact of adoption of the technologies of interest on workforce and capacity will be discussed narratively, although detailed modelling will not be conducted as part of this assessment.

Existing guidance on the use of AI-supported detection and diagnosis in colonoscopy

Guidance from the European Society of Gastrointestinal Endoscopy (ESGE, 2019) suggests potential incorporation of computer-aided diagnosis (detection and characterisation of lesions) to colonoscopy if *in vivo*, high-quality multicentre clinical studies demonstrating acceptable and reproducible accuracy for colorectal neoplasia are available.[8] It highlights that significant risks associated with implementation may be endoscopist deskilling and over-reliance on AI, unrepresentative training datasets and hacking. The ESGE also published a position statement on the expected value of AI in gastrointestinal endoscopy in 2022.[9] For acceptance of AI in CADe and CADx, it recommends the following:

- For AI-supported colorectal polyp detection (CADe), the AI-supported adenoma detection rate (ADR) should be "comparable to that of experienced endoscopists";
- For AI-supported optical diagnosis (CADx) of diminutive polyps (≤5 mm), AI-supported characterisation should "match performance standards for implementing resect and discard and diagnose and leave strategies";
- For the management of polyps ≥ 6 mm, AI-supported characterisation should be "comparable to that of experienced endoscopists in selecting lesions amenable to endoscopic resection".

Health Technology Wales (HTW) guidance on AI-assisted endoscopy in the detection of gastrointestinal cancer and pre-cancerous lesions recommends routine adoption of CADe colonoscopy for detecting lower gastrointestinal cancer and pre-cancerous lesions.

The EAG will review available guidance and ensure that additional concerns raised within these resources are discussed in its report. Studies cited within this guidance will be reviewed to identify

additional studies relevant for inclusion in this review. It will also highlight any additional concerns or benefits in relation to these technologies raised by clinical experts consulted by the EAG.

Impact of CADx function of tests

The NICE scope outlines that the usefulness of CADx may depend on how resect and discard strategies are used within the NHS; currently, experts have advised that the use of this may be limited but that a trial has recently started within the BCSP. The EAG intends to explore the use of resect and discard strategies within its economic model where data permits (Section 4.3).

Variation in the versions of the technology used within clinical trials

The EAG considers that it is likely that different studies may use different versions of the technologies compared to the most up to date version or compared to other trials, for example due to software updates. Information on whether the same software version was used in all patients within a trial, or whether updates to software occurred during trials, was noted as an important factor to consider during the scoping workshop. The EAG will highlight any concerns in its report based on information available from the evidence identified or additional information provided by the companies. Trials will not be excluded based on the version of the technology used but this may be explored as a potential source of heterogeneity within analyses (for example, by performing sensitivity analyses) if this information is consistently reported within the trials.

Population datasets used to train technologies may differ to the UK population

There may be concerns about the applicability of technologies to UK populations if data used to train the AI technologies deviate substantially from the UK population. The EAG will extract this information where available from the evidence identified, as well as any additional information provided by the companies, and highlight any concerns in its report.

Limited data for some outcomes

The EAG considers it likely that evidence identified in the SLRs for certain outcomes in the NICE final scope will be limited or non-existent. Where possible, the EAG will perform supplementary searches or estimate outcomes using the economic model or clinical expert feedback, but this may not be possible for all outcomes. For example, it may be particularly difficult to identify evidence on ease of use/acceptability of technologies to healthcare professionals and patients.

Furthermore, the EAG considers the availability of evidence for AI-supported polyp diagnosis in the real-time colonoscopy setting may be more limited compared to AI-supported polyp detection, meaning there may be less evidence on which to base any conclusions.

Where relevant, the impact of uncertainty for outcomes informed by limited data will be explored via sensitivity analyses in the economic model.

2.8 Areas of agreement at the scoping workshop that are outside the scope of the appraisal and therefore do not require any detailed assessment (e.g. key factors for which evidence is already accepted).

No areas were specifically excluded from the assessment but it was acknowledged that there may be limited data for certain populations identified in the scope.

3 Methods for assessing the outcomes arising from the use of the interventions

3.1 Population

The population of interest in this assessment is people undergoing colonoscopy because they have been referred for investigation via one of the following pathways:

- Colonoscopy through the NHS BCSP;
- Colonoscopy for investigation of symptoms suggestive of CRC;
- Surveillance colonoscopy because of a hereditary risk of CRC;
- Surveillance colonoscopy because of IBD;
- Surveillance colonoscopy post-polypectomy or post-CRC resection.

The EAG will analyse data for the colonoscopy population overall initially for each intervention and, where data allows, subgroup analyses based on these populations will be explored, as outlined below.

It is possible that there is a lack of evidence for certain groups covered by this population (for example, patients with IBD or patients with known or suspected polyposis are often excluded from trials in this area) and the EAG will highlight any groups for which clinical evidence is lacking or limited in its report. It will ensure that non-randomised controlled trials (RCTs) are reviewed for this subgroup data, even if there are sufficient data from RCTs for the overall colonoscopy population.

If studies span multiple subgroups (i.e. not all patients fall within a specific colonoscopy indication subgroup), data will be extracted separately for specific subgroups where available for inclusion in the subgroup analyses. Where separate data are not available, studies will be assigned to a specific subgroup if ≥80% of those included fall within that subgroup. Studies where there is not this majority will be included in the subgroup where most of the patients are categorised, but the impact of these studies on the analysis will be explored through sensitivity analyses. Studies where this is unclear or not reported will not be included in the subgroup analyses unless the information is provided following contact with study authors.

3.2 Interventions

The EAG notes that AI can be used to support the detection (CADe) or diagnosis (CADx) of polyps during colonoscopy, both of which are covered in the NICE scope. Some technologies may have both elements whereas others only have one of these functions. Evidence for each technology will be assessed separately.

The following 11 technologies have been identified for inclusion in this assessment as part of the NICE scope. Further details of these technologies are provided in Section 2.2:

- Argus® (Endosoft);
- CAD EYE® (Fujifilm Healthcare UK Ltd.);
- CADDIE™ (Odin Vision);
- Discovery™ (Pentax Medical UK);
- ENDO-AID™ (Olympus Medical Systems Corp.);
- ENDOANGEL® Lower Gastrointestinal Endoscope Image Auxiliary Diagnostic Equipment (Wuhan ENDOANGEL Medical Technology Co. Ltd.);
- Endoscopic Multimedia Information System (EMIS™; EndoMetric Corporation);
- EndoScreener® (WISION AI);
- GI Genius™ (Medtronic);
- MAGNETIQ-COLO™ (MAGNETIQ-EYE);
- WISE VISION® (NEC Corporation).

The EAG will only consider these specific technologies in this assessment and will exclude data on any other technologies that may be identified through the literature search.

The NICE final scope outlines that the intention is that the AI technologies are to be used to support rather than replace clinician judgement, with final decisions about whether or not polyps are removed made by the endoscopist. This has also been supported by clinical expert feedback provided to the EAG. Therefore, data relating to the use of AI alone for the detection or diagnosis of polyps in colonoscopy will not be prioritised for inclusion in this review. For example, studies that report the sensitivity and specificity of AI when applied alone without any endoscopist input, rather than with endoscopist input, will be excluded unless their inclusion would provide data for interventions or outcomes for which there is no evidence from studies meeting the preferred criteria. Where the assessments have been performed in real-time during a colonoscopy but the use

of AI as an adjunct to endoscopist judgement is unclear, studies will be included with additional uncertainties of this evidence highlighted and sensitivity analyses performed.

Studies that do not involve application of the AI technology during real-time colonoscopy will not be included, for example, studies where AI technologies are retrospectively applied to images or videos from colonoscopies (i.e. accuracy measures are based on *ex vivo* rather than *in vivo* application of the technology). This is because studies of this kind will not capture the impact of the colonoscopy environment on outcomes of using the technology (such as time pressures) and interactions between the technology and the endoscopist that would occur during a colonoscopy would not be captured. For example, suggestions made by the AI technology in real-time may prompt endoscopists to investigate particular areas in more detail or for longer. In addition, guidance from ESGE in 2019 is that for incorporation of AI technologies into colonoscopy, *in vivo* evidence should be available, as outlined in Section 2.7. Given the time constraints of this assessment and the rationale described, the EAG considers this approach to be a reasonable one to prioritise the inclusion of evidence that is likely to be most useful for decision-making.

It is possible that the skill and experience of the endoscopist may impact the potential benefits associated with AI-supported colonoscopy technologies. Where data allows, differences in outcomes based on endoscopist skill and experience will be explored using subgroup and sensitivity analyses. Subgroup analyses will be constructed based on input from the EAG's clinical experts based on how skill and experience is reported in the trials, and criteria outlined by the Joint Advisory Group in Gastrointestinal Endoscopy (JAG) will be considered if studies report these details.

The EAG anticipates there may be issues surrounding the versions of the technologies used in the clinical trials and versions currently in use, for example due to software updates. The EAG will highlight any concerns in its report based on information available from the evidence identified or additional information provided by the companies. Information on whether the same software version was used in all patients within a trial, or whether updates to software occurred during trials, will be recorded if this is available. Trials will not be excluded based on the version of the technology used but the EAG may explore this as a potential source of heterogeneity within analyses (for example, by performing sensitivity analyses) if this information is consistently reported within the trials.

There may be concerns about the applicability of technologies to UK populations if data used to train the AI technologies deviate substantially from the UK population. The EAG will extract this

information where available from the evidence identified, as well as any additional information provided by the companies, and highlight any concerns in its report. Furthermore, some technologies may be excluded from use in certain populations; the EAG will extract details of this where reported as part of the summary for each technology.

3.3 Comparators

The relevant comparator for this assessment is colonoscopy performed without the use of AI-supported technologies. This may be with or without the use of VCE, dye-based chromoendoscopy or Endocuff Vision™. The EAG will include any study's description of colonoscopy and will consult its clinical experts if there are concerns about the applicability of this comparator in any study, for example if there are particular features (such as additional devices used with the colonoscope not outlined in the NICE scope) of the colonoscopy procedure used that may not be considered "standard" in the UK. Based on this feedback the EAG will decide whether or not the study should be included. If there are sufficient data to do so, the EAG will explore the impact of using VCE, dye-based chromoendoscopy or Endocuff Vision™ on the outcomes of AI-supported colonoscopy technologies using subgroup or sensitivity analyses.

3.4 Outcomes

Outcomes outlined in the NICE final scope (including cost outcomes) are summarised in Section 2.6. Evidence for some of these outcomes will be identified from the clinical evidence (as summarised in Section 3.6 below), while others may be obtained from the economic review or economic model, which will link different types of data. More details on the types of evidence that will be sought as part of the clinical evidence review, and key study designs, are provided in the subsequent section.

The EAG is aware that information on outcomes related to the number of polyps detected or detection rates varies widely across studies in terms of the specific types of polyps that details are provided for. For example, ADRs may be reported but rates specifically for advanced adenomas and for other types of polyp (such as sessile serrated lesions) and location of polyps (such as right-sided polyps) may also be provided. Based on feedback from its clinical experts that these are important factors to consider, the EAG will extract all of this information where reported. However, if time is limited, priorities for analyses will be based on those that are most commonly reported across studies or which are expected to be a key component in the EAG's economic model.

3.5 Types of clinical evidence and study design

The following provides an outline of the types of clinical evidence that will be sought and the key study designs for each. This will either come from the main SLR for evidence on AI-supported colonoscopy technologies or from further targeted searches based on data gaps identified throughout the project.

The EAG will prioritise data for AI with endoscopist input combined, with data based on AI interpretation alone only included if its inclusion would provide data for interventions or outcomes for which there is no evidence from studies meeting the preferred criteria. Where the assessments have been performed in real-time during a colonoscopy but the use of AI as an adjunct to endoscopist judgement is unclear, studies will be included with additional uncertainties of this evidence highlighted and sensitivity analyses performed.

End-to-end studies comparing the use of AI-supported colonoscopy to colonoscopy without AI-supported technologies

These types of study involve the assignment of patients to AI-supported colonoscopy or colonoscopy procedures without the use of AI, with treatment/follow-up decisions made based on the results of the respective method and downstream outcomes associated with each method (such as impact on cancer detection or mortality) captured within the study. The EAG considers it unlikely that long-term studies reporting the impact on outcomes such as cancer development and mortality will be available but will consider them for inclusion if any are identified. However, based on scoping searches performed by the EAG (including a review of SLRs in the area),[10, 11] there are RCTs that assign patients to one of these two colonoscopy methods reporting shorter term, procedural-related outcomes such as ADR and procedural time, which will be included in this review where all other inclusion criteria are met. If data for outcomes are available from RCTs, the EAG may decide not to extract data from other study types for that particular outcome for each intervention.

Diagnostic accuracy of AI-supported colonoscopy

Feedback from the EAG's clinical experts was that colonoscopy is the gold standard for detection of polyps currently. Based on its scoping searches, the EAG is aware that some tandem studies where both colonoscopy without AI and AI-supported colonoscopy are performed in the same patients for polyp detection; however, these studies generally involve the removal of polyps during whichever procedure is performed first, with histological testing performed on excised polyps to determine

Downes N, *et al. BMJ Digit Health* 2026; 2:e000046. doi: 10.1136/bmjdh-2026-000046

whether they are adenomas.[10] These studies are likely to provide some useful diagnostic information, such as adenoma miss rates for each type of colonoscopy when performed first. If data for outcomes are available from RCTs, the EAG may decide not to extract data from other study types for that particular outcome for each intervention.

For polyp detection, based on its scoping searches, the EAG is aware of studies that report typical diagnostic accuracy measures such as sensitivity, specificity and area under the curve (AUC) but notes that these are mostly based on *ex vivo* application of the AI technologies to images or videos from colonoscopies rather than performed in real-time during a colonoscopy.[11] As described in Section 3.2, the EAG does not consider these data to be useful and will exclude them given they will not capture use of the technology during a real-time colonoscopy with associated procedural factors and interaction between the AI technology and endoscopist, which may limit conclusions that can be drawn from these studies, and there is ESGE guidance that *in vivo* data for technologies should be available for them to be considered for use. It will, however, report the sensitivity/specificity of each technology based on training/validation sets where reported in the studies included in the review as part of the general information about each AI technology.

The EAG will consider the inclusion of diagnostic accuracy data for AI-supported polyp detection (where it is used during a real-time colonoscopy) from studies using colonoscopy as the reference standard but considers these data may be scarce. Other reference standards may be considered in discussion with the EAG's clinical experts if available and if data using the preferred reference standard are not available, as long as the accuracy of the AI-supported technology has been assessed in real-time (i.e. accuracy measures are based on *in vivo* rather than *ex vivo* application of the technology).

For AI-supported diagnosis of polyps, diagnostic accuracy data is expected to be available with results on histology used as the reference standard, which is considered to be the reference standard for diagnosis by the EAG's clinical experts and will be included in this assessment. Other reference standards may be considered in discussion with the EAG's clinical experts if available and if data using the preferred reference standard are not available. As for polyp detection, these data will only be included if the AI technology was applied in real-time rather than retrospectively to videos or images (i.e. accuracy measures based on *in vivo* rather than *ex vivo* application of the technology).

Additional clinical inputs for the economic model

Depending on the evidence identified in the initial clinical searches and the design of the economic model, it may be necessary to search for and identify additional types of clinical evidence to link outcomes from the studies available to longer term outcomes for use in the economic model. Pragmatic decisions about the inclusion of these in terms of study design and setting will be made depending on the time available and requirements of the economic model, and the EAG may seek advice from its clinical experts in terms of the most suitable sources to inform the modelling.

3.6 Overview of systematic literature review methodology

An SLR of the clinical evidence for AI-supported polyp detection during colonoscopy will be performed. Methods used in the review will follow principles outlined in guidance from the Centre for Reviews & Dissemination (CRD),[12] the PRISMA statement,[13] the NICE health technology evaluations manual and guidance from Cochrane,[14-16] including the Cochrane Handbooks for Systematic Reviews of Diagnostic Test Accuracy and Systematic Reviews of Interventions as applicable for each study. Further details on the methods used for searching, data extraction, quality assessment and evidence synthesis are provided in the sections that follow.

As noted in Section 3.5, additional searches may be required if there are data gaps, such as targeted searches to inform parameters used in the economic model; these will be performed as required throughout the project based on discussions within the EAG and with NICE.

3.7 Search strategy

The EAG's strategy for identifying evidence on AI-supported colonoscopy technologies for polyp detection/diagnosis will comprise the following key elements:

- Searching of electronic databases, trial registers and websites (see breakdown below);
- Contact with experts in the field;
- Review of bibliographies of retrieved papers and existing SLRs.

Electronic databases:

- MEDLINE (R) ALL (via Ovid);
- Embase (via Ovid);
- Cochrane Database of Systematic Reviews (CDSR; via Cochrane Library);
- Cochrane Central Register of Controlled Trials (CENTRAL; via Cochrane Library).

Clinical trial/systematic review registers:

- World Health Organization (WHO) International Clinical Trials Registry Platform (ICTRP);
- Clinicaltrials.gov;
- PROSPERO.

Conferences

- American Society of Clinical Oncology (ASCO);
- American Association for Cancer Research (AACR);
- European Society for Medical Oncology (ESMO);
- European Association for Cancer Research (EACR) Congress;
- British Society of Gastroenterology (BSG) Annual Meeting/BSG Live;
- World Congress of Gastrointestinal (GI) Endoscopy (ENDO);
- Digestive Disease Week (DDW);
- European Society of Gastrointestinal Endoscopy (ESGE) Days;
- The European Society of Coloproctology Annual Conference;
- Asian Pacific Digestive Week (APDW).

Health technology assessments

- International Network of Agencies for Health Technology Assessment (INAHTA) Database;
- NICE;
- Scottish Intercollegiate Guidelines Network (SIGN);
- HTA Wales;
- Canada's Drug Agency (formerly Canadian Agency for Drugs and Technologies in Health [CADTH]).

Other sources

- Websites of manufacturers of the relevant AI technologies;
- Company submissions provided as part of this appraisal;
- Food and Drug Administration (FDA) website.

For searches of electronic databases, search terms will include terms for colonoscopy combined with terms for AI, using a combination of free-text searches and subject headings. Free-text searches for individual product names will also be included, without the need to be combined with other terms

for colonoscopy or AI. Draft MEDLINE and Embase search strategies are included in Appendix 8.1. Strategies used for other sources will be detailed in the EAG's report.

Based on consultation with its clinical experts and a review of other SLRs in the area, including the report produced by HTW,[10] the EAG will apply the search strategy from 2010 onwards given that data concerning the technologies of interest are unlikely to have been published prior to this. Searches will also be limited to human studies, with no filters for study design applied.

Reference lists of included papers as well as relevant SLRs and existing guidance in the area will be reviewed to identify any additional studies relevant for this review. Where time allows and where deemed important for the review, study authors or companies will be contacted for further information.

3.8 Data extraction strategy

Study selection

Titles and abstracts of each record retrieved from searches of electronic databases will be assessed against inclusion and exclusion criteria for the review by two independent reviewers, with any discrepancies between the two resolved by discussion, with involvement of a third reviewer where necessary. Full texts of those records identified as relevant will be obtained and the same process of double-reviewing will be performed for the full text review.

One reviewer will perform the searches of sources other than electronic databases and identify data relevant for inclusion in the review, including registries, conference proceedings, health technology assessment (HTA) databases and other websites/sources. If there is uncertainty about the inclusion of any data identified solely through these sources, this will be discussed with a second reviewer.

Studies not published in the English language will be included if sufficient information can be extracted from non-English language full-texts or from an abstract published in English, including sufficient information to allow a critical appraisal and sufficient data for results to be included in analyses. If time allows, the EAG may explore the use of freely available online translation systems to aid in extracting sufficient information from non-English languages studies.

Studies that are available solely as abstracts or that are not peer-reviewed will only be included if there is sufficient information to allow a critical appraisal and results are presented in sufficient

detail to be included in analyses, particularly where sufficient data are already available from full-text, peer-reviewed articles.

Data extraction

Data from relevant studies will be extracted into standardised data extraction forms, with different forms likely required for different types of study. Data for each study will be extracted by a single reviewer, with validation performed by a second reviewer. Any conflicts will be resolved through discussion, with involvement of a third reviewer if required. The data extraction process will be piloted at the start of the data extraction stage to improve consistency between reviewers.

Multiple papers for the same study will be linked and extracted as a single study. As a default, data from the latest appropriate publication will be used as the primary source, with additional publications reviewed for any additional data not reported elsewhere. Where information key to the review is missing from studies, if time allows and where deemed important for the review, study authors or companies will be contacted for further information. If no response is received within the timeframe requested by the EAG, it will be assumed that this information is unavailable.

3.9 Quality assessment strategy

Quality assessment tools appropriate for each study design will be used, including version 2 of the Cochrane Risk of Bias tool (ROB2) for RCTs and QUADAS-2 for diagnostic accuracy studies.[17, 18] For studies that do not match either of these designs, alternative quality assessment tools will be identified relevant to the study design. Each study will be quality assessed by one reviewer at the study-level, with scores validated by a second reviewer and any conflicts discussed with involvement of a third reviewer if necessary. For any domains on these tools that require a judgement at an outcome level (for example, missing data or outcome assessment methods), the assessment will be based on the primary outcome for each study due to time constraints. The possible effects of study quality on the review findings will be discussed.

3.10 Methods of analysis/synthesis

Data for each technology will be analysed separately given the inherent differences likely to be associated with different technologies. Where data are limited for particular interventions and/or outcomes, assumptions may need to be made in terms of inclusion in the economic model.

Extracted data and quality assessments will be presented in tables within the EAG's report, with results of any meta-analyses presented in tables and Forest plots.

For analyses of data from RCTs, where meta-analyses are possible (i.e. where there is more than one study reporting the same outcome for a specific technology), fixed or random effects meta-analyses will be performed using Review Manager (RevMan) for comparisons between each AI-supported colonoscopy technology and colonoscopy without the use of AI technologies.[19] Arm-based data (such as adenoma detection rate in each arm) and relative effect measures (such as odds ratios or risk ratios) will be extracted from each study (where available) and the summary statistics used for the EAG's analyses will be based on the availability of data across studies to ensure as much data as possible can be meta-analysed where appropriate. For relative effect measures reported within studies, unadjusted versions as well as adjusted versions will be extracted; where multiple adjusted analyses are presented, the analysis with the most variables adjusted for will be preferentially extracted, with details of any other adjusted analyses reported narratively for information. Where available, the results of intention to treat (ITT) analyses within studies will be preferentially extracted.

Subgroup analyses will be performed where data allows as outlined in Section 3.1 and any concerns about additional heterogeneity between studies and the potential impact on results will be commented on in the EAG's report. Data that cannot be meta-analysed will be summarised narratively.

The EAG considers it unlikely that there will be sufficient diagnostic accuracy data (e.g. sensitivity and specificity) to perform meta-analyses but it will consider the use of meta-analytic options outlined in guidance from Cochrane if sufficient data are identified and if it is decided that these data would be useful for the economic model.[20] Otherwise, the EAG will present the data as reported in each study and include a narrative summary. In addition, data on AEs are likely to be scarce given they are usually few in number and may vary between patients; therefore, it is anticipated that there will be limited data to include in meta-analyses. Raw data on AEs will be extracted and presented for each study, with meta-analyses performed where data for the same AE is reported in at least two studies per technology. Where meta-analyses are not possible, a narrative description of AEs will be included.

3.11 Methods for estimating quality of life

Any quality of life data relating to the use of AI technologies (and colonoscopy without AI technologies where reported) identified as part of the clinical SLR described above will be extracted. Additional quality of life data for use in the economic model will be identified as part of Section 4 or as part of additional searches mentioned in Section 3.6.

4 Methods for synthesising evidence of cost-effectiveness

The economic evaluation will assess the cost-effectiveness of AI software to support in the detection and characterisation of polyps during colonoscopy, compared with colonoscopy without AI, in all populations eligible for colonoscopy (i.e. people undergoing screening or surveillance for CRC, or those with signs or symptoms suggestive of CRC).

An SLR of existing economic evaluations will be undertaken to ascertain the existent of extant cost-effectiveness models that could be used for the current assessment. Materials related to known ongoing relevant diagnostic appraisals (for example, the ongoing appraisal for PillCam COLON 2 [GID-DG10083]),[21] will also be considered. If no extant cost-effectiveness models are identified, a *de novo* economic model will be developed, incorporating elements of existing models where possible.

4.1 Identifying and systematically reviewing published cost-effectiveness studies

An SLR will be undertaken to identify published full economic evaluations of AI-supported colonoscopy for detection and characterisation of polyps during colonoscopy, in all populations eligible for colonoscopy. A search filter to identify economic evaluations will be applied to the search strategies and the electronic databases will be searched from inception until the latest available version.

The following databases will be searched for relevant studies:

- MEDLINE (R) ALL (via Ovid);
- Embase (via Ovid);
- EconLit (via Ovid);
- Cochrane Database of Systematic Reviews (CDSR; via Cochrane Library);
- Cochrane Central Register of Controlled Trials (CENTRAL; via Cochrane Library);
- International Network of Agencies for Health Technology Assessment (INAHTA) Database;
- NHS Economic Evaluation Database (NHS EED).

Separate searches will be carried out for supporting information on costs and resource use, as well as on utility data. Study selection and data extraction will be carried out as described in Section 3.8.

The search strategy will combine terms capturing the interventions or comparators (Section 3.2 and 3.3) of interest and the target population (Section 3.1). Health economic and quality of life search

terms will be applied to capture the study designs of interest (cost-effectiveness, cost and quality of life, health state utility values [HSUVs]).

In addition, clinical experts in the field will be contacted with a request for details of published and unpublished studies of which they may have knowledge. Furthermore, identified SLRs and companies' submissions will be searched for additional references.

Main findings from the studies identified from the SLR will be presented with a narrative synthesis and structured tables.

4.2 Evaluation of costs and cost effectiveness

Full economic evaluations addressing the cost-effectiveness of any AI-supported colonoscopy system published in English will be critically appraised, and may be used to identify evidence sources to inform structural assumptions and input parameter values to be used in the EAG's model.

No restriction by setting or geographical location will be applied to the search strategy. If sufficient data is available from a UK setting, data from non-UK based studies will not be extracted.

The methodological quality of the full economic evaluations identified in the review will also be assessed using the Drummond checklist.[22]

4.3 Development of a health economic model

If no suitable extant cost-effectiveness model is identified from the SLR, a *de novo* model will be developed following the completion of the SLR and discussion with clinical experts. The model will assess the cost effectiveness of AI-supported colonoscopy for detection and characterisation of polyps during colonoscopy, compared with colonoscopy without AI. The interventions included in the model will be any of the technologies included in the scope (detailed in Section 3.2) for which sufficient efficacy data are available to accurately parametrise the model. The model will consider the CADe functionality of the individual technologies; CADx functionalities will also be considered for technologies incorporating this functionality, for which appropriate efficacy data are available to parametrise the model. The model will assume the current UK clinical practice for polyp removal (i.e. resection of all polyps, with the exception of rectal hyperplastic polyps, which are left *in situ*, followed by histopathological examination of all removed polyps). If sufficient relevant efficacy data are available, alternative strategies including 'resect and discard' (i.e., removal of some polyps

without sending for histopathological testing) and 'diagnose and leave in place' (i.e. some polyps considered low risk are not removed) will also be considered for diminutive polyps.

While these strategies are not currently widely used in UK clinical practice, it is the approach recommended for experts by the ESGE 2024 guideline on endoscopic mucosal resection (EMR),[23] and may ultimately be adopted more widely in the UK; in particular, the use of this strategy is being started within the NHS BCSP. A key benefit of CADx technology would be in supporting optical diagnosis to facilitate adoption of these strategies, reducing the requirement for polypectomies and histopathological examination of benign polyps.

The population considered in the model will be all patients eligible for colonoscopy; the following subpopulations may be considered separately:

- People undergoing routine screening for CRC;
- People undergoing surveillance for CRC, due to a personal history of CRC;
- People undergoing surveillance for CRC, due to a prior polypectomy;
- People undergoing surveillance for CRC, due to increased risk resulting from hereditary CRC syndromes;
- People undergoing surveillance for CRC, due to IBD;
- People who have presented with signs or symptoms indicative of CRC.

The inclusion of the populations described above in the model will be conditional on the availability of appropriate relevant data to parametrise effectiveness; this may differ between technologies (i.e., the model may include analyses for a given population for some technologies but not others).

Since it is likely that AI technologies will provide the greatest benefit for endoscopists with a reduced level of skill or experience, subgroups based on endoscopist skill or experience may be incorporated into the model, if sufficient data are available.

The economic assessment will be undertaken from the perspective of the NHS and Personal Social Services. A lifetime horizon will be used, and both costs and benefits will be discounted at 3.5% per annum.

Model parameters (e.g. utilities, cost data) will be populated from the results of the economic and outcome searches and combined with unit costs from NHS reference costs and other relevant publications of UK health care costs as appropriate. The EAG will seek expert opinion if published

data are not available to inform all model parameters. All evidence will be evaluated according to the recommendations of the NICE health technology evaluations manual.[14]

4.3.1 Model structure

The structure of the model will take into consideration previous economic models related to colonoscopy for polyp detection or diagnosis, with a particular focus on models related to AI-supported technologies, and clinical evidence identified from the SLR. While the exact model structure is subject to change, it is anticipated that event pathways may be modelled by using a hybrid model; a decision tree will be used to determine outcomes for the initial colonoscopy, followed by a natural history submodule with a state transition structure to estimate long-term costs and benefits. The decision tree submodule will include branches based on patients' true underlying condition, colonoscopy results, and point of entry into the natural history submodule. Patients' underlying condition will reflect whether the patient has a healthy epithelium (which may include the presence of benign polyps), adenoma (i.e., pre-cancerous polyps) or CRC (i.e., malignant lesions which may also be detected during colonoscopy). Possible colonoscopy outcomes represented in the decision tree will include missed low-risk adenomas (LRA), high-risk adenomas (HRA) or CRC, either due to detection failure or misdiagnosis, and unnecessary benign polyp removal due to misdiagnosis. The natural history submodule will reflect disease progression and care pathways for adenomas and CRC; the proposed structure will be aligned with current UK NHS clinical practice, and validated by clinical experts. Similar model structures have been used in previous related NICE diagnostic assessments, for example, the appraisals for virtual chromoendoscopy technologies to detect colorectal polyps (DG28) and quantitative faecal immunochemical testing (DG56).[24, 25]

Potential structures for each submodule are shown in Supplementary Figures 1 and 2 below, although these are subject to change based on evidence identified in the SLRs described above.

Supplementary Figure 1. Potential decision tree submodule structure

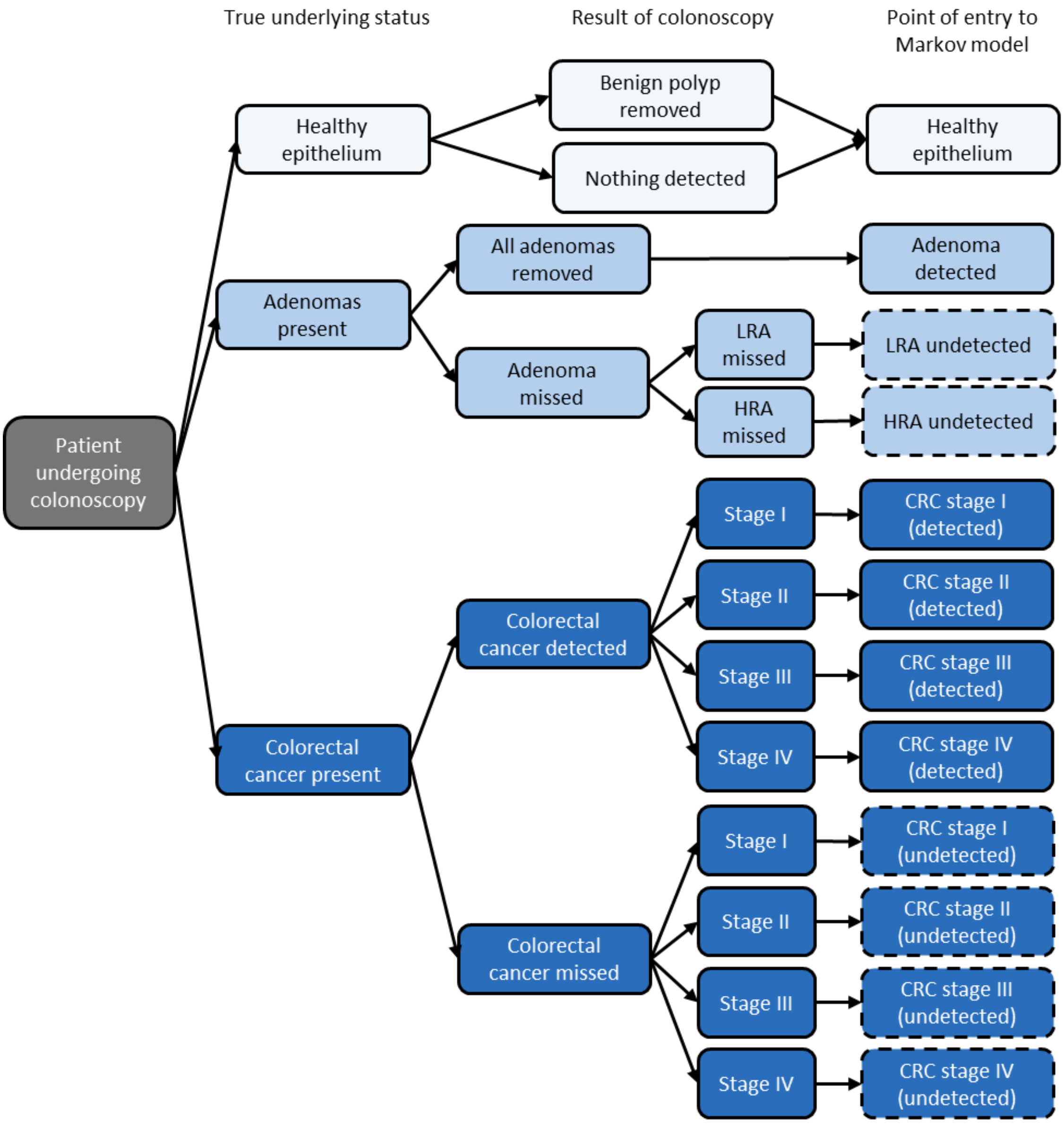

Abbreviations: CRC, colorectal cancer; HRA, high-risk adenoma; LRA, low-risk adenoma.

Supplementary Figure 2. Potential natural history submodule structure

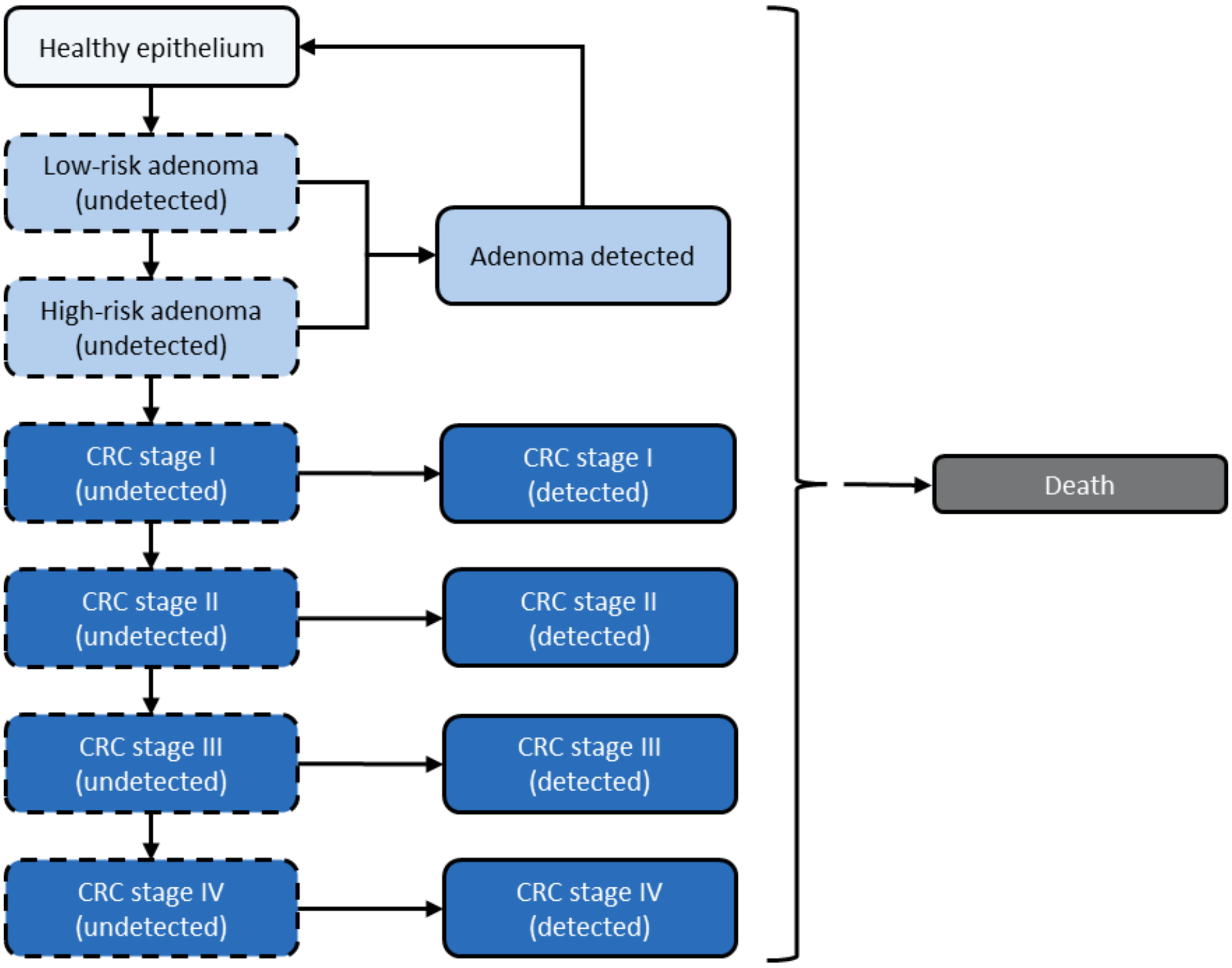

Abbreviations: CRC, colorectal cancer; HRA, high-risk adenoma; LRA, low-risk adenoma.

It is anticipated that surrogate outcomes will be used to determine the effectiveness of the intervention and comparator technologies during the initial phase (i.e., the probability of failing to identify adenomas). In particular, the results of initial scoping searches indicate that studies assessing CADe colonoscopy often focus on the ADR, the proportion of patients undergoing colonoscopy with at least one adenoma detected, or the adenoma miss rate (AMR), the proportion of total detected adenomas which are identified by the technology of interest. Long-term outcomes (for example, the rates of CRC diagnosis or mortality) are rarely reported. Mortality will be estimated using survival data for relevant CRC stages, or aligned with age- and sex-adjusted general population rates, if no cancer is present.

The economic analysis will consider the following cost categories:

- Cost of colonoscopy with/without polypectomy, as appropriate;
- Additional cost per patient of AI system;

- Cost of histological examination of removed polyps;
- Cost associated with AEs associated with colonoscopy/polypectomy;
- Costs for treatment and follow-up care for CRC.

Utility values will be assigned to each health state in the natural history submodule; these will either be utility values for the relevant CRC stage sourced from the literature with age-adjustment applied, or aligned with age- and sex-adjusted general population values if no CRC is present. Disutilities corresponding to AEs resulting from colonoscopies and polypectomies will also be applied.

The output of the economic model will be incremental cost effectiveness ratios (ICERs), using quality-adjusted life-years (QALYs) as the measure of effectiveness. The estimated change in number of colonoscopies, polypectomies and histopathological tests will also be estimated. Various scenario analyses will be conducted to test the robustness of the model to changes in parameter assumptions and potentially also to alternative data sources. A one-way deterministic scenario analysis (DSA) will be conducted to explore individual parameter uncertainty. To assess the overall uncertainty in the model estimates, a probabilistic sensitivity analysis (PSA) will be conducted using appropriately sampled values for all relevant parameters in the model. If the model is non-linear, the central estimate produced by the PSA is likely to be a more accurate reflection of the results compared to the deterministic results.

Downes N, *et al. BMJ Digit Health* 2026; 2:e000046. doi: 10.1136/bmjdh-2026-000046

5 Handling information from the companies

All data submitted by the company(s) will be considered if received by the EAG no later than 26 June 2025. Data arriving after this date may not be considered. If the data meet the inclusion criteria for the review they will be extracted and quality assessed in accordance with the procedures outlined in this protocol.

Any 'academic in confidence' (AIC) or 'commercial in confidence' (CIC) data provided by a company and specified as such will be highlighted in yellow or blue, respectively, and underlined in the assessment report (followed by an indication of the relevant company name e.g. in brackets).

6 Competing interests of authors

None.

7 Timetable/milestones

Milestones for this project are detailed in Supplementary Table 2 below.

Supplementary Table 2. Future milestones for this project

Milestone	Date to be completed
Progress report	26/11/24
Draft report to NICE	04/03/25
Final report and economic model to NICE	12/08/25

Abbreviations: NICE, National Institute for Health and Care Excellence.

8 Appendices

8.1 Draft MEDLINE and Embase search strategies

Supplementary Table 3. EAG search strategy for MEDLINE via Ovid – 28th August 2024

#	Searches	Results (28/08/2024)
1	Colonoscopy/	32,630
2	Sigmoidoscopy/	4,919
3	Proctoscopy/	2,134
4	(colonoscop* or polypect* or sigmoidoscop* or proctoscop* or coloscop* or ileocolonoscop* or anoscop* or rectoscop* or proctosigmoidoscop*).tw,kf.	49,476
5	1 or 2 or 3 or 4	63,970
6	Endoscopy, Gastrointestinal/	21,811
7	endoscop*.tw,kf.	267,703
8	6 or 7	273,098
9	exp intestine, large/	151,934
10	lower gastrointestinal tract/	205
11	(colon or colons or colonic or sigmoid or sigmoids or rectum* or rectal or colorect* or anus or anal or cecum or caecum or cecal or caecal).tw,kf.	586,452
12	(lower bowel* or lower intestin* or lower gastrointestin* or lower gastro-intestin* or lower GI or large bowel* or large intestin*).tw,kf.	32,069
13	9 or 10 or 11 or 12	641,147
14	8 and 13	33,898
15	5 or 14	82,317
16	exp Artificial Intelligence/	206,347
17	exp Machine Learning/	74,342
18	Deep Learning/	22,051
19	((artificial or machine* or comput* or augment* or amplif*) adj2 intelligen*).tw,kf.	59,117
20	AI.tw,kf.	60,501
21	((machine or deep or transfer* or hierarch* or computer) adj2 (learn* or reasoning)).tw,kf.	185,827
22	Sentiment Analysis/	213
23	("sentiment analysis" or "opinion mining").tw,kf.	2,062

24	Support Vector Machine/	10,791
25	(vector adj2 machine).tw,kf.	24,448
26	neural networks, computer/	54,616
27	((neural or convolut* or artificial) adj2 network).tw,kf.	84,905
28	(CNN or CNNs or ANN or ANNs).tw,kf.	40,958
29	"neural net".tw,kf.	628
30	Natural Language Processing/	7,033
31	(natural adj2 language adj2 process*).tw,kf.	10,397
32	"large language model".tw,kf.	1,003
33	("cognitive computing" or "computer vision").tw,kf.	10,094
34	Image Processing, Computer-Assisted/	144,731
35	Pattern Recognition, Automated/	26,686
36	Image Interpretation, Computer-Assisted/	48,597
37	Diagnosis, Computer-Assisted/	24,494
38	((computer or machine) adj1 (aid* or base* or assist* or support*)).tw,kf.	80,359
39	"CADe".tw,kf.	448
40	"CADx".tw,kf.	304
41	16 or 17 or 18 or 19 or 20 or 21 or 22 or 23 or 24 or 25 or 26 or 27 or 28 or 29 or 30 or 31 or 32 or 33 or 34 or 35 or 36 or 37 or 38 or 39 or 40	666,037
42	15 and 41	2,440
43	(GI Genius or GIGenius or ENDO-AID or ENDOAID or WISE VISION or WISEVISION or CAD-EYE or CADEYE or MAGENTIQ or EndoAngel or Endo-Angel or CADDIE or Endoscreener or Endo-screener).tw,kf.	91
44	(Discovery and Pentax).tw,kf.	2
45	(Argus or EMIS or Endoscopic Multimedia Information System).tw,kf.	1,619
46	45 and 22	0
47	43 or 44 or 46	93
48	42 or 47	2,481
49	exp animals/ not humans/	5,252,742
50	48 not 49	2,431
51	50	2,431
52	limit 51 to yr="2010 -Current"	1,897

Database: Ovid MEDLINE(R) ALL 1946 to August 27, 2024. Search run on 28th August 2024. The number of hits are reported from a search during the protocol development stage for illustration only. The search will be repeated at the start of the systematic literature review stage of the project.

Supplementary Table 4. EAG search strategy for Embase via Ovid – 28th August 2024

#	Searches	Results (28/08/2024)
1	colonoscopy/	110,660
2	exp polypectomy/	12,424
3	exp endoscopic polypectomy/	3,185
4	sigmoidoscopy/	14,756
5	rectoscopy/	2,949
6	ileocolonoscopy/	1,628
7	(colonoscop* or polypect* or sigmoidoscop* proctoscop* or coloscop* or ileocolonoscop* or anoscop* or rectoscop* or proctosigmoidoscop*).tw,kf.	92,367
8	1 or 2 or 3 or 4 or 5 or 6 or 7	143,991
9	gastrointestinal endoscopy/	42,777
10	endoscop*.tw,kf.	435,498
11	9 or 10	450,366
12	exp large intestine/	213,223
13	sigmoid/	20,888
14	lower gastrointestinal tract/	909
15	exp rectum/	43,445
16	exp anus/	22,959
17	cecum/	22,296
18	(colon or colons or colonic or sigmoid or sigmoids or rectum* or rectal or colorect* or anus or anal or cecum or caecum or cecal or caecal).tw,kf.	835,126
19	(lower bowel* or lower intestin* or lower gastrointestin* or lower gastro-intestin* or lower GI or large bowel* or large intestin*).tw,kf.	42,760
20	12 or 13 or 14 or 15 or 16 or 17 or 18 or 19	912,043
21	11 and 20	71,426
22	8 or 21	180,358
23	artificial intelligence/	85,289

24	cognitive technology/	7
25	exp machine learning/	506,582
26	deep learning/	63,048
27	((artificial or machine* or comput* or augment* or amplif*) adj2 intelligen*).tw,kf.	68,471
28	AI.tw,kf.	80,002
29	((machine or deep or transfer* or hierarch* or computer) adj2 (learn* or reasoning)).tw,kf.	215,096
30	sentiment analysis/	883
31	("Sentiment analysis" or "opinion mining").tw,kf.	1,810
32	exp support vector machine/	47,569
33	(vector adj2 machine).tw,kf.	29,352
34	cognitive computing/	41
35	computer vision/	4,551
36	("cognitive computing" or "computer vision").tw,kf.	10,779
37	natural language processing/	13,708
38	(natural adj2 language* adj2 process*).tw,kf.	12,095
39	large language model/	2,065
40	"large language model".tw,kf.	1,028
41	artificial neural network/	59,989
42	convolutional neural network/	34,166
43	((neural or convolut* or artificial) adj2 network*).tw,kf.	142,786
44	(CNN or CNNs or ANN or ANNs).tw,kf.	99,324
45	"neural net".tw,kf.	793
46	computer analysis/	124,813
47	computer assisted diagnosis/	43,320
48	pattern recognition/	37,600
49	((computer or machine) adj1 (aid* or base* or assist* or support*)).tw,kf.	101,321
50	"CADe".tw,kf.	791
51	"CADx".tw,kf.	445
52	23 or 24 or 25 or 26 or 27 or 28 or 29 or 30 or 31 or 32 or 33 or 34 or 35 or 36 or 37 or 38 or 39 or 40 or 41 or 42 or 43 or 44 or 45 or 46 or 47 or 48 or 49 or 50 or 51	996,005

53	22 and 52	4,637
54	(GI Genius or GIGenius or ENDO-AID or ENDOAID or WISE VISION or WISEVISION or CAD-EYE or CADEYE or MAGENTIQ or EndoAngel or Endo-Angel or CADDIE or Endoscreener or Endo-screener).tw,kf.	304
55	(Discovery and Pentax).tw,kf.	7
56	(Argus or EMIS or Endoscopic Multimedia Information System).tw,kf.	2,239
57	56 and 22	14
58	54 or 55 or 57	325
59	53 or 58	4,760
60	exp animals/ not humans/	11,818,451
61	59 not 60	4,106
62	limit 61 to yr="2010 -Current"	3,653
Database: Ovid Embase 1946 to August 27, 2024. Search run on 28th August 2024. The number of hits are reported from a search during the protocol development stage for illustration only. The search will be repeated at the start of the systematic literature review stage of the project.		

9 References

1. Cancer Research UK. About cancer - bowel cancer. Available from: https://www.cancerresearchuk.org/about-cancer/bowel-cancer. Date accessed: Aug 24.
2. Bowel Cancer UK. About bowel cancer - bowel cancer. Available from: https://www.bowelcanceruk.org.uk/about-bowel-cancer/bowel-cancer/. Date accessed: Aug 24.
3. Cancer Research UK. Bowel cancer statistics. Available from: https://www.cancerresearchuk.org/health-professional/cancer-statistics/statistics-by-cancer-type/bowel-cancer#heading-One. Date accessed: Dec 2024.
4. NHS England. Screening and earlier diagnosis. Available from: https://www.england.nhs.uk/cancer/early-diagnosis/screening-and-earlier-diagnosis/. Date accessed: Dec 2024.
5. National Health Service (NHS). Bowel polyps, 2023. Available from: https://www.nhs.uk/conditions/bowel-polyps/. Date accessed: Dec 2024.
6. National Institute for Health and Care Excellence (NICE). Artificial intelligence (AI) technologies to help detect or characterise colorectal polyps. HealthTech Guidance [HTG773]: Final Scope, 2024. Available from: https://www.nice.org.uk/guidance/htg773/history. Date accessed: Mar 26.
7. Ahmad A, Wilson A, Haycock A, Humphries A, Monahan K, Suzuki N, et al. Evaluation of a real-time computer-aided polyp detection system during screening colonoscopy: AI-DETECT study. *Endoscopy* 2022; **55**: 313-9.
8. Bisschops R, East JE, Hassan C, Hazewinkel Y, Kamiński MF, Neumann H, et al. Advanced imaging for detection and differentiation of colorectal neoplasia: European Society of Gastrointestinal Endoscopy (ESGE) Guideline - Update 2019. *Endoscopy* 2019; **51**: 1155-79.
9. Messmann H, Bisschops R, Antonelli G, Libânio D, Sinonquel P, Abdelrahim M, et al. Expected value of artificial intelligence in gastrointestinal endoscopy: European Society of Gastrointestinal Endoscopy (ESGE) Position Statement. *Endoscopy* 2022; **54**: 1211-31.
10. Health Technology Wales (Technoleg Iechyd Cymru). Artificial Intelligence (AI)-assisted endoscopy in the detection of gastrointestinal cancer and pre-cancerous lesions, 2024. Available from: https://healthtechnology.wales/reports-guidance/ai-assisted-endoscopy-for-gastrointestinal-cancer/. Date accessed: Jul 24.
11. Nazarian S, Glover B, Ashrafian H, Darzi A, Teare J. Diagnostic Accuracy of Artificial Intelligence and Computer-Aided Diagnosis for the Detection and Characterization of Colorectal Polyps: Systematic Review and Meta-analysis. *J Med Internet Res* 2021; **23**: e27370.
12. Centre for Reviews and Dissemination (CRD). University of York. Systematic reviews: CRD's guidance for undertaking reviews in health care, 2009. Available from: https://www.york.ac.uk/media/crd/Systematic_Reviews.pdf. Date accessed: July 24.
13. Page MJ, McKenzie JE, Bossuyt PM, Boutron I, Hoffmann TC, Mulrow CD, et al. The PRISMA 2020 statement: an updated guideline for reporting systematic reviews. *BMJ* 2021; **372**: n71.
14. National Institute for Health and Care Excellence (NICE). NICE health technology evaluations: the manual, 2022. Available from: https://www.nice.org.uk/process/pmg36. Date accessed: July 24.
15. Deeks JJ BP, Leeflang MM, Takwoingi Y (editors),. Cochrane Handbook for Systematic Reviews of Diagnostic Test Accuracy. Version 2.0 (updated July 2023). Cochrane, 2023. Available from: https://training.cochrane.org/handbook-diagnostic-test-accuracy/current. Date accessed: July 24.
16. Higgins JPT TJ, Chandler J, Cumpston M, Li T, Page MJ, Welch VA (editors),. Cochrane Handbook for Systematic Reviews of Interventions version 6.4 (updated August 2023). Cochrane, 2023. Available from: www.training.cochrane.org/handbook. Date accessed: July 24.
17. Higgins JPT SJ, Page MJ, Elbers RG, Sterne JAC,. Chapter 8: Assessing risk of bias in a randomized trial. In: Higgins JPT, Thomas J, Chandler J, Cumpston M, Li T, Page MJ, Welch VA (editors).

Cochrane Handbook for Systematic Reviews of Interventions version 6.4 (updated August 2023). Cochrane, 2023. Available from: www.training.cochrane.org/handbook. Date accessed: Aug 24.
18. Reitsma JB RA, Whiting P, Yang B, Leeflang MM, Bossuyt PM, Deeks JJ,. Chapter 8: Assessing risk of bias and applicability. In: Deeks JJ, Bossuyt PM, Leeflang MM, Takwoingi Y (editors). Cochrane Handbook for Systematic Reviews of Diagnostic Test Accuracy. Version 2.0 (updated July 2023). Cochrane, 2023. Available from: https://training.cochrane.org/handbook-diagnostic-test-accuracy/current. Date accessed: Aug 24.
19. The Cochrane Collaboration. Review Manager (RevMan). 2024.
20. Macaskill P TY, Deeks JJ, Gatsonis C,. Chapter 9: Understanding meta-analysis. In: Deeks JJ, Bossuyt PM, Leeflang MM, Takwoingi Y (editors). Cochrane Handbook for Systematic Reviews of Diagnostic Test Accuracy. Version 2.0 (updated July 2023). Cochrane., 2023. Available from: https://training.cochrane.org/handbook-diagnostic-test-accuracy/current Date accessed: Aug 24.
21. National Institute for Health and Care Excellence (NICE). PillCam COLON 2 for investigation of the colon through direct visualisation. In development [GID-DG10083]. Available from: https://www.nice.org.uk/guidance/indevelopment/gid-dg10083/documents [Last accessed: 13 August 2024]. 2024.
22. Drummond MF, Sculpher MJ, Claxton K, Stoddart GL, Torrance GW. *Methods for the economic evaluation of health care programmes*: Oxford university press; 2015.
23. Ferlitsch M, Hassan C, Bisschops R, Bhandari P, Dinis-Ribeiro M, Risio M, et al. Colorectal polypectomy and endoscopic mucosal resection: European Society of Gastrointestinal Endoscopy (ESGE) Guideline - Update 2024. *Endoscopy* 2024; **56**: 516-45.
24. National Institue for Health and Care Excellence. Virtual chromoendoscopy to assess colorectal polyps during colonoscopy [DG28]. Available from: https://www.nice.org.uk/guidance/dg28 [Last accessed: 06 August 2024]. 2017.
25. National Institue for Health and Care Excellence. Quantitative faecal immunochemical testing to guide colorectal cancer pathway referral in primary care [DG56]. Available from: https://www.nice.org.uk/guidance/dg56 [Last accessed: 06 August 2024]. 2023.
