# Supplementary material for: Computer-aided polyp detection and characterisation systems to support colonoscopy: a systematic review with results stratified by each individual artificial intelligence system
Source: BMJ Digit Health Ai. 2026 Jun 22;2(1):e000046. doi: 10.1136/bmjdh-2026-000046 (PMC13528337; doi:10.1136/bmjdh-2026-000046)
Supplement: Supplementary file 2 [file bmjdh-2-1-bmjdh-2026-000046-s002.pdf]

Online Supplementary File 2

1 Systematic literature review (SLR) methods

1.1 Search methods

1.1.1 Further details of methods

When designing search strategies, previously published SLRs in this area were reviewed to support identification of relevant terminology. In this regard, the Health Technology Wales (HTW) Evidence Appraisal Report on artificial intelligence (AI)-assisted endoscopy for gastrointestinal cancer was a particularly useful resource.[1] Based on discussions with clinical experts and a review of other SLRs in the area, including the aforementioned HTW report,[1] searches in MEDLINE, Embase, Cochrane Central Register of Controlled Trials (CENTRAL) and Cochrane Database of Systematic Reviews (CDSR) were limited to 2010 onwards given these AI systems have emerged after this date. Search strategies were designed and produced by one reviewer, with draft strategies for MEDLINE and Embase validated by a second reviewer.

De novo MEDLINE, Embase, CENTRAL and CDSR searches were performed, with search terms including terms for colonoscopy combined with terms for AI, using a combination of free-text searches and subject headings. Free-text searches for individual product names were also included in these searches, without the need to be combined with other terms for colonoscopy or AI. MEDLINE and Embase searches included lines to exclude animal studies from the search results. For the update in June 2025, date limits were added with the aim of capturing only records added to the databases since the last searches were performed. Furthermore, a correction of an error identified in the MEDLINE search was made in the update search in June 2025 (see footnote of Supplementary Table 5).

Searches for MEDLINE and Embase were performed separately via Ovid, and searches of CENTRAL and CDSR were performed separately via the Cochrane Library. Records from each of these four searches were imported into the freely available version of Rayyan software in September 2024 where they were deduplicated against one another by one reviewer.[2] This process was repeated in June 2025 during the update, with new records also deduplicated against existing records in Rayyan.

Searches of grey literature sources were also performed to identify relevant studies not indexed in the databases searched and to identify ongoing studies. The sources described in Section 1.1.2 below were searched by a single reviewer. Reference lists of included papers and of relevant SLRs were reviewed to identify any additional studies that may have been relevant for inclusion in the review.

1.1.2 Grey literature sources

1.1.2.1 Clinica trial/systematic review registries

The following clinical trial and systematic review registries were searched:

- World Health Organization (WHO) International Clinical Trials Registry Platform (ICTRP);
- Clinicaltrials.gov;
- PROSPERO.

The search strategy for these resources are presented in Section 1.1.2 below, including for the original and update searches. Searches during the update in June 2025 included date limits with the aim of focusing on new records since the original searches.

1.1.2.2 Conference proceedings

The following conference proceedings were searched as part of the original review in September 2024 and/or the update in June 2025:

- American Society of Clinical Oncology (ASCO) conference abstracts 2022, 2023 and 2024;
- American Association for Cancer Research (AACR) conference abstracts 2022, 2023 and 2024;
- European Society for Medical Oncology (ESMO) conference abstracts 2022 and 2023 (2024 had not occurred at the time of searches taking place);
- European Association for Cancer Research (EACR) Congress abstracts 2022, 2023 and 2024;
- British Society of Gastroenterology (BSG) Annual Meeting/BSG Live abstracts 2022, 2023 and 2024;
- World Congress of Gastrointestinal (GI) Endoscopy (ENDO) abstracts 2022 and 2024;

- Digestive Disease Week (DDW) conference abstracts 2022, 2023, 2024 and 2025;
- European Society of Gastrointestinal Endoscopy (ESGE) Days conference abstracts 2022, 2023, 2024 and 2025;
- The European Society of Coloproctology (ESCP) Annual Conference abstracts 2022,2023 and 2024;
- Asian Pacific Digestive Week (APDW) conference abstracts 2022, 2023 and 2024.

During the June 2025 update, a review of any new ASCO, AACR, ESMO and EACR conferences since September 2024 was not prioritised, given the low yield of relevant abstracts from these cancer-specific conferences in the first search.

1.1.2.3 Health technology assessment (HTA) groups

The websites of the following health HTA bodies were searched for relevant appraisals, with any relevant studies within these appraisals crosschecked against studies already identified from searches of databases and other sources:

- International Network of Agencies for Health Technology Assessment (INAHTA) Database;
- National Institute for Health and Care Excellence (NICE);
- Scottish Intercollegiate Guidelines Network (SIGN);
- Health Technology Wales (HTW);
- Canada's Drug Agency (CDA-AMC; formerly Canadian Agency for Drugs and Technologies in Health [CADTH]).

For the update searches in June 2025, the inclusion of a date filter was only possible for the INAHTA database and only by year, rather than exact date. Search strategies for these sources are outlined in Section 1.1.3 below.

1.1.2.4 Other sources

Other sources of completed or ongoing studies included the following, which were reviewed in September 2024 and again in June 2025:

- Manufacturer submissions and websites – for manufacturers participating and supplying a submission as part of the NICE assessment, these submissions were reviewed for mentions of published and ongoing clinical trials relating to the AI systems

of interest and considered them for inclusion in the review. Manufacturer websites were also reviewed with the same aim;

- US Food and Drug Administration (FDA) website – the term "colonoscop*" was used to search the FDA website for any relevant records relating to this assessment.
- Reference lists of included papers and of relevant SLRs were reviewed to identify any additional studies that may have been relevant for inclusion in the review.

1.1.3 Full search strategies

1.1.3.1 MEDLINE via Ovid

Supplementary Table 5. Search strategy for Medline via Ovid – clinical SLR – 04/09/24

#	Searches	Results (04/09/24)
1	Colonoscopy/	32,659
2	Sigmoidoscopy/	4,921
3	Proctoscopy/	2,135
4	(colonoscop* or polypect* or sigmoidoscop* or proctoscop* or coloscop* or ileocolonoscop* or anoscop* or rectoscop* or proctosigmoidoscop*).tw,kf.	49,554
5	1 or 2 or 3 or 4	64,059
6	Endoscopy, Gastrointestinal/	21,819
7	endoscop*.tw,kf.	268,038
8	6 or 7	273,432
9	exp intestine, large/	151,984
10	lower gastrointestinal tract/	205
11	(colon or colons or colonic or sigmoid or sigmoids or rectum* or rectal or colorect* or anus or anal or cecum or caecum or cecal or caecal).tw,kf.	587,177
12	(lower bowel* or lower intestin* or lower gastrointestin* or lower gastro-intestin* or lower GI or large bowel* or large intestin*).tw,kf.	32,093
13	9 or 10 or 11 or 12	641,888
14	8 and 13	33,941
15	5 or 14	82,428
16	exp Artificial Intelligence/	207,269
17	exp Machine Learning/	74,885
18	Deep Learning/	22,261
19	((artificial or machine* or comput* or augment* or amplif*) adj2 intelligen*).tw,kf.	59,607
20	AI.tw,kf.	60,878
21	((machine or deep or transfer* or hierarch* or computer) adj2 (learn* or reasoning)).tw,kf.	186,925
22	Sentiment Analysis/	213
23	("sentiment analysis" or "opinion mining").tw,kf.	2,066

24	Support Vector Machine/	10,826
25	(vector adj2 machine).tw,kf.	24,540
26	neural networks, computer/	54,795
27	((neural or convolut* or artificial) adj2 network).tw,kf.	85,230
28	(CNN or CNNs or ANN or ANNs).tw,kf.	41,098
29	"neural net".tw,kf.	629
30	Natural Language Processing/	7,102
31	(natural adj2 language adj2 process*).tw,kf.	10,461
32	"large language model".tw,kf.	1,049
33	("cognitive computing" or "computer vision").tw,kf.	10,142
34	Image Processing, Computer-Assisted/	144,869
35	Pattern Recognition, Automated/	26,697
36	Image Interpretation, Computer-Assisted/	48,670
37	Diagnosis, Computer-Assisted/	24,510
38	((computer or machine) adj1 (aid* or base* or assist* or support*)).tw,kf.	80,461
39	"CADe".tw,kf.	453
40	"CADx".tw,kf.	307
41	16 or 17 or 18 or 19 or 20 or 21 or 22 or 23 or 24 or 25 or 26 or 27 or 28 or 29 or 30 or 31 or 32 or 33 or 34 or 35 or 36 or 37 or 38 or 39 or 40	668,045
42	15 and 41	2,450
43	(GI Genius or GIGenius or ENDO-AID or ENDOAID or WISE VISION or WISEVISION or CAD-EYE or CADEYE or MAGENTIQ or EndoAngel or Endo-Angel or CADDIE or Endoscreener or Endo-screener).tw,kf.	92
44	(Discovery and Pentax).tw,kf.	2
45	(Argus or EMIS or Endoscopic Multimedia Information System).tw,kf.	1,622
46	45 and 22	0
47	43 or 44 or 46	94
48	42 or 47	2,491
49	exp animals/ not humans/	5,254,851
50	48 not 49	2,441
51	50	2,441
52	limit 51 to yr="2010 -Current"	1,907

Database(s): Ovid MEDLINE(R) ALL 1946 to September 03, 2024.

Note that an error in this search strategy was later identified (line 46 mistakenly combines line 45 with line 22, whereas the intention was to combine line 45 with line 15). The impact of this was reviewed and there was no major impact on the search results. Given much of the deduplication and sifting of database records had already occurred by this stage, updated results from the corrected search strategy were not incorporated into the sift. The search strategy was corrected for the update searches performed in June 2025 (see Supplementary Table 6).

Abbreviations: SLR, systematic literature review.

Supplementary Table 6. Search strategy for Medline via Ovid – clinical SLR – 11/06/25 update

#	Searches	Results (11/06/25)
1	Colonoscopy/	33,577
2	Sigmoidoscopy/	4,943
3	Proctoscopy/	2,143
4	(colonoscop* or polypect* or sigmoidoscop* or proctoscop* or coloscop* or ileocolonoscop* or anoscop* or rectoscop* or proctosigmoidoscop*).tw,kf.	51,681
5	1 or 2 or 3 or 4	66,321
6	Endoscopy, Gastrointestinal/	22,130
7	endoscop*.tw,kf.	280,239
8	6 or 7	285,637
9	exp intestine, large/	154,395
10	lower gastrointestinal tract/	208
11	(colon or colons or colonic or sigmoid or sigmoids or rectum* or rectal or colorect* or anus or anal or cecum or caecum or cecal or caecal).tw,kf.	611,191
12	(lower bowel* or lower intestin* or lower gastrointestin* or lower gastro-intestin* or lower GI or large bowel* or large intestin*).tw,kf.	32,948
13	9 or 10 or 11 or 12	666,550
14	8 and 13	35,520
15	5 or 14	85,589
16	exp Artificial Intelligence/	239,761
17	exp Machine Learning/	95,790
18	Deep Learning/	29,999
19	((artificial or machine* or comput* or augment* or amplif*) adj2 intelligen*).tw,kf.	79,536
20	AI.tw,kf.	77,152
21	((machine or deep or transfer* or hierarch* or computer) adj2 (learn* or reasoning)).tw,kf.	229,247
22	Sentiment Analysis/	214
23	("sentiment analysis" or "opinion mining").tw,kf.	2,449
24	Support Vector Machine/	12,037
25	(vector adj2 machine).tw,kf.	27,527
26	neural networks, computer/	62,326
27	((neural or convolut* or artificial) adj2 network).tw,kf.	96,165
28	(CNN or CNNs or ANN or ANNs).tw,kf.	46,698
29	"neural net".tw,kf.	656
30	Natural Language Processing/	8,110
31	(natural adj2 language adj2 process*).tw,kf.	12,390
32	"large language model".tw,kf.	2,317
33	("cognitive computing" or "computer vision").tw,kf.	11,766
34	Image Processing, Computer-Assisted/	150,388
35	Pattern Recognition, Automated/	26,949
36	Image Interpretation, Computer-Assisted/	50,338

37	Diagnosis, Computer-Assisted/	24,914
38	((computer or machine) adj1 (aid* or base* or assist* or support*)).tw,kf.	84,269
39	"CADe".tw,kf.	512
40	"CADx".tw,kf.	345
41	16 or 17 or 18 or 19 or 20 or 21 or 22 or 23 or 24 or 25 or 26 or 27 or 28 or 29 or 30 or 31 or 32 or 33 or 34 or 35 or 36 or 37 or 38 or 39 or 40	743,294
42	15 and 41	2,734
43	(GI Genius or GIGenius or ENDO-AID or ENDOAID or WISE VISION or WISEVISION or CAD-EYE or CADEYE or MAGENTIQ or EndoAngel or Endo-Angel or CADDIE or Endoscreener or Endo-screener).tw,kf.	100
44	(Discovery and Pentax).tw,kf.	2
45	(Argus or EMIS or Endoscopic Multimedia Information System).tw,kf.	1,721
46	45 and 15	2
47	43 or 44 or 46	104
48	42 or 47	2,778
49	exp animals/ not humans/	5,348,122
50	48 not 49	2,727
51	50	2,727
52	limit 51 to yr="2010 -Current"	2,192
53	limit 52 to dt=20240904-20250611	294
Database(s): Ovid MEDLINE(R) ALL 1946 to June 10, 2025.<br>Abbreviations: SLR, systematic literature review.		

1.1.3.2 Embase via Ovid

Supplementary Table 7. Search strategy for Embase via Ovid – clinical SLR – 04/09/24

#	Searches	Results (04/09/24)
1	colonoscopy/	110,793
2	exp polypectomy/	12,434
3	exp endoscopic polypectomy/	3,187
4	sigmoidoscopy/	14,766
5	rectoscopy/	2,952
6	ileocolonoscopy/	1,635
7	(colonoscop* or polypect* or sigmoidoscop* or proctoscop* or coloscop* or ileocolonoscop* or anoscop* or rectoscop* or proctosigmoidoscop*).tw,kf.	97,412
8	1 or 2 or 3 or 4 or 5 or 6 or 7	145,407
9	gastrointestinal endoscopy/	42,803
10	endoscop*.tw,kf.	435,863
11	9 or 10	450,740
12	exp large intestine/	213,408

13	sigmoid/	20,917
14	lower gastrointestinal tract/	912
15	exp rectum/	43,520
16	exp anus/	22,970
17	cecum/	22,320
18	(colon or colons or colonic or sigmoid or sigmoids or rectum* or rectal or colorect* or anus or anal or cecum or caecum or cecal or caecal).tw,kf.	835,980
19	(lower bowel* or lower intestin* or lower gastrointestin* or lower gastro-intestin* or lower GI or large bowel* or large intestin*).tw,kf.	42,805
20	12 or 13 or 14 or 15 or 16 or 17 or 18 or 19	912,945
21	11 and 20	71,477
22	8 or 21	181,653
23	artificial intelligence/	85,709
24	cognitive technology/	7
25	exp machine learning/	508,262
26	deep learning/	63,401
27	((artificial or machine* or comput* or augment* or amplif*) adj2 intelligen*).tw,kf.	68,904
28	AI.tw,kf.	80,413
29	((machine or deep or transfer* or hierarch* or computer) adj2 (learn* or reasoning)).tw,kf.	215,958
30	sentiment analysis/	891
31	("Sentiment analysis" or "opinion mining").tw,kf.	1,823
32	exp support vector machine/	47,617
33	(vector adj2 machine).tw,kf.	29,407
34	cognitive computing/	42
35	computer vision/	4,567
36	("cognitive computing" or "computer vision").tw,kf.	10,805
37	natural language processing/	13,790
38	(natural adj2 language* adj2 process*).tw,kf.	12,146
39	large language model/	2,136
40	"large language model".tw,kf.	1,067
41	artificial neural network/	60,059
42	convolutional neural network/	34,202
43	((neural or convolut* or artificial) adj2 network*).tw,kf.	143,046
44	(CNN or CNNs or ANN or ANNs).tw,kf.	99,364
45	"neural net".tw,kf.	795
46	computer analysis/	124,824
47	computer assisted diagnosis/	43,350
48	pattern recognition/	37,615
49	((computer or machine) adj1 (aid* or base* or assist* or support*)).tw,kf.	101,398
50	"CADe".tw,kf.	793
51	"CADx".tw,kf.	445

52	23 or 24 or 25 or 26 or 27 or 28 or 29 or 30 or 31 or 32 or 33 or 34 or 35 or 36 or 37 or 38 or 39 or 40 or 41 or 42 or 43 or 44 or 45 or 46 or 47 or 48 or 49 or 50 or 51	998,314
53	22 and 52	4,659
54	(GI Genius or GIGenius or ENDO-AID or ENDOAID or WISE VISION or WISEVISION or CAD-EYE or CADEYE or MAGENTIQ or EndoAngel or Endo-Angel or CADDIE or Endoscreener or Endo-screener).tw,kf.	304
55	(Discovery and Pentax).tw,kf.	7
56	(Argus or EMIS or Endoscopic Multimedia Information System).tw,kf.	2,242
57	56 and 22	14
58	54 or 55 or 57	325
59	53 or 58	4,782
60	exp animals/ not humans/	11,821,428
61	59 not 60	4,128
62	limit 61 to yr="2010 -Current"	3,661

Database(s): Embase 1974 to 2024 September 03.
Abbreviations: SLR, systematic literature review.

Supplementary Table 8. Search strategy for Embase via Ovid – clinical SLR – 11/06/25 update

#	Searches	Results (11/06/25)
1	colonoscopy/	120,386
2	exp polypectomy/	13,601
3	exp endoscopic polypectomy/	3,296
4	sigmoidoscopy/	15,765
5	rectoscopy/	3,097
6	ileocolonoscopy/	1,869
7	(colonoscop* or polypect* or sigmoidoscop* or proctoscop* or coloscop* or ileocolonoscop* or anoscop* or rectoscop* or proctosigmoidoscop*).tw,kf.	105,954
8	1 or 2 or 3 or 4 or 5 or 6 or 7	156,564
9	gastrointestinal endoscopy/	44,766
10	endoscop*.tw,kf.	464,716
11	9 or 10	480,133
12	exp large intestine/	223,197
13	sigmoid/	22,016
14	lower gastrointestinal tract/	1,067
15	exp rectum/	45,913
16	exp anus/	24,291
17	cecum/	24,211
18	(colon or colons or colonic or sigmoid or sigmoids or rectum* or rectal or colorect* or anus or anal or cecum or caecum or cecal or caecal).tw,kf.	885,731
19	(lower bowel* or lower intestin* or lower gastrointestin* or lower gastro-intestin* or lower GI or large bowel* or large intestin*).tw,kf.	44,855

20	12 or 13 or 14 or 15 or 16 or 17 or 18 or 19	965,255
21	11 and 20	77,482
22	8 or 21	195,484
23	artificial intelligence/	108,730
24	cognitive technology/	15
25	exp machine learning/	596,046
26	deep learning/	82,092
27	((artificial or machine* or comput* or augment* or amplif*) adj2 intelligen*).tw,kf.	91,541
28	AI.tw,kf.	102,010
29	((machine or deep or transfer* or hierarch* or computer) adj2 (learn* or reasoning)).tw,kf.	262,641
30	sentiment analysis/	1,168
31	("Sentiment analysis" or "opinion mining").tw,kf.	2,122
32	exp support vector machine/	55,073
33	(vector adj2 machine).tw,kf.	32,464
34	cognitive computing/	52
35	computer vision/	6,010
36	("cognitive computing" or "computer vision").tw,kf.	12,514
37	natural language processing/	16,550
38	(natural adj2 language* adj2 process*).tw,kf.	14,243
39	large language model/	5,468
40	"large language model".tw,kf.	2,496
41	artificial neural network/	67,576
42	convolutional neural network/	41,409
43	((neural or convolut* or artificial) adj2 network*).tw,kf.	160,166
44	(CNN or CNNs or ANN or ANNs).tw,kf.	106,197
45	"neural net".tw,kf.	828
46	computer analysis/	125,382
47	computer assisted diagnosis/	42,335
48	pattern recognition/	38,316
49	((computer or machine) adj1 (aid* or base* or assist* or support*)).tw,kf.	107,844
50	"CADe".tw,kf.	1,004
51	"CADx".tw,kf.	528
52	23 or 24 or 25 or 26 or 27 or 28 or 29 or 30 or 31 or 32 or 33 or 34 or 35 or 36 or 37 or 38 or 39 or 40 or 41 or 42 or 43 or 44 or 45 or 46 or 47 or 48 or 49 or 50 or 51	1,120,058
53	22 and 52	5,707
54	(GI Genius or GIGenius or ENDO-AID or ENDOAID or WISE VISION or WISEVISION or CAD-EYE or CADEYE or MAGENTIQ or EndoAngel or Endo-Angel or CADDIE or Endoscreener or Endo-screener).tw,kf.	439
55	(Discovery and Pentax).tw,kf.	10
56	(Argus or EMIS or Endoscopic Multimedia Information System).tw,kf.	2,391
57	56 and 22	15
58	54 or 55 or 57	464

59	53 or 58	5,853
60	exp animals/ not humans/	5,928,890
61	59 not 60	5,809
62	limit 61 to yr="2010 -Current"	5,292
63	limit 62 to dc=20240904-20250611	1,147
Database(s): Embase 1974 to 2025 June 10.<br>Abbreviations: SLR, systematic literature review.		

1.1.3.3 CENTRAL via Cochrane Library

Supplementary Table 9. Search strategy for CENTRAL via Cochrane Library – clinical SLR – 04/09/24

#	Searches	Results (04/09/24)
#1	MeSH descriptor: [Colonoscopy] explode all trees	3065
#2	MeSH descriptor: [Sigmoidoscopy] explode all trees	368
#3	MeSH descriptor: [Proctoscopy] explode all trees	107
#4	(colonoscop* OR polypect* OR sigmoidoscop* OR proctoscop* OR coloscop* OR ileocolonoscop* OR anoscop* OR rectoscop* OR proctosigmoidoscop*)	11450
#5	(#1 OR #2 OR #3 OR #4)	11489
#6	MeSH descriptor: [Endoscopy, Gastrointestinal] this term only	1072
#7	endoscop*	40419
#8	(#6 OR #7)	40419
#9	MeSH descriptor: [Intestine, Large] explode all trees	4361
#10	MeSH descriptor: [Lower Gastrointestinal Tract] this term only	11
#11	(colon OR colons OR colonic OR sigmoid OR sigmoids OR rectum* OR rectal OR colorect* OR anus OR anal OR cecum OR caecum OR cecal OR caecal)	59710
#12	(lower bowel* OR lower intestin* OR lower gastrointestin* OR lower gastro-intestin* OR lower GI OR large bowel* OR large intestin*)	23750
#13	(#9 or #10 or #11 or #12)	77938
#14	(#8 AND #13)	9560
#15	(#5 OR #14)	16802
#16	MeSH descriptor: [Artificial Intelligence] explode all trees	3279
#17	MeSH descriptor: [Machine Learning] explode all trees	1009
#18	MeSH descriptor: [Deep Learning] explode all trees	331
#19	((artificial OR machine* OR comput* OR augment* OR amplif*) NEAR/2 intelligen*)	2573
#20	AI	11382
#21	((machine OR deep OR transfer* OR hierarch* OR computer) NEAR/2 (learn* OR reasoning))	4994
#22	MeSH descriptor: [Sentiment Analysis] this term only	0
#23	"Sentiment analysis" OR "opinion mining"	18

#24	MeSH descriptor: [Support Vector Machine] this term only	63
#25	(vector NEAR/2 machine)	548
#26	"cognitive computing" OR "computer vision"	180
#27	MeSH descriptor: [Natural Language Processing] this term only	73
#28	(natural NEAR/2 language* NEAR/2 process*)	288
#29	"large language model"	24
#30	MeSH descriptor: [Neural Networks, Computer] explode all trees	641
#31	((neural OR convolut* OR artificial) NEAR/2 network*)	2168
#32	(CNN OR CNNs OR ANN OR ANNs)	5234
#33	"neural net"	15
#34	MeSH descriptor: [Diagnosis, Computer-Assisted] this term only	809
#35	MeSH descriptor: [Image Processing, Computer-Assisted] this term only	2373
#36	MeSH descriptor: [Pattern Recognition, Automated] this term only	237
#37	MeSH descriptor: [Image Interpretation, Computer-Assisted] this term only	1080
#38	((computer OR machine) NEXT (aid* OR base* OR assist* OR support*))	26739
#39	"CADe" OR "CADx"	341
#40	(#16 OR #17 OR #18 OR #19 OR #20 OR #21 OR #22 OR #23 OR #24 OR #25 OR #26 OR #27 OR #28 OR #29 OR #30 OR #31 OR #32 OR #33 OR #34 OR #35 OR #36 OR #37 OR #38 OR #39)	50015
#41	(#15 AND #40)	913
#42	(GI Genius OR GIGenius OR ENDO-AID OR ENDOAID OR WISE VISION OR WISEVISION OR CAD-EYE OR CADEYE OR MAGENTIQ OR EndoAngel OR Endo-Angel OR CADDIE OR Endoscreener OR Endo-screener)	347
#43	(Discovery AND Pentax)	5
#44	(Argus OR EMIS OR Endoscopic Multimedia Information System)	133
#45	(#44 AND #15)	2
#46	(#42 OR #43 OR #45)	354
#47	(#41 OR #46)	1125
#48	#47 with Publication Year from 2010 to 2024, in Trials	782

Database(s): Cochrane Library, filtered for trials using “Trials” selection under “Content Type” filter of “limits” panel.
Abbreviations: CENTRAL, Cochrane Central Register of Controlled Trials; SLR, systematic literature review.

Supplementary Table 10. Search strategy for CENTRAL via Cochrane Library – clinical SLR – 11/06/25 update

#	Searches	Results (11/06/25)
#1	MeSH descriptor: [Colonoscopy] explode all trees	3015
#2	MeSH descriptor: [Sigmoidoscopy] explode all trees	359
#3	MeSH descriptor: [Proctoscopy] explode all trees	96
#4	(colonoscop* OR polypect* OR sigmoidoscop* OR proctoscop* OR coloscop* OR ileocolonoscop* OR anoscop* OR rectoscop* OR proctosigmoidoscop*)	11803
#5	(#1 OR #2 OR #3 OR #4)	11837

#6	MeSH descriptor: [Endoscopy, Gastrointestinal] this term only	1063
#7	endoscop*	41772
#8	(#6 OR #7)	41772
#9	MeSH descriptor: [Intestine, Large] explode all trees	4316
#10	MeSH descriptor: [Lower Gastrointestinal Tract] this term only	11
#11	(colon OR colons OR colonic OR sigmoid OR sigmoids OR rectum* OR rectal OR colorect* OR anus OR anal OR cecum OR caecum OR cecal OR caecal)	61438
#12	(lower bowel* OR lower intestin* OR lower gastrointestin* OR lower gastro-intestin* OR lower GI OR large bowel* OR large intestin*)	24248
#13	(#9 or #10 or #11 or #12)	80027
#14	(#8 AND #13)	9892
#15	(#5 OR #14)	17383
#16	MeSH descriptor: [Artificial Intelligence] explode all trees	3565
#17	MeSH descriptor: [Machine Learning] explode all trees	1156
#18	MeSH descriptor: [Deep Learning] explode all trees	376
#19	((artificial OR machine* OR comput* OR augment* OR amplif*) NEAR/2 intelligen*)	3269
#20	AI	12394
#21	((machine OR deep OR transfer* OR hierarch* OR computer) NEAR/2 (learn* OR reasoning))	5503
#22	MeSH descriptor: [Sentiment Analysis] this term only	0
#23	"Sentiment analysis" OR "opinion mining"	19
#24	MeSH descriptor: [Support Vector Machine] this term only	73
#25	(vector NEAR/2 machine)	586
#26	"cognitive computing" OR "computer vision"	218
#27	MeSH descriptor: [Natural Language Processing] this term only	78
#28	(natural NEAR/2 language* NEAR/2 process*)	322
#29	"large language model"	86
#30	MeSH descriptor: [Neural Networks, Computer] explode all trees	686
#31	((neural OR convolut* OR artificial) NEAR/2 network*)	2246
#32	(CNN OR CNNs OR ANN OR ANNs)	5358
#33	"neural net"	16
#34	MeSH descriptor: [Diagnosis, Computer-Assisted] this term only	815
#35	MeSH descriptor: [Image Processing, Computer-Assisted] this term only	2340
#36	MeSH descriptor: [Pattern Recognition, Automated] this term only	238
#37	MeSH descriptor: [Image Interpretation, Computer-Assisted] this term only	1089
#38	((computer OR machine) NEXT (aid* OR base* OR assist* OR support*))	27105
#39	"CADe" OR "CADx"	384
#40	(#16 OR #17 OR #18 OR #19 OR #20 OR #21 OR #22 OR #23 OR #24 OR #25 OR #26 OR #27 OR #28 OR #29 OR #30 OR #31 OR #32 OR #33 OR #34 OR #35 OR #36 OR #37 OR #38 OR #39)	52131
#41	(#15 AND #40)	994

#42	(GI Genius OR GIGenius OR ENDO-AID OR ENDOAID OR WISE VISION OR WISEVISION OR CAD-EYE OR CADEYE OR MAGENTIQ OR EndoAngel OR Endo-Angel OR CADDIE OR Endoscreener OR Endo-screener)	384
#43	(Discovery AND Pentax)	5
#44	(Argus OR EMIS OR Endoscopic Multimedia Information System)	135
#45	(#44 AND #15)	3
#46	(#42 OR #43 OR #45)	392
#47	(#41 OR #46)	1225
#48	#47 with Publication Year from 2010 to 2025, in Trials	869
#49	#48 with Cochrane Library publication date Between Sep 2024 and Jun 2025	108
Database(s): Cochrane Library, filtered for trials using “Trials” selection under “Content Type” filter of “limits” panel. Abbreviations: CENTRAL, Cochrane Central Register of Controlled Trials; SLR, systematic literature review.		

1.1.3.4 CDSR via Cochrane Library

Supplementary Table 11. Search strategy for CDSR via Cochrane Library – clinical SLR – 04/09/24

#	Searches	Results (04/09/24)
#1	MeSH descriptor: [Colonoscopy] explode all trees	3065
#2	MeSH descriptor: [Sigmoidoscopy] explode all trees	368
#3	MeSH descriptor: [Proctoscopy] explode all trees	107
#4	(colonoscop* OR polypect* OR sigmoidoscop* OR proctoscop* OR coloscop* OR ileocolonoscop* OR anoscop* OR rectoscop* OR proctosigmoidoscop*)	11450
#5	(#1 OR #2 OR #3 OR #4)	11489
#6	MeSH descriptor: [Endoscopy, Gastrointestinal] this term only	1072
#7	endoscop*	40419
#8	(#6 OR #7)	40419
#9	MeSH descriptor: [Intestine, Large] explode all trees	4361
#10	MeSH descriptor: [Lower Gastrointestinal Tract] this term only	11
#11	(colon OR colons OR colonic OR sigmoid OR sigmoids OR rectum* OR rectal OR colorect* OR anus OR anal OR cecum OR caecum OR cecal OR caecal)	59710
#12	(lower bowel* OR lower intestin* OR lower gastrointestin* OR lower gastro-intestin* OR lower GI OR large bowel* OR large intestin*)	23750
#13	(#9 or #10 or #11 or #12)	77938
#14	(#8 AND #13)	9560
#15	(#5 OR #14)	16802
#16	MeSH descriptor: [Artificial Intelligence] explode all trees	3279
#17	MeSH descriptor: [Machine Learning] explode all trees	1009
#18	MeSH descriptor: [Deep Learning] explode all trees	331
#19	((artificial OR machine* OR comput* OR augment* OR amplif*) NEAR/2 intelligen*)	2573
#20	AI	11382

#21	((machine OR deep OR transfer* OR hierarch* OR computer) NEAR/2 (learn* OR reasoning))	4994
#22	MeSH descriptor: [Sentiment Analysis] this term only	0
#23	"Sentiment analysis" OR "opinion mining"	18
#24	MeSH descriptor: [Support Vector Machine] this term only	63
#25	(vector NEAR/2 machine)	548
#26	"cognitive computing" OR "computer vision"	180
#27	MeSH descriptor: [Natural Language Processing] this term only	73
#28	(natural NEAR/2 language* NEAR/2 process*)	288
#29	"large language model"	24
#30	MeSH descriptor: [Neural Networks, Computer] explode all trees	641
#31	((neural OR convolut* OR artificial) NEAR/2 network*)	2168
#32	(CNN OR CNNs OR ANN OR ANNs)	5234
#33	"neural net"	15
#34	MeSH descriptor: [Diagnosis, Computer-Assisted] this term only	809
#35	MeSH descriptor: [Image Processing, Computer-Assisted] this term only	2373
#36	MeSH descriptor: [Pattern Recognition, Automated] this term only	237
#37	MeSH descriptor: [Image Interpretation, Computer-Assisted] this term only	1080
#38	((computer OR machine) NEXT (aid* OR base* OR assist* OR support*))	26739
#39	"CADe" OR "CADx"	341
#40	(#16 OR #17 OR #18 OR #19 OR #20 OR #21 OR #22 OR #23 OR #24 OR #25 OR #26 OR #27 OR #28 OR #29 OR #30 OR #31 OR #32 OR #33 OR #34 OR #35 OR #36 OR #37 OR #38 OR #39)	50015
#41	(#15 AND #40)	913
#42	(GI Genius OR GIGenius OR ENDO-AID OR ENDOAID OR WISE VISION OR WISEVISION OR CAD-EYE OR CADEYE OR MAGENTIQ OR EndoAngel OR Endo-Angel OR CADDIE OR Endoscreener OR Endo-screener)	347
#43	(Discovery AND Pentax)	5
#44	(Argus OR EMIS OR Endoscopic Multimedia Information System)	133
#45	(#44 AND #15)	2
#46	(#42 OR #43 OR #45)	354
#47	(#41 OR #46)	1125
#48	#47 with Cochrane Library publication date Between Jan 2010 and Sep 2024, in Cochrane Reviews	182

Database(s): Cochrane Library, filtered for systematic reviews using “Cochrane Reviews” selection under “Content Type” filter of “limits” panel.

Abbreviations: CDSR, Cochrane Database of Systematic Reviews; SLR, systematic literature review.

Supplementary Table 12. Search strategy for CDSR via Cochrane Library – clinical SLR – 11/06/25 update

#	Searches	Results (11/06/25)
#1	MeSH descriptor: [Colonoscopy] explode all trees	3015
#2	MeSH descriptor: [Sigmoidoscopy] explode all trees	359
#3	MeSH descriptor: [Proctoscopy] explode all trees	96
#4	(colonoscop* OR polypect* OR sigmoidoscop* OR proctoscop* OR coloscop* OR ileocolonoscop* OR anoscop* OR rectoscop* OR proctosigmoidoscop*)	11803
#5	(#1 OR #2 OR #3 OR #4)	11837
#6	MeSH descriptor: [Endoscopy, Gastrointestinal] this term only	1063
#7	endoscop*	41772
#8	(#6 OR #7)	41772
#9	MeSH descriptor: [Intestine, Large] explode all trees	4316
#10	MeSH descriptor: [Lower Gastrointestinal Tract] this term only	11
#11	(colon OR colons OR colonic OR sigmoid OR sigmoids OR rectum* OR rectal OR colorect* OR anus OR anal OR cecum OR caecum OR cecal OR caecal)	61438
#12	(lower bowel* OR lower intestin* OR lower gastrointestin* OR lower gastro-intestin* OR lower GI OR large bowel* OR large intestin*)	24248
#13	(#9 or #10 or #11 or #12)	80027
#14	(#8 AND #13)	9892
#15	(#5 OR #14)	17383
#16	MeSH descriptor: [Artificial Intelligence] explode all trees	3565
#17	MeSH descriptor: [Machine Learning] explode all trees	1156
#18	MeSH descriptor: [Deep Learning] explode all trees	376
#19	((artificial OR machine* OR comput* OR augment* OR amplif*) NEAR/2 intelligen*)	3269
#20	AI	12394
#21	((machine OR deep OR transfer* OR hierarch* OR computer) NEAR/2 (learn* OR reasoning))	5503
#22	MeSH descriptor: [Sentiment Analysis] this term only	0
#23	"Sentiment analysis" OR "opinion mining"	19
#24	MeSH descriptor: [Support Vector Machine] this term only	73
#25	(vector NEAR/2 machine)	586
#26	"cognitive computing" OR "computer vision"	218
#27	MeSH descriptor: [Natural Language Processing] this term only	78
#28	(natural NEAR/2 language* NEAR/2 process*)	322
#29	"large language model"	86
#30	MeSH descriptor: [Neural Networks, Computer] explode all trees	686
#31	((neural OR convolut* OR artificial) NEAR/2 network*)	2246
#32	(CNN OR CNNs OR ANN OR ANNs)	5358
#33	"neural net"	16
#34	MeSH descriptor: [Diagnosis, Computer-Assisted] this term only	815

#35	MeSH descriptor: [Image Processing, Computer-Assisted] this term only	2340
#36	MeSH descriptor: [Pattern Recognition, Automated] this term only	238
#37	MeSH descriptor: [Image Interpretation, Computer-Assisted] this term only	1089
#38	((computer OR machine) NEXT (aid* OR base* OR assist* OR support*))	27105
#39	"CADe" OR "CADx"	384
#40	(#16 OR #17 OR #18 OR #19 OR #20 OR #21 OR #22 OR #23 OR #24 OR #25 OR #26 OR #27 OR #28 OR #29 OR #30 OR #31 OR #32 OR #33 OR #34 OR #35 OR #36 OR #37 OR #38 OR #39)	52131
#41	(#15 AND #40)	994
#42	(GI Genius OR GIGenius OR ENDO-AID OR ENDOAID OR WISE VISION OR WISEVISION OR CAD-EYE OR CADEYE OR MAGENTIQ OR EndoAngel OR Endo-Angel OR CADDIE OR Endoscreener OR Endo-screener)	384
#43	(Discovery AND Pentax)	5
#44	(Argus OR EMIS OR Endoscopic Multimedia Information System)	135
#45	(#44 AND #15)	3
#46	(#42 OR #43 OR #45)	392
#47	(#41 OR #46)	1225
#48	#47 with Cochrane Library publication date Between Jan 2010 and Jun 2025, in Cochrane Reviews	190
#49	#48 with Cochrane Library publication date Between Sep 2024 and Jun 2025	11

Database(s): Cochrane Library, filtered for systematic reviews using “Cochrane Reviews” selection under “Content Type” filter of “limits” panel.

Abbreviations: CDSR, Cochrane Database of Systematic Reviews; SLR, systematic literature review.

1.1.3.5 WHO ICTRP

The search strategy for WHO ICTRP was (colonoscop* or polypect* or sigmoidoscop* or proctoscop* or coloscop* or ileocolonoscop* or anoscop* or rectoscop* or proctosigmoidoscop* or endoscop*) AND (AI or artificial or intelligen* or machine or learn* or neural or computer* or CADe or CADx), entered into the title field with recruitment status set as “ALL”. For the update performed in June 2025, a date limit of 14/09/24 to 11/06/25 was added to the “date of registration” field.

1.1.3.6 Clinicaltrials.gov

For Clinicaltrials.gov, the following search strategy was used:

- Intervention field: colonoscopy OR polypectomy OR sigmoidoscopy OR proctoscopy OR coloscopy OR ileocolonoscopy OR anoscopy OR rectoscopy OR proctosigmoidoscopy OR endoscopy
- Other terms field: AI OR artificial OR intelligence OR intelligent OR machine OR learning OR neural OR computer OR computerised OR computerized OR CADe OR CADx

For the update in June 2025, a date limit of 16/09/24 to 11/06/25 was added to the "results first posted" field.

1.1.3.7 PROSPERO

Supplementary Table 13. Search strategy for PROSPERO – clinical SLR – 15/09/24

#	Searches	Results (15/09/24)
1	MeSH DESCRIPTOR colonoscopy	112
2	MeSH DESCRIPTOR sigmoidoscopy	5
3	MeSH DESCRIPTOR proctoscopy	1
4	(colonoscop* or polypect* or sigmoidoscop* or proctoscop* or coloscop* or ileocolonoscop* or anoscop* or rectoscop* or proctosigmoidoscop*)	1038
5	#1 OR #2 OR #3 OR #4	1048
6	MeSH DESCRIPTOR Endoscopy, Gastrointestinal	39
7	endoscop*	5018
8	#6 OR #7	5018
9	MeSH DESCRIPTOR Intestine, large EXPLODE ALL TREES	184
10	MeSH DESCRIPTOR lower gastrointestinal tract	2
11	(colon or colons or colonic or sigmoid or sigmoids or rectum* or rectal or colorect* or anus or anal or cecum or caecum or cecal or caecal)	7599
12	(lower bowel* or lower intestin* or lower gastrointestin* or lower gastro-intestin* or lower GI or large bowel* or large intestin*)	431
13	#9 OR #10 OR #11 OR #12	7787
14	#8 AND #13	957
15	#5 OR #14	1635
16	MeSH DESCRIPTOR artificial intelligence EXPLODE ALL TREES	477
17	MeSH DESCRIPTOR machine learning EXPLODE ALL TREES	154
18	MeSH DESCRIPTOR deep learning	23
19	((artificial or machine* or comput* or augment* or amplif*) adj2 intelligen*)	2870
20	AI	4607
21	((machine or deep or transfer* or hierarch* or computer) adj2 (learn* or reasoning))	3305
22	"sentiment analysis" or "opinion mining"	25
23	MeSH DESCRIPTOR support vector machine	0

24	(vector adj2 machine)	179
25	MeSH DESCRIPTOR neural networks, computer	6
26	((neural or convolut* or artificial) adj2 network)	585
27	(CNN or CNNs or ANN or ANNs)	2488
28	"neural net"	5
29	MeSH DESCRIPTOR natural language processing	8
30	(natural adj2 language adj2 process*)	247
31	"large language model"	33
32	("cognitive computing" or "computer vision")	162
33	MeSH DESCRIPTOR image processing, computer-assisted	16
34	MeSH DESCRIPTOR pattern recognition, automated	1
35	MeSH DESCRIPTOR image interpretation, computer-assisted	4
36	MeSH DESCRIPTOR diagnosis, computer-assisted	9
37	((computer or machine) adj1 (aid* or base* or assist* or support*))	2148
38	"CADe"	68
39	"CADx"	8
40	#16 OR #17 OR #18 OR #19 OR #20 OR #21 OR #22 OR #23 OR #24 OR #25 OR #26 OR #27 OR #28 OR #29 OR #30 OR #31 OR #32 OR #33 OR #35 OR #34 OR #36 OR #37 OR #38 OR #39	11273
41	#15 AND #40	152
42	(GI Genius or GIGenius or ENDO-AID or ENDOAID or WISE VISION or WISEVISION or CAD-EYE or CADEYE or MAGENTIQ or EndoAngel or Endo-Angel or CADDIE or Endoscreener or Endo-screener)	2
43	(Discovery and Pentax)	1
44	(Argus or EMIS or Endoscopic Multimedia Information System)	26
45	#44 AND #15	1
46	#42 OR #43 OR #45	2
47	#41 OR #46	153
Abbreviations: SLR, systematic literature review.		

Supplementary Table 14. Search strategy for PROSPERO – clinical SLR – 11/06/25 update

#	Searches	Results (11/06/25)
1	MeSH DESCRIPTOR colonoscopy	479
2	MeSH DESCRIPTOR sigmoidoscopy	32
3	MeSH DESCRIPTOR proctoscopy	3
4	(colonoscop* or polypect* or sigmoidoscop* or proctoscop* or coloscop* or ileocolonoscop* or anoscop* or rectoscop* or proctosigmoidoscop*)	1331
5	#1 OR #2 OR #3 OR #4	1331
6	MeSH DESCRIPTOR Endoscopy, Gastrointestinal	208
7	endoscop*	6269

8	#6 OR #7	6269
9	MeSH DESCRIPTOR Intestine, large EXPLODE ALL TREES	563
10	MeSH DESCRIPTOR lower gastrointestinal tract	2
11	(colon or colons or colonic or sigmoid or sigmoids or rectum* or rectal or colorect* or anus or anal or cecum or caecum or cecal or caecal)	9354
12	(lower bowel* or lower intestin* or lower gastrointestin* or lower gastro-intestin* or lower GI or large bowel* or large intestin*)	484
13	#9 OR #10 OR #11 OR #12	9571
14	#8 AND #13	1170
15	#5 OR #14	2027
16	MeSH DESCRIPTOR artificial intelligence EXPLODE ALL TREES	4696
17	MeSH DESCRIPTOR machine learning EXPLODE ALL TREES	1701
18	MeSH DESCRIPTOR deep learning	515
19	((artificial or machine* or comput* or augment* or amplif*) adj2 intelligen*)	4880
20	AI	5287
21	((machine or deep or transfer* or hierarch* or computer) adj2 (learn* or reasoning))	5087
22	"sentiment analysis" or "opinion mining"	38
23	MeSH DESCRIPTOR support vector machine	42
24	(vector adj2 machine)	224
25	MeSH DESCRIPTOR neural networks, computer	204
26	((neural or convolut* or artificial) adj2 network)	700
27	(CNN or CNNs or ANN or ANNs)	2678
28	"neural net"	4
29	MeSH DESCRIPTOR natural language processing	102
30	(natural adj2 language adj2 process*)	416
31	"large language model"	95
32	("cognitive computing" or "computer vision")	220
33	MeSH DESCRIPTOR image processing, computer-assisted	77
34	MeSH DESCRIPTOR pattern recognition, automated	9
35	MeSH DESCRIPTOR image interpretation, computer-assisted	16
36	MeSH DESCRIPTOR diagnosis, computer-assisted	40
37	((computer or machine) adj1 (aid* or base* or assist* or support*))	2580
38	"CADe"	74
39	"CADx"	12
40	#16 OR #17 OR #18 OR #19 OR #20 OR #21 OR #22 OR #23 OR #24 OR #25 OR #26 OR #27 OR #28 OR #29 OR #30 OR #31 OR #32 OR #33 OR #35 OR #34 OR #36 OR #37 OR #38 OR #39	14264
41	#15 AND #40	193
42	(GI Genius or GIGenius or ENDO-AID or ENDOAID or WISE VISION or WISEVISION or CAD-EYE or CADEYE or MAGENTIQ or EndoAngel or Endo-Angel or CADDIE or Endoscreener or Endo-screener)	5
43	(Discovery and Pentax)	1

44	(Argus or EMIS or Endoscopic Multimedia Information System)	31
45	#44 AND #15	1
46	#42 OR #43 OR #45	5
47	#41 OR #46	195

The 195 records identified were further filtered by adding a date limit from 16/09/24 to 11/06/25 to the "date of registration" field, leaving a total of 54 new records identified in this update from PROSPERO.
Abbreviations: SLR, systematic literature review.

1.1.3.8 Health technology assessment groups

Different approaches to searching these websites were taken depending on the number of records and the compatibility with certain ways of searching. NICE and HTW websites were searched using "colonoscop* or polypect* or sigmoidoscop* proctoscop* or coloscop* or ileocolonoscop* or anoscop* or rectoscop* or proctosigmoidoscop* or endoscop*". The full list of SIGN guidance was reviewed given there were fewer than 50 records, and the term "artificial" was searched on the website of Canada's Drug Agency (with "project line" set to "health technology review") given more complex strings did not appear to function (such as those used to search NICE and HTW websites) and the likelihood that relevant documents would mention AI somewhere in the record. For the update in June 2025, there was no way of restricting or filtering for only new records since the previous searches in NICE, HTW, SIGN or Canada's Drug Agency; instead, all records were retrieved with a focus on those from 2024 onwards, with those from September 2024 onwards reviewed if a month was clearly reported. A more comprehensive strategy based on Medical Subject Heading (MeSH) and free-text terms for colonoscopy was used for INAHTA, which is presented in Supplementary Tables 15 and 16.

Supplementary Table 15. Search strategy for INAHTA database – clinical SLR – 15/09/24

#	Searches	Results (15/09/24)
1	"Colonoscopy"[mh]	46
2	"Sigmoidoscopy"[mh]	9
3	"Proctoscopy"[mh]	1
4	"Endoscopy, Gastrointestinal"[mh]	60
5	(colonoscop* or polypect* or sigmoidoscop* or proctoscop* or coloscop* or ileocolonoscop* or anoscop* or rectoscop* or proctosigmoidoscop* or endoscop*)	440
6	((colonoscop* or polypect* or sigmoidoscop* or proctoscop* or coloscop* or ileocolonoscop* or anoscop* or rectoscop* or proctosigmoidoscop* or endoscop*)) OR ("Endoscopy, Gastrointestinal"[mh]) OR ("Proctoscopy"[mh]) OR ("Sigmoidoscopy"[mh]) OR ("Colonoscopy"[mh])	452

Abbreviations: INAHTA, International Network of Agencies for Health Technology Assessment; SLR, systematic literature review.

Supplementary Table 16. Search strategy for INAHTA database – clinical SLR – 12/06/25 update

#	Searches	Results (12/06/25)
1	"Colonoscopy"[mh]	46
2	"Sigmoidoscopy"[mh]	9
3	"Proctoscopy"[mh]	1
4	"Endoscopy, Gastrointestinal"[mh]	62
5	(colonoscop* or polypect* or sigmoidoscop* or proctoscop* or coloscop* or ileocolonoscop* or anoscop* or rectoscop* or proctosigmoidoscop* or endoscop*)	441
6	((colonoscop* or polypect* or sigmoidoscop* or proctoscop* or coloscop* or ileocolonoscop* or anoscop* or rectoscop* or proctosigmoidoscop* or endoscop*)) OR ("Endoscopy, Gastrointestinal"[mh]) OR ("Proctoscopy"[mh]) OR ("Sigmoidoscopy"[mh]) OR ("Colonoscopy"[mh])	454

The 454 records above were filtered by year to obtain 21 records between 2024 and 2025. It was not possible to refine results further by exact date to obtain only those that were new since the previous search was run.

Abbreviations: INAHTA, International Network of Agencies for Health Technology Assessment; SLR, systematic literature review.

1.2 Inclusion and exclusion

Supplementary Table 17. Inclusion and exclusion criteria of the clinical SLR

Factor	Inclusion	Exclusion	Comment
Population	Any human population undergoing colonoscopy.	Animal studies and human populations not undergoing colonoscopy were excluded.	NA
Interventions	AI systems of interest:<br>• Argus® (Endosoft);<br>• CAD EYE® (Fujifilm Healthcare UK Ltd.);<br>• CADDIE™ (Odin Vision);<br>• Discovery™ (Pentax Medical UK);<br>• ENDO-AID™ (Olympus Medical Systems Corp.);<br>• ENDOANGEL® Lower Gastrointestinal Endoscope Image Auxiliary Diagnostic Equipment (Wuhan ENDOANGEL Medical Technology Co. Ltd.);<br>• Endoscopic Multimedia Information System (EMIS™; EndoPerv LLC., previously EndoMetric Corporation);<br>• EndoScreener® (WISION AI);<br>• GI Genius™ (Medtronic);<br>• MAGENTIQ-COLO™ (MAGENTIQ-EYE);<br>• WISE VISION® (NEC Corporation).<br><br>They could be used with or without the use of VCE, dye-based chromoendoscopy or ENDOCUFF VISION™.	Any alternative AI-supported colonoscopy systems not listed in the NICE final scope were excluded.<br><br>Evidence for the AI system used as an adjunct to endoscopist judgement was prioritised; if evidence for key outcomes from at least one study per intervention and population was identified, studies reporting results for AI when used alone (autonomous AI) were excluded, as adjunct use aligns with how the AI systems are expected to be used in clinical practice.<br><br>When a diagnostic accuracy study compared adjunct AI use with an autonomous AI group, only the adjunct AI group was included in the report.	In February 2025, WISE VISION® was removed from the SLR given the authors were notified that it no longer met the requirements of the NICE scope as it had been withdrawn from the UK market.<br><br>Only AI systems with publicly available data are covered in this publication, meaning data for CADDIE™ and EMIS™ are not covered.<br><br>For brevity, only trials using the AI systems adjunctly have been included in this publication and associated supplementary materials.
Comparators	Colonoscopy performed without the use of AI-supported colonoscopy systems (i.e. standard colonoscopy).	Any other comparator.	NA

	This could be with or without the use of VCE, dye-based chromoendoscopy or ENDOCUFF VISION™.<br><br>Diagnostic accuracy studies did not necessarily need to have a relevant comparator arm to be included.		
Study design	RCTs or non-randomised studies permitted Single-arm studies considered for studies reporting diagnostic accuracy data.<br><br>Studies must have applied the AI system to colonoscopies in real-time rather than applying to videos or photographs of colonoscopies that had previously occurred as this best reflects the way in which they will be used in clinical practice.<br><br>For studies where the focus was not on diagnostic accuracy data (e.g. they focused on outcomes such as ADR or APC rather than reporting sensitivity or specificity data), non-randomised studies were only included if there were no RCTs for that particular intervention and population for key outcomes.<br><br>Conference abstracts were only considered for inclusion where information was not available from any full text publications for an intervention, population or key outcome.	While SLRs and meta-analyses were included up until full text screening to allow reference lists to be searched for relevant primary studies, the reviews themselves were excluded after this had taken place.<br><br>Studies applying AI systems to videos or photographs rather than live colonoscopies were excluded (i.e. *ex vivo* rather than *in vivo*).	NA
Reference standards (diagnostic accuracy studies only)	For diagnostic accuracy studies, the most relevant reference standards were considered to be:<br>• CADe (polyp detection) – standard colonoscopy;<br>• CADx (polyp characterisation) – histology.	NA	NA

	Other reference standards could be considered where available and if data using the preferred reference standard was scarce.		
Outcomes	Outcomes of interest were:<br>• Measures of ability or accuracy to detect polyps or cancer;<br>• Measures of ability to characterise identified polyps;<br>• Measures related to healthcare resource use (such as time to do a colonoscopy, need for repeat colonoscopy to be done, need for a second observer);<br>• Time to colonoscopy and impact on waiting lists;<br>• Number of polyp removal procedures;<br>• Incidences that the technology does not function;<br>• Impact on decision making;<br>• Ease of use/acceptability of the technologies to healthcare professionals;<br>• Morbidity (including outcomes related to the colonoscopy procedure and cancer, such as incidence of post-colonoscopy CRC);<br>• Mortality;<br>• Health-related quality of life (including anxiety);<br>• Acceptability of tests to patients.	Outcomes not falling into any of the categories listed in the previous column.	For brevity, this publication focuses on the following specific outcomes as these were either included in the associated health economic model (a separate publication covering the economic modelling associated with this research is anticipated) or were otherwise reported for multiple AI systems and considered to be key for assessing the impact of the AI systems on colonoscopy procedures:<br>▪ ADR (overall, advanced and non-advanced, and for different size categories);<br>▪ APC;<br>▪ AMR;<br>▪ Neoplastic/hyperplastic polyp DR;<br>▪ SSL DR and significant polyp DR;<br>▪ Diagnostic accuracy of the CADx functionality for relevant AI systems in all polyps, all diminutive polyps and diminutive polyps separated into rectosigmoid and non-rectosigmoid, or proximal and distal, categories;<br>▪ Procedural duration outcomes (withdrawal time and total procedure time);<br>▪ Adverse events.

Other	No limits on language were applied. Google Translate was considered where required to facilitate understanding of the paper to assess inclusion.	NA	NA

Abbreviations: ADR, adenoma detection rate; AI, artificial intelligence; AMR, adenoma miss rate; APC, adenomas per colonoscopy; CADe, computer-aided polyp detection; CADx, computer-aided polyp characterisation; CRC, colorectal cancer; DR, detection rate; NA, not applicable; NICE, National Institute for Health and Care Excellence; RCT, randomised controlled trial; SLR, systematic literature review; SSL, sessile serrated lesion; VCE, virtual chromoendoscopy.

1.3 Synthesis and statistical analysis

1.3.1 Further details on synthesis methods

Data have been analysed separately for each intervention with no pooling of data for different interventions. For analyses of non-diagnostic accuracy data, such as data from randomised controlled trials (RCTs) comparing dichotomous (e.g. adenoma detection rate [ADR]) or continuous (e.g. adenomas per colonoscopy [APC]) outcomes, meta-analyses have been performed for each intervention in Review Manager.[3] On review of the included studies, a high likelihood of clinical and methodological heterogeneity within the trials was noted (for example, based on differences in populations included, adjunct technologies used and endoscopist experience or expertise). Therefore, random effects models have been preferred for the analyses.

Furthermore, the main analyses for these outcomes exclude studies considered to be at high risk of bias unless no other studies were available for a particular intervention OR the study at high risk of bias covered a population that was not well covered in studies at a lower risk of bias (such as patients with inflammatory bowel disease [IBD]). This approach of excluding studies at a higher risk of bias is in line with guidance in Section 7.6.2 of the Cochrane Handbook for Systematic Reviews of Interventions version 6.5.[4] This led to the exclusion of six RCTs from the main analyses across interventions, but sensitivity analyses with these studies included were performed.

Dichotomous outcomes have been presented as risk ratios (RRs), unless event rates in the whole study were <1.0%, in which case Peto odds ratios have been used. For adverse events (AEs), it was often the case that zero events in both study arms were reported; Forest plots for AEs have been presented using risk difference given estimates are not calculable for RRs or Peto odds ratios (ORs) for studies with zero events in both arms. Outcomes reported as continuous measures, for example means with standard deviations (SD) for each trial arm have primarily been analysed as a continuous outcome, with mean differences used as the effect measure. For APC, an additional analysis for each intervention was performed, with it analysed as an incidence rate ratio (IRR). This was performed as it was identified as a suitable input for a scenario in the economic model (the economic modelling is not covered in this publication but a separate publication discussing this is anticipated). Calculation of the IRR requires the total number of adenomas across all colonoscopies to be divided by the total number of

colonoscopies in each arm. This information was used to calculate rate ratios using the MedCalc tool.[5] Where these data were not available, either an unadjusted IRR reported in the paper was used in the meta-analysis, or the total number of adenomas were estimated by multiplying the mean per colonoscopy value in each treatment arm by the number of colonoscopies, which was then used to calculate an IRR. Due to time constraints, this additional analysis was only performed for the APC outcome and not for other similar outcomes such as polyps per colonoscopy or sessile serrated lesions per colonoscopy.

Some studies had multiple arms that met the criteria for inclusion under intervention (AI-supported colonoscopy) and/or comparator (standard colonoscopy) colonoscopies outlined in the NICE final scope.[6] For example, one study included two CAD EYE® groups and two standard colonoscopy groups (one with and one without the use of ENDOCUFF VISION™) and another study covering ENDO-AID™ was similar.[7, 8] In these cases, outcome data from the separate arms were combined into a single computer-aided polyp detection (CADe) arm and a single standard colonoscopy arm, as the use of ENDOCUFF VISION™ is possible within UK clinical practice, although it may be variable). This was done by totalling events and number analysed for dichotomous outcomes and through use of an online calculator that is based on formulae and methods reported in Sections 6.2.9, 6.5.2.10 and 23.3.4 of the Cochrane Handbook for Systematic Reviews of Interventions for continuous outcomes.[4, 9]

Raw data from RCTs have been included in meta-analyses rather than adjusted or unadjusted effect estimate data where reported, given there were no major concerns about using unadjusted data from RCTs and it was more commonly reported than adjusted data. Where raw data were not reported but effect estimates such as RR with 95% confidence intervals (CIs) were, the generic inverse variance method was used to ensure all studies could be included in meta-analyses of the same outcome for each intervention. For tandem studies reporting outcomes such as ADR or APC, data for the first randomised intervention only was included in the meta-analyses as this better aligns with the parallel non-tandem studies, and also aligns with the approach in the HTW report.[1]

The main analyses were performed within the whole/mixed colonoscopy population regardless of endoscopist experience; as prespecified in the final protocol, subgroup analyses based on colonoscopy indication population and endoscopist experience and expertise were performed.[10] For colonoscopy indication, the approach was to report any within-trial subgroup analyses however they had been reported in the trial, as well as to separate whole studies into specific subgroups based on which indication most patients within each trial were categorised

under. Additional analyses where whole studies categorised based on the majority were combined with within-trial subgroup data for studies that reported it were also explored, as were variations of these analyses where only studies with >80% of participants falling into a specific category were included. Categories included were loosely based on those included in Table 1 of the NICE final scope but had to be adapted in some cases given studies often separated populations in a different way.[6]

For endoscopist experience and expertise subgroup analyses, various ways that individual studies defined experience or expertise were reviewed and potential strategies for exploring this via subgroup analyses were proposed. These were then reviewed by specialist committee members involved in the NICE appraisal and any feedback was used to add to or alter the way in which they were explored. The following approaches were considered for each intervention, although it should be noted that many were not feasible for many interventions given the limited number of studies or the limited variation in terms of colonoscopy indications covered within a specific intervention:

- Any analyses performed within individual trials regarding expertise or experience to be reviewed;
- Comparison between colonoscopies performed by screening-accredited endoscopists and those without this accreditation (if reported by studies);
- Separation of studies including only non-trainees, only trainees or a mix of trainees and non-trainees (as defined in the study);
- Separation into expert/experienced and non-expert/less experienced based on the definitions used within each trial (for example, some studies may define experienced endoscopists as those with >2,000 colonoscopies while others might have a lower threshold or use a different factor to define experience);
- Separation of studies based on baseline ADR of the endoscopists participating – a threshold of 40 to 50% or 45% was suggested by two specialist committee members as useful for separating between Bowel Cancer Screening Programme (BCSP)-level endoscopists and non-BCSP endoscopists.

A similar approach to using whole studies and within-trial subgroup data, as well as exploring whole studies categorised based on the majority and where >80% were within a certain subgroup, was followed where data allowed.

In reality, subgroup analyses for population and endoscopist experience/expertise were difficult to construct and to interpret; specialist committee members themselves noted that subgroup analyses for endoscopist experience were likely to be difficult given the variation between studies and it was rarely possible to separate studies in the most clinically useful way, which may be based on a baseline ADR threshold of 40% before study enrolment, given this is likely to separate screening and non-screening endoscopists. The wide variation in methods to define experience or expertise, and the different ways of separating populations for colonoscopy indication subgroup analyses, coupled with the fact that there was often only one or two studies within each category means these analyses are considered to be exploratory. ADR and APC were prioritised for subgroup analyses as these were usually the most commonly reported outcomes across interventions.

Sensitivity analyses for specific meta-analyses were performed were deemed necessary, for example where data from two studies of a trial had been combined into a single arm this was explored and where there was a concern about a study given its comparator was slightly different to other studies. Sensitivity analyses including studies at high risk of bias were also explored, as the default was to exclude them. Due to time constraints, sensitivity analyses were explored for ADR and APC outcomes only.

No meta-analyses of diagnostic accuracy data were performed. Diagnostic accuracy data were scarcely reported for CADe but for computer-aided polyp characterisation (CADx) there were some studies reporting data, particularly for CAD EYE® and GI Genius™ systems. However, on review of the studies in terms of population, outcomes reported, analysis methods and use of the AI system (i.e. adjunct or autonomous), the overlap was considered to be too limited; there are large concerns about heterogeneity and the meta-analysis of these data is not considered to be robust or meaningful at this stage. Instead, the CADx diagnostic accuracy data from each study have been presented separately. The following observations contributed to the concerns about heterogeneity:

- Population included – some included anyone undergoing colonoscopy while others required the presence of at least one polyp of a specific type (e.g. at least one diminutive rectosigmoid polyp);
- Analysis methods – some included high- and low-confidence diagnoses in the analysis while others only included high-confidence diagnoses, and sessile serrated lesions (SSLs) were treated differently in different analyses (i.e. adenomatous in some, non-adenomatous in others, or excluded completely);

- Overlap of outcomes within interventions was limited – in most cases only two studies for the same intervention and outcome were available, with the only category with more than this being an analysis where AI is used autonomously rather than as an adjunct, which does not align with the expected use of the AI system in clinical practice.

1.4 Search results and included studies

1.4.1 PRISMA flow diagram

Supplementary Figure 3. PRISMA flow diagram of records included in the clinical systematic literature review

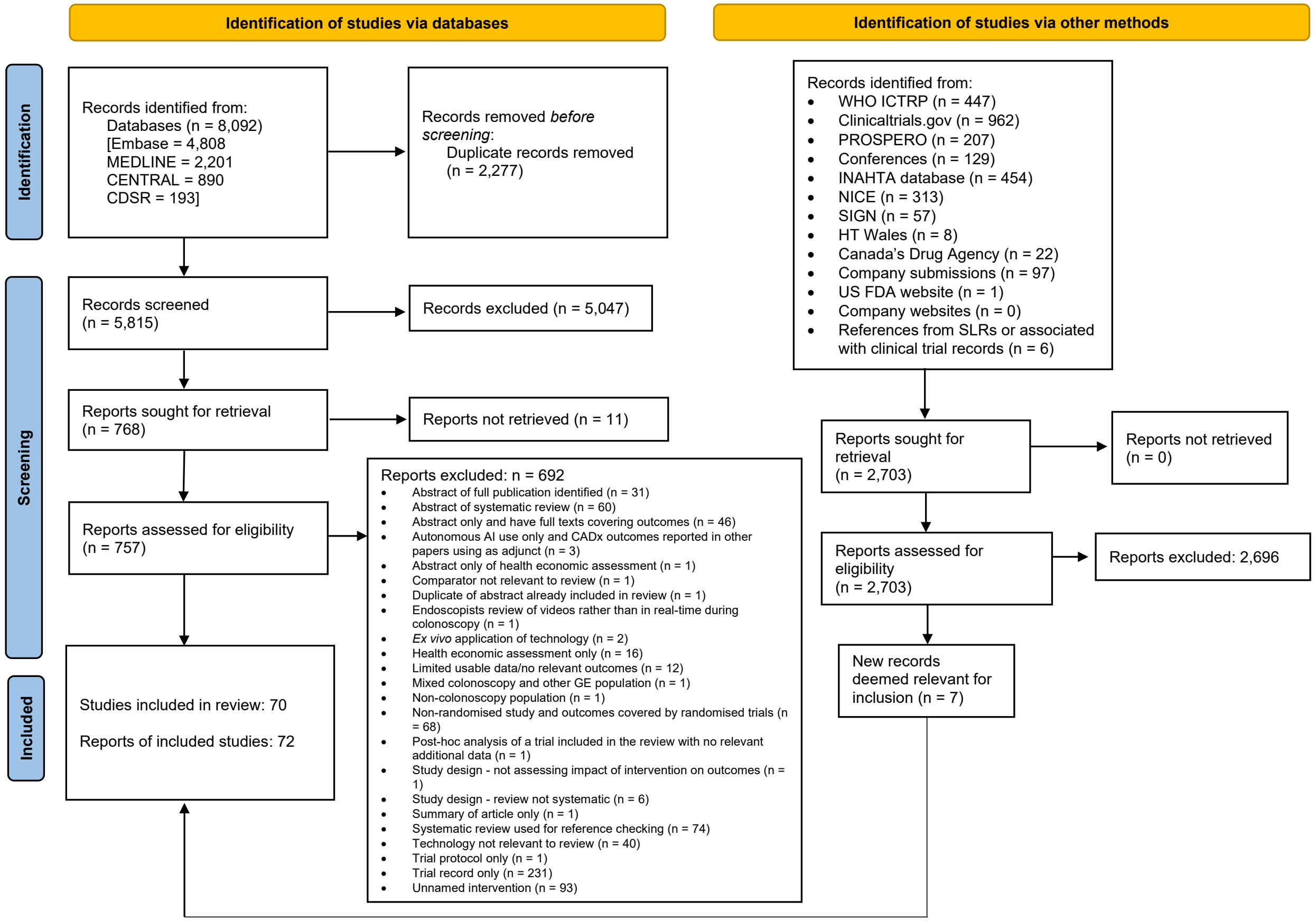

Note: this PRISMA diagram is reflective of the full clinical evidence base covered in the report produced for the NICE appraisal;[11] this publication does not cover all outcomes and Ai systems included in the full NICE appraisal, and only studies using AI systems as an adjunct to endoscopist judgement have been included in this publication, with information in this publication obtained from 52 of the 70 included studies outlined in the PRISMA.
Abbreviations: AI, artificial intelligence; CADx, computer-aided characterisation; CDSR, Cochrane Database of Systematic Reviews; CENTRAL, Cochrane Central Register of Controlled Trials; FDA, US Food and Drug Administration; GE, gastroenterology; HT Wales, Health Technology Wales; INAHTA, International Network of Agencies for Health Technology Assessment; NICE, National Institute for Health and Care Excellence; PRISMA, Preferred Reporting Items for Systematic reviews and Meta-Analyses; SIGN, Scottish Intercollegiate Guidelines Network; SLR, systematic literature review; WHO ICTRP, World Health Organization International Clinical Trials Registry Platform.

1.4.2 Studies included in this publication

Supplementary Table 18. Summary of studies prioritised for inclusion in the clinical review (for data covered in this publication)

Study (country, sites) – trial name	Population	Intervention*	Comparator*	Endoscopist experience	Study design	Reference standard (if applicable)
**Argus® (Endosoft)**						
**CADe studies**						
Strapko 2023[12, 13] (USA, single site)	Adults undergoing screening, surveillance or diagnostic colonoscopy	Argus®-assisted colonoscopy (n=344)	Standard colonoscopy without Argus® (n=342)	Not reported	Parallel RCT<br>Abstract + limited information from instructions for use manual only	NA
**CADx studies**						
None – not described as a function of Argus®						
**CAD EYE® (Fujifilm Healthcare UK Ltd.)**						
**CADe studies**						
Aniwan 2023[7] (Thailand, single site)	Asymptomatic adults 50-75 years undergoing routine	CAD EYE®-assisted	Standard HD colonoscopy using white light (n=625)†	7 staff attendings, 10 trainees. Average baseline ADR 33%.	Parallel RCT	NA

	screening colonoscopy or following positive FIT	colonoscopy (n=620)†				
Desai 2024[14] (USA, 12 sites)	≥45 years undergoing screening or surveillance colonoscopy for history of polyps (surveillance interval ≥3 years)	CAD EYE®-assisted colonoscopy (n=509)	Standard HD colonoscopy (n=522)	≥1000 colonoscopies, baseline ADR 25 to 40%	Parallel RCT	Histology (for some DA measures of CADe function)
Djinbachian 2024[15] (Canada, single site)	45 to 80 years undergoing elective colonoscopy for screening, surveillance or diagnosis purposes	CAD EYE®-assisted colonoscopy with water exchange and caecal retroflexion (n=229)	Standard colonoscopy with no CAD EYE®, water exchange or caecal retroflexion (n=238)	Board-certified gastroenterologists (n=4) or trainees (n=1)	Parallel RCT	NA
Hiratsuka 2025[16] (Japan, single site)	≥20 years scheduled for lower gastrointestinal endoscopy (screening, symptomatic and surveillance colonoscopies)	CAD EYE®-assisted colonoscopy (n=48)	Standard colonoscopy (n=46)	Expert (≥10 years' experience) and non-expert (<10 years' experience) endoscopists included. ~40% procedures performed by experts.	Tandem RCT	NA
Huneburg 2023[17] (Germany, single site) – CADLY trial	≥18 years with LS and MLH1, MSH2 or MSH6 pathogenic germline variant with 10-36 months since last colonoscopy	CAD EYE®-assisted colonoscopy (n=50)	Standard HD white-light colonoscopy (n=46)	Experienced in LS endoscopic surveillance (>1000 total colonoscopies, >300 in LS patients)	Parallel RCT	NA

Miyaguchi 2024[18] (Japan, single site)	≥20 years undergoing colonoscopy due to positive FIT, abdominal symptoms or for follow-up of colon polyps	CAD EYE®-assisted colonoscopy (n=400)	Standard HD colonoscopy with white-light imaging and LCI (n=400)	Experts and trainees, experts defined as >1000 colonoscopies and trainees as <1000 colonoscopies	Parallel RCT	NA
Nakashima 2023[19] (Japan, single site)	21 to 81 years undergoing primary endoscopic screening for CRC, following a positive FIT of occult blood or patients with colorectal neoplasia undergoing endoscopic resection	CAD EYE®-assisted colonoscopy (n=207)	Standard HD colonoscopy with white-light imaging (n=208)	Experienced. Board-certified trainers of the Japan Gastroenterological Endoscopy Society or board certified fellow of the Japan Gastroenterological Society	Tandem RCT	NA
Rondonotti 2022[20] (Italy, 5 sites) - AIFIT trial	50 to 74 years undergoing colonoscopy as part of CRC screening programme following positive FIT	CAD EYE®-assisted colonoscopy (n=405)	Standard HD white-light colonoscopy (n=395)	Qualified to work in FIT-based screening programme (≥300 colonoscopies per year, caecal intubation rate ≥95%, ADR ≥25%)	Parallel RCT	NA
Scholer 2024[21] (Sweden, 2 sites)	40 to 90 years undergoing colonoscopy for cancer screening, alarm symptoms or other reasons such as positive faecal occult stool test, polyp surveillance,	CAD EYE®-assisted colonoscopy (n=98)	Standard HD white-light or LCI colonoscopy (n=95)	Experienced (≥400 prior colonoscopies) and inexperienced (<400 prior colonoscopies) endoscopists included	Parallel RCT‡	NA

	hereditary CRC and diarrhoea					
Tiankanon 2024[22] (Thailand, 3 sites)	Asymptomatic, 50 to 75 years undergoing routine screening colonoscopy or screening following a positive FIT	CAD EYE®-assisted colonoscopy (n=400)	Standard HD white-light colonoscopy (n=400)	Baseline ADR ≥35% (from ≥100 prior screening colonoscopies in mixed population of primary colonoscopies and following positive FIT). Average baseline ADR was 42.6%. Includes attending physicians and fellows under supervision	Parallel RCT	NA
Yamaguchi 2024[23] (Japan, 3 sites)	≥20 years scheduled for colonoscopy following positive FIT or for surveillance following colonic polypectomy	CAD EYE®-assisted colonoscopy (n=113)	Standard colonoscopy (n=118)	Performed by trainees (third/fourth year physician with up to 20 prior colonoscopies) back-to-back an expert (>5000 colonoscopies), who performed resections and could assist with insertion and performed observation separately to the trainee.	Parallel RCT with tandem procedures performed by experts	NA
Zimmermann-Fraedrich 2025[24] (Germany, 12 sites)	≥50 years undergoing screening colonoscopy (age cut-offs 50 years for men and 55 years for women) or diagnosis colonoscopy	CAD EYE®-assisted colonoscopy (n=812)	Standard colonoscopy (n=815)	Experienced examiners (not defined)	Parallel RCT	NA

	(including polyp follow-up and symptom evaluation)					
**CADx studies**						
Djinbachian 2024[25] (Canada, single site)	45 to 80 years undergoing colonoscopy (screening, surveillance or diagnostic)	CAD-EYE®-assisted optical diagnosis (n=179 polyps)	NA – autonomous AI assessment reported in paper but not extracted given adjunct AI assessment prioritised from this paper	Between 1 and >30 years' experience with optical diagnosis. Procedural volume 300 and 1500 colonoscopies per year. All participated in previous optical diagnosis-based studies.	Parallel RCT	Histology
Rondonotti 2023[26] (Italy, 4 sites) – ABC trial	18 to 85 years undergoing outpatient colonoscopy (symptoms, surveillance, FIT positive and primary screening) with at least one DRSP detected	CAD-EYE®-assisted optical diagnosis (n=550 DRSP)	Endoscopist optical diagnosis alone (n=540 DRSP)	Experts and non-experts included. Experts had undertaken specific training programme, had auditing and monitoring and performed optical diagnosis on regular basis according to ESGE curriculum.	Prospective non-randomised	Histology
Sato 2024[27] (Japan, 3 sites)	20 to 85 years scheduled to undergo colonoscopy following positive FIT, for symptoms, screening or where endoscopist	CAD-EYE®-assisted optical diagnosis (n=380 lesions)	Endoscopist optical diagnosis alone (n=380 lesions)	Experts (≥1500 colonoscopies) and non-experts (<1500 colonoscopies)	Prospective non-randomised	Histology

	otherwise deemed a colonoscopy necessary					
Taghiakbari 2025[28] (Canada, single site)	45 to 80 years undergoing outpatient colonoscopy (indications not reported)	CAD-EYE®-assisted optical diagnosis (n=138 diminutive polyps resected and discarded or diagnosed and left)	NA – no comparator assessment reported	Academic endoscopists with training and experience in CADx-assisted and CADx-unassisted optical diagnosis	Prospective non-randomised	Expert video review – polyps resected and discarded or diagnosed and left in place
**Studies reporting CADe and CADx data**						
Cassinotti 2023[29] (Italy, single site)<br><br>No CADe data for outcomes covered in this publication, unclear if CADx use autonomous or adjunct	Patients with UC undergoing endoscopic surveillance	CAD EYE® + LCI/BLI (n=62; 113 lesions)	WLE and LCI as separate comparators<br>(n=62; 113 lesions)[§]	Not reported	Non-randomised, prospective tandem study<br>Abstract only	Histology for CADx function
Alali 2025[30] (Kuwait, single site)<br><br>CADx data not included as likely autonomous use	≥45 years undergoing average-risk screening or surveillance colonoscopy	Detection: CAD-EYE®-assisted colonoscopy (n=51)<br><br>Characterisation: CAD-EYE® use, unclear if adjunct to endoscopist judgement or autonomous (n=69 polyps)	Detection: Standard HDWL colonoscopy (n=51)<br><br>Characterisation: standard HDWL colonoscopy with chromoendoscopy (n=52 polyps)	Experienced endoscopists (≥1000 colonoscopies)	Parallel RCT	Histology for CADx function
**Discovery™ (Pentax Medical UK)**						

**CADe studies**						
Maas 2024 - Discovery™[31] (Canada, France, Germany, Italy, Netherlands, Russia – 7 sites) – DISCOVERY II trial	≥18 years scheduled for non-iFOBT screening, surveillance or diagnosis colonoscopy	Discovery™-assisted colonoscopy (n=250)	Standard HD colonoscopy (n=247)	>2000 colonoscopies (500 was requirement)	Parallel RCT	NA
**CADx studies**						
Lopez-Serrano 2024[32] (Spain, single site) – CUDISIA trial	≥18 years at risk of CRC undergoing surveillance colonoscopy for ulcerative colitis	Discovery™-assisted optical diagnosis (n=61 lesions)	VCE with iSCAN assessment optical diagnosis (n=61 lesions)	Endoscopists with extensive experience in DCE and VCE	Prospective non-randomised	Histology
**ENDO-AID™ (Olympus Medical Systems Corp.)**						
**CADe studies**						
Gimeno-Garcia 2023[33] (Tenerife, single site)	≥18 years, including patients with colonoscopy for average-risk population screening, post-polypectomy surveillance, rectal bleeding, anaemia, familial CRC screening, change in bowel habits, chronic diarrhoea and suspicion of CRC	ENDO-AID™-assisted colonoscopy (n=185)	Standard HD colonoscopy (n=185)	High and low detectors included (ADR ≥40% and <40%, respectively), with >2000 lifetime colonoscopies	Parallel RCT	NA

Lau 2024[34] (Hong Kong, single site) – ENDOAIDTRAIN trial	≥18 years undergoing elective colonoscopy for screening, surveillance or diagnostic purposes (i.e. symptoms)	ENDO-AID™-assisted colonoscopy (n=386)	Standard HD white-light colonoscopy (n=380)	Trainees (<500 procedures and <3 years' experience) with supervisors present on-site or next-door supervision (supervisors could alert trainees to missed polyps and assist with caecal intubation and/or resection of polyps)	Parallel RCT	NA
Lui 2024[8] (Hong Kong, 2 sites)	≥40 years undergoing elective colonoscopy for screening, surveillance or diagnostic workup	ENDO-AID™-assisted colonoscopy with or without ENDOCUFF VISION™ (n=468)[ǁ]	Standard HD white-light colonoscopy (n=214)	Range from 1 to 23 years' experience, historical ADR range 30 to 53%	Parallel RCT	NA
Spada 2025[35] (Italy, 2 sites) – ACCENDO-Colo trial	40 to 85 years undergoing screening (opportunistic or immunological FOBT-based) or surveillance colonoscopy	ENDO-AID™-assisted colonoscopy (n=611)	Standard HD colonoscopy (n= 617)	Experienced endoscopists (>2000 examinations)	Parallel RCT	NA
Vilkoite 2023[36] (Latvia, single site)	≥18 years referred for colonoscopy by the family doctor	ENDO-AID™-assisted colonoscopy (n=194)	Standard colonoscopy with NBI (n=206)	Average 2000 colonoscopy examinations per year between two endoscopists; 8- and 15-years' experience	Parallel RCT[‡]	NA
**CADx studies**						
None – not described as a function of ENDO-AID™						

ENDOANGEL® Lower Gastrointestinal Endoscope Image Auxiliary Diagnostic Equipment (Wuhan ENDOANGEL Medical Technology Co. Ltd.)						
**CADe studies**						
Gong 2020[37] (China, single site)	18 to 75 years undergoing colonoscopy (for screening, clinical symptoms or surveillance)	ENDOANGEL® -assisted colonoscopy (n=355)	Standard colonoscopy (n=349)	Endoscopy experience of 1 to 3 years with total colonoscopies 1500 to 4000	Parallel RCT‡	NA
Yao 2022[38] (China, single site)	≥18 years undergoing colonoscopy for screening, post-polypectomy surveillance or gastrointestinal symptoms	ENDOANGEL® -assisted colonoscopy (n=268)	Standard HD colonoscopy (n=271)	>2000 screening colonoscopies required	Parallel RCT	NA
Yao 2024[39] (China, 3 sites)	>18 years undergoing diagnostic, screening or surveillance colonoscopy	ENDOANGEL® -assisted colonoscopy (n=227)¶	Standard HD white-light colonoscopy (n=229)¶	Novices (>1 year gastroenterology fellowship experience and no prior experience or training in colonoscopy) performed withdrawal phase (experts performed insertion; ≥5000 colonoscopies)	Tandem RCT	NA
Zhang 2023[40] (China, 3 sites)	18 to 75 years undergoing colonoscopy for diagnosis or screening	ENDOANGEL® -assisted colonoscopy (n=643)	Standard colonoscopy (n=650)	At least 1 year experience and total volume of 100 colonoscopies	Parallel RCT‡	NA

**CADx studies**						
None – not described as a function of ENDOANGEL®						
**EndoScreener® (WISION AI)**						
**CADe studies**						
Glissen Brown 2022[41] (USA, 4 sites)	≥22 years undergoing colonoscopy for CRC screening or surveillance	EndoScreener® - assisted colonoscopy (n=113)	Standard HD white-light colonoscopy (n=110)	Experienced endoscopists with high baseline ADR (ADR in HDWL-first group was 44.0%)	Tandem RCT	NA
Liu 2020[42] (China, single site)	14 to 90 years undergoing colonoscopy for any indication	EndoScreener® - assisted colonoscopy (n=393)	Standard HD colonoscopy (n=397)	Senior, mid-level and junior endoscopists included	Parallel RCT	NA
Wang 2019[43] (China, single site)	Symptomatic or screening colonoscopies, mean age 50 years	EndoScreener® - assisted colonoscopy (n=522)	Standard HD colonoscopy (n=536)	Senior, mid-level and junior endoscopists included, defined based on number of prior colonoscopies (10,000+, 3000 to 10,000 and 100 to 500, respectively)	Parallel RCT	NA
Wang 2020 (effect of a deep…)[44] (China, single site) - CADe-DB trial	18 to 75 years undergoing diagnostic or screening colonoscopies	EndoScreener® - assisted colonoscopy (n=484)	Standard white-light HD colonoscopy with sham CADe system (n=478)	Senior endoscopists with at least 5 years' experience and at least 1000 colonoscopies per year	Parallel RCT	NA
Wang 2020 (lower adenoma miss…)[45] (China, single site)	18 to 75 years referred for diagnostic, screening or surveillance	EndoScreener® - assisted colonoscopy (n=184)	Standard white-light HD colonoscopy (n=185)	Experienced endoscopists from division of gastroenterology	Tandem RCT	NA

	colonoscopy (prior polypectomy)					
Wang 2023[46] (China, 4 sites)	18 to 75 years undergoing symptomatic, screening or surveillance colonoscopy	EndoScreener® - assisted colonoscopy (n=636)	Standard white-light HD colonoscopy with observer assistance (n=625)	Endoscopists with >2000 colonoscopy screening performed procedures. In observer group, trainees were observers (100 to 500 procedures, qualified in colonoscopy)	Parallel RCT	NA
**CADx studies**						
None – not described as a function of EndoScreener®						
**GI Genius™ (Medtronic)**						
**CADe studies**						
Ahmad 2023[47] (UK, single site) – AI-DETECT trial	60 to 74 years with positive FIT test within NHS BCSP, established history of adenomas attending for surveillance colonoscopy within BCSP or >55 years referred for colonoscopy due to large/multiple adenomas during screening flexible sigmoidoscopy	GI Genius™ - assisted colonoscopy (n=308)	Standard HD colonoscopy (n=306)	Endoscopists working at an NHS bowel cancer screening centre. Between 46 and 109 colonoscopies with ADR between 56 and 80%	Parallel RCT	NA

Engelke 2023[48] (Sweden, single site)	≥18 years having colonoscopy for primary screening, post-polypectomy surveillance, tumour follow-up or work-up for GI symptoms such as bleeding, anaemia, IBD (diagnostic colonoscopy)	GI Genius™ - assisted colonoscopy (n=122)	Standard HD colonoscopy (n=110)	Trained endoscopists (no further details)	Parallel RCT‡	NA
Karsenti 2023[49] (France, single site) – COLO-Genius trial	≥18 years undergoing total colonoscopy	GI Genius™ - assisted colonoscopy (n=1003)	Standard colonoscopy (n=1012)	Endoscopists with >2000 prior colonoscopies	Parallel RCT	NA
Lagstrom 2025[50] (Denmark, 4 sites)	≥18 years undergoing screening following a positive FIT (>100 µg/l), surveillance or diagnostic colonoscopy	GI Genius™ - assisted colonoscopy (n=400)	Standard colonoscopy (n=395)	Experts (>1000 colonoscopies) and non-experts (≤1000 colonoscopies) included	Parallel RCT‡	NA
Levartovsky 2023[51] (Israel, single site)	Colonoscopies in patients with IBD (no further information)	Colonoscopies performed after incorporation of GI Genius™ (n=759)	Colonoscopies performed prior to incorporation of GI Genius™ (n=237)	Conducted at high-volume gastroenterology department	Retrospective Abstract only	NA
Mangas-Sanjuan 2023[52] (Spain, 6 sites) – CADILLAC trial	≥18 years presenting for colonoscopy after positive FIT or for CRC screening	GI Genius™ - assisted colonoscopy (n=1610)	Standard HD colonoscopy (n=1603)	Endoscopist experience unclear, but all are screening colonoscopies	Parallel RCT	NA
Ortiz 2024[53] (Belgium, Germany, Italy, Spain – 17 sites) – TIMELY trial	≥18 years undergoing surveillance	GI Genius™ - assisted	Standard white-light HD colonoscopy (n=216)	ADR ≥20% for screening colonoscopy and ≥35% following	Parallel RCT	NA

	colonoscopy for LS (germline variant in MLH1, MSH2, MSH6 or EPCAM)	colonoscopy (n=214)		positive FIT required. Also required >2000 colonoscopies, and training in optical diagnosis and chromoendoscopy techniques		
Repici 2020[54] (Italy, 3 sites) – AID trial	40 to 80 years undergoing colonoscopy for primary CRC screening, post-polypectomy surveillance or following a positive FIT	GI Genius™ - assisted colonoscopy (n=341)	Standard HD colonoscopy (n=344)	>2000 screening colonoscopies required. Centres involved in organised CRC screening programme	Parallel RCT	NA
Repici 2022[55] (Italy, Switzerland – 5 sites) – AID2 trial	40 to 80 years undergoing colonoscopy for primary screening (outside regional screening programme), following positive FIT within screening programme, post-polypectomy surveillance and diagnostic colonoscopy for signs/symptoms	GI Genius™ - assisted colonoscopy (n=330)	Standard HD colonoscopy (n=330)	Non-expert (<2000 colonoscopies lifetime)	Parallel RCT	NA
Scholer 2024[21] (Sweden, 2 sites)	40 to 90 years undergoing colonoscopy for cancer screening,	GI Genius™ - assisted colonoscopy (n=24)	Standard HD white-light or LCI colonoscopy (n=23)	Experienced (≥400 prior colonoscopies) and inexperienced (<400 prior	Parallel RCT‡	NA

	alarm symptoms or other reasons such as positive faecal occult stool test, polyp surveillance, hereditary CRC and diarrhoea			colonoscopies) endoscopists included		
Seager 2024[56] (UK, 12 sites) – COLO-DETECT trial	≥18 years undergoing planned colonoscopy for GI symptoms, surveillance after prior colonic pathology (polyps, CRC or any other than IBD), due to family history of CRC, or CRC screening	GI Genius™ - assisted colonoscopy (n=1015)	Standard HD colonoscopy (n=1017)	Median 10 years independent, 49.3% BCSP accredited	Parallel RCT	NA
Thiruvengadam 2024[57] (USA, single site)	≥30 years with any colonoscopy indication	GI Genius™ - assisted colonoscopy (n=550)	Standard white-light HD colonoscopy (n=550)	At least 1000 colonoscopies required with baseline ADR ≥25%	Parallel RCT	NA
Wallace 2022[58] (USA, Italy, UK – 8 sites)	≥45 years undergoing screening or surveillance colonoscopy for CRC	GI Genius™ - assisted colonoscopy (n=116)	Standard colonoscopy (n=114)	At least 1000 colonoscopies with ADR between 20 and 40% (or PDR between 30 and 70%)	Tandem RCT	NA
**CADx studies**						
Bernhofer 2025[59] (Austria, single site) – AC-CADx trial	≥18 years undergoing elective colonoscopy by a	GI Genius™ - assisted optical diagnosis (n=630 lesions)	NA – two comparator assessments reported but not	Trainee endoscopists (<500 colonoscopies and no formal optical diagnosis training).	Prospective non-randomised	Histology

	trainee endoscopist for any reason		relevant to review (autonomous AI assessment and expert optical diagnosis based on videos only)	Support from experienced endoscopists where required for polypectomies.		
Rondonotti 2024[60] (Italy, single site)	18 to 80 years referred for colonoscopy for screening, symptoms or post-polypectomy surveillance and with detection of at least one DRSP	GI Genius™-assisted optical diagnosis (n=376 polyps)	NA – autonomous AI assessment reported in paper but not extracted given adjunct AI assessment prioritised from this paper	Experts and non-experts in optical diagnosis included. Experts defined as dedicated training, prior studies in optical diagnosis, periodical auditing and monitoring according to ESGE curricula.<br><br>Met ESGE quality criteria and personal experience >300 colonoscopies	Prospective non-randomised	Histology
Hassan 2022[61] (Italy, single site) - CHANGE trial	≥40 years undergoing colonoscopy for primary CRC screening, post-polypectomy surveillance, symptoms or signs, or following a positive FIT	GI Genius™-assisted optical diagnosis (n=544 polyps)	NA – autonomous AI assessment reported in paper but not extracted given adjunct AI assessment prioritised from this paper	Endoscopists >2000 screening colonoscopies, trained in optical diagnosis and participating in prior studies on polyp characterisation with BLI	Prospective non-randomised	Histology
**MAGENTIQ-COLO™ (MAGENTIQ-EYE)**						
**CADe studies**						

Maas 2024 – MAGENTIQ-COLO™ [62] (Germany, Israel, Netherlands, USA – 10 sites)	18 to 90 years scheduled for non iFOBT screening or surveillance colonoscopy with last colonoscopy at least 3 years prior	MAGENTIQ-COLO™ -assisted colonoscopy (n=449)	Standard HD colonoscopy (n=467)	ADR between 25 and 40% required	Parallel and tandem RCT	NA
**CADx studies**						
None – not described as a function of MAGENTIQ-COLO™						

*n refers to the number of patients analysed, unless otherwise specified to be lesions analysed;

†Four separate groups with (n=308 CAD EYE®, n=315 without CAD EYE®) and without (n=312 CAD EYE®, n=310 without CAD EYE®) ENDOCUFF VISION™ reported but combined into two groups (CAD EYE®-assisted and standard colonoscopy) for the purpose of this analysis;

‡Study was considered to be at a high risk of bias and was not included in primary analyses in this assessment, unless it covered outcomes not covered by other studies;

§Kudo, NICE and Kudo-IBD classifications reported as comparators for diagnostic accuracy data;

‖The ENDOCUFF VISION™ + ENDO-AID™ (n=230) and ENDO-AID™ only (n=238) groups in this study were combined into a single ENDO-AID™ group for the purpose of this analysis;

¶Only the two novice groups (with and without ENDOANGEL®; n=227 and n=229) from this study were included in analyses in this assessment given the third group (expert endoscopists without ENDOANGEL®) is not comparable to either of the other two groups given the endoscopist experience differs and may introduce additional bias into results.

Note: studies included in this table are a subset of those that were included in the full evidence assessment report produced for the NICE appraisal.[11] Specifically, this publication focuses on data from trials that used the AI systems of interest as an adjunct to endoscopist judgement and reported on outcomes prioritised for inclusion in this publication, and data for CADDIE™ and EMIS™ systems are not included given these data were not publicly available at the time of writing.

Abbreviations: ADR, adenoma detection rate; AI, artificial intelligence; BCSP, Bowel Cancer Screening Programme; BLI, blue-light imaging; CADe, computer-aided detection; CADx, computer-aided characterisation; CRC, colorectal cancer; DA, diagnostic accuracy; DCE, dye-based chromoendoscopy; DRSP, diminutive rectosigmoid polyp; EPCAM, epithelial cell adhesion molecule gene; EMIS™, Endoscopic Multimedia Information System™; ESGE, European Society of Gastrointestinal Endoscopy; FIT, faecal immunochemical test; FOBT, faecal occult blood test; GI, gastrointestinal; HD, high-definition; HDWL, high-definition white-light; IBD, inflammatory bowel disease; iFOBT, immunochemical faecal occult blood test; LCI, linked-colour imaging; LS, Lynch syndrome; MLH1, mutL homolog 1; MSH2, mutS homolog 2; MSH6, mutS homolog 6; NA, not applicable; NBI, narrow-band imaging; NICE, National Institute for Health and Care Excellence; PDR, polyp detection rate; RCT, randomised controlled trial; UC, ulcerative colitis; VCE, virtual chromoendoscopy; WLE, white-light endoscopy.

2 Additional SLR results

2.1 Computer-aided polyp detection (CADe) data

2.1.1 Advanced ADR

Supplementary Figure 4. Meta-analyses of advanced ADR for each included CADe system

Study or Subgroup	CADe Events	CADe Total	No CADe Events	No CADe Total	Weight	Risk Ratio M-H, Random, 95% CI
**12.2.2 CAD EYE®**						
Aniwan 2023	68	620	50	625	18.2%	1.37 [0.97, 1.94]
Desai 2024	33	509	33	522	12.4%	1.03 [0.64, 1.64]
Djinbachian 2024	30	229	19	238	9.8%	1.64 [0.95, 2.83]
Huneburg 2023	4	50	2	46	1.3%	1.84 [0.35, 9.58]
Nakashima 2023	15	207	16	208	6.9%	0.94 [0.48, 1.86]
Rondonotti 2022	75	405	63	395	21.0%	1.16 [0.86, 1.58]
Tiankanon 2024	41	400	24	400	11.7%	1.71 [1.05, 2.77]
Zimmermann-Fraedrich 2025	55	812	68	815	18.6%	0.81 [0.58, 1.14]
**Subtotal (95% CI)**		**3232**		**3249**	**100.0%**	**1.18 [0.98, 1.44]**
Total events	321		275			
Heterogeneity: Tau² = 0.02; Chi² = 10.00, df = 7 (P = 0.19); I² = 30%						
Test for overall effect: Z = 1.71 (P = 0.09)						
**12.2.4 ENDO-AID™**						
Gimeno-Garcia 2023	18	155	19	157	15.9%	0.96 [0.52, 1.76]
Lau 2024	32	386	38	380	26.0%	0.83 [0.53, 1.30]
Lui 2024	81	468	25	214	29.0%	1.48 [0.98, 2.25]
Spada 2025	45	611	38	617	29.1%	1.20 [0.79, 1.81]
**Subtotal (95% CI)**		**1620**		**1368**	**100.0%**	**1.12 [0.86, 1.45]**
Total events	176		120			
Heterogeneity: Tau² = 0.01; Chi² = 3.79, df = 3 (P = 0.28); I² = 21%						
Test for overall effect: Z = 0.84 (P = 0.40)						
**12.2.5 ENDOANGEL®**						
Yao 2022	5	268	5	271	36.4%	1.01 [0.30, 3.45]
Yao 2024	11	227	7	229	63.6%	1.59 [0.63, 4.02]
**Subtotal (95% CI)**		**495**		**500**	**100.0%**	**1.35 [0.64, 2.82]**
Total events	16		12			
Heterogeneity: Tau² = 0.00; Chi² = 0.33, df = 1 (P = 0.57); I² = 0%						
Test for overall effect: Z = 0.79 (P = 0.43)						
**12.2.7 GI Genius™**						
Karsenti 2023	93	1003	77	1012	8.0%	1.22 [0.91, 1.63]
Mangas-Sanjuan 2023	490	1610	500	1603	62.5%	0.98 [0.88, 1.08]
Repici 2020	35	341	33	344	3.3%	1.07 [0.68, 1.68]
Repici 2022	42	330	42	330	4.2%	1.00 [0.67, 1.49]
Seager 2024	180	1001	187	1009	19.6%	0.97 [0.81, 1.17]
Thiruvengadam 2024	26	550	24	550	2.3%	1.08 [0.63, 1.86]
**Subtotal (95% CI)**		**4835**		**4848**	**100.0%**	**1.00 [0.92, 1.08]**
Total events	866		863			
Heterogeneity: Tau² = 0.00; Chi² = 2.30, df = 5 (P = 0.81); I² = 0%						
Test for overall effect: Z = 0.03 (P = 0.97)						

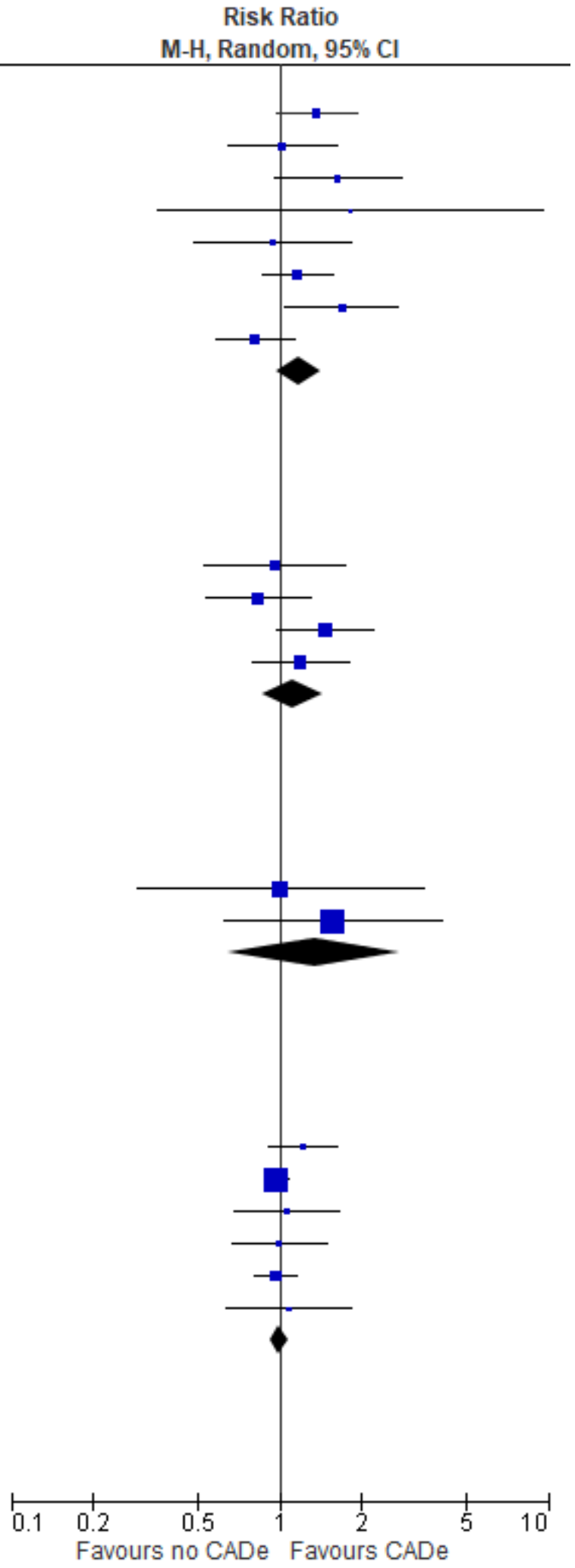

Abbreviations: ADR, adenoma detection rate; CADe, computer-aided polyp detection; CI, confidence interval; M-H, Mantel-Haenszel.

2.1.2 Non-advanced ADR

Supplementary Figure 5. Meta-analyses of non-advanced ADR for each included CADe system

Study or Subgroup	CADe Events	CADe Total	No CADe Events	No CADe Total	Weight	Risk Ratio M-H, Random, 95% CI
**12.3.4 ENDO-AID™**						
Gimeno-Garcia 2023	85	155	64	157	100.0%	1.35 [1.06, 1.70]
**Subtotal (95% CI)**		**155**		**157**	**100.0%**	**1.35 [1.06, 1.70]**
Total events	85		64			
Heterogeneity: Not applicable						
Test for overall effect: Z = 2.46 (P = 0.01)						
**12.3.5 ENDOANGEL®**						
Yao 2022	53	268	37	271	100.0%	1.45 [0.99, 2.13]
**Subtotal (95% CI)**		**268**		**271**	**100.0%**	**1.45 [0.99, 2.13]**
Total events	53		37			
Heterogeneity: Not applicable						
Test for overall effect: Z = 1.89 (P = 0.06)						
**12.3.7 GI Genius™**						
Repici 2020	142	341	103	344	27.3%	1.39 [1.13, 1.71]
Repici 2022	134	330	105	330	27.3%	1.28 [1.04, 1.57]
Thiruvengadam 2024	223	550	175	550	45.4%	1.27 [1.09, 1.49]
**Subtotal (95% CI)**		**1221**		**1224**	**100.0%**	**1.31 [1.17, 1.45]**
Total events	499		383			
Heterogeneity: Tau² = 0.00; Chi² = 0.50, df = 2 (P = 0.78); I² = 0%						
Test for overall effect: Z = 4.89 (P < 0.00001)						

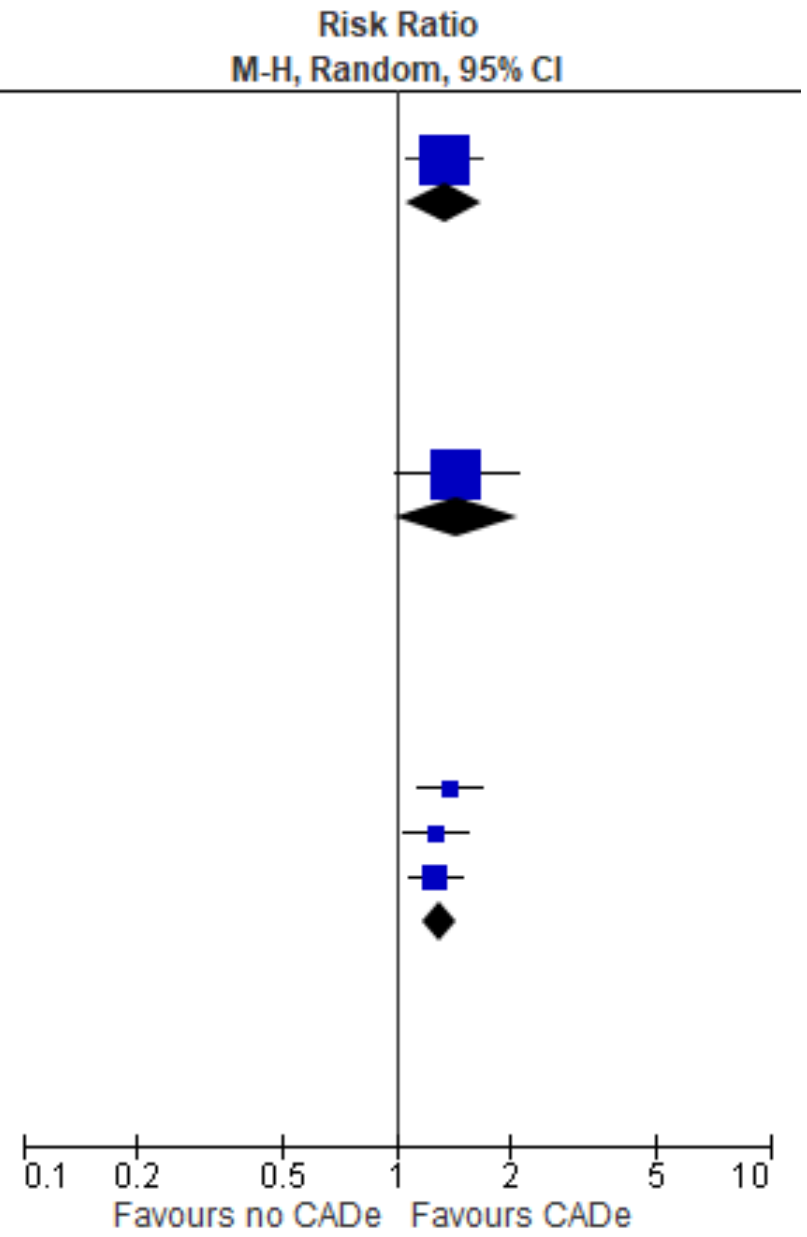

Abbreviations: ADR, adenoma detection rate; CADe, computer-aided polyp detection; CI, confidence interval; M-H, Mantel-Haenszel.

2.1.3 ADR by size

Supplementary Figure 6. Meta-analyses of ADR for different size categories – CAD EYE®

	CADe		No CADe			Risk Ratio
**Study or Subgroup**	**Events**	**Total**	**Events**	**Total**	**Weight**	**M-H, Random, 95% CI**
**12.4.1 ≤5 mm (or <5 mm)**						
Desai 2024	181	509	159	522	32.8%	1.17 [0.98, 1.39]
Rondonotti 2022	170	405	131	395	30.5%	1.27 [1.06, 1.52]
Zimmermann-Fraedrich 2025	218	812	202	815	36.7%	1.08 [0.92, 1.28]
**Subtotal (95% CI)**		**1726**		**1732**	**100.0%**	**1.16 [1.05, 1.29]**
Total events	569		492			
Heterogeneity: Tau² = 0.00; Chi² = 1.56, df = 2 (P = 0.46); I² = 0%						
Test for overall effect: Z = 2.98 (P = 0.003)						
**12.4.2 6-9 mm**						
Rondonotti 2022	64	405	53	395	21.6%	1.18 [0.84, 1.65]
Zimmermann-Fraedrich 2025	199	812	180	815	78.4%	1.11 [0.93, 1.32]
**Subtotal (95% CI)**		**1217**		**1210**	**100.0%**	**1.12 [0.96, 1.31]**
Total events	263		233			
Heterogeneity: Tau² = 0.00; Chi² = 0.09, df = 1 (P = 0.76); I² = 0%						
Test for overall effect: Z = 1.46 (P = 0.14)						
**12.4.3 <10 mm**						
Aniwan 2023	280	620	248	625	100.0%	1.14 [1.00, 1.30]
**Subtotal (95% CI)**		**620**		**625**	**100.0%**	**1.14 [1.00, 1.30]**
Total events	280		248			
Heterogeneity: Not applicable						
Test for overall effect: Z = 1.95 (P = 0.05)						
**12.4.4 ≥ 10 mm**						
Aniwan 2023	64	620	52	625	28.1%	1.24 [0.88, 1.76]
Rondonotti 2022	64	405	57	395	31.5%	1.10 [0.79, 1.52]
Zimmermann-Fraedrich 2025	79	812	85	815	40.4%	0.93 [0.70, 1.25]
**Subtotal (95% CI)**		**1837**		**1835**	**100.0%**	**1.06 [0.88, 1.28]**
Total events	207		194			
Heterogeneity: Tau² = 0.00; Chi² = 1.56, df = 2 (P = 0.46); I² = 0%						
Test for overall effect: Z = 0.65 (P = 0.52)						

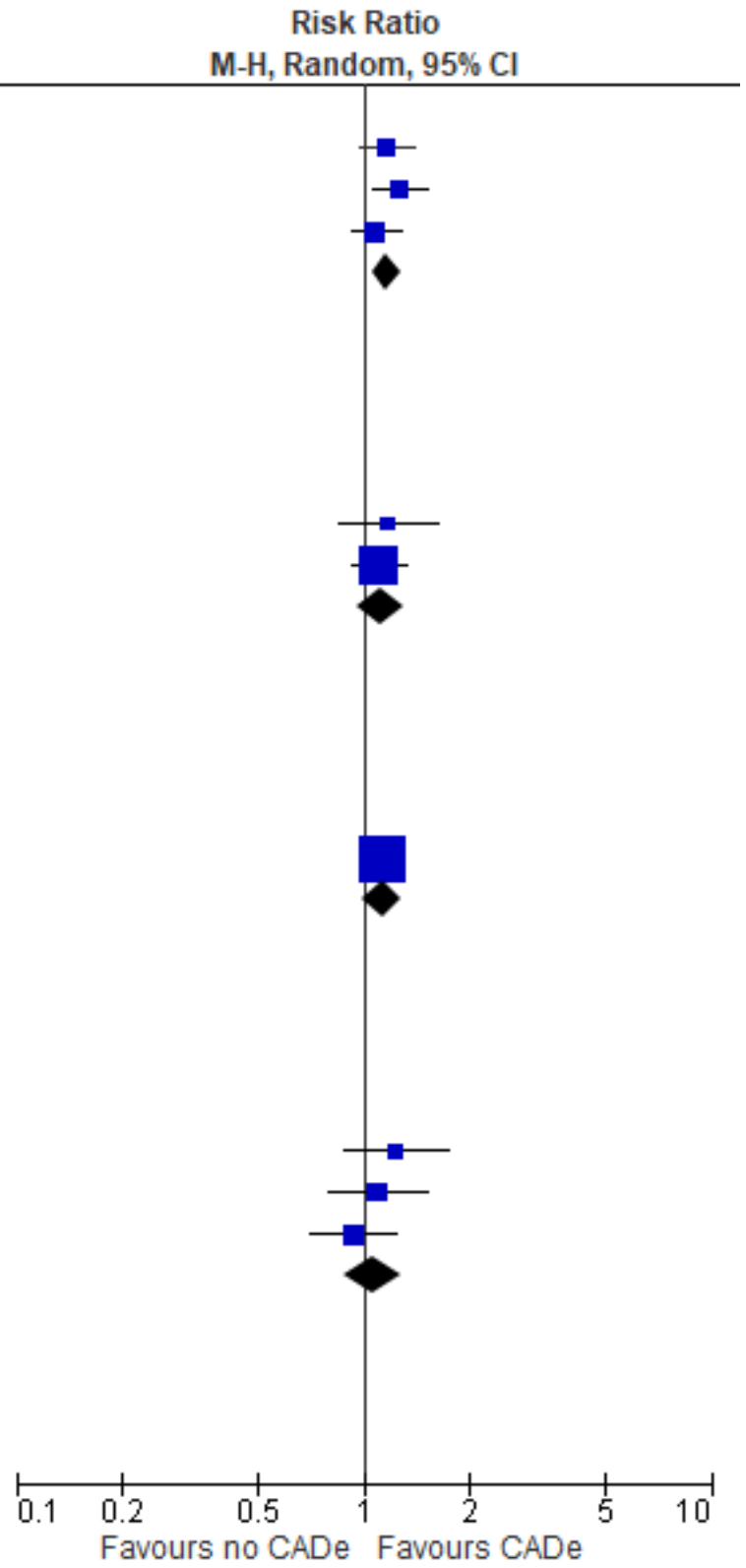

Abbreviations: ADR, adenoma detection rate; CADe, computer-aided polyp detection; CI, confidence interval; M-H, Mantel-Haenszel.

Supplementary Figure 7. Meta-analyses of ADR for different size categories – Discovery™

Study or Subgroup	CADe Events	CADe Total	No CADe Events	No CADe Total	Weight	Risk Ratio M-H, Random, 95% CI
**12.5.1 ≤5 mm**						
Maas 2024 (Discovery)	77	250	67	247	100.0%	1.14 [0.86, 1.50]
**Subtotal (95% CI)**		**250**		**247**	**100.0%**	**1.14 [0.86, 1.50]**
Total events	77		67			
Heterogeneity: Not applicable						
Test for overall effect: Z = 0.90 (P = 0.37)						
**12.5.2 6-9 mm**						
Maas 2024 (Discovery)	28	250	38	247	100.0%	0.73 [0.46, 1.15]
**Subtotal (95% CI)**		**250**		**247**	**100.0%**	**0.73 [0.46, 1.15]**
Total events	28		38			
Heterogeneity: Not applicable						
Test for overall effect: Z = 1.37 (P = 0.17)						
**12.5.4 ≥ 10 mm**						
Maas 2024 (Discovery)	14	250	14	247	100.0%	0.99 [0.48, 2.03]
**Subtotal (95% CI)**		**250**		**247**	**100.0%**	**0.99 [0.48, 2.03]**
Total events	14		14			
Heterogeneity: Not applicable						
Test for overall effect: Z = 0.03 (P = 0.97)						

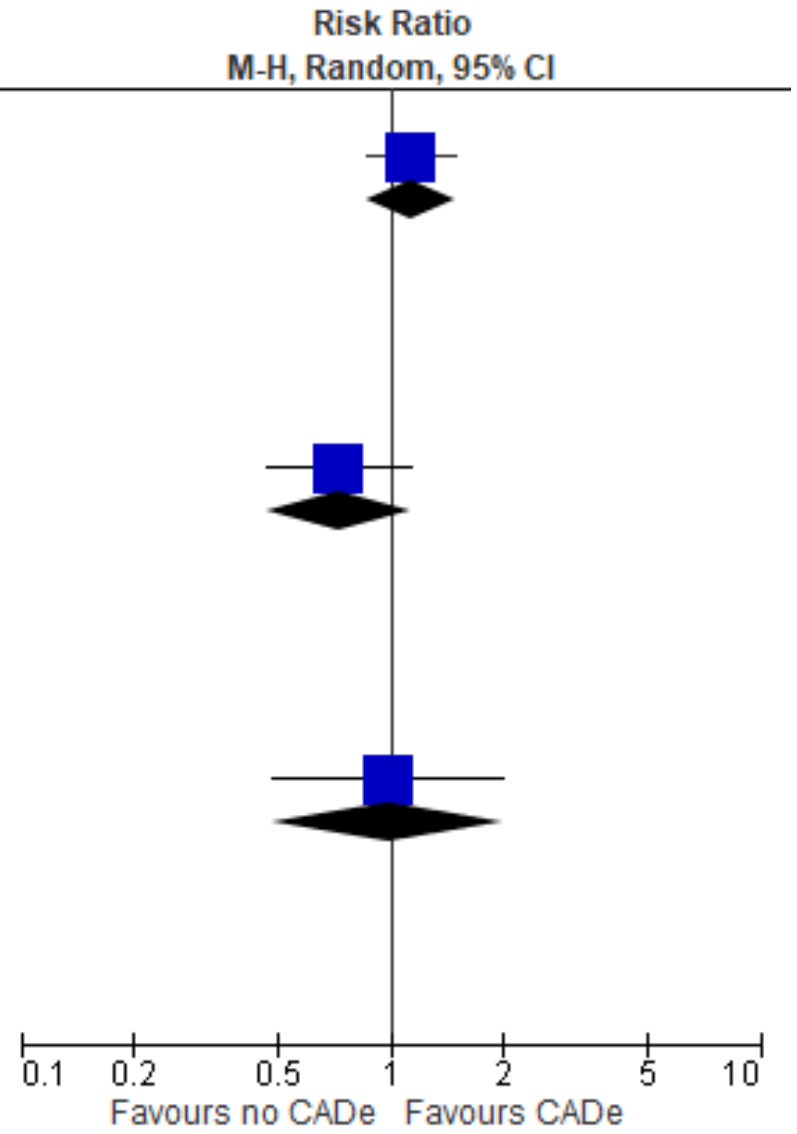

Abbreviations: ADR, adenoma detection rate; CADe, computer-aided polyp detection; CI, confidence interval; M-H, Mantel-Haenszel.

Supplementary Figure 8. Meta-analyses of ADR for different size categories – ENDO-AID™

Study or Subgroup	CADe Events	CADe Total	No CADe Events	No CADe Total	Weight	Risk Ratio M-H, Random, 95% CI
**12.6.1 <5 or ≤5 mm**						
Gimeno-Garcia 2023	80	155	57	155	40.6%	1.40 [1.09, 1.81]
Lau 2024	156	386	95	380	59.4%	1.62 [1.31, 2.00]
**Subtotal (95% CI)**		**541**		**535**	**100.0%**	**1.53 [1.30, 1.80]**
Total events	236		152			
Heterogeneity: Tau² = 0.00; Chi² = 0.70, df = 1 (P = 0.40); I² = 0%						
Test for overall effect: Z = 5.07 (P < 0.00001)						
**12.6.2 5-10 or 6-9 mm**						
Gimeno-Garcia 2023	24	155	22	155	12.7%	1.09 [0.64, 1.86]
Lau 2024	142	386	111	380	87.3%	1.26 [1.03, 1.54]
**Subtotal (95% CI)**		**541**		**535**	**100.0%**	**1.24 [1.02, 1.50]**
Total events	166		133			
Heterogeneity: Tau² = 0.00; Chi² = 0.24, df = 1 (P = 0.62); I² = 0%						
Test for overall effect: Z = 2.18 (P = 0.03)						
**12.6.4 >10 or ≥ 10 mm**						
Gimeno-Garcia 2023	17	155	15	155	54.6%	1.13 [0.59, 2.19]
Lau 2024	7	386	16	380	45.4%	0.43 [0.18, 1.04]
**Subtotal (95% CI)**		**541**		**535**	**100.0%**	**0.73 [0.28, 1.88]**
Total events	24		31			
Heterogeneity: Tau² = 0.31; Chi² = 3.01, df = 1 (P = 0.08); I² = 67%						
Test for overall effect: Z = 0.65 (P = 0.52)						

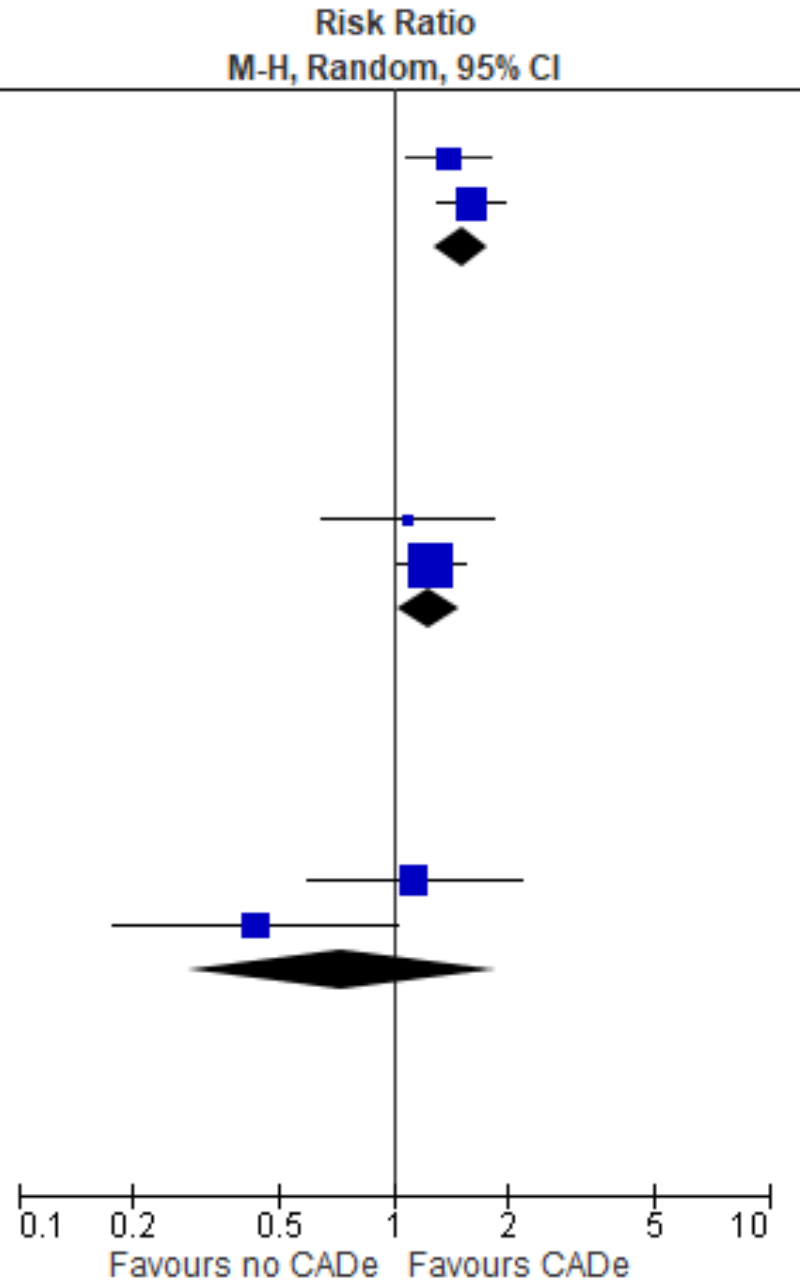

Abbreviations: ADR, adenoma detection rate; CADe, computer-aided polyp detection; CI, confidence interval; M-H, Mantel-Haenszel.

Supplementary Figure 9. Meta-analyses of ADR for different size categories – ENDOANGEL®

Study or Subgroup	CADe Events	CADe Total	No CADe Events	No CADe Total	Weight	Risk Ratio M-H, Random, 95% CI
**12.7.1 ≤5 mm**						
Yao 2022	54	268	39	271	61.5%	1.40 [0.96, 2.04]
Yao 2024	33	227	27	229	38.5%	1.23 [0.77, 1.98]
**Subtotal (95% CI)**		**495**		**500**	**100.0%**	**1.33 [0.99, 1.79]**
Total events	87		66			
Heterogeneity: Tau² = 0.00; Chi² = 0.17, df = 1 (P = 0.68); I² = 0%						
Test for overall effect: Z = 1.91 (P = 0.06)						
**12.7.2 6-9 mm**						
Yao 2022	13	268	4	271	39.1%	3.29 [1.09, 9.95]
Yao 2024	14	227	10	229	60.9%	1.41 [0.64, 3.11]
**Subtotal (95% CI)**		**495**		**500**	**100.0%**	**1.96 [0.87, 4.42]**
Total events	27		14			
Heterogeneity: Tau² = 0.12; Chi² = 1.49, df = 1 (P = 0.22); I² = 33%						
Test for overall effect: Z = 1.63 (P = 0.10)						
**12.7.4 ≥ 10 mm**						
Yao 2022	2	268	2	271	25.2%	1.01 [0.14, 7.13]
Yao 2024	7	227	5	229	74.8%	1.41 [0.45, 4.38]
**Subtotal (95% CI)**		**495**		**500**	**100.0%**	**1.30 [0.49, 3.46]**
Total events	9		7			
Heterogeneity: Tau² = 0.00; Chi² = 0.08, df = 1 (P = 0.77); I² = 0%						
Test for overall effect: Z = 0.52 (P = 0.60)						

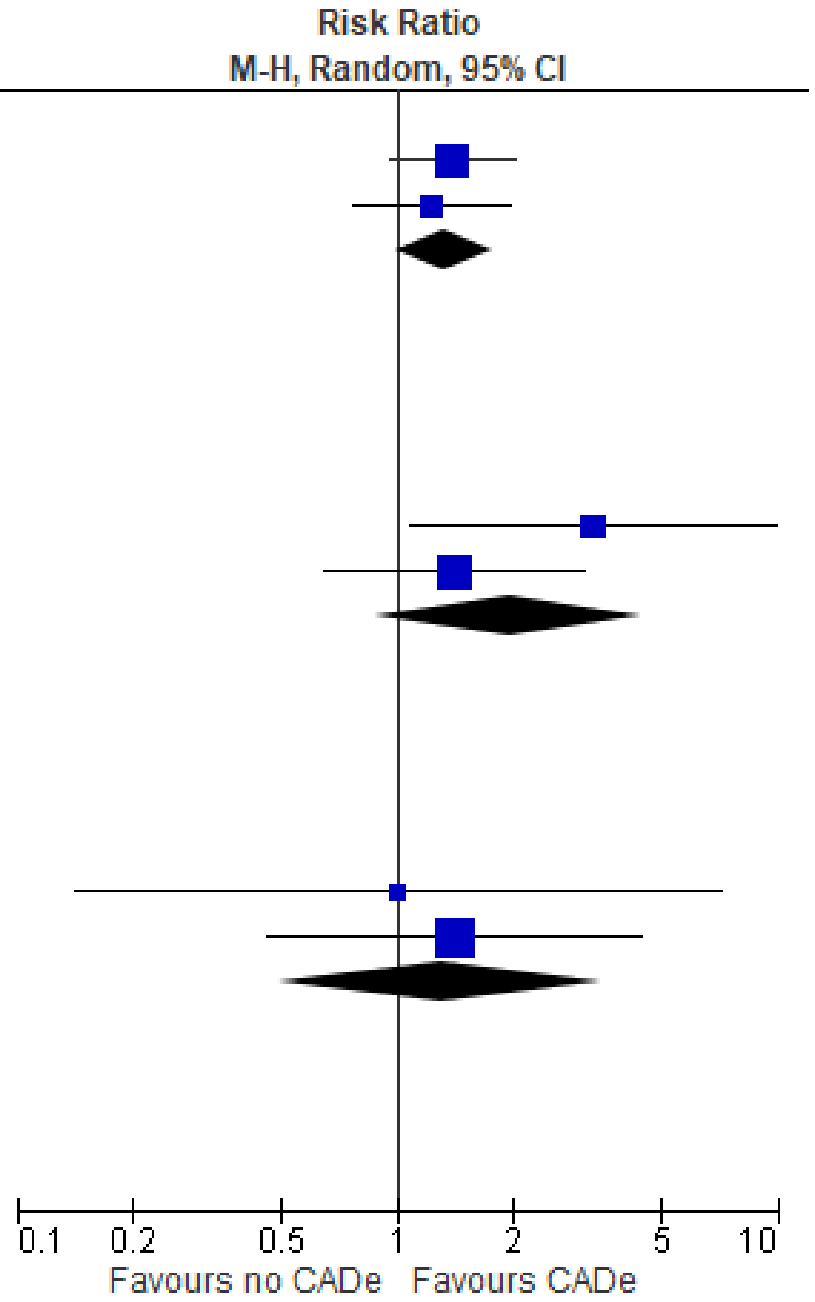

Abbreviations: ADR, adenoma detection rate; CADe, computer-aided polyp detection; CI, confidence interval; M-H, Mantel-Haenszel.

Supplementary Figure 10. Meta-analyses of ADR for different size categories – GI Genius™

Study or Subgroup	CADe Events	CADe Total	No CADe Events	No CADe Total	Weight	Risk Ratio M-H, Random, 95% CI
**12.8.1 ≤5 mm**						
Mangas-Sanjuan 2023	761	1610	681	1603	81.8%	1.11 [1.03, 1.20]
Repici 2020	115	341	91	344	9.1%	1.27 [1.01, 1.61]
Repici 2022	109	330	94	330	9.1%	1.16 [0.92, 1.46]
**Subtotal (95% CI)**		**2281**		**2277**	**100.0%**	**1.13 [1.05, 1.21]**
Total events	985		866			
Heterogeneity: Tau² = 0.00; Chi² = 1.26, df = 2 (P = 0.53); I² = 0%						
Test for overall effect: Z = 3.46 (P = 0.0005)						
**12.8.2 6-9 mm**						
Mangas-Sanjuan 2023	340	1610	314	1603	55.8%	1.08 [0.94, 1.24]
Repici 2020	36	341	20	344	21.0%	1.82 [1.07, 3.07]
Repici 2022	34	330	26	330	23.2%	1.31 [0.80, 2.13]
**Subtotal (95% CI)**		**2281**		**2277**	**100.0%**	**1.26 [0.94, 1.69]**
Total events	410		360			
Heterogeneity: Tau² = 0.04; Chi² = 3.94, df = 2 (P = 0.14); I² = 49%						
Test for overall effect: Z = 1.53 (P = 0.13)						
**12.8.3 <10 mm**						
Repici 2022	143	330	120	330	100.0%	1.19 [0.99, 1.44]
**Subtotal (95% CI)**		**330**		**330**	**100.0%**	**1.19 [0.99, 1.44]**
Total events	143		120			
Heterogeneity: Not applicable						
Test for overall effect: Z = 1.82 (P = 0.07)						
**12.8.4 ≥ 10 mm**						
Mangas-Sanjuan 2023	437	1610	423	1603	89.8%	1.03 [0.92, 1.15]
Repici 2020	36	341	28	344	5.3%	1.30 [0.81, 2.08]
Repici 2022	32	330	27	330	4.9%	1.19 [0.73, 1.93]
**Subtotal (95% CI)**		**2281**		**2277**	**100.0%**	**1.05 [0.94, 1.17]**
Total events	505		478			
Heterogeneity: Tau² = 0.00; Chi² = 1.14, df = 2 (P = 0.57); I² = 0%						
Test for overall effect: Z = 0.86 (P = 0.39)						

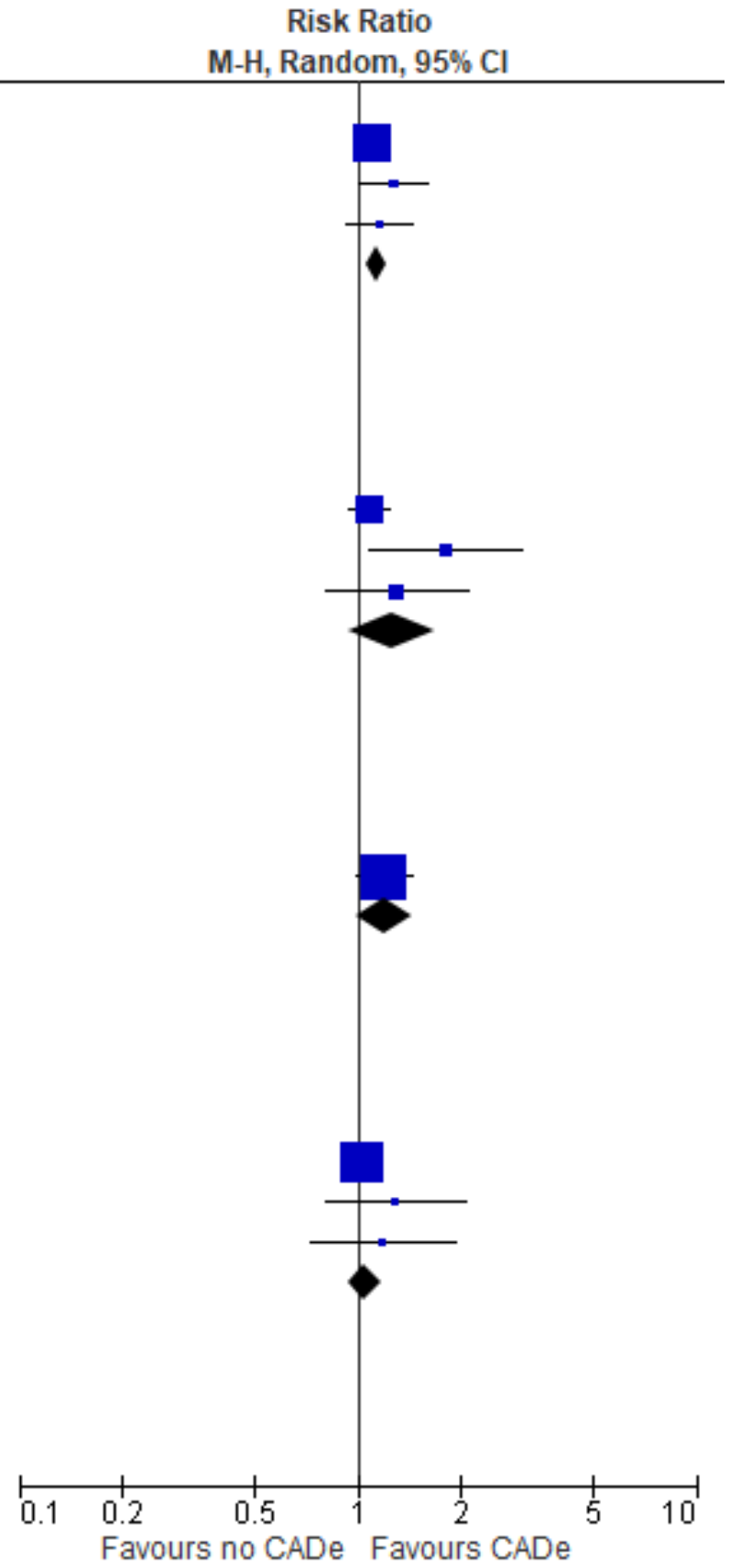

Abbreviations: ADR, adenoma detection rate; CADe, computer-aided polyp detection; CI, confidence interval; M-H, Mantel-Haenszel.

2.1.4 Overall APC analysed as mean difference

Supplementary Figure 11. Meta-analyses of overall APC as a mean difference

Study or Subgroup	Mean Difference	SE	Weight	Mean Difference IV, Random, 95% CI
**12.9.1 CAD EYE®**				
Alali 2025	0.24	0.1786	4.6%	0.24 [-0.11, 0.59]
Aniwan 2023	0.24	0.0918	17.3%	0.24 [0.06, 0.42]
Desai 2024	0.14	0.0969	15.6%	0.14 [-0.05, 0.33]
Djinbachian 2024	0.11	0.1173	10.6%	0.11 [-0.12, 0.34]
Miyaguchi 2024	0.37	0.1071	12.7%	0.37 [0.16, 0.58]
Nakashima 2023	0.2	0.1429	7.2%	0.20 [-0.08, 0.48]
Rondonotti 2022	0.22	0.102	14.1%	0.22 [0.02, 0.42]
Tiankanon 2024	0.38	0.0969	15.6%	0.38 [0.19, 0.57]
Yamaguchi 2024	0	0.25	2.3%	0.00 [-0.49, 0.49]
**Subtotal (95% CI)**			**100.0%**	**0.24 [0.16, 0.31]**
Heterogeneity: Tau² = 0.00; Chi² = 6.89, df = 8 (P = 0.55); I² = 0%				
Test for overall effect: Z = 6.22 (P < 0.00001)				
**12.9.2 Discovery™**				
Maas 2024 (Discovery)	0	0.0969	100.0%	0.00 [-0.19, 0.19]
**Subtotal (95% CI)**			**100.0%**	**0.00 [-0.19, 0.19]**
Heterogeneity: Not applicable				
Test for overall effect: Z = 0.00 (P = 1.00)				
**12.9.3 ENDO-AID™**				
Gimeno-Garcia 2023	0.62	0.2041	2.5%	0.62 [0.22, 1.02]
Lau 2024	0.62	0.1327	5.7%	0.62 [0.36, 0.88]
Lui 2024	0.45	0.0051	80.7%	0.45 [0.44, 0.46]
Spada 2025	0.35	0.0918	11.1%	0.35 [0.17, 0.53]
**Subtotal (95% CI)**			**100.0%**	**0.45 [0.39, 0.52]**
Heterogeneity: Tau² = 0.00; Chi² = 3.52, df = 3 (P = 0.32); I² = 15%				
Test for overall effect: Z = 13.78 (P < 0.00001)				
**12.9.4 ENDOANGEL®**				
Yao 2022	0.07	0.0357	77.5%	0.07 [0.00, 0.14]
Yao 2024	0.05	0.0663	22.5%	0.05 [-0.08, 0.18]
**Subtotal (95% CI)**			**100.0%**	**0.07 [0.00, 0.13]**
Heterogeneity: Tau² = 0.00; Chi² = 0.07, df = 1 (P = 0.79); I² = 0%				
Test for overall effect: Z = 2.08 (P = 0.04)				
**12.9.5 EndoScreener®**				
Glissen Brown 2022	0.29	0.2398	100.0%	0.29 [-0.18, 0.76]
**Subtotal (95% CI)**			**100.0%**	**0.29 [-0.18, 0.76]**
Heterogeneity: Not applicable				
Test for overall effect: Z = 1.21 (P = 0.23)				
**12.9.6 GI Genius™**				
Ahmad 2023	0.3	0.2245	2.2%	0.30 [-0.14, 0.74]
Karsenti 2023	0.18	0.0612	29.5%	0.18 [0.06, 0.30]
Mangas-Sanjuan 2023	0.19	0.0816	16.6%	0.19 [0.03, 0.35]
Ortiz 2024	0	0.1327	6.3%	0.00 [-0.26, 0.26]
Repici 2020	0.36	0.1071	9.6%	0.36 [0.15, 0.57]
Repici 2022	0.22	0.1378	5.8%	0.22 [-0.05, 0.49]
Seager 2024	0.35	0.1071	9.6%	0.35 [0.14, 0.56]
Thiruvengadam 2024	0.29	0.0765	18.9%	0.29 [0.14, 0.44]
Wallace 2022	0.33	0.2908	1.3%	0.33 [-0.24, 0.90]
**Subtotal (95% CI)**			**100.0%**	**0.23 [0.17, 0.30]**
Heterogeneity: Tau² = 0.00; Chi² = 7.47, df = 8 (P = 0.49); I² = 0%				
Test for overall effect: Z = 6.97 (P < 0.00001)				
**12.9.7 MAGENTIQ-COLO™**				
Maas 2024 (MAGENTIQ-COLO)	0.19	0.0765	100.0%	0.19 [0.04, 0.34]
**Subtotal (95% CI)**			**100.0%**	**0.19 [0.04, 0.34]**
Heterogeneity: Not applicable				
Test for overall effect: Z = 2.48 (P = 0.01)				

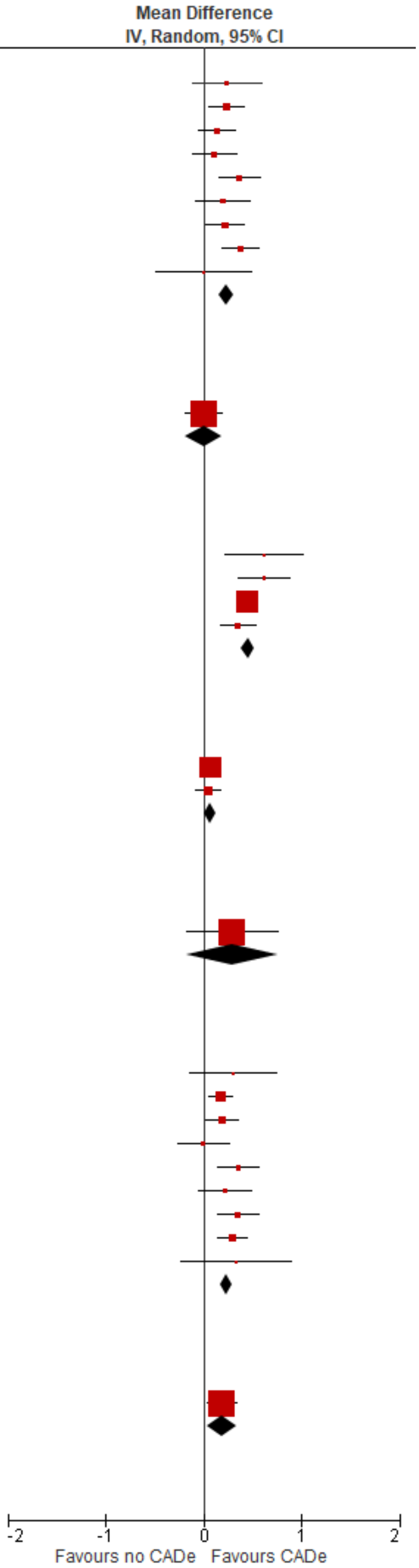

Abbreviations: APC, adenomas per colonoscopy; CADe, computer-aided polyp detection; CI, confidence interval; IV, inverse variance; SE, standard error.

2.1.5 Per-patient AMR analyses

Supplementary Figure 12. Meta-analyses of AMR – per-patient analysed as a risk ratio

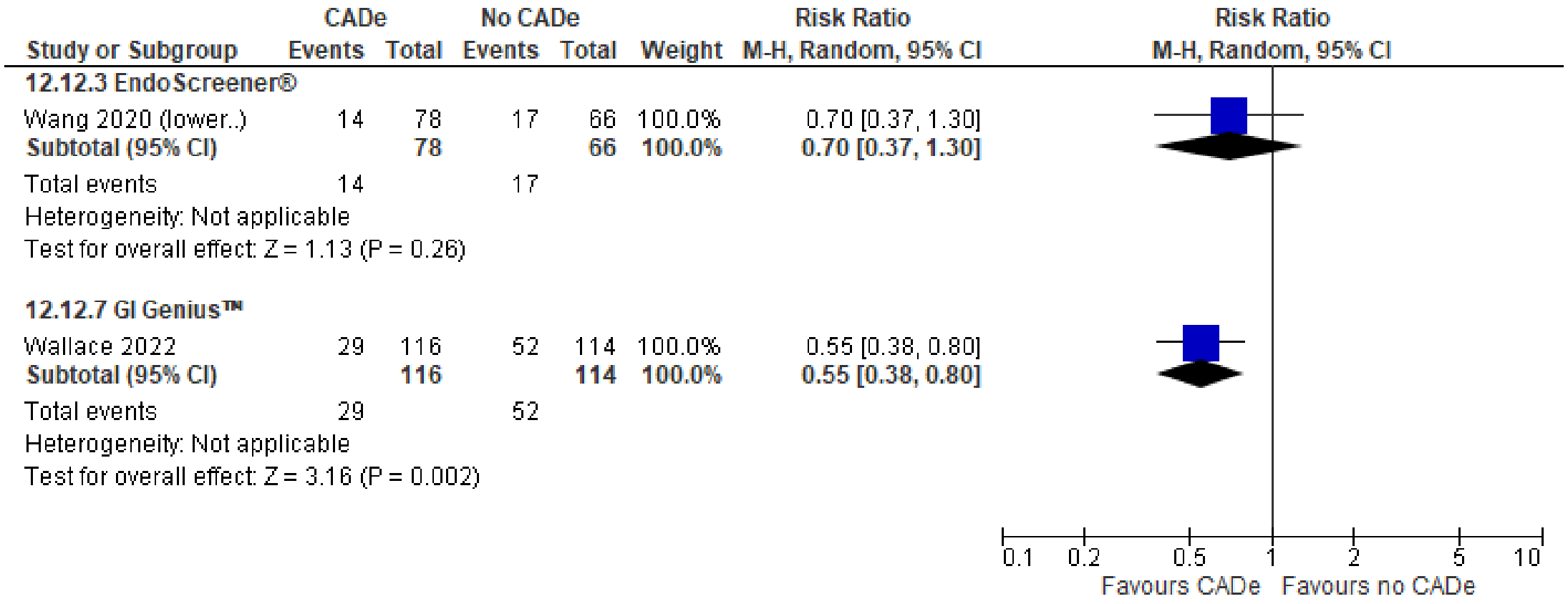

Abbreviations: AMR, adenoma miss rate; CADe, computer-aided polyp detection; CI, confidence interval; M-H, Mantel-Haenszel.

Supplementary Figure 13. Meta-analyses of AMR – per-patient analysed as a mean difference

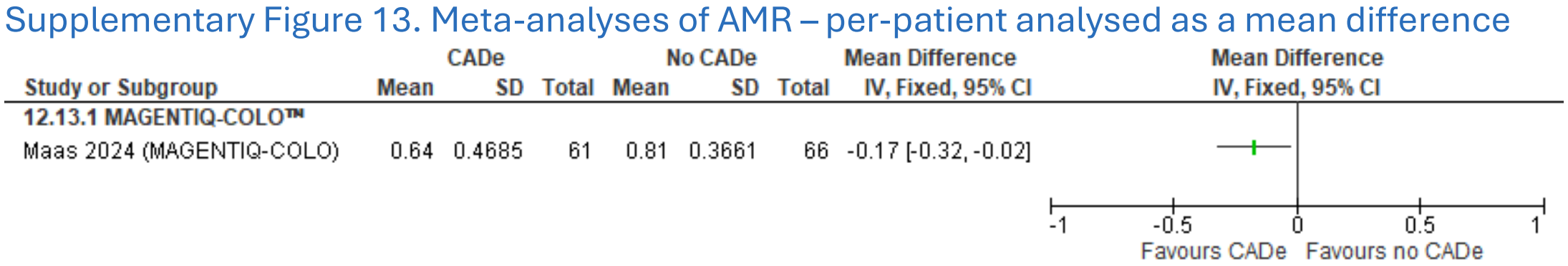

Abbreviations: AMR, adenoma miss rate; CADe, computer-aided polyp detection; CI, confidence interval; IV, inverse variance; SD standard deviation.

2.1.6 Significant polyp detection rate (adenoma or sessile serrated lesion) analyses

Supplementary Figure 14. Analysis of significant polyp detection rate (adenoma or sessile serrated lesion) – GI Genius™

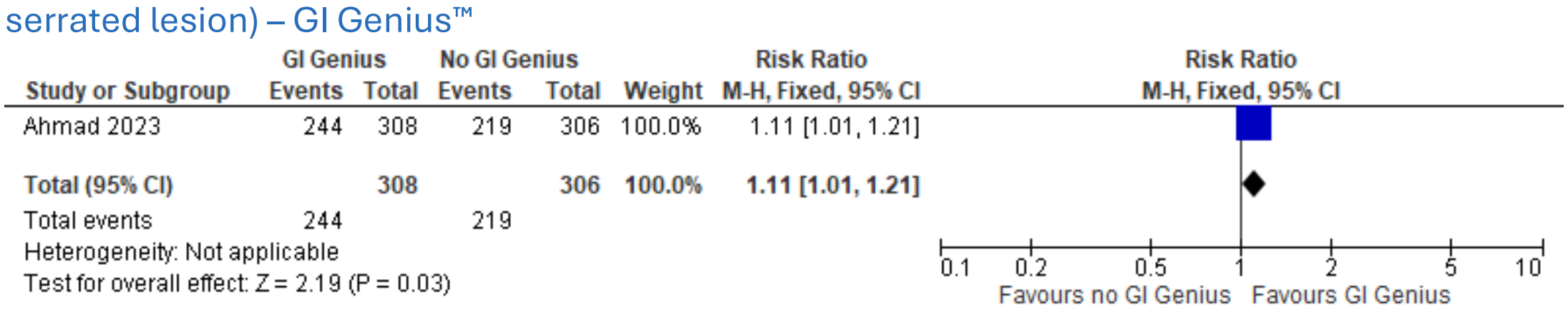

Abbreviations: CI, confidence interval; M-H, Mantel-Haenszel.

2.1.7 Hyperplastic/non-neoplastic polyp detection rate

Supplementary Figure 15. Meta-analyses of hyperplastic/non-neoplastic polyp detection or resection rate

Study or Subgroup	CADe Events	CADe Total	No CADe Events	No CADe Total	Weight	Risk Ratio M-H, Random, 95% CI
**12.16.1 CAD EYE® - non-neoplastic/hyperplastic polyp detection rate**						
Huneburg 2023	22	50	22	46	11.4%	0.92 [0.60, 1.42]
Rondonotti 2022	81	405	58	395	22.1%	1.36 [1.00, 1.85]
Zimmermann-Fraedrich 2025	229	812	188	815	66.5%	1.22 [1.03, 1.44]
**Subtotal (95% CI)**		**1267**		**1256**	**100.0%**	**1.21 [1.04, 1.41]**
Total events	332		268			
Heterogeneity: Tau² = 0.00; Chi² = 2.13, df = 2 (P = 0.34); I² = 6%						
Test for overall effect: Z = 2.54 (P = 0.01)						
**12.16.2 ENDO-AID™ - non-neoplastic detection/resection rate**						
Gimeno-Garcia 2023	47	155	29	157	14.5%	1.64 [1.09, 2.46]
Lau 2024	201	386	133	380	85.5%	1.49 [1.26, 1.76]
**Subtotal (95% CI)**		**541**		**537**	**100.0%**	**1.51 [1.29, 1.76]**
Total events	248		162			
Heterogeneity: Tau² = 0.00; Chi² = 0.20, df = 1 (P = 0.66); I² = 0%						
Test for overall effect: Z = 5.22 (P < 0.00001)						
**12.16.4 ENDOANGEL® - non-precancerous polyp detection rate**						
Yao 2022	126	268	94	271	100.0%	1.36 [1.10, 1.67]
**Subtotal (95% CI)**		**268**		**271**	**100.0%**	**1.36 [1.10, 1.67]**
Total events	126		94			
Heterogeneity: Not applicable						
Test for overall effect: Z = 2.88 (P = 0.004)						
**12.16.5 GI Genius™ - non-neoplastic polyp detection rate**						
Repici 2020	68	341	57	344	25.4%	1.20 [0.88, 1.65]
Repici 2022	40	330	39	330	15.0%	1.03 [0.68, 1.55]
Thiruvengadam 2024	139	550	130	550	59.6%	1.07 [0.87, 1.32]
**Subtotal (95% CI)**		**1221**		**1224**	**100.0%**	**1.09 [0.93, 1.29]**
Total events	247		226			
Heterogeneity: Tau² = 0.00; Chi² = 0.48, df = 2 (P = 0.78); I² = 0%						
Test for overall effect: Z = 1.11 (P = 0.27)						
**12.16.7 GI Genius™ - non-neoplastic polyp resection rate**						
Repici 2020	68	262	57	198	100.0%	0.90 [0.67, 1.22]
**Subtotal (95% CI)**		**262**		**198**	**100.0%**	**0.90 [0.67, 1.22]**
Total events	68		57			
Heterogeneity: Not applicable						
Test for overall effect: Z = 0.68 (P = 0.50)						

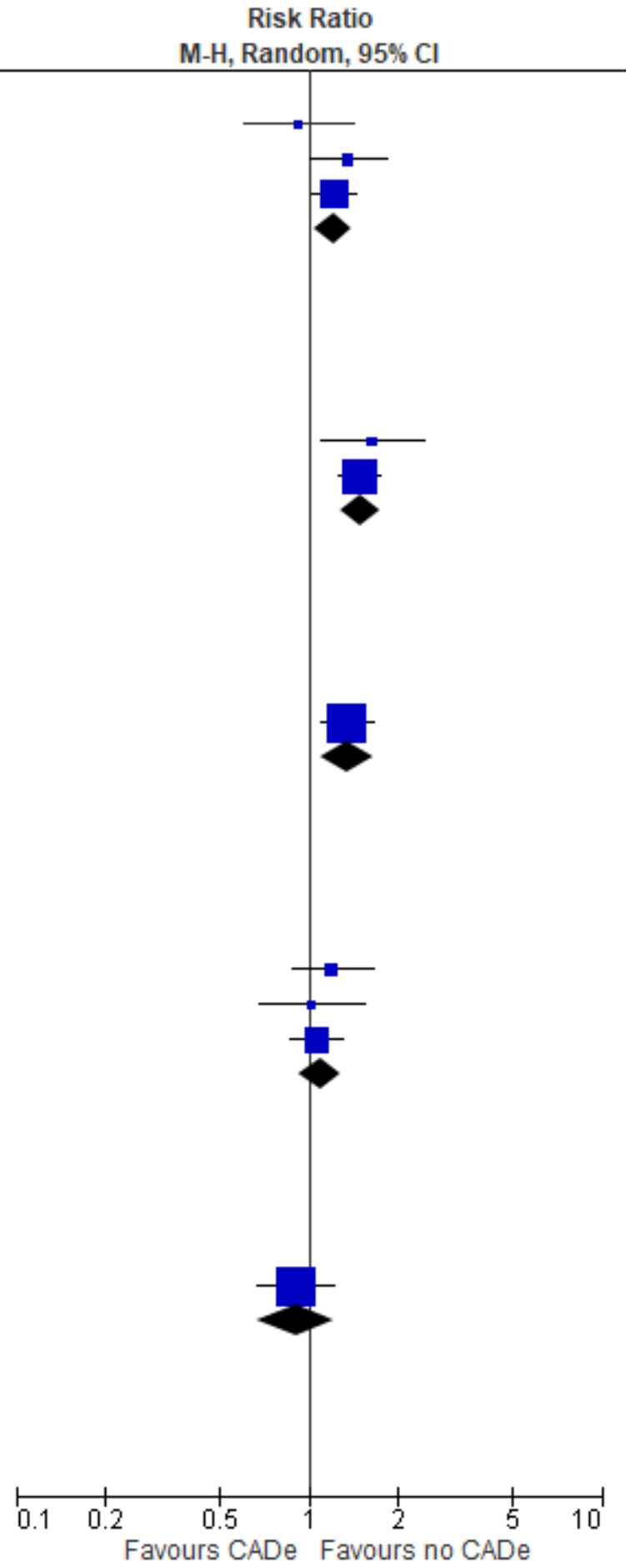

Abbreviations: CADe, computer-aided polyp detection; CI, confidence interval; M-H, Mantel-Haenszel.

2.2 Computer-aided polyp diagnosis (CADx) data tables

2.2.1 All polyps

Supplementary Table 19. Diagnostic accuracy data for adjunct CAD EYE® optical diagnosis – neoplastic vs hyperplastic

Index test	Reference standard	Sensitivity (95% CI)	Specificity (95% CI)	Accuracy (95% CI)	Comments
**Cassinotti 2023 (endoscopic surveillance of patients with ulcerative colitis) – 133 lesions assessed[29]**					
**Outcome: classification of any polyps (no details on classification categories, i.e. neoplastic vs hyperplastic)**					
CAD EYE®-assisted optical diagnosis with BLI and LCI (no further details)	Histology	83% (95% CI NR)	65% (95% CI NR)	68% (95% CI NR)	• Possible adjunct use of CAD EYE® – although criteria used in conjunction with unclear<br>• No details of whether only high confidence diagnoses included or how SSLs treated in the analysis.<br>• CAD EYE® "not inferior" to conventional Kudo and NICE classifications, but Kudo-IBD had higher sensitivity, and statistically significantly higher specificity and accuracy (p-value <0.05).<br>• NPV values were 96.0% for CAD EYE®-assisted optical
Endoscopist optical diagnosis alone (no further details)<br><br>Optical diagnosis performed using conventional Kudo, NICE and Kudo-IBD classifications	Histology	• Conventional Kudo: 67%<br>• NICE: 72%<br>• Kudo-IBD: 89%<br><br>(95% CI NR)	• Conventional Kudo: 71%<br>• NICE: 68%<br>• Kudo-IBD: 83%<br><br>(95% CI NR)	• Conventional Kudo: 71%<br>• NICE: 68%<br>• Kudo-IBD: 84%<br><br>(95% CI NR)	

					diagnosis, 93.0% for endoscopist optical diagnosis with conventional Kudo classification and 94.0% for endoscopist optical diagnosis with NICE classification (NR for endoscopist optical diagnosis with Kudo-IBD classification). 95% CIs were not reported.
**Sato 2024 (positive FIT, symptoms, screening colonoscopy or where colonoscopy otherwise required) – 380 lesions assessed[27]**					
**Outcome: classification of any polyps (neoplastic or hyperplastic categories)**					
CAD EYE®-assisted optical diagnosis<br><br>Unclear but assume based on combination of endoscopist diagnoses on WLI, magnified and non-magnified BLI with AI diagnoses on magnified and non-magnified BLI<br><br>WLI based on endoscopist experience, non-magnified BLI based on NBI International Colorectal Endoscopic classification and magnified BLI based on JNET criteria	Histology	94.3% (95% CI, 91.1 to 96.7%)	71.3% (95% CI, 60.0 to 80.8%)	89.5% (95% CI, 85.9 to 92.4%)	• SSLs considered to be hyperplastic in line with AI which is not able to determine these lesions.<br>• Excludes polyps considered by endoscopists to be whitish diminutive polyps or rectosigmoid, invasive cancer and submucosal tumours.<br>• Does not appear to limit to high-confidence diagnoses.

Endoscopist optical diagnosis alone<br><br>Reported separately when using WLI, non-magnified BLI and magnified BLI<br><br>WLI based on endoscopist experience, non-magnified BLI based on NBI International Colorectal Endoscopic classification and magnified BLI based on JNET criteria	Histology	**WLI**<br>90.0% (95% CI, 86.0 to 93.2%)<br><br>**Non-magnified BLI**<br>93.0% (95% CI, 89.5 to 95.6%)<br><br>**Magnified BLI**<br>94.3% (95% CI, 91.1 to 96.7%)	**WLI**<br>68.8% (95% CI, 57.4 to 78.7%)<br><br>**Non-magnified BLI**<br>70.0% (95% CI, 58.7 to 79.7%)<br><br>**Magnified BLI**<br>68.8% (95% CI, 57.4 to 78.7%)	**WLI**<br>85.5% (95% CI, 81.6 to 88.9%)<br><br>**Non-magnified BLI**<br>88.2% (95% CI, 84.5 to 91.2%)<br><br>**Magnified BLI**<br>88.9% (95% CI, 85.4 to 91.9%)	• NPV values were 77.0% (95% CI, 67.4 to 84.5%) for CAD EYE®-assisted optical diagnosis, and 64.7% (95% CI, 55.9 to 72.6%), 72.7% (95% CI, 63.3 to 91.2%) and 76.4% (95% CI, 66.6 to 84.0%) for endoscopist optical diagnosis using WLI, non-magnified BLI and magnified BLI, respectively.
Abbreviations: AI, artificial intelligence; BLI, blue-light imaging; CI, confidence interval; FIT, faecal immunochemical test; IBD, inflammatory bowel disease; JNET, Japan Narrow Band Imaging Expert Team; LCI, linked-colour imaging; NBI, narrow-band imaging; NICE, NBI International Colorectal Endoscopic criteria; NPV, negative predictive value; NR, not reported; SSL, sessile serrated lesion; WLI, white-light imaging.					

Supplementary Table 20. Diagnostic accuracy data for Discovery™-assisted optical diagnosis – dysplasia vs non-dysplasia

Index test	Reference standard	Sensitivity (95% CI)	Specificity (95% CI)	Accuracy (95% CI)	Comments
**Outcome: classification of any polyps into dysplasia vs non-dysplasia**					
**Lopez-Serrano 2024 (surveillance colonoscopy in consecutive ulcerative colitis patients at risk of CRC) – 48 resected polyps assessed[32]**					

Discovery™-assisted optical diagnosis<br><br>Paris and Kudo pit pattern classification (alongside Discovery™) used for optical diagnosis	Histology based on Vienna criteria	90.0% (71.0 to 100.0%)	5.0% (0.0 to 12.0%)	22.9% (NR)	• Categorised into dysplasia (low-grade dysplasia, high-grade dysplasia, invasive carcinoma and sessile serrated adenomas with dysplasia) or non-dysplasia (hyperplastic polyps, sessile serrated polyps without dysplasia, post-inflammatory polyps, scarring tissue and other nonspecific non-neoplastic mucosal changes);<br>• Does not appear to limit only to high confidence diagnoses.<br>• Unclear whether used AI system as intended, as no CADx function for Discovery™ outlined in manufacturer submission.<br>• NPV values were 67.0% (95% CI, 54.0 to 100.0%) and 83.0% (95% CI, 54.0 to 100.0%), respectively, for Discovery™-assisted optical diagnosis and virtual chromoendoscopy-assisted colonoscopy, respectively.
Virtual chromoendoscopy-assisted colonoscopy<br><br>Paris and Kudo pit pattern classification used for optical diagnosis	Histology based on Vienna criteria	90.0% (71.0 to 100.0%)	13.0% (2.0 to 24.0%)	29.2% (NR)	
Abbreviations: CI, confidence interval; CRC, colorectal cancer; NPV, negative predictive value; NR, not reported.					

Supplementary Table 21. Diagnostic accuracy data for GI Genius™-assisted optical diagnosis – adenoma vs non-adenoma

Index test	Reference standard	Sensitivity (95% CI)	Specificity (95% CI)	Accuracy (95% CI)	Comments
**Outcome: classification of colorectal polyps of any size into adenomatous or non-adenomatous**					
**Hassan 2022 (colonoscopy for primary CRC screening, post-polypectomy surveillance, positive FIT test or for symptoms/signs) – 544 polyps assessed[61]**					

GI Genius™-assisted optical diagnosis<br>Adenomatous vs non-adenomatous<br><br>Unclear which criteria used for optical diagnosis alongside GI Genius™<br><br>Expert endoscopists included (>2000 screening colonoscopies, trained in optical diagnosis and prior studies on polyp characterisation with BLI)	Histopathology	80% (73.7 to 85.3%)	93.1% (89.8 to 95.6%)	88.1% (85.0 to 90.7%)	• SSLs considered to be non-adenomatous in the analysis;<br>• High and low confidence diagnoses included;<br>• Does not appear to have excluded those where AI could not make a prediction.<br>• NPV value was 88.2% (95% CI, 84.3 to 91.4%).
Abbreviations: AI, artificial intelligence; BLI, blue-light imaging; CI, confidence interval; CRC, colorectal cancer; FIT, faecal immunochemical test; NPV, negative predictive value; SSL, sessile serrated lesion.					

2.2.2 All diminutive polyps

Supplementary Table 22. Diagnostic accuracy data for CAD EYE®-assisted optical diagnosis in diminutive polyps – adenoma, hyperplastic and serrated histologies

Index test	Reference standard	Sensitivity (95% CI)	Specificity (95% CI)	Accuracy (95% CI)	Comments
**Outcome: classification of diminutive (≤5 mm) polyps of any location into adenoma, hyperplastic and serrated histologies**					
**Djinbachian 2024 (colonoscopy for screening, surveillance or diagnostic purposes) – 179 polyps assessed[25]**					
CAD EYE®-assisted optical diagnosis (all prior experience with optical diagnosis, experience varied)<br><br>Unclear which criteria used for optical diagnosis	Histopathology	83.6% (75.4 to 90.0%)	63.8% (51.3 to 75.0%)	72.1% (95% CI, 65.5 to 78.6%)	• Main analysis does not limit to high-confidence diagnoses only;<br>• The main analysis also allows for the classification of SSLs as its own group;<br>• Analysed all polyps ≤5 mm that were resected and retrieved for histopathology, were not normal mucosa/inflammatory polyps and where optical diagnosis was performed.<br>• NPV value was 71.0% (95% CI, 58.1 to 81.8%).
**Outcome: classification of diminutive (≤5 mm) polyps of any location that underwent a resect-and-discard or diagnose-and-leave strategy into adenomatous or non-adenomatous categories**					

**Taghiakbari 2025 (outpatient colonoscopy – no further details) – 138 polyps assessed[28]**					
CAD EYE®-assisted optical diagnosis<br><br>Unclear which criteria used for optical diagnosis	Video review by 3 expert endoscopists (as polyps not sent for histology in this study)	89.9% (95% CI, 81.0 to 95.5%)	89.8% (95% CI, 79.2 to 96.2%)	89.9% (95% CI, 83.6 to 94.3%)	• Analysis limited to high-confidence diagnoses;<br>• SSLs or suspected SSLs not captured as these were supposed to be sent for histology;<br>• Reference standard less than ideal as video review by experts rather than histology;<br>• Analysed those polyps that were not sent for histology but where resect-and-discard or diagnose-and-leave approaches were implemented.<br>• NPV value was 86.9% (95% CI, 77.4 to 92.8%).
**Outcome: classification of diminutive (≤5 mm) polyps of any location that underwent a diagnose-and-leave strategy into adenomatous or non-adenomatous categories**					
**Taghiakbari 2025 (outpatient colonoscopy – no further details) – 40 polyps assessed[28]**					
CAD EYE®-assisted optical diagnosis<br><br>Unclear which criteria used for optical diagnosis	Video review by 3 expert endoscopists (as polyps not sent for histology in this study)	25.0% (95% CI, 0.63 to 80.6%)	100.0% (95% CI, 90.3 to 100.0%)	92.5% (95% CI, 79.6 to 98.4%)	• Analysis limited to high-confidence diagnoses;<br>• SSLs or suspected SSLs not captured as these were supposed to be sent for histology;<br>• Reference standard less than ideal as video review by experts rather than histology;<br>• Analysed those polyps that were not sent for histology but where diagnose-and-leave approach was implemented.<br>• NPV value was 92.3% (95% CI, 87.2 to 95.5%).
**Outcome: classification of diminutive (≤5 mm) polyps of any location that underwent a resect-and-discard strategy into adenomatous or non-adenomatous categories**					
**Taghiakbari 2025 (outpatient colonoscopy – no further details) – 98 polyps assessed[28]**					
CAD EYE®-assisted optical diagnosis<br><br>Unclear which criteria used for optical diagnosis	Video review by 3 expert endoscopists (as polyps not sent for histology in this study)	93.3% (95% CI, 85.1 to 97.8%)	73.9% (95% CI, 51.6 to 89.8%)	88.8% (95% CI, 80.8 to 94.3%)	• Analysis limited to high-confidence diagnoses;<br>• SSLs or suspected SSLs not captured as these were supposed to be sent for histology;<br>• Reference standard less than ideal as video review by experts rather than histology;

					• Analysed those polyps that were not sent for histology but where resect-and-discard approach was implemented.<br>• NPV value was 77.3% (95% CI, 58.5 to 89.1%).
Abbreviations: CI, confidence interval; NPV, negative predictive value; SSL, sessile serrated lesion.					

Supplementary Table 23. Diagnostic accuracy data for GI Genius™-assisted optical diagnosis in diminutive polyps – adenomatous vs non-adenomatous

Index test	Reference standard	Sensitivity (95% CI)	Specificity (95% CI)	Accuracy (95% CI)	Comments
**Outcome: classification of diminutive (≤5 mm) colorectal polyps into adenomatous or non-adenomatous**					
**Hassan 2022 (colonoscopy for primary CRC screening, post-polypectomy surveillance, positive FIT test or for symptoms/signs) – 476 polyps assessed[61]**					
GI Genius™-assisted optical diagnosis<br>Adenomatous vs non-adenomatous<br><br>Unclear which criteria used for optical diagnosis alongside GI Genius™<br><br>Expert endoscopists included (>2000 screening colonoscopies, trained in optical diagnosis and prior studies on polyp characterisation with BLI)	Histopathology	78.6% (71.7% to 84.5%)	94.0% (90.7% to 96.4%)	88.4% (85.2% to 91.1%)	• SSLs considered to be non-adenomatous in the analysis;<br>• High and low confidence diagnoses included;<br>• Does not appear to have excluded those where AI could not make a prediction.<br>• NPV value was 88.4% (95% CI, 84.4 to 91.7%).

**Rondonotti 2024 (colonoscopy for screening, symptoms or post-polypectomy surveillance with at least one diminutive colorectal polyp) – 376 polyps assessed[60]**					
GI Genius™-assisted optical diagnosis<br>Adenomatous vs non-adenomatous<br><br>Unclear which criteria used for optical diagnosis alongside GI Genius™<br><br>Experts and non-experts included (experts had training, prior studies of optical diagnosis, auditing and monitoring and performed on regular basis according to ESGE criteria)	Histopathology	94.8% (91.1 to 97.1%)	58.9% (49.7 to 67.5%)	83.0% (78.8 to 86.6%)	• SSLs considered to be non-adenomatous in the analysis;<br>• Only high confidence diagnoses included in analysis;<br>• Does not appear to have excluded those where AI could not make a prediction.<br>• NPV value was 84.9% (95% CI, 75.2 to 91.4%).
Abbreviations: AI, artificial intelligence; BLI, blue-light imaging; CI, confidence interval; CRC, colorectal cancer; ESGE, European Society of Gastrointestinal Endoscopy; FIT, faecal immunochemical test; NPV, negative predictive value; SSL, sessile serrated lesion.					

2.2.3 Diminutive polyps separated into rectosigmoid and non-rectosigmoid location

Supplementary Table 24. Diagnostic accuracy data for CAD EYE®-assisted optical diagnosis in diminutive polyps separated by rectosigmoid and non-rectosigmoid location

Index test	Reference standard	Sensitivity (95% CI)	Specificity (95% CI)	Accuracy (95% CI)	Comments
**Outcome: classification of diminutive (≤5 mm) rectosigmoid polyps into adenoma, hyperplastic and serrated histologies**					
**Djinbachian 2024 (colonoscopy for screening, surveillance or diagnostic purposes) – unclear number of polyps assessed[25]**					
CAD EYE®-assisted optical diagnosis (all prior experience with optical diagnosis, experience varied)<br><br>Unclear which criteria used for optical diagnosis	Histopathology	100.0% (83.9 to 100.0)	71.4% (55.4 to 84.3)	NR	• Main analysis does not limit to high-confidence diagnoses;<br>• Main analysis allows for the classification of SSLs as own group;<br>• Analysed all rectosigmoid polyps ≤5 mm that were resected and retrieved for histopathology, were not normal mucosa/inflammatory polyps and where optical diagnosis was performed.

					• NPV value was 100.0% (95% CI, 90.7 to 100.0%).
**Outcome: classification of diminutive (≤5 mm) rectosigmoid polyps into adenomatous vs non-adenomatous**					
**Rondonotti 2023 (aged 18-85 years undergoing outpatient colonoscopy – no further details) – 550 (CAD EYE®) or 540 (endoscopist) polyps assessed**<br>**High-confidence endoscopist diagnoses only[26]**					
CAD EYE®-assisted optical diagnosis (expert and non-expert endoscopists)<br><br>Optical diagnosis based on CAD EYE® and BASIC criteria	Histopathology using Vienna classification	88.6% (83.7 to 91.4%)	88.1% (83.9 to 91.4%)	88.4% (85.3 to 90.9%)	• Non-adenomatous on histology included hyperplastic polyps, SSLs, inflammatory polyps or normal mucosal samples;<br>• Only analysed diminutive rectosigmoid polyps that could be retrieved and where high-confidence optical diagnosis could be made by the endoscopist.<br>• NPV values were 91.0% (95% CI, 87.1 to 93.9%) and 90.9% (95% CI, 86.8 to 93.7%) for CAD EYE®-assisted optical diagnosis and endoscopist optical diagnosis, respectively.
Endoscopist optical diagnosis only (expert and non-expert endoscopists)<br><br>Optical diagnosis based on BASIC criteria	Histopathology using Vienna classification	88.6 % (83.6 to 92.2%)	88.8% (84.5 to 91.9%)	88.7% (85.7 to 91.2%)	
**Rondonotti 2023 (aged 18-85 years undergoing outpatient colonoscopy – no further details) – unclear number of polyps assessed**<br>**Low-confidence endoscopist diagnoses only[26]**					
CAD EYE®-assisted optical diagnosis<br><br>Optical diagnosis based on CAD EYE® and BASIC criteria	Histopathology using Vienna classification	NR	NR	50.0% (45.3% to 74.9%)	• Non-adenomatous on histology included hyperplastic polyps, SSLs, inflammatory polyps or normal mucosal samples;<br>• Only analysed diminutive rectosigmoid polyps that could be retrieved and where low-confidence optical diagnosis was made by the endoscopist;<br>• No data reported for endoscopist optical diagnosis only assessment.<br>• NPV value was 70.6% (95% CI, 44.0 to 87.1%).
**Outcome: classification of diminutive (≤5 mm) non-rectosigmoid polyps into adenomatous vs non-adenomatous**					
**Rondonotti 2023 (aged 18-85 years undergoing outpatient colonoscopy – no further details) – unclear number of polyps assessed**<br>**High-confidence endoscopist diagnoses only[26]**					
CAD EYE®-assisted optical diagnosis<br><br>Optical diagnosis based on CAD EYE® and BASIC criteria	Histopathology using Vienna classification	NR	66.7% (53.5% to 69.6%)	87.0% (86.0% to 92.9%)	• Non-adenomatous on histology included hyperplastic polyps, SSLs, inflammatory polyps or normal mucosal samples;

					• Only analysed diminutive non-rectosigmoid polyps that could be retrieved and where high-confidence optical diagnosis could be made by the endoscopist;<br>• Non-rectosigmoid polyps defined as those proximal to the rectosigmoid tract;<br>• No data reported for endoscopist optical diagnosis only assessment.<br>• NPV value was 72.4 % (95% CI, 58.8 to 82.9%).
Abbreviations: CI, confidence interval; NPV, negative predictive value; NR, not reported; SSL, sessile serrated lesion.					

Supplementary Table 25. Diagnostic accuracy data for GI Genius™-assisted optical diagnosis in diminutive rectosigmoid polyps

Index test	Reference standard	Sensitivity (95% CI)	Specificity (95% CI)	Accuracy (95% CI)	Comments
**Outcome: classification of diminutive (≤5 mm) rectosigmoid colorectal polyps into adenomatous or non-adenomatous**					
**Hassan 2022 (colonoscopy for primary CRC screening, post-polypectomy surveillance, positive FIT test or for symptoms/signs) – 279 polyps assessed[61]**					
GI Genius™-assisted optical diagnosis - adenomatous vs non-adenomatous<br><br>Unclear which criteria used for optical diagnosis alongside GI Genius™<br><br>Expert endoscopists included (>2000 screening colonoscopies, trained in optical diagnosis and prior studies on polyp characterisation with BLI)	Histopathology	81. 2% (63.5 to 82.8%)	98.0% (95.3 to 99.3%)	96.1% (93.1 to 98%)	• SSLs considered to be non-adenomatous in the analysis;<br>• Only high confidence diagnoses included in analysis;<br>• Does not appear to have excluded those where AI could not make a prediction.<br>• NPV value was 97.6% (95% CI, 94.8 to 99.1%).

**Outcome: correct classification of non-adenomatous histology in diminutive rectosigmoid polyps**					
**Bernhofer 2025 (elective colonoscopy for any reason performed by a trainee endoscopist) – 223 polyps[59]**					
GI Genius™-assisted optical diagnosis - adenomatous vs non-adenomatous<br><br>Optical diagnosis based on NICE and JNET classifications with GI Genius assistance, NBI used<br><br>Trainee endoscopists with no formal optical diagnosis training prior to the study, with experts (>2000 colonoscopies) able to support with polypectomies	Histopathology	NR	NR	NR	• Only NPV reported: 90.3% (95% CI, 85.0 to 94.0%);<br>• Unclear how SSLs treated in analysis;<br>• Does not appear to limit to high confidence diagnoses;<br>• PIVI 2 requirement for diagnose-and-leave strategy states that non-neoplastic DRSPs can be left without resection and requires NPV of at least 90% for adenomatous histology.
Abbreviations: AI, artificial intelligence; BLI, blue-light imaging; CI, confidence interval; CRC, colorectal cancer; DRSPs, diminutive rectosigmoid polyps; FIT, faecal immunochemical test; JNET, Japan Narrow Band Imaging Expert Team; NBI, narrow-band imaging; NICE, NBI International Colorectal Endoscopic criteria; NPV, negative predictive value; NR, not reported; PIVI 2, Preservation and Incorporation of Valuable Endoscopic Innovations – diagnose-and-leave strategy; SSL, sessile serrated lesion.					

2.2.4 Diminutive polyps separated into proximal and distal location

Supplementary Table 26. Diagnostic accuracy data for GI Genius™-assisted optical diagnosis in diminutive distal and proximal polyps

Index test	Reference standard	Sensitivity (95% CI)	Specificity (95% CI)	Accuracy (95% CI)	Comments
**Outcome: classification of diminutive (≤5 mm) distal colorectal polyps into adenomatous or non-adenomatous**					
**Rondonotti 2024 (colonoscopy for screening, symptoms or post-polypectomy surveillance with at least one diminutive colorectal polyp) – 163 polyps assessed[60]**					

GI Genius™-assisted optical diagnosis - adenomatous vs non-adenomatous<br><br>Unclear which criteria used for optical diagnosis alongside GI Genius™<br><br>Experts and non-experts included (experts had training, prior studies of optical diagnosis, auditing and monitoring and performed on regular basis according to ESGE criteria)	Histopathology	91.4% (83.3 to 95.9%)	64.3% (51.9 to 75.1%)	79.8% (72.8 to 85.6%)	• SSLs considered to be non-adenomatous in the analysis;<br>• Only high confidence diagnoses included in analysis;<br>• Does not appear to have excluded those where AI could not make a prediction.<br>• NPV value was 84.9% (95% CI, 71.9 to 92.8%).
**Outcome: classification of diminutive (≤5 mm) proximal colorectal polyps into adenomatous or non-adenomatous**					
**Rondonotti 2024 (colonoscopy for screening, symptoms or post-polypectomy surveillance with at least one diminutive colorectal polyp) – 213 polyps assessed[60]**					
GI Genius™-assisted optical diagnosis - adenomatous vs non-adenomatous<br><br>Unclear which criteria used for optical diagnosis alongside GI Genius™<br><br>Experts and non-experts included (experts had training, prior studies of optical diagnosis, auditing and monitoring and performed on regular basis according to ESGE criteria)	Histopathology	96.9% (92.4 to 98.8%)	51.9% (38.0 to 65.5%)	85.5% (80.0 to 89.9%)	• SSLs considered to be non-adenomatous in the analysis;<br>• Only high confidence diagnoses included in analysis;<br>• Does not appear to have excluded those where AI could not make a prediction.<br>• NPV value was 84.8% (95% CI, 80.0 to 89.9%).
Abbreviations: AI, artificial intelligence; CI, confidence interval; ESGE, European Society of Gastrointestinal Endoscopy; NPV, negative predictive value; SSL, sessile serrated lesion.					

2.3 Procedural duration outcomes

Supplementary Figure 16. Meta-analyses of withdrawal time

Study or Subgroup	CADe Mean	CADe SD	CADe Total	No CADe Mean	No CADe SD	No CADe Total	Weight	Mean Difference IV, Random, 95% CI
**12.17.1 CAD EYE® - polypectomies etc. excluded or unclear**								
Alali 2025	9.04	2.06	51	8.49	1.74	51	5.8%	0.55 [-0.19, 1.29]
Aniwan 2023	9.2	5.35	620	8.85	4.33	625	10.8%	0.35 [-0.19, 0.89]
Desai 2024	11.28	4.59	509	10.8	4.81	522	9.6%	0.48 [-0.09, 1.05]
Huneburg 2023	15	3.7	50	14	3.5	46	1.5%	1.00 [-0.44, 2.44]
Miyaguchi 2024	7	2.0347	400	7	2.0347	400	39.9%	0.00 [-0.28, 0.28]
Nakashima 2023	6.86	1.71	207	6.65	1.74	208	28.8%	0.21 [-0.12, 0.54]
Yamaguchi 2024	13.06	3.23	113	13.05	4.01	118	3.6%	0.01 [-0.93, 0.95]
**Subtotal (95% CI)**			**1950**			**1970**	**100.0%**	**0.19 [0.01, 0.37]**
Heterogeneity: Tau² = 0.00; Chi² = 5.34, df = 6 (P = 0.50); I² = 0%								
Test for overall effect: Z = 2.11 (P = 0.03)								
**12.17.2 ENDO-AID™ - polypectomies etc. excluded or unclear**								
Gimeno-Garcia 2023	7.97	1.36	155	7.81	1.26	157	46.1%	0.16 [-0.13, 0.45]
Lau 2024	14.94	8.08	386	13.74	8.66	380	6.3%	1.20 [0.01, 2.39]
Lui 2024	8.81	5.08	468	8.4	2.9	214	19.8%	0.41 [-0.19, 1.01]
Spada 2025	9.8	4.14	611	9.86	4.31	617	27.8%	-0.06 [-0.53, 0.41]
**Subtotal (95% CI)**			**1620**			**1368**	**100.0%**	**0.21 [-0.10, 0.52]**
Heterogeneity: Tau² = 0.03; Chi² = 4.41, df = 3 (P = 0.22); I² = 32%								
Test for overall effect: Z = 1.35 (P = 0.18)								
**12.17.3 ENDOANGEL® - polypectomies etc. excluded**								
Yao 2022	9.94	3.93	268	9.36	4.09	271	44.8%	0.58 [-0.10, 1.26]
Yao 2024	7.35	3.41	227	7.32	2.9	229	55.2%	0.03 [-0.55, 0.61]
**Subtotal (95% CI)**			**495**			**500**	**100.0%**	**0.28 [-0.26, 0.81]**
Heterogeneity: Tau² = 0.05; Chi² = 1.46, df = 1 (P = 0.23); I² = 31%								
Test for overall effect: Z = 1.01 (P = 0.31)								
**12.17.4 ENDOANGEL® - polypectomies etc. included**								
Yao 2022	10.52	4.24	268	9.71	4.13	271	100.0%	0.81 [0.10, 1.52]
**Subtotal (95% CI)**			**268**			**271**	**100.0%**	**0.81 [0.10, 1.52]**
Heterogeneity: Not applicable								
Test for overall effect: Z = 2.25 (P = 0.02)								
**12.17.5 EndoScreener® - biopsies excluded**								
Liu 2020	6.71	1.63	393	6.62	1.22	397	17.9%	0.09 [-0.11, 0.29]
Wang 2019	6.18	1.38	522	6.07	1.11	536	31.7%	0.11 [-0.04, 0.26]
Wang 2020 (effect.)	6.48	1.32	484	6.37	1.09	478	31.0%	0.11 [-0.04, 0.26]
Wang 2023	7.06	1.59	636	6.87	1.9	625	19.3%	0.19 [-0.00, 0.38]
**Subtotal (95% CI)**			**2035**			**2036**	**100.0%**	**0.12 [0.04, 0.21]**
Heterogeneity: Tau² = 0.00; Chi² = 0.62, df = 3 (P = 0.89); I² = 0%								
Test for overall effect: Z = 2.81 (P = 0.005)								
**12.17.6 EndoScreener® - biopsies included**								
Liu 2020	7.29	1.98	393	6.94	1.53	397	21.4%	0.35 [0.10, 0.60]
Wang 2019	6.89	1.79	522	6.39	1.21	536	38.3%	0.50 [0.32, 0.68]
Wang 2020 (effect.)	7.46	2.02	484	6.99	1.57	478	25.0%	0.47 [0.24, 0.70]
Wang 2023	8.2	2.77	636	7.67	2.53	625	15.2%	0.53 [0.24, 0.82]
**Subtotal (95% CI)**			**2035**			**2036**	**100.0%**	**0.46 [0.35, 0.58]**
Heterogeneity: Tau² = 0.00; Chi² = 1.16, df = 3 (P = 0.76); I² = 0%								
Test for overall effect: Z = 7.97 (P < 0.00001)								
**12.17.7 GI Genius™ - mixed inclusion/exclusion of polypectomies etc.**								
Mangas-Sanjuan 2023	16.9	10.3	1610	15.7	9.4	1603	22.0%	1.20 [0.52, 1.88]
Repici 2022	8.15	1.6	330	7.98	1.5	330	37.4%	0.17 [-0.07, 0.41]
Seager 2024 - whole study	9.86	4.1964	313	9.36	4.1964	402	24.0%	0.50 [-0.12, 1.12]
Wallace 2022	9.926	4.968	229	9.525	4.773	230	16.6%	0.40 [-0.49, 1.29]
**Subtotal (95% CI)**			**2482**			**2565**	**100.0%**	**0.51 [0.05, 0.98]**
Heterogeneity: Tau² = 0.14; Chi² = 8.29, df = 3 (P = 0.04); I² = 64%								
Test for overall effect: Z = 2.15 (P = 0.03)								

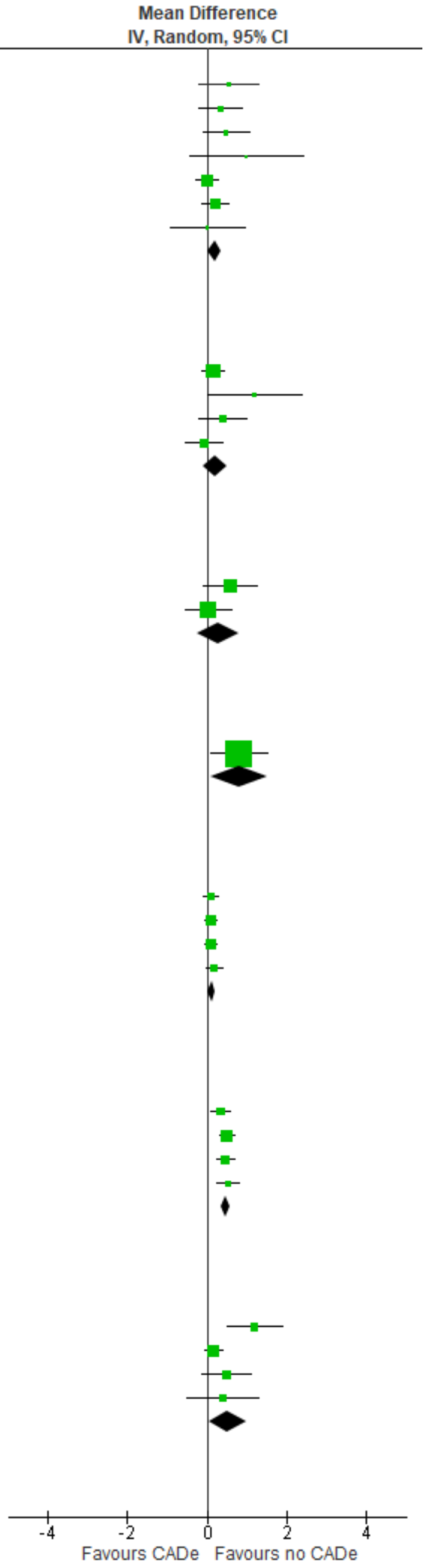

Note: additional data were available from some studies as medians with or without IQRs, which could not be included in the above meta-analyses, but generally supported the conclusions made in the main publication, including data for Discovery™ and MAGENTIQ-COLO™ systems.

Abbreviations: CADe, computer-aided polyp detection; CI, confidence interval; IQR, interquartile range; IV, inverse variance; SD, standard deviation.

Supplementary Figure 17. Meta-analyses of total procedure duration

Study or Subgroup	CADe Mean	CADe SD	CADe Total	No CADe Mean	No CADe SD	No CADe Total	Weight	Mean Difference IV, Random, 95% CI
**12.18.1 CAD EYE® - assume nothing excluded**								
Desai 2024	19.9	8.8	509	19.2	10.5	522	78.1%	0.70 [-0.48, 1.88]
Huneburg 2023	22	5.3	50	21.1	5.8	46	21.9%	0.90 [-1.33, 3.13]
**Subtotal (95% CI)**			**559**			**568**	**100.0%**	**0.74 [-0.30, 1.79]**
Heterogeneity: Tau² = 0.00; Chi² = 0.02, df = 1 (P = 0.88); I² = 0%								
Test for overall effect: Z = 1.40 (P = 0.16)								
**12.18.6 EndoScreener® - assume nothing excluded**								
Liu 2020	13.37	5.15	393	13.01	4.64	397	35.8%	0.36 [-0.32, 1.04]
Wang 2019	12.52	4.38	522	12.1	4.08	536	64.2%	0.42 [-0.09, 0.93]
**Subtotal (95% CI)**			**915**			**933**	**100.0%**	**0.40 [-0.01, 0.81]**
Heterogeneity: Tau² = 0.00; Chi² = 0.02, df = 1 (P = 0.89); I² = 0%								
Test for overall effect: Z = 1.91 (P = 0.06)								
**12.18.7 GI Genius™ - specific to those with no polyps removed**								
Seager 2024 - whole study	23.97	9.3723	315	22.5	9.3723	405	100.0%	1.47 [0.09, 2.85]
**Subtotal (95% CI)**			**315**			**405**	**100.0%**	**1.47 [0.09, 2.85]**
Heterogeneity: Not applicable								
Test for overall effect: Z = 2.09 (P = 0.04)								

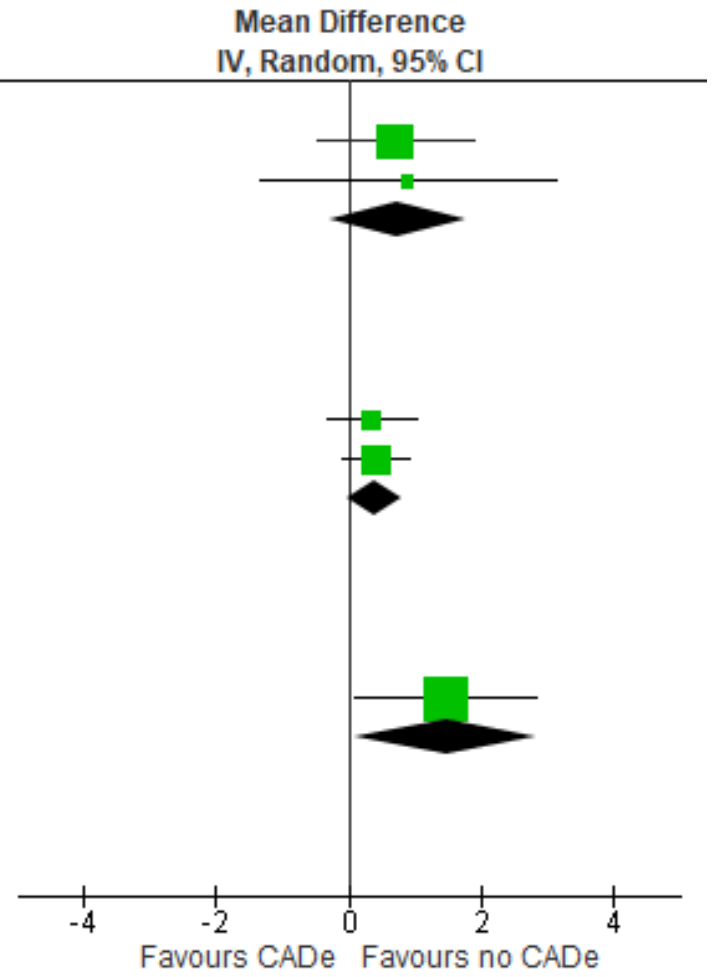

Note: additional data were available from some studies as medians with or without IQRs, which could not be included in the above meta-analyses, but generally supported the conclusions made in the main publication, including data for the Discovery™ system.

Abbreviations: CADe, computer-aided polyp detection; CI, confidence interval; IQR, interquartile range; IV, inverse variance; SD, standard deviation.

2.4 Adverse events

Supplementary Figure 18. Meta-analyses of adverse events

Study or Subgroup	CADe Events	CADe Total	No CADe Events	No CADe Total	Weight	Risk Difference M-H, Random, 95% CI
**12.19.1 Argus®**						
Strapko 2023 (abstract only)	0	344	0	342	100.0%	0.00 [-0.01, 0.01]
**Subtotal (95% CI)**		**344**		**342**	**100.0%**	**0.00 [-0.01, 0.01]**
Total events	0		0			
Heterogeneity: Not applicable						
Test for overall effect: Z = 0.00 (P = 1.00)						
**12.19.2 CAD EYE®**						
Alali 2025	0	51	0	51	0.2%	0.00 [-0.04, 0.04]
Aniwan 2023	0	620	0	625	26.5%	0.00 [-0.00, 0.00]
Desai 2024	5	509	3	522	2.3%	0.00 [-0.01, 0.01]
Djinbachian 2024	0	229	0	238	3.7%	0.00 [-0.01, 0.01]
Huneburg 2023	0	50	0	46	0.2%	0.00 [-0.04, 0.04]
Miyaguchi 2024	0	400	0	400	11.0%	0.00 [-0.00, 0.00]
Tiankanon 2024	0	400	0	400	11.0%	0.00 [-0.00, 0.00]
Zimmermann-Fraedrich 2025	0	812	0	815	45.2%	0.00 [-0.00, 0.00]
**Subtotal (95% CI)**		**3071**		**3097**	**100.0%**	**0.00 [-0.00, 0.00]**
Total events	5		3			
Heterogeneity: Tau² = 0.00; Chi² = 1.05, df = 7 (P = 0.99); I² = 0%						
Test for overall effect: Z = 0.11 (P = 0.91)						
**12.19.3 ENDO-AID™**						
Lau 2024	1	386	2	380	11.4%	-0.00 [-0.01, 0.01]
Spada 2025	0	611	0	617	88.6%	0.00 [-0.00, 0.00]
**Subtotal (95% CI)**		**997**		**997**	**100.0%**	**-0.00 [-0.00, 0.00]**
Total events	1		2			
Heterogeneity: Tau² = 0.00; Chi² = 0.53, df = 1 (P = 0.47); I² = 0%						
Test for overall effect: Z = 0.20 (P = 0.84)						
**12.19.4 ENDOANGEL®**						
Yao 2022	0	268	0	271	100.0%	0.00 [-0.01, 0.01]
**Subtotal (95% CI)**		**268**		**271**	**100.0%**	**0.00 [-0.01, 0.01]**
Total events	0		0			
Heterogeneity: Not applicable						
Test for overall effect: Z = 0.00 (P = 1.00)						
**12.19.5 EndoScreener®**						
Glissen Brown 2022	0	113	0	110	1.1%	0.00 [-0.02, 0.02]
Liu 2020	0	393	0	397	14.1%	0.00 [-0.00, 0.00]
Wang 2019	0	522	0	536	25.2%	0.00 [-0.00, 0.00]
Wang 2020 (effect..)	0	484	0	478	20.8%	0.00 [-0.00, 0.00]
Wang 2020 (lower..)	0	184	0	185	3.1%	0.00 [-0.01, 0.01]
Wang 2023	0	636	0	625	35.7%	0.00 [-0.00, 0.00]
**Subtotal (95% CI)**		**2332**		**2331**	**100.0%**	**0.00 [-0.00, 0.00]**
Total events	0		0			
Heterogeneity: Tau² = 0.00; Chi² = 0.00, df = 5 (P = 1.00); I² = 0%						
Test for overall effect: Z = 0.00 (P = 1.00)						
**12.19.6 GI Genius™**						
Ahmad 2023	0	308	0	306	14.1%	0.00 [-0.01, 0.01]
Karsenti 2023	1	1003	0	1012	75.2%	0.00 [-0.00, 0.00]
Ortiz 2024	0	214	0	216	6.9%	0.00 [-0.01, 0.01]
Seager 2024	25	1003	19	1012	3.5%	0.01 [-0.01, 0.02]
Wallace 2022	4	118	5	127	0.3%	-0.01 [-0.05, 0.04]
**Subtotal (95% CI)**		**2646**		**2673**	**100.0%**	**0.00 [-0.00, 0.00]**
Total events	30		24			
Heterogeneity: Tau² = 0.00; Chi² = 2.37, df = 4 (P = 0.67); I² = 0%						
Test for overall effect: Z = 0.78 (P = 0.44)						

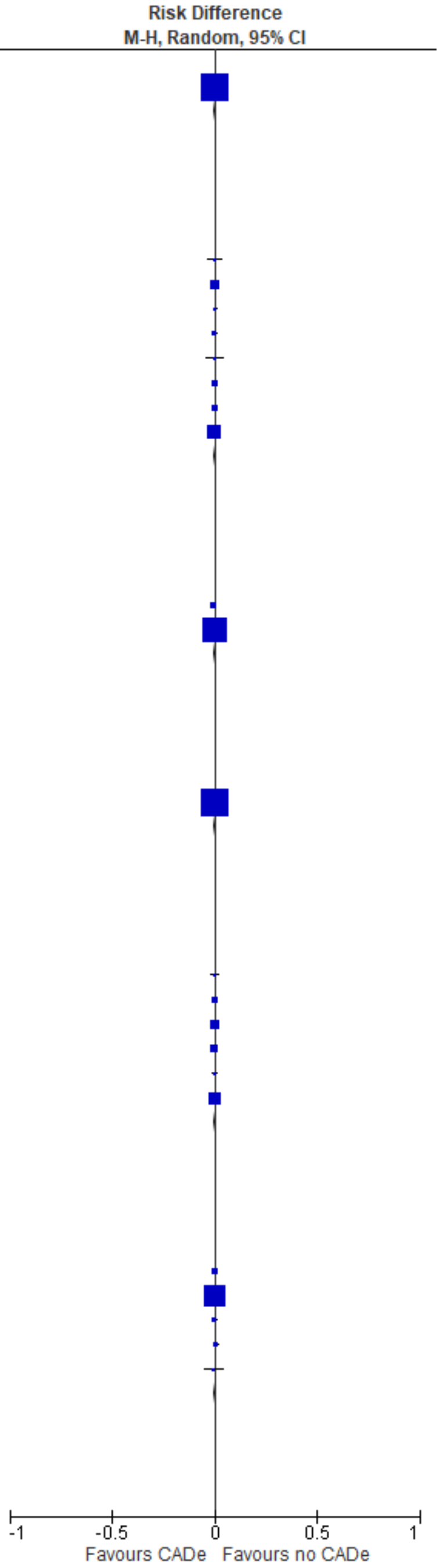

Abbreviations: CADe, computer-aided polyp detection; CI, confidence interval; M-H, Mantel-Haenszel.

2.5 Colonoscopy indication subgroup analyses

2.5.1 CAD EYE®

Supplementary Figure 19. ADR colonoscopy indication subgroup analysis with CAD EYE®-majority in whole studies and within-trial subgroup data – granular screening categories

Study or Subgroup	CAD EYE Events	CAD EYE Total	No CAD EYE Events	No CAD EYE Total	Weight	Risk Ratio M-H, Random, 95% CI
**22.1.1 Majority FIT+ screening**						
Miyaguchi 2024 - FIT-positive	50	83	33	82	14.7%	1.50 [1.09, 2.05]
Nakashima 2023 - whole study	123	207	99	208	27.8%	1.25 [1.04, 1.50]
Rondonotti 2022 - whole study	217	405	179	395	33.5%	1.18 [1.03, 1.36]
Yamaguchi 2024 - whole study	66	113	72	118	24.0%	0.96 [0.77, 1.18]
**Subtotal (95% CI)**		**808**		**803**	**100.0%**	**1.18 [1.02, 1.37]**
Total events	456		383			
Heterogeneity: Tau² = 0.01; Chi² = 6.30, df = 3 (P = 0.10); I² = 52%						
Test for overall effect: Z = 2.25 (P = 0.02)						
**22.1.2 Majority other screening or any screening combined**						
Alali 2025 - whole study	24	51	19	51	2.2%	1.26 [0.80, 2.00]
Aniwan 2023 - whole study	344	620	300	625	34.9%	1.16 [1.04, 1.29]
Desai 2024 - screening	101	254	108	286	9.9%	1.05 [0.85, 1.30]
Hiratsuka 2025 - whole study	36	48	33	46	7.6%	1.05 [0.82, 1.33]
Tiankanon 2024 - whole study	200	400	153	400	17.4%	1.31 [1.12, 1.53]
Zimmermann-Fraedrich 2025 - whole study	325	812	306	815	28.0%	1.07 [0.94, 1.20]
**Subtotal (95% CI)**		**2185**		**2223**	**100.0%**	**1.14 [1.06, 1.22]**
Total events	1030		919			
Heterogeneity: Tau² = 0.00; Chi² = 5.29, df = 5 (P = 0.38); I² = 6%						
Test for overall effect: Z = 3.70 (P = 0.0002)						
**22.1.4 Majority Lynch Syndrome surveillance**						
Huneburg 2023 - whole study	18	50	12	46	100.0%	1.38 [0.75, 2.54]
**Subtotal (95% CI)**		**50**		**46**	**100.0%**	**1.38 [0.75, 2.54]**
Total events	18		12			
Heterogeneity: Not applicable						
Test for overall effect: Z = 1.03 (P = 0.30)						
**22.1.6 Majority other surveillance**						
Desai 2024 - surveillance	137	255	116	236	56.3%	1.09 [0.92, 1.30]
Djinbachian 2024 - whole study	113	229	91	238	43.7%	1.29 [1.05, 1.59]
**Subtotal (95% CI)**		**484**		**474**	**100.0%**	**1.18 [1.00, 1.38]**
Total events	250		207			
Heterogeneity: Tau² = 0.00; Chi² = 1.46, df = 1 (P = 0.23); I² = 31%						
Test for overall effect: Z = 1.95 (P = 0.05)						

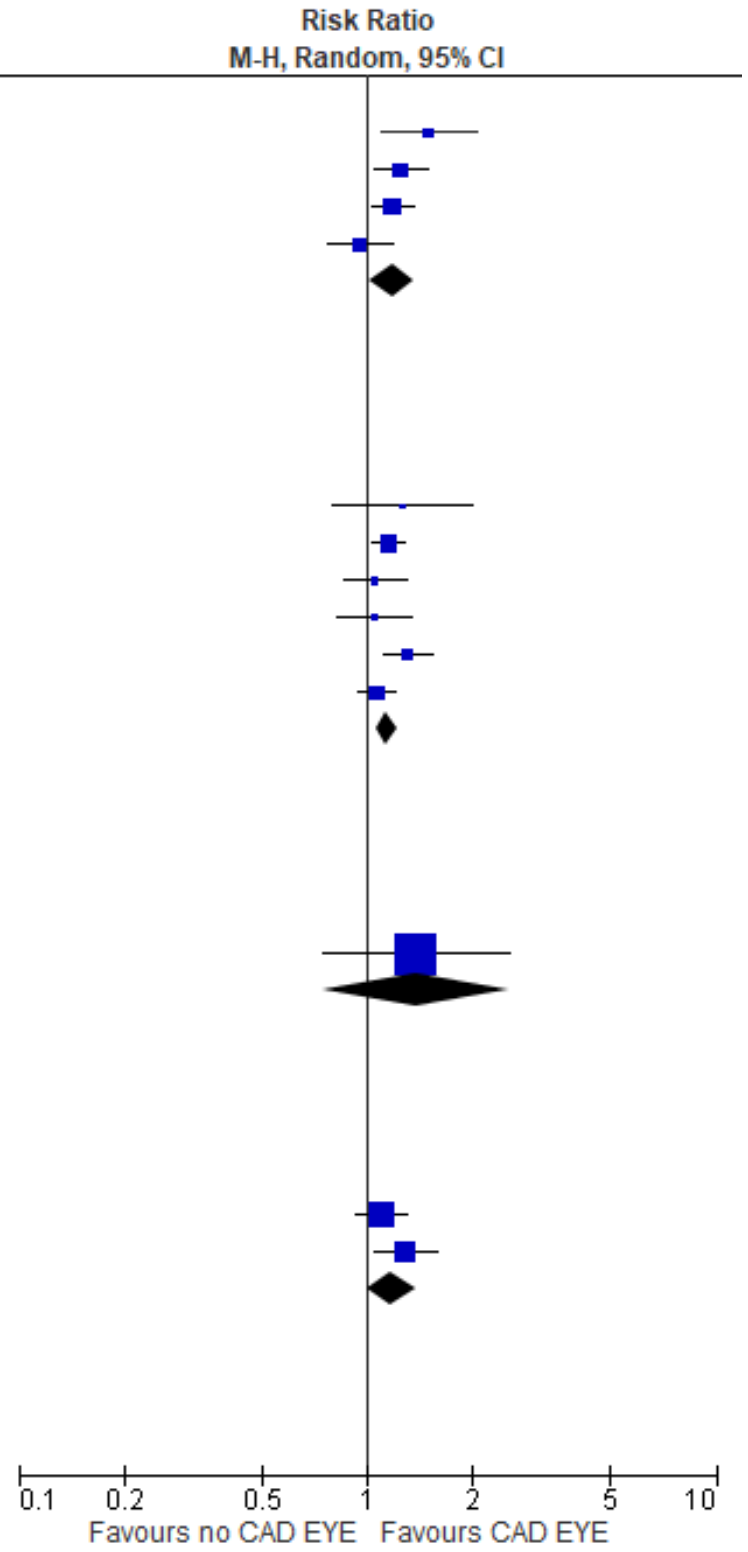

Test for subgroup differences: Chi² = 0.65, df = 3 (P = 0.89), I² = 0%

Abbreviations: ADR, adenoma detection rate; CI, confidence interval; M-H, Mantel-Haenszel.

Supplementary Figure 20. ADR colonoscopy indication subgroup analysis with CAD EYE®-majority in whole studies and within-trial subgroup data – broader screening category

Study or Subgroup	CAD EYE Events	CAD EYE Total	No CAD EYE Events	No CAD EYE Total	Weight	Risk Ratio M-H, Random, 95% CI
**22.3.1 Majority screening**						
Alali 2025 - whole study	24	51	19	51	1.9%	1.26 [0.80, 2.00]
Aniwan 2023 - whole study	344	620	300	625	19.7%	1.16 [1.04, 1.29]
Desai 2024 - screening	101	254	108	286	7.7%	1.05 [0.85, 1.30]
Hiratsuka 2025 - whole study	36	48	33	46	6.1%	1.05 [0.82, 1.33]
Miyaguchi 2024 - FIT-positive	50	83	33	82	3.8%	1.50 [1.09, 2.05]
Nakashima 2023 - whole study	123	207	99	208	9.8%	1.25 [1.04, 1.50]
Rondonotti 2022 - whole study	217	405	179	395	14.2%	1.18 [1.03, 1.36]
Tiankanon 2024 - whole study	200	400	153	400	12.1%	1.31 [1.12, 1.53]
Yamaguchi 2024 - whole study	66	113	72	118	7.7%	0.96 [0.77, 1.18]
Zimmermann-Fraedrich 2025 - whole study	325	812	306	815	17.1%	1.07 [0.94, 1.20]
**Subtotal (95% CI)**		**2993**		**3026**	**100.0%**	**1.15 [1.08, 1.23]**
Total events	1486		1302			
Heterogeneity: Tau² = 0.00; Chi² = 11.89, df = 9 (P = 0.22); I² = 24%						
Test for overall effect: Z = 4.25 (P < 0.0001)						
**22.3.4 Majority Lynch Syndrome surveillance**						
Huneburg 2023 - whole study	18	50	12	46	100.0%	1.38 [0.75, 2.54]
**Subtotal (95% CI)**		**50**		**46**	**100.0%**	**1.38 [0.75, 2.54]**
Total events	18		12			
Heterogeneity: Not applicable						
Test for overall effect: Z = 1.03 (P = 0.30)						
**22.3.6 Majority other surveillance**						
Desai 2024 - surveillance	137	255	116	236	56.3%	1.09 [0.92, 1.30]
Djinbachian 2024 - whole study	113	229	91	238	43.7%	1.29 [1.05, 1.59]
**Subtotal (95% CI)**		**484**		**474**	**100.0%**	**1.18 [1.00, 1.38]**
Total events	250		207			
Heterogeneity: Tau² = 0.00; Chi² = 1.46, df = 1 (P = 0.23); I² = 31%						
Test for overall effect: Z = 1.95 (P = 0.05)						
Test for subgroup differences: Chi² = 0.38, df = 2 (P = 0.83), I² = 0%						

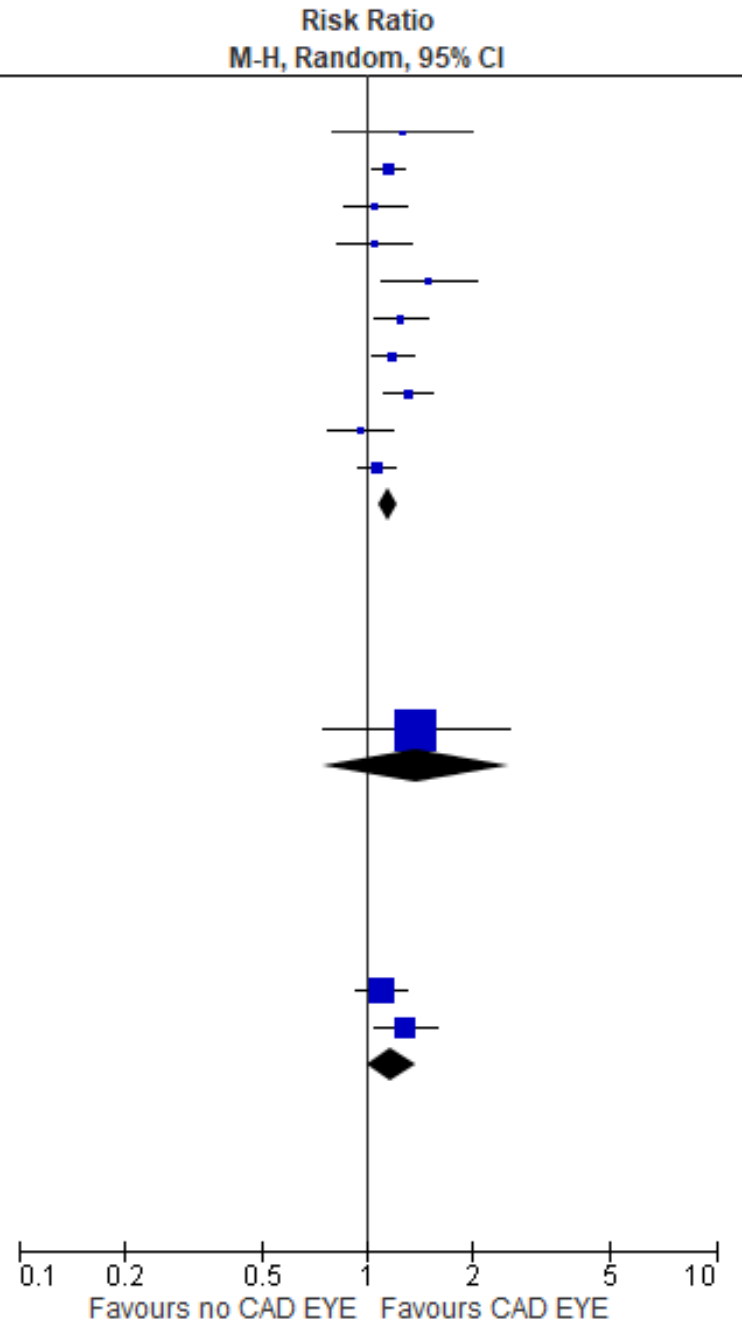

Abbreviations: ADR, adenoma detection rate; CI, confidence interval; M-H, Mantel-Haenszel.

Supplementary Figure 21. APC colonoscopy indication subgroup analysis with CAD EYE®-majority in whole studies and within-trial subgroup data – reported as mean difference

Study or Subgroup	CAD EYE Mean	CAD EYE SD	CAD EYE Total	No CAD EYE Mean	No CAD EYE SD	No CAD EYE Total	Weight	Mean Difference IV, Random, 95% CI
**22.2.1 Majority FIT+ screening**								
Miyaguchi 2024 - whole study	1.31	1.6277	400	0.94	1.4243	400	12.3%	0.37 [0.16, 0.58]
Nakashima 2023 - whole study	1.21	1.36	207	1.01	1.5	208	7.8%	0.20 [-0.08, 0.48]
Rondonotti 2022 - whole study	1.126	1.54	405	0.904	1.32	395	13.7%	0.22 [0.02, 0.42]
Yamaguchi 2024 - whole study	1.4	1.8	113	1.4	2	118	2.6%	0.00 [-0.49, 0.49]
**Subtotal (95% CI)**			**1125**			**1121**	**36.3%**	**0.25 [0.13, 0.38]**
Heterogeneity: Tau² = 0.00; Chi² = 2.43, df = 3 (P = 0.49); I² = 0%								
Test for overall effect: Z = 4.01 (P < 0.0001)								
**22.2.2 Majority other screening or any screening combined**								
Alali 2025 - whole study	0.75	0.99	51	0.51	0.78	51	5.1%	0.24 [-0.11, 0.59]
Aniwan 2023 - whole study	1.21	1.65	620	0.97	1.54	625	16.4%	0.24 [0.06, 0.42]
Desai 2024 - screening	0.748	1.322	254	0.776	1.628	286	9.3%	-0.03 [-0.28, 0.22]
Tiankanon 2024 - whole study	1.04	1.63	400	0.66	1.05	400	14.7%	0.38 [0.19, 0.57]
**Subtotal (95% CI)**			**1325**			**1362**	**45.5%**	**0.22 [0.05, 0.39]**
Heterogeneity: Tau² = 0.02; Chi² = 6.52, df = 3 (P = 0.09); I² = 54%								
Test for overall effect: Z = 2.54 (P = 0.01)								
**22.2.3 Majority surveillance**								
Desai 2024 - surveillance	1.231	1.824	255	0.936	1.285	236	7.7%	0.30 [0.02, 0.57]
Djinbachian 2024 - whole study	0.86	1.2288	229	0.75	1.3313	238	10.5%	0.11 [-0.12, 0.34]
**Subtotal (95% CI)**			**484**			**474**	**18.2%**	**0.19 [0.01, 0.36]**
Heterogeneity: Tau² = 0.00; Chi² = 1.00, df = 1 (P = 0.32); I² = 0%								
Test for overall effect: Z = 2.05 (P = 0.04)								
**Total (95% CI)**			**2934**			**2957**	**100.0%**	**0.23 [0.15, 0.31]**
Heterogeneity: Tau² = 0.00; Chi² = 10.33, df = 9 (P = 0.32); I² = 13%								
Test for overall effect: Z = 5.58 (P < 0.00001)								
Test for subgroup differences: Chi² = 0.39, df = 2 (P = 0.82), I² = 0%								

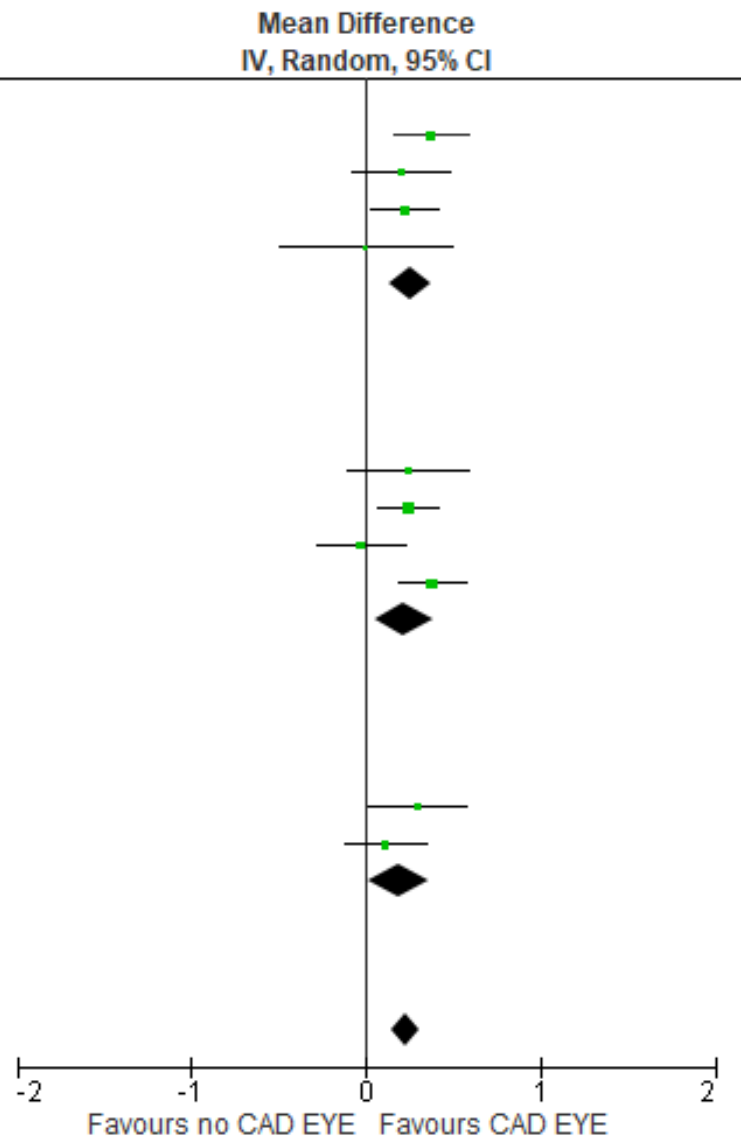

Abbreviations: APC, adenomas per colonoscopy; CI, confidence interval; IV, inverse variance; SD, standard deviation.

2.5.2 Discovery™

Supplementary Figure 22. ADR colonoscopy indication subgroup analysis with Discovery™ - within-trial subgroup analysis

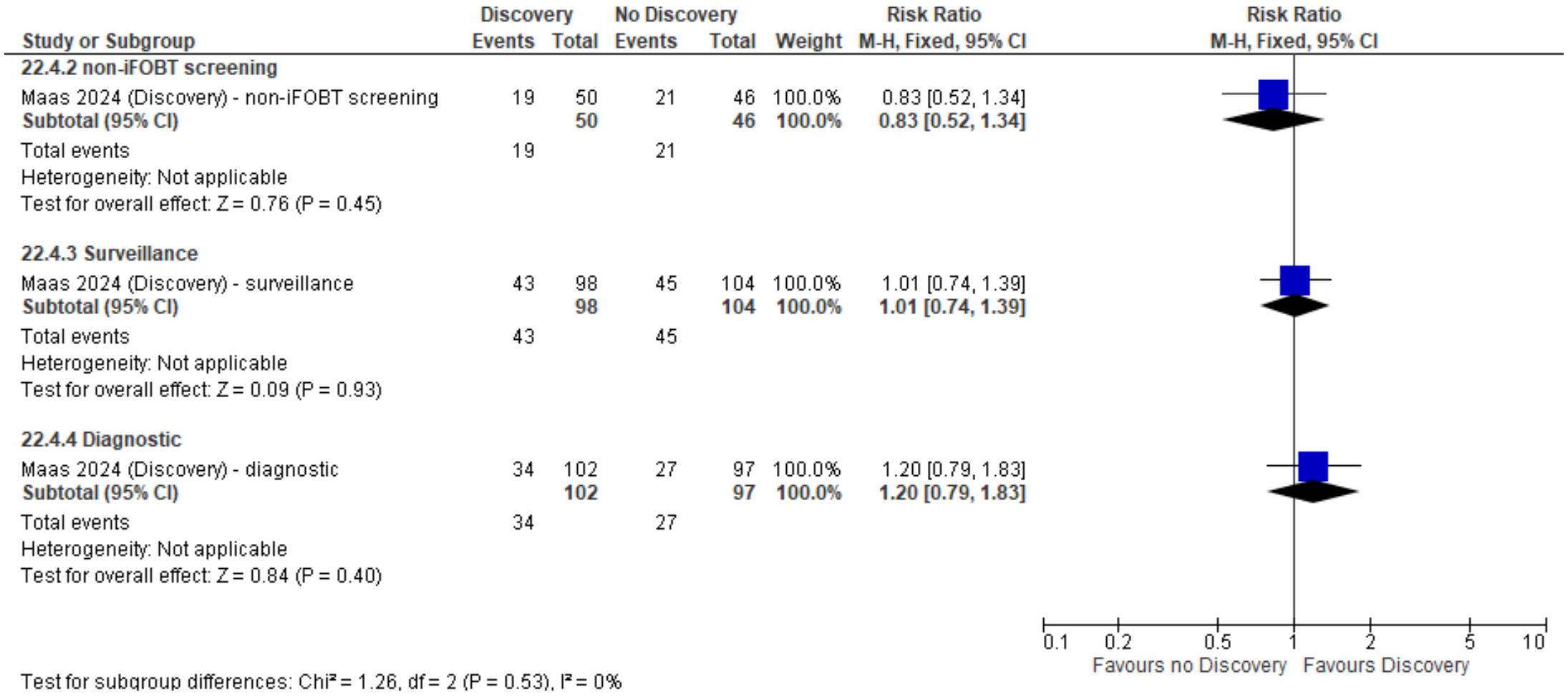

Abbreviations: ADR, adenoma detection rate; CI, confidence interval; iFOBT, immunochemical faecal occult blood test; M-H, Mantel-Haenszel.

Supplementary Figure 23. APC colonoscopy indication subgroup analysis with Discovery™ - within-trial subgroup analysis - reported as mean difference

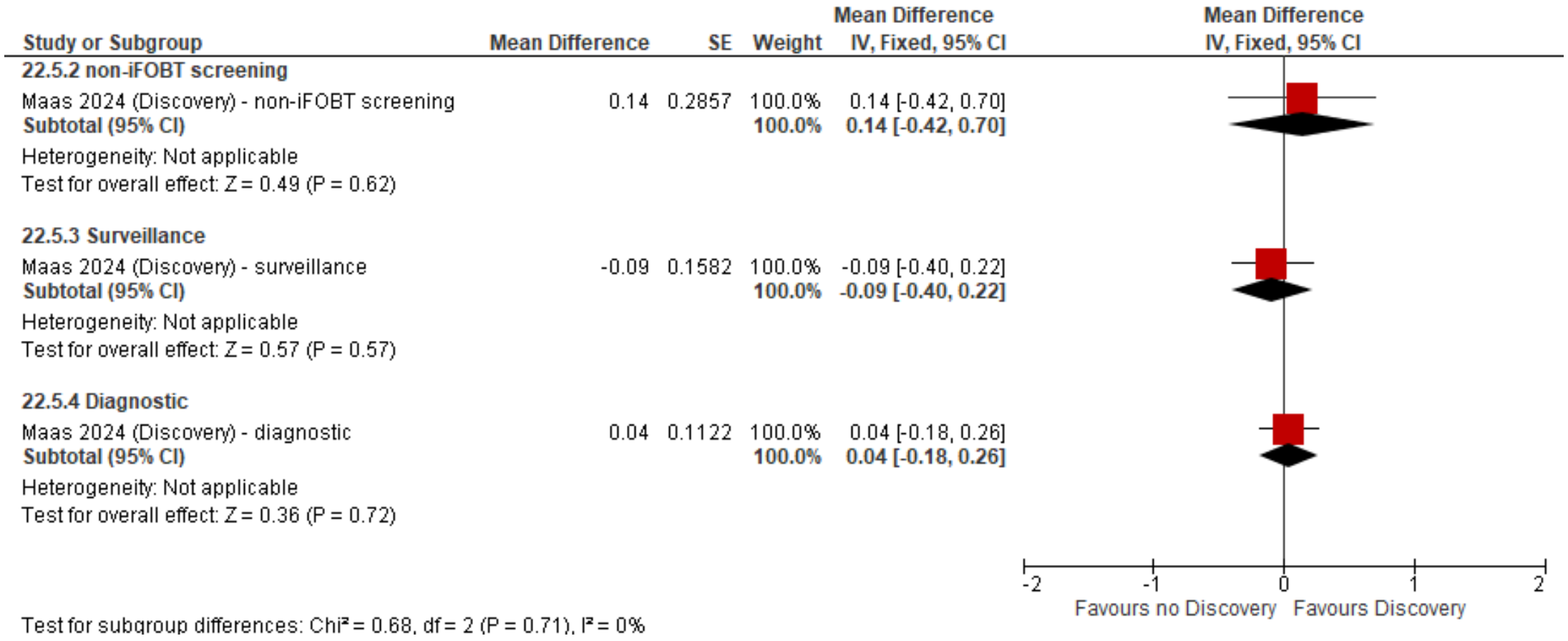

Abbreviations: APC, adenomas per colonoscopy; CI, confidence interval; iFOBT, immunochemical faecal occult blood test; IV, inverse variance; SE, standard error.

2.5.3 ENDO-AID™

Supplementary Figure 24. ADR colonoscopy indication subgroup analysis with ENDO-AID™-majority in whole studies and within-trial subgroup data

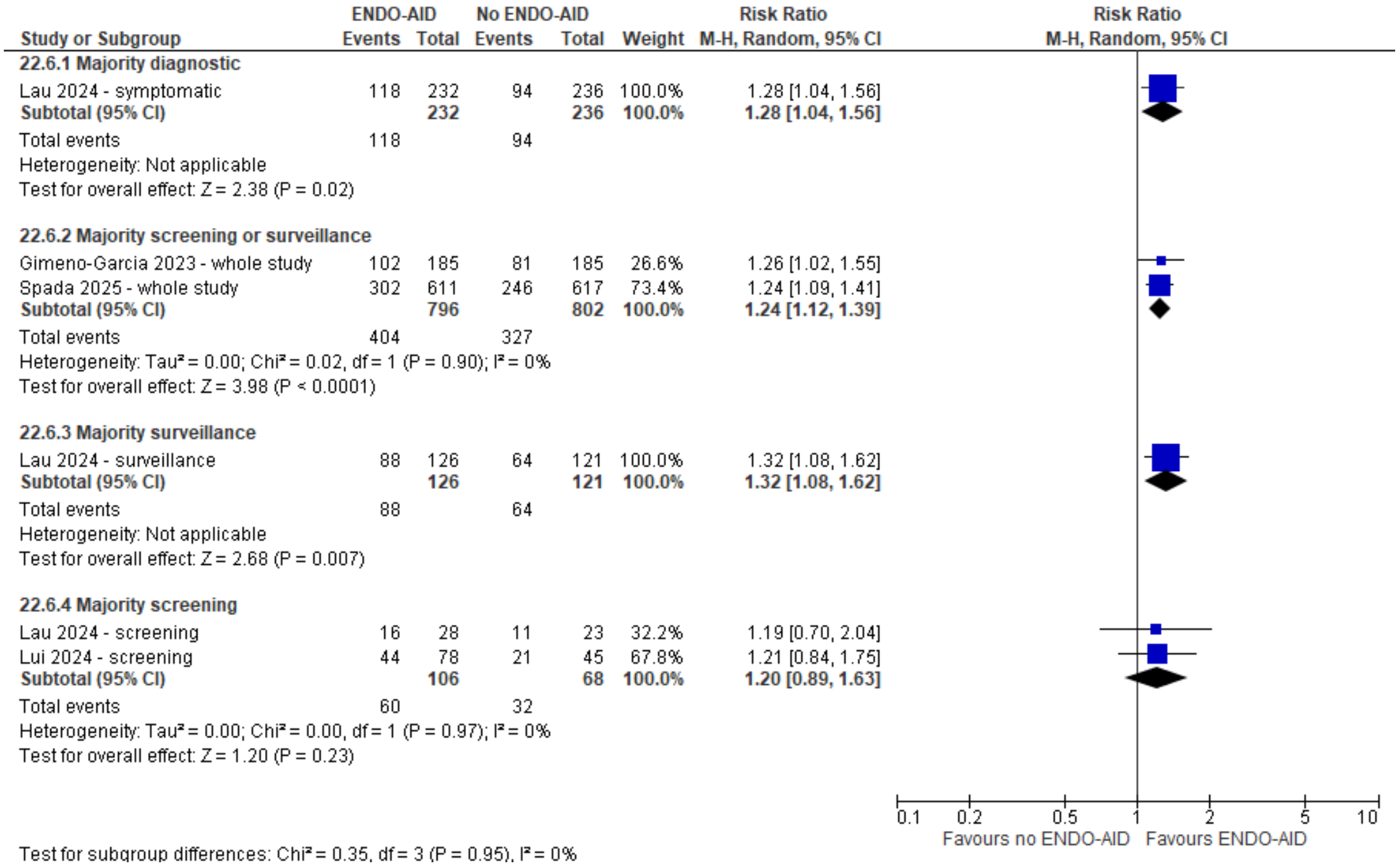

Abbreviations: ADR, adenoma detection rate; CI, confidence interval; M-H, Mantel-Haenszel.

Supplementary Figure 25. APC colonoscopy indication subgroup analysis with ENDO-AID™-majority in whole studies and within-trial subgroup data – reported as mean difference

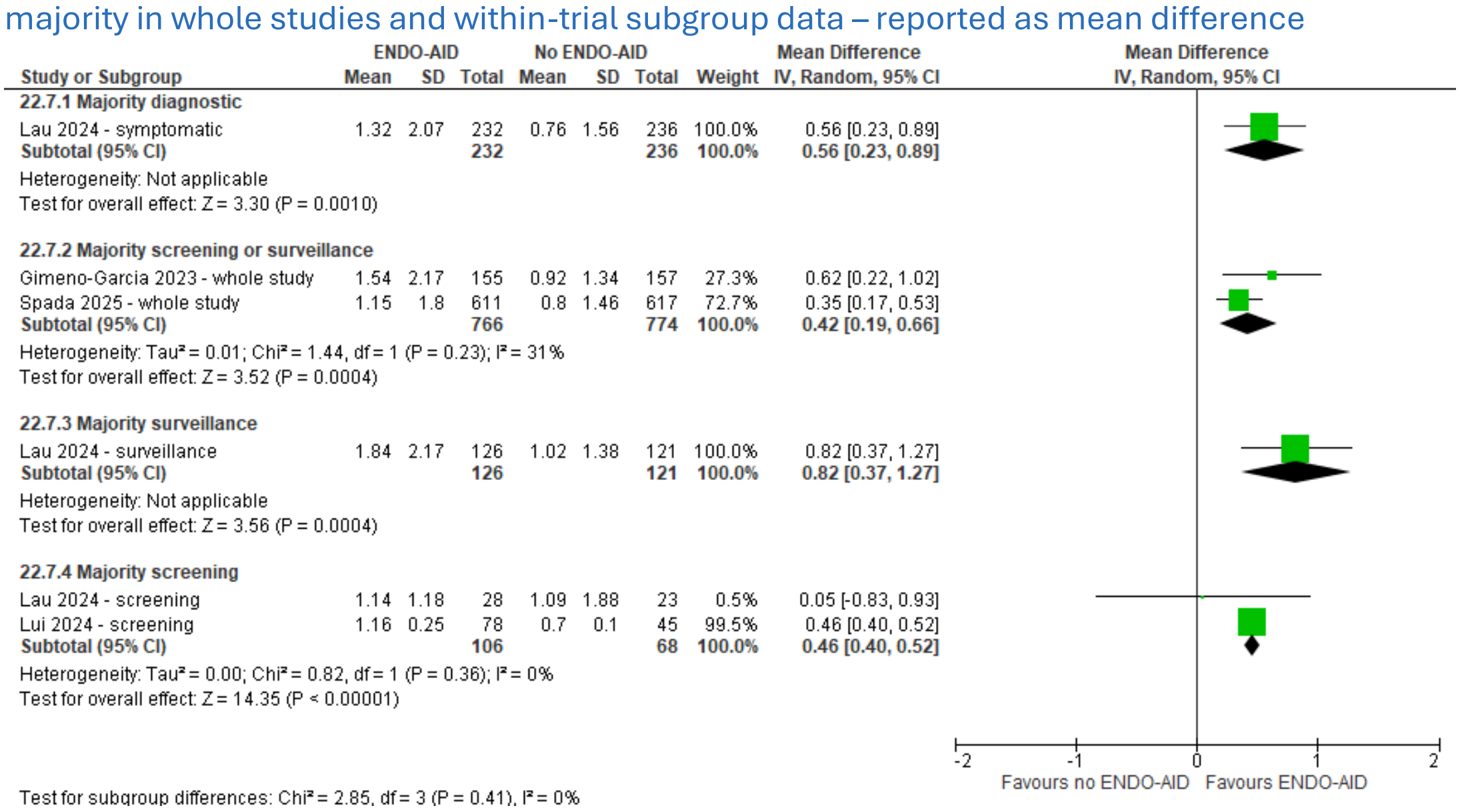

Abbreviations: APC, adenomas per colonoscopy; CI, confidence interval; IV, inverse variance; SD, standard deviation.

2.5.4 EndoScreener®

Supplementary Figure 26. ADR colonoscopy indication subgroup analysis with EndoScreener® - majority in whole studies

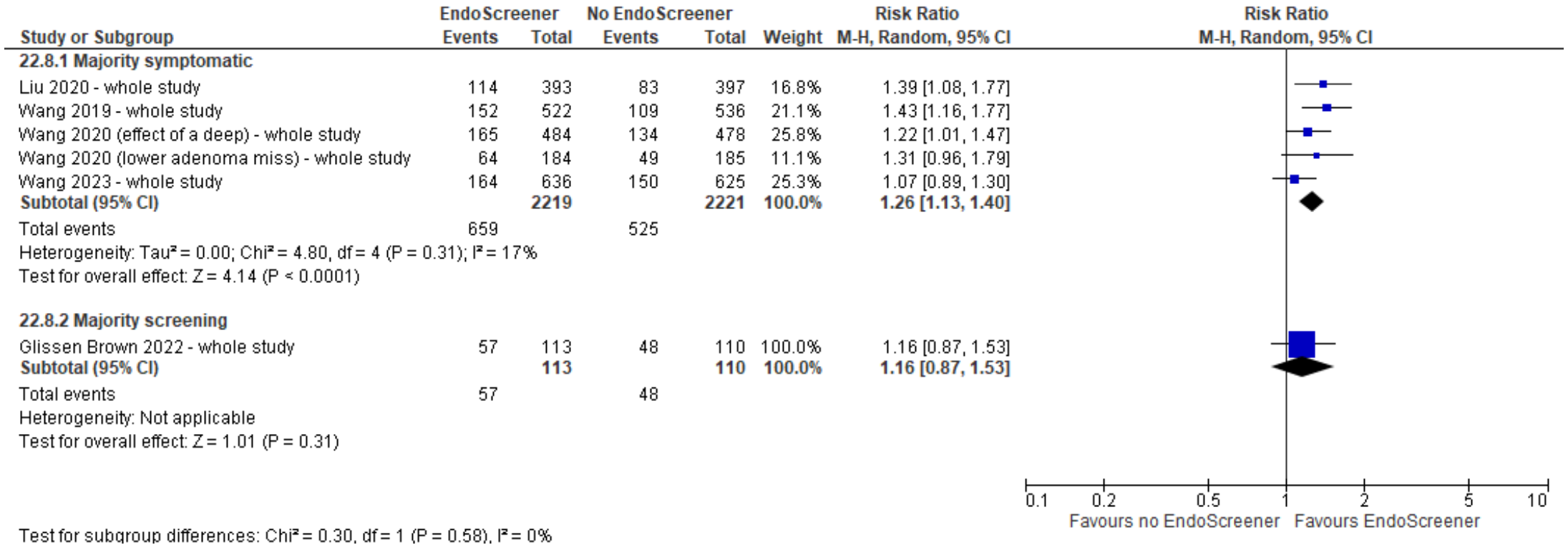

Abbreviations: ADR, adenoma detection rate; CI, confidence interval; M-H, Mantel-Haenszel.

Supplementary Figure 27. APC colonoscopy indication subgroup analysis with EndoScreener® - majority in whole studies - reported as IRR

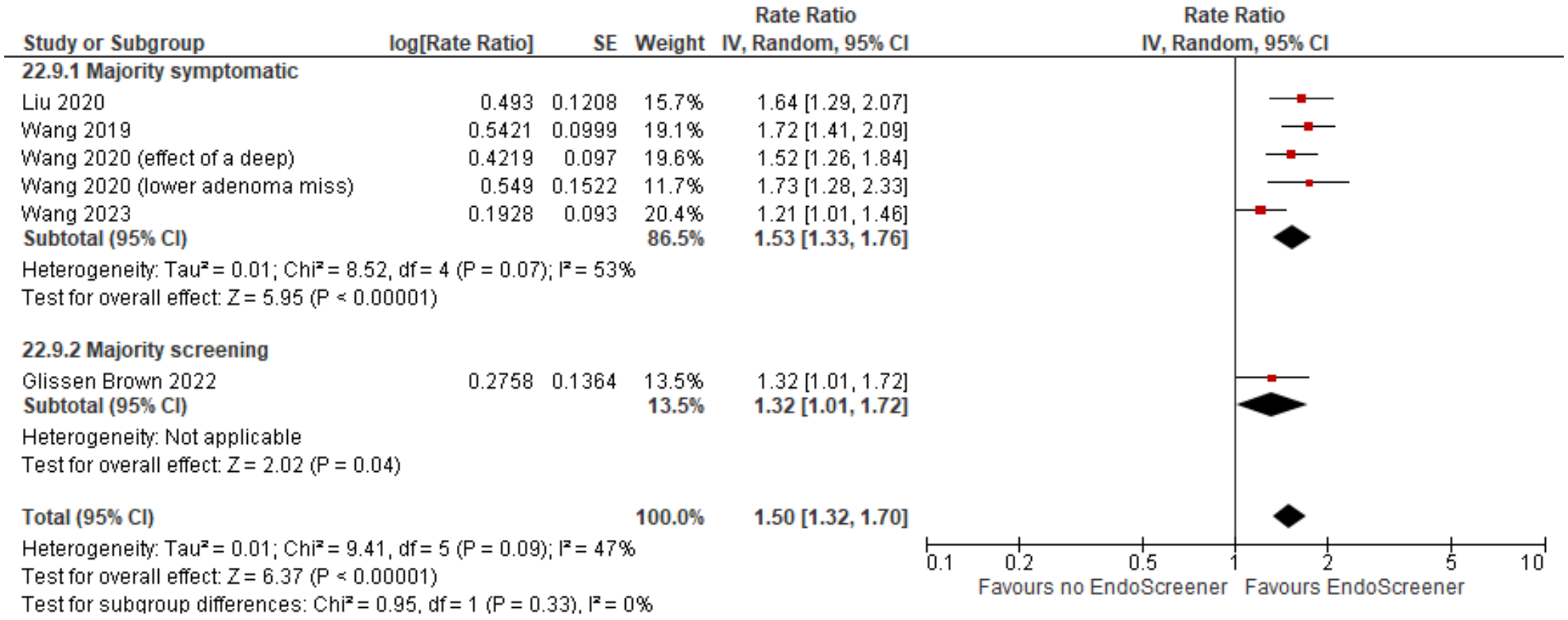

Abbreviations: APC, adenomas per colonoscopy; CI, confidence interval; IRR, incidence rate ratio; IV, inverse variance; SE, standard error.

2.5.5 GI Genius™

Supplementary Figure 28. ADR colonoscopy indication subgroup analysis with GI Genius™ - majority in whole studies and within-trial subgroup data – granular screening and surveillance categories

Study or Subgroup	GI Genius Events	GI Genius Total	No GI Genius Events	No GI Genius Total	Weight	Risk Ratio M-H, Random, 95% CI
**22.10.1 Majority FIT+ or within BSCP including post-polypectomy surveillance**						
Ahmad 2023 - whole study	220	308	134	306	20.8%	1.63 [1.41, 1.89]
Mangas-Sanjuan 2023 - whole study	1033	1610	990	1603	24.0%	1.04 [0.99, 1.10]
Repici 2020 - FIT-positive	61	102	46	105	15.3%	1.37 [1.04, 1.79]
Repici 2022 - FIT-positive	20	24	10	24	7.8%	2.00 [1.21, 3.32]
Seager 2024 - screening	404	594	368	603	23.1%	1.11 [1.02, 1.21]
Thiruvengadam 2024 - FIT-positive	20	32	15	32	9.0%	1.33 [0.84, 2.10]
**Subtotal (95% CI)**		**2670**		**2673**	**100.0%**	**1.30 [1.10, 1.55]**
Total events	1758		1563			
Heterogeneity: Tau² = 0.03; Chi² = 41.35, df = 5 (P < 0.00001); I² = 88%						
Test for overall effect: Z = 3.02 (P = 0.003)						
**22.10.2 Majority post-polypectomy surveillance**						
Karsenti 2023 - whole study	376	1003	341	1012	76.9%	1.11 [0.99, 1.25]
Repici 2022 - post-polypectomy surveillance	77	127	65	118	23.1%	1.10 [0.89, 1.36]
**Subtotal (95% CI)**		**1130**		**1130**	**100.0%**	**1.11 [1.00, 1.23]**
Total events	453		406			
Heterogeneity: Tau² = 0.00; Chi² = 0.01, df = 1 (P = 0.93); I² = 0%						
Test for overall effect: Z = 1.98 (P = 0.05)						
**22.10.3 Majority symptomatic/diagnostic**						
Repici 2020 - GE symptoms	36	76	26	85	18.8%	1.55 [1.04, 2.31]
Repici 2022 - GI symptoms	33	81	40	94	22.5%	0.96 [0.67, 1.36]
Seager 2024 - symptomatic	151	386	109	383	43.2%	1.37 [1.12, 1.68]
Thiruvengadam 2024 - diagnostic	35	104	23	104	15.5%	1.52 [0.97, 2.39]
**Subtotal (95% CI)**		**647**		**666**	**100.0%**	**1.32 [1.08, 1.60]**
Total events	255		198			
Heterogeneity: Tau² = 0.01; Chi² = 4.36, df = 3 (P = 0.23); I² = 31%						
Test for overall effect: Z = 2.72 (P = 0.006)						
**22.10.4 Majority other screening**						
Repici 2022 - primary CRC screening	46	98	32	94	22.2%	1.38 [0.97, 1.96]
Thiruvengadam 2024 - screening	160	381	120	359	77.8%	1.26 [1.04, 1.52]
**Subtotal (95% CI)**		**479**		**453**	**100.0%**	**1.28 [1.09, 1.51]**
Total events	206		152			
Heterogeneity: Tau² = 0.00; Chi² = 0.21, df = 1 (P = 0.65); I² = 0%						
Test for overall effect: Z = 2.95 (P = 0.003)						
**22.10.5 Majority Lynch Syndrome surveillance**						
Ortiz 2024 - whole study	70	214	79	216	100.0%	0.89 [0.69, 1.16]
**Subtotal (95% CI)**		**214**		**216**	**100.0%**	**0.89 [0.69, 1.16]**
Total events	70		79			
Heterogeneity: Not applicable						
Test for overall effect: Z = 0.84 (P = 0.40)						
**22.10.6 Majority other surveillance/not defined**						
Thiruvengadam 2024 - surveillance	19	33	31	55	22.8%	1.02 [0.70, 1.48]
Wallace 2022 - whole study	72	116	70	114	77.2%	1.01 [0.82, 1.24]
**Subtotal (95% CI)**		**149**		**169**	**100.0%**	**1.01 [0.85, 1.21]**
Total events	91		101			
Heterogeneity: Tau² = 0.00; Chi² = 0.00, df = 1 (P = 0.96); I² = 0%						
Test for overall effect: Z = 0.14 (P = 0.89)						
**22.10.7 Majority mixed screening/surveillance**						
Repici 2020 - screening/surveillance	90	163	67	154	100.0%	1.27 [1.01, 1.59]
**Subtotal (95% CI)**		**163**		**154**	**100.0%**	**1.27 [1.01, 1.59]**
Total events	90		67			
Heterogeneity: Not applicable						
Test for overall effect: Z = 2.06 (P = 0.04)						
Test for subgroup differences: Chi² = 12.06, df = 6 (P = 0.06), I² = 50.2%						

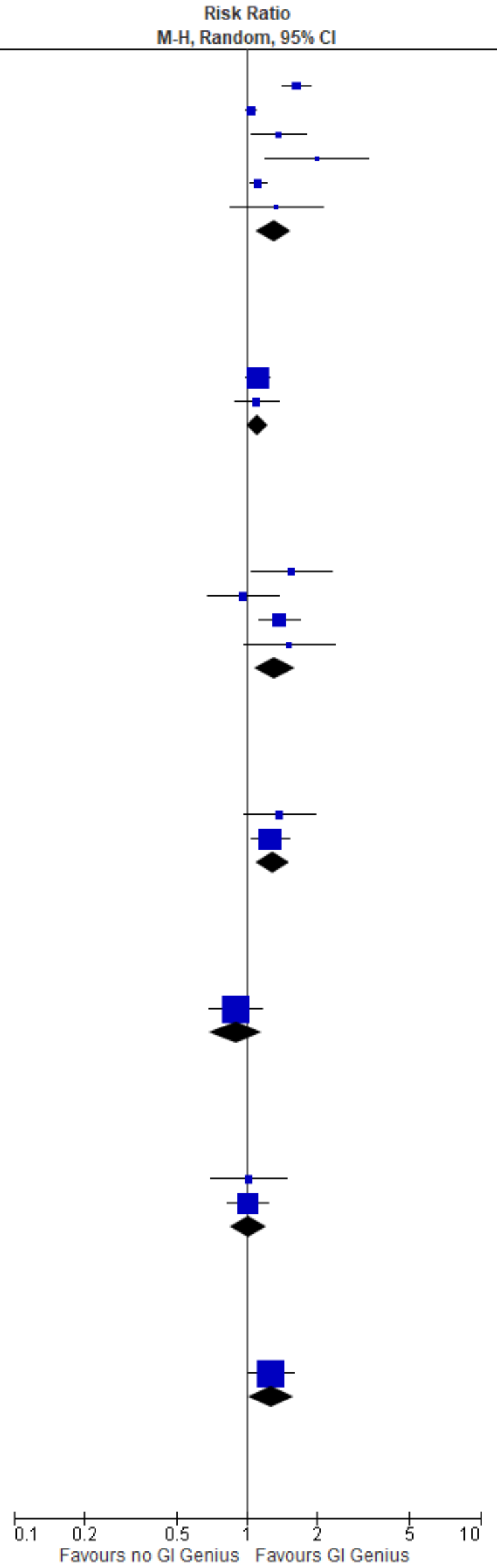

Abbreviations: ADR, adenoma detection rate; BCSP, Bowel Cancer Screening Programme; CI, confidence interval; CRC, colorectal cancer; FIT, faecal immunochemical test; GE/GI, gastrointestinal symptoms; M-H, Mantel-Haenszel.

Supplementary Figure 29. ADR colonoscopy indication subgroup analysis with GI Genius™ - majority in whole studies and within-trial subgroup data – screening and surveillance combined as single category

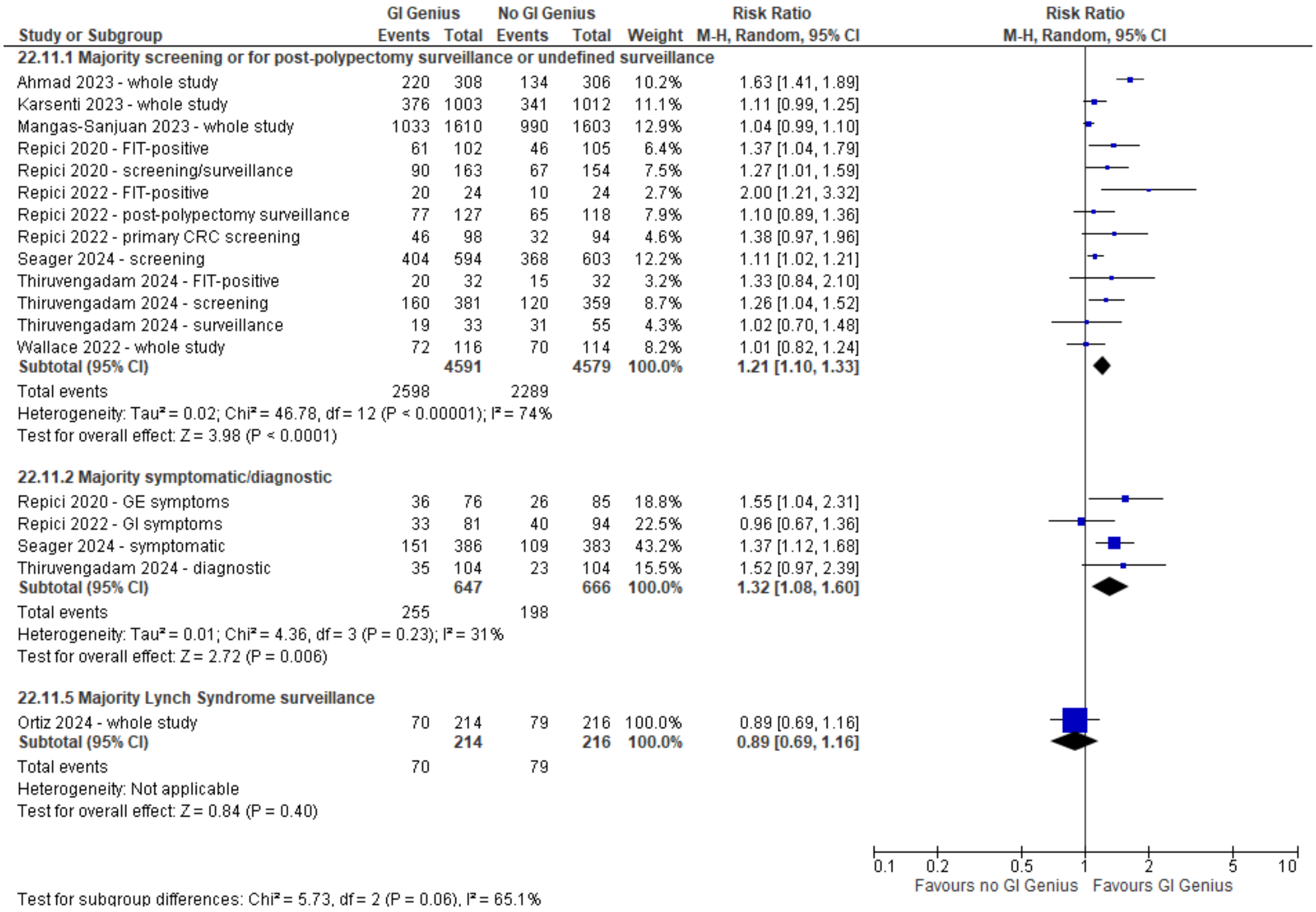

Abbreviations: ADR, adenoma detection rate; CI, confidence interval; CRC, colorectal cancer; FIT, faecal immunochemical test; GE/GI, gastrointestinal symptoms; M-H, Mantel-Haenszel.

Supplementary Figure 30. ADR colonoscopy indication subgroup analysis with GI Genius™ - majority in whole studies and within-trial subgroup data – all screening combined and separated from surveillance

Study or Subgroup	GI Genius Events	GI Genius Total	No GI Genius Events	No GI Genius Total	Weight	Risk Ratio M-H, Random, 95% CI
**22.14.1 Majority FIT+ or within BSCP or other screening**						
Ahmad 2023 - whole study	220	308	134	306	14.3%	1.63 [1.41, 1.89]
Mangas-Sanjuan 2023 - whole study	1033	1610	990	1603	16.9%	1.04 [0.99, 1.10]
Repici 2020 - FIT-positive	61	102	46	105	10.1%	1.37 [1.04, 1.79]
Repici 2020 - screening/surveillance	90	163	67	154	11.4%	1.27 [1.01, 1.59]
Repici 2022 - FIT-positive	20	24	10	24	4.8%	2.00 [1.21, 3.32]
Repici 2022 - primary CRC screening	46	98	32	94	7.7%	1.38 [0.97, 1.96]
Seager 2024 - screening	404	594	368	603	16.2%	1.11 [1.02, 1.21]
Thiruvengadam 2024 - FIT-positive	20	32	15	32	5.6%	1.33 [0.84, 2.10]
Thiruvengadam 2024 - screening	160	381	120	359	12.8%	1.26 [1.04, 1.52]
**Subtotal (95% CI)**		**3312**		**3280**	**100.0%**	**1.29 [1.13, 1.47]**
Total events	2054		1782			
Heterogeneity: Tau² = 0.03; Chi² = 45.64, df = 8 (P < 0.00001); I² = 82%						
Test for overall effect: Z = 3.84 (P = 0.0001)						
**22.14.2 Majority post-polypectomy surveillance or other surveillance**						
Karsenti 2023 - whole study	376	1003	341	1012	57.7%	1.11 [0.99, 1.25]
Repici 2022 - post-polypectomy surveillance	77	127	65	118	17.3%	1.10 [0.89, 1.36]
Thiruvengadam 2024 - surveillance	19	33	31	55	5.7%	1.02 [0.70, 1.48]
Wallace 2022 - whole study	72	116	70	114	19.3%	1.01 [0.82, 1.24]
**Subtotal (95% CI)**		**1279**		**1299**	**100.0%**	**1.08 [0.99, 1.19]**
Total events	544		507			
Heterogeneity: Tau² = 0.00; Chi² = 0.78, df = 3 (P = 0.85); I² = 0%						
Test for overall effect: Z = 1.78 (P = 0.07)						
**22.14.3 Majority symptomatic/diagnostic**						
Repici 2020 - GE symptoms	36	76	26	85	18.8%	1.55 [1.04, 2.31]
Repici 2022 - GI symptoms	33	81	40	94	22.5%	0.96 [0.67, 1.36]
Seager 2024 - symptomatic	151	386	109	383	43.2%	1.37 [1.12, 1.68]
Thiruvengadam 2024 - diagnostic	35	104	23	104	15.5%	1.52 [0.97, 2.39]
**Subtotal (95% CI)**		**647**		**666**	**100.0%**	**1.32 [1.08, 1.60]**
Total events	255		198			
Heterogeneity: Tau² = 0.01; Chi² = 4.36, df = 3 (P = 0.23); I² = 31%						
Test for overall effect: Z = 2.72 (P = 0.006)						
**22.14.5 Majority Lynch Syndrome surveillance**						
Ortiz 2024 - whole study	70	214	79	216	100.0%	0.89 [0.69, 1.16]
**Subtotal (95% CI)**		**214**		**216**	**100.0%**	**0.89 [0.69, 1.16]**
Total events	70		79			
Heterogeneity: Not applicable						
Test for overall effect: Z = 0.84 (P = 0.40)						
Test for subgroup differences: Chi² = 10.05, df = 3 (P = 0.02), I² = 70.1%						

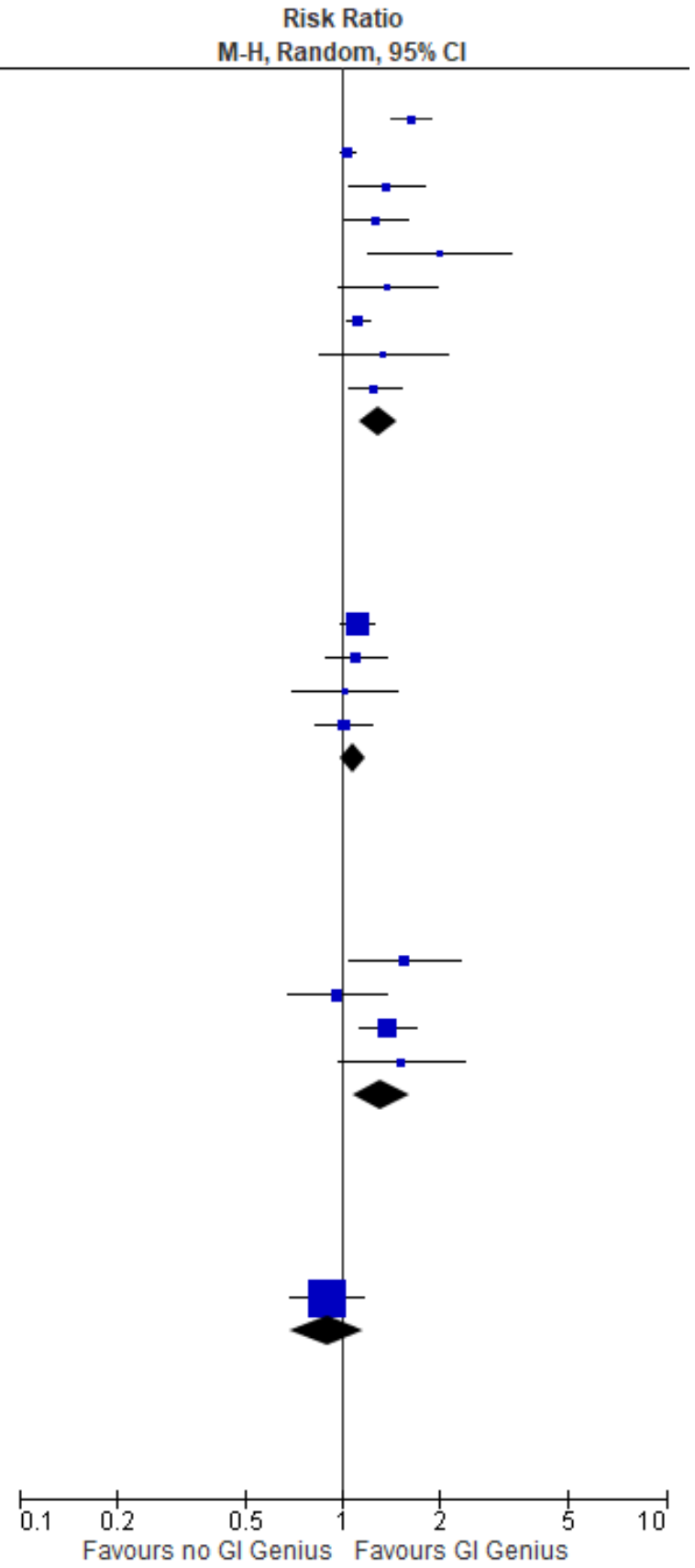

Abbreviations: ADR, adenoma detection rate; BCSP, Bowel Cancer Screening Programme; CI, confidence interval; CRC, colorectal cancer; FIT, faecal immunochemical test; GE/GI, gastrointestinal symptoms; M-H, Mantel-Haenszel.

Supplementary Figure 31. APC colonoscopy indication subgroup analysis with GI Genius™ - majority in whole studies and within-trial subgroup data – granular screening and surveillance categories

	GI Genius			No GI Genius				Mean Difference
**Study or Subgroup**	**Mean**	**SD**	**Total**	**Mean**	**SD**	**Total**	**Weight**	**IV, Random, 95% CI**
**22.12.1 Majority FIT+ or within BSCP including post-polypectomy surveillance**								
Ahmad 2023 - whole study	2.4	2.9	308	2.1	2.6	306	7.3%	0.30 [-0.14, 0.74]
Mangas-Sanjuan 2023 - whole study	1.78	2.38	1610	1.59	2.2	1603	48.8%	0.19 [0.03, 0.35]
Repici 2020 - whole study	1.07	1.54	341	0.71	1.2	344	30.3%	0.36 [0.15, 0.57]
Seager 2024 - screening	2.08	3.36	608	1.61	2.16	616	13.6%	0.47 [0.15, 0.79]
**Subtotal (95% CI)**			**2867**			**2869**	**100.0%**	**0.29 [0.17, 0.41]**
Heterogeneity: Tau² = 0.00; Chi² = 3.20, df = 3 (P = 0.36); I² = 6%								
Test for overall effect: Z = 4.73 (P < 0.00001)								
**22.12.2 Majority post-polypectomy surveillance**								
Karsenti 2023 - whole study	0.89	1.49	1003	0.71	1.3	1012	83.3%	0.18 [0.06, 0.30]
Repici 2022 - whole study	1.26	1.82	330	1.04	1.75	330	16.7%	0.22 [-0.05, 0.49]
**Subtotal (95% CI)**			**1333**			**1342**	**100.0%**	**0.19 [0.08, 0.30]**
Heterogeneity: Tau² = 0.00; Chi² = 0.07, df = 1 (P = 0.79); I² = 0%								
Test for overall effect: Z = 3.28 (P = 0.001)								
**22.12.3 Majority symptomatic**								
Seager 2024 - symptomatic	0.76	1.33	393	0.57	1.18	392	100.0%	0.19 [0.01, 0.37]
**Subtotal (95% CI)**			**393**			**392**	**100.0%**	**0.19 [0.01, 0.37]**
Heterogeneity: Not applicable								
Test for overall effect: Z = 2.12 (P = 0.03)								
**22.12.4 Majority other screening**								
Thiruvengadam 2024 - whole study	0.89	1.46	550	0.6	1.12	550	100.0%	0.29 [0.14, 0.44]
**Subtotal (95% CI)**			**550**			**550**	**100.0%**	**0.29 [0.14, 0.44]**
Heterogeneity: Not applicable								
Test for overall effect: Z = 3.70 (P = 0.0002)								
**22.12.5 Majority Lynch Syndrome surveillance**								
Ortiz 2024 - whole study	0.64	1.57	214	0.64	1.17	216	100.0%	0.00 [-0.26, 0.26]
**Subtotal (95% CI)**			**214**			**216**	**100.0%**	**0.00 [-0.26, 0.26]**
Heterogeneity: Not applicable								
Test for overall effect: Z = 0.00 (P = 1.00)								
**22.12.6 Majority other surveillance/not defined**								
Wallace 2022 - whole study	1.79	2.63	116	1.46	1.7	114	100.0%	0.33 [-0.24, 0.90]
**Subtotal (95% CI)**			**116**			**114**	**100.0%**	**0.33 [-0.24, 0.90]**
Heterogeneity: Not applicable								
Test for overall effect: Z = 1.13 (P = 0.26)								

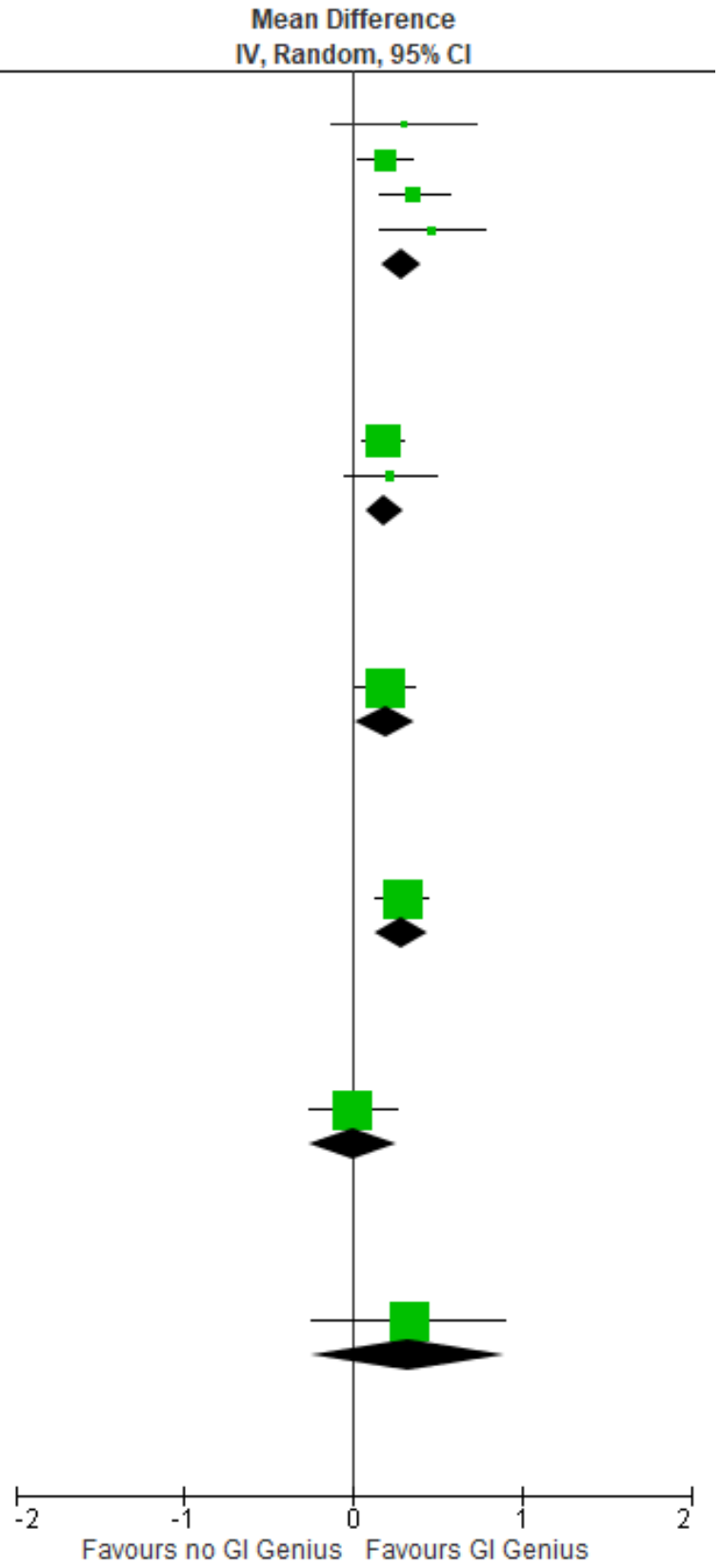

Test for subgroup differences: Chi² = 5.32, df = 5 (P = 0.38), I² = 6.0%

Abbreviations: APC, adenomas per colonoscopy; BCSP, Bowel Cancer Screening Programme; CI, confidence interval; FIT, faecal immunochemical test; IV, inverse variance; SD, standard deviation.

Supplementary Figure 32. APC colonoscopy indication subgroup analysis with GI Genius™ - majority in whole studies and within-trial subgroup data – screening and surveillance combined as single category

Study or Subgroup	GI Genius Mean	GI Genius SD	GI Genius Total	No GI Genius Mean	No GI Genius SD	No GI Genius Total	Weight	Mean Difference IV, Random, 95% CI
**22.13.1 Majority FIT+ or within BSCP including post-polypectomy surveillance**								
Ahmad 2023 - whole study	2.4	2.9	308	2.1	2.6	306	2.6%	0.30 [-0.14, 0.74]
Karsenti 2023 - whole study	0.89	1.49	1003	0.71	1.3	1012	32.8%	0.18 [0.06, 0.30]
Mangas-Sanjuan 2023 - whole study	1.78	2.38	1610	1.59	2.2	1603	19.5%	0.19 [0.03, 0.35]
Repici 2020 - whole study	1.07	1.54	341	0.71	1.2	344	11.4%	0.36 [0.15, 0.57]
Repici 2022 - whole study	1.26	1.82	330	1.04	1.75	330	6.6%	0.22 [-0.05, 0.49]
Seager 2024 - screening	2.08	3.36	608	1.61	2.16	616	4.9%	0.47 [0.15, 0.79]
Thiruvengadam 2024 - whole study	0.89	1.46	550	0.6	1.12	550	20.7%	0.29 [0.14, 0.44]
Wallace 2022 - whole study	1.79	2.63	116	1.46	1.7	114	1.5%	0.33 [-0.24, 0.90]
**Subtotal (95% CI)**			**4866**			**4875**	**100.0%**	**0.25 [0.18, 0.32]**
Heterogeneity: Tau² = 0.00; Chi² = 5.18, df = 7 (P = 0.64); I² = 0%								
Test for overall effect: Z = 6.93 (P < 0.00001)								
**22.13.3 Majority symptomatic**								
Seager 2024 - symptomatic	0.76	1.33	393	0.57	1.18	392	100.0%	0.19 [0.01, 0.37]
**Subtotal (95% CI)**			**393**			**392**	**100.0%**	**0.19 [0.01, 0.37]**
Heterogeneity: Not applicable								
Test for overall effect: Z = 2.12 (P = 0.03)								
**22.13.5 Majority Lynch Syndrome surveillance**								
Ortiz 2024 - whole study	0.64	1.57	214	0.64	1.17	216	100.0%	0.00 [-0.26, 0.26]
**Subtotal (95% CI)**			**214**			**216**	**100.0%**	**0.00 [-0.26, 0.26]**
Heterogeneity: Not applicable								
Test for overall effect: Z = 0.00 (P = 1.00)								
Test for subgroup differences: Chi² = 3.38, df = 2 (P = 0.18), I² = 40.9%								

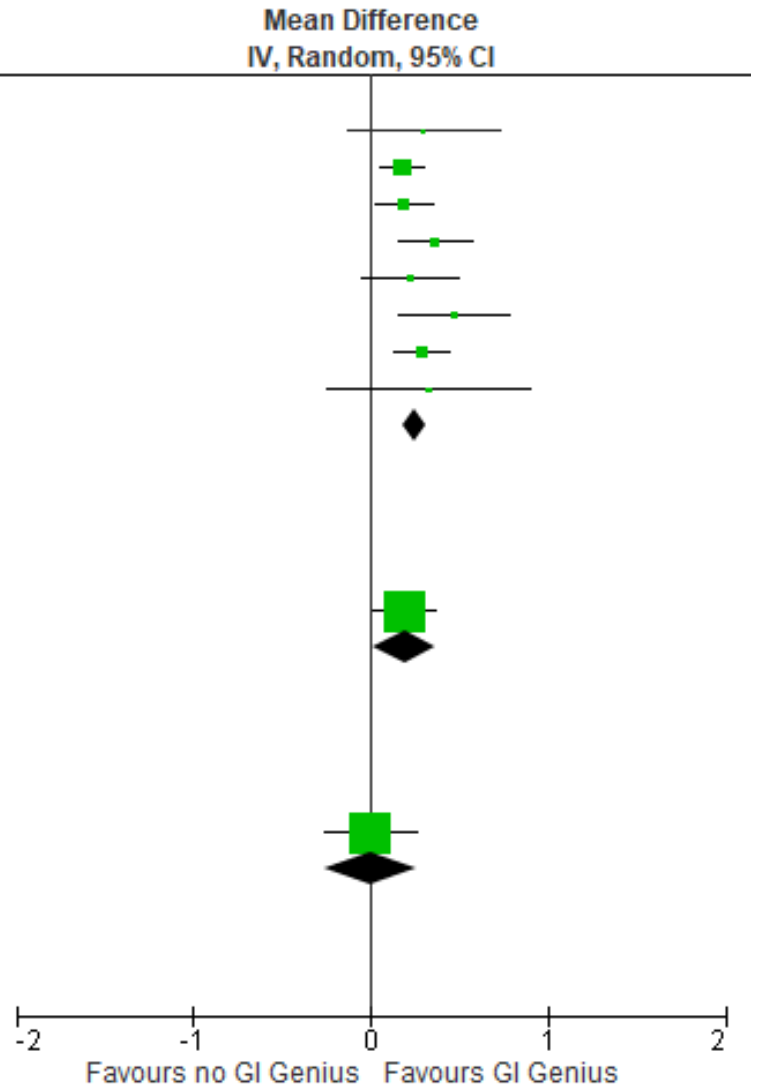

Abbreviations: APC, adenomas per colonoscopy; BCSP, Bowel Cancer Screening Programme; CI, confidence interval; FIT, faecal immunochemical test; IV, inverse variance; SD, standard deviation.

2.5.6 MAGENTIQ-COLO™

Supplementary Figure 33. ADR colonoscopy indication subgroup analysis with MAGENTIQ-COLO™ - within-trial subgroup data

Study or Subgroup	MAGENTIQ-COLO Events	MAGENTIQ-COLO Total	No MAGENTIQ-COLO Events	No MAGENTIQ-COLO Total	Weight	Risk Ratio M-H, Fixed, 95% CI
**22.33.2 non-iFOBT screening**						
Maas 2024 (MAGENTIQ-COLO) - non-iFOBT screening	88	255	69	259	100.0%	1.30 [1.00, 1.69]
**Subtotal (95% CI)**		**255**		**259**	**100.0%**	**1.30 [1.00, 1.69]**
Total events	88		69			
Heterogeneity: Not applicable						
Test for overall effect: Z = 1.92 (P = 0.05)						
**22.33.3 Surveillance**						
Maas 2024 (MAGENTIQ-COLO) - surveillance	79	194	69	208	100.0%	1.23 [0.95, 1.59]
**Subtotal (95% CI)**		**194**		**208**	**100.0%**	**1.23 [0.95, 1.59]**
Total events	79		69			
Heterogeneity: Not applicable						
Test for overall effect: Z = 1.56 (P = 0.12)						
Test for subgroup differences: Chi² = 0.08, df = 1 (P = 0.77), I² = 0%						

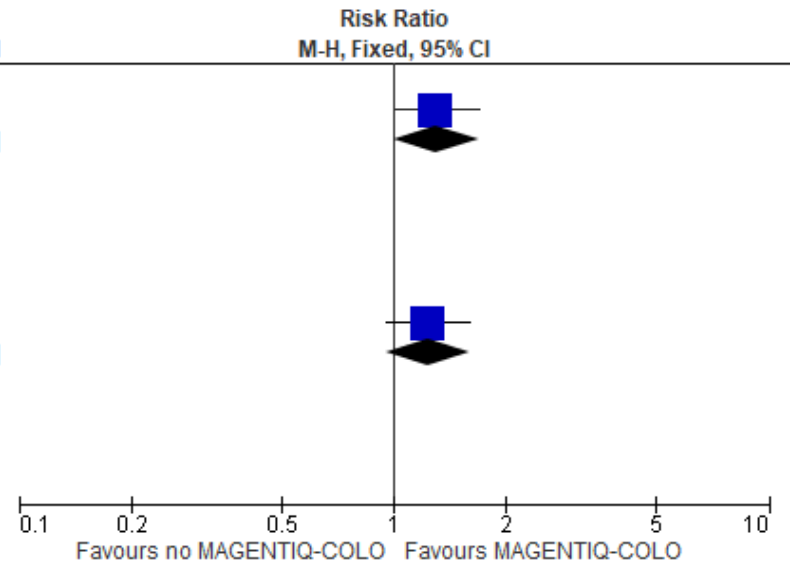

Abbreviations: ADR, adenoma detection rate; CI, confidence interval; M-H, Mantel-Haenszel.

Supplementary Figure 34. APC colonoscopy indication subgroup analysis with MAGENTIQ-COLO™ - within-trial subgroup data – reported at mean difference

Study or Subgroup	MAGENTIQ-COLO Mean	SD	Total	No MAGENTIQ-COLO Mean	SD	Total	Weight	Mean Difference IV, Fixed, 95% CI
**22.37.2 non-iFOBT screening**								
Maas 2024 (MAGENTIQ-COLO) - non-iFOBT screening	0.58	0.973	255	0.41	0.8173	259	100.0%	0.17 [0.01, 0.33]
**Subtotal (95% CI)**			**255**			**259**	**100.0%**	**0.17 [0.01, 0.33]**
Heterogeneity: Not applicable								
Test for overall effect: Z = 2.14 (P = 0.03)								
**22.37.3 Surveillance**								
Maas 2024 (MAGENTIQ-COLO) - surveillance	0.85	1.5536	194	0.63	1.1705	208	100.0%	0.22 [-0.05, 0.49]
**Subtotal (95% CI)**			**194**			**208**	**100.0%**	**0.22 [-0.05, 0.49]**
Heterogeneity: Not applicable								
Test for overall effect: Z = 1.59 (P = 0.11)								
Test for subgroup differences: Chi² = 0.10, df = 1 (P = 0.75), I² = 0%								

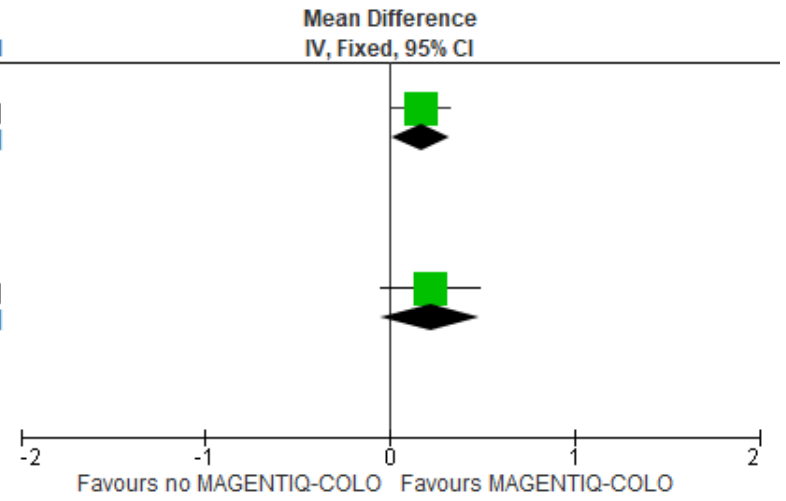

Abbreviations: APC, adenomas per colonoscopy; CI, confidence interval; IV, inverse variance; SD, standard deviation.

2.6 Endoscopist experience subgroup analyses

2.6.1 CAD EYE®

Supplementary Figure 35. ADR endoscopist experience subgroup analysis with CAD EYE®- majority in whole studies and within-trial subgroup data (trainee vs non-trainee)

Study or Subgroup	CAD EYE Events	Total	No CAD EYE Events	Total	Weight	Risk Ratio M-H, Random, 95% CI
**9.5.1 Majority trainee**						
Aniwan 2023	344	620	300	625	14.5%	1.16 [1.04, 1.29]
Miyaguchi 2024 - trainees	90	142	58	149	6.1%	1.63 [1.28, 2.06]
Tiankanon 2024	200	400	153	400	10.2%	1.31 [1.12, 1.53]
Yamaguchi 2024	66	113	72	118	7.1%	0.96 [0.77, 1.18]
**Subtotal (95% CI)**		**1275**		**1292**	**37.8%**	**1.23 [1.03, 1.46]**
Total events	700		583			
Heterogeneity: Tau² = 0.02; Chi² = 12.39, df = 3 (P = 0.006); I² = 76%						
Test for overall effect: Z = 2.34 (P = 0.02)						
**9.5.2 Majority non-trainee**						
Alali 2025	24	51	19	51	2.0%	1.26 [0.80, 2.00]
Desai 2024	238	509	224	522	12.0%	1.09 [0.95, 1.25]
Djinbachian 2024	113	229	91	238	7.3%	1.29 [1.05, 1.59]
Huneburg 2023	18	50	12	46	1.2%	1.38 [0.75, 2.54]
Miyaguchi 2024 - experts	145	258	116	251	9.3%	1.22 [1.02, 1.44]
Rondonotti 2022	217	405	179	395	11.5%	1.18 [1.03, 1.36]
Zimmermann-Fraedrich 2025	325	812	306	815	13.2%	1.07 [0.94, 1.20]
**Subtotal (95% CI)**		**2314**		**2318**	**56.4%**	**1.14 [1.07, 1.22]**
Total events	1080		947			
Heterogeneity: Tau² = 0.00; Chi² = 4.31, df = 6 (P = 0.63); I² = 0%						
Test for overall effect: Z = 4.06 (P < 0.0001)						
**9.5.3 Unclear**						
Hiratsuka 2025	36	48	33	46	5.8%	1.05 [0.82, 1.33]
**Subtotal (95% CI)**		**48**		**46**	**5.8%**	**1.05 [0.82, 1.33]**
Total events	36		33			
Heterogeneity: Not applicable						
Test for overall effect: Z = 0.36 (P = 0.72)						
**Total (95% CI)**		**3637**		**3656**	**100.0%**	**1.17 [1.10, 1.26]**
Total events	1816		1563			
Heterogeneity: Tau² = 0.01; Chi² = 18.38, df = 11 (P = 0.07); I² = 40%						
Test for overall effect: Z = 4.63 (P < 0.00001)						
Test for subgroup differences: Chi² = 1.17, df = 2 (P = 0.56), I² = 0%						

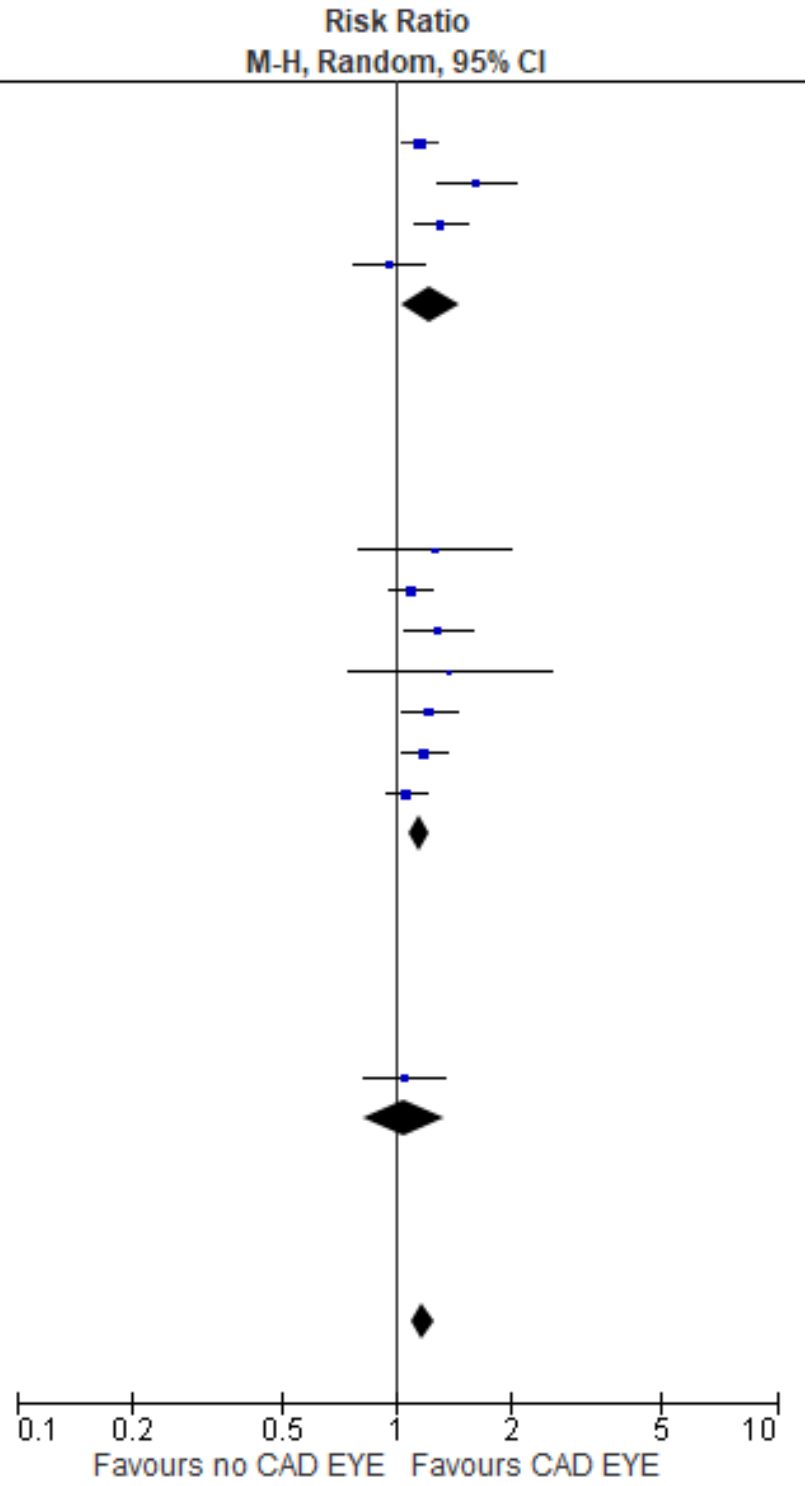

Abbreviations: ADR, adenoma detection rate; CI, confidence interval; M-H, Mantel-Haenszel.

Supplementary Figure 36. ADR endoscopist experience subgroup analysis with CAD EYE®-majority in whole studies and within-trial subgroup data (baseline ADR)

Study or Subgroup	log[Risk Ratio]	SE	Weight	Risk Ratio IV, Random, 95% CI
**16.7.1 Baseline average ADR ≤40%**				
Aniwan 2023	0.1484	0.0557	85.7%	1.16 [1.04, 1.29]
Rondonotti 2022 - baseline ADR ≤40%	0.1398	0.1365	14.3%	1.15 [0.88, 1.50]
**Subtotal (95% CI)**			**100.0%**	**1.16 [1.05, 1.28]**
Heterogeneity: Tau² = 0.00; Chi² = 0.00, df = 1 (P = 0.95); I² = 0%				
Test for overall effect: Z = 2.85 (P = 0.004)				
**16.7.2 Baseline average ADR >40%**				
Rondonotti 2022 - baseline ADR 41 to 45%	0.2231	0.1242	23.1%	1.25 [0.98, 1.59]
Rondonotti 2022 - baseline ADR ≥46%	0.1484	0.1295	21.2%	1.16 [0.90, 1.50]
Tiankanon 2024	0.27	0.0799	55.7%	1.31 [1.12, 1.53]
**Subtotal (95% CI)**			**100.0%**	**1.26 [1.12, 1.42]**
Heterogeneity: Tau² = 0.00; Chi² = 0.65, df = 2 (P = 0.72); I² = 0%				
Test for overall effect: Z = 3.91 (P < 0.0001)				
**16.7.3 Baseline ADR not reported - trainees permitted**				
Djinbachian 2024	0.2546	0.105	34.3%	1.29 [1.05, 1.58]
Miyaguchi 2024 - trainees	0.4886	0.1233	32.2%	1.63 [1.28, 2.08]
Yamaguchi 2024	-0.0408	0.1125	33.5%	0.96 [0.77, 1.20]
**Subtotal (95% CI)**			**100.0%**	**1.26 [0.94, 1.68]**
Heterogeneity: Tau² = 0.05; Chi² = 10.23, df = 2 (P = 0.006); I² = 80%				
Test for overall effect: Z = 1.56 (P = 0.12)				
**16.7.4 Baseline ADR not reported - experienced only**				
Alali 2025	0.2311	0.2357	2.6%	1.26 [0.79, 2.00]
Desai 2024	0.0862	0.0701	29.3%	1.09 [0.95, 1.25]
Huneburg 2023	0.3221	0.3111	1.5%	1.38 [0.75, 2.54]
Miyaguchi 2024 - experts	0.1989	0.0914	17.2%	1.22 [1.02, 1.46]
Nakashima 2023	0.2231	0.0938	16.4%	1.25 [1.04, 1.50]
Zimmermann-Fraedrich 2025	0.0677	0.0661	33.0%	1.07 [0.94, 1.22]
**Subtotal (95% CI)**			**100.0%**	**1.14 [1.06, 1.23]**
Heterogeneity: Tau² = 0.00; Chi² = 3.40, df = 5 (P = 0.64); I² = 0%				
Test for overall effect: Z = 3.40 (P = 0.0007)				
**16.7.5 Baseline ADR not reported - unclear if trainees permitted**				
Hiratsuka 2025	0.0488	0.1261	100.0%	1.05 [0.82, 1.34]
**Subtotal (95% CI)**			**100.0%**	**1.05 [0.82, 1.34]**
Heterogeneity: Not applicable				
Test for overall effect: Z = 0.39 (P = 0.70)				

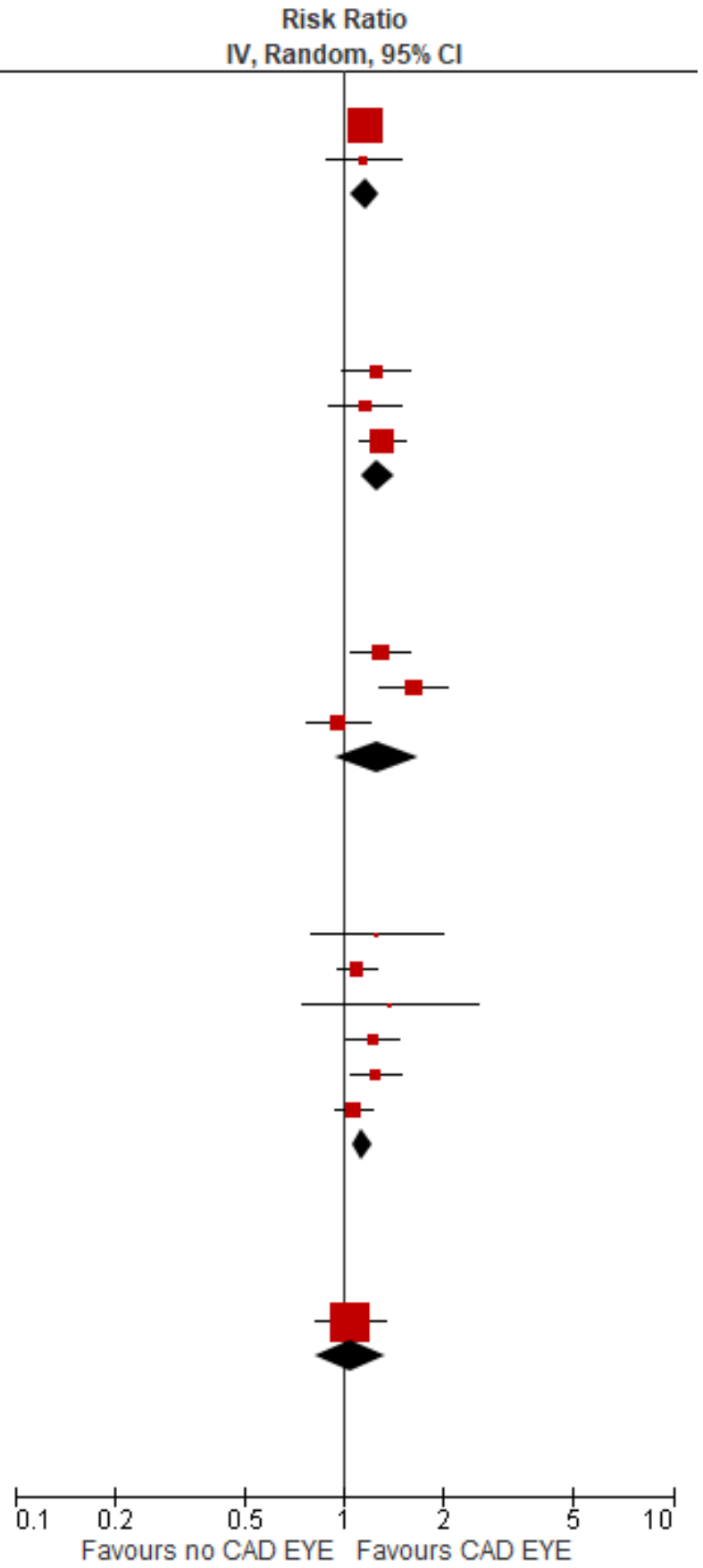

Test for subgroup differences: Chi² = 3.18, df = 4 (P = 0.53), I² = 0%

Abbreviations: ADR, adenoma detection rate; CI, confidence interval; IV, inverse variance; SE, standard error.

Supplementary Figure 37. APC endoscopist experience subgroup analysis with CAD EYE®-majority in whole studies (trainee vs non-trainee) – reported as mean difference

Study or Subgroup	CAD EYE Mean	CAD EYE SD	CAD EYE Total	No CAD EYE Mean	No CAD EYE SD	No CAD EYE Total	Weight	Mean Difference IV, Random, 95% CI
**16.10.1 Majority trainee**								
Aniwan 2023	1.21	1.65	620	0.97	1.54	625	17.7%	0.24 [0.06, 0.42]
Tiankanon 2024	1.04	1.63	400	0.66	1.05	400	15.4%	0.38 [0.19, 0.57]
Yamaguchi 2024	1.4	1.8	113	1.4	2	118	2.3%	0.00 [-0.49, 0.49]
**Subtotal (95% CI)**			**1133**			**1143**	**35.5%**	**0.28 [0.13, 0.43]**
Heterogeneity: Tau² = 0.00; Chi² = 2.51, df = 2 (P = 0.29); I² = 20%								
Test for overall effect: Z = 3.71 (P = 0.0002)								
**16.10.2 Majority non-trainee**								
Alali 2025	0.75	0.99	51	0.51	0.78	51	4.7%	0.24 [-0.11, 0.59]
Desai 2024	0.99	1.61	509	0.849	1.484	522	15.6%	0.14 [-0.05, 0.33]
Djinbachian 2024	0.86	1.2288	229	0.75	1.3313	238	10.3%	0.11 [-0.12, 0.34]
Miyaguchi 2024	1.31	1.6277	400	0.94	1.4243	400	12.4%	0.37 [0.16, 0.58]
Nakashima 2023	1.21	1.36	207	1.01	1.5	208	7.3%	0.20 [-0.08, 0.48]
Rondonotti 2022	1.126	1.54	405	0.904	1.32	395	14.1%	0.22 [0.02, 0.42]
**Subtotal (95% CI)**			**1801**			**1814**	**64.5%**	**0.21 [0.12, 0.30]**
Heterogeneity: Tau² = 0.00; Chi² = 3.46, df = 5 (P = 0.63); I² = 0%								
Test for overall effect: Z = 4.46 (P < 0.00001)								
**Total (95% CI)**			**2934**			**2957**	**100.0%**	**0.24 [0.16, 0.31]**
Heterogeneity: Tau² = 0.00; Chi² = 6.82, df = 8 (P = 0.56); I² = 0%								
Test for overall effect: Z = 6.24 (P < 0.00001)								
Test for subgroup differences: Chi² = 0.59, df = 1 (P = 0.44), I² = 0%								

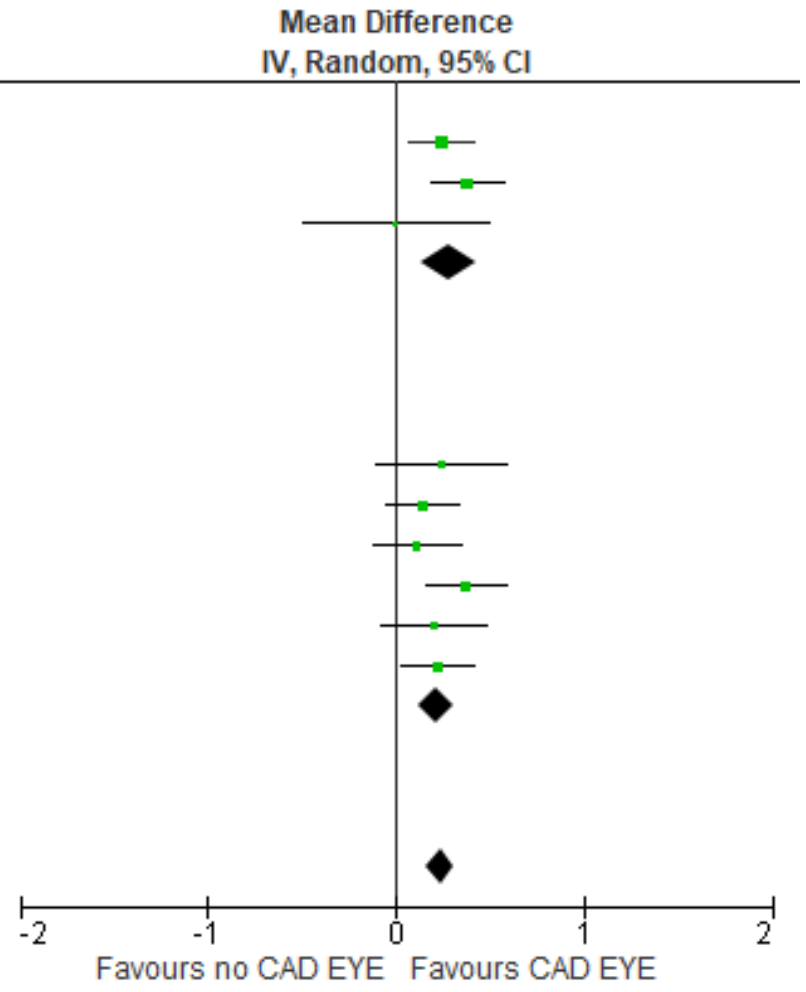

Abbreviations: APC, adenomas per colonoscopy; CI, confidence interval; IV, inverse variance; SD, standard deviation.

Supplementary Figure 38. APC endoscopist experience subgroup analysis with CAD EYE®-majority in whole studies and within-trial subgroup data (baseline ADR) – reported as mean difference

Study or Subgroup	CAD EYE Mean	CAD EYE SD	CAD EYE Total	No CAD EYE Mean	No CAD EYE SD	No CAD EYE Total	Weight	Mean Difference IV, Random, 95% CI
**16.12.1 Baseline average ADR ≤40%**								
Aniwan 2023	1.21	1.65	620	0.97	1.54	625	17.7%	0.24 [0.06, 0.42]
**Subtotal (95% CI)**			**620**			**625**	**17.7%**	**0.24 [0.06, 0.42]**
Heterogeneity: Not applicable								
Test for overall effect: Z = 2.65 (P = 0.008)								
**16.12.2 Baseline average ADR >40%**								
Tiankanon 2024	1.04	1.63	400	0.66	1.05	400	15.4%	0.38 [0.19, 0.57]
**Subtotal (95% CI)**			**400**			**400**	**15.4%**	**0.38 [0.19, 0.57]**
Heterogeneity: Not applicable								
Test for overall effect: Z = 3.92 (P < 0.0001)								
**16.12.3 Baseline ADR not reported - trainees permitted**								
Djinbachian 2024	0.86	1.2288	229	0.75	1.3313	238	10.3%	0.11 [-0.12, 0.34]
Miyaguchi 2024	1.31	1.6277	400	0.94	1.4243	400	12.4%	0.37 [0.16, 0.58]
Yamaguchi 2024	1.4	1.8	113	1.4	2	118	2.3%	0.00 [-0.49, 0.49]
**Subtotal (95% CI)**			**742**			**756**	**25.1%**	**0.21 [-0.01, 0.42]**
Heterogeneity: Tau² = 0.02; Chi² = 3.55, df = 2 (P = 0.17); I² = 44%								
Test for overall effect: Z = 1.90 (P = 0.06)								
**16.12.4 Baseline ADR not reported - experienced only**								
Alali 2025	0.75	0.99	51	0.51	0.78	51	4.7%	0.24 [-0.11, 0.59]
Desai 2024	0.99	1.61	509	0.849	1.484	522	15.6%	0.14 [-0.05, 0.33]
Nakashima 2023	1.21	1.36	207	1.01	1.5	208	7.3%	0.20 [-0.08, 0.48]
Rondonotti 2022	1.126	1.54	405	0.904	1.32	395	14.1%	0.22 [0.02, 0.42]
**Subtotal (95% CI)**			**1172**			**1176**	**41.7%**	**0.19 [0.07, 0.31]**
Heterogeneity: Tau² = 0.00; Chi² = 0.44, df = 3 (P = 0.93); I² = 0%								
Test for overall effect: Z = 3.22 (P = 0.001)								
**Total (95% CI)**			**2934**			**2957**	**100.0%**	**0.24 [0.16, 0.31]**
Heterogeneity: Tau² = 0.00; Chi² = 6.82, df = 8 (P = 0.56); I² = 0%								
Test for overall effect: Z = 6.24 (P < 0.00001)								
Test for subgroup differences: Chi² = 2.89, df = 3 (P = 0.41), I² = 0%								

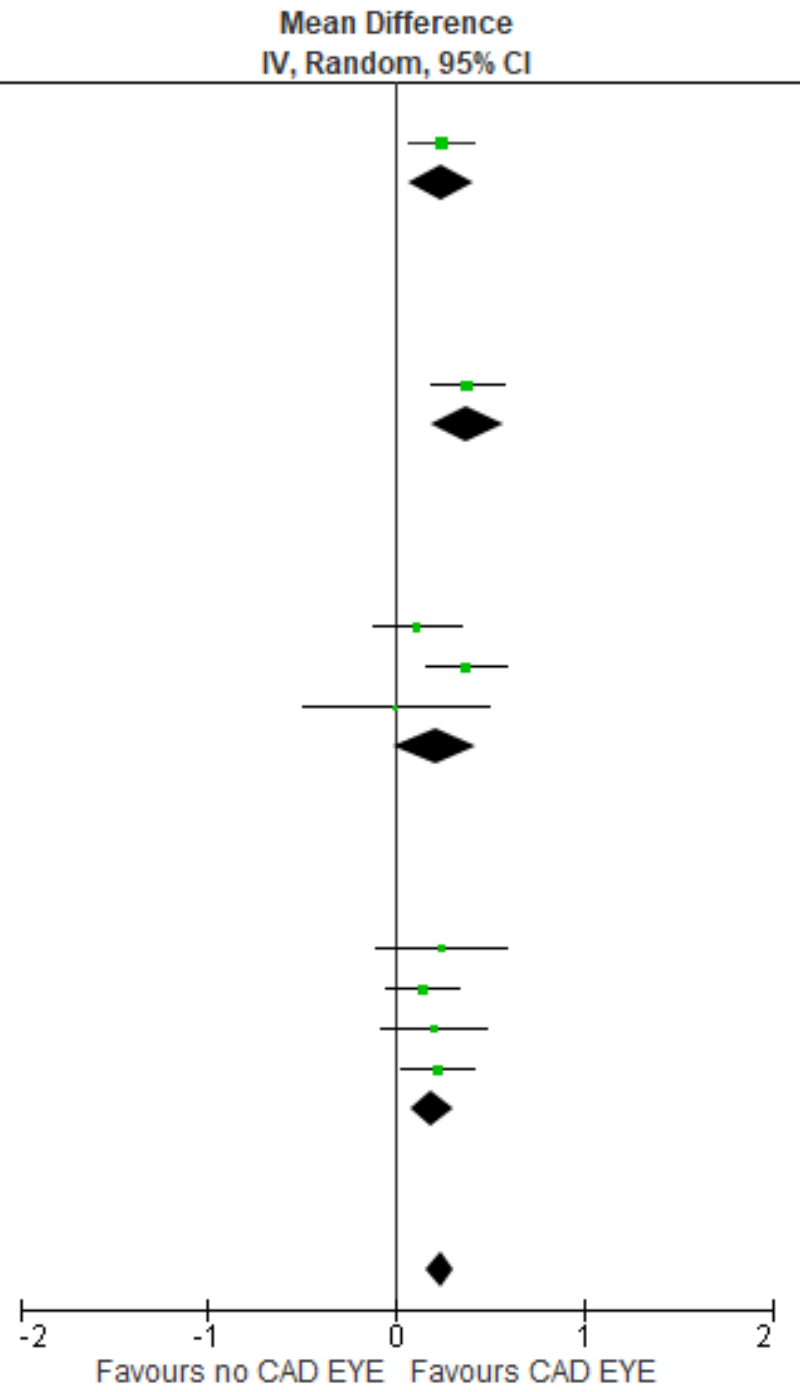

Abbreviations: ADR, adenoma detection rate; APC, adenomas per colonoscopy; CI, confidence interval; IV, inverse variance; SD, standard deviation.

2.6.2 ENDO-AID™

Supplementary Figure 39. ADR endoscopist experience subgroup analysis with ENDO-AID™ - trainee inclusion permitted vs not permitted

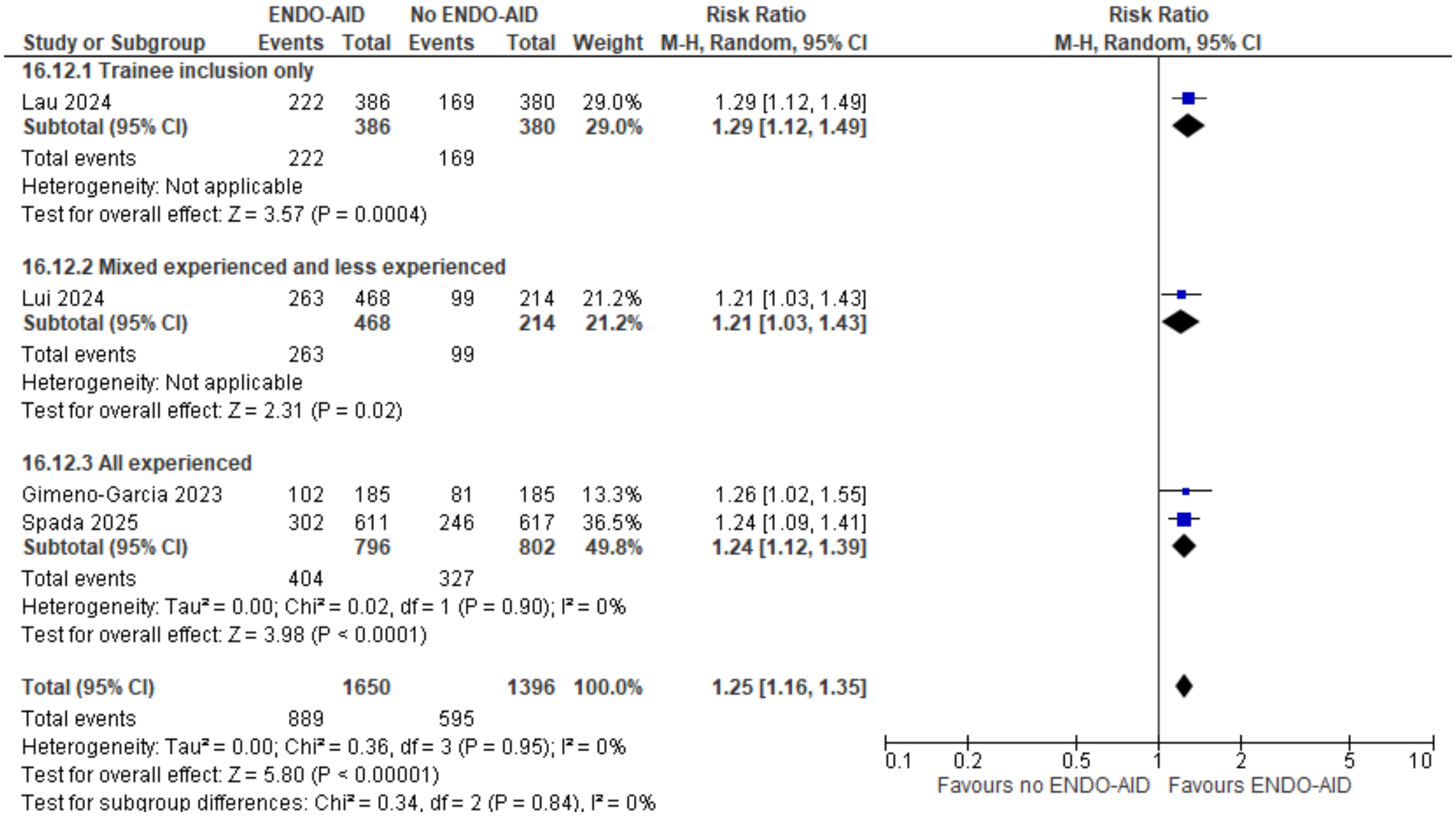

Abbreviations: ADR, adenoma detection rate; CI, confidence interval; M-H, Mantel-Haenszel.

Supplementary Figure 40. APC endoscopist experience subgroup analysis with ENDO-AID™ - trainee inclusion permitted vs not permitted – reported as mean difference

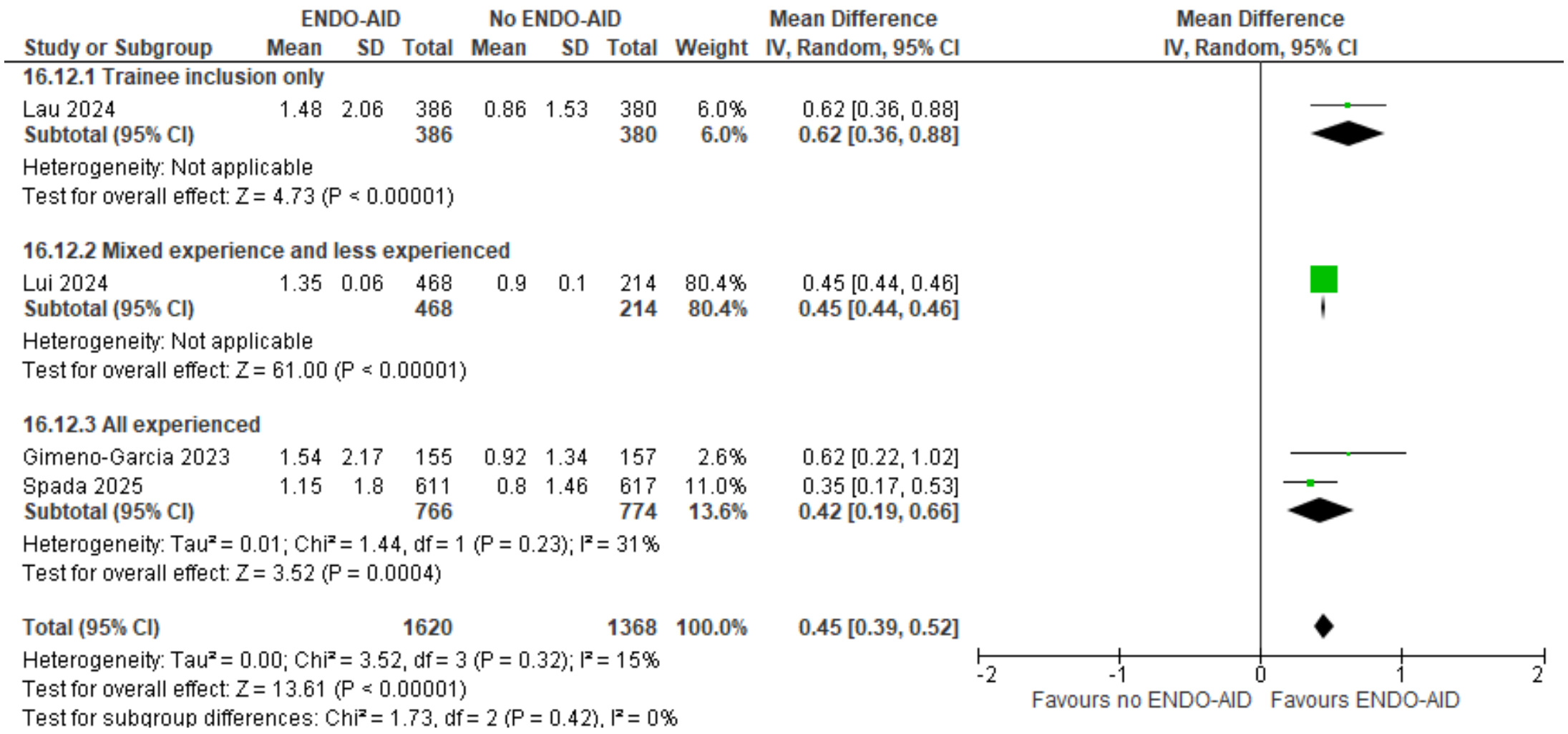

Abbreviations: APC, adenomas per colonoscopy; CI, confidence interval; IV, inverse variance; SD, standard deviation.

2.6.3 ENDOANGEL®

Supplementary Figure 41. ADR endoscopist experience subgroup analysis with ENDOANGEL® - novices vs experienced endoscopists

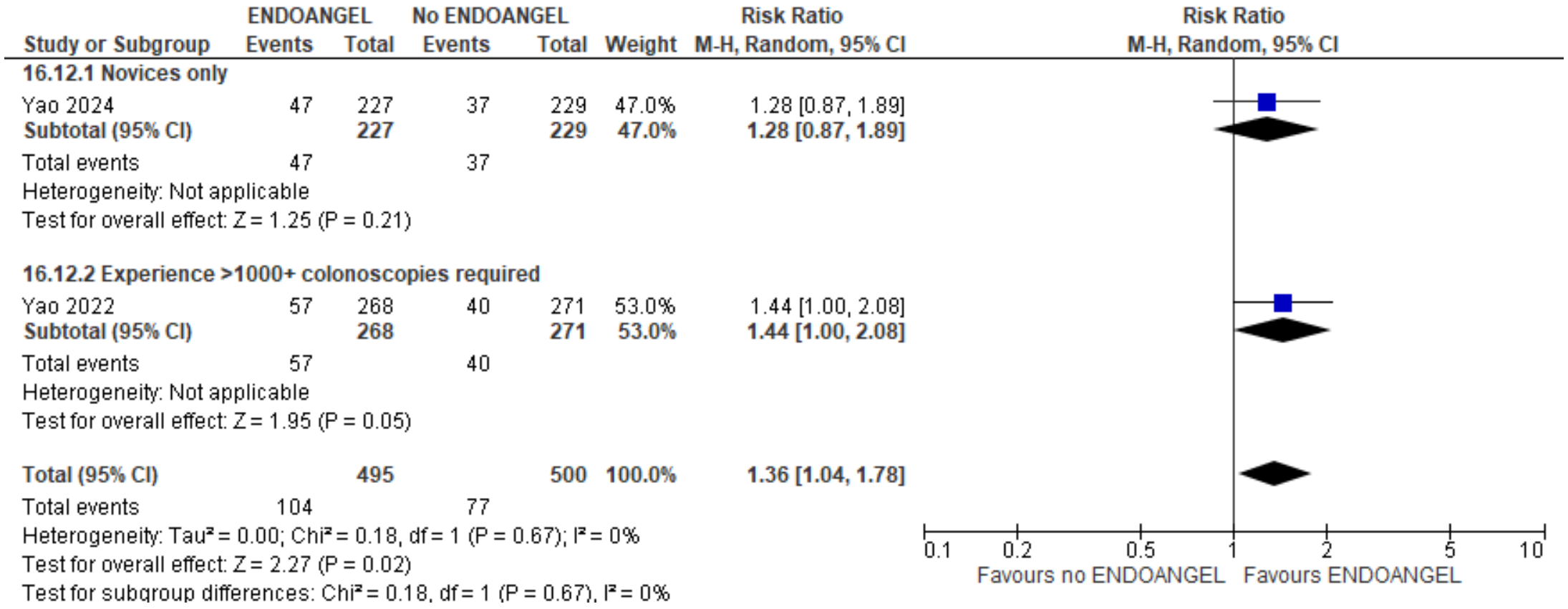

Abbreviations: ADR, adenoma detection rate; CI, confidence interval; M-H, Mantel-Haenszel.

Supplementary Figure 42. APC endoscopist experience subgroup analysis with ENDOANGEL® - novices vs experienced endoscopists – reported as mean difference

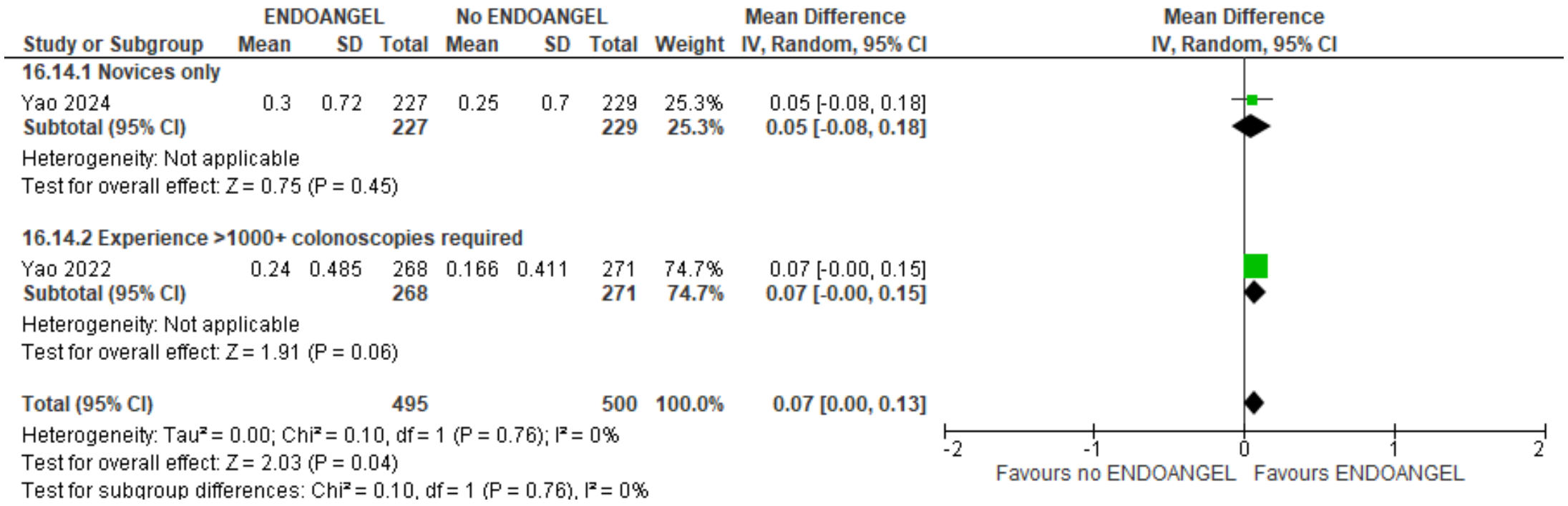

Abbreviations: APC, adenomas per colonoscopy; CI, confidence interval; IV, inverse variance; SD, standard deviation.

2.6.4 EndoScreener®

Supplementary Figure 43. ADR endoscopist experience subgroup analysis with EndoScreener® - mixed experience vs experienced only

Study or Subgroup	EndoScreener Events	EndoScreener Total	No EndoScreener Events	No EndoScreener Total	Weight	Risk Ratio M-H, Random, 95% CI
**16.12.1 Experienced only**						
Glissen Brown 2022	57	113	48	110	11.0%	1.16 [0.87, 1.53]
Wang 2020 (effect of a deep)	165	484	134	478	23.8%	1.22 [1.01, 1.47]
Wang 2020 (lower adenoma miss)	64	184	49	185	9.0%	1.31 [0.96, 1.79]
Wang 2023	164	636	150	625	23.2%	1.07 [0.89, 1.30]
**Subtotal (95% CI)**		**1417**		**1398**	**67.0%**	**1.17 [1.04, 1.31]**
Total events	450		381			
Heterogeneity: Tau² = 0.00; Chi² = 1.45, df = 3 (P = 0.69); I² = 0%						
Test for overall effect: Z = 2.67 (P = 0.008)						
**16.12.2 Any experience included**						
Liu 2020	114	393	83	397	14.3%	1.39 [1.08, 1.77]
Wang 2019	152	522	109	536	18.7%	1.43 [1.16, 1.77]
**Subtotal (95% CI)**		**915**		**933**	**33.0%**	**1.41 [1.20, 1.66]**
Total events	266		192			
Heterogeneity: Tau² = 0.00; Chi² = 0.04, df = 1 (P = 0.85); I² = 0%						
Test for overall effect: Z = 4.19 (P < 0.0001)						
**Total (95% CI)**		**2332**		**2331**	**100.0%**	**1.24 [1.13, 1.37]**
Total events	716		573			
Heterogeneity: Tau² = 0.00; Chi² = 5.09, df = 5 (P = 0.41); I² = 2%						
Test for overall effect: Z = 4.55 (P < 0.00001)						
Test for subgroup differences: Chi² = 3.60, df = 1 (P = 0.06), I² = 72.2%						

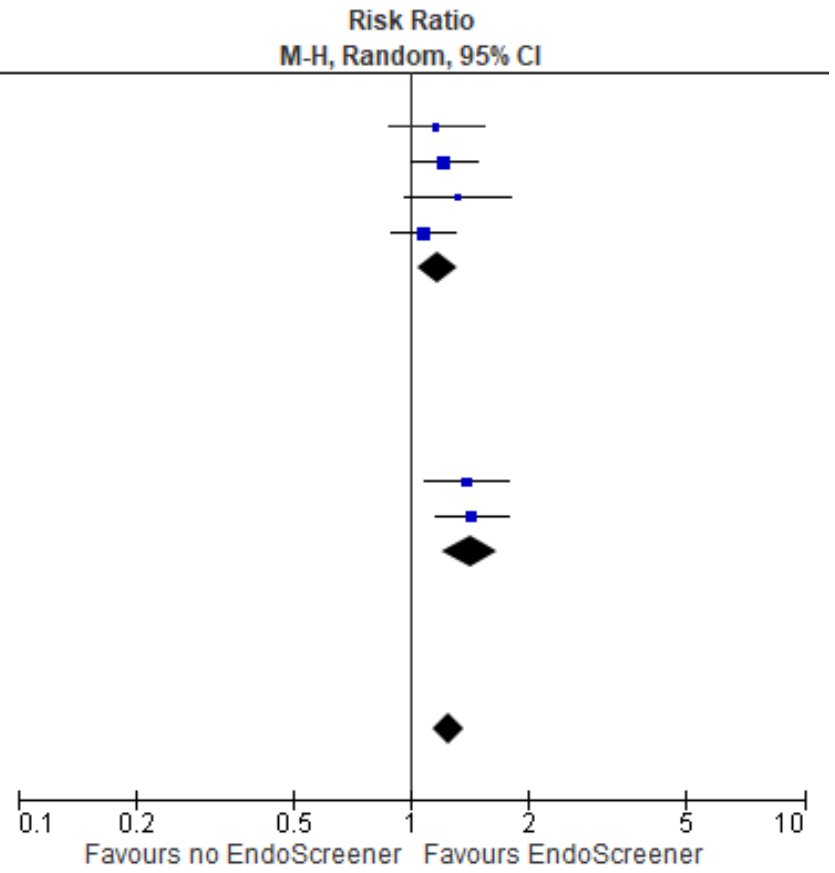

Abbreviations: ADR, adenoma detection rate; CI, confidence interval; M-H, Mantel-Haenszel.

Supplementary Figure 44. APC as an IRR endoscopist experience subgroup analysis with EndoScreener® - mixed experience vs experienced only

Study or Subgroup	log[Rate Ratio]	SE	Weight	Rate Ratio IV, Random, 95% CI
**16.14.1 Experienced only**				
Glissen Brown 2022	0.2758	0.1364	13.5%	1.32 [1.01, 1.72]
Wang 2020 (effect of a deep)	0.4219	0.097	19.6%	1.52 [1.26, 1.84]
Wang 2020 (lower adenoma miss)	0.549	0.1522	11.7%	1.73 [1.28, 2.33]
Wang 2023	0.1928	0.093	20.4%	1.21 [1.01, 1.46]
**Subtotal (95% CI)**			**65.3%**	**1.41 [1.21, 1.64]**
Heterogeneity: Tau² = 0.01; Chi² = 5.30, df = 3 (P = 0.15); I² = 43%				
Test for overall effect: Z = 4.45 (P < 0.00001)				
**16.14.2 Any experience included**				
Liu 2020	0.493	0.1208	15.7%	1.64 [1.29, 2.07]
Wang 2019	0.5421	0.0999	19.1%	1.72 [1.41, 2.09]
**Subtotal (95% CI)**			**34.7%**	**1.69 [1.45, 1.96]**
Heterogeneity: Tau² = 0.00; Chi² = 0.10, df = 1 (P = 0.75); I² = 0%				
Test for overall effect: Z = 6.78 (P < 0.00001)				
**Total (95% CI)**			**100.0%**	**1.50 [1.32, 1.70]**
Heterogeneity: Tau² = 0.01; Chi² = 9.41, df = 5 (P = 0.09); I² = 47%				
Test for overall effect: Z = 6.37 (P < 0.00001)				
Test for subgroup differences: Chi² = 2.73, df = 1 (P = 0.10), I² = 63.4%				

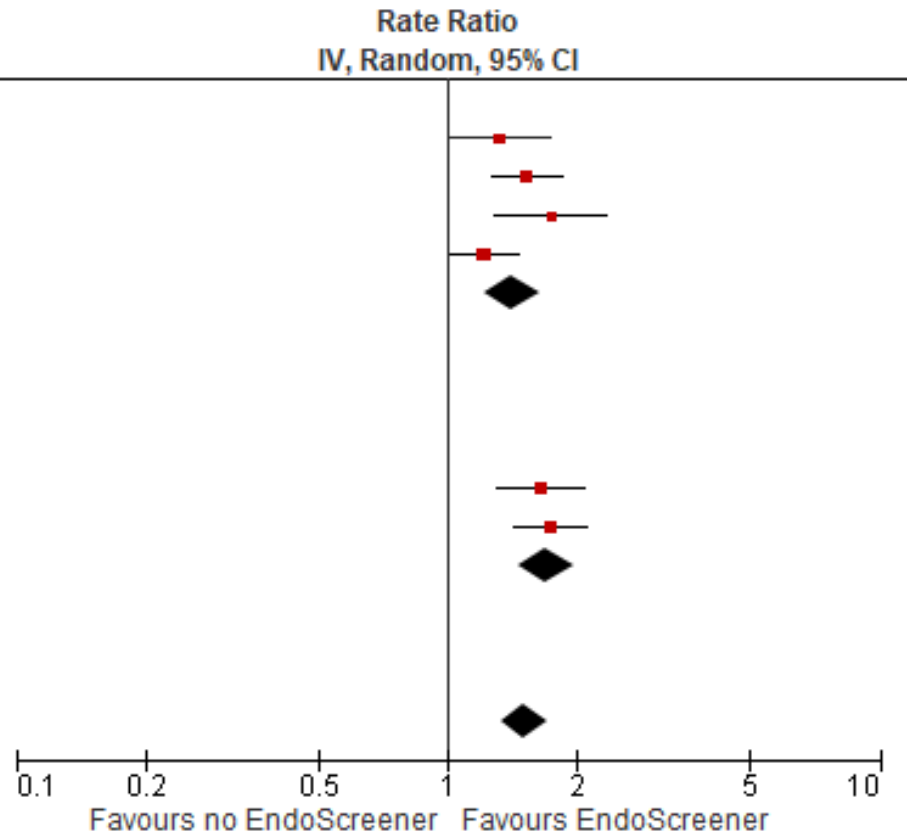

Abbreviations: APC, adenomas per colonoscopy; CI, confidence interval; IRR, incidence rate ratio; IV, inverse variance; SE, standard error.

2.6.5 GI Genius™

Supplementary Figure 45. ADR endoscopist experience subgroup analysis with GI Genius™ - screening accreditation or mixed

Study or Subgroup	GI Genius Events	GI Genius Total	No GI Genius Events	No GI Genius Total	Weight	Risk Ratio M-H, Random, 95% CI
**16.12.1 Screening endoscopist accreditation required or observed**						
Ahmad 2023	220	308	134	306	11.2%	1.63 [1.41, 1.89]
Mangas-Sanjuan 2023	1033	1610	990	1603	14.2%	1.04 [0.99, 1.10]
Repici 2020	187	341	139	344	10.7%	1.36 [1.16, 1.59]
**Subtotal (95% CI)**		**2259**		**2253**	**36.1%**	**1.31 [0.97, 1.77]**
Total events	1440		1263			
Heterogeneity: Tau² = 0.07; Chi² = 39.47, df = 2 (P < 0.00001); I² = 95%						
Test for overall effect: Z = 1.77 (P = 0.08)						
**16.12.2 Mixed experience, no requirement for screening accreditation but no trainees**						
Karsenti 2023	376	1003	341	1012	12.2%	1.11 [0.99, 1.25]
Ortiz 2024	70	214	79	216	7.4%	0.89 [0.69, 1.16]
Repici 2022	176	330	147	330	10.8%	1.20 [1.02, 1.40]
Seager 2024	555	980	477	986	13.3%	1.17 [1.08, 1.27]
Thiruvengadam 2024	234	550	189	550	11.0%	1.24 [1.06, 1.44]
Wallace 2022	72	116	70	114	9.1%	1.01 [0.82, 1.24]
**Subtotal (95% CI)**		**3193**		**3208**	**63.9%**	**1.14 [1.06, 1.22]**
Total events	1483		1303			
Heterogeneity: Tau² = 0.00; Chi² = 6.73, df = 5 (P = 0.24); I² = 26%						
Test for overall effect: Z = 3.70 (P = 0.0002)						
**Total (95% CI)**		**5452**		**5461**	**100.0%**	**1.18 [1.07, 1.30]**
Total events	2923		2566			
Heterogeneity: Tau² = 0.02; Chi² = 46.40, df = 8 (P < 0.00001); I² = 83%						
Test for overall effect: Z = 3.21 (P = 0.001)						
Test for subgroup differences: Chi² = 0.84, df = 1 (P = 0.36), I² = 0%						

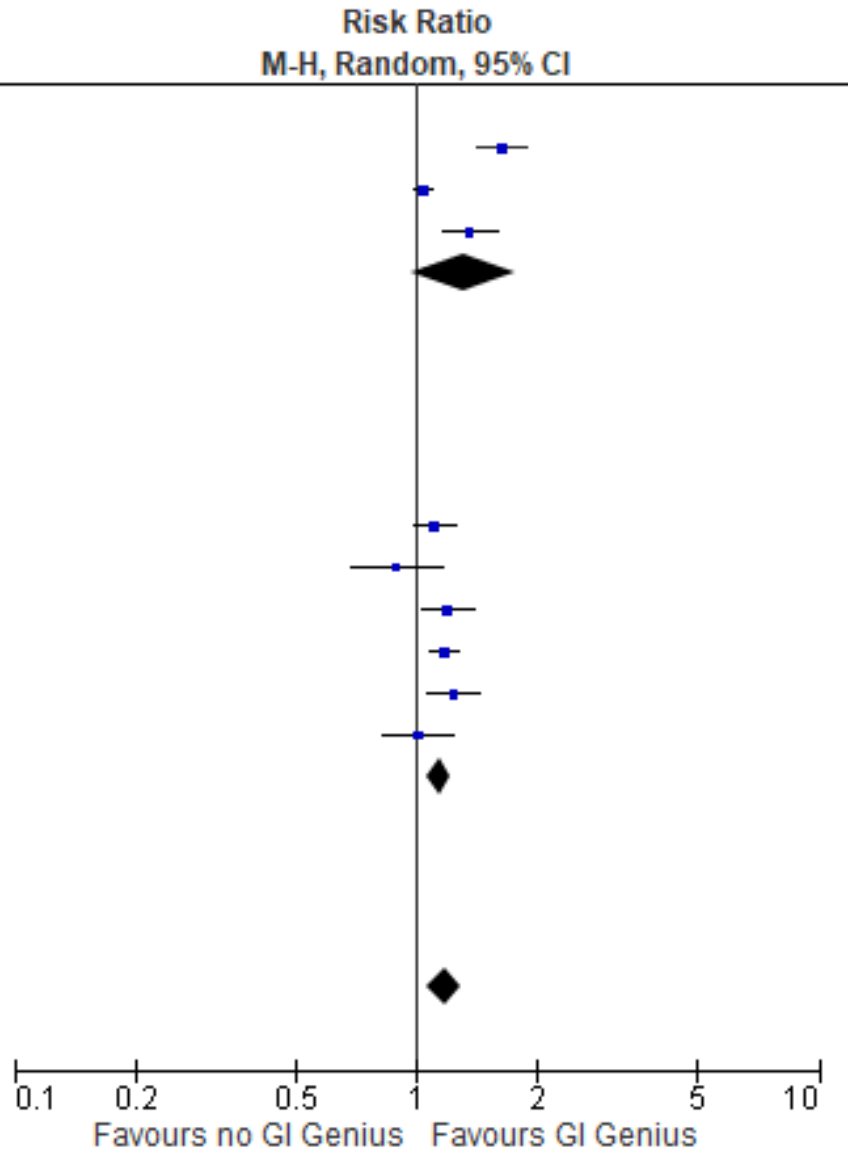

Abbreviations: ADR, adenoma detection rate; CI, confidence interval; M-H, Mantel-Haenszel.

Supplementary Figure 46. APC endoscopist experience subgroup analysis with GI Genius™ - screening accreditation or mixed, with or without trainees – reported as mean difference

Study or Subgroup	GI Genius Mean	GI Genius SD	GI Genius Total	No GI Genius Mean	No GI Genius SD	No GI Genius Total	Weight	Mean Difference IV, Random, 95% CI
**16.14.1 Screening endoscopist accreditation required or observed**								
Ahmad 2023	2.4	2.9	308	2.1	2.6	306	2.3%	0.30 [-0.14, 0.74]
Mangas-Sanjuan 2023	1.78	2.38	1610	1.59	2.2	1603	17.2%	0.19 [0.03, 0.35]
Repici 2020	1.07	1.54	341	0.71	1.2	344	10.1%	0.36 [0.15, 0.57]
**Subtotal (95% CI)**			**2259**			**2253**	**29.6%**	**0.26 [0.14, 0.38]**
Heterogeneity: Tau² = 0.00; Chi² = 1.68, df = 2 (P = 0.43); I² = 0%								
Test for overall effect: Z = 4.16 (P < 0.0001)								
**16.14.2 Mixed experience, no requirement for screening accreditation but no trainees**								
Karsenti 2023	0.89	1.49	1003	0.71	1.3	1012	29.0%	0.18 [0.06, 0.30]
Ortiz 2024	0.64	1.57	214	0.64	1.17	216	6.3%	0.00 [-0.26, 0.26]
Repici 2022	1.26	1.82	330	1.04	1.75	330	5.8%	0.22 [-0.05, 0.49]
Seager 2024	1.56	2.82	1001	1.21	1.91	1009	9.7%	0.35 [0.14, 0.56]
Thiruvengadam 2024	0.89	1.46	550	0.6	1.12	550	18.3%	0.29 [0.14, 0.44]
Wallace 2022	1.79	2.63	116	1.46	1.7	114	1.3%	0.33 [-0.24, 0.90]
**Subtotal (95% CI)**			**3214**			**3231**	**70.4%**	**0.22 [0.14, 0.31]**
Heterogeneity: Tau² = 0.00; Chi² = 5.52, df = 5 (P = 0.36); I² = 9%								
Test for overall effect: Z = 5.12 (P < 0.00001)								
**Total (95% CI)**			**5473**			**5484**	**100.0%**	**0.23 [0.17, 0.30]**
Heterogeneity: Tau² = 0.00; Chi² = 7.42, df = 8 (P = 0.49); I² = 0%								
Test for overall effect: Z = 6.92 (P < 0.00001)								
Test for subgroup differences: Chi² = 0.20, df = 1 (P = 0.66), I² = 0%								

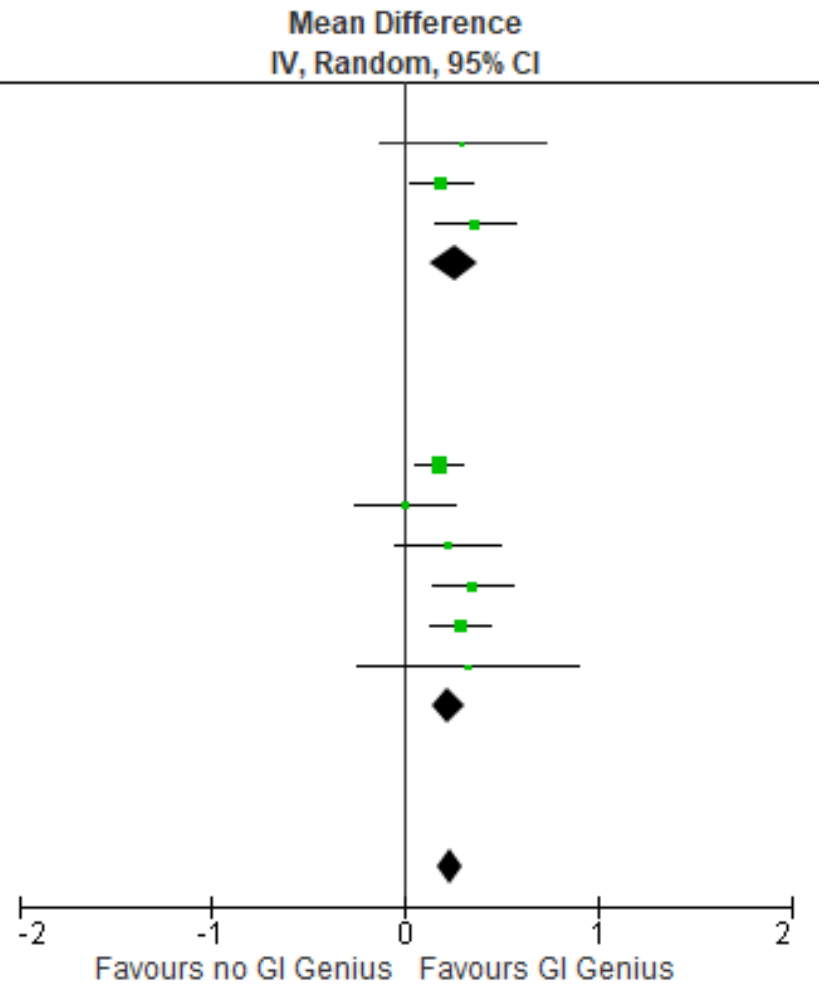

Abbreviations: APC, adenomas per colonoscopy; CI, confidence interval; IV, inverse variance; SD, standard deviation.

References

1. Health Technology Wales. Artificial Intelligence (AI)-assisted endoscopy in the detection of gastrointestinal cancer and pre-cancerous lesions, 2024. Available from: https://healthtechnology.wales/reports-guidance/ai-assisted-endoscopy-for-gastrointestinal-cancer/. Date accessed: Feb 2025.

2. Mourad Ouzzani HH, Zbys Fedorowicz, and Ahmed Elmagarmid. Rayyan—a web and mobile app forsystematic reviews. *Systematic Reviews* 2016; **5**.

3. Review Manager (RevMan). Version 5.3.5. The Cochrane Collaboration, 2014. Available at revman.cochrane.org.

4. Higgins J, Thomas J, Chandler J, Cumpston M, Li T, Page M, et al. Cochrane Handbook for Systematic Reviews of Interventions version 6.5 (updated August 2024). Cochrane, 2024. Available from: www.training.cochrane.org/handbook. Date accessed: Feb 26.

5. MedCalc Software Ltd. Comparison of two rates. Version 23.1.5., 2025. Available from: https://www.medcalc.org/calc/rate_comparison.php. Date accessed: Jan 2025.

6. National Institute for Health and Care Excellence (NICE). Artificial intelligence (AI) technologies to help detect or characterise colorectal polyps. HealthTech Guidance [HTG773]: Final Scope, 2024. Available from: https://www.nice.org.uk/guidance/htg773/history. Date accessed: Mar 26.

7. Aniwan S, Mekritthikrai K, Kerr SJ, Tiankanon K, Vandaungden K, Sritunyarat Y, et al. Computer-aided detection, mucosal exposure device, their combination, and standard colonoscopy for adenoma detection: a randomized controlled trial. *Gastrointestinal Endoscopy* 2023; **97**: 507-16.

8. Lui TK-L, Lam CP-M, To EW-P, Ko MK-L, Tsui VWM, Liu KS-H, et al. Endocuff With or Without Artificial Intelligence-Assisted Colonoscopy in Detection of Colorectal Adenoma: A Randomized Colonoscopy Trial. *Official journal of the American College of Gastroenterology | ACG* 2024; **119**: 1318-25.

9. StatsToDo. StatsToDo: Combining n, mean, and Standard Deviation from Multiple Groups. Available from: https://www.statstodo.com/CombineMeansSDs.php. Date accessed: Nov 2024.

10. BMJ Technology Assessment Group (BMJ-TAG). Artificial intelligence (AI) technologies to help detect or characterise colorectal polyps. HealthTech Guidance [HTG773]: Final protocol v2, 2024. Available from: https://www.nice.org.uk/guidance/htg773/history. Date accessed: Mar 2026.

11. National Institute for Health and Care Excellence (NICE). Artificial intelligence (AI) technologies to help detect or characterise colorectal polyps. HealthTech guidance [HTG773], 2026. Available from: https://www.nice.org.uk/guidance/HTG773. Date accessed: Mar 26.

12. Strapko A, Syed T, Baratta A, Strapko AM, Alexander K. P3030 - Artificial Intelligence (CAD-E)-Assisted Colonoscopy Helps Increase Adenoma Detection Rate (ADR) in the Afternoon

Session. *ACG 2023 Annual Scientific Meeting Abstracts*. 2023. p. ACG 2023 Annual Scientific Meeting; 24 October 2023; Vancouver, BC, Canada: American College of Gastroenterology.

13. EndoSoft®. Argus-PD-LC. Instructions for Use. 2023.

14. Desai M, Ausk K, Brannan D, Chhabra R, Chan W, Chiorean M, et al. Use of a Novel Artificial Intelligence System Leads to the Detection of Significantly Higher Number of Adenomas During Screening and Surveillance Colonoscopy: Results From a Large, Prospective, US Multicenter, Randomized Clinical Trial. *The American journal of gastroenterology* 2024; **119**: 1383-91.

15. Djinbachian R, Taghiakbari M, Barkun A, Medawar E, Alj A, Sidani S, et al. Optimized computer-assisted technique for increasing adenoma detection during colonoscopy: a randomized controlled trial. *Surgical Endoscopy* 2024; **39**: 1120-7.

16. Hiratsuka Y, Hisabe T, Ohtsu K, Yasaka T, Takeda K, Miyaoka M, et al. Evaluation of Artificial Intelligence: Computer-aided Detection of Colorectal Polyps. *Journal of the anus, rectum and colon* 2025; **9**: 79-87.

17. Huneburg R, Bucksch K, Schmeiser F, Heling D, Marwitz T, Aretz S, et al. Real-time use of artificial intelligence (CADEYE) in colorectal cancer surveillance of patients with Lynch syndrome-A randomized controlled pilot trial (CADLY). *United European gastroenterology journal* 2023; **11**: 60-8.

18. Miyaguchi K, Tsuzuki Y, Hirooka N, Matsumoto H, Ohgo H, Nakamoto H, et al. Linked-color imaging with or without artificial intelligence for adenoma detection: a randomized trial. *Endoscopy* 2024; **56**: 376-83.

19. Nakashima H, Kitazawa N, Fukuyama C, Kawachi H, Kawahira H, Momma K, et al. Clinical Evaluation of Computer-Aided Colorectal Neoplasia Detection Using a Novel Endoscopic Artificial Intelligence: A Single-Center Randomized Controlled Trial. *Digestion* 2023; **104**: 193-201.

20. Rondonotti E, Di Paolo D, Rizzotto ER, Alvisi C, Buscarini E, Spadaccini M, et al. Efficacy of a computer-aided detection system in a fecal immunochemical test-based organized colorectal cancer screening program: a randomized controlled trial (AIFIT study). *Endoscopy* 2022; **54**: 1171-9.

21. Scholer J, Alavanja M, de Lange T, Yamamoto S, Hedenstrom P, Varkey J. Impact of AI-aided colonoscopy in clinical practice: a prospective randomised controlled trial. *BMJ open gastroenterology* 2024; **11**: e001247.

22. Tiankanon K, Aniwan S, Kerr SJ, Mekritthikrai K, Kongtab N, Wisedopas N, et al. Improvement of adenoma detection rate by two computer-aided colonic polyp detection systems in high adenoma detectors: a randomized multicenter trial. *Endoscopy* 2024; **56**: 273-82.

23. Yamaguchi D, Shimoda R, Miyahara K, Yukimoto T, Sakata Y, Takamori A, et al. Impact of an artificial intelligence-aided endoscopic diagnosis system on improving endoscopy quality for trainees in colonoscopy: Prospective, randomized, multicenter study. *Digestive endoscopy : official journal of the Japan Gastroenterological Endoscopy Society* 2024; **36**: 40-8.

24. Zimmermann-Fraedrich K, Sehner S, Rosch T, Aschenbeck J, Schubert S, Liceni T, et al. No Effect of Computer Aided Diagnosis on Colonoscopic Adenoma Detection in a Large Pragmatic Multicenter Randomized Study. *American Journal of Gastroenterology* 2025: 10.14309/ajg.0000000000003500.

25. Djinbachian R, Haumesser C, Taghiakbari M, Pohl H, Barkun A, Sidani S, et al. Autonomous Artificial Intelligence vs Artificial Intelligence-Assisted Human Optical Diagnosis of Colorectal Polyps: A Randomized Controlled Trial. *Gastroenterology* 2024; **167**: 392-9.e2.

26. Rondonotti E, Hassan C, Tamanini G, Antonelli G, Andrisani G, Leonetti G, et al. Artificial intelligence-assisted optical diagnosis for the resect-and-discard strategy in clinical practice: the Artificial intelligence BLI Characterization (ABC) study. *Endoscopy* 2023; **55**: 14-22.

27. Sato K, Kuramochi M, Tsuchiya A, Yamaguchi A, Hosoda Y, Yamaguchi N, et al. Multicentre study to assess the performance of an artificial intelligence instrument to support qualitative diagnosis of colorectal polyps. *BMJ Open Gastroenterology* 2024; **11**: e001553.

28. Taghiakbari M, Rex DK, Pohl H, Djinbachian R, Huang F, Hassan C, et al. Pragmatic Resect and Discard Implementation Using Computer-Assisted Optical Polyp Diagnosis. *Gastroenterology* 2025; **168**: 154-6.e2.

29. Cassinotti A, Zadro V, Parravicini M, Ferraris M, Balzarini M, Sessa F, et al. LCI/BLI chromoendoscopy plus CAD-EYE artificial intelligence for the detection and characterization of endoscopic visible lesions in ulcerative colitis. *Journal of Crohn's and Colitis* 2023; **17**: i291.

30. Alali AA, Alhashmi A, Alotaibi N, Ali N, Alali M, Alfadhli A. Artificial Intelligence for Adenoma and Polyp Detection During Screening and Surveillance Colonoscopy: A Randomized-Controlled Trial. *Journal of clinical medicine* 2025; **14**: 581.

31. Maas MHJ, Rath T, Spada C, Soons E, Forbes N, Kashin S, et al. A computer-aided detection system in the everyday setting of diagnostic, screening, and surveillance colonoscopy: an international, randomized trial. *Endoscopy* 2024; **56**: 843-50.

32. Lopez-Serrano A, Voces A, Lorente JR, Santonja FJ, Algarra A, Latorre P, et al. Artificial intelligence for dysplasia detection during surveillance colonoscopy in patients with ulcerative colitis: A cross-sectional, non-inferiority, diagnostic test comparison study. *Gastroenterologia y hepatologia* 2024; **48**: 502210.

33. Gimeno-Garcia AZ, Hernandez Negrin D, Hernandez A, Nicolas-Perez D, Rodriguez E, Montesdeoca C, et al. Usefulness of a novel computer-aided detection system for colorectal neoplasia: a randomized controlled trial. *Gastrointestinal endoscopy* 2023; **97**: 528-36.e1.

34. Lau LHS, Ho JCL, Lai JCT, Ho AHY, Wu CWK, Lo VWH, et al. Effect of Real-Time Computer-Aided Polyp Detection System (ENDO-AID) on Adenoma Detection in Endoscopists-in-Training: A Randomized Trial. *Clinical gastroenterology and hepatology : the official clinical practice journal of the American Gastroenterological Association* 2024; **22**: 630-41.e4.

35. Spada C, Salvi D, Ferrari C, Hassan C, Barbaro F, Belluardo N, et al. A comprehensive RCT in screening, surveillance, and diagnostic AI-assisted colonoscopies (ACCENDO-Colo study). *Digestive and Liver Disease* 2025; **57**: 762-9.

36. Vilkoite I, Tolmanis I, Meri HA, Polaka I, Mezmale L, Anarkulova L, et al. The Role of an Artificial Intelligence Method of Improving the Diagnosis of Neoplasms by Colonoscopy. *Diagnostics (Basel, Switzerland)* 2023; **13**: 701.

37. Gong D, Wu L, Zhang J, Mu G, Shen L, Liu J, et al. Detection of colorectal adenomas with a real-time computer-aided system (ENDOANGEL): a randomised controlled study. *The lancet Gastroenterology & hepatology* 2020; **5**: 352-61.

38. Yao L, Zhang L, Liu J, Zhou W, He C, Zhang J, et al. Effect of an artificial intelligence-based quality improvement system on efficacy of a computer-aided detection system in colonoscopy: a four-group parallel study. *Endoscopy* 2022; **54**: 757-68.

39. Yao L, Li X, Wu Z, Wang J, Luo C, Chen B, et al. Effect of artificial intelligence on novice-performed colonoscopy: a multicenter randomized controlled tandem study. *Gastrointestinal endoscopy* 2024; **99**: 91-9.e9.

40. Zhang H, Wu Q, Sun J, Wang J, Zhou L, Cai W, et al. A computer-aided system improves the performance of endoscopists in detecting colorectal polyps: a multi-center, randomized controlled trial. *Frontiers in medicine* 2023; **10**: 1341259.

41. Glissen Brown JR, Mansour NM, Wang P, Chuchuca MA, Minchenberg SB, Chandnani M, et al. Deep Learning Computer-aided Polyp Detection Reduces Adenoma Miss Rate: A United States Multi-center Randomized Tandem Colonoscopy Study (CADeT-CS Trial). *Clinical gastroenterology and hepatology : the official clinical practice journal of the American Gastroenterological Association* 2022; **20**: 1499-507.e4.

42. Liu P, Wang P, Glissen Brown JR, Berzin TM, Zhou G, Liu W, et al. The single-monitor trial: an embedded CADe system increased adenoma detection during colonoscopy: a prospective randomized study. *Therapeutic advances in gastroenterology* 2020; **13**: 1756284820979165.

43. Wang P, Berzin TM, Glissen Brown JR, Bharadwaj S, Becq A, Xiao X, et al. Real-time automatic detection system increases colonoscopic polyp and adenoma detection rates: a prospective randomised controlled study. *Gut* 2019; **68**: 1813-9.

44. Wang P, Liu X, Berzin TM, Glissen Brown JR, Liu P, Zhou C, et al. Effect of a deep-learning computer-aided detection system on adenoma detection during colonoscopy (CADe-DB trial): a double-blind randomised study. *The lancet Gastroenterology & hepatology* 2020; **5**: 343-51.

45. Wang P, Liu P, Glissen Brown JR, Berzin TM, Zhou G, Lei S, et al. Lower Adenoma Miss Rate of Computer-Aided Detection-Assisted Colonoscopy vs Routine White-Light Colonoscopy in a Prospective Tandem Study. *Gastroenterology* 2020; **159**: 1252-61.e5.

46. Wang P, Liu X-G, Kang M, Peng X, Shu M-L, Zhou G-Y, et al. Artificial intelligence empowers the second-observer strategy for colonoscopy: a randomized clinical trial. *Gastroenterology report* 2023; **11**: goac081.

47. Ahmad A, Wilson A, Haycock A, Humphries A, Monahan K, Suzuki N, et al. Evaluation of a real-time computer-aided polyp detection system during screening colonoscopy: AI-DETECT study. *Endoscopy* 2023; **55**: 313-9.

48. Engelke C, Graf M, Maass C, Tews HC, Kraus M, Ewers T, et al. Prospective study of computer-aided detection of colorectal adenomas in hospitalized patients. *Scandinavian journal of gastroenterology* 2023; **58**: 1194-9.

49. Karsenti D, Tharsis G, Perrot B, Cattan P, Percie du Sert A, Venezia F, et al. Effect of real-time computer-aided detection of colorectal adenoma in routine colonoscopy (COLO-GENIUS): a single-centre randomised controlled trial. *The lancet Gastroenterology & hepatology* 2023; **8**: 726-34.

50. Lagstrom RMB, Brauner KB, Bielik J, Rosen AW, Crone JG, Gogenur I, et al. Improvement in adenoma detection rate by artificial intelligence-assisted colonoscopy: Multicenter quasi-randomized controlled trial. *Endoscopy International Open* 2025; **13**: a25215169.

51. Levartovsky A, Levy I, Bruckmayer L, Klang E, Ben-Horin S, Kopylov U. Real-world artificial intelligence-aided colonoscopy does not improve adenoma detection rates in patients with Inflammatory Bowel Disease. *Journal of Crohn's and Colitis* 2023; **17**: i415-i6.

52. Mangas-Sanjuan C, de-Castro L, Cubiella J, Diez-Redondo P, Suarez A, Pellise M, et al. Role of Artificial Intelligence in Colonoscopy Detection of Advanced Neoplasias. *Annals of Internal Medicine* 2023; **176**: 1145-52.

53. Ortiz O, Daca-Alvarez M, Rivero-Sanchez L, Gimeno-Garcia AZ, Carrillo-Palau M, Alvarez V, et al. An artificial intelligence-assisted system versus white light endoscopy alone for adenoma detection in individuals with Lynch syndrome (TIMELY): an international, multicentre, randomised controlled trial. *The lancet Gastroenterology & hepatology* 2024; **9**: 802-10.

54. Repici A, Badalamenti M, Maselli R, Correale L, Radaelli F, Rondonotti E, et al. Efficacy of Real-Time Computer-Aided Detection of Colorectal Neoplasia in a Randomized Trial. *Gastroenterology* 2020; **159**: 512-20.e7.

55. Repici A, Spadaccini M, Antonelli G, Correale L, Maselli R, Galtieri PA, et al. Artificial intelligence and colonoscopy experience: lessons from two randomised trials. *Gut* 2022; **71**: 757-65.

56. Seager A, Sharp L, Neilson LJ, Brand A, Hampton JS, Lee TJW, et al. Polyp detection with colonoscopy assisted by the GI Genius artificial intelligence endoscopy module compared with standard colonoscopy in routine colonoscopy practice (COLO-DETECT): a multicentre, open-label, parallel-arm, pragmatic randomised controlled trial. *The lancet Gastroenterology & hepatology* 2024; **9**: 911-23.

57. Thiruvengadam NR, Solaimani P, Shrestha M, Buller S, Carson R, Reyes-Garcia B, et al. The Efficacy of Real-time Computer-aided Detection of Colonic Neoplasia in Community Practice: A Pragmatic Randomized Controlled Trial. *Clinical gastroenterology and hepatology : the official clinical practice journal of the American Gastroenterological Association* 2024; **22**: 2221-30.e15.

58. Wallace MB, Sharma P, Bhandari P, East J, Antonelli G, Lorenzetti R, et al. Impact of Artificial Intelligence on Miss Rate of Colorectal Neoplasia. *Gastroenterology* 2022; **163**: 295-304.e5.

59. Bernhofer S, Prosenz J, Duller C, Venturi D, Maieron A. The Augmented Colonoscopy with Computer-Aided polyp Characterization (AC-CADx) study - prospective study comparing

the diagnostic reliability of optical diagnosis of trainees with experts without AI. *American Journal of Gastroenterology* 2025: 10.14309/ajg.0000000000003558.

60. Rondonotti E, Bergna IMB, Paggi S, Amato A, Andrealli A, Scardino G, et al. White light computer-aided optical diagnosis of diminutive colorectal polyps in routine clinical practice. *Endoscopy international open* 2024; **12**: E676-E83.

61. Hassan C, Balsamo G, Lorenzetti R, Zullo A, Antonelli G. Artificial Intelligence Allows Leaving-In-Situ Colorectal Polyps. *Clinical gastroenterology and hepatology : the official clinical practice journal of the American Gastroenterological Association* 2022; **20**: 2505-13.e4.

62. Maas MHJ, Neumann H, Shirin H, Katz LH, Benson AA, Kahloon A, et al. A computer-aided polyp detection system in screening and surveillance colonoscopy: an international, multicentre, randomised, tandem trial. *The Lancet Digital health* 2024; **6**: e157-e65.
