# Supplementary material for: Computer-aided polyp detection and characterisation systems to support colonoscopy: a systematic review with results stratified by each individual artificial intelligence system
Source: BMJ Digit Health Ai. 2026 Jun 22;2(1):e000046. doi: 10.1136/bmjdh-2026-000046 (PMC13528337; doi:10.1136/bmjdh-2026-000046)
Supplement: Supplementary file 3 [file bmjdh-2-1-bmjdh-2026-000046-s003.pdf]

Online Supplementary File 3

1 Data extraction table summaries

1.1 Summary of study characteristics

1.1.1 Computer-aided polyp detection (CADe)

Supplementary Table 27. Summary of study characteristics – included clinical studies - CADe

Study (study design; country)	Inclusion and exclusion criteria	Colonoscopy procedure, AI use and histology	Analyses[a]
**Argus® (Endosoft)**			
Strapko 2023[1] (parallel RCT – abstract only; USA; N=686)	Inclusion: ≥40 years; screening, surveillance or diagnostic colonoscopy; informed consent by patient or legal representative<br><br>Exclusion: <40 years; therapeutic colonoscopy; Lynch syndrome or FAP; contraindication for colonoscopy or biopsies of the colon; uncontrolled coagulopathy; confirmed diagnosis of IBD prior to colonoscopy; short bowel or ileostomy; pregnancy	Procedure: not reported<br><br>AI use: CADe, adjunct (no further details. Unclear when activated<br><br>Histology: not reported	Stratification: unclear<br><br>Analysis population: mITT[b]<br><br>Subgroups: none
**CAD EYE® (Fujifilm Healthcare UK Ltd.)**			

Alali 2025[2] (parallel RCT; Kuwait; N=102)	Inclusion: ≥45 years; average-risk screening or surveillance colonoscopy; provided informed consent; adequate bowel preparation (BBPS ≥2 per segment and overall score ≥6)<br><br>Exclusion: history of IBD; alarm symptoms (e.g. weight loss or rectal bleeding); known or suspected familial polyposis syndrome (e.g. FAP or Lynch syndrome); history of colon resection; poor bowel preparation; caecum not reached on colonoscopy	Procedure: HD white-light colonoscopy – no further details.<br><br>AI use: CADe (adjunct) and CADx (unclear, possibly autonomous). Activated during withdrawal.<br><br>Histology: All polyps removed using varying techniques and sent for histopathological examination by expert pathologists blinded to intervention. Vienna and WHO classifications used.	Stratification: unclear<br><br>Analysis population: ITT<br><br>Subgroups: none
Aniwan 2023[3] (parallel RCT; Thailand; N=1,245)	Inclusion: 50 to 75 years; asymptomatic; routine screening colonoscopy or screened after a positive FIT<br><br>Exclusion: IBD, familial polyposis syndrome, history of CRC or symptoms of CRC; post-polypectomy surveillance; prior colonic resection; prior pelvic radiation	Procedure: White-light; HD colonoscope; ENDOCUFF VISION™ used two of four arms; withdrawal time set to 6 to 10 min excluding irrigation and polypectomy<br><br>AI use: CADe, adjunct. Activated prior to insertion.<br><br>Histology: All detected polyps other than rectosigmoid polyps that appeared hyperplastic were resected and sent to expert pathologist	Stratification: unclear<br><br>Analysis population: ITT<br><br>Subgroups: none
Desai 2024[4] (parallel RCT; USA; N=1,031)	Inclusion: ≥45 years; screening or surveillance colonoscopy for history of polyps (surveillance	Procedure: HD colonoscope; Endoscopists adhered to usual withdrawing technique with minimum 6 min withdrawing and examining	Stratification: none reported<br><br>Analysis population: mITT[c]

	interval of 3 years or greater); able to provide informed consent<br><br>Exclusion: History of colon resection; IBD; FAP; severe comorbidity, including end-stage CV/pulmonary/liver/renal disease; pregnancy; unable to provide or refused to provide informed consent	colonic mucosa; use of advanced imaging or VCE not permitted<br><br>AI use: CADe, adjunct. Unclear when activated. EW10-EC02 CADe software-only medical device<br><br>Histology: Each polyp detected was removed or biopsied according to endoscopist judgement and sent to histology	Subgroups: screening and surveillance populations
Djinbachian 2024[5] (parallel RCT; Canada; N=467)	Inclusion: 45 to 80 years; elective colonoscopy for screening, surveillance or diagnostic purposes<br><br>Exclusion: known IBD; active colitis; coagulopathy; FAP; poor general health with ASA >3; emergency colonoscopy	Procedure: ELUXEO 7000 system with EC-706S-A/M and EC-760S-A/L colonoscopes.<br><br>AI use: CADe, adjunct. Activated during withdrawal. EW01-EC02 software. AI group also had water exchange and caecal retroflexion, whereas standard colonoscopy group did not.<br><br>Histology: all detected polyps resected and histologically assessed.	Stratification: unclear<br><br>Analysis population: ITT<br><br>Subgroups: endoscopist ADR >25% vs ≤25%
Hiratsuka 2025[6] (tandem RCT; Japan; N=94)	Inclusion: ≥20 years; scheduled for lower GI endoscopy<br><br>Exclusion: prior colectomy; IBD, FAP or other polyposis; experiencing difficulty with deep insertion; deemed difficult to safely examine (e.g. advanced	Procedure: Fujifilm ELUXEO 7000 endoscope system used with EC-760ZP colonoscope. Similarly skilled endoscopists performed first and second procedures in each patient but was a different endoscopist for the two procedures.	Stratification: history of colorectal polyps (yes/no); age <70 vs ≥70 years; male vs female<br><br>Analysis population: mITT[d]

	dementia); some eventually excluded due to poor bowel preparation	AI use: CADe, adjunct. Unclear when activated. EX-1 expansion unit with EW10-EC02 software.<br><br>Histology: all polyps other than whitish polyps and small polyps of sigmoid colon or rectum that were considered to be endoscopically hyperplastic were biopsied.	Subgroups: endoscopists with ≥10 vs <10 years' experience
Huneburg 2023[7] (parallel RCT; Germany; N=96)	Inclusion: ≥18 years; pathogenic germline variant in MLH1, MSH2 or MSH6 (Lynch Syndrome); written informed consent; ability to follow study instructions and likely to attend and complete all required visits; interval to last colonoscopy 10-36 months (had to have at least one prior colonoscopy)<br><br>Exclusion: Inability to understand the study; physical or psychiatric condition/ a systemic disease which may compromise safety of the subject, confound the trial results, interfere with the subject's participation in the study or prevent sufficient compliance; simultaneous participation in another clinical trial or participation in trial of investigational medicinal product within 30 days prior to trial start; screening laboratory test results within the following parameters (quick >50%, thrombocytes >50.000 G/l); current or planned pregnancy or nursing women; previous extensive colorectal surgery	Procedure: HD white-light endoscopy; endoscopists used LCI, BLI and AI characterisation mode (for AI group) to assess each lesion; lesions resected endoscopically using standard polypectomy techniques.<br><br>AI use: CADe, adjunct. Activated during withdrawal<br><br>Histology: Histology of all lesions assessed using Vienna criteria by experienced blinded GI pathologist	Stratification: previous colorectal surgery (yes/no) and affected MMR gene (MLH1/MSH2/MSH6)<br><br>Analysis population: mITT[e]<br><br>Subgroups: history vs no history of CRC

	(proctocolectomy or colectomy with ileorectal anastomosis); index colonoscopy; insufficient bowel preparation (BBPS score <2 by colonic segment)		
Miyaguchi 2024[8] (parallel RCT; Japan; N=800)	Inclusion: ≥20 years; colonoscopy for positive FIT, follow-up of colon polyps or abdominal symptoms; written informed consent<br><br>Exclusion: colonoscopy without bowel preparation; intestinal obstruction, stenosis or fistula; history of colorectal surgery; active IBD; diverticulitis; active or suspected colorectal bleeding; ileus, alcoholism, hyperthyroidosis, severe heart failure, liver disease or renal dysfunction, and pregnancy also mentioned in trial record	Procedure: LCI colonoscopy using attached transparent hoods; white-light imaging during anal insertion and LCI during withdrawal<br><br>AI use: CADe, adjunct. Activated during withdrawal. CAD EYE® EW10-EC02<br><br>Histology: Biopsy or resection of observed polyps performed and sent for histopathological analysis	Stratification: unclear<br><br>Analysis population: ITT<br><br>Subgroups: FIT vs other indication, trainee vs expert endoscopists
Nakashima 2023[9] (tandem RCT; Japan; N=415)	Inclusion: 21 to 81 years; undergoing endoscopy as a primary endoscopic screening for CRC, patients who tested positive for the FIT for occult blood or patients with colorectal neoplasia undergoing endoscopic resection (surveillance post-resection of polyps in trial record); willingness to participate in the RCT; written informed consent<br><br>Exclusion: prior colorectal surgery; IBD	Procedure: white-light imaging without magnification, chromoendoscopy, or image-enhanced endoscopy; adenomas >6 mm indicated for endoscopic resection, diminutive polypoid adenomas ≤5 mm could be followed up rather than removed; tandem procedures performed by same examiner and process of insertion and withdrawal repeated<br><br>AI use: CADe, adjunct. Unclear when activated.	Stratification: unclear<br><br>Analysis population: possibly ITT - unclear<br><br>Subgroups: none

		Histology: performed by single pathologist specialising in GI tract blinded to endoscopy results	
Rondonotti 2022[10] (parallel RCT; Italy; N=800)	Inclusion: 50 to 74 years; within CRC screening programme with a positive FIT screening test<br><br>Exclusion: not eligible for screening program (i.e. colonoscopy within previous 5 years, personal history of CRC, colonic adenomas, IBD, severe comorbidity); prior colonic resection; antithrombotic therapy precluding polyp resection and pathology assessment; inadequate bowel preparation (defined as BBPS <2 in at least one colonic segment); caecal intubation not achieved; refusal to give informed written consent	Procedure: HD white-light colonoscopy; withdrawal time of at least 6 min (2 min in right, transverse and left colon) was mandatory; all identified polyps removed or biopsied other than diminutive (1 to 5 mm) hyperplastic polyps in the rectum and judged as not clinically significant by the endoscopist<br><br>AI use: CADe, adjunct. Activated prior to insertion. Characterisation mode not used.<br><br>Histology: Vienna and WHO classifications described; histology assessed by expert pathologists qualified for FIT-based CRC screening programme	Stratification: endoscopy centre<br><br>Analysis population: mITT[f]<br><br>Subgroups: endoscopist baseline ADR ≤40%, 41 to 45% and ≥46%
Scholer 2024[11] (parallel RCT; Sweden; N=193)	Inclusion: 40 to 90 years; cancer screening, alarm symptoms (i.e., iron-deficiency anaemia, suspicion of malignancy following rectal examination and CT findings that raise suspicion of malignancy), inconclusive CT findings (suggestive of a benign but inconclusive cause) and other (positive faecal occult blood stool test, polyp surveillance, hereditary CRC, diarrhoea)	Procedure: white-light imaging or LCI depending on preference of examiner; standard of paediatric HD colonoscopes<br><br>AI use: CADe, adjunct. Activated during withdrawal.	Stratification: endoscopist experience<br><br>Analysis population: per protocol[g]<br><br>Subgroups: none for CAD EYE® data specifically

	Exclusion: history of IBD; contraindication for polypectomy or known polyps; incomplete examinations due to factors such as obstructive cancer, technical issues or inadequate bowel preparation; BBPS <2 in one segment or a total BBPS <6	Histology: assessed by pathologists blinded to intervention.	
Tiankanon 2024[12] (parallel RCT; Thailand; N=800)	Inclusion: 50 to 75 years; routine screening colonoscopy or screened after positive FIT; asymptomatic<br><br>Exclusion: known CRC history; IBD; familial polyposis syndrome; prior colonic resection; also prior pelvic radiation listed in trial record	Procedure: white-light using HD colonoscopes without magnification; polypectomy during insertion and withdrawal<br><br>AI use: CADe, adjunct. Activated prior to insertion. EW10-EC02 software and compatible expansion unit EX-1<br><br>Histology: all resected polyps sent to GI pathologists blinded to intervention; WHO criteria used	Stratification: none reported<br><br>Analysis population: ITT<br><br>Subgroups: none
Yamaguchi 2024[13] (parallel RCT; Japan; N=231)	Inclusion: ≥20 years; scheduled for outpatient colonoscopy due to positive FIT, surveillance after colonic polypectomy (trial record also suggests digestive symptoms, GI cancer screening indication, family history of CRC or colorectal adenoma, or others needing lower GI endoscopy by physician in charge would initially have been eligible); understand and agree to consent document	Procedure: Performed by trainee in back-to-back method with an expert; expert could support with advancing endoscope to caecum as needed; each section of colon observed by trainee followed by expert in turn; trainee first observed and measured the polyps in absence of expert followed by expert assessment; expert confirmed polyps and adenomas and performed additional observation, endoscopic	Stratification: unclear<br><br>Analysis population: mITT[h]<br><br>Subgroups: none

	Exclusion: ileus; suspected bowel obstruction; toxic megacolon; prior abdominal or pelvic surgery; IBD; advanced malignancy; severe liver damage (Child-Pugh grade C); dementia or other cognitive disorders; hypersensitivity to bowel preparation drugs for colonoscopy; pregnancy or lactation	polypectomy or endoscopic mucosal resection as needed.<br><br>AI use: CADe, adjunct. Activated during withdrawal. Characterisation function used to diagnose lesions if necessary when measuring polyps<br><br>Histology: not reported	
Zimmermann-Fraedrich 2025[14] (parallel RCT; Germany; N=1,627)	Inclusion: ≥50 years; screening colonoscopy (current age cut-offs 50 years for men and 55 years for women) or diagnostic colonoscopy (including polyp follow-up and symptom evaluation)<br><br>Exclusion: not reported	Procedure: colonoscopes from 700 series used – no further details<br><br>AI use: CADe, adjunct. Activated prior to insertion.<br><br>Histology: all detected polyps removed and sent for histological analysis by histopathologists specialised in GI pathology.	Stratification: unclear<br><br>Analysis population: ITT<br><br>Subgroups: none
**Discovery™ (Pentax Medical UK)**			
Maas 2024 - Discovery™[15] (parallel RCT; Canada, France, Germany, Italy, Netherlands, Russia; N=497)	Inclusion: ≥18 years; scheduled for non-iFOBT screening, surveillance, or diagnostic (excluding iFOBT-positive referrals) colonoscopy<br><br>Exclusion: known colorectal tumours or polyps upon referral; referral for therapeutic procedures; inadequately corrected coagulation disorder or	Procedure: HD PENTAX colonoscopes used; aim minimum withdrawal time 6 min (excluding polypectomies or other interventions) with upper withdrawal time of 10 min; all lesions collected other than diminutive (1 to 5 mm)	Stratification: index colonoscopy vs not<br><br>Analysis population: mITT[l]<br><br>Subgroups: screening, surveillance and diagnostic indications; baseline

	continued use of anticoagulation medication; ASA score of ≥3; known or suspected IBD	polyps located in rectum and considered hyperplastic by endoscopist<br><br>AI use: CADe, adjunct. Activated before insertion, mandatory use during withdrawal. Software versions 1.0.3.1 and 1.0.4 were used.<br><br>Histology: Vienna classification used by experienced pathologists for histological assessment of all resected polyps; pathologists blinded to intervention and endoscopic diagnosis	endoscopist ADR (low, medium and high detector tertiles)
**ENDO-AID™ (Olympus Medical Systems Corp.)**			
Gimeno-Garcia 2023[16] (parallel RCT; Tenerife; N=370)	Inclusion: ≥18 years; colonoscopy scheduled for morning shift (requirements for colonoscopy indication unclear or did not apply)<br><br>Exclusion: colon resection; treatment with anticoagulants or antiplatelet agents that may preclude polyp resection; a recent good-quality colonoscopy (<6 months) (i.e., scheduled for endoscopic therapy); IBD; incomplete colonoscopy; inadequate preparation assessed by the BBPS; diagnosis or high suspicion for hereditary CRC; patients with polyposis syndromes	Procedure: minimum 6 min withdrawal time (excluding biopsy and resections); white-light imaging; use of add-on devices not permitted; techniques such as NBI or dye-based chromoendoscopy only used for characterisation of detected lesions at discretion of endoscopists; all polyps removed other than tiny hyperplastic polyps in rectum<br><br>AI use: CADe, adjunct. Activated during withdrawal. Target mode at most sensitive setting (type A) used	Stratification: unclear<br><br>Analysis population: ITT<br><br>Subgroups: endoscopist experience (high vs low detectors - ≥40% vs <40% baseline ADR)

		Histology: two pathologists specialising in colon pathology blinded to assignment group performed histological assessment; Vienna classification used for histology	
Lau 2024[17] (parallel RCT; Hong Kong; N=766)	Inclusion: ≥18 years; undergoing elective colonoscopies for screening, surveillance, or diagnostic purposes<br><br>Exclusion: incomplete colonoscopies or inadequate bowel preparation; contraindications to colonoscopy or polypectomy; known colorectal lesions for staged procedures; previous colonic resection; personal history of CRC/polyposis syndrome/IBD, advanced comorbid conditions (ASA grade ≥4); pregnancy; unable to obtain informed consent	Procedure: white-light HD endoscopy; NBI or other enhanced imaging only permitted for characterisation; no magnification or chromoendoscopy permitted; use of distal attachment devices not permitted; performed by trainees with supervisors present (minimal interference in junior endoscopists' decisions) – could alert when polyp missed and support with caecal intubation, could also advise during resection or take over the resection; all polyps removed with exception of diminutive non-neoplastic hyperplastic polyps as judged by operators<br><br>AI use: CADe, adjunct. Activated during withdrawal. Target mode of technology used in this study.<br><br>Histology: Vienna classification used for histological assessment by independent pathologists blinded to intervention	Stratification: age (<65 years vs ≥65 years), sex and endoscopist experience (beginner vs intermediate level)<br><br>Analysis population: mITT[m]<br><br>Subgroups: symptomatic, screening and surveillance indications; beginner vs intermediate level endoscopists

Lui 2024[18] (parallel RCT; Hong Kong; N=682)	Inclusion: ≥40 years; scheduled for elective colonoscopy for screening, surveillance, or diagnostic workup.<br><br>Exclusion: pregnant women; inability to provide written informed consent; prior colorectal resection; personal history of CRC; IBD; FAP, Peutz-Jeghers syndrome or other polyposis syndromes; deemed unsuitable or high risk for polypectomy (with bleeding tendencies or severe comorbid illnesses)	Procedure: white-light HD colonoscopy; ENDOCUFF VISION™ used within one randomised group; all detected polyps removed during withdrawal only; minimum 6 min withdrawal time<br><br>AI use: CADe, adjunct. Activated during withdrawal.<br><br>Histology: WHO classification used for histological assessment by experienced pathologists blinded to intervention	Stratification: Colonoscopy indication and endoscopist experience<br><br>Analysis population: ITT<br><br>Subgroups: screening population separate
Spada 2025[19] (parallel RCT; Italy; N=1,228)	Inclusion: 40 to 85 years; screening (opportunistic or immunological FOBT based) or surveillance colonoscopy<br><br>Exclusion: IBD; history of surgical resection of any part of the colon; known polyps on referral; polyposis syndrome; referral for therapeutic procedures; inadequately managed anticoagulation disorders or use of anticoagulation medications; unable to provide informed consent	Procedure: HD procedures. Vital staining and VCE not permitted for polyp detection. Withdrawal time minimum of 6 min from caecum excluding interventions.<br><br>AI use: CADe, adjunct. Activated during withdrawal. Of two modes available, mode B used (suppress more false positives compared to A)<br><br>Histology: Resected polyps sent for histopathological examination according to Vienna criteria by experienced pathologists.	Stratification: male vs female; age categories (40 to 50, 51 to 50, 61 to 70, 71 to 80 and 81 to 85 years)<br><br>Analysis population: ITT for some outcomes, mITT only for others[n]<br><br>Subgroups: none

Vilkoite 2023[20] (parallel RCT; Latvia; N=400)	Inclusion: ≥18 years; any patient sent for colonoscopy examination by the family doctor; signed informed consent form<br><br>Exclusion: previously undergone colonoscopy examination; IBD; hereditary polyposis syndrome; known CRC; previously undergone colorectal surgery; contraindications for polypectomy; bad bowel preparation on a BBPS of 0 to 1 in any of the three bowel segments; standard contraindications to colonoscopy such as acute diverticulitis and known or suspected perforation	Procedure: minimum withdrawal time 7 min; methylene blue staining used to detect flat polyps of right colon; at least two pieces from polyps taken before polypectomy<br><br>AI use: CADe, adjunct. Activated during withdrawal.<br><br>Histology: all removed polyps and specimens sent for histological assessment by expert pathologists according to WHO criteria	Stratification: unclear<br><br>Analysis population: mITT[o]<br><br>Subgroups: none
**ENDOANGEL® Lower Gastrointestinal Endoscope Image Auxiliary Diagnostic Equipment (Wuhan ENDOANGEL Medical Technology Co. Ltd.)**			
Gong 2020[21] (parallel RCT; China; N=704)	Inclusion: 18 to 75 years; consecutive patients undergoing colonoscopy - trial record expands on this (colonoscopy for screening, clinical symptoms or surveillance); able to provide informed consent and willing to comply with all study process<br><br>Exclusion: contraindications to colonoscopy indication; history of IBD; CRC or colorectal surgery; previous unsuccessful colonoscopy; contraindication for biopsy; suspected or known bowel obstruction of perforation; or currently pregnant or lactating; trial record also includes known polyposis syndrome and other high-risk diseases or special circumstances	Procedure: advanced optical imaging required following detection to assess morphology with biopsies or removal of suspected adenomas performed – those not removed of biopsied considered non-adenomatous<br><br>AI use: CADe, adjunct. Activated prior to insertion.<br><br>Histology: adenomas diagnosed based on pathological results of those resected or biopsied (only suspected adenomas were removed or biopsied)	Stratification: unclear<br><br>Analysis population: ITT<br><br>Subgroups: none

	that researcher believes makes them unsuitable for participation		
Yao 2022[22] (parallel RCT; China; N=539)	Inclusion: ≥18 years; attended endoscopy centre between 1 July and 15 October 2020 (colonoscopy indication requirements unclear or did not apply); ability to sign and understand informed consent documents<br><br>Exclusion: known contraindications to biopsy; bowel obstruction or perforation; pregnant or lactating; suffering from polyposis syndromes; history of IBD, CRC, or colorectal surgery; caecum was not reached; suspicion for polyposis syndromes, IBD, intestinal tuberculosis or CRC; others also listed in trial record - other clinical trial participation, drug or alcohol abuse or mental disorder in last 5 years	Procedure: HD colonoscopes; minimum 6 min withdrawal time; all polyps removed or biopsied with exception of diminutive hyperplastic-appearing polyps located in the rectum according to endoscopist judgement; unbiopsied rectal polyps arranged for resection one month after examination and pathology followed up<br><br>AI use: CADe, adjunct. Activated during withdrawal.<br><br>Histology: not reported	Stratification: unclear<br><br>Analysis population: mITT[p]<br><br>Subgroups: none
Yao 2024[23] (tandem RCT; China; N=456)	Inclusion: >18 years; colonoscopy for diagnostic, screening or surveillance; able to read, understand and sign informed consent form<br><br>Exclusion: known contraindication for biopsy; bowel obstruction or perforation; currently pregnant or lactating; experiencing polyposis syndromes; history of IBD, CRC or colorectal surgery; caecum not reached during procedure; highly suspected of	Procedure: white-light endoscopy with NBI at discretion of endoscopist; tandem colonoscopies on same day; all detected polyps biopsied or removed with exception of diminutive hyperplastic-appearing polyps located in rectum and according to judgement of standby expert endoscopists; insertion performed by experts in all three groups with withdrawal phase performed by novices in two	Stratification: unclear<br><br>Analysis population: mITT[q]<br><br>Subgroups: none

	having polyposis syndromes, IBD, intestinal tuberculosis or CRC; insertion time >15 minutes in the first pass; drug or alcohol abuse or mental illness within the last 5 years; known intestinal stenosis or space-occupying tumour; trial record also mentions history of allergy to pre-used spasmolysis, unable to perform biopsy and polyp removal due to coagulation disorders or oral anticoagulation, and high-risk diseases or other special conditions that the investigator considers to be unsuitable for inclusion	of three groups; second-pass endoscopists not blinded to results of first procedure; minimum 6 min withdrawal time<br><br>AI use: CADe, adjunct. Activated during withdrawal.<br><br>Histology: pathological assessment reported, no further details	
Zhang 2023[24] (parallel RCT; China; N=1,293)	Inclusion: 18 to 75 years; colonoscopy for diagnosis or screening; able to provide written informed consent; full legal capacity<br><br>Exclusion: contraindications to colonoscopy (history of acute myocardial infarction within 6 months, severe hypohepatia, renal failure, and mental disorders); use of anticoagulants (aspirin, warfarin, etc.); known polyposis syndromes, familial polyposis or IBD; known or highly suspected CRC; prior colorectal surgery; pregnancy; currently participating in other clinical trials	Procedure: when detected, routine diagnostic and treatment processes at each hospital followed to decide whether to perform a polypectomy<br><br>AI use: CADe, adjunct. Activated during withdrawal.<br><br>Histology: no histology – videos of procedure reviewed by an independent evaluation group (two experts with experience >5 ears and total volume >3000 colonoscopies) which labelled if examination was positive (polyp detected) or not. Third endoscopist involved if disagreement.	Stratification: unclear<br><br>Analysis population: FAS/mITT[r]<br><br>Subgroups: none

**EndoScreener® (WISION AI)**			
Glissen Brown 2022[25] (tandem RCT; USA; N=223)	Inclusion: ≥22 years; presenting for colonoscopy for CRC screening or surveillance<br><br>Exclusion: diagnostic colonoscopy (for indications such as GI haemorrhage); IBD; colorectal masses >2 cm in size; referred for endoscopic mucosal resection; standard contraindications to colonoscopy such as acute diverticulitis and known or suspected perforation; diminished cognitive capacity also mentioned in trial record.	Procedure: HD white-light colonoscopy; all polyps removed at detection using standard polypectomy techniques or biopsied other than diminutive hyperplastic polyps of rectum at discretion of endoscopist if deemed clinically insignificant<br><br>AI use: CADe, adjunct. Activated during withdrawal.<br><br>Histology: assessed by clinical pathologists blinded to intervention	Stratification: unclear<br><br>Analysis population: mITT[s]<br><br>Subgroups: none
Liu 2020[26] (parallel RCT; China; N=790)	Inclusion: 14 to 90 years; underwent colonoscopy for any indication between September 2018 and February 2019<br><br>Exclusion: history of IBD and highly suspected cases during colonoscopy examination; history of adenoma polyposis and highly suspected cases during colonoscopy examination; history of CRC and highly suspected cases during colonoscopy examination; history of colon surgery; contradiction of biopsy; failed procedure to insertion to cecum.	Procedure: high image-quality colonoscopes and HD monitors; routine colonoscopy procedure; all polyps identified by endoscopist taken as cold forceps biopsy for pathological examination<br><br>AI use: CADe, adjunct. Activated during withdrawal.<br><br>Histology: pathological assessment mentioned, no further details	Stratification: unclear<br><br>Analysis population: mITT[t]<br><br>Subgroups: none

Wang 2019[27] (parallel RCT; China; N=1,058)	Inclusion: age inclusion criterion unclear or did not apply; underwent a colonoscopy from September 2017 to February 2018<br><br>Exclusion: history of IBD; CRC; colorectal surgery; contraindication for biopsy; prior failed colonoscopy; high suspicion of polyposis syndromes, IBD or typical advanced CRC	Procedure: HD colonoscopes and monitors; detected polyps underwent cold forceps biopsy for histology<br><br>AI use: CADe, adjunct. Activated during withdrawal.<br><br>Histology: pathological assessment mentioned, no further details	Stratification: unclear<br><br>Analysis population: mITT[u]<br><br>Subgroups: none
Wang 2020 (effect of a deep…)[28] (parallel RCT; China; N=962)	Inclusion: 18 to 75 years; presenting for diagnostic (symptomatic) or screening colonoscopies<br><br>Exclusion: history of IBD, CRC or colorectal surgery; contraindication for biopsy (e.g., use of anticoagulants); previously unsuccessful colonoscopy (i.e. did not reach caecum); at high suspicion for polyposis syndromes, IBD or CRC	Procedure: white-light HD colonoscopy; sham system used for comparator arm; cold forceps biopsy used to obtain samples for pathology<br><br>AI use: CADe, adjunct. Activated during withdrawal.<br><br>Histology: pathological assessment mentioned, no further details	Stratification: unclear<br><br>Analysis population: mITT[u]<br><br>Subgroups: none
Wang 2020 (lower adenoma miss…)[29] (tandem RCT; China; N=369)	Inclusion: 18 to 75 years; referred for diagnostic, screening colonoscopy or surveillance colonoscopy (for patients who underwent previous polypectomy)<br><br>Exclusion: history of IBD, CRC, colorectal surgery or contraindication for biopsy; caecum was not reached; high suspicion for polyposis syndromes,	Procedure: same day tandem HD colonoscopies performed by same endoscopist in each patient; white-light only with NBI at discretion of endoscopists; all polyps biopsied or removed by cold forceps biopsy with larger ones referred for later resection, with exception of diminutive (≤2 mm) rectal polyps considered to be hyperplastic by endoscopist	Stratification: unclear<br><br>Analysis population: mITT[v]<br><br>Subgroups: none

	IBD or CRC; cases of difficult insertion, defined as insertion time >7 minutes in first pass	AI use: CADe, adjunct. Activated during withdrawal.<br><br>Histology: all biopsies sent for pathological examination, no further details	
Wang 2023[30] (parallel RCT; China; N=1,261)	Inclusion: 18 to 75 years; symptomatic, screening or surveillance colonoscopy<br><br>Exclusion: history of IBD, CRC, polyposis syndromes or colorectal surgery; contraindication for biopsy; current lower GI bleeding; poor general condition; did not consent before randomisation; during colonoscopy, those highly suspected of suffering from polyposis syndromes, IBD or CRC mass, or the caecum was not reached; after colonoscopy, patients who withdrew their consent or failed the pathology assessment due to insufficient tissue from cold-forceps biopsy.	Procedure: white-light HD colonoscopy with other imaging modalities at endoscopist discretion; all polyps verified by endoscopist biopsied or removed suing cold forceps biopsy other than diminutive (≤2 mm) rectal polyps deemed to be hyperplastic by endoscopist; standard colonoscopy group included trainees as observers for comparison against AI<br><br>AI use: CADe, adjunct. Activated during withdrawal. Version 1.01 used.<br><br>Histology: all biopsies sent for pathological examination, no further details	Stratification: unclear<br><br>Analysis population: mITT[w]<br><br>Subgroups: none
**GI Genius™ (Medtronic)**			
Ahmad 2023[31] (parallel RCT; UK; N=614)	Inclusion: 60 to 74 years; positive FIT attending for screening colonoscopy within the NHS BCSP or an established history of adenomas attending for surveillance colonoscopy within the BCSP, or patients aged 55 years referred for colonoscopy due	Procedure: routine HD colonoscopy with option of ENDOCUFF VISION™ or transparent plastic cap; typical-appearing small, hyperplastic, shiny rectosigmoid polyps left *in situ* and not included in assessment of polyps detected	Stratification: unclear<br><br>Analysis population: ITT<br><br>Subgroups: none

	to large or multiple adenomas being found during screening flexible sigmoidoscopy.<br><br>Exclusion: risk profile (family history or other reasons); follow-up was conducted outside the BCSP; did not give consent to the study	AI use: CADe, adjunct. Activated prior to insertion. First commercially available GI Genius™ system used (CB1708-EU)<br><br>Histology: post-procedure histology results reviewed within 2 weeks	
Engelke 2023[32] (parallel RCT; Sweden; N=232)	Inclusion: ≥18 years; colonoscopy for primary screening, post-polypectomy surveillance, tumour follow-up or work-up for GI symptoms such as bleeding and anaemia, IBD, diarrhoea and tumour search (diagnostic colonoscopy); obtained informed consent<br><br>Exclusion: pre-planned partial colonoscopy or planned repetition of colonoscopy due to an unprepared colon	Procedure: HD colonoscopy; polypectomies performed using forceps, cold snare or diathermic snares depending on size<br><br>AI use: CADe, adjunct. Unclear when activated.<br><br>Histology: all resected specimens underwent histopathological examination, no further details	Stratification: unclear<br><br>Analysis population: unclear but possibly ITT<br><br>Subgroups: diagnostic colonoscopy vs pre-planned colonoscopies (also broken down into more specific subgroups for indication); expert endoscopists (>200 colonoscopies annually and >50 polypectomies within last three years) vs inexperienced endoscopists
Karsenti 2023[33] (parallel RCT; France; N=2,015)	Inclusion: ≥18 years; undergoing total colonoscopy; ASA score 1 to 3;<br><br>Exclusion: referred for polyp resection; previous colonic surgery; colonic stenosis; recent acute diverticulitis (<6 weeks before colonoscopy); IBD; pregnancy; haemostasis disorders (e.g., partial thromboplastin time >42 seconds, prothrombin time <50% or platelet count <50,000 platelets per µL);	Procedure: limited procedural details reported<br><br>AI use: CADe, adjunct. Activated during withdrawal. Version 2.0.2 used.<br><br>Histology: histopathological data collected 1 to 2 weeks following colonoscopy; pathologists were blinded to intervention	Stratification: none<br><br>Analysis population: mITT[x]<br><br>Subgroups: endoscopist experience (low, medium and high baseline ADR)

	treated with clopidogrel, ticagrelor or prasugrel; participating in another clinical study; incomplete colonoscopy or inappropriate bowel preparation score		
Lagstrom 2025[34] (parallel RCT; Denmark; N=795)	Inclusion: ≥18 years; screening following positive FIT (>100 μg/L), surveillance or diagnostic colonoscopy<br><br>Exclusion: colonoscopy for removal of previously detected polyps; control colonoscopies due to IBD; emergency colonoscopies; bowel preparation so poor that prevented colonoscopy being performed; cancer suspected during the colonoscopy	Procedure: limited procedural details reported<br><br>AI use: CADe, adjunct. Activated during withdrawal.<br><br>Histology: not reported	Stratification: unclear<br><br>Analysis population: per protocol[y]<br><br>Subgroups: expert (>1000 colonoscopies) vs non-expert (≤1000 colonoscopies)
Levartovsky 2023[35] (retrospective study – abstract only; Israel; N=996)	Inclusion: colonoscopies 11 months prior to introduction of technology and 15 months after<br><br>Exclusion: colonoscopy for evaluation of IBD severity, for known or suspected malignancy or therapeutic endoscopy; incomplete colonoscopies; colonoscopies with inadequate preparation	Procedure: compared results from before (11 months prior) and after (15-month period) introduction of AI technology. No further details.<br><br>AI use: CADe, adjunct. Unclear when activated.<br><br>Histology: not reported	Stratification: NA - retrospective<br><br>Analysis population: NA - retrospective<br><br>Subgroups: none
Mangas-Sanjuan 2023[36] (parallel RCT; Spain; N=3,213)	Inclusion: ≥18 years; presenting for colonoscopy after a first positive FIT on CRC screening (haemoglobin 20 microg/g faeces)	Procedure: HD endoscopes; minimum 6 min withdrawal time; all polyps removed other than those considered non-resectable and diminutive hyperplastic-appearing polyps in	Stratification: centre, sex and age<br><br>Analysis population: mITT[z]

	Exclusion: personal history of CRC, IBD, colorectal surgery, terminal illness or severe disease; familial CRC or family history of inherited CRC syndrome; lack of written informed consent	rectum judged not to be clinically significant; targeted indigo carnine or VCE could be used for characterisation but not detection;<br><br>AI use: CADe, adjunct. Activated during withdrawal. Version 2.0.0 used.<br><br>Histology: pathologists specialising in GI oncology and blinded to intervention evaluated polyp histology using WHO classification	Subgroups: endoscopist baseline ADR <60% vs ≥60%
Ortiz 2024[37] (parallel RCT; Belgium, Germany, Italy, Spain; N=430)	Inclusion: ≥18 years; surveillance colonoscopy for Lynch syndrome (pathogenic or likely pathogenic, according to American College of Medical Genetics and Genomics guidelines, germline variant in MLH1, MSH2, MSH6, or EPCAM)<br><br>Exclusion: history of total colectomy; concomitant IBD; inability or refusal to sign the informed consent; colonoscopy within the past 12 months; inadequate bowel preparation; incomplete procedure; PMS2 mutation.	Procedure: HD white-light endoscopy; use of add-on devices permitted; minimum 6 min withdrawal time; VCE and other imaging techniques only for characterisation; removal of lesions using current guidelines for polypectomy techniques; all detected lesions resected and sent for histology with exception of diminutive (≤5 mm) rectosigmoid polyps with a high-confidence of being hyperplastic<br><br>AI use: CADe, adjunct. Activated during withdrawal.<br><br>Histology: pathology assessed by expert pathologists specialising in GI pathology using the Vienna classification, European guidelines for quality assurance in CRC screening and WHO criteria	Stratification: centre<br><br>Analysis population: ITT<br><br>Subgroups: low vs high detector endoscopists

Repici 2020[38] (parallel RCT; Italy; N=685)	Inclusion: 40 to 80 years; colonoscopy for primary CRC screening, post-polypectomy surveillance, or work up following positive FIT (threshold 20 µg/Hb/g faeces) or for symptoms/signs<br><br>Exclusion: personal history of CRC, IBD or a colonic resection; antithrombotic therapy precluding polyp resection; lack of informed written consent	Procedure: HD colonoscopy; minimum 6 min withdrawal time; all polyps removed or biopsied with exception of diminutive hyperplastic-appearing polyps located in rectum and judged not to be clinically significant by endoscopist; magnification, chromoendoscopy or light-modification technologies restricted to polyp characterisation at endoscopist discretion<br><br>AI use: CADe, adjunct. Activated prior to insertion.<br><br>Histology: expert pathologists participating in the organised screening programme and blinded to intervention assessed histology using Vienna classification	Stratification: gender, age, personal history of adenomas and site<br><br>Analysis population: mITT[aa]<br><br>Subgroups: FIT+, GI symptoms and screening/surveillance indications
Repici 2022[39] (parallel RCT; Italy, Switzerland; N=660)	Inclusion: 40 to 80 years; colonoscopy for colorectal neoplasia diagnosis, divided into primary screening colonoscopy (outside regional screening programme), work up following positive FIT test (threshold 20 µg Hb/g faeces) within national screening programme, post-polypectomy surveillance, and work up for symptoms/signs (diagnostic colonoscopy)	Procedure: HD colonoscopy; minimum 6 min withdrawal time<br><br>AI use: CADe, adjunct. Activated prior to insertion.<br><br>Histology: Vienna classification used for histology, no further details	Stratification: gender, age, personal history of adenomas and site<br><br>Analysis population: mITT[aa]<br><br>Subgroups: FIT+, post-polypectomy surveillance, primary CRC screening and GI symptom indications

	Exclusion: personal history of CRC, IBD or colonic resection; antithrombotic therapy precluding polyp resection; lack of informed written consent		
Scholer 2024[11] (parallel RCT; Sweden; N=47)	Inclusion: 40 to 90 years; cancer screening, alarm symptoms (i.e., iron-deficiency anaemia, suspicion of malignancy following rectal examination and CT findings that raise suspicion of malignancy), inconclusive CT findings (suggestive of a benign but inconclusive cause) and other (positive faecal occult blood stool test, polyp surveillance, hereditary CRC, diarrhoea)<br><br>Exclusion: history of IBD; contraindication for polypectomy or known polyps; incomplete examinations due to factors such as obstructive cancer, technical issues or inadequate bowel preparation; BBPS <2 in one segment or a total BBPS <6	Procedure: white-light imaging or LCI depending on preference of examiner; standard of paediatric HD colonoscopes<br><br>AI use: CADe, adjunct. Activated during withdrawal.<br><br>Histology: assessed by pathologists blinded to intervention.	Stratification: endoscopist experience<br><br>Analysis population: per protocol[g]<br><br>Subgroups: none for GI Genius™ data specifically
Seager 2024[40] (parallel RCT; UK; N=2,032)	Inclusion: ≥18 years; planned colonoscopy for GI symptoms, surveillance after previous colonic pathology (e.g. previous polyps, cancer or other colorectal pathology other than IBD) or due to family history of CRC (one first-degree relative with CRC diagnosed before age of 50 years, or two first-degree relatives with CRC diagnosed at any age), or for CRC screening	Procedure: ENDOCUFF VISION™ used in minority of patients; standard procedures followed<br><br>AI use: CADe, adjunct. Activated prior to insertion. Version 2.0 used.	Stratification: age category, sex, colonoscopy subpopulation and NHS Trust<br><br>Analysis population: ITT<br><br>Subgroups: screening vs symptomatic colonoscopy indications

	Exclusion: unable to provide written informed consent; absolute contraindications to colonoscopy; established or suspected large bowel obstruction or pseudo-obstruction; known CRC or polyposis syndromes, colonic strictures or active colitis; undergoing colonoscopy for assessment or surveillance of IBD; pregnant (confirmed or suspected); undergoing planned assessment or treatment of a known and current pathology or lesion (including polypectomy site checks); referred with polyps identified on a bowel scope procedure (a previous national screening programme comprised of one-off flexible sigmoidoscopy at 55 years); remained on therapeutic anticoagulation for the procedure (excluding low-dose aspirin 75 mg once daily, which could be contained)	Histology: histology assessments mentioned but no further details	
Thiruvengadam 2024[41] (parallel RCT; USA; N=1,100)	Inclusion: ≥30 years; colonoscopy for any indication<br><br>Exclusion: prior colectomy; lack of informed consent	Procedure: white-light HD colonoscopy; use of magnification, chromoendoscopy and light modification techniques at discretion of endoscopist; minimum withdrawal time 8 min; required to removal all polyps proximal to sigmoid colon/rectum, with removal in sigmoid colon/rectum left to endoscopist discretion (diminutive polyps deemed to be hyperplastic could be left *in situ*); biopsies were taken if resections not feasible	Stratification: procedural indication<br><br>Analysis population: ITT<br><br>Subgroups: screening, surveillance positive FIT and diagnostic colonoscopy indications

		AI use: CADe, adjunct. Activated prior to insertion.<br><br>Histology: Vienna classification and 5th WHO classification used for histological assessment by two independent pathologists blinded to intervention	
Wallace 2022[42] (tandem RCT; USA, Italy, UK; N=230)	Inclusion: ≥45 years; screening or surveillance colonoscopy for CRC; average risk for CRC<br><br>Exclusion: pregnant women or women planning pregnancy; history of IBD, colon resection, FAP, serrated polyposis syndrome, overt lower GI bleeding, colonic stricture or radiation therapy to the abdomen; contraindications to colonoscopy (e.g., acute diverticulitis or toxic megacolon); symptoms requiring random biopsy of the colon.	Procedure: minimum 6 min withdrawal time; all detected polyps removed using standard polyp resection techniques; some endoscopist discretion permitted for diminutive (≤5 mm) polyps that appeared hyperplastic and were within 25 cm of the anus and biopsies could be taken where resection not feasible; use of VCE, NBI, LCI or other techniques including magnification or zoom was not permitted for detection but could be used for characterisation once detected; spray chromoendoscopy was not permitted; tandem procedures performed on same day by same endoscopist<br><br>AI use: CADe, adjunct. Activated prior to insertion.	Stratification: study endoscopist, age and colonoscopy indication<br><br>Analysis population: FAS/mITT[ab]<br><br>Subgroups: none

		Histology: Vienna classification or serrated lesion classification used by expert histopathologist blinded to intervention	
**MAGENTIQ-COLO™ (MAGENTIQ-EYE)**			
Maas 2024 – MAGENTIQ-COLO™[43] (parallel and tandem RCT; Germany, Israel, Netherlands, USA; N=916)	Inclusion: 18 to 90 years; scheduled for non- iFOBT screening (not referred following a positive iFOBT test) or surveillance colonoscopy if last colonoscopy performed at least 3 years prior to scheduled colonoscopy; able to provide written informed consent prior to study procedures; able to communicate clearly with investigators and study staff<br><br>Exclusion: known or suspected colorectal tumour or polyp; therapeutic colonoscopy (e.g. endoscopic mucosal resection, or intervention to stop lower GI bleeding); inadequately corrected anticoagulation use or disorder; pregnancy or potential pregnancy; inadequate bowel preparation (BBPS <6 or score <2 in any segment); known IBD; any clinically significant condition in opinion of investigator that would preclude study participation; unable or unwilling to comply with requirements of protocol; employees of investigator and study site or sponsor, or family members of these; new diagnosis of active colitis, polyposis syndrome, colonic stricture, or	Procedure: routine HD colonoscopy; distal devices excluded; minimum withdrawal time 6 to 10 min excluding interventions; diminutive polyps (≤5 mm) in the rectum could be left *in situ* if determined to be hyperplastic<br><br>AI use: CADe, adjunct. Activated prior to insertion. Version 1.0 with software version 1.7.2<br><br>Histology: each resected polyp sent for histopathological examination using Vienna classification; experienced pathologists performed assessments	Stratification: study site and colonoscopy indication<br><br>Analysis population: mITT[ac]<br><br>Subgroups: screening vs surveillance colonoscopy indication

	obstructing colorectal cancer not allowing complete colonoscopy		

[a]Stratification refers to stratification at randomisation, subgroups refers to colonoscopy indication or endoscopist experience subgroups

[b]Following excluded from analysis: colonic inflammation of >30 cm during colonoscopy, incomplete colonoscopy for any reason, incomplete recording or technical failure of AI system

[c]Following excluded from analysis: screening or randomisation failures, poor or inadequate bowel prep, inadequate withdrawal time or incomplete colonoscopy

[d]Following excluded from analysis: difficulty with deep insertion, poor bowel preparation and history of colorectal surgery

[e]Following excluded from analysis: those with insufficient bowel preparation

[f]Following excluded from analysis: no caecal intubation achieved or inadequate bowel preparation (<2 in any segment)

[g]Following excluded from analysis: ineligible age, IBD, BBPS <6 or incomplete examination

[h]Following excluded from analysis: unsuccessful caecal intubation or procedure cancellation

[i]Following excluded from analysis: inadequate or missing BBPS score (score <6), active colitis, polyposis syndrome, colonic stricture or obstructing CRC impeding a complete colonoscopy, those for whom a quality colonoscopy could not be performed, and participants in the Yaroslavl, Russia, study site enrolled after 24 February 2022

[j]Following excluded from analysis: those with inadequate bowel preparation, incomplete colonoscopy or where distal attachment device used

[k]Following excluded from mITT analysis: those with inadequate bowel preparation (BBPS <2 in any segment), missed caecal intubation or withdrawal time <6 min

[l]Following excluded from analysis: incomplete colonoscopy or poor bowel preparation (score 0 or 1 in any of three bowel sections)

[m]Following excluded from analysis: those with CRC, inadequate bowel preparation or IBD

[n]Following excluded from analysis: those with intestinal tuberculosis, IBD or insertion time too long

[o]Following excluded from analysis: video recording failed, unable to complete evaluation (e.g. due to inability to tolerate exam or unable to insert colonoscope) or researcher considered inappropriate for exclusion as too many polyps

[p]Following excluded from analysis: poor bowel preparation (score 0 to 1 in on BBPS any of three segments), patient discomfort, partial colectomy not reported, clinician decision, caecum not reached and computer hardware failure

[q]Following excluded from analysis: failed procedures, IBD, CRC or polyposis

[r]Following excluded from analysis: failed procedures, IBD, CRC

[s]Following excluded from analysis: caecum not reached in first or second procedure, CRC identified or other (unclear)

[t]Following excluded from analysis: failed procedures, IBD, CRC, polyposis or unable to make slice (presumably biopsy)

[u]Following excluded from analysis: those with misplaced consent forms

[v]Following excluded from analysis: poor bowel cleansing, pain/difficult colonoscopy/minor adverse events, tumour identified, pathology sample lost after sending

[w]Following excluded from analysis: incomplete colonoscopy or inadequate colon cleansing

[x]Following excluded from analysis: inadequate bowel cleansing

[y]Following excluded from analysis: prior to start of second colonoscopy due to adverse events, subject withdrawal, technical problems, incomplete colonoscopy due to poor bowel preparation, investigator decision, safety reasons or other reasons not defined

[z]Following excluded from analysis: inadequate bowel preparation, inability to examine colon, missing bowel preparation score data or not completing both tandem procedures for the tandem analyses

Abbreviations: ADR, adenoma detection rate; AI, artificial intelligence; ASA, American Society of Anesthesiologists; BBPS, Boston Bowel Preparation Scale; BCSP, Bowel Cancer Screening Programme; BLI, blue-light imaging; CADe, computer-aided detection; CADx, computer-aided characterisation; CRC, colorectal cancer; CT, computed tomography; CV, cardiovascular; EMIS™, Endoscopic Multimedia Information System; EPCAM, epithelial cell adhesion molecule gene; FAP, familial adenomatous polyposis; FAS, full analysis set; FIT, faecal immunochemical test; FOBT, faecal occult blood test; GI, gastrointestinal; HD, high-definition; IBD, inflammatory bowel disease; iFOBT, immunochemical faecal occult blood test; ITT, intention to treat; LCI, linked-colour imaging; mITT, modified intention to treat; MLH1, mutL homolog 1; MMR, mismatch repair; MSH2, mutS homolog 2; MSH6, mutS homolog 2; NA, not applicable; NBI, narrow-band imaging; NHS, National Health Service; PMS2, PMS1 homolog 2, mismatch repair system component; RCT, randomised controlled trial; VCE, virtual chromoendoscopy; WHO, World Health Organization.

1.1.2 Computer-aided polyp characterisation (CADx)

Supplementary Table 28. Summary of study characteristics – included clinical studies – CADx

Study (study design; country)	Inclusion criteria	Exclusion criteria	Colonoscopy procedure	Histology/reference standard and comparator	AI use	Analyses[a]
**CAD EYE® (Fujifilm Healthcare UK Ltd.)**						
Cassinotti 2023[44] (prospective NRS – abstract only; Italy; N=113 lesions)	UC patients undergoing endoscopic surveillance	Not reported	Not reported	Reference standard: histology<br><br>Comparison: WLI and LCI separately	CADe (adjunct) and CADx (unclear)[b]<br><br>CAD EYE®. Used with LCI. BLI also mentioned. Unclear when activated.	Exclusions: not reported<br><br>SSLs: not reported<br><br>No subgroup data
Djinbachian 2024[45] (parallel RCT; Canada; N=179 lesions)	45 to 80 years; undergoing colonoscopy between September 2022 and June 2023	Known coagulopathy (INR of >2.5 or platelet count of <50,000/mm^3); IBD; emergency colonoscopy; poor general health (ASA class of >3); trial record also suggests active colitis, familial	Polyps between 1 and 5 mm in size were analysed according to group randomly assigned to; HD endoscopes used.	Reference standard: histology by board-certified pathologists blinded to intervention<br><br>Comparison: none (autonomous AI but not prioritised given adjunct data available from this study)	CADx, adjunct<br><br>CAD EYE®. Unclear when CADx system activated. Polyp held at centre of screen for at least 5 seconds with consistent CADx diagnosis (neoplastic vs hyperplastic).	Exclusions: polyps >5 mm, unresected or unretrieved polyps, normal mucosa/inflammatory polyps or polyps where no OD performed<br><br>SSLs: serrated pathology could be

		polyposis syndrome and poor bowel prep (BBPS total score <6 and right segment score <2)			Endoscopist input based on WLI and BLI. Final diagnosis based on endoscopist judgement with high/low confidence noted.	assigned based on endoscopist judgement<br><br>No subgroup data but sensitivity analyses for high confidence diagnoses only and with removal of SSLs or advanced adenomas
Rondonotti 2023[46] (prospective NRS; Italy; N=550 lesions)	18 to 85 years; adults undergoing outpatient colonoscopy with at least one DRSP detected	history of CRC; hereditary polyposis syndromes or hereditary non-polyposis CRC; inadequate bowel preparation (i.e. BBPS <2 in at least one colonic segment); incomplete colonic examination (caecal intubation not achieved scheduled for partial examinations); polypectomy not performed due to ongoing anticoagulation; urgent	All polyps identified by endoscopist assessed in terms of size, location and morphology. Resected and sent for histology. All polyps ≤5 mm characterised, with each step using BLI. Magnification not used for OD. No prespecified observation time. BASIC criteria used by endoscopists to classify as adenoma or non-adenoma.	Reference standard: histology assessed by expert pathologists blinded to OD using Vienna classification. Second pathologist if disagreement between histology and high-confidence AI-assisted OD of DRSPs<br><br>Comparison: endoscopist OD alone (BASIC criteria)	CADx, adjunct<br><br>CAD EYE®. AI system switched on after endoscopist OD performed. Automatic classification by system (neoplastic vs hyperplastic) in BLI mode recorded. Only recorded when technically reliable and stable over time. Final diagnosis of adenoma or non-adenoma based on judgement of endoscopist on review of AI results.	Exclusions: low confidence OD, polyps not retrieved, polyps other than DRSPs<br><br>SSLs: considered within same group as hyperplastic polyps<br><br>Some subgroup data for expert vs non-expert endoscopists. Also sensitivity analyses for high vs low confidence diagnoses and rectosigmoid vs non-rectosigmoid location.

		colonoscopy scheduled.	Confidence level recorded (high or low).		Confidence level (high or low) noted.	
Sato 2024[47] (prospective NRS; Japan; N=380 lesions)	20 to 85 years; scheduled to undergo colonoscopy (positive FIT, symptoms such as abdominal pain or constipation, screening colonoscopy or where endoscopist otherwise deemed colonoscopy necessary); written informed consent provided	IBD; FAP; history of colorectal resection other than appendectomy; colorectal stenosis; pregnancy; abnormal blood coagulation function; inability to manage anticoagulation and antiplatelet medication according to Japanese guidelines; severe organ failure; inappropriate for enrolment by endoscopist	Colonoscopies routinely performed with magnification using HD colonoscopes. WLI for detection. OD using WLI non-magnified BLI and magnified BLI without CADx. CADx diagnoses using non-magnified BLI and magnified BLI. Paris, JNET and NICE classifications mentioned.	Reference standard: histology performed by at least two pathologists blinded to endoscopist assessments. Classification into neoplastic vs non-neoplastic.<br><br>Comparison: endoscopist assessment alone (autonomous AI also reported but not prioritised)	CADx, adjunct<br><br>CAD EYE®. Can be used with WLI and BLI with or without magnification. AI system switched on after endoscopist OD performed. Final assessment and confidence based on endoscopist review of AI diagnoses to supplement their own judgement. Confidence level (high or low) noted.	Exclusions: endoscopist classified as diminutive polyps in rectosigmoid colon, invasive cancers or submucosal tumours; no histological assessment; colonoscopies not performed<br><br>SSLs: considered within same group as hyperplastic polyps<br><br>Some subgroup data for expert vs non-expert endoscopists (≥1500 vs <1500 colonoscopies).
Taghiakbari 2025[48] (prospective NRS; Canada; N=138 lesions)	45 to 80 years; outpatient colonoscopy –	Not reported	BLI mode used with CADx. Magnification colonoscopes	Reference standard: expert review of videos by three experts (not	CADx, adjunct	Exclusions: polyps that did not undergo resect-and-discard or

	focuses on polyps that were not sent for histology		available for most colonoscopies – high magnification could be used when there were doubts about advanced histology for a diminutive lesion. WASP and JNET criteria used to identify serrated polyps and polyps with advanced histology, respectively.	histology, as polyps undergoing resect-and-discard and diagnose-and-leave approaches not sent for histology)<br><br>Comparison: none	CAD EYE®. Adenomatous or non-adenomatous classification.	diagnose-and-leave approach (i.e. where they were sent for histology instead); CADx-assisted OD not eventually performed<br><br>SSLs: likely excluded as these were supposed to be resected and sent for histology when identified by endoscopists<br><br>No subgroup data
**Discovery™ (Pentax Medical UK)**						
Lopez-Serrano 2024[49] (prospective NRS; Spain; N=61 lesions)	≥18 years; surveillance colonoscopy in UC patients at risk of CRC[c]; written informed consent to perform	personal history of CRC or high-grade dysplasia; previous colectomy (partial or complete); coagulopathy that prevents biopsies or polypectomy; colonoscopy performed in the	HD WLE used up to caecum, with no iSCAN processor settings selected. Two endoscopists worked in parallel, one assessing using VCE and one using AI system. Only suspicious areas were	Reference standard: histology according to Vienna classification by experienced GI pathologist<br><br>Comparison: VCE with iSCAN assessment	CADx, adjunct<br><br>Discovery™. Activated during withdrawal.	Exclusions: active inflammation during colonoscopy, inadequate bowel preparation, lost with no histology possible<br><br>SSLs: serrated adenomas with dysplasia classified as

	colonoscopy and deep sedation	previous 6 months; pregnant or nursing women; inadequate bowel preparation (stool remnants that could not be washed off, corresponding to BBPS <2 in at least one segment); presence of endoscopic UC activity (Mayo endoscopic subscore for UC ≥2); colonic stenosis; incomplete colonoscopy	resected for further analysis (mucosal irregularities not entirely secondary to chronic or active UC)	(Paris and Kudo pit classifications)		dysplastic, serrated adenomas without dysplasia grouped as non-dysplastic<br><br>No subgroup data
**GI Genius™ (Medtronic)**						
Bernhofer 2025[50] (prospective NRS; Austria; N=630 lesions)	≥18 years; elective colonoscopy for any reason performed by a trainee endoscopist; informed consent obtained	Uninterrupted oral anticoagulation; history of CRC; history of IBD; colonoscopy taken over by non-trainee endoscopist except for lesion resection purposes	Procedures performed with AI active using HD-WLE and NBI during withdrawal. OD based on NICE and JNET classifications. Trainees performed all procedures, but experts could support	Reference standard: histology mentioned, no further details<br><br>Comparison: none (autonomous AI and expert endoscopist assessment but not prioritised given	CADx, adjunct<br><br>GI Genius™ CADx module (version 3.0.0). Classification as adenomatous or non-adenomatous.	Exclusions: colonoscopy taken over by non-trainee endoscopist except for lesion resection purposes; missing histology

			with resections. All polyps resected and sent for histopathological evaluation.	adjunct AI use data available from this study and experts only reviewed videos)		SSLs: unclear how SSLs treated in the analysis<br><br>No subgroup data
Hassan 2022[51] (prospective NRS; Italy; n=544 lesions)	≥40 years; colonoscopy for primary CRC screening, post-polypectomy surveillance, post-FIT positive (20 µh haemoglobin/g faeces) or for symptoms/signs	Personal history of CRC or IBD (or hereditary polyposis or non-polyposis syndromes from trial record); previous colonic resection; emergency colonoscopy; antithrombotic therapy precluding polyp resection; lack of informed written consent	WLE used for detection of polyps. HD endoscopy systems used. CADx prediction performed first. Blue-light chromoendoscopy system then used for endoscopist OD. Confidence in OD recorded as high or low.	Reference standard: histology using Vienna classification by single expert pathologist blinded to AI and endoscopist diagnosis<br><br>Comparison: none (autonomous AI but not prioritised given adjunct data available from this study)	CADx, adjunct<br><br>GI Genius™. Version 3.0.0. No zoom or VCE permitted for CADx characterisation. White-light used.AI predictions include adenoma, non-adenoma and no prediction. Final diagnosis based on endoscopist judgement considering AI results and own assessment.	Exclusions: polyps not retrieved<br><br>SSLs: treated as non-adenomas in analysis<br><br>No subgroup data but scenarios with any confidence diagnoses included
Rondonotti 2024[52] (prospective NRS; Italy; N=376 lesions)	18 to 80 years; adult outpatients referred for screening, symptomatic or	Increased risk of harbouring adenomatous lesions (e.g., history of CRC, hereditary polyposis	Polyps other than 1 to 3 mm located in rectum with obvious hyperplastic appearance were	Reference standard: histology evaluated by expert pathologists blinded to OD. Vienna	CADx, adjunct<br><br>GI Genius™. Version 3.0.1. Non-magnified white-light. Final OD	Exclusions: polyps >5 mm, not retrieved for histology, high confidence

	post-polypectomy surveillance colonoscopy; at least one diminutive (≤5 mm) polyp detected	syndrome or hereditary non-polyposis colorectal cancer); newly diagnosed IBD; polypectomy not performed because of ongoing anticoagulation; urgent colonoscopy scheduled	resected and sent for histology. If AI detected polyps endoscopist had overlooked, asked to check marked area again. AI assessment performed first using white-light, with endoscopist able to use BLI to obtain AI-assisted OD. Confidence high or low noted.	and WHO classifications used.<br><br>Comparison: none (autonomous AI but not prioritised given adjunct data available from this study)	based on endoscopist judgement including interpretation of AI results and own assessment.	endoscopist OD could not be made<br><br>SSLs: treated as non-adenomatous in the analysis<br><br>Some subgroup data for expert vs non-expert endoscopists

[a]Only subgroup data relevant to the review protocol (i.e. colonoscopy indication or endoscopist experience) have been mentioned here

[b]Limited CADe data available so included in CADx tables only;

[c]UC requirements included: confirmed colonic disease by endoscopy and histology, involvement of ≥30% of the colonic surface or any extent if concomitant with primary sclerosing cholangitis and duration of disease >7 years (or any duration if concomitant with primary sclerosing cholangitis)

Abbreviations: AI, artificial intelligence; ASA, American Society of Anesthesiologists; BBPS, Boston Bowel Preparation Scale; BLI, blue-light imaging; CADe, computer-aided detection; CADx, computer-aided characterisation; CRC, colorectal cancer; DRSP, diminutive rectosigmoid polyp; FAP, familial adenomatous polyposis; FIT, faecal immunochemical test; GI, gastrointestinal; HD, high-definition; IBD, inflammatory bowel disease; INR, international normalised ratio; JNET, Japan Narrow Band Imaging Expert Team; LCI, linked-colour imaging; NBI, narrow-band imaging; NICE, NBI International Colorectal Endoscopic criteria; NRS, non-randomised study; OD, optical diagnosis; RCT, randomised controlled trial; SSL, sessile serrated lesion; UC, ulcerative colitis; VCE, virtual chromoendoscopy; WASP, Workgroup Serrated Polyps and Polyposis; WHO, World Health Organization; WLE, white-light endoscopy; WLI, white-light imaging.

1.2 Summary of patient characteristics

1.2.1 CADe

Supplementary Table 29. Summary of patient characteristics – included clinical studies - CADe

Study	Age	Sex	Colonoscopy indication	History of bowel conditions	Endoscopist experience	Bowel preparation score
**Argus® (Endosoft)**						
Strapko 2023[1] (parallel RCT – abstract only; USA; N=686)	AI: mean 63 (SD 9.9) years<br><br>No AI: mean 62.9 (SD 9.2) years	AI: 43.0% male<br><br>No AI: 48.0% male	Breakdown not reported	• History of CRC and adenomas unclear<br>• IBD excluded	Not reported	Not reported
**CAD EYE® (Fujifilm Healthcare UK Ltd.)**						
Alali 2025[2] (parallel RCT; Kuwait; N=102)	AI: mean 51.1 (SD 7.7) years	AI: 58.8% male<br><br>No AI: 41.2% male	• Screening, 94.1% AI vs 94.1% no AI<br>• Surveillance, 5.9% AI vs 5.9% no AI	• History of CRC and adenomas unclear<br>• IBD and prior resections excluded	• Experienced endoscopists (≥1000 colonoscopies) included	**Overall BBPS:**

	No AI: mean 54.5 (SD 8.3) years					AI: median 8.0 (IQR 6.0 to 9.0)<br><br>No AI: median 8.0 (IQR 6.0 to 9.0)<br><br>BBPS overall score ≥6 (≥2 each segment) required for inclusion
Aniwan 2023[3] (parallel RCT; Thailand; N=1,245)[a]	AI: mean 62.3 (SD 6.9) years<br><br>No AI: mean 62.1 (SD 6.9) years	AI: 42.7% male<br><br>No AI: 38.1% male	Full breakdown not reported.<br><br>11.6% (AI) and 10.2% (no AI) faecal occult blood positive	• Personal history of CRC excluded<br>• 13.4% (AI) and 11.7% (no AI) with family history of CRC<br>• History of adenomas unclear<br>• IBD excluded	• Average baseline ADR of 33.0%<br>• Included 7 staff attendings and 10 trainees under supervision<br>• 48.7% (AI) and 46.6% (no AI) performed by staff attendings	**Overall BBPS:**<br><br>AI: mean 8.0 (SD 1.3)<br><br>No AI: mean 8.1 (SD 1.2)
Desai 2024[4] (parallel RCT; USA; N=1,031)	AI: mean 58.9 (SD 9.5) years<br><br>No AI: mean 59.3	AI: 51.5% male<br><br>No AI: 48.3% male	• Screening, 49.9% AI vs 54.8% no AI;<br>• Surveillance for polyp history (≥3-year interval), 50.1% AI vs 45.2% no AI	• History of CRC and adenomas unclear<br>• IBD excluded	• ADR 25 to 40% required and ≥1000 colonoscopies	BBPS score ≥6 required for inclusion in analysis

	(SD 10.1) years					
Djinbachian 2024[5] (parallel RCT; Canada; N=467)	AI: mean 64.0 (IQR 8.4)<br><br>No AI: 64.1 (IQR 8.7)	AI: 49.8% male<br><br>No AI: 51.7% male	• FIT-positive, 6,6% AI vs 7.1% no AI;<br>• Screening, 14.0 AI vs 15.5% no AI<br>• Surveillance, 55.0% AI vs 52.5% no AI<br>• Diagnostic, 18.3% AI vs 21.8% no AI<br>• Other, 6.1% AI vs 2.9% no AI	• History of CRC and adenomas unclear<br>• Family history of CRC: 26.6% AI vs 26.4% no AI<br>• IBD and colitis excluded	• Board-certified gastroenterologists (n=4) or trainees (n=1)<br>• Diverse specialities (one expert in therapeutic endoscopy, one early career gastroenterologist specialised in IBD, one expert in neuromotility, one expert in genetics and hereditary polyposis and one fellow in first year of training)	84.3% in AI and 89.0% in no AI reported to have adequate bowel preparation – scale and threshold used to define this unclear
Hiratsuka 2025[6] (tandem RCT; Japan; N=94)	AI: mean 67.4 (SD 3.6)<br><br>No AI: 70.7 (SD 2.6)	AI: 64.6% male<br><br>No AI: 63.0% male	• Screening, 60.4% AI vs 58.7% no AI<br>• Symptomatic, 8.4% AI vs 17.4% no AI<br>• Surveillance, 31.2% AI vs 23.9% no AI	• CRC history: 37.5% AI vs 41.3% no AI<br>• History of adenomas not reported<br>• Prior colectomy excluded<br>• FAP or other polyposis excluded<br>• IBD or other bowel conditions unclear	• Expert (≥10 years' endoscopy experience) and non-expert (<10 years' endoscopy experience) permitted<br>• 39.6% in AI and 43.5% in non-AI groups performed by experts	**Overall BBPS:**<br><br>AI: mean 8.52 (SD 0.62)<br><br>No AI: mean 8.4 (SD 0.8)
Huneburg 2023[7] (parallel RCT; Germany; N=96)	AI: mean 50.3 (SD 11.9) years	AI: 40.0% male<br><br>No AI: 45.6% male	Specific to Lynch syndrome patients:	• 44.0% (AI) and 39.1% (no AI) with personal history of CRC and colon surgery	• Extensive experience in Lynch syndrome endoscopic surveillance	BBPS score ≥2 in all segments required for inclusion

	No AI: mean 46.3 (SD 11.8) years		• MLH1 variant: 34.0% AI vs 37.0% no AI<br>• MSH2 variant: 50.0% AI vs 50.0% no AI<br>• MSH6 variant: 16.0% AI vs 13.0% no AI	• 22.0% (AI) and 17.4% (no AI) with history of adenomas<br>• 44<br>• IBD or other bowel conditions unclear	• >1000 total colonoscopies and >300 colonoscopies in Lynch Syndrome	
Miyaguchi 2024[8] (parallel RCT; Japan; N=800)	AI: mean 65.1 (95% CI, 63.7 to 66.5) years<br><br>No AI: mean 66.1 (95% CI, 64.7 to 67.4) years	AI: 55.8% male<br><br>No AI: 57.3% male	• FIT positive, 34.0% AI vs 32.2% no AI;<br>• Symptomatic, 20.8% AI vs 20.5% no AI<br>• Polyp surveillance, 18.8% AI vs 19.3% no AI<br>• Screening, 26.5% AI vs 28.0% no AI	• History of CRC and adenomas unclear<br>• 20.0% (AI) and 18.1% (no AI) with prior abdominal surgery<br>• 27.7% in both groups with diverticulitis<br>• 18.1% (AI) and 16.9% (no AI) with ulcerative colitis	• Experts (>1000 colonoscopies) and trainees (<1000 colonoscopies) included<br>• 64.5% (AI) and 62.8% (no AI) of procedures performed by experts	Aronchick *et al.* score of fair or good required for inclusion
Nakashima 2023[9] (tandem RCT; Japan; N=415)	AI: mean 54.9 (SD 10.9) years<br><br>No AI: mean 55.9 (SD 10.4) years	AI: 73.9% male<br><br>No AI: 69.7% male	• Screening, 7.2% AI vs 8.2% no AI<br>• FIT positive, 56.5% AI vs 51.9% no AI<br>• Surveillance, 36.2% AI vs 39.9% no AI	• History of CRC and adenomas unclear<br>• IBD excluded	• Board Certified Trainers of the Japan Gastroenterological Endoscopy Society. Experienced.	**Overall BBPS:**<br><br>AI: mean 8.61 (SD 0.69)<br><br>No AI: mean 8.68 (SD 0.67)
Rondonotti 2022[10] (parallel RCT; Italy; N=800)	AI: median 62.0 (IQR	AI: 52.6% male	All FIT positive	• History of CRC and adenomas excluded	• Qualified to work in the FIT-based screening programme	BBPS score ≥2 in all segments

	56 to 68) years<br><br>No AI: median 61.0 (IQR 55 to 67) years	No AI: 49.6% male		• IBD excluded	(300+ colonoscopies/year, caecal intubation rate ≥95%, ADR ≥25%).<br>• 30-min training using CADe system and performed ≥10 colonoscopies using system before entry	required for inclusion
Scholer 2024[11] (parallel RCT; Sweden; N=193)[b]	AI: mean 65.9 (SD 11.5) years<br><br>No AI: mean 66.8 (SD 11.5) years	AI: 53.0% male<br><br>No AI: 47.0% male	• Screening, 1.0% in both groups<br>• Alarm symptoms (iron-deficiency anaemia), 58.0% AI vs 53% no AI<br>• Inconclusive CT findings, 2.0% AI vs 5.0% no AI<br>• Other (positive FOBT, polyp surveillance, hereditary CRC, diarrhoea, etc.), 39.0% AI vs 41.0% no AI	• History of CRC and adenomas unclear<br>• IBD or other bowel conditions unclear	• Experienced and inexperienced included (inexperienced defined as those with fewer than 400 prior colonoscopies)<br>• 70.5% (AI) and 84.7% (no AI) procedures performed by experienced endoscopists	**Overall BBPS:**<br><br>AI: mean 8.2 (SD 1.2)<br><br>No AI: mean 8.3 (SD 1.1)<br><br>BBPS score ≥2 in all segments required for inclusion
Tiankanon 2024[12] (parallel RCT; Thailand; N=800)	AI: mean 63.2 (SD 6.5) years<br><br>No AI: mean 62.2	AI: 35.8% male<br><br>No AI: 38.5% male	• FIT positive, 11.3% AI vs 16.0% no AI<br>• Routine screening, 88.8% AI vs 84.0% no AI	• History of CRC excluded<br>• History of adenomas unclear<br>• IBD excluded<br>• 7.5% (AI) and 10.5% (no AI) with family CRC history	• Baseline ADR ≥35% required from mix of primary colonoscopies and FIT positive colonoscopies within last 5 years across ≥100 procedures<br>• Baseline average all endoscopists was 42.6%	**Overall BBPS:**<br><br>AI: 9.0 (8.0 to 9.0) – assume median and IQR

	(SD 7.3) years				• Complete ≥20 CADe procedures before study initiation<br>• Includes attending physicians and trainees - 38.8% (AI) and 39.3% (no AI) procedures performed by attending physicians	No AI: 9.0 (8.0 to 9.0) – assume median and IQR
Yamaguchi 2024[13] (parallel RCT; Japan; N=231)	AI: mean 63.1 (SD 10.8) years<br><br>No AI: mean 63.3 (SD 11.8) years	AI: 73.9% male<br><br>No AI: 69.7% male	• FIT positive, 46.9% AI vs 54.2% no AI<br>• Detailed examination, 19.5% AI vs 22.0% no AI<br>• Polyp surveillance, 13.3% AI vs 11.0% no AI<br>• Abdominal symptoms, 11.5% AI vs 8.5% no AI<br>• Other, 8.9% AI vs 4.2% no AI	• History of CRC and adenomas unclear<br>• 11.5% (AI) and 11.0% (no AI) with family history of CRC<br>• IBD excluded	• Trainees in third or fourth year as physician with limited colonoscopy experience (0 to 20 cases)<br>• Performed in conjunction with gastroenterology experts (>5000 prior colonoscopies)	**Overall BBPS:**<br><br>AI: mean 8.4 (SD 1.2)<br><br>No AI: mean 8.2 (SD 1.5)<br><br>98.2% (AI) and 95.8% with fair, good or excellent rating on Modified Aronchick scale.

Zimmermann-Fraedrich 2025[14] (parallel RCT; Germany; N=1,627)	AI: mean 63.0 (SD 8.8) years<br><br>No AI: mean 63.2 (SD 8.7) years	AI: 59.9% male<br><br>No AI: 60.5% male	• Screening, 71.9% AI vs 71.0% no AI<br>• Diagnostic (polyp follow-up and symptom evaluation), 28.1% AI vs 29.0% no AI	• History of CRC and adenomas unclear<br>• IBD or other bowel conditions unclear	• Experienced examiners included (not defined)	**Overall BBPS – simplified version used, scale unclear:**<br><br>AI: median 3.0 (IQR 2.0 to 3.0) – 98.2% adequate<br><br>No AI: median 3.0 (IQR 2.0 to 3.0) – 97.4% adequate
**Discovery™ (Pentax Medical UK)**						
Maas 2024 - Discovery™[15] (parallel RCT; Canada, France, Germany, Italy, Netherlands, Russia; N=497)	AI: median 61.0 (IQR 52 to 69) years<br><br>No AI: median 61.0 (IQR 52 to 69) years	AI: 43.6% male<br><br>No AI: 44.9% male	• Screening (non-iFOBT), 20.0% AI vs 18.6% no A<br>• Surveillance, 39.2% AI vs 42.1% no AI<br>• Diagnostic, 40.8% AI vs 39.3% no AI	• History of CRC and adenomas unclear<br>• 24.0% (AI) and 18.2% (no AI) with family history of CRC<br>• IBD excluded	• Experienced endoscopists with at least 500 prior colonoscopies requirement (all had at least 2000)<br>• Training with CADe with minimum of five procedures before study start	Total BBPS score ≥6 required for inclusion

ENDO-AID™ (Olympus Medical Systems Corp.)						
Gimeno-Garcia 2023[16] (parallel RCT; Tenerife; N=370)	AI: mean 62.99 (SD 10.26) years<br><br>No AI: mean 64.71 (SD 11.79) years	AI: 52.9% male<br><br>No AI: 52.9% male	• Average risk population screening, 31.6% AI vs 35.7% no AI<br>• Post-polypectomy surveillance, 32.9% AI vs 31.8% no AI<br>• Rectal bleeding, 12.9% AI vs 10.2% no AI<br>• Anaemia, 9.0% AI vs 7.6% no AI<br>• Familial CRC screening, 5.8% AI vs 6.4% no AI<br>• Change in bowel habits, 3.9% AI vs 3.2% no AI<br>• Chronic diarrhoea, 2.6% AI vs 3.2% no AI<br>• Suspicion of CRC, 1.3% AI vs 1.9% no AI	• History of CRC excluded<br>• History of adenomas unclear	• High (ADR ≥40%) and low (ADR <40%) detectors included<br>• >2000 lifetime colonoscopies required	**Overall BBPS:**<br><br>AI: mean 7.37 (SD 1.4)<br><br>No AI: mean 7.23 (SD 1.2)<br><br>85.4% in both groups with BBPS score ≥6 and score ≥2 in each segment
Lau 2024[17] (parallel RCT; Hong Kong; N=766)	AI: mean 66.0 (SD 10.1) years<br><br>No AI: mean 65.4	AI: 53.1% male<br><br>No AI: 55.5% male	• Screening, 7.3% AI vs 6.1% no AI<br>• Surveillance, 32.6% AI vs 31.8% no AI<br>• Symptomatic, 60.1% AI vs 62.1% no AI	• History of CRC excluded<br>• 19.5% (AI) and 15.6% (no AI) with family history of CRC<br>• History of adenomas unclear<br>• IBD excluded	• Endoscopists in training with <500 procedures and <3 years' experience (gastroenterologists or surgeons in training)<br>• Of these, beginners and intermediates included	**Overall BBPS:**<br><br>AI: mean 7.85 (SD 1.2)

	(SD 11.3) years				(beginner <200 procedures, intermediate 200 to 500 procedures)<br>• All performed at least 20 colonoscopies under supervision and received training on CADe system before study initiation<br>• 28.5% (AI) and 27.6% (no AI) performed by beginners	No AI: mean 7.84 (SD 1.2)<br><br>100.0% in both groups with BBPS score ≥2 in each segment
Lui 2024[18] (parallel RCT; Hong Kong; N=682)[c]	AI: mean 65.2 (SD 10.2) years<br><br>No AI: mean 65.5 (SD 10.7) years	AI: 53.2% male<br><br>No AI: 50.5% male	• Screening, 16.7% AI vs 21.0% no AI<br>• Surveillance, 28.0% AI vs 29.0% no AI<br>• Diagnostic, 55.3% AI vs 50.0% no AI	• History of CRC excluded<br>• History of adenomas unclear<br>• IBD excluded	• Experienced (>7 years) and less experienced included<br>• Range 1 to 23 years' experience, historical ADR range 30 to 53%<br>• 45.9% (AI) and 40.7% (no AI) had procedures performed by experienced endoscopists	**Overall BBPS:**<br><br>AI: mean 7.1 (SD 1.7)<br><br>No AI: mean 7.1 (SD 1.8)
Spada 2025[19] (parallel RCT; Italy; N=1,228)	AI: mean 62.3 (SD 10.5) years<br><br>No AI: mean 61.9 (SD 10.0) years	AI: 50.9% male<br><br>No AI: 47.3% male	• Post-polypectomy surveillance, 19.6% AI vs 17.8% no AI<br>• Direct screening, 38.0% AI vs 38.1% no AI<br>• FIT-positive, 17.2% AI vs 20.3% no AI	• History of CRC unclear;<br>• 19.6% AI and 17.8% no AI with prior polypectomies (unclear if all had at least one adenoma)<br>• IBD excluded<br>• Prior colon resection excluded	• Experienced endoscopists (>2000 examinations)<br>• Minimum of 5 CADe procedures to familiarise with system prior to study	94.6% AI and 94.0% no AI considered to have adequate BBPS overall score (≥6)

			• Symptoms, 25.2% AI vs 23.7% no AI	• Polyposis syndromes excluded		
Vilkoite 2023[20] (parallel RCT; Latvia; N=400)	AI: 50.1 (15.4) years – assume mean and SD<br><br>No AI: 51.2 (14.5) years – assume mean and SD	AI: 46.9% male<br><br>No AI: 49.5% male	Breakdown not reported	• History of CRC excluded<br>• Prior colonoscopy excluded (and presumably prior adenomas)<br>• IBD excluded	• Two endoscopists with average of 2000 colonoscopies pr year (one 8 years' experience, one >15 years' experience)	BBPS score ≥2 in each segment required for inclusion in analysis
**ENDOANGEL® Lower Gastrointestinal Endoscope Image Auxiliary Diagnostic Equipment (Wuhan ENDOANGEL Medical Technology Co. Ltd.)**						
Gong 2020[21] (parallel RCT; China; N=704)	AI: 50.0 (37 to 58) years – assume median and IQR<br><br>No AI: 49.0 (36 to 57) years – assume	AI: 53.0% male<br><br>No AI: 45.0% male	• Health examination, 17.0% AI vs 18.0% no AI<br>• Diagnostic, 79.0% AI vs 76.0% no AI<br>• Surveillance, 4.0% AI vs 6.0% no AI	• History of CRC excluded<br>• History of adenomas unclear<br>• IBD excluded	• Endoscopists with experience of 1 to 3 years and total colonoscopy volume 1500 to 4000 procedures	94.1% (AI) and 93.7% (no AI) had total BBPS score ≥6 and score of ≥2 in each segment

	median and IQR					
Yao 2022[22] (parallel RCT; China; N=539)	AI: mean 50.69 (SD 13.15) years<br><br>No AI: mean 50.85 (SD 13.56) years	AI: 45.2% male<br><br>No AI: 42.1% male	• Screening, 88.81% AI vs 88.93% no AI<br>• Diagnostic, 0.75% AI vs 1.11% no AI<br>• Surveillance, 10.45% AI vs 9.96% no AI	• History of CRC excluded<br>• 5.6% (AI) and 4.8% (no AI) with family history of CRC<br>• History of adenomas unclear<br>• IBD excluded	• Experienced endoscopists with >2000 prior screening colonoscopies	84.7% (AI) and 85.2% (no AI) with BBPS score ≥2 in all segments
Yao 2024[23] (tandem RCT; China; N=456)[d]	AI: mean 50.6 (SD 12.3) years<br><br>No AI: mean 49.9 (SD 11.7) years	AI: 51.5% male<br><br>No AI: 53.7% male	• Screening, 63.9% AI vs 63.8% no AI<br>• Diagnostic, 26.4% AI vs 28.0% no AI<br>• Surveillance, 9.7% AI vs 8.3% no AI	• History of CRC excluded<br>• 8.8% (AI) and 7.0% (no AI) with family history of CRC<br>• History of adenomas unclear<br>• IBD excluded	• Novices (>1 year gastroenterology fellowship experience and no prior experience or training in colonoscopy)<br>• Hands on teaching with 20 colonoscopies before study initiation<br>• Tandem procedures performed by experts with >5000 to >15000 prior colonoscopies	86.3% (AI) and 88.2% (no AI) with BBPS total score ≥6 and score ≥2 in all segments
Zhang 2023[24] (parallel RCT; China; N=1,293)	AI: mean 52.9 (SD 12.4) years	AI: 40.4% male	Breakdown not reported	• History of CRC excluded<br>• 17.0% (AI) and 17.2% (no AI) with family history of CRC	• >1 year colonoscopy experience and at least 100 prior colonoscopies	**Overall BBPS:**

	No AI: mean 53.2 (SD 12.4) years	No AI: 44.8% male		• History of adenomas unclear<br>• IBD excluded		AI: mean 7.2 (SD 1.3)<br><br>No AI: mean 7.2 (SD 1.4)
**EndoScreener® (WISION AI)**						
Glissen Brown 2022[25] (tandem RCT; USA; N=223)	AI: mean 61.2 (SD 9.8) years<br><br>No AI: mean 60.5 (SD 8.5) years	AI: 47.8% male<br><br>No AI: 61.8% male	• Screening, 60.2% AI vs 59.1% no AI<br>• Surveillance, 39.8% AI vs 40.9% no AI	• History of CRC and adenomas unclear<br>• IBD excluded	• Experienced endoscopists with high baseline ADR (first-pass ADR in no AI first group was 44.0%)	**Overall BBPS:**<br><br>AI: 9.0 (IQR 8.0 to 9.0) – assume median<br><br>No AI: 9.0 (IQR 8.0 to 9.0) – assume median<br><br>96.5% (AI) and 98.2% (no AI) with total BBPS score ≥6 and score ≥2

						in each segment
Liu 2020[26] (parallel RCT; China; N=790)	AI: mean 49.8 (SD 13.1) years<br><br>No AI: mean 48.8 (SD 13.0) years	AI: 45.8% male<br><br>No AI: 48.9% male	• Screening, 24.9% AI vs 21.2% no AI<br>• Symptomatic, 75.1% AI vs 78.8% no AI	• History of CRC excluded<br>• 9.2% (AI) and 5.8% (no AI) with family history of CRC<br>• 6.9% (AI) and 7.1% (no AI) with history of adenomas<br>• IBD excluded	• Experienced and junior endoscopists included<br>• 30.5% (AI) and 32.0% (no AI) performed by senior doctor	**Overall BBPS:**<br><br>AI: mean 6.7 (SD 1.5)<br><br>No AI: mean 6.7 (SD 1.4)<br><br>79.1% (AI) and 84.1% (no AI) with total BBPS score ≥6 and score ≥2 in each segment
Wang 2019[27] (parallel RCT; China; N=1,058)	AI: mean 51.1 (SD 13.2) years<br><br>No AI: mean 49.4	AI: 50.4% male<br><br>No AI: 46.5% male	• Screening, 7.7% AI vs 8.2% no AI<br>• Symptomatic, 92.3% AI vs 91.8% no AI	• History of CRC excluded<br>• 6.1% (AI) and 6.5% (no AI) with family history of CRC<br>• 2.9% (AI) and 2.6% (no AI) with history of adenomas	• Experienced and junior endoscopists included<br>• Juniors defined as those with between 100 and 500 colonoscopies, experienced had at least 3000 prior colonoscopies	**Overall BBPS:**<br><br>AI: mean 6.6 (SD 1.2)

	(SD 13.8) years			• IBD excluded	• 38.9% (AI) and 43.5% (no AI) performed by senior doctor	No AI: mean 6.6 (SD 1.3)<br><br>86.0% (AI) and 85.3% (no AI) with total BBPS score ≥6 and score ≥2 in each segment
Wang 2020 (effect of a deep…)[28] (parallel RCT; China; N=962)	AI: median 49.0 (IQR 39.0 to 60.0) years<br><br>No AI: median 49.0 (IQR 40.3 to 56.0) years	AI: 50.0% male<br><br>No AI: 53.0% male	• Screening, 17.0% AI vs 16.0% no AI<br>• Symptomatic, 83.0% AI vs 84.0% no AI	• History of CRC excluded<br>• 8.7% (AI) and 8.0% (no AI) with family history of CRC<br>• 9.9% (AI) and 8.0% (no AI) with history of adenomas<br>• IBD excluded	• Senior endoscopists with ≥5 years' experience and ≥1000 colonoscopies per year	**Overall BBPS:**<br><br>AI: mean 6.8 (SD 1.4)<br><br>No AI: mean 7.0 (SD 1.4)<br><br>85.0% (AI) and 86.0% (no AI) with total BBPS score ≥6 and score ≥2 in each segment

Wang 2020 (lower adenoma miss…)[29] (tandem RCT; China; N=369)	AI: mean 47.7 (SD 10.8) years<br><br>No AI: mean 47.2 (SD 10.4) years	AI: 50.5% male<br><br>No AI: 46.5% male	• Screening, 31.5% AI vs 29.8% no AI<br>• Symptomatic, 58.2% AI vs 63.2% no AI<br>• Surveillance, 10.3% AI vs 7.0% no AI	• History of CRC excluded<br>• 7.1% (AI) and 7.0% (no AI) with family history of CRC<br>• 5.4% (AI) and 3.2% (no AI) with history of adenomas<br>• IBD excluded	• Experienced endoscopists (no further details)	**Overall BBPS:**<br><br>AI: mean 7.1 (SD 1.4)<br><br>No AI: mean 7.2 (SD 1.4)<br><br>86.4% (AI) and 87.0% (no AI) with total BBPS score ≥6 and score ≥2 in each segment
Wang 2023[30] (parallel RCT; China; N=1,261)	AI: median 46.0 (IQR 36.8 to 54.0) years<br><br>No AI: median 47.0	AI: 57.2% male<br><br>No AI: 52.2% male	• Screening, 17.6% AI vs 16.3% no AI<br>• Symptomatic, 76.6% AI vs 78.4% no AI<br>• Surveillance, 5.8% AI vs 5.3% no AI	• History of CRC excluded<br>• 5.4% (AI) and 6.2% (no AI) with family history of CRC<br>• 2.0% (AI) and 3.0% (no AI) with history of adenomas<br>• IBD excluded	• Experienced endoscopists (>2000 screening colonoscopies)	**Overall BBPS:**<br><br>AI: mean 6.8 (SD 1.3)<br><br>No AI: mean 6.9 (SD 1.3)

	(IQR 37.0 to 55.0) years					83.0% (AI) and 81.4% (no AI) with total BBPS score ≥6 and score ≥2 in each segment
**GI Genius™ (Medtronic)**						
Ahmad 2023[31] (parallel RCT; UK; N=614)	AI: mean 66.2 (SD 5.4) years<br><br>No AI: mean 66.4 (SD 5.4) years	AI: 35.7% male<br><br>No AI: 32.0% male	All within NHS BCSP, breakdown of specific indications not reported	• History of CRC and adenomas unclear<br>• 5.4% (AI) and 6.2% (no AI) with family history of CRC<br>• 2.0% (AI) and 3.0% (no AI) with history of adenomas<br>• IBD excluded	• Experienced endoscopists working within NHS bowel cancer screening centre<br>• Performed between 46 and 109 screening colonoscopies with PDR between 63 and 85% (and ADR between 56 and 80%)	Not reported
Engelke 2023[32] (parallel RCT; Sweden; N=232)	AI: median 66.0 (IQR 49.0 to 76.0) years<br><br>No AI: median 67.0	AI: 47.0% male<br><br>No AI: 51.0% male	• Primary screening, 3.3% AI vs 4.5% no AI<br>• Pre-planned polypectomy, 5.7% AI vs 1.8% no AI<br>• Tumour follow-up, 4.1% AI vs 3.6% no AI	• History of CRC and adenomas unclear<br>• 5.4% (AI) and 6.2% (no AI) with family history of CRC	• Trained endoscopists<br>• Those with >200 colonoscopies annually and >50 polypectomies annually in last 3 years considered experts	**Overall BBPS:**<br><br>AI: median 8.0 (IQR 5.0 to 9.0)

	(IQR 51.0 to 78.0) years		• Diagnostic, 86.9% AI vs 90.0% no AI	• 2.0% (AI) and 3.0% (no AI) with history of adenomas<br>• 6.5% of whole trial had IBD as indication (unclear if others had IBD where not primary indication)	• 59.0% (AI) and 53.0% (no AI) performed by experienced investigator	No AI: mean 7.0 (IQR 5.0 to 9.0)<br><br>58.2% (AI) and 60.0% (no AI) with total BBPS score ≥6 and score ≥2 in each segment
Karsenti 2023[33] (parallel RCT; France; N=2,015)	AI: mean 58.4 (SD 11.4) years<br><br>No AI: mean 58.4 (SD 11.8) years	AI: 48.0% male<br><br>No AI: 49.2% male	• Family history of polyps or cancer, 17.2% AI vs 17.4% no AI<br>• Personal history of polyps or cancer, 24.8% AI vs 27.5% no AI<br>• Individual screening, 13.8% AI vs 16.1% no AI<br>• FIT positive, 7.9% AI vs 6.3% no AI<br>• Haematochezia, 10.6% AI vs 9.3% no AI	• History of CRC and adenomas unclear – but some had this as indication for colonoscopy<br>• IBD excluded	• Experienced endoscopists with history of >2000 colonoscopies	98.0% (AI) and 97.8% (no AI) with total BBPS score ≥6 and score ≥2 in each segment

			• Digestive symptoms, 16.4% AI vs 16.0% no AI<br>• Other, 9.4% AI vs 7.4% no AI			
Lagstrom 2025[34] (parallel RCT; Denmark; N=795)	AI: mean 64.0 (SD 11.1) years<br><br>No AI: mean 63.4 (SD 11.1) years	AI: 55.8% male<br><br>No AI: 50.6% male	• Diagnostic (cancer suspected), 17.0% AI vs 27.3% no AI<br>• Diagnostic (benign disease suspected), 12.3% AI vs 9.9% no AI<br>• FIT-positive (>100 µg/L), 40.0% AI vs 40.5% no AI<br>• Post-polypectomy surveillance, 22.3% AI vs 14.2% no AI<br>• Post-CRC surgery control, 3.3% AI vs 3.5% no AI<br>• HNPCC control, 2.5% AI vs 2.5% no AI<br>• Diverticulitis follow-up, 2.8% AI vs 2.0% no AI	• History of CRC not excluded (3.3% AI and 3.5% no AI with prior CRC surgery)<br>• 22.3% AI and 14.2% no AI with prior polypectomies (unclear if all had at least one adenoma)<br>• IBD control colonoscopies excluded<br>• Other bowel conditions unclear	• Experts (>1000 colonoscopies) and non-experts (≤1000 colonoscopies) permitted<br>• 83.3% in AI and 79.2% in no AI group has procedure performed by expert	56.8% and 54.7% of those randomised has bowel preparation of “good” on Modified Aronchick score
Levartovsky 2023[35] (retrospective study – abstract	Pre-AI: median 43.8	Pre-AI: 55.3% males	All patients had IBD (colonoscopies for IBD	• History of CRC and adenomas unclear<br>• IBD patients included (100%)	• Experienced and inexperienced endoscopists appear to be included<br>• No definitions provided	Not reported

only; Israel; N=996)	(IQR 28.7 to 61.2) years<br><br>AI: median 44.5 (IQR 30.7 to 59.1)years	AI: 54.3% males	severity assessment excluded)<br><br>62.9% (pre-AI) and 57.2% (AI) Crohn's disease			
Mangas-Sanjuan 2023[36] (parallel RCT; Spain; N=3,213)	AI: mean 60.7 (SD 5.8) years<br><br>No AI: mean 60.6 (SD 5.7) years	AI: 53.7% male<br><br>No AI: 53.2% male	All within FIT-based screening programme	• History of CRC excluded<br>• History of adenomas unclear<br>• IBD excluded	• ≥10 procedures with CADe prior to recruitment<br>• Unclear experience but all FIT-based screening	**Overall BBPS:**<br><br>AI: mean 7.8 (SD 1.3)<br><br>No AI: mean 7.8 (SD 1.3)<br><br>All required BBPS score of ≥2 in each segment to be included in analysis
Ortiz 2024[37] (parallel RCT; Belgium, Germany, Italy, Spain; N=430)	AI: mean 47.8 (SD 14.0) years	AI: 41.0% male<br><br>No AI: 40.0% male	All surveillance for Lynch syndrome<br>• MLH1, 35.0% AI vs 30.0% no AI	• 20.0% (AI) and 19.0% (no AI) with history of CRC<br>• History of adenomas unclear	• ADR ≥20% screening and ≥35% FIT screening<br>• ≥2000 colonoscopies	96.0% (AI) and 97.0% (no AI) with BBPS total score ≥6

	No AI: mean 50.1 (SD 14.5) years		• MSH2, 42.0% AI vs 44.0% no AI<br>• MSH6, 22.0% AI vs 22.0% no AI<br>• EPCAM, 1.0% AI vs <1.0% no AI	• 29.0% (AI) and 24.0% (no AI) with prior non-CRC neoplasia<br>• IBD excluded	• Trained in optical diagnosis and chromoendoscopy techniques<br>• ≥10 colonoscopies with CADe prior to study	and score ≥2 in each segment
Repici 2020[38] (parallel RCT; Italy; N=685)	AI: mean 61.5 (SD 9.7) years<br><br>No AI: mean 61.1 (SD 10.6) years	AI: 50.4% male<br><br>No AI: 48.0% male	• FIT positive, 29.9% AI vs 30.5% no AI<br>• Primary CRC screening, 22.6% AI vs 22.1% no AI<br>• Surveillance, 25.2% AI vs 22.7% no AI<br>• GI symptoms, 22.3% AI vs 24.7% no AI	• History of CRC excluded<br>• History of adenomas unclear<br>• IBD excluded	• Experienced endoscopists - >2000 colonoscopies	99.4% in both groups with BBPS score ≥2 in each segment
Repici 2022[39] (parallel RCT; Italy, Switzerland; N=660)	AI: mean 61.9 (SD 9.8) years<br><br>No AI: mean 62.6 (SD 10.2) years	AI: 52.7% male<br><br>No AI: 47.3% male	• FIT positive, 7.3% AI vs 7.3% no AI<br>• Primary CRC screening, 29.7% AI vs 28.5% no AI<br>• Surveillance, 38.5% AI vs 35.7% no AI<br>• GI symptoms, 24.5% AI vs 28.5% no AI	• History of CRC excluded<br>• 38.5% in both groups with history of adenomas<br>• IBD excluded	• Non-expert endoscopists - <2000 colonoscopies<br>• Eligible to perform screening colonoscopies and non-complex therapeutic procedures autonomously (not novices)	99.7% (AI) and 99.4% (no AI) with BBPS score >2 in each segment
Scholer 2024[11] (parallel RCT; Sweden; N=47)[b]	AI: mean 65.9 (SD 11.5) years	AI: 53.0% male	• Screening, 1.0% in both groups	• History of CRC and adenomas unclear<br>• IBD or other bowel conditions unclear	• Experienced and inexperienced included (inexperienced defined as	**Overall BBPS:**

	No AI: mean 66.8 (SD 11.5) years	No AI: 47.0% male	• Alarm symptoms (iron-deficiency anaemia), 58.0% AI vs 53% no AI<br>• Inconclusive CT findings, 2.0% AI vs 5.0% no AI<br>• Other (positive FOBT, polyp surveillance, hereditary CRC, diarrhoea, etc.), 39.0% AI vs 41.0% no AI		those with fewer than 400 prior colonoscopies)<br>• 70.5% (AI) and 84.7% (no AI) procedures performed by experienced endoscopists	AI: mean 8.2 (SD 1.2)<br><br>No AI: mean 8.3 (SD 1.1)<br><br>BBPS score ≥2 in all segments required for inclusion
Seager 2024[40] (parallel RCT; UK; N=2,032)	AI: mean 62.5 (SD 10.8) years<br><br>No AI: mean 62.2 (SD 10.8) years	AI: 55.9% male<br><br>No AI: 55.6% male	• Screening – FIT positive, 60.4% AI vs 60.8% no AI<br>• Symptomatic, 39.6% AI vs 39.2% no AI	• History of CRC and adenomas unclear<br>• IBD or other bowel conditions unclear	• Pre-trial CADe training<br>• Median 10 years as independent colonoscopist<br>• 49.3% BCSP accredited<br>• Qualified to perform colonoscopy independent of supervision	Not reported
Thiruvengadam 2024[41] (parallel RCT; USA; N=1,100)	AI: median 56.0 (IQR 50.0 to 62.0) years<br><br>No AI: median 54.0	AI: 40.7% male<br><br>No AI: 37.3% male	• Screening, 69.3% AI vs 65.3% no AI<br>• Surveillance, 6.0% AI vs 10.0% no AI<br>• FIT positive, 5.8% AI vs 5.8% no AI<br>• Diagnostic, 18.9% AI vs 18.9% no AI	• History of CRC and adenomas unclear<br>• IBD or other bowel conditions unclear	• ≥1000 colonoscopies<br>• Baseline ADR ≥25%<br>• No trainees included<br>• Up to 5 colonoscopies with CADe before study initiation	91.5% (AI) and 90.4% (no AI) with BBPS total score ≥6 and score ≥2 in each segment

	(IQR 49.0 to 62.0) years					
Wallace 2022[42] (tandem RCT; USA, Italy, UK: N=230)	AI: mean 63.0 (SD 8.2) years<br><br>No AI: mean 64.6 (SD 8.1) years	AI: 69.0% male<br><br>No AI: 67.5% male	• Screening, 35.3% AI vs 36.0% no AI<br>• Surveillance <3 years, 10.3% AI vs 12.3% no AI<br>• Surveillance 3 to 10 years, 54.3% AI vs 51.8% no AI	• History of CRC and adenomas unclear<br>• IBD excluded	• ≥1000 colonoscopies<br>• ADR between 20 and 40% (or PDR between 30 and 70%)	**Overall BBPS:**<br><br>AI: mean 8.0 (SD 1.3)<br><br>No AI: mean 8.1 (SD 1.5)<br><br>97.4% (AI) and 93.9% (no AI) with total BBPS score ≥6 and score ≥2 in each segment
**MAGENTIQ-COLO™ (MAGENTIQ-EYE)**						
Maas 2024 – MAGENTIQ-COLO™[43] (parallel and tandem RCT; Germany, Israel, Netherlands, USA; N=916)[e]	AI: median 59.7 (IQR NR for combined groups) years	AI: 52.6% male<br><br>No AI: 55.0% male	• Non-iFOBT screening, 56.8% AI vs 55.5% no AI<br>• Surveillance, 43.2% AI vs 44.5% no AI	• History of CRC and adenomas unclear<br>• IBD or other bowel conditions unclear	• ADR between 25 and 40%	All had BBPS total score ≥6 and score ≥2 in each segment to be included in analysis

	No AI: median 60.1 (IQR NR for combined groups) years					

[a]Data for two CADe and two standard colonoscopy arms (with and without ENDOCUFF VISION™) combined for purpose of this review

[b]Reported for overall trial rather than specifically for CAD EYE® or GI Genius™ interventions

[c]Data for two CADe arms (with and without ENDOCUFF VISION™) combined for purpose of this review

[d]Data for two novice arms included in analysis only, as expert colonoscopy group not comparable

[e]Parallel and tandem groups for CADe and standard colonoscopy groups combined for most outcomes (based on which procedure performed first for tandem arms)

Abbreviations: ADR, adenoma detection rate; AI, artificial intelligence; BBPS, Boston Bowel Preparation Scale; BCSP, Bowel Cancer Screening Programme; CADe, computer-aided detection; CRC, colorectal cancer; CT, computed tomography; EMIS™, Endoscopic Multimedia Information System; FAP, familial adenomatous polyposis; FIT, faecal immunochemical test; FOBT, faecal occult blood test; GI, gastrointestinal; HNPCC, hereditary nonpolyposis colorectal cancer; IBD, inflammatory bowel disease; iFOBT, immunochemical faecal occult blood test; IQR, interquartile range; MLH1, mutL homolog 1; MSH2, mutS homolog 2; MSH6, mutS homolog 6; NHS, National Health Service; NR, not reported; PDR, polyp detection rate; RCT, randomised controlled trial; SD, standard deviation.

1.2.2 CADx

Supplementary Table 30. Summary of patient characteristics – included clinical studies - CADx

Study[a]	Age	Sex	Colonoscopy indication	History of bowel conditions	Endoscopist experience	Bowel preparation
**CAD EYE® (Fujifilm Healthcare UK Ltd.)**						

Cassinotti 2023[44] (prospective NRS – abstract only; Italy; N=113 lesions)	AI: mean 54.0 (SD NR) years<br><br>No AI: mean 54.0 (SD NR) years	Not reported	All endoscopic surveillance of ulcerative colitis	• History of CRC and adenomas unclear<br>• IBD included – all ulcerative colitis patients	Not reported	Not reported
Djinbachian 2024[45] (parallel RCT; Canada; N=179 lesions)	Mean 64.0 (SD 8.4) years	49.8% male	• Screening, 20.5%<br>• Surveillance, 55.0%<br>• Diagnostic, 18.3%	• History of CRC and adenomas unclear<br>• 26.6% with family history of CRC<br>• IBD and active colitis excluded	• Between 1 and >30 years' experience in optical diagnosis<br>• Procedural volume between 300 and 1500 colonoscopies per year	Trial record suggests those with inadequate bowel preparation (total BBPS score <6 or score <2 in right segment) excluded
Rondonotti 2023[46] (prospective NRS; Italy; N=550 lesions)	Mean 63.7 (SD 10.4) years	52.5% male	• Symptoms, 25.0%<br>• Surveillance, 36.1%<br>• FIT positive screening, 17.8%<br>• Primary screening, 21.1%	• History of CRC excluded<br>• History of adenomas unclear<br>• IBD or other bowel conditions unclear	• Experts and non-experts included<br>• Experts followed dedicated training programme, auditing and performed optical diagnosis regularly<br>• Formal 45 min training on optical diagnosis, BLI, BASIC system and AI features	Those with inadequate bowel preparation excluded (BBPS score <2 in any segment)

Sato 2024[47] (prospective NRS; Japan; N=380 lesions)	Median 70.0 (range 32 to 85) years	68.0% male	Includes positive faecal immunochemistry tests, symptoms such as abdominal pain or constipation, screening colonoscopy or where endoscopist otherwise deemed colonoscopy necessary – no breakdown provided	• History of CRC and adenomas unclear<br>• IBD excluded<br>• FAP excluded<br>• Prior colorectal resection other than appendectomy excluded	• Experts (≥1500 colonoscopies) and non-experts (<1500 colonoscopies) permitted<br>• 70% of colonoscopies performed by experts	Poor or inadequate bowel preparation (not defined) not included
Taghiakbari 2025[48] (prospective NRS; Canada; N=138 lesions)	Mean 67.2 (SD 8.8) years	50.5% male	Outpatient colonoscopy – no breakdown provided	• History of CRC and adenomas unclear<br>• IBD and other bowel conditions unclear	• Academic endoscopists with training and experience in CADx-assisted and CADx-unassisted OD performed all cases	Not reported
**Discovery™ (Pentax Medical UK)**						
Lopez-Serrano 2024[49] (prospective NRS; Spain; N=61 lesions)	Median 54.0 (IQR 48.0 to 63.0) years at index colonoscopy	57.7% male	All surveillance colonoscopy in ulcerative colitis patients at risk of CRC	• History of CRC excluded<br>• 27.0% with family history of CRC<br>• 23.1% with history of colorectal dysplasia	• Senior endoscopist with extensive experience in DCE and VCE<br>• Assisted by experienced nurse	Those with inadequate bowel preparation excluded (BBPS score <2 in any segment)

				• IBD included – all ulcerative colitis patients		
**GI Genius™ (Medtronic)**						
Bernhofer 2025[50] (prospective NRS; Austria; N=630 lesions)	Mean 63.8 (SD 12.7) years	48.9% male	• Screening, 38.2%<br>• Surveillance, 20.9%<br>• Polypectomy, 15.6%<br>• GI complaints, 14.7%<br>• Other, 10.7%	• History of CRC excluded<br>• History of adenomas unclear<br>• IBD excluded<br>• Other bowel conditions unclear	• Trainee endoscopists performed procedures (<500 colonoscopies) with experts (>2000 colonoscopies) able to support with polypectomies	Not reported
Hassan 2022[51] (prospective NRS; Italy: n=544 lesions)	Mean 66.6 (SD 10.2) years	46.3% male	• FIT positive, 16.6%<br>• Screening, 25.9%<br>• Post-polypectomy surveillance, 24.7%<br>• Diagnostic, 32.7%	• History of CRC excluded<br>• History of adenomas unclear<br>• IBD excluded	• Experienced endoscopists (>2000 screening colonoscopies)<br>• Trained in optical diagnosis and participation in previous polyp characterisation studies with BLI	Not reported
Rondonotti 2024[52] (prospective NRS; Italy; N=376 lesions)	Median 66.0 (IQR 57.0 to 73.0) years	55.0% male	• Symptoms, 32.5%<br>• Surveillance, 37.0%<br>• FIT positive, 21.0%<br>• Primary screening, 9.5%	• History of CRC excluded<br>• History of adenomas unclear<br>• IBD excluded	• Experts and non-experts in optical diagnosis<br>• Experts followed training, participated in prior studies of optical diagnosis, had auditing	Not reported

					and monitoring and performed optical diagnosis on a regular basis<br>• Median 20 years endoscopy practice<br>• All at least 300 colonoscopies	

[a]For most studies of CADx, both assessments were performed on the same patients meaning characteristics reported apply to both assessments.

Abbreviations: ADR, adenoma detection rate; AI, artificial intelligence; BBPS, Boston Bowel Preparation Scale; BLI, blue-light imaging; CADx, computer-aided characterisation; CRC, colorectal cancer; CSR, clinical study report; DCE, dye-based chromoendoscopy; FAP, familial adenomatous polyposis; FIT, faecal immunochemical test; GI, gastrointestinal; IBD, inflammatory bowel disease; IQR, interquartile range; OD, optical diagnosis; NRS, non-randomised study; RCT, randomised controlled trial; SD, standard deviation; VCE, virtual chromoendoscopy.

2 Excluded studies

2.1 Studies excluded from the National Institute for Health and Care Excellence (NICE) assessment

Supplementary Table 31. Table of studies excluded at the full-text appraisal stage of the database searches of the clinical systematic literature review.

Study/trial register number	Title	Reason for exclusion
Abdelrahim 2021[53]	Validation of a novel AI system (CADEYE) for in vivo characterization of colorectal polyps	Abstract only and have full texts covering outcomes
Abdelrahim 2024[54]	New AI model for neoplasia detection and characterisation in inflammatory bowel disease.	Technology not relevant to review
ACTRN12622000866707 2022[55]	Does Artificial Intelligence Improve Polyp Detection at Colonoscopy?	Trial record only
Adiwinata 2023[56]	The Impact of Artificial Intelligence in Improving Polyp and Adenoma Detection Rate During Colonoscopy: Systematic-Review and Meta-Analysis.	Systematic review used for reference checking
Agazzi 2021[57]	Real-time artificial intelligence-aided colonoscopy experience: The impact on routine clinical practice in a high-volume center-preliminary data	Non-randomised study and outcomes covered by randomised trials

Ahmad 2021[58]	Early evaluation of a computer assisted polyp detection system in bowel cancer screening	Non-randomised study and outcomes covered by randomised trials
Ahsan 2024[59]	IS THERE A BENEFIT FOR COMPUTER-AIDED DETECTION OF POLYPS DURING SCREENING COLONOSCOPY AMONG EXPERIENCED ENDOSCOPISTS IN A COMMUNITY HOSPITAL SETTING?	Non-randomised study and outcomes covered by randomised trials
Ahsan 2024[60]	The Impact of Computer-aided Detection Technology in Adenoma Detection Rate Among Experienced Endoscopists in the Community Setting.	Non-randomised study and outcomes covered by randomised trials
Alahmad 2024[61]	REAL WORLD EVIDENCE ON THE EFFICIENCY OF AI- ENHANCED COLONOSCOPY: A CASE-CONTROL STUDY	Non-randomised study and outcomes covered by randomised trials
Ali 2024[62]	ADHERENCE OF COMPUTER-AIDED POLYP DETECTION (CADE) CLINICAL TRIALS TO THE CONSOLIDATED STANDARDS OF REPORTING TRIALS-ARTIFICIAL INTELLIGENCE (CONSORT-AI) EXTENSION GUIDELINE - EVIDENCE REPORT FROM A SYSTEMATIC REVIEW AND META-ANALYSIS	Abstract of systematic review
Ali 2021[63]	Adenoma and polyp detection rates by implementing artificial intelligence systems: A metaanalysis	Abstract of systematic review
Aljabiri 2023[64]	THE USE OF ARTIFICAL INTELLIGENCE IN COLONOSCOPY IMPROVES ADENOMA DETECTION RATES AND INVERSELY	Unnamed intervention

	REDUCES THE RISK OF INTERVAL COLORECTAL CANCER; FIRST COMPARATIVE STUDY IN UAE	
Akram 2025[65]	EFFECTIVENESS OF THE COMBINATION OF ENDOCUFF-ASSISTED AND COMPUTER-AIDED COLONOSCOPY: A META-ANALYSIS OF RANDOMIZED CONTROLLED TRIALS	Abstract of systematic review
Alahmad 2023[66]	Impact of Artificial Intelligence Enhanced Colonoscopy in a Clinical Practice Setting	Unnamed intervention
Anand 2023[67]	Artificial Intelligence-Aided Colonoscopy in a Real World Setting	Non-randomised study and outcomes covered by randomised trials
Anderer 2024[68]	Meta-Analysis: AI-Assisted Colonoscopy Increases Detection of Polyps, Adenomas	Summary of article only
Aniwan 2022[69]	THE DIFFERENCES IN ADENOMA DETECTION RATES AND OTHER INDICES BETWEEN STANDARD SCREENING COLONOSCOPY VS. COMPUTER-AIDED DETECTION VS. MUCOSAL EXPOSURE DEVICE VS. THE COMBINATION: A RANDOMIZED TRIAL	Abstract of full publication identified
Anonymous 2024[70]	Artificial Intelligence-Assisted Colonoscopy for Detecting Polyps, Adenomas, Precancerous Lesions, and Colorectal Cancer: Health Technologies	Systematic review used for reference checking
Antonelli 2025[71]	ARTIFICIAL INTELLIGENCE FOR LEAVING IN SITU COLORECTAL POLYPS: RESULTS FROM A RANDOMISED TRIAL	Abstract of full publication identified

Areia 2022[72]	Cost-effectiveness of artificial intelligence for screening colonoscopy: a modelling study	Health economic assessment only
Areia 2022[73]	Cost-effectiveness of artificial intelligence for screening colonoscopy: a modelling study	Health economic assessment only
Armonis 2024[74]	COMPUTER-AIDED DETECTION IMPROVES ADENOMA DETECTION RATE FOR SCREENING COLONOSCOPY: A PROSPECTIVE TANDEM STUDY	Abstract only and have full texts covering outcomes
Ashat 2021[75]	Impact of real-time use of artificial intelligence in improving adenoma detection during colonoscopy: A systematic review and meta-analysis.	Systematic review used for reference checking
Aslam 2023[76]	The effectiveness of real-time computer-aided and quality control systems in colorectal adenoma and polyp detection during colonoscopies: a meta-analysis.	Systematic review used for reference checking
Aziz 2022[77]	COMPARISON OF ARTIFICIAL INTELLIGENCE WITH OTHER INTERVENTIONS TO IMPROVE ADENOMA DETECTION RATE FOR COLONOSCOPY: A NETWORK META-ANALYSIS OF RANDOMIZED CONTROLLED TRIALS	Abstract of systematic review
Aziz 2020[78]	The impact of deep convolutional neural network-based artificial intelligence on colonoscopy outcomes: A systematic review with meta-analysis.	Systematic review used for reference checking

Aziz 2024[79]	Comparison of Artificial Intelligence With Other Interventions to Improve Adenoma Detection Rate for Colonoscopy: A Network Meta-analysis.	Systematic review used for reference checking
Bai 2019[80]	Establishment and real-world validation of a computer-assisted polyp identification and localization system based on deep learning	Unnamed intervention
Bai 2025[81]	The role of artificial intelligence in colorectal cancer and polyp detection: A systematic review	Abstract of systematic review
Bang 2021[82]	Computer-Aided Diagnosis of Diminutive Colorectal Polyps in Endoscopic Images: Systematic Review and Meta-analysis of Diagnostic Test Accuracy.	Systematic review used for reference checking
Barkun 2022[83]	COST-EFFECTIVENESS ANALYSIS OF ARTIFICIAL INTELIGENCE-AIDED COLONOSCOPY FOR ADENOMA DETECTION IN COLORECTAL CANCER - A CANADIAN PERSPECTIVE	Health economic assessment only
Barkun 2023[84]	Cost-effectiveness of Artificial Intelligence-Aided Colonoscopy for Adenoma Detection in Colon Cancer Screening.	Health economic assessment only
Barua 2021[85]	Artificial intelligence for polyp detection during colonoscopy: a systematic review and meta-analysis.	Systematic review used for reference checking
Barua 2022[86]	ARTIFICIAL INTELLIGENCE FOR REAL-TIME OPTICAL DIAGNOSIS OF NEOPLASTIC POLYPS DURING COLONOSCOPY	Unnamed intervention

Barua 2022[87]	ARTIFICIAL INTELLIGENCE FOR REAL-TIME OPTICAL DIAGNOSIS OF NEOPLASTIC POLYPS DURING COLONOSCOPY	Unnamed intervention
Barua 2022[88]	Real-Time Artificial Intelligence-Based Optical Diagnosis of Neoplastic Polyps during Colonoscopy.	Technology not relevant to review
Behncke 2023[89]	Does Current Reimbursement Drive the Adoption of Computer-Aided Applications to Increase the Adenoma Detection in Colonoscopies - a Provider-Based Impact Model for Germany, France, and Italy	Health economic assessment only
Beran 2025[90]	ENDOCUFF WITH OR WITHOUT ARTIFICIAL INTELLIGENCE-ASSISTED COLONOSCOPY FOR DETECTION OF COLORECTAL ADENOMA: A SYSTEMATIC REVIEW AND META-ANALYSIS OF RANDOMIZED CONTROLLED TRIALS	Abstract of systematic review
Bergagnini 2023[91]	COMPUTER-AIDED DETECTION WITH ENDOCUFF IMPROVES ADENOMA DETECTION RATE: A QUALITY IMPROVEMENT INITIATIVE	Abstract only and have full texts covering outcomes
Bergna 2023[92]	ARTIFICIAL INTELLIGENCE SYSTEM USING WHITE LIGHT FOR REAL-TIME OPTICAL CHARACTERIZATION OF COLONIC POLYPS	Abstract only and have full texts covering outcomes
Bernhofer 2021[93]	The impact of artificial intelligence on the adenoma detection rate (ADR): A comparison between experienced and trainee endoscopists' adr	Non-randomised study and outcomes covered by randomised trials

Bernhofer 2024[94]	Augmented Colonoscopy with Computer- Aided polyp characterization - evaluation of the performance of an artificial intelligence application in the classification of colorectal polyps	Abstract only and have full texts covering outcomes
Bilal 2020[95]	Using Computer-Aided Polyp Detection During Colonoscopy.	Non-randomised study and outcomes covered by randomised trials
Bin Goh 2024[96]	SENIOR ENDOSCOPISTS ARE MORE LIKELY TO TRUST AND ACCEPT AI-ASSISTED COLONOSCOPY FOR DETECTION AND TREATMENT OF POLYPS COMPARED TO JUNIOR ENDOSCOPISTS	Unnamed intervention
Biscaglia 2022[97]	REAL-TIME ARTIFICIAL INTELLIGENCE-AIDED COLONOSCOPY ELIMINATES DIFFERENCES IN ADENOMA DETECTION RATE BETWEEN TRAINEES AND EXPERIENCED ENDOSCOPISTS IN TANDEM-COLONOSCOPIES	Unnamed intervention
Biscaglia 2022[98]	Real-time, computer-aided, detection-assisted colonoscopy eliminates differences in adenoma detection rate between trainee and experienced endoscopists.	Non-randomised study and outcomes covered by randomised trials
Brand 2020[99]	Influence of artificial intelligence on polyp detection in a real life scenario	Abstract only and have full texts covering outcomes
Brand 2021[100]	Artificial intelligence for polyp detection during colonoscopy-an in-depth analysis of a commercially available system	Non-randomised study and outcomes covered by randomised trials

Bretthauer 2025[101]	Use of computer-assisted detection (CADe) colonoscopy in colorectal cancer screening and surveillance: European Society of Gastrointestinal Endoscopy (ESGE) Position Statement	Systematic review used for reference checking
Budzyn 2024[102]	Endoscopist De-Skilling after Exposure to Artificial Intelligence in Colonoscopy: A Multicenter Observational Study	Non-randomised study and outcomes covered by randomised trials
Burke 2024[103]	Artificial intelligence-assisted adenoma detection in people with Lynch syndrome	Full text not retrieved
Burton 2024[104]	PATIENT PERSPECTIVE OF USE OF ARTIFICIAL INTELLIGENCE DURING COLONOSCOPY	Unnamed intervention
Bustamante-Balen 2025[105]	Cost-effectiveness analysis of artificial intelligence-aided colonoscopy for adenoma detection and characterization in Spain	Health economic assessment only
Bustamante-Balen 2024[106]	EE600 Cost-Effectiveness and Budget Impact Analysis of Introducing Artificial Intelligence-Aided Colonoscopy for Adenoma Detection and Characterization in Spain	Abstract only of health economic assessment
Caillo 2024[107]	COLODETECT 1: comparative evaluation of endocuff with computer-aided detection versus computer-aided detection alone versus standard colonoscopy for enhancing adenoma detection rates during screening colonoscopy-a pilot study	Non-randomised study and outcomes covered by randomised trials

Calce 2025[108]	ENDOSCOPISTS AND COMPUTER-AIDED DECISION SUPPORT INTERACTION FOR DETECTION OF COLORECTAL POLYPS	Unnamed intervention
Carlini 2025[109]	Large language models for detecting colorectal polyps in endoscopic images	Full text not retrieved
Chadha 2024[110]	Artificial Intelligence Improves Adenoma Detection Amongst Gastroenterology Fellows	Unnamed intervention
Chang 2024[111]	AI-ASSISTED COLONOSCOPY IMPROVES ADR IN GASTROENTEROLOGY FELLOWS: AN INTERIM ANALYSIS OF A RANDOMIZED CONTROL TRIAL	Abstract only and have full texts covering outcomes
Chaudhary 2024[112]	Novel Artificial Intelligence (AI) Systems in Detecting Adenomas in Colonoscopy: A Systematic Review and Network Meta-Analysis	Abstract of systematic review
Cheng 2025[113]	EVALUATION OF ARTIFICIAL INTELLIGENCE FOR ADENOMA DETECTION IN WATER EXCHANGE COLONOSCOPY: INTERIM ANALYSIS OF THE WEAID RANDOMIZED CONTROLLED TRIAL	Abstract only and have full texts covering outcomes
Cheng 2023[114]	Evaluation of a Computer-Aided Detection Device During Water Exchange Colonoscopy: A Pragmatic Implementation Performance Improvement Study	Non-randomised study and outcomes covered by randomised trials

Cheng 2024[115]	Evaluating the Performance of a Computer-Aided Diagnosis System in Implementing Diagnose-and-Leave and Resect-and-Discard Strategies for Diminutive Colorectal Polyps: A Real-World Pragmatic Study	Abstract only and have full texts covering outcomes
Cheng 2024[116]	PERFORMANCE OF REAL-TIME COMPUTER-AIDED POLYP DETECTION USING WATER EXCHANGE COLONOSCOPY: A PRELIMINARY PILOT STUDY	Abstract of full publication identified
Cheong 2024[117]	Colon AI-scopy: Artificial Intelligence Influence on Detection and Removal of Non-Neoplastic Polyps, Diminutive Hyperplastic Polyps, and Sessile Serrated Polyps in Screening Colonoscopy	Unnamed intervention
ChiCTR2400088486 2024[118]	The effect of artificial intelligence-assisted shortening of colonoscopy withdrawal time on adenoma detection	Trial record only
ChiCTR2400091641 2024[119]	Study on the detection value of colorectal adenomas by different artificial intelligence-assisted diagnostic devices	Trial record only
ChiCTR2500095651 2025[120]	Comparison between AI+Magic-Cap-assisted colonoscopy and traditional colonoscopy	Trial record only
ChiCTR1800017675 2018[121]	The impact of a computer aided diagnosis system based on deep learning on incresing polyp detection rate during colonoscopy, a prospective double blind study	Trial record only

ChiCTR1900021984 2019[122]	A multicenter randomized controlled study for evaluating the effectiveness of artificial intelligence in improving colonoscopy quality	Trial record only
ChiCTR1900023086 2019[123]	The impact of a colon polyp detection CAD system based on deep learning on colon adenoma miss rate: a randomized prospective tandem study	Trial record only
ChiCTR1900025235 2019[124]	A multicenter prospective randomized controlled trial for the impact of a computer-aided colon polyp detection system based on deep learning on colon adenoma detection during colonoscopy in comparison with junior endoscopist second observer	Trial record only
ChiCTR1900026726 2019[125]	The comparison of AI-assisted colonoscopy and conventional colonoscopy for the detection of polyps in colorectal cancer screening in White Light and LCI mode	Trial record only
ChiCTR1900027307 2019[126]	Artificial Intelligence-assisted Colonoscopy for Detection of Colon Polyps: a Prospective Randomized Cohort Study	Trial record only
ChiCTR2000034887 2020[127]	Difference Analysis of Colonoscopy Detection Rate of Adenoma Assisted by Artificial Intelligence	Trial record only
ChiCTR2000034889 2020[128]	Comparing adenoma detection rate of cap-assisted colonoscopy and conventional colonoscopy with and without artificial intelligence: a prospective, randomized, single-center trial	Trial record only

ChiCTR2100045262 2021[129]	Effects of phased application of artificial intelligence-assisted polyp diagnosis system on independent colonoscopy performance of endoscopists: a multicenter randomized controlled trial	Trial record only
ChiCTR2200063455 2022[130]	A prospective, multicenter, randomized control trial of a real-time quality-control system for the colonoscopy examination of outpatient	Trial record only
ChiCTR2200063891 2022[131]	Research on Artificial Intelligence Disease Identification System Based on Dynamic Big Data of Digestive Endoscope	Trial record only
ChiCTR2200064399 2022[132]	Effect of an Artificial Intelligence Computer-Aided Detection System on Adenoma Detection: a Multicenter Randomized Controlled Trial	Trial record only
ChiCTR2300067573 2023[133]	A multi-center study to observe the adenoma detection rate for different colonoscopy withdrawal time with AI-assisted detection system or not	Trial record only
ChiCTR2300071120 2023[134]	A Clinical Trial of the Effectiveness and Safety of Software Assisting Diagnose the Intestinal Polyp Digestive Endoscopy by Analysis of Colonoscopy Medical Images From Electronic Digestive Endoscopy Equipment	Trial record only
ChiCTR2300073421 2023[135]	A clinical trial to validate the effectiveness and safety of AI-assisted colonoscopy	Trial record only

ChiCTR2400082293 2024[136]	To verify the effect of artificial intelligence-assisted diagnosis combined with water exchange technology on the diagnostic efficiency of colonoscopy	Trial record only
ChiCTR2400082752 2024[137]	The effect of artificial intelligence on the adenoma detection rates and missed rates in water-assisted colonoscopy	Trial record only
Chieng 2022[138]	Effect of artificial intelligence on adenoma detection during colonoscopy: The first New Zealand experience	Non-randomised study and outcomes covered by randomised trials
Chikatimalla 2025[139]	REAL-TIME ARTIFICIAL INTELLIGENCE VS STANDARD COLONOSCOPY IN THE EARLY DETECTION OF COLORECTAL CANCER: A META-ANALYSIS	Abstract of systematic review
Chilakapati 2024[140]	Enhancing Colorectal Cancer Detection by Using Artificial Intelligence-Driven Colonoscopy to Detect Polyp and Adenoma Rates	Abstract of systematic review
Chin 2023[141]	One-year review of real-time artificial intelligence (AI)-aided endoscopy performance.	Non-randomised study and outcomes covered by randomised trials
Cho 2022[142]	THE PERFORMANCE OF CAD-EYETM FOR DIFFERENTIAL DIAGNOSIS OF COLORECTAL POLYPS	Abstract only and have full texts covering outcomes
Chow 2024[143]	LONG-TERM IMPACT OF ARTIFICIAL INTELLIGENCE ON COLORECTAL ADENOMA DETECTION IN A SAFETY-NET HOSPITAL: A ONE-YEAR FOLLOW-UP	Abstract of full publication identified

Chow 2024[144]	Long-term impact of artificial intelligence on colorectal adenoma detection in high-risk colonoscopy.	Non-randomised study and outcomes covered by randomised trials
Chow 2023[145]	Impact of Artificial Intelligence on Colorectal Adenoma Detection in High-Risk Colonoscopy: Initial Experience at a Safety-Net Hospital	Non-randomised study and outcomes covered by randomised trials
Chowdary 2023[146]	Application of machine learning approaches in the diagnosis and management of colorectal cancer	Abstract of systematic review
Chung 2024[147]	A prospective comparison of two computer aided detection systems with different false positive rates in colonoscopy	Technology not relevant to review
Claassen 2025[148]	IMPROVING ADENOMA AND POLYP DETECTION RATES WITH COMPUTER-AIDED DETECTION: LESSENING DISPARITIES IN COLORECTAL CANCER SCREENING IN RURAL AMERICA	Unnamed intervention
Contreras 2023[149]	The Impact of CADe on Sessile Serrated Adenomas Detection Rate During Colonoscopy	Unnamed intervention
Contreras 2023[150]	The Evaluation of Artificial Intelligence on Adenoma Detection Rate in a Latin American Community Setting	Unnamed intervention
Contreras 2025[151]	IMPACT OF CADE ON ADENOMA DETECTION RATES DURING LINKED COLOR IMAGING-ENHANCED COLONOSCOPIES IN A NON-ACADEMIC OUTPATIENT FACILITY IN THE DOMINICAN REPUBLIC	Unnamed intervention

Contreras 2022[152]	Adenoma Detection Rate Using LCI vs White Light Colonoscopy Both With and Without the Use of Artificial Intelligence: A Prospective Study in a Non-Academic Center in Latin America	Unnamed intervention
Cooper 2024[153]	Head-to-Head Comparison of Two Computer Aided Detection (CAD-e) Systems on Colonoscopy Performance Metrics	Non-randomised study and outcomes covered by randomised trials
Coron 2021[154]	Is artifical intelligence (CAD EYE) useful to not only detect but also to characterize small colorectal polyps? First results from a prospective french multicenter study	Abstract only and have full texts covering outcomes
Coronel 2022[155]	PHYSICIAN AND STAFF ATTITUDES TOWARDS IMPLEMENTATION OF ARTIFICIAL INTELLIGENCE-ASSISTED COLONOSCOPY	Limited usable data/no relevant outcomes
Correia 2021[156]	Artificial intelligence in the characterization of colorectal polyps: A prospective study in a clinical setting using cadeye	Abstract of full publication identified
de Castro 2023[157]	?EL EMPLEO DE UN DISPOSITIVO DE INTELIGENCIA ARTIFICIAL EN COLONOSCOPIAS DE CRIBADO DE CCR MEJORA LOS ESTANDARES DE CALIDAD DE LAS COLONOSCOPIAS? EXPERIENCIA EN UN HOSPITAL DE SEGUNDO NIVEL	Unnamed intervention
De Lange 2024[158]	Artificial intelligence for characterization of colorectal polyps: Prospective multicenter study.	Deprioritised as autonomous AI only

Deliwala 2020[159]	Artificial Intelligence (AI)-Guided vs Routine Colonoscopy for Colorectal Polyps: A Meta-Analysis of Recent Randomized Controlled Trials	Abstract of systematic review
Deliwala 2021[160]	Artificial intelligence (AI) real-time detection vs. routine colonoscopy for colorectal neoplasia: a meta-analysis and trial sequential analysis.	Systematic review used for reference checking
Desai 2024[161]	COMPUTER-AIDED POLYP DETECTION INCREASES ADENOMA DETECTION RATE IN A HIGH ADENOMA DETECTING GROUP: A MULTI-SITE COMMUNITY PRACTICE EXPERIENCE	Unnamed intervention
Djinbachian 2024[162]	COMPARING ENDOSCOPIST DIAGNOSIS OF COLORECTAL POLYPS ASSISTED BY ARTIFICIAL INTELLIGENCE (CADX) VS CADX WITHOUT ENDOSCOPIST INPUT: A RANDOMIZED CONTROLLED TRIAL	Unnamed intervention
Djinbachian 2024[163]	AUTONOMOUS ARTIFICIAL INTELLIGENCE VERSUS AI ASSISTED HUMAN OPTICAL DIAGNOSIS OF COLORECTAL POLYPS: A RANDOMIZED CONTROLLED TRIAL	Unnamed intervention
Djinbachian 2024[164]	OPTIMIZED COMPUTER ASSISTED TECHNIQUE FOR INCREASING ADENOMA DETECTION DURING COLONOSCOPY: A RANDOMIZED CONTROLLED TRIAL	Unnamed intervention
Djinbachian 2025[165]	Accuracy of histopathology evaluation in diminutive colonic polyps diagnosed as neoplastic by computer-aided characterisation	Full text not retrieved

Djinbachian 2024[166]	Autonomous Artificial Intelligence versus AI Assisted Human optical diagnosis of colorectal polyps: a randomized controlled trial	Unnamed intervention
Dominitz 2025[167]	DURABILITY OF THE IMPACT OF ARTIFICIAL INTELLIGENCE COMPUTER-AIDED DETECTION (CADE) ON THE ALL-INDICATION ADENOMA DETECTION RATE (ADR) AND OTHER COLONOSCOPY QUALITY INDICATORS: A RANDOMIZED PRAGMATIC STUDY	Abstract only and have full texts covering outcomes
Dominitz 2024[168]	IMPACT OF ARTIFICIAL INTELLIGENCE COMPUTER-AIDED DETECTION (CADE) ON THE ALL-INDICATION ADENOMA DETECTION RATE (ADR): PRAGMATIC QUALITY IMPROVEMENT (QI) STUDY IN A NATIONAL HEALTHCARE SYSTEM	Non-randomised study and outcomes covered by randomised trials
Doring 2021[169]	Artificial intelligence in endoscopic screening for colorectal cancer- expensive add-on or cost saving	Health economic assessment only
Dos Santos 2023[170]	Performance of artificial intelligence in the characterization of colorectal lesions.	Deprioritised as autonomous AI only
DRKS00023157 2020[171]	Real-time use of artificial intelligence (CADEYE) in the colo-rectal cancer surveillance of Lynch syndrome patients (CADLY)	Trial record only
DRKS00024943 2021[172]	Computer-aided detection of polyps during colonoscopy - a prospective, controlled study	Trial record only
DRKS00026687 2021[173]	Artificial Intelligence in screening colonoscopy	Trial record only

DRKS00030695 2023[174]	Real-time use of artificial intelligence (CAD EYE) in the colorectal cancer surveillance of Lynch syndrome patients (CADLYII) - an international multicenter trial	Trial record only
Eelbode 2020[175]	879 A PROSPECTIVE MULTI-CENTER VALIDATION STUDY FOR AUTOMATED POLYP DETECTION AS A SECOND OBSERVER	Unnamed intervention
El Zoghbi 2023[176]	Artificial Intelligence-Assisted Optical Diagnosis: A Comprehensive Review of Its Role in Leave-In-Situ and Resect-and-Discard Strategies in Colonoscopy.	Systematic review used for reference checking
Elhadi 2024[177]	Using Artificial Intelligence-Enhanced White-Light Colonoscopy for Predicting Deeply Invasive Colorectal Cancer: A Diagnostic Accuracy Meta-Analysis	Abstract of systematic review
Ellison 2023[178]	Effect of Artificial Intelligence Polyp Detection in an Office-Based Endoscopy Practice	Non-randomised study and outcomes covered by randomised trials
Ellrichmann 2023[179]	THE USE OF ARTIFICIAL INTELLIGENCE IMPROVES QUALITY CRITERIA IN SCREENING COLONOSCOPY	Abstract only and have full texts covering outcomes
England 2024[180]	Artificial Intelligence And Health Technology Assessment: Playing Catch-Up	Abstract of systematic review
Foroutan 2025[181]	Computer aided detection and diagnosis of polyps in adult patients undergoing colonoscopy: a living clinical practice guideline	Systematic review used for reference checking

Fitting 2021[182]	Development and comparison of the polyp detection system endomind with a commercially available cade system	Non-randomised study and outcomes covered by randomised trials
Gach 2023[183]	Artificial Intelligence During Colonoscopy: The First Thousand Procedures	Unnamed intervention
Gallagher 2024[184]	AI in Action: Augmenting Adenoma Detection Rates in a Community-Based Gastroenterology Practice	Non-randomised study and outcomes covered by randomised trials
Gangwani 2023[185]	Comparing Adenoma Detection Rate (ADR) in Single vs Dual Observer vs Artificial Intelligence-Assisted Colonoscopy: A Network Analysis of Randomized Controlled Trials	Abstract of systematic review
Gangwani 2024[186]	Single Versus Second Observer vs Artificial Intelligence to Increase the ADENOMA Detection Rate of Colonoscopy-A Network Analysis.	Systematic review used for reference checking
Gross 2011[187]	Computer-based classification of small colorectal polyps by using narrow-band imaging with optical magnification.	Technology not relevant to review
Gu 2024[188]	Disconnect Between Perceptions of Artificial Intelligence and Adenoma Detection Rate at a Tertiary Center: Survey and Retrospective	Non-randomised study and outcomes covered by randomised trials
Guerrero 2022[189]	Computer-Aided Detection (CADe) and Its Effect on Adenoma Detection Rate (ADR) in a Single Tertiary Center	Unnamed intervention

Guerrero 2020[190]	Artificial intelligence for polyp detection during colonoscopy: A systematic review and meta-analysis	Abstract of systematic review
Halvorsen 2025[191]	Benefits, burden, and harms of computer aided polyp detection with artificial intelligence in colorectal cancer screening: microsimulation modelling study.	Health economic assessment only
Halvorsen 2025[192]	Cost-Effectiveness for Artificial Intelligence in Colonoscopy.	Study design - review not systematic
Hann 2021[193]	Current status and limitations of artificial intelligence in colonoscopy.	Study design - review not systematic
Hardy 2025[194]	Explainable endoscopic artificial intelligence method for real-time in situ significant rectal lesion characterization: a prospective cohort study	Technology not relevant to review
Hassan 2024[195]	Computer-aided diagnosis for the resect-and-discard strategy for colorectal polyps: a systematic review and meta-analysis	Systematic review used for reference checking
Hassan 2022[196]	ARTIFICIAL INTELLIGENCE FOR LEAVING-IN-SITU COLORECTAL POLYPS: RESULTS OF A REAL TIME CLINICAL TRIAL	Unnamed intervention
Hassan 2022[197]	ARTIFICIAL INTELLIGENCE FOR LEAVING-IN-SITU COLORECTAL POLYPS: RESULTS OF A CLINICAL TRIAL	Unnamed intervention
Hassan 2022[198]	CHARACTERIZATION COMPARISON BETWEEN TWO CAD SYSTEMS (COMBO CAD STUDY) IN REAL-LIFE ENDOSCOPY: AN INTERIM ANALYSIS	Abstract of full publication identified

Hassan 2022[199]	CHARACTERIZATION COMPARISON BETWEEN TWO CAD SYSTEMS (COMBO CAD STUDY) IN REAL- LIFE ENDOSCOPY: AN INTERIM ANALYSIS	Abstract of full publication identified
Hassan 2020[200]	Computer-aided detection-assisted colonoscopy: classification and relevance of false positives.	Limited usable data/no relevant outcomes
Hassan 2021[201]	Artificial intelligence for non-polypoid colorectal neoplasms.	Study design - review not systematic
Hassan 2024[202]	Computer-Aided Diagnosis for Leaving Colorectal Polyps In Situ : A Systematic Review and Meta-analysis.	Systematic review used for reference checking
Hassan 2023[203]	Cost-utility analysis of real-time artificial intelligence-assisted colonoscopy in Italy.	Health economic assessment only
Hassan 2023[204]	Comparative Performance of Artificial Intelligence Optical Diagnosis Systems for Leaving in Situ Colorectal Polyps.	Deprioritised as autonomous AI only
Hassan 2021[205]	Performance of artificial intelligence in colonoscopy for adenoma and polyp detection: a systematic review and meta-analysis.	Systematic review used for reference checking
Hassan 2023[206]	Real-Time Computer-Aided Detection of Colorectal Neoplasia During Colonoscopy : A Systematic Review and Meta-analysis.	Systematic review used for reference checking
Hassan 2020[207]	New artificial intelligence system: first validation study versus experienced endoscopists for colorectal polyp detection.	*Ex vivo* application of technology

Haviland 2025[208]	A RETROSPECTIVE ANALYSIS OF ADENOMA DETECTION WITH GI GENIUS, ENDOCUFF, THEIR COMBINATION, AND STANDARD COLONOSCOPY	Non-randomised study and outcomes covered by randomised trials
Herman 2024[209]	Time and Experience Do Not Lead to Improved Adenoma Detection Rate With Artificial Intelligence-Assisted Colonoscopy: An 11-Month Implementation Trial	Unnamed intervention
Herman 2024[210]	Artificial Intelligence-Assisted Colonoscopy Is Associated With Higher Conversions From Screening to Therapeutic Exams	Unnamed intervention
Herman 2024[211]	Head-to-Head Real World Comparative Analysis of Two Artificial Intelligence-Assisted Colonoscopy Systems	Unnamed intervention
Herman 2024[212]	699 ARTIFICIAL INTELLIGENCE-ASSISTED COLONOSCOPY FAILED TO INCREASE ADENOMA DETECTION RATE: AN IMPLEMENTATION STUDY	Unnamed intervention
Hocke 2022[213]	DISCOVERY AI COMBINATION WITH G-EYE COLONOSCOPY SIGNIFICANTLY INCREASES ADENOMA DETECTION RATE - RESULTS OF A MULTICENTER STUDY	Abstract only and have full texts covering outcomes
Holanda 2025[214]	COMPUTER-AIDED DETECTION WITH OR WITHOUT MUCOSAL-EXPOSURE DEVICES IN COLONOSCOPY: A SYSTEMATIC REVIEW AND META-ANALYSIS WITH TRIAL SEQUENTIAL ANALYSIS	Abstract of systematic review

Horiuchi 2019[215]	REAL-TIME COMPUTER-AIDED DIAGNOSIS OF DIMINUTIVE COLORECTAL POLYPS USING AN AUTOFLUORESCENCE IMAGING SYSTEM	Unnamed intervention
Horiuchi 2019[216]	Real-time computer-aided diagnosis of diminutive rectosigmoid polyps using an auto-fluorescence imaging system and novel color intensity analysis software.	Technology not relevant to review
Hsu 2023[217]	IMPLEMENTATION OF THE NOVEL AI-BASED CECAL RECOGNITION SYSTEM IMPROVED THE ADENOMA DETECTION RATE IN SCREENING COLONOSCOPY	Technology not relevant to review
Htet 2023[218]	TIME TO IMPLEMENT RESECT & DISCARD SERVICE INTO PRACTICE: TWO NOVEL WAYS OF POLYP SIZING AND OPTICAL DIAGNOSIS WITH CADX	Technology not relevant to review
Htet 2024[219]	REAL-TIME COMPARATIVE STUDY OF CADX AND SIZING DEVICES FOR COLORECTAL POLYPS DURING COLONOSCOPY: A SOLUTION TO IMPLEMENT RESECT & DISCARD?	Abstract only and have full texts covering outcomes
Htet 2025[220]	CADE RESULTS FROM A LARGE INTERNATIONAL, MULTI-CENTRE, RANDOMISED-CONTROLLED TRIAL: MORE ADENOMAS DETECTED, NO INCREASE IN UNNECESSARY POLYPECTOMIES	Technology not relevant to review

Huang 2023[221]	Artificial Intelligence-Assisted Colonoscopy Improves Adenoma Detection Rate (ADR) in Both Low and High ADR Endoscopists: A Meta-Analysis	Abstract of systematic review
Huang 2022[222]	Effect of artificial intelligence-aided colonoscopy for adenoma and polyp detection: a meta-analysis of randomized clinical trials.	Systematic review used for reference checking
Huneburg 2022[223]	Real-time use of artificial intelligence in colorectal cancer surveillance of patients with Lynch syndrome - a randomized controlled trial	Abstract of full publication identified
Ishiyama 2022[224]	Impact of the clinical use of artificial intelligence-assisted neoplasia detection for colonoscopy: a large-scale prospective, propensity score-matched study (with video).	Technology not relevant to review
ISRCTN15467766 2024[225]	Future of real time endoscopy, artificial intelligence	Trial record only
ISRCTN68236490 2023[226]	ColoVision: using computers to instantly find and describe colorectal polyps	Trial record only
Jabbal 2023[227]	Utilization of Endocuff-Assisted Colonoscopy and Computer-Aided Detection in Optimizing Colonoscopies in the Elderly	Abstract only and have full texts covering outcomes
Jaber 2024[228]	COMPARISON OF COLONOSCOPY QUALITY METRICS USING ARTIFICIAL INTELLIGENCE ASSISTED COLONOSCOPY VERSUS STANDARD COLONOSCOPY: A SYSTEMATIC REVIEW AND META-ANALYSIS OF RANDOMIZED CONTROLLED TRIALS	Abstract of systematic review

James 2023[229]	REAL-WORLD VALIDATION OF A COMPUTER-AIDED DIAGNOSIS SYSTEM FOR CHARACTERIZATION OF POLYP HISTOLOGY IN COLONOSCOPY: A PROSPECTIVE MULTICENTER STUDY	Unnamed intervention
Jawwad 2025[230]	Novel Artificial Intelligence Systems in Detecting Adenomas in Colonoscopy: A Systemic Review and Network Meta-Analysis	Systematic review used for reference checking
Jimenez 2024[231]	ARTIFICIAL INTELLIGENCE ENHANCES ADENOMA DETECTION RATE IN LYNCH SYNDROME DURING COLONOSCOPY	Abstract only and have full texts covering outcomes
Jin 2024[232]	Effect of an artificial intelligence-assisted recognition system on colonoscopy quality	Full text not retrieved
Jin 2024[233]	Efficacy of artificial intelligence in reducing miss rates of GI adenomas, polyps, and sessile serrated lesions: a meta-analysis of randomized controlled trials	Systematic review used for reference checking
Jooton 2020[234]	PCN115 Economic Evaluation of Artificial Intelligence-Assisted Colonoscopy for Routine Screening of Low- to High-Risk Colorectal Cancer Patients in the United Kingdom	Health economic assessment only
Jooton 2020[235]	PCN99 Economic Evaluation of Artificial Intelligence-Assisted Colonoscopy for Routine Screening of High-Risk Colorectal Cancer Patients in Spain	Health economic assessment only

JPRN-jRCT1032230396 2023[236]	Clinical trial of colonoscopy using computer-aided detection systems in colorectal cancer screening	Trial record only
JPRN-UMIN000050685 2023[237]	The usefulness of computer-aided detection technologies based on artificial intelligence with image enhanced endoscopy for detecting colon adenoma; single center, randomized controlled trial	Trial record only
JPRN-UMIN000051437 2023[238]	A multicenter prospective randomized controlled trial evaluating the usefulness of a real-time colonoscopy diagnostic support system	Trial record only
JPRN-UMIN000053693 2024[239]	Influence of fatigue and stress on endoscopy accuracy and its relationship to AI colonoscopy: a multicenter clinical trial	Trial record only
JPRN-UMIN000053777 2024[240]	Randomised trial examining the sessile serrated lesion detection rate using linked color imaging in combination with an artificial intelligence assisted colonoscopy	Trial record only
jRCTs022190014 2019[241]	CAD for Polyp Detection Trial	Trial record only
jRCTs032190061 2019[242]	The validation study of detectability and diagnostic accuracy of AI-aided endoscopic diagnosis system for colonoscopy	Trial record only
Kader 2024[243]	RANDOMIZED CONTROLLED TRIAL OF A CLOUD-BASED ARTIFICIAL INTELLIGENCE (AI) COMPUTER-AIDED DIAGNOSIS (CADX) SYSTEM IN NON-EXPERT ENDOSCOPISTS	Abstract of full publication identified

Kader 2024[244]	RANDOMIZED CONTROLLED TRIAL OF A CLOUD-BASED ARTIFICIAL INTELLIGENCE POLYP DETECTION SYSTEM (CADDIE)	Abstract of full publication identified
Kader 2025[245]	CLOUD-BASED ARTIFICIAL INTELLIGENCE FOR DETECTION OF COLORECTAL NEOPLASIA - A RANDOMIZED CONTROLLED TRIAL (EAGLE TRIAL)	Abstract of full publication identified
Kader 2024[246]	RANDOMIZED CONTROLLED TRIAL (RCT) OF A CLOUDBASED ARTIFICIAL INTELLIGENCE (AI) COMPUTERAIDED DIAGNOSIS (CADX) SYSTEM INNON-EXPERT ENDOSCOPISTS	Abstract of full publication identified
Kader 2024[247]	Randomized controlled trial of a cloud-based artificial intelligence (AI) computer-aided diagnosis (CADx) system in non-expert endoscopists (CADDIE)	Abstract of full publication identified
Kader 2024[248]	Randomized controlled trial of a cloud-based artificial intelligence polyp detection system (CADDIE)	Abstract of full publication identified
Kader 2024[249]	RANDOMISED CONTROLLED TRIAL OF A CLOUD-BASED ARTIFICIAL INTELLIGENCE POLYP DETECTION SYSTEM (CADDIE)	Abstract of full publication identified
Kamba 2019[250]	The real-time detection and differential diagnosis of colorectal polyps in colonoscopy with an artificial intelligence algorithm; a prospective observational study	Unnamed intervention

Kamba 2021[251]	ID:3519580 A MULTICENTRE RANDOMIZED CONTROLLED TRIAL TO VERIFY THE REDUCIBILITY OF ADENOMA MISS RATE OF COLONOSCOPY ASSISTED WITH ARTIFICIAL INTELLIGENCE BASED SOFTWARE	Unnamed intervention
Kamba 2021[252]	Reducing adenoma miss rate of colonoscopy assisted by artificial intelligence: a multicenter randomized controlled trial.	Technology not relevant to review
Kandel 2024[253]	ARTIFICIAL INTELLIGENE AIDED COLONOSOCPY DOES NOT IMPROVE ENDOSCOPIST PERFORMACE IN COMMUNITY SETTINGS	Abstract only and have full texts covering outcomes
Kandel 2024[254]	Artificial Intelligence-Aided Colonoscopy Does Not Improve Endoscopist Performance in Community Settings	Non-randomised study and outcomes covered by randomised trials
Karthikeyan 2024[255]	COMPUTER-AIDED DETECTION OF POLYPS WITH ARTIFICIAL INTELLIGENCE DURING COLONOSCOPY IN THE HANDS OF AN EXPERIENCED COLORECTAL SURGEON	Non-randomised study and outcomes covered by randomised trials
Kawamoto 2022[256]	Systematic review of artificial intelligence-based image diagnosis for inflammatory bowel disease	Systematic review used for reference checking
Keshtkar 2023[257]	A Systematic Review and Meta-analysis of Convolutional Neural Network in the Diagnosis of Colorectal Polyps and Cancer.	Systematic review used for reference checking

Keswani 2023[258]	ADOPTION OF A COMPUTER-AIDED DETECTION SYSTEM SIGNIFICANTLY IMPROVES POLYP DETECTION IN ROUTINE CLINICAL PRACTICE	Abstract of full publication identified
Keswani 2024[259]	A Computer-Aided Detection (CADe) System Significantly Improves Polyp Detection in Routine Practice.	Non-randomised study and outcomes covered by randomised trials
Keswani 2024[260]	Adoption of a Computer-Aided Detection System May Improve Polyp Detection in Patients With Positive Stool-Based Testing	Non-randomised study and outcomes covered by randomised trials
Khatri 2023[261]	The Use of Artificial Intelligence to Improve Adenoma Detection Rate in a Community Practice	Non-randomised study and outcomes covered by randomised trials
Khouri 2024[262]	Effect of Computer-Aided Detection Device on the Adenoma Detection Rate and Serrated Detection Rate Among Trainee Fellows	Non-randomised study and outcomes covered by randomised trials
Kim 2025[263]	Role of Artificial Intelligence in Improving Quality of Colonoscopy	Systematic review used for reference checking
Kim 2025[264]	GI Genius increases small and right-sided adenoma and sessile serrated lesion detection rate when used with EndoCuff in a real-world setting: a retrospective United States study.	Non-randomised study and outcomes covered by randomised trials
Kim 2024[265]	The role of artificial intelligence in colonoscopy	Systematic review used for reference checking

Kim 2024[266]	EFFICACY OF COMPUTER-AIDED POLYP DETECTION WHEN USED ALONE AND IN CONJUNCTION WITH A MUCOSA-EXPOSURE DEVICE DURING COLONOSCOPY IN A REAL-WORLD SETTING	Non-randomised study and outcomes covered by randomised trials
Klare 2017[267]	Computer assisted detection of polyps during colonoscopy-results from an initial technical study	Unnamed intervention
Kliegis 2022[268]	Can a Polyp Detection and Characterization System Predict Complete Resection?	Limited usable data/no relevant outcomes
Kobayashi 2024[269]	Detailed Superiority of the CAD EYE Artificial Intelligence System over Endoscopists for Lesion Detection and Characterization Using Unique Movie Sets.	Endoscopists review of videos rather than live during colonoscopy
Kode 2024[270]	IMPACT OF ARTIFICIAL INTELLIGENCE ON ADENOMA DETECTION RATE OF GASTROENTEROLOGISTS AT A TERTIARY CARE ENDOSCOPY SUITE: A QUALITY IMPROVEMENT STUDY AND STATISTICAL ANALYSIS	Non-randomised study and outcomes covered by randomised trials
Koh 2023[271]	Real-time artificial intelligence (AI)-aided endoscopy improves adenoma detection rates even in experienced endoscopists: a cohort study in Singapore.	Non-randomised study and outcomes covered by randomised trials
Koleth 2022[272]	Artificial intelligence in gastroenterology: Where are we heading?.	Systematic review used for reference checking

Kominami 2016[273]	Computer-aided diagnosis of colorectal polyp histology by using a real-time image recognition system and narrow-band imaging magnifying colonoscopy.	Technology not relevant to review
Kudaravalli 2022[274]	Artificial Intelligence-Assisted Colon Polyp Detection: Initial Experience by Gastroenterology Fellows	Abstract only and have full texts covering outcomes
Kumar 2025[275]	Artificial intelligence breakthrough in diagnosis, treatment, and prevention of colorectal cancer - A comprehensive review	Systematic review used for reference checking
Kuo 2024[276]	META-ANALYSIS COMPARING ADVANCED ADENOMA DETECTION RATE OF WATER EXCHANGE AND COMPUTER-AIDED DETECTION COLONOSCOPY	Abstract of systematic review
Labaki 2024[277]	Artificial Intelligence in Gastrointestinal Endoscopy	Study design - review not systematic
Ladabaum 2023[278]	COMPUTER-AIDED DETECTION OF POLYPS DOES NOT IMPROVE COLONOSCOPIST PERFORMANCE IN A PRAGMATIC IMPLEMENTATION TRIAL	Non-randomised study and outcomes covered by randomised trials
Lam 2024[279]	LEVEL OF ACCEPTANCE AND TRUST OF ARTIFICIAL INTELLIGENCE AMONG GASTROENTEROLOGY NURSES	Technology not relevant to review
Lambin 2021[280]	Artificial intelligence for improving screening colonoscopies	Full text not retrieved

Lau 2023[281]	EFFECT of REAL-TIME COMPUTER-AIDED POLYP DETECTION SYSTEM (ENDOAID) on ADENOMA DETECTION in ENDOSCOPIST-IN-TRAINING: A SINGLE-BLIND RANDOMIZED CONTROLLED TRIAL (ENDOAID-TRAIN STUDY)	Abstract of full publication identified
Lau 2021[282]	New computer-aided polyp detection system (Endo-aid) increased the 5-10mm adenoma detection rate in junior endoscopists during colonoscopies-a pilot study	Non-randomised study and outcomes covered by randomised trials
Lee 2024[283]	EXPLORING ENDOSCOPIST PERCEPTIONS OF ARTIFICIAL INTELLIGENCE-AIDED COLONOSCOPY: A QUALITATIVE ANALYSIS	Limited usable data/no relevant outcomes
Lee 2023[284]	TANDEM STUDY DESIGN IS LESS LIKELY TO DEMONSTRATE IMPROVED ADENOMA DETECTION RATE THAN PARALLEL STUDY DESIGN IN THE ASSESSMENT OF ARTIFICIAL INTELLIGENCE-ASSISTED COLONOSCOPY	Abstract of systematic review
Lee 2024[285]	Impact of study design on adenoma detection in the evaluation of artificial intelligence-aided colonoscopy: a systematic review and meta-analysis.	Systematic review used for reference checking
Lee 2023[286]	Impact of Artificial Intelligence on Adenoma Detection Rate of Gastroenterologists at a Tertiary Care Endoscopy Suite: A Quality Improvement Study and Descriptive Analysis	Non-randomised study and outcomes covered by randomised trials

Lei 2020[287]	Adenoma detection rate is not influenced by the time of day in computer-aided detection colonoscopy.	Non-randomised study and outcomes covered by randomised trials
Levy 2022[288]	ARTIFICIAL INTELLIGENCE- AIDED COLONOSCOPY DOES NOT INCREASE ADENOMA DETECTION RATE IN ROUTINE CLINICAL PRACTICE	Abstract only and have full texts covering outcomes
Levy 2022[289]	Artificial Intelligence-Aided Colonoscopy Does Not Increase Adenoma Detection Rate in Routine Clinical Practice	Non-randomised study and outcomes covered by randomised trials
Li 2023[290]	Cost-effectiveness analysis of an artificial intelligence-assisted diagnosis and treatment system for gastrointestinal endoscopy	Health economic assessment only
Li 2022[291]	Real World Validation of an Artificial Intelligence Characterization Support System for Prediction of Polyp Histology in Colonoscopy: Interim Analysis of a Prospective Multicenter Study	Abstract only and have full texts covering outcomes
Li 2021[292]	Artificial intelligence can increase the detection rate of colorectal polyps and adenomas: a systematic review and meta-analysis.	Systematic review used for reference checking
Li 2022[293]	Performance and comparison of artificial intelligence and human experts in the detection and classification of colonic polyps.	Systematic review used for reference checking
Li 2025[294]	CAN ARTIFICIAL INTELLIGENCE ASSISTED COLONOSCOPY COMPENSATE FOR INADEQUATE BOWEL PREPARATION?	Non-randomised study and outcomes covered by randomised trials

Lin 2022[295]	COMPARISON OF COMPUTER-AIDED POLYP DETECTION (CADE) AND ENDOSCOPIC MECHANICAL ATTACHMENT ON ADENOMA DETECTION RATE: A SYSTEMATIC REVIEW AND META-ANALYSIS	Abstract of systematic review
Linlawan 2024[296]	USING COMPUTER-AIDED POLYP DETECTION SYSTEM(CADE) TO MAINTAIN THE HIGH QUALITY IN ADENOMA RATE DURING COMMUNITY-BASED COLORECTAL CANCER SCREENING IN THAILAND: A RANDOMIZED TRIAL	Unnamed intervention
Liu 2023[297]	ENDOANGEL versus water exchange for the detection of colorectal adenomas	Non-randomised study and outcomes covered by randomised trials
Liu 2020[298]	Study on detection rate of polyps and adenomas in artificial-intelligence-aided colonoscopy	Technology not relevant to review
Lou 2023[299]	Artificial intelligence for colorectal neoplasia detection during colonoscopy: a systematic review and meta-analysis of randomized clinical trials.	Systematic review used for reference checking
Lu 2022[300]	LONG-TERM EFFECTIVENESS OF ARTIFICIAL INTELLIGENCE SYSTEM IN IMPROVING ADENOMA DETECTION RATE: A MULTICENTER SELF-CONTROLLED STUDY	Unnamed intervention
Lu 2022[301]	ARTIFICIAL INTELLIGENCE ASSISTANCE IMPROVES AS ENDOSCOPIST FATIGUE INCREASES: SECONDARY ANALYSIS OF TWO RANDOMIZED TRIALS	Limited usable data/no relevant outcomes

Lu 2023[302]	Assessment of the Role of Artificial Intelligence in the Association Between Time of Day and Colonoscopy Quality.	Limited usable data/no relevant outcomes
Lui 2020[303]	ACCURACY OF ARTIFICIAL INTELLIGENCE ON HISTOLOGICAL PREDICTION AND DETECTION OF COLORECTAL POLYPS: A SYSTEMATIC REVIEW AND METAANALYSIS	Abstract of systematic review
Lui 2024[304]	1246 FINDINGS AT SURVEILLANCE COLONOSCOPY IN HIGH-RISK PATIENTS WITH PRIOR COMPUTED-ASSISTED DETECTION DURING INDEX COLONOSCOPY: A PROPENSITY SCORE MATCHING ANALYSIS	Unnamed intervention
Lui 2022[305]	POTENTIAL IMPACTS OF COMPUTER-AIDED DETECTION OF COLORECTAL POLYPS ON COLONOSCOPY SURVEILLANCE INTERVALS	Non-randomised study and outcomes covered by randomised trials
Lui 2022[306]	COMPUTER-ASSISTED DETECTION VERSUS CONVENTIONAL COLONOSCOPY ON DETECTION AND MISS RATES OF PROXIMAL COLONIC LESIONS: A MULTI-CENTRE, RANDOMIZED, TANDEM COLONOSCOPY STUDY	Unnamed intervention
Lui 2023[307]	The Impacts of Computer-Aided Detection of Colorectal Polyps on Subsequent Colonoscopy Surveillance Intervals: Simulation Study.	Non-randomised study and outcomes covered by randomised trials
Lui 2020[303]	Accuracy of artificial intelligence on histology prediction and detection of colorectal polyps: a systematic review and meta-analysis.	Systematic review used for reference checking

Lui 2023[308]	Computer-assisted detection versus conventional colonoscopy for proximal colonic lesions: a multicenter, randomized, tandem-colonoscopy study.	Technology not relevant to review
Lui 2020[309]	ARTIFICIAL INTELLIGENCE-ASSISTED REAL-TIME DETECTION REDUCES MISSED LESIONS DURING COLONOSCOPY: A RETROSPECTIVE AND PROSPECTIVE STUDY	Unnamed intervention
Lui 2024[310]	ARTIFICIAL INTELLIGENCE ASSISTED MONITORING OF EFFECTIVE WITHDRAWAL TIME AND ADENOMA DETECTION RATE OF INDIVIDUAL ENDOSCOPIST	Non-randomised study and outcomes covered by randomised trials
Lui 2023[311]	COMPUTER-ASSISTED DETECTION WITH OR WITHOUR ENDOCUFF ON DETECTION OF COLORECTAL ADENOMA: A RANDOMIZED CONTROLLED TRIAL (INTERIM ANALYSIS)	Abstract only and have full texts covering outcomes
Lui 2025[312]	PROSPECTIVE EVALUATION OF ARTIFICIAL INTELLIGENCE-ASSISTED MONITORING OF EFFECTIVE WITHDRAWAL TIME VERSUS STANDARD WITHDRAWAL TIME ON ADENOMA PER COLONOSCOPY	Study design - not assessing impact of intervention on outcomes
Lui 2025[313]	Surveillance findings in high-risk patients after baseline computer-assisted detection colonoscopy: a propensity score matching analysis	Non-randomised study and outcomes covered by randomised trials
Luo 2021[314]	Artificial Intelligence-Assisted Colonoscopy for Detection of Colon Polyps: a Prospective, Randomized Cohort Study.	Technology not relevant to review

Lwin 2025[315]	Clinical significance of computer-aided quality assessment systems in colonoscopy: a comprehensive review.	Systematic review used for reference checking
Ma 2024[316]	Efficacy and safety of computer-aided detection(CADe) in colonoscopy for colorectal neoplasia detection: A meta-analysis	Systematic review used for reference checking
Maan 2025[317]	Artificial Intelligence in Endoscopy Quality Measures	Study design - review not systematic
Maas 2023[318]	A NOVEL COMPUTER-AIDED POLYP DETECTION SYSTEM in DAILY CLINICAL CARE: AN INTERNATIONAL MULTICENTER, RANDOMIZED, TANDEM TRIAL	Abstract of full publication identified
Maida 2023[319]	EFFECTIVENESS OF ARTIFICIAL INTELLIGENCE FOR COLONOSCOPY ON ADENOMA AND POLYP MISS RATE: A METAANALYSIS OF TANDEM RCTS	Abstract of systematic review
Maida 2025[320]	Effectiveness of artificial intelligence assisted colonoscopy on adenoma and polyp miss rate: A meta-analysis of tandem RCTs	Systematic review used for reference checking
Makar 2024[321]	Use of Artificial Intelligence Improves Colonoscopy Performance in Adenoma Detection: A Systematic Review and Meta-Analysis.	Systematic review used for reference checking
Malik 2024[322]	Is Adenoma Detection Any Different From Polyp Detection With Artificial Intelligence in Colonoscopy? A Meta-Analysis of Randomized Controlled Data	Abstract of systematic review

Mangas-Sanjuan 2023[323]	ROLE OF ARTIFICIAL INTELLIGENCE IN COLONOSCOPY DETECTION OF ADVANCED LESIONS: A RANDOMIZED TRIAL	Unnamed intervention
Mangas-Sanjuan 2023[324]	ROLE of ARTIFICIAL INTELLIGENCE in COLONOSCOPY DETECTION of ADVANCED LESIONS	Abstract only and have full texts covering outcomes
Mansour 2023[325]	Artificial Intelligence in Colonoscopy	Systematic review used for reference checking
McGrath 2025[326]	DOES AI INFLUENCE ADENOMA DETECTION RATES IN FIT-POSITIVE PATIENTS	Unnamed intervention
Mehta 2023[327]	Effectiveness of artificial intelligence-assisted colonoscopy in early diagnosis of colorectal cancer: a systematic review	Systematic review used for reference checking
Mekritthikra 2022[328]	THE PERFORMANCE OF REAL-TIME COMPUTER-AIDED CHARACTERIZATION OF COLORECTAL NEOPLASIA IN SCREENING COLONOSCOPY: A PROSPECTIVE STUDY	Abstract only and have full texts covering outcomes
Melquist 2022[329]	CAUSES OF FALSE POSITIVE DETECTIONS BY CADE DURING REAL-TIME COLONOSCOPY AND ITS CLINICAL IMPACT: A SYSTEMATIC REVIEW AND META-ANALYSIS	Abstract of systematic review
Milluzzo 2021[330]	ID: 3522041 INCREMENTAL YIELD OF ARTIFICIAL INTELLIGENCE IN FOLLOW-UP SCREENING COLONOSCOPIES - AN INTERIM ANALYSIS	Abstract only and have full texts covering outcomes

Milluzzo 2021[331]	Incremental Yield Of Artificial Intelligence In Followup Screening Colonoscopies: An Interim Analysis	Abstract only and have full texts covering outcomes
Milluzzo 2021[332]	Incremental yield of artificial intelligence in follow-up screening colonoscopies-an interim analysis on 432 patients	Abstract only and have full texts covering outcomes
Milluzzo 2021[333]	Incremental yield of artificial intelligence in follow-up screening colonoscopies-an interim analysis	Abstract only and have full texts covering outcomes
Minegishi 2022[334]	Comprehensive Diagnostic Performance of Real-Time Characterization of Colorectal Lesions Using an Artificial Intelligence-Assisted System: A Prospective Study.	Technology not relevant to review
Ming Yen Koh 2024[335]	REAL-WORLD ASSESSMENT OF THE EFFICACY OF COMPUTER-ASSISTED DIAGNOSIS (CADX) IN COLONOSCOPY - A COHORT STUDY IN SINGAPORE	Abstract only and have full texts covering outcomes
Misawa 2022[336]	A PROSPECTIVE STUDY OF REAL-TIME USE OF COMPUTER-AIDED CHARACTERIZATION FOR COLORECTAL LESIONS	Unnamed intervention
Misawa 2022[337]	A PROSPECTIVE STUDY OF REAL-TIME COMPUTER-AIDED CHARACTERIZATION OF COLORECTAL LESIONS: DIAGNOSTIC PERFORMANCE AND IMPACT ON HUMAN DIAGNOSIS	Technology not relevant to review

Miyaguchi 2024[338]	Artificial intelligence-assisted linked color imaging versus linked color imaging for colorectal adenoma detection: the first randomized controlled trial	Trial record only
Mizukami 2023[339]	Usefulness of AI-Equipped Endoscopy for Detecting Colorectal Adenoma during Colonoscopy Screening: Confirm That Colon Neoplasm Finely Can Be Identified by AI without Overlooking Study (Confidential Study).	Non-randomised study and outcomes covered by randomised trials
Mohan 2020[340]	Real-time computer aided colonoscopy versus standard colonoscopy for improving adenoma detection rate: A meta-analysis of randomized-controlled trials	Systematic review used for reference checking
Mohan 2020[341]	Accuracy of convolutional neural network-based artificial intelligence in diagnosis of gastrointestinal lesions based on endoscopic images: A systematic review and meta-analysis.	Systematic review used for reference checking
Moosvi 2020[342]	Computer-Aided Polyp Detection during Colonoscopy: A Systematic Review and Meta-Analysis	Abstract of systematic review
Mori 2022[343]	COST-EFFECTIVENESS OF ARTIFICIAL INTELLIGENCE FOR SCREENING COLONOSCOPY	Health economic assessment only
Mori 2020[344]	Cost savings in colonoscopy with artificial intelligence-aided polyp diagnosis: An add-on analysis of a clinical trial	Technology not relevant to review

Mori 2019[345]	482 PERFORMANCE OF NON-EXPERT ENDOSCOPISTS IN OPTICAL BIOPSY OF DIMINUTIVE COLORECTAL POLYPS WITH REAL-TIME USE OF ARTIFICIAL INTELLIGENCE	Unnamed intervention
Mori 2018[346]	Optical biopsy of diminutive colorectal polyps with real-time use of "artificial intelligence"-assisted endoscopy	Unnamed intervention
Mori 2017[347]	Diagnostic yield of "artificial intelligence"-assisted endocytoscopy for colorectal polyps: A prospective study	Unnamed intervention
Mori 2021[348]	Artificial intelligence-assisted colonoscopy for cancer recognition: A multicenter study designed to obtain regulatory approval	Unnamed intervention
Mori 2020[349]	Cost savings in colonoscopy with artificial intelligence-aided polyp diagnosis: an add-on analysis of a clinical trial (with video).	Technology not relevant to review
Mori 2018[350]	Real-Time Use of Artificial Intelligence in Identification of Diminutive Polyps During Colonoscopy: A Prospective Study.	Technology not relevant to review
Mori 2023[351]	Impact of Artificial Intelligence on Colonoscopy Surveillance After Polyp Removal: A Pooled Analysis of Randomized Trials.	Systematic review used for reference checking
Morimoto 2025[352]	DEVELOPMENT OF COMPUTER-AIDED DIAGNOSTIC SYSTEMS FOCUSED ON THE JNET CLASSIFICATIONS FOR COLORECTAL LESIONS	Unnamed intervention

Morimoto 2025[353]	Efficiency of Real-time Computer-aided Polyp Detection during Surveillance Colonoscopy: A Pilot Study.	Technology not relevant to review
Mwango 2024[354]	Effect of artificial intelligence-aided colonoscopy on the adenoma detection rate: A systematic review	Systematic review used for reference checking
Narimiti 2022[355]	AI Assisted Colonoscopy Does Not Affect Mental Workload in Gastroenterologists	Abstract only and have full texts covering outcomes
Nayar 2024[356]	The Impact of Real Time Artificial Intelligence Enhanced Colonoscopy: A One Year Performance Review	Unnamed intervention
Nazarian 2021[357]	Diagnostic Accuracy of Artificial Intelligence and Computer-Aided Diagnosis for the Detection and Characterization of Colorectal Polyps: Systematic Review and Meta-analysis.	Systematic review used for reference checking
NCT03622281 2018[358]	Quality Improvement Intervention in Colonoscopy Using Artificial Intelligence	Trial record only
NCT03925337 2019[359]	Computer Aided Detection of Polyps in the Colon	Trial record only
NCT03967756 2019[360]	Impact of Automatic Polyp Detection System on Adenoma Detection Rate	Trial record only
NCT04071678 2019[361]	A Randomized Controlled Multicenter Study of Artificial Intelligence Assisted Digestive Endoscopy	Trial record only

NCT04074577 2019[362]	Computer Aided Detection, Tandem Colonoscopy Study	Trial record only
NCT04102631 2019[363]	A Multicenter Study Evaluating the Effectiveness of Endo.Angel in Improving the Quality of Colonoscopy	Trial record only
NCT04126265 2019[364]	Artificial Intelligence-assisted Colonoscopy for Detection of Colon Polyps	Trial record only
NCT04294355 2020[365]	Artificial Intelligence-assisted Colonoscopy on Detection of Missed Proximal Lesions	Trial record only
NCT04325815 2020[366]	CADDIE Trial - Computer Aided Diagnosis and Detection for Intelligent Endoscopy	Trial record only
NCT04422548 2020[367]	Does AI-assisted Colonoscopy Improve Adenoma Detection in Screening Colonoscopy?	Trial record only
NCT04440865 2020[368]	Impact of Artificial Intelligence Genius® System-assisted Colonoscopy vs. Standard Colonoscopy (COLO-GENIUS)	Trial record only
NCT04441580 2020[369]	Assessing the Additional Neoplasia Yield of Computer-aided Colonoscopy in a Screening Setting	Trial record only
NCT04453956 2020[370]	A Single Center Study on Comparing the Different Function of EndoAngel in Improving the Quality of Colonscopy	Trial record only

NCT04485715 2020[371]	Application of AI in Polypectomy	Trial record only
NCT04640792 2020[372]	A Study to Evaluate the Safety and Efficacy of the Use of ME-APDS During Colonoscopy	Trial record only
NCT04673136 2020[373]	Usefulness of GI-GENIUS in FIT-based Colorectal Cancer Screening Program	Trial record only
NCT04691401 2020[374]	Impact of Artificial Intelligence (AI) on Adenoma Detection During Colonoscopy in FIT+ Patients	Trial record only
NCT04723758 2021[375]	COLO-DETECT: can an Artificial Intelligence Device Increase Detection of Polyps During Colonoscopy?	Trial record only
NCT04727814 2021[376]	Comparison of Polyp Detection and False Alarm Rates in Water Exchange and Air Insufflation Colonoscopy	Trial record only
NCT04837599 2021[377]	Artificial Intelligence Performance in Colonoscopy in Daily Practice	Trial record only
NCT04838951 2021[378]	Effect of Real-time Computer-aided System (ENDO-AID) on Adenoma Detection in Endoscopist-in-training	Trial record only
NCT04894708 2020[379]	Study on the Use of Artificial Intelligence (Fujifilm) for Polyp Detection in Colonoscopy	Trial record only

NCT04909671 2021[380]	Evaluation of ArTificial Intelligence System (Gi-Genius) for adenoMa dEtection in Lynch Syndrome	Trial record only
NCT04912037 2021[381]	A Study on the Effectiveness of AI-assisted Colonoscopy in Improving the Effect of Colonoscopy Training for Trainees	Trial record only
NCT04945044 2021[382]	Artificial Intelligence Aid Systems in Colorectal Adenoma Detection	Trial record only
NCT05013125 2021[383]	ENDO-AID Assisted Tandem Colonoscopy RCT	Trial record only
NCT05064124 2021[384]	Early diAgnosis Real-Time Healthcare System for CANcer Trial	Trial record only
NCT05133544 2021[385]	Endocuff With or Without AI-assisted Colonoscopy	Trial record only
NCT05139186 2021[386]	The EYE Study Enhancing the Diagnostic Yield of Standard Colonoscopy by Artificial Intelligence Aided Endoscopy	Trial record only
NCT05141773 2021[387]	Artificial Intelligence Aid Systems and Endocuff in Colorectal Adenoma Detection	Trial record only
NCT05158725 2021[388]	Comparison of Colonoscopy Adenoma Detection Yield	Trial record only
NCT05178095 2021[389]	Artificial Intelligence in Colonic Polyp Detection	Trial record only

NCT05237310 2022[390]	Comparing Detection of Standard Colonoscopy, CAD-EYE and Combined CAD-EYE and G-EYEÂ® Aided Colonoscopy	Trial record only
NCT05240625 2022[391]	Clinical Efficacy Evaluation of a Computer-aided Colonoscopy as Compared With the Standard Colonoscopy	Trial record only
NCT05275556 2022[392]	Gastroenterology Artificial INtelligence System for Detecting Colorectal Polyps (The GAIN Study)	Trial record only
NCT05317351 2022[393]	A Prospective Randomized Study Comparing the Adenoma Detection Yield of SC, AI and Combined AI and G-EYEÂ®	Trial record only
NCT05318495 2022[394]	A Dual Tandem Study - SC vs. CAD-EYE vs. CAD-EYE With G-EYE	Trial record only
NCT05323279 2022[395]	Evaluate the Effects of An AI System on Colonoscopy Quality of Novice Endoscopists	Trial record only
NCT05391477 2022[396]	Artificial Intelligence for Diminutive Polyp Characterization	Trial record only
NCT05414448 2022[397]	Combination of Artificial Intelligence and Mucosal Exposure Device to Enhance Colorectal Neoplasia Detection	Trial record only
NCT05423964 2022[398]	Impact of AI on Trainee ADR	Trial record only
NCT05448300 2022[399]	Adenoma Detection Rate in Water and Air Colonoscopy Using Computer-aided System	Trial record only

NCT05481632 2022[400]	Validating the Safety and Effectiveness of ENDOANGEL Lower Gastrointestinal Endoscope Image Auxiliary Diagnostic Software	Trial record only
NCT05500248 2022[401]	Artificial Intelligence for Leaving in Situ Colorectal Polyps	Trial record only
NCT05523271 2022[402]	A Multi Center Study Comparing the Efficacy of CAD EYE and the Standard of Care (White Light)	Trial record only
NCT05568992 2022[403]	Prospective, Randomized Controlled Study to Evaluate the Effect of Artificial Intelligence Assisted Optical Diagnosis of Advanced Adenomas	Trial record only
NCT05594576 2022[404]	Comparison of the ENDOCUFF VISION® Endoscopy Cap Coupled With GI GENIUS Artificial Intelligence Compared to Each Device Alone in Improving Colonic Adenoma Detection Rate During Colonoscopy	Trial record only
NCT05611151 2022[405]	Real-time Computer-Aided Detection of Colonic Adenomas With NEC WISE VISION Endoscopy	Trial record only
NCT05718193 2023[406]	Real-Time Artificial Intelligence Assissted Colonoscopy to Identify and Classify Polyps	Trial record only
NCT05730192 2023[407]	EAGLE Trial CADDIE Artificial Intelligence Endoscopy	Trial record only
NCT05740137 2023[408]	Adenoma Detection Rate in Artificial Intelligence-assisted Colonoscopy	Trial record only
NCT05829590 2023[409]	AI-assisted Colonoscopy Report System In Improving Reporting Quality	Trial record only

NCT05862948 2023[410]	Accuracy of Endo-aid in Consecutive Patients Referred for Colonoscopy	Trial record only
NCT05870332 2023[411]	Nationwide Study of Artificial Intelligence in Adenoma Detection for Colonoscopy	Trial record only
NCT05941689 2023[412]	Efficacy of Artificial Intelligence-assisted Colonic Polyp Detection System	Trial record only
NCT05943288 2023[413]	Safety and Efficacy of the Olympus CADe System in Real-time Colonoscopy	Trial record only
NCT05963191 2023[414]	CAD-EYE System for the Detection of Neoplastic Lesions in Patients With Lynch Syndrome	Trial record only
NCT05963724 2023[415]	Real-Time CAD for Colonic Neoplasia: a RCT	Trial record only
NCT05990218 2023[416]	Artificial Intelligence in the Detection of Right Sided Colonic Polyp in Different Operator Experience	Trial record only
NCT06041945 2023[417]	Artificial Intelligence to Implement Cost-saving Strategies for Colonoscopy Screening Based on in Vivo Prediction of Polyp Histology	Trial record only
NCT06062095 2023[418]	Computer Aided Diagnosis (CADx) for Colorectal Polyps Resect-and-Discard Strategy	Trial record only
NCT06077435 2023[419]	Comparing CADe Software for Enhanced Polyp Detection	Trial record only

NCT06160466 2023[420]	Assessing the Additional Neoplasia Yield of Computer-aided Colonoscopy in Follow-up Patients in a Screening Setting	Trial record only
NCT06173258 2023[421]	Water Exchange Colonoscopy With Artificial Intelligence-assisted Detection	Trial record only
NCT06216405 2024[422]	Performance of Artificial Intelligence in Colonoscopy for Right Colon Polyp Detection	Trial record only
NCT06251700 2024[423]	Longterm Effectiveness of Artificial Intelligence-assisted Colonoscopy on Adenoma Recurrence	Trial record only
NCT06281392 2023[424]	Artificial Intelligence and Dysplasia Detection in Inflammatory Bowel Disease (EIIDISIA Study)	Trial record only
NCT06469671 2024[425]	Effectiveness of Artificial Intelligence - Assisted Colonoscopy in Colorectal Neoplasms	Trial record only
NCT06543862 2024[426]	Autonomous Artificial Intelligence Versus AI Assisted Human Optical Diagnosis	Trial record only
NCT06617468 2024[427]	Effect of the Computer Aided Diagnosis with Explainable Artificial Intelligence for Colon Polyp on Optical Diagnosis and Acceptance of Technology	Trial record only
NCT06621225 2024[428]	Artificial Intelligence in Colonoscopy	Trial record only

NCT06654128 2024[429]	The Yield of Artificial Intelligence (GI Genius) in Lynch Syndrome - a Randomized Tandem-colonoscopy Trial	Trial record only
NCT06676930 2024[430]	Impact of Artificial Intelligence on Trainee Polyp Miss Rates	Trial record only
NCT06715384 2024[431]	Evaluation of a CAM System for Colorectal Polyp Size Measurement	Trial record only
NCT06786793 2025[432]	Artificial Intelligence in Colonoscopy	Trial record only
NCT06792292 2025[433]	Artificial Intelligence-Assisted Colonoscopy in Colorectal Cancer Screening in a General Hospital	Trial record only
NCT06799793 2024[434]	Artificial Intelligence-based Screening Models for Prevention and Early Detection of Colorectal Cancer	Trial record only
NCT06786793 2025[435]	Artificial Intelligence in Endoscopic Diagnosis of Colorectal Polyps: A Prospective Randomized Study	Trial record only
NCT06773832 2025[436]	Artificial Intelligence Predicts the Pathology and Endoscopic Classification of Colorectal Polyps During Colonoscopy	Trial record only
NCT06676930 2024[437]	Impact of Computer Aided Detection on Trainee Polyp Miss Rates Using a Tandem Colonoscopy Design	Trial record only

NCT06345105 2024[438]	A Prospective Evaluation of the Correlation Between Real Time Artificial Intelligence-derived Effective Withdrawal Time and Adenoma Detection Rate	Trial record only
NCT06469671 2024[439]	Artificial Intelligence - Assisted Colonoscopy in Diagnosis of Colorectal Neoplasms: Russian Multicenter Randomised Open - Label Trial	Trial record only
NCT06335654 2024[440]	Real-time Artificial Intelligence-based Endocytoscopic Diagnosis of Colorectal Neoplasms: a Single Center, Prospective Clinical Study	Trial record only
NCT06173258 2023[441]	Evaluation of Artificial Intelligence for Adenoma Detection in Water Exchange Colonoscopy: the WEAID Randomized Controlled Trial (Water Exchange With Artificial Intelligence-assisted Detection)	Trial record only
NCT05963191 2023[442]	Evaluation of the CAD-EYE System for the Detection of Colorectal Neoplastic Lesions in Patients With Lynch Syndrome	Trial record only
NCT05941689 2023[443]	Research on the Auxiliary Diagnosis and Treatment System of Digestive Endoscopy Based on Artificial Intelligence: An Efficacy Study of Artificial Intelligence-assisted Colonic Polyp Detection System	Trial record only
NCT06041945 2023[444]	Saving by Artificial Intelligence for Virtual Endoscopy Biopsy Artificial Intelligence to Implement Cost-saving Strategies for Colonoscopy Screening Based on in Vivo Prediction of Polyp Histology	Trial record only

NCT06077435 2023[445]	Comparing CADe Software for Enhanced Polyp Detection: A Randomized Controlled Trial	Trial record only
NCT06116864 2023[446]	Enhancing Polyp Detection: A Randomized Controlled Trial Comparing Combined Computer-Aid Detection and Polyp-Detecting Colonoscope Attachment to Computer-Aid Detection Alone in Patients Undergoing Colonoscopy	Trial record only
NCT05888623 2023[447]	Computer Assisted Detection of Neoplasia During Colonoscopy Evaluation (CADeNCE)	Trial record only
NCT05829590 2023[448]	Speech and Image Recognition Based System in Improving Reporting Quality During Colonoscopy	Trial record only
NCT05822895 2023[449]	A Comprehensive Review of the Impact of a COmPuter-aIded reaL-time pOlyp deTection System on Adult Colonoscopy (COPILOT Study) - a Single Institution Adoption Experience	Trial record only
NCT05730192 2023[450]	Evaluation of Artificial Intelligence for Detection of Gastrointestinal Lesions in Endoscopy (EAGLE)	Trial record only
NCT05829447 2023[451]	Impact on Polyp Detection of a Computer Aided Detection System (CADEYE) Combined With a Balloon Mucosal Exposure Device (G-EYE 760R) in Individuals Participating in a Organized Colorectal Cancer Screening Program	Trial record only

NCT05687318 2023[452]	A Clinical Trial of the Effectiveness and Safety of Software Assisting Diagnose the Intestinal Polyp Digestive Endoscopy by Analysis of Colonoscopy Medical Images From Electronic Digestive Endoscopy Equipment, a Prospective, Multicenter, Randomized Stratified Block, Incomplete Blind Setting, Para...	Trial record only
NCT05990218 2023[453]	Efficacy of Artificial Intelligence in the Detection of Right Sided Colonic Polyp in Operators with Different Endoscopic Experience: a Randomized Control Trial	Trial record only
NCT05734820 2023[454]	Real-time Computer-aided Polyp/Adenoma Detection During Screening Colonoscopy: a Single-center Crossover Trial	Trial record only
NCT06059378 2023[455]	Using Artificial Intelligence-assisted Optical Polyp Diagnosis for Diminutive Colorectal Polyps	Trial record only
NCT05754229 2023[456]	Accuracy of Real Time Characterization in Artificial Intelligence-assisted Colonoscopy - A Prospective Quality Assurance Study	Trial record only
NCT05942677 2023[457]	Comparison of Flat Colorectal Lesion Detection by Artificial Intelligence-assisted Colonoscopy Versus Endoscopists: AIChallenge - Medtronic	Trial record only
NCT05963724 2023[458]	Efficacy of Real-Time Computer Aided-Detected of Colonic Neoplasia in an Underserved Population, A Randomized Controlled Trial	Trial record only

NCT05943288 2023[459]	Safety and Efficacy of the Olympus Endoscopy Computer-Aided Detection (CADe) System in Detection of Colorectal Neoplasia's During Real-time Colonoscopy: A European Prospective, Multicenter, Randomized Controlled Trial (EuroCADe)	Trial record only
NCT06063720 2023[460]	A Prospective Evaluation of the Correlation Between Artificial Intelligence-derived Effective Withdrawal Time and Adenoma Detection Rate	Trial record only
NCT05619614 2022[461]	The Influence of Artificial Intelligence (AI) Assisted Polyp Detection (Discovery System) on Visual Gaze Patterns During Real-time Colonoscopy	Trial record only
NCT05542030 2022[462]	Accuracy of CAD Eye in the Detection of Colonic Remaining Lesions After Endoscopic Mucosal Resection: a Pilot Study	Trial record only
NCT05594576 2022[463]	Comparison of the ENDOCUFF VISION Endoscopy Cap Coupled with GI GENIUSTM Artificial Intelligence Compared to Each Device Alone in Improving Colonic Adenoma Detection Rate During Colonoscopy	Trial record only
NCT05523271 2022[464]	A Multi-center Control Study to Determine the Efficacy of CADEYE in Detecting Colon Polyps in Comparison to Standard of Care	Trial record only
NCT05414383 2022[465]	A Prospective Study to Evaluate the Diagnostic Accuracy of Computer-aided Diagnosis (CADx) System in Real-time Characterization of Colorectal Neoplasia	Trial record only

NCT05349110 2022[466]	Real Time Computer-aided Diagnosis (CADx) of Diminutive Colorectal Polyps Using Artificial Intelligence	Trial record only
NCT05318495 2022[467]	A Dual Tandem Study Comparing the Adenoma Detection and Miss-rate of SC to That of Artificial Intelligence (CAD-EYE) Aided Colonoscopy and to That of Artificial Intelligence (CAD-EYE) and G-EYE Aided Colonoscopy	Trial record only
NCT05423964 2022[468]	Impact of Artificial Intelligence on Trainee Adenoma Detection Rate	Trial record only
NCT05323279 2022[469]	Evaluate the Effects of An Artificial Intelligence System on Colonoscopy Quality of Novice Endoscopists: A Randomized Controlled Trial	Trial record only
NCT05500248 2022[470]	Impact of Computer-aided Optical Diagnosis (CADx) in Predicting Histology of Diminutive Rectosigmoid Polyps: a Multicenter Prospective Trial	Trial record only
NCT05236790 2022[471]	Performance Evaluation of CAD-EYE and SCALE-EYE for Detection, Classification, and Measurement of Colorectal Polyps: a Prospective Study	Trial record only
NCT05244278 2022[472]	Artificial Intelligence (AI) Assisted Real-time Adenoma Detection and Classification During Colonoscopies	Trial record only
NCT05237310 2022[473]	Dual Tandem Study Comparing the Adenoma Detection and Miss-rate of Standard Colonoscopy to That of Artificial Intelligence (CAD-EYE)	Trial record only

	and to That of Artificial Intelligence (CAD-EYE) and G-EYE Aided Colonoscopy	
NCT05220345 2022[474]	Synergistic Effect of G-Eye Balloon for Behind the Folds Visualization With Artificial Intelligence Assisted Polyp Detection (Discovery System) on Adenoma Detection Rate. 'Discovery III Study'	Trial record only
NCT05391477 2022[475]	Efficacy and Cost-effectiveness of an Artificial Intelligence System (GI-Genius) on the Characterization of Diminutive Colorectal Polyps Within a Colorectal Cancer Screening Program: a Multicenter Randomized Controlled Trial (ODDITY Trial)	Trial record only
NCT05214625 2022[476]	Questionnaire Study Concerning Artificial Intelligence and Its Application in (Gastrointestinal) Healthcare - Patients' and Physicians' Perspectives	Trial record only
NCT05611151 2022[477]	Real-time Computer-Aided Detection of Colonic Adenomas With NEC WISE VISION Endoscopy: Prospective, Randomized Clinical Performance Evaluation	Trial record only
NCT05492656 2022[478]	Accuracy and Feasibility of CADx System for White Light Colonic Polyp Characterization	Trial record only
NCT05448300 2022[479]	Adenoma Detection Using Real-Time Computer-Aided Colon Polyp Detection System to Compare Water Exchange and Air Insufflation - A Randomized Controlled Trial	Trial record only

NCT05414448 2022[480]	Combination of Artificial Intelligence (ENDOAID) and Mucosal Exposure Device (ENDOCUFF) to Enhance Colorectal Neoplasia Detection: a Randomized Controlled Trial	Trial record only
NCT05322993 2022[481]	Improving Polyp Detection Rate by Artificial Intelligence in Colonoscopy	Trial record only
NCT05034185 2021[482]	Real-World Validation of an Artificial Intelligence Characterization Support (CADx) System for Prediction of Polyp Histology in Colonoscopy: A Prospective Multicentre Study	Trial record only
NCT05141773 2021[483]	Computer-assisted Adenoma Detection Coloscopy With Endo-AID Artificial Intelligence System and Endocuff Versus Endocuff Assisted Colonoscopy: a Randomized Controlled Trial	Trial record only
NCT05171634 2021[484]	Impact of Artificial Intelligence in Dysplasia Detection During Colonoscopy in Patients With Long-data Ulcerative Colitis: a Crossover Study	Trial record only
NCT05178095 2021[485]	Artificial Intelligence in Colonic Polyp Detection	Trial record only
NCT05133544 2021[486]	Endocuff With or Without Artificial Intelligence-assisted Colonoscopy in Detection of Colorectal Adenoma: a Randomized Colonoscopy Trial	Trial record only
NCT05141409 2021[487]	The COMBO CAD Study: Characterization cOMparison Between twO CAD Systems	Trial record only

NCT05064124 2021[488]	Early DiAgnosis Real-Time Healthcare System for CANcer Trial	Trial record only
NCT04915833 2021[489]	Real-time Computer-aided Polyp Detection During Screening Colonoscopy Performed by Expert Endoscopists	Trial record only
NCT04945044 2021[490]	Usefulness of the Endo-AID Artificial Intelligence System in the Detection of Colorectal Adenomas. a Randomized Controlled Trial	Trial record only
NCT04979962 2021[491]	Colorectal Polyp Detection Comparing Computer Assisted Colonoscopy With Conventional Colonoscopy	Trial record only
NCT04921488 2021[492]	Cross-sectional, Multi-center Study Comparing Diagnostic Performance Between the CAD EYE System and the Physician on Histological Prediction of Colonic Polyps in Screening of Colorectal Cancer by Colonoscopy	Trial record only
NCT04884581 2021[493]	The CHANGE Study: Characterization Helping the Assessment of Colorectal Neoplasia in Gastrointestinal Endoscopy	Trial record only
NCT04749277 2021[494]	Artificial Intelligence in the Characterization of Small and Diminutive Colorectal Polyps: A Prospective Study in a Clinical Setting Using CAD EYE	Trial record only
NCT04777019 2021[495]	Computer Aided Polyp Detection (C3PO) Trial: A Multicenter International Trial Evaluating Diagnostic Accuracy of Artificial Intelligence System in Detecting Colon Polyps	Trial record only

NCT04723758 2021[496]	COLO-DETECT: A Randomised Controlled Trial of Lesion Detection at Colonoscopy Using the GI Genius Artificial Intelligence Platform	Trial record only
NCT04747665 2021[497]	Patient and Endoscopists' Experiences and Perceptions of Colonoscopy and New Technologies in Colonoscopy	Trial record only
NCT04727814 2021[498]	Polyp Detection and False Alarm Rates by Computer-Aided Analysis of Videos of Withdrawal Phase of Colonoscopy in a Randomized Controlled Trial of Water Exchange Versus Air Insufflation	Trial record only
NCT05080088 2021[499]	A Pilot Study: Retrospective Evaluation of 3 Colonic Adenoma Detection Strategies During a Colonoscopy: Endoscopy Cap Associated With the Artificial Intelligence GI GENIUS TM System, the Artificial Intelligence GI GENIUS TM Alone and Colonoscopy Alone	Trial record only
NCT05089071 2021[500]	Effect of Two Colonoscopy AI Systems for Colon Polyp Detection According to the False Positive Rates of the Systems: A Single-center Prospective Study	Trial record only
NCT05158725 2021[501]	Comparison of Colonoscopy Adenoma Detection Yield of Standard Colonoscopy, Discovery Aided Colonoscopy, and Discovery and G-EYE Aided Colonoscopy	Trial record only
NCT05013125 2021[502]	Assessment of Efficacy of ENDO-AID Assisted Colonoscopy in Adenoma Detection: a Single Centre Randomised Controlled Trial	Trial record only

NCT04912037 2021[503]	A Study on the Effectiveness of Artificial Intelligence-assisted Colonoscopy in Improving the Effect of Colonoscopy Training for Trainees	Trial record only
NCT04442607 2020[504]	Clinical vAliDation of ARTificial Intelligence in POlyp Detection	Trial record only
NCT04260321 2020[505]	The AID Study 2: Artificial Intelligence for Colorectal Adenoma Detection 2	Trial record only
NCT04607083 2020[506]	Impact of Computer-aided Optical Diagnosis (CAD) in Predicting Histology of Diminutive Rectosigmoid Polyps: a Multicenter Prospective Trial (Artificial Intelligence BLI Characterization - ABC Study)	Trial record only
NCT04453956 2020[507]	A Prospective, Randomized, Single-blind, 2 x 2 Factorial Design Single Center Study Evaluating the Polyp Detection and Quality Monitoring Function of EndoAngel in Improving the Quality of Colonoscopy	Trial record only
NCT04294355 2020[508]	Artificial Intelligence-assisted Colonoscopy Versus Conventional Colonoscopy for Missed Lesions in the Proximal Colon: A Prospective Multi-center Randomized Study in Asia	Trial record only
NCT04894708 2020[509]	Prospective Randomized Study on the Use of Artificial Intelligence (Fujifilm) for Polyp Detection in Colonoscopy	Trial record only
NCT04485715 2020[510]	Application of Artificial Intelligence in Colorectal Polypectomy	Trial record only

NCT04610177 2020[511]	Prospective Multicenter Study of Artificial Intelligence-assisted Quality Evaluation System for Colonoscopy	Trial record only
NCT04676308 2020[512]	The CERTAIN Study: Combining Endo-cuff in a Randomized Trial for Artificial Intelligence Navigation	Trial record only
NCT04640792 2020[513]	A Randomized Two Arm Multi-Center Study to Evaluate the Safety and Efficacy of the Use of Magentiq Eye's Automatic Polyp Detection System (ME-APDS) During Colonoscopy	Trial record only
NCT04586556 2020[514]	Artificial Intelligence for Real-time Detection and Monitoring of Colorectal Polyps	Trial record only
NCT04440865 2020[515]	Impact of Artificial Intelligence Genius System-assisted Colonoscopy vs. Standard Colonoscopy on Adenoma Detection Rate in Routine Practice: a Prospective Randomized Controlled Trial	Trial record only
NCT04399590 2020[516]	Comparing the Number of False Activations Between Two Artificial Intelligence CADe Systems: the NOISE Study	Trial record only
NCT04422548 2020[517]	Does AI-assisted Colonoscopy Improve Adenoma Detection in Screening Colonoscopy? A Multi-center Randomized Controlled	Trial record only
NCT04359355 2020[518]	Development and Validation of a New Artificial Intelligence System for Automated Detection of Colorectal Polyps During Colonoscopy	Trial record only

NCT04335318 2020[519]	Real Life AI in Polyp Detection	Trial record only
NCT04349787 2020[520]	Improving Optical Diagnosis of Colorectal Polyps Using Computer-aided Diagnosis (CADx) and the BLI Adenoma Serrated International Classification (BASIC)	Trial record only
NCT04378660 2020[521]	Artificial Intelligence Validation Trial for Polyp Detection: Pilot Study	Trial record only
NCT04227795 2020[522]	Artificial Intelligence-Assisted Real-time Detection of Missed Lesions During Colonoscopy: A Prospective Study	Trial record only
NCT04441580 2020[523]	Resa Diagnostica Aggiuntiva Dell'Intelligenza Artificiale Nella Colonscopia (GENIAL COLONOSCOPY), Per lo Screening Del Carcinoma Colorettale	Trial record only
NCT04589078 2020[524]	Polyp REcognition Assisted by a Device Interactive Characterization Tool - The PREDICT Study	Trial record only
NCT04325815 2020[525]	Multi-Centre, Open-label, Randomised, Prospective Trial to Assess Efficacy and Safety of the CADDIE Artificial Intelligence System for Improving Endoscopic Detection of Colonic Polyps in Real-time	Trial record only
NCT04216901 2019[526]	A Single Center Study on the Effectiveness and Safety of Polyp Detection and Polyp Classification With Artificial Intelligence	Trial record only
NCT04079478 2019[527]	The AID Study: Artificial Intelligence for Colorectal Adenoma Detection	Trial record only

NCT04102631 2019[528]	A Prospective, Randomized, Single-blind, Parallel-controlled Multicenter Study Evaluating the Effectiveness of Endo.Angel in Improving the Quality of Colonoscopy	Trial record only
NCT03925337 2019[529]	Computer Aided Detection of Polyps in the Colon	Trial record only
NCT04074577 2019[530]	Computer Aided Detection, Tandem Colonoscopy Study	Trial record only
NCT03954548 2019[531]	Prospective, Randomized, Multicenter, Tandem Study Evaluating the Safety and Effectiveness of the CB-17-08 Augmented Endoscopy System for the Detection of Mucosal Colorectal Polyps in Adult Patients Undergoing Screening or Surveillance Colonoscopy for CRC	Trial record only
NCT03842059 2019[532]	Computer-aided Detection With Deep Learning for Colorectal Adenoma During Colonoscopic Examination	Trial record only
NCT03775811 2018[533]	In Vivo Computer-aided Prediction of Polyp Histology on White Light Colonoscopy	Trial record only
NCT03637712 2018[534]	Deep-Learning for Automatic Polyp Detection During Colonoscopy	Trial record only
NCT03761771 2018[535]	Validating the Performance of Artificial Intelligence in Identifying Polyps in Real-world Colonoscopy	Trial record only
NCT03359343 2017[536]	Computer-aided Classification of Colorectal Polyp by Using Linked Colour Imaging	Trial record only

NCT03069833 2017[537]	Computer Aided Diagnosis of Colorectal Neoplasms During Colonoscopic Examination	Trial record only
NCT02838888 2016[538]	Computer-assisted Detection of Colonic Polyps	Trial record only
Nguyen 2024[539]	AI-Assisted Colonoscopy May Increase ADR in Right Side of Colon in Gastroenterology Fellows: A Randomized Control Trial	Abstract only and have full texts covering outcomes
Nguyen 2023[540]	Artificial Intelligence-Assisted Colonoscopy Improves ADR of Gastroenterology Fellows: Results of a Prospective Cohort Study	Non-randomised study and outcomes covered by randomised trials
NL-OMON22821 2020[541]	Discovery II Study	Trial record only
NL-OMON49600 2020[542]	Discovery: pentax* Computer-aided Detection to Improve Adenoma Detection in a Real-time Setting - The Discovery II Study. A randomized clinical trial	Trial record only
NL-OMON50965 2021[543]	Randomized Two Arm Multi-Center Study to Evaluate the Safety and Efficacy of the Use of Magentiq Eye's Automatic Polyp Detection System (ME-APDS) During Colonoscopy	Trial record only
NL-OMON51017 2021[544]	Effect of a computer-aided detection system (CAD EYE) on adenoma detection in patients with Lynch syndrome: an international, multicenter parallel randomized controlled trial	Trial record only

Norwood 2024[545]	LEAVING NO STONE UNTURNED - ROLE OF ARTIFICIAL INTELLIGENCE IN DETECTION OF SMALLER LESIONS DURING COLONOSCOPY	Unnamed intervention
Norwood 2024[546]	Performance of Computer-Aided Detection and Quality of Bowel Preparation: A Comprehensive Analysis of Colonoscopy Outcomes	Non-randomised study and outcomes covered by randomised trials
Okiye 2024[547]	A Comparison of Distal Attachment Devices, Artificial Intelligence, and Standard Colonoscopy for Adenoma Detection Rate and Withdrawal Times: Advantage or Hindrance	Unnamed intervention
Oleksiw 2025[548]	ARTIFICIAL INTELLIGENCE AND ENDOSCOPIST DIAGNOSTIC AGREEMENT AS A FRAMEWORK FOR COLORECTAL POLYP OPTICAL DIAGNOSIS IMPLEMENTATION	Unnamed intervention
Oleksiw 2025[549]	IMPLEMENTATION STRATEGIES TO OPTIMIZE DIAGNOSTIC ACCURACY OF COMPUTER-ASSISTED OPTICAL POLYP DIAGNOSIS	Unnamed intervention
O'Mara 2022[550]	Comparing the Adenoma Detection Rate of Endocuff-Assisted Colonoscopy (EAC) Against Combined Artificial Intelligence and Endocuff-Assisted Colonoscopy (AEAC)	Unnamed intervention
Okumura 2024[551]	Evaluating false-positive detection in a computer-aided detection system for colonoscopy.	Technology not relevant to review

Olabintan 2024[552]	ENVIRONMENTAL IMPACT OF OPTICAL DIAGNOSIS BY ARTIFICIAL INTELLIGENCE IN COLONOSCOPY: A PROSPECTIVE TRIAL	Unnamed intervention
Ortiz 2024[553]	EVALUATION OF ARTIFICIAL INTELLIGENCE-ASSISTED COLONOSCOPY FOR ADENOMA DETECTION IN LYNCH SYNDROME: A MULTICENTRE RANDOMIZED CONTROLLED TRIAL (TIMELY STUDY)	Abstract of full publication identified
Ortiz 2024[554]	Evaluation of Artificial Intelligence-Assisted Colonoscopy for Adenoma Detection in Lynch Syndrome: a multicentre randomized controlled trial (Timely study)	Abstract of full publication identified
Orzeszko 2024[555]	Effect of artificial intelligence implementation to the latest generation 4K colonoscopy	Non-randomised study and outcomes covered by randomised trials
Pagador 2025[556]	Identification of clinical needs for the improvement of ai-assisted colonoscopy cad systems	Limited usable data/no relevant outcomes
Pal 2024[557]	Artificial intelligence in endoscopy related to inflammatory bowel disease: A systematic review.	Systematic review used for reference checking
Pan 2021[558]	Artificial Intelligence-Aid Colonoscopy Vs. Conventional Colonoscopy for Polyp and Adenoma Detection: A Systematic Review of 7 Discordant Meta-Analyses.	Systematic review used for reference checking
Pannala 2020[559]	Artificial intelligence in gastrointestinal endoscopy.	Systematic review used for reference checking

Park 2024[560]	A prospective multicenter randomized controlled trial on artificial intelligence assisted colonoscopy for enhanced polyp detection	Technology not relevant to review
Pasam 2023[561]	ADENOMA DETECTION RATES WITH COMPUTER AIDED COLONOSCOPY AND DISTAL ATTACHMENT MUCOSAL EXPOSURE DEVICES - A SYSTEMATIC REVIEW AND NETWORK META-ANALYSIS	Abstract of systematic review
Patel 2024[562]	AI IN COLONOSCOPY FOR THE DETECTION OF COLORECTAL NEOPLASIA: A META-ANALYSIS OF RANDOMIZED AND NON-RANDOMIZED STUDIES	Abstract of systematic review
Patel 2024[563]	BENEFITS AND HARMS OF INCORPORATING AI DURING COLONOSCOPY FOR TRAINEES: A SYSTEMATIC REVIEW AND META-ANALYSIS OF PUBLISHED LITERATURE	Abstract of systematic review
Patel 2023[564]	COMPARISON OF MEAN ADENOMA PER COLONOSCOPY USING ARTIFICIAL INTELLIGENCE SYSTEMS VS STANDARD COLONOSCOPY: A SYSTEMATIC REVIEW AND META-ANALYSIS OF RANDOMIZED CONTROLLED STUDIES	Abstract of systematic review
Patel 2023[565]	EXISTING ARTIFICIAL INTELLIGENCE SYSTEMS DO NOT IMPROVE SESSILE SERRATED LESION DETECTION: A META-ANALYSIS OF RANDOMIZED CONTROLLED STUDIES	Abstract of systematic review

Patel 2024[566]	AI IN COLONOSCOPY BENEFITS THOSE ENDOSCOPISTS WITH LOW BASELINE ADR: AN AGGREGATE META-ANALYSIS AND META-REGRESSION OF ENDOSCOPIST-LEVEL DATA	Abstract of systematic review
Patel 2023[567]	COMPARISON OF ADENOMA DETECTION RATE IN SCREENING OR SURVEILLANCE COLONOSCOPY USING ARTIFICIAL INTELLIGENCE VS STANDARD COLONOSCOPY: A META-ANALYSIS OF RANDOMIZED CONTROLLED STUDIES	Abstract of systematic review
Patel 2021[568]	Artificial intelligence increases adenoma detection even in 'high-detector' colonoscopy: Early evidence for human: machine interaction	Non-randomised study and outcomes covered by randomised trials
Patel 2024[569]	Lack of Effectiveness of Computer Aided Detection for Colorectal Neoplasia: A Systematic Review and Meta-Analysis of Nonrandomized Studies.	Systematic review used for reference checking
Patel 2025[570]	ROLE OF ENDOSCOPIST ON PERFORMANCE OF ARTIFICIAL INTELLIGENCE IN NEOPLASIA DETECTION DURING COLONOSCOPY: METAANALYSIS AND METAREGRESSION OF ENDOSCOPIST LEVEL DATA FROM 25 STUDIES	Abstract of systematic review
Patel 2023[571]	Artificial Intelligence in Colonoscopy in the Community Setting	Unnamed intervention
Patel 2024[572]	Lack of Effectiveness of Computer Aided Detection for Colorectal Neoplasia: a Systematic Review and Meta-analysis of Non-Randomized Studies	Abstract of systematic review

Pecere 2022[573]	Endoscopists performance in optical diagnosis of colorectal polyps in artificial intelligence studies.	Systematic review used for reference checking
Pfeifer 2021[574]	Computer-aided detection of colorectal polyps using a newly generated deep convolutional neural network: from development to first clinical experience.	Non-randomised study and outcomes covered by randomised trials
Prijic 2022[575]	ARTIFICIAL INTELLIGENCE: A NEW TOOL IN ENDOSCOPIC SURVEILLANCE IN INFLAMMATORY BOWEL DISEASE PATIENTS	Limited usable data/no relevant outcomes
Putera 2024[576]	REAL-WORLD STUDY OF SEQUENTIAL IMPLEMENTATION OF PHYSICIAN ADR REPORTING AND CAD-E SYSTEM IN IMPROVING PRAGMATIC ADENOMA DETECTION RATE (ADR) IN ELECTIVE COLONOSCOPY IN A TERTIARY HOSPITAL IN SINGAPORE	Non-randomised study and outcomes covered by randomised trials
Quan 2019[577]	Artificial intelligence based computer aided detection system reliably detects polyps earlier than physicians during colonoscopy	Unnamed intervention
Quan 2021[578]	Increased Polyp Detection In A Western Population Using A Real-Time Artificial Intelligence-Based System During Colonoscopy: A Pilot Study	Unnamed intervention
Quan 2022[579]	Clinical evaluation of a real-time artificial intelligence-based polyp detection system: a US multi-center pilot study.	Technology not relevant to review
Qaqish 2024[580]	The Impact of Artificial Intelligence-Assisted Colonoscopy on Key Colonoscopy Quality Indicators in the Underserved Population	Unnamed intervention

Radadiya 2023[581]	ARE ALL COMPUTER-AIDED DETECTION SYSTEMS (CADE) CREATED EQUAL? - COMPARING ADENOMA DETECTION RATE OF DIFFERENT CADE SYSTEMS: NETWORK META-ANALYSIS OF RANDOMIZED CONTROLLED TRIALS	Abstract of systematic review
Rath 2020[582]	Computer-aided detection of colorectal polyps using a newly generated deep convolutional neural network	Unnamed intervention
Renelus 2023[583]	AI ASSISTED COLONOSCOPY INCREASES ADENOMA DETECTION IN AVERAGE RISK SCREENING POPULATION	Unnamed intervention
Renelus 2023[584]	Computer-Aided Detection Device Increase Adenoma Detection in Anemic Patients Undergoing Diagnostic Colonoscopy	Unnamed intervention
Repici 2020[585]	876 REAL-TIME COMPUTER AIDED DIAGNOSIS FOR DETECTION OF COLORECTAL NEOPLASIA AT COLONOSCOPY	Abstract of full publication identified
Repici 2021[586]	Efficacy of real-time computer-aided detection of colorectal neoplasia in a non-expert setting: A randomized controlled trial	Abstract of full publication identified
Rex 2024[587]	Artificial Intelligence for Real-Time Prediction of the Histology of Colorectal Polyps by General Endoscopists.	Unnamed intervention
Rex 2022[588]	Strengths and Weaknesses of an Artificial Intelligence Polyp Detection Program as Assessed by a High-Detecting Endoscopist.	Limited usable data/no relevant outcomes

Rex 2025[589]	Detection of large flat colorectal lesions by artificial intelligence: a persistent weakness and blind spot	Full text not retrieved
Richter 2022[590]	INFLUENCE OF ARTIFICIAL INTELLIGENCE ON THE ADENOMA DETECTION RATE THROUGHOUT THE DAY	Non-randomised study and outcomes covered by randomised trials
Richter 2023[591]	Influence of Artificial Intelligence on the Adenoma Detection Rate throughout the Day.	Non-randomised study and outcomes covered by randomised trials
Rizkala 2024[592]	Accuracy of Computer-aided Diagnosis in Colonoscopy Varies according to Polyp Location. A Systematic Review and Meta-analysis.	Systematic review used for reference checking
Rizkala 2025[593]	Role of Artificial Intelligence for Colon Polyp Detection and Diagnosis and Colon Cancer.	Systematic review used for reference checking
Robles-Medranda 2023[594]	REAL-TIME COMPUTER-AIDED POLYP AND ADENOMA DETECTION DURING SCREENING COLONOSCOPY IN EXPERT AND NON-EXPERT ENDOSCOPISTS: A SINGLE CENTER STUDY	Unnamed intervention
Robles-Medranda 2022[595]	REAL-TIME COMPUTER-AIDED POLYP/ ADENOMA DETECTION DURING SCREENING COLONOSCOPY: A SINGLE-CENTER DIAGNOSTIC TRIAL	Non-randomised study and outcomes covered by randomised trials
Robles-Medranda 2022[596]	REAL-TIME COMPUTER-AIDED POLYP/ADENOMA DETECTION DURING SCREENING COLONOSCOPY: A SINGLE-CENTER DIAGNOSTIC TRIAL	Non-randomised study and outcomes covered by randomised trials

Roccato 2019[597]	Artificial intelligence-aided colonoscopy: A retrospective analysis of effect on procedure time	Unnamed intervention
Rocchetto 2024[598]	Combining a Computer Aided Detection system (CADe) and G-EYE balloon for adenoma detection in a FIT-based organized colorectal cancer screening program: preliminary results of a randomized controlled trial	Unnamed intervention
Rodriguez-Diaz 2019[599]	ARTIFICIAL INTELLIGENCE AUGMENTS REAL-TIME HISTOLOGY OF COLON POLYPS USING COMBINED-MODALITY NARROW BAND IMAGE CLASSIFICATION AND ELASTIC-SCATTERING SPECTROSCOPY	Technology not relevant to review
Rodriguez-Diaz 2023[600]	REAL TIME CLINICAL VALIDATION OF COMPUTER AIDED DIAGNOSIS WITH AUGMENTED REALITY FOR HISTOLOGY ASSESSMENT OF COLORECTAL POLYPS	Unnamed intervention
Rodriguez-Diaz 2022[601]	EVALUATION OF REAL-TIME COLORECTAL POLYP HISTOLOGY ASSESSMENT USING COMPUTER AIDED DIAGNOSIS WITH AUGMENTED REALITY VISUALIZATION: PRELIMINARY RESULTS FROM PILOT STUDY	Unnamed intervention
Ronborg 2022[602]	Can artificial intelligence improve the quality of colonoscopy investigations? Evaluation of the GI genius endoscopy module in daily clinical practice	Abstract only and have full texts covering outcomes

Ronborg 2024[603]	Assessing the potential of artificial intelligence to enhance colonoscopy adenoma detection in clinical practice: a prospective observational trial.	Non-randomised study and outcomes covered by randomised trials
Rondonotti 2021[604]	COMPUTER-AIDED OPTICAL DIAGNOSIS OF DIMINUTIVE RECTOSIGMOID POLYPS IN CLINICAL PRACTICE: A MULTICENTER PROSPECTIVE STUDY	Abstract only and have full texts covering outcomes
Sabbagh 2024[605]	Ceiling Effect of CADe in Improving Detection Rates in FIT Positive Cases Among Gastroenterologists With High Baseline ADR	Non-randomised study and outcomes covered by randomised trials
Sabran 2024[606]	Artificial intelligence-assisted colonoscopy detection rate on colorectal neoplasia patient: a systematic review of RCTs	Abstract of systematic review
Saleepol 2024[607]	IMPACT OF A REAL-TIME COMPUTER-AIDED POLYP CHARACTERIZATION IN SCREENING COLONOSCOPY PERFORMED BY TRAINEES VERSUS EXPERIENCED ENDOSCOPISTS: A RANDOMIZED CONTROLLED TRIAL	Abstract only and have full texts covering outcomes
Salvi 2023[608]	BENEFITS FROM A COMPUTER-AIDED DETECTION DEVICE IN COLONOSCOPY (ACCENDO-COLO) - AN INTERIM ANALYSIS OF AN ITALIAN MULTICENTER RANDOMIZED CLINICAL TRIAL	Abstract only and have full texts covering outcomes
Samuel 2023[609]	Impact of AI-Assisted Colonoscopy on Sessile Serrated Adenoma Detection in a Community GI Practice Setting	Non-randomised study and outcomes covered by randomised trials

Sanchez-Peralta 2020[610]	Deep learning to find colorectal polyps in colonoscopy: A systematic literature review.	Systematic review used for reference checking
Satiya 2020[611]	Is Artificial Intelligence for Colonoscopy Ready for Prime-Time: A Systematic Review and Meta-Analysis of Randomized Controlled Trials	Abstract of systematic review
Sato 2024[612]	A MULTICENTER SINGLE-ARM PROSPECTIVE STUDY TO ASSESS THE PERFORMANCE OF AN ARTIFICIAL INTELLIGENCE TO SUPPORT CHARACTERIAZTION OF COLORECTAL POLYPS	Unnamed intervention
Scalvini 2025[613]	Strategies to Enhance the Adenoma Detection Rate (ADR) and the Serrated Polyp Detection Rate (SPDR) in Colonoscopy: A Comprehensive Review	Systematic review used for reference checking
Schacher 2024[614]	ARTIFICIAL INTELLIGENCE IN COLONOSCOPY: A REAL-WORLD EVALUATION	Abstract only and have full texts covering outcomes
Schauer 2022[615]	Artificial intelligence improves adenoma detection rate during colonoscopy.	Non-randomised study and outcomes covered by randomised trials
Schauer 2021[616]	Artificial intelligence for polyp detection during colonoscopy: A win for humans?	Non-randomised study and outcomes covered by randomised trials
Schmidt 2024[617]	PATIENT PERSPECTIVES AND ACCEPTABILITY OF ARTIFICIAL INTELLIGENCE USED DURING SCREENING COLONOSCOPY	Unnamed intervention

Schrader 2022[618]	Artificial intelligence in screening colonoscopy	Systematic review used for reference checking
Seager 2022[619]	Trial protocol for COLO-DETECT: A randomized controlled trial of lesion detection comparing colonoscopy assisted by the GI Genius TM artificial intelligence endoscopy module with standard colonoscopy.	Trial protocol only
Seager 2024[620]	THE EFFECT OF ENDOCUFF VISION ON POLYP DETECTION DURING COLONOSCOPY WITH THE GI GENIUS COMPUTER-AIDED DETECTION DEVICE	Post-hoc analysis of a trial included in the review with no relevant additional data
Seager 2024[621]	BMJ GUT BEST LABORATORY SCIENCE ABSTRACT: COLO-DETECT: A RANDOMISED CONTROLLED TRIAL OF THE GI GENIUS AI DEVICE FOR POLYP DETECTION DURING ROUTINE COLONOSCOPY	Abstract of full publication identified
Sekiguchi 2025[622]	Protocol for a multicenter randomized controlled trial to assess the usefulness of computer-aided detection systems for colonoscopy in colorectal cancer screening in the Asia-Pacific region (project CAD/NCCH2217)	Full text not retrieved
Sekiguchi 2023[623]	Cost-effectiveness analysis of computer-aided detection systems for colonoscopy in Japan.	Health economic assessment only
Shah 2022[624]	ARTIFICIAL INTELLIGENCE AND COMPUTER AIDED DETECTION (CADE) SYSTEMS IMPROVE ADENOMA MISS RATES, ADENOMA	Abstract of systematic review

	DETECTION RATES AND POLYP DETECTION RATES: A SYSTEMATIC REVIEW AND META-ANALYSIS	
Shah 2023[625]	Effect of computer-aided colonoscopy on adenoma miss rates and polyp detection: A systematic review and meta-analysis.	Systematic review used for reference checking
Shah 2025[626]	ASSESSING THE ECONOMIC IMPACT AND CLINICAL BENEFITS OF ARTIFICAL INTELLIGENCE (AI)- ASSISTED COLONOSCOPY USING SIMULATION MODELS: IS AI WORTH THE HYPE? - A SYSTEMATIC REVIEW AND META-ANALYSIS	Abstract of systematic review
Shao 2023[627]	Comparing Sessile Serrated Adenoma/Polyp Detection Rate Between Water Exchange and Computer-Aided Detection Colonoscopy Using Pooled Data From Randomized Controlled Trials	Abstract of systematic review
Shao 2023[628]	Comparing Advanced Adenoma Detection Rate Between Water Exchange and Computer-Aided Colonoscopy Using Pooled Data From Randomized Controlled Trials	Abstract of systematic review
Shao 2023[629]	Comparing Adenoma Detection Rate Between Computer-Aided Detection and Water Exchange Colonoscopy Using Pooled RCTs Data - An Interim Report	Abstract of systematic review
Shao 2024[630]	WATER EXCHANGE AND COMPUTER-AIDED DETECTION IMPROVED ADENOMA DETECTION RATE - A META-ANALYSIS OF POOLED DATA FROM RANDOMIZED CONTROLLED TRIALS	Abstract of systematic review

Shao 2024[631]	WATER EXCHANGE IS SUPERIOR TO COMPUTER-AIDED DETECTION IN DETECTING SESSILE SERRATED ADENOMA/POLYP	Abstract of systematic review
Shao 2022[632]	Effects of ai-assisted colonoscopy on adenoma miss rate/adenoma detection rate: A protocol for systematic review and meta-analysis.	Systematic review used for reference checking
Sharma 2024[633]	393 ARTIFICIAL INTELLIGENCE ASSISTED COLONOSCOPY IMPROVES PDR AND ADR IN RIGHT COLON IN PATIENTS WITH IBD: A COHORT STUDY IN A HIGH-VOLUME CENTER	Unnamed intervention
Shaukat 2021[634]	ID: 3526633 THE EFFECT OF CHANGING THE SPECIFICITY OF AN ARTIFICIAL INTELLIGENCE-AIDED POLYP DETECTION DEVICE AND ITS IMPACT ON CLINICAL PERFORMANCE	Unnamed intervention
Shaukat 2022[635]	Computer-Aided Detection Improves Adenomas per Colonoscopy for Screening and Surveillance Colonoscopy: A Randomized Trial.	Technology not relevant to review
Shen 2021[636]	Real-time use of a computer-aided system for polyp detection during colonoscopy, an ambispective study.	Technology not relevant to review
Shinozaki 2025[637]	Computer-aided diagnosis for colorectal polyp in comparison with endoscopists: Systematic review and meta-analysis	Systematic review used for reference checking

Siggens 2024[638]	THE FIRST EVER REAL-TIME EVALUATION OF A NOVEL CADE-IBD ALGORITHM FOR DETECTION OF NEOPLASIA DURING SURVEILLANCE COLONOSCOPY IN COLITIS PATIENTS	Technology not relevant to review
Siggens 2025[639]	A VALIDATION STUDY EVALUATING THE ADDED VALUE OF A NOVEL COMPUTER AIDED DETECTION SYSTEM FOR NEOPLASIA DETECTION IN INFLAMMATORY BOWEL DISEASE (CADE-IBD)	Technology not relevant to review
Siggens 2023[640]	VALIDATION AND REAL-TIME PERFORMANCE OF A NOVEL CAD-X ALGORITHM FOR CHARACTERISATION AND SIZING OF COLORECTAL POLYPS	Unnamed intervention
Siggens 2023[641]	VALIDATION OF A NOVEL CADE ALGORITHM FOR DETECTION OF NEOPLASIA IN IBD: DATA FROM IMAGE BASED AND REAL-TIME EVALUATION	Technology not relevant to review
Siggens 2023[642]	A NOVEL CADX ALGORITHM FOR CHARACTERISATION AND SIZING OF COLORECTAL POLYPS MEETS PIVI 1 AND PIVI 2 THRESHOLD	Technology not relevant to review
Singh 2019[643]	Artificial Intelligence for Colon Polyp Detection: Get a Machine for Your Unit Now!	Abstract only and have full texts covering outcomes
Sinonquel 2025[644]	THE CLINICAL VALIDATION OF A COMPUTER-ADIDE POLYP DETECTION MODEL INTEGRATED AS A PLUG-AND-PLAY ENDOSCOPY DEVICE (ALTER-EGO TRIAL)	Unnamed intervention

Sinonquel 2025[645]	CLINICAL IMPACT OF THE MISMATCH BETWEEN OPTICAL DIAGNOSIS AND HISTOLOGY: A POST- HOC SUB-ANALYSIS OF THE CAD-ARTIPOD TRIAL	Technology not relevant to review
Sinonquel 2020[646]	Artificial intelligence for colorectal polyp detection: A validation trial with real-time unblinding	Unnamed intervention
Sinonquel 2023[647]	CLINICAL VALIDATION OF A COMPUTER-AIDED DETECTION MODEL FOR COLORECTAL POLYP DETECTION (CAD-ARTIPOD) TRIAL USING A SECOND OBSERVER AND REAL-TIME UNBLINDING	Unnamed intervention
Sinonquel 2021[648]	Real-time unblinding for validation of a new CADe tool for colorectal polyp detection.	Technology not relevant to review
Sinonquel 2024[649]	Clinical consequences of computer-aided colorectal polyp detection.	Unnamed intervention
Sivananthan 2021[650]	ID: 3525827 PERFORMANCE OF COMPUTER AIDED DETECTION SYSTEMS IN FLAT, SESSILE AND DIMINUITIVE ADENOMAS: A META-ANALYSIS	Abstract of systematic review
Sivananthan 2022[651]	Does computer-aided diagnostic endoscopy improve the detection of commonly missed polyps? A meta-analysis.	Systematic review used for reference checking

Soleymanjahi 2023[652]	PERFORMANCE OF ARTIFICIAL INTELLIGENCE ASSISTED COLONOSCOPY VS CONVENTIONAL COLONOSCOPY: A SYSTEMATIC REVIEW AND META-ANALYSIS	Abstract of systematic review
Soleymanjahi 2024[653]	DOES COMPUTER-AIDED DETECTION (CADE) OFFER ANY ADVANTAGE TO CONVENTIONAL COLONOSCOPY: A COMPREHENSIVE COMPARISON OF EFFICACY AND SAFETY MEASURES?	Abstract of systematic review
Soleymanjahi 2024[654]	PERFORMANCE OF DIFFERENT COMPUTER-AIDED DETECTION (CADE) PLATFORMS COMPARED TO CONVENTIONAL COLONOSCOPY: A SYSTEMATIC REVIEW AND META-ANALYSIS	Abstract of systematic review
Soleymanjahi 2024[655]	PROVIDER TRUST TOWARDS ADOPTING REAL TIME ARTIFICIAL INTELLIGENCE IN COLONOSCOPY: A SYSTEMATIC REVIEW	Abstract of systematic review
Soleymanjahi 2024[656]	Artificial Intelligence-Assisted Colonoscopy for Polyp Detection	Systematic review used for reference checking
Soleymanjahi 2024[657]	Perceived Advantages and Disadvantages of Adopting Real Time Artificial Intelligence in Colonoscopy by Providers: A Systematic Review	Abstract of systematic review
Soo 2024[658]	Detection of sessile serrated adenoma using artificial intelligence-enhanced endoscopy: an Asian perspective.	Limited usable data/no relevant outcomes
Soons 2022[659]	Real-time colorectal polyp detection using a novel computer-aided detection system (CADe): a feasibility study.	Non-randomised study and outcomes covered by randomised trials

Spadaccini 2022[660]	COMPARING NUMBER AND RELEVANCE OF FALSE ACTIVATIONS BETWEEN TWO ARTIFICIAL INTELLIGENCE CADE SYSTEMS: THE NOISE STUDY	Unnamed intervention
Spadaccini 2022[661]	COMPARING NUMBER AND RELEVANCE OF FALSE ACTIVATIONS BETWEEN TWO ARTIFICIAL INTELLIGENCE CADE SYSTEMS: THE NOISE STUDY	Unnamed intervention
Spadaccini 2023[662]	REAL-TIME COMPUTER-AIDED DETECTION OF COLORECTAL NEOPLASIA DURING COLONOSCOPY: SYSTEMATIC REVIEW AND META-ANALYSIS	Abstract of systematic review
Spadaccini 2022[663]	PERFORMANCE OF AI-AIDED COLONOSCOPY FOR THE DETECTION OF HIGH-RISK COLORECTAL CANCER PRECURSORS: A SYSTEMATIC REVIEW AND META-ANALYSIS	Abstract of systematic review
Spadaccini 2024[664]	VARIABILITY IN COMPUTER-AIDED DETECTION EFFECT ON ADENOMA DETECTION RATE IN RANDOMIZED CONTROLLED TRIALS: A META-REGRESSION ANALYSIS	Abstract of systematic review
Spadaccini 2024[665]	ARTIFICIAL INTELLIGENCE AND COLORECTAL NEOPLASIA DETECTION PERFORMANCES IN FIT+ PATIENTS: A META-ANALYSIS AND SYSTEMATIC REVIEW	Abstract of systematic review

Spadaccini 2023[666]	IN VIVO CONCORDANCE BETWEEN TWO ARTIFICIAL INTELLIGENCE SYSTEMS FOR LEAVING IN SITU COLORECTAL POLYPS	Abstract only and have full texts covering outcomes
Spadaccini 2021[667]	ARTIFICIAL INTELLIGENCE VERSUS ADVANCED IMAGING FOR DETECTION OF COLORECTAL NEOPLASIA: A NETWORK METAANALYSIS	Systematic review used for reference checking
Spadaccini 2022[668]	Comparing the number and relevance of false activations between 2 artificial intelligence computer-aided detection systems: the NOISE study.	Non-randomised study and outcomes covered by randomised trials
Spadaccini 2023[669]	Combination of Mucosa-Exposure Device and Computer-Aided Detection for Adenoma Detection During Colonoscopy: A Randomized Trial.	Comparator not relevant to review
Spadaccini 2021[670]	Computer-aided detection versus advanced imaging for detection of colorectal neoplasia: a systematic review and network meta-analysis.	Systematic review used for reference checking
Spadaccini 2025[671]	Artificial intelligence and colorectal neoplasia detection performances in patients with positive fecal immunochemical test: Meta-analysis and systematic review	Systematic review used for reference checking
Spadaccini 2025[672]	Variability in computer-aided detection effect on adenoma detection rate in randomized controlled trials: A meta-regression analysis	Systematic review used for reference checking

Su 2020[673]	Impact of a real-time automatic quality control system on colorectal polyp and adenoma detection: a prospective randomized controlled study (with videos).	Technology not relevant to review
Sultan 2025[674]	AGA Living Clinical Practice Guideline on Computer-Aided Detection-Assisted Colonoscopy	Systematic review used for reference checking
Syed 2023[675]	Artificial Intelligence (CAD-E)-Assisted Colonoscopy Helps Increase Adenoma Detection Rate (ADR) in the Afternoon Session	Duplicate of abstract already included in review
Taghiakbari 2025[676]	PRAGMATIC IMPLEMENTATION OF RESECT AND DISCARD AND DIAGNOSE AND LEAVE STRATEGIES USING AUTONOMOUS COMPUTER-ASSISTED OPTICAL POLYP DIAGNOSIS	Abstract only and have full texts covering outcomes
Takasu 2025[677]	IMPACT OF INTRODUCING ARTIFICIAL INTELLIGENCE ON COLONOSCOPY IN REAL-WORLD CLINICAL PRACTICE	Non-randomised study and outcomes covered by randomised trials
Tanaka 2021[678]	Evaluation of cad eyeTM based on artificial intelligence technology for detection and characterization of colorectal neoplasia in a clinical setting	Abstract only and have full texts covering outcomes
Tang 2021[679]	Comparing polyp detection rate between water exchange and air insufflation by a computeraided detection algorithm: An analysis of withdrawal phase videos from a randomized controlled trial	Unnamed intervention
Tang 2022[680]	ADENOMA DETECTION USING REAL-TIME COMPUTER-AIDED COLON POLYP DETECTION SYSTEM TO COMPARE WATER	Unnamed intervention

	EXCHANGE AND AIR INSUFFLATION - A PILOT CONTROLLED STUDY	
Tang 2022[681]	Polyp detection and false-positive rates by computer-aided analysis of withdrawal-phase videos of colonoscopy of the right-sided colon segment in a randomized controlled trial comparing water exchange and air insufflation.	Technology not relevant to review
Tang 2021[682]	A review of water exchange and artificial intelligence in improving adenoma detection.	Study design - review not systematic
Tang 2025[683]	REAL-TIME COMPUTER-AIDED SYSTEM TO COMPARE RIGHT COLON ADENOMA DETECTION AND ADENOMA PER COLONOSCOPY RATES IN WATER EXCHANGE AND AIR INSUFFLATION - A RANDOMIZED CONTROLLED STUDY	Unnamed intervention
Tariq 2025[684]	Perception and Understanding of Artificial Intelligence Among Gastroenterology Fellows and Early Career Gastroenterologists: A Nationwide Cross-Sectional Survey Study	Mixed colonoscopy and other gastroenterology population
TCTR20200929003 2020[685]	Comparison of computer-aided diagnosis colonoscopy, Endocuff-Assisted Colonoscopy, combination of computer-aided diagnosis colonoscopy and Endocuff-Assisted Colonoscopy and High-definition Colonoscopy for Adenomas Detection in Colorectal Cancer Screening	Trial record only
TCTR20220826004 2022[686]	Comparison between two computer-aided diagnosis colonoscopy systems (Deep GI system and CAD EYE system) and High-definition	Trial record only

	Colonoscopy for Adenomas Detection in Colorectal Cancer Screening: a randomized control Trial	
TCTR20230504002 2023[687]	Adenoma Miss rate in Artificial Intelligence-Based versus Conventional Colonoscopy, A Prospective Randomized Trial	Trial record only
TCTR20230706006 2023[688]	Comparison between two computer-aided polyp detection colonoscopy systems and High-definition Colonoscopy for Adenomas Detection in Colorectal Cancer Screening: a multi-center randomized control Trial	Trial record only
TCTR20240710001 2024[689]	Using Computer-Aided polyp detection system (CADe) to maintain the high quality in adenoma detection rate during community-based colorectal cancer screening in Thailand: a randomized trial	Trial record only
Tham 2023[690]	Knowledge, perceptions and behaviours of endoscopists towards the use of artificial intelligence-aided colonoscopy.	Full text not retrieved
Thijssen 2024[691]	Improving the endoscopic recognition of early colorectal carcinoma using artificial intelligence: current evidence and future directions	Systematic review used for reference checking
Thiruvengadam 2023[692]	THE IMPACT OF COMPUTER-AIDED DETECTION ON PATIENTS UNDERGOING SCREENING COLONOSCOPY PERFORMED BY LOW- AND HIGH-DETECTOR ENDOSCOPISTS: A MICROSIMULATION ANALYSIS	Unnamed intervention
Thiruvengadam 2022[693]	COST-EFFECTIVENESS OF THE ADDITION OF REAL-TIME COMPUTER-AIDED DETECTION OF ADENOMAS TO SCREENING	Health economic assessment only

	COLONOSCOPY IN COLORECTAL CANCER SCREENING OF AVERAGE-RISK PERSONS AT 45 YEARS OF AGE	
Thiruvengadam 2023[694]	EFFICACY OF REAL-TIME COMPUTER AIDED-DETECTED OF COLONIC NEOPLASIA IN AN UNDERSERVED POPULATION, A RANDOMIZED CONTROLLED TRIAL	Abstract of full publication identified
Thiruvengadam 2023[695]	An Evaluation of Critical Factors for the Cost-Effectiveness of Real-Time Computer-Aided Detection: Sensitivity and Threshold Analyses Using a Microsimulation Model.	Health economic assessment only
Thomas 2023[696]	Advancing Colorectal Cancer Screening: A Comprehensive Systematic Review of Artificial Intelligence (AI)-Assisted Versus Routine Colonoscopy.	Systematic review used for reference checking
Tiankanon 2023[697]	THE IMPROVEMENT ON ADENOMA DETECTION RATE AND OTHER SECONDARY INDICATORS OF THE TWO REAL-TIME ARTIFICIAL INTELLIGENCES IN HIGH ADENOMA DETECTORS: A RANDOMIZED MUTI-CENTER TRIAL	Abstract of full publication identified
Tolosa 2023[698]	REDUCTION OF ADENOMA MISS RATE WITH ARTIFICIAL INTELLIGENCE (AI): A META-ANALYSIS OF RANDOMIZED TANDEM TRIALS OF AI-ASSISTED COLONOSCOPY	Abstract of systematic review
UMIN000013917 2014[699]	Prospective study for endocytoscopy-based computer-aided diagnosis system for small colorectal lesions	Trial record only

UMIN000044031 2021[700]	Evaluation of the impact of CAD EYE on the quality of colonoscopy and the learning curve of gastroenterology fellows	Trial record only
UMIN000044748 2021[701]	Artificial Intelligence in Colonoscopy for Cancer Prevention -a Randomized Health Service Implementation Trial-	Trial record only
UMIN000046361 2021[702]	A randomized control trial of adenoma detection rate in artificial intelligence-assisted colonoscopy using linked color imaging	Trial record only
UMIN000046502 2022[703]	Examination of detection ability of colorectal polyps with AI colonoscopy	Trial record only
UMIN000047666 2022[704]	Veridation research of colonoscopy using artificial intelligence	Trial record only
Vadhwana 2023[705]	The Role of Artificial Intelligence in Prospective Real-Time Histological Prediction of Colorectal Lesions during Colonoscopy: A Systematic Review and Meta-Analysis.	Systematic review used for reference checking
van der Zander 2022[706]	REAL-TIME CLASSIFICATION OF COLORECTAL POLYPS USING ARTIFICIAL INTELLIGENCE - A PROSPECTIVE PILOT STUDY COMPARING TWO COMPUTER-AIDED DIAGNOSIS SYSTEMS AND ONE EXPERT ENDOSCOPIST	Abstract only and have full texts covering outcomes
Van Langendonc 2021[707]	Computer aided detection (CADE) in colonoscopy: an end-user experience using two systems	Abstract only and have full texts covering outcomes

Vinsard 2025[708]	IMPACT OF ARTIFICIAL INTELLIGENCE ON RIGHT COLONIC ADENOMA MISS RATE: A PRAGMATIC QUASI-RANDOMIZED TRIAL	Unnamed intervention
Wadhwa 2023[709]	Diagnostic Accuracy of Artificial Intelligence in Classification of Colonic Polyps - A Real World Prospective Study	Abstract only and have full texts covering outcomes
Wallace 2022[710]	IMPACT OF ARTIFICIAL INTELLIGENCE ON MISS RATE OF COLORECTAL NEOPLASIA: A RANDOMIZED TANDEM CLINICAL TRIAL	Unnamed intervention
Wang 2021[711]	Artificial intelligence-assisted detection and classification of colorectal polyps under colonoscopy: A systematic review and meta-analysis	Systematic review used for reference checking
Wang 2022[712]	Artificial Intelligence - based Colorectal Polyp Diagnostic System Can Increase the Detection Rate of Polyps: A Prospective Randomized Controlled Study	Full text not retrieved
Wang 2018[713]	Assistance of a real-time automatic colon polyp detection system increases polyp and adenoma detection during colonoscopy: A prospective randomized controlled study	Unnamed intervention
Wang 2018[714]	Automatic polyp detection during colonoscopy increases adenoma detection: An interim analysis of a prospective randomized control study	Unnamed intervention
Wang 2024[715]	A retrospective study of computer-aided detection system for detection improvement of adenomas	Full text not retrieved

Wang 2019[716]	Colonoscopy with embedded deep learning computer-aided detection system improves adenoma detection without increasing physician fatigue: A prospective randomized study	Abstract only and have full texts covering outcomes
Wang 2020[717]	859 COMPUTER-AIDED-DETECTION EMBEDDED COLONOSCOPY VERSUS ROUTINE COLONOSCOPY: A PROSPECTIVE, RANDOMIZED TANDEM TRIAL	Abstract of full publication identified
Wang 2021[718]	Artificial intelligence-assisted detection and classification of colorectal polyps under colonoscopy: a systematic review and meta-analysis.	Systematic review used for reference checking
Wang 2024[719]	ENDOANGEL improves detection of missed colorectal adenomas in second colonoscopy: A retrospective study.	Non-randomised study and outcomes covered by randomised trials
Wang 2024[720]	AI Assisted Colonoscopy Improves Polyp Detection in Obese Patients	Unnamed intervention
Wang 2024[721]	Artificial Intelligence-Assisted Colonoscopy Improves PDR in Patients With IBD: A Cohort Study in a High Volume Center	Unnamed intervention
Warman 2024[722]	Non-Neoplastic Polyp Detection Using AI, GI Genius, With Experienced Endoscopist - Pilot Study	Non-randomised study and outcomes covered by randomised trials
Wei 2023[723]	Artificial Intelligence-Assisted Colonoscopy in Real World Clinical Practice: A Systematic Review and Meta-Analysis	Abstract of systematic review

Wei 2023[724]	EVALUATION OF ARTIFICIAL INTELLIGENCE ENABLED COMPUTER AIDED DETECTION ASSISTANCE IN DETECTING COLON POLYPS IN THE COMMUNITY (AI-SEE): A MULTICENTER RANDOMIZED CLINICAL TRIAL	Technology not relevant to review
Wei 2024[725]	Artificial Intelligence-Assisted Colonoscopy in Real-World Clinical Practice: A Systematic Review and Meta-Analysis.	Systematic review used for reference checking
Wenderott 2024[726]	Effects of artificial intelligence implementation on efficiency in medical imaging-a systematic literature review and meta-analysis	Systematic review used for reference checking
Wong 2021[727]	The study on artificial intelligence (AI) colonoscopy in affecting the rate of polyp detection in colonoscopy - A single center retrospective study	Abstract of full publication identified
Wong 2022[728]	The study on artificial intelligence (AI) colonoscopy in affecting the rate of polyp detection in colonoscopy: A single centre retrospective study	Non-randomised study and outcomes covered by randomised trials
Wong 2022[729]	The study on artificial intelligence (AI) colonoscopy in affecting the rate of polyp detection in colonoscopy - a single center retrospective study	Abstract of full publication identified
Wong 2021[730]	The study on artificial intelligence (ai) colonoscopy in affecting the rate of polyp detection in colonoscopy - a single-center retrospective study	Abstract of full publication identified
Wu 2023[731]	Efficacy of Water Exchange vs Computer-Aided Detection: A Bayesian Network Meta-Analysis of Randomized Controlled Trials	Abstract of systematic review

Xu 2023[732]	Implementation of Artificial Intelligence Device for Polyp Detection During Colonoscopy at an Academic County Hospital System	Non-randomised study and outcomes covered by randomised trials
Xu 2025[733]	Artificial intelligence system improves the quality of digestive endoscopy: A prospective pretest and post-test single-center clinical trial	Non-colonoscopy population
Xu 2021[734]	Artificial intelligence-assisted colonoscopy: A prospective, multicenter, randomized controlled trial of polyp detection.	Technology not relevant to review
Xu 2021[735]	Comparison of diagnostic performance between convolutional neural networks and human endoscopists for diagnosis of colorectal polyp: A systematic review and meta-analysis.	Abstract of systematic review
Yamada 2019[736]	Development of a real-time endoscopic image diagnosis support system using deep learning technology in colonoscopy.	*Ex vivo* application of technology. As of February 2025, WISE VISION® not available to the NHS (see Section 1.3.1 of the main report)
Yamada 2021[737]	Detection of flat colorectal neoplasia by artificial intelligence: A systematic review.	Systematic review used for reference checking
Yamaguchi 2023[738]	EVALUATION OF THE IMPACT OF AN AI-AIDED ENDOSCOPIC DIAGNOSIS SYSTEM ON IMPROVING ENDOSCOPY QUALITY AND INCREASING THE LEARNING CURVE FOR BEGINNING COLONOSCOPY TRAINEES: A PROSPECTIVE RANDOMIZED MULTI-CENTER STUDY	Abstract of full publication identified

Yang 2022[739]	Clinical application and diagnostic accuracy of artificial intelligence in colonoscopy for inflammatory bowel disease: systematic review.	Systematic review used for reference checking
Yi 2024[740]	REAL-TIME USE OF ARTIFICIAL INTELLIGENCE IN CHARACTERIZATION OF DIMINUTIVE POLYPS DURING COLONOSCOPY: A SYSTEMATIC REVIEW AND META-ANALYSIS	Abstract of systematic review
Yu 2019[741]	Improved adenoma detection with ENDOANGEL: A randomized controlled trial	Abstract only and have full texts covering outcomes
Yu 2024[742]	ASSESSMENT OF THE ROLE OF FALSE-POSITIVE ALERTS IN COMPUTER-AIDED POLYP DETECTION FOR ASSISTANCE CAPABILITIES: A SECONDARY ANALYSIS OF RANDOMIZED CLINICAL TRIAL	Limited usable data/no relevant outcomes
Zha 2024[743]	Diagnostic Accuracy of Artificial Intelligence in Endoscopy: Umbrella Review.	Systematic review used for reference checking
Zhang 2021[744]	An artificial intelligence-based quality improvement system significantly improved the efficacy of computer-aided detection system in colonoscopy: A 2*2 factorial analysis	Abstract of full publication identified
Zhang 2024[745]	Assessment of the role of false-positive alerts in computer-aided polyp detection for assistance capabilities.	Limited usable data/no relevant outcomes

Zhang 2021[746]	Artificial Intelligence-Aided Colonoscopy for Polyp Detection: A Systematic Review and Meta-Analysis of Randomized Clinical Trials.	Systematic review used for reference checking
Zhang 2025[747]	The Effect of Computer-Aided Device on Adenoma Detection Rate in Different Implement Scenarios: A Real-World Study	Technology not relevant to review
Zhao 2025[748]	Artificial intelligence in colorectal sessile serrated lesion: recent progress	Systematic review used for reference checking
Zhao 2021[749]	Influence of artificial intelligence on colonoscopy in different examination periods	Unnamed intervention
Zhao 2022[750]	CLINICAL STUDY OF ARTIFICIAL INTELLIGENCE ASSISTED COLONOSCOPY IN DETECTING COLORECTAL POLYPS	Unnamed intervention
Zhao 2021[751]	Establishment and validation of a computer-assisted colonic polyp localization system based on deep learning.	Technology not relevant to review
Zhou 2019[752]	A REAL-TIME AUTOMATIC DEEP LEARNING POLYP DETECTION SYSTEM INCREASES POLYP AND ADENOMA DETECTION DURING COLONOSCOPY: A PROSPECTIVE DOUBLE-BLIND RANDOMIZED STUDY	Unnamed intervention
Zippelius 2021[753]	Prospective evaluation of a new artificial intelligence system for detection of colonpolyps	Abstract only and have full texts covering outcomes

Zippelius 2022[754]	Diagnostic accuracy of a novel artificial intelligence system for adenoma detection in daily practice: a prospective nonrandomized comparative study.	Full text not retrieved

2.2 Studies included in NICE assessment but not covered in this publication

Supplementary Table 32. Table of studies included in NICE assessment but not covered in this publication.

Study	Title	Reason for exclusion
Li 2023 – CAD EYE®[755]	Real-World Validation of a Computer-Aided Diagnosis System for Prediction of Polyp Histology in Colonoscopy: A Prospective Multicenter Study.	Autonomous CADx use – not prioritised for this publication
Picardo 2023 – CAD EYE®[756]	PP-495 Evaluation of the artificial intelligencesystem CAD-EYE to characterize lesions in inflammatory bowel disease surveillance.	Autonomous CADx use – not prioritised for this publication
Zavyalov 2024 – CAD EYE®[757]	CAD EYE for real-time detection and differentiation of colorectal lesions.	Autonomous CADx use – not prioritised for this publication
Odin Vision 2024 CADDIE CSR[758] – CADDIE™	CSR for CADDIE trial for the CADDIE™ technology.	Data provided confidentially as part of NICE assessment

Odin Vision 2024 EAGLE CSR[759] – CADDIE™	CSR for EAGLE trial for the CADDIE™ technology.	Data provided confidentially as part of NICE assessment
EMIS™ trial (NCT05241210) 2025[760, 761]	Preliminary data for NCT05241210 trial for EMIS™.	Data provided confidentially as part of NICE assessment
Baumer 2023[762] – GI Genius™	Accuracy of polyp characterization by artificial intelligence and endoscopists: a prospective, non-randomized study in a tertiary endoscopy center.	Autonomous CADx use – not prioritised for this publication
Koh 2024[763] – GI Genius™	Real-World Assessment of the Efficacy of Computer-Assisted Diagnosis in Colonoscopy: A Single Institution Cohort Study in Singapore.	Autonomous CADx use – not prioritised for this publication
Ladabaum 2023[764] – GI Genius™	BELIEFS AND ATTITUDES ABOUT ARTIFICIAL INTELLIGENCE (AI) AMONG COLONOSCOPIST PARTICIPANTS IN A PRAGMATIC IMPLEMENTATION TRIAL OF COMPUTER-AIDED DETECTION (CADE) OF POLYPS THAT DID NOT REPLICATE THE POSITIVE RESULTS OF RANDOMIZED TRIALS.	Only reports outcomes not prioritised for inclusion in this publication
NAIAD trial[765] – GI Genius™	Preliminary data for NAIAD trial for GI Genius™.	Data provided confidentially as part of NICE assessment
Nehme 2023[766] – GI Genius™	Performance and attitudes toward real-time computer-aided polyp detection during colonoscopy in a large tertiary referral center in the United States.	Only reports outcomes not prioritised for inclusion in this publication

Olabintan 2025[767] – GI Genius™	UK ENDOSCOPISTS' PERSPECTIVES ON ARTIFICIAL INTELLIGENCE IN ENHANCING POLYP MANAGEMENT AND ENDOSCOPIC PRACTICE.	Only reports outcomes not prioritised for inclusion in this publication
Pinto 2022[768] – GI Genius™	ACUITY OF ARTIFICIAL INTELLIGENCE ASSISTED COLONOSCOPY IN LYNCH SYNDROME PATIENTS.	Only reports outcomes not prioritised for inclusion in this publication
Seager 2024[769] – GI Genius™	USERS' OPINIONS & EXPERIENCES OF A COMPUTERAIDED DETECTION DEVICE FOR COLONOSCOPY AND POTENTIAL EFFECTS ON ADOPTION AND IMPLEMENTATION.	Only reports outcomes not prioritised for inclusion in this publication
Anderson 2024[770] – clinician thoughts on using three unnamed AI colonoscopy systems	ARTIFICIAL INTELLIGENCE IN COLONOSCOPY: REAL WORLD EXPERIENCE FROM THE SOUTHWEST ENDOSCOPY GROUP.	Only reports outcomes not prioritised for inclusion in this publication
Burton 2025[771] – patient perceptions on AI use in colonoscopy	Patient Perspective of Use of Artificial Intelligence During Colonoscopy.	Only reports outcomes not prioritised for inclusion in this publication
Magahis 2023[772] – clinician thoughts on AI in colonoscopy	Impact of Artificial Intelligence on Gastroenterology Training and Education: A Survey of Fellows' Perspectives.	Only reports outcomes not prioritised for inclusion in this publication
Schmidt 2025[773] – patient perceptions on AI use in colonoscopy	Understanding Patients' Current Acceptability of Artificial Intelligence During Colonoscopy for Polyp Detection: A Single-Center Study.	Only reports outcomes not prioritised for inclusion in this publication

3 Risk of bias assessments

3.1 Publication bias

A formal statistical assessment of publication bias was not performed for most meta-analyses in this assessment given it is recommended that tests for funnel plot asymmetry, used to assess publication bias, are not performed unless there are at least 10 meta-analysed studies (see Section 13.3.5.4 of the Cochrane Handbook for Systematic Reviews of Interventions version 6.5),[774] which was only the case for the adenoma detection rate (ADR) and adenomas per colonoscopy (APC; when analysed as an incidence rate ratio [IRR]) analyses for the CAD EYE® intervention; however, the denominator for the APC IRR analysis was number of polyps rather than number of patients, so the funnel plot for this outcome has not been reviewed for evidence of publication bias. There are 12 studies included in the CAD EYE® ADR meta-analysis. The funnel plot appears to be largely symmetrical with the exception of study spread in the lower section of the graph, as there is some representation on the lower right-hand side of the plot but not the lower left-hand side (Supplementary Figure 47). However, the researchers consider this insufficient to make conclusions about whether or not publication bias is present and has commented further on the potential for publication bias based on methods used and trial records identified below.

Supplementary Figure 47. Funnel plot for ADR outcome in CAD EYE® vs standard colonoscopy meta-analysis

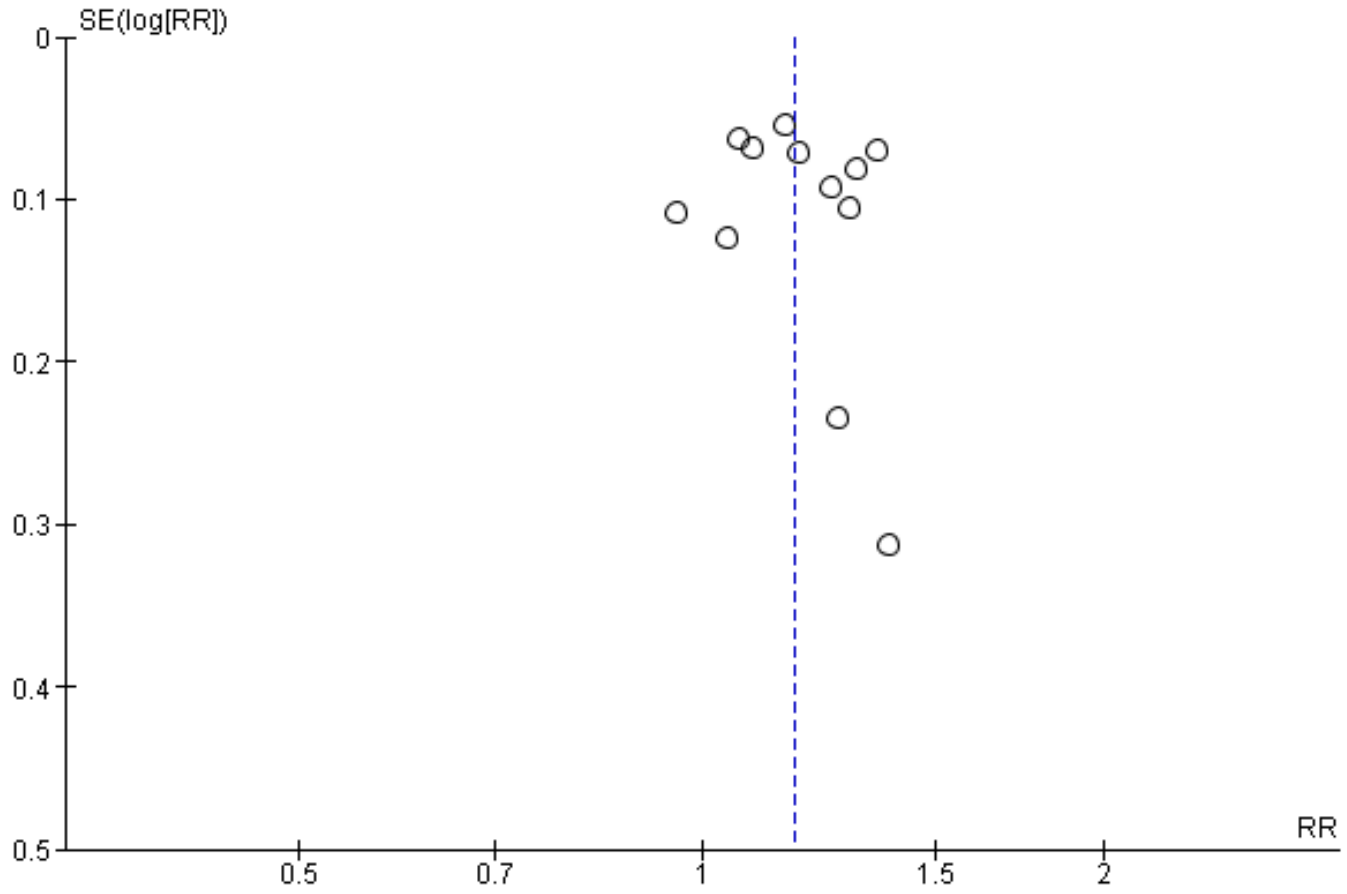

Abbreviations: ADR, adenoma detection rate.

The exclusion of data from conference abstracts unless no other data were available from full text publications for a particular outcome and intervention may increase the risk of publication bias to some extent; however, it considers this to be a necessary limitation in this assessment given the time constraints on the project and given data from abstracts are associated with fewer details in terms of methodology and would be considered to be at a higher risk of bias in this review.

There may be some concerns about publication bias for this topic area based on a review of clinical trial records where a publication has not yet been identified. Of those that were due to complete by 2022 at the latest, at least 16 trial records that did not appear to have a publication associated with them as of January 2025 were identified; however, there could be other reasons for this, such as trials being discontinued or trial numbers not being cited in publications. Despite this, the researchers consider it possible that publication bias is a risk associated with evidence in this topic area.

3.2 Cochrane Risk of Bias tool Version 2 tables – randomised controlled trials (RCTs)

Supplementary Table 33. Risk of bias assessment conducted at the study level for RCTs included in the SLR – Cochrane Risk of Bias 2 tool

Study	Domain 1 – randomisation process	Domain 2 – deviations from intended interventions	Domain 3 – missing outcome data	Domain 4 – measurement of the outcome	Domain 5 – selection of the reported result	Overall bias	Comments
**CAD EYE®**							
Alali 2025[2]	**Some concerns** Computer-based randomisation with sealed envelopes for concealment. Mostly well-balanced but larger differences for gender and valve intubation, possibly due to small sample size.	**Low** Patients blinded, endoscopists aware of treatments but deviations from intended intervention unlikely. ITT analysis.	**Low** All randomised participants were analysed.	**Some concerns** Endoscopists aware of intervention possibly introducing some operator bias, but pathologists blinded.	**Some concerns** No statistical analysis plan identified but no major concerns about selective reporting.	**Some concerns**	Risk of bias mostly due to lack of endoscopist blinding (although unavoidable for this type of intervention) and larger imbalances at baseline for gender and valve intubation.
Aniwan 2023[3]	**Low** Online randomisation, sealed envelopes for concealment. No concerns about baseline differences.	**Low** Patient and endoscopists aware of treatment but deviations from intended intervention unlikely. ITT analysis.	**Low** All randomised participants were analysed.	**Some concerns** Endoscopists aware of intervention possibly introducing some operator bias, but pathologists blinded.	**Some concerns** No statistical analysis plan identified but no major concerns about selective reporting.	**Some concerns**	Risk of bias mostly due to lack of endoscopist blinding (although unavoidable for this type of intervention).
Desai 2024[4]	**Low** Computer-based randomisation, unclear concealment. No concerns about baseline differences.	**Some concerns** Unclear if patients blinded, endoscopists not blinded. Some withdrawn if insufficient	**Some concerns** ~9% of randomised participants were not analysed, similar in both groups.	**Some concerns** Endoscopists aware of intervention possibly introducing some operator bias, unclear if pathologists blinded.	**Some concerns** No statistical analysis plan identified but no major concerns	**Some concerns**	Risk of bias mostly due to lack of endoscopist blinding (although unavoidable for this type of intervention).

		colonoscopy time. mITT analysis.			about selective reporting.		
Djinbachian 2024[5]	**Low** Electronic randomisation process, with electronic concealment. No concerns about baseline differences.	**Low** Unclear if patients blinded, endoscopists not blinded but deviations from intended intervention unlikely. ITT analysis.	**Low** All randomised participants were analysed.	**Some concerns** Endoscopists aware of intervention possibly introducing some operator bias, unclear if pathologists blinded.	**Some concerns** No statistical analysis plan identified but no major concerns about selective reporting.	**Some concerns**	Risk of bias mostly due to lack of endoscopist blinding (although unavoidable for this type of intervention).
Hiratsuka 2025[6]	**Low** Random allocation method with minimisation described, no details on concealment. No concerns about baseline differences.	**Low** Unclear if patients blinded, endoscopists not blinded but deviations from intended intervention unlikely. mITT analysis.	**Low** ~6% of randomised participants were not analysed, similar in both groups.	**Some concerns** Endoscopists aware of intervention possibly introducing some operator bias, unclear if pathologists blinded.	**Some concerns** No statistical analysis plan identified but no major concerns about selective reporting.	**Some concerns**	Risk of bias mostly due to lack of endoscopist blinding (although unavoidable for this type of intervention).
Huneburg 2023[7] – CADLY trial	**Some concerns** Online randomisation, sealed envelopes for concealment. Imbalance in proportion with prior extra-colonic cancer (higher in AI group; 38.0 vs 19.6%) but no concerns for other variables.	**Low** Patient and endoscopists aware of treatment but deviations from intended intervention unlikely. mITT analysis.	**Low** Only 5 patients (~5.0% randomised) excluded from analysis, similar in both groups .	**Some concerns** Endoscopists aware of intervention possibly introducing some operator bias, but pathologists blinded.	**Some concerns** No statistical analysis plan identified but no major concerns about selective reporting.	**Some concerns**	Risk of bias mostly due to lack of endoscopist blinding (although unavoidable for this type of intervention) and imbalance in proportions with prior extra-colonic cancer.
Miyaguchi 2024[8]	**Low** Online randomisation, sealed envelopes for concealment. No	**Low** Unclear if patients blinded, endoscopists not blinded. No concerns about	**Low** All randomised participants were analysed.	**Some concerns** Endoscopists aware of intervention possibly introducing some	**Some concerns** No statistical analysis plan identified but no major concerns	**Some concerns**	Risk of bias mostly due to lack of endoscopist blinding (although unavoidable for

	concerns about baseline differences.	deviations from intended intervention. ITT analysis.		operator bias, unclear if pathologists blinded.	about selective reporting.		this type of intervention).
Nakashima 2023[9]	**Low** Computerised randomisation, allocation likely concealed by system. No concerns about baseline differences.	**Low** Patients blinded, endoscopists not blinded. No concerns about deviations from intended interventions. Unclear but possibly ITT analysis.	**Some concerns** Assume all randomised but unclear as lack of information about missing data in the published paper.	**Some concerns** Endoscopists aware of intervention possibly introducing some operator bias, but pathologists blinded.	**Some concerns** No statistical analysis plan identified. Primary outcome aligns with registration but not all secondary outcomes listed there.	**Some concerns**	Risk of bias mostly due to lack of endoscopist blinding (although unavoidable for this type of intervention) and lack of information regarding missing data
Rondonotti 2022[10] – AIFIT trial	**Low** Computerised randomisation, sealed envelopes for concealment. No concerns about baseline differences.	**Low** Patients blinded, endoscopists not blinded. No concerns about deviations from intended interventions. mITT analysis.	**Low** <5% in each group excluded from analysis.	**Some concerns** Endoscopists aware of intervention possibly introducing some operator bias, but pathologists blinded.	**Some concerns** No statistical analysis plan identified but no major concerns about selective reporting.	**Some concerns**	Risk of bias mostly due to lack of endoscopist blinding (although unavoidable for this type of intervention).
Scholer 2024[11a]	**Some concerns** Sealed envelopes used for randomisation. Imbalance in endoscopist experience is a concern (70.0 vs 85.0% experienced)	**Low** Patient and endoscopists aware of treatment. No concerns about deviations from intended interventions. Possibly a mITT analysis.	**High** ~16% those randomised excluded, including some before starting the procedure. Similar in both groups, but unclear breakdown for CAD EYE® specific groups.	**Some concerns** Use of other lighting options encouraged in the AI group. Endoscopists aware of intervention possibly introducing some operator bias, but pathologists blinded.	**Some concerns** No statistical analysis plan identified. Limited outcomes reported for the group specifically receiving CAD EYE®.	**High**	Risk of bias mostly due to imbalance in endoscopist experience, missing data, and lack of endoscopist blinding (although unavoidable for this type of intervention).
Tiankanon 2024[12]	**Low** Computerised randomisation,	**Low** Patients and endoscopists not	**Low** All randomised participants appear	**Some concerns** Endoscopists aware of intervention possibly	**Some concerns** No statistical analysis plan	**Some concerns**	Risk of bias mostly due to lack of endoscopist

	allocation likely concealed by system. No concerns about baseline differences.	blinded. No information about deviations from intended interventions. ITT analysis.	to have been analysed.	introducing some operator bias, but pathologists blinded.	identified but no major concerns about selective reporting.		blinding (although unavoidable for this type of intervention).
Yamaguchi 2024[13]	**Low** Computerised randomisation, allocation concealed through personnel not involved in other trial processes. No concerns about baseline differences.	**Low** Patients blinded, endoscopists not blinded. No concerns about deviations from intended interventions. mITT analysis.	**Low** Only ~6% of randomised patients excluded from analysis	**Some concerns** Endoscopists aware of intervention possibly introducing some operator bias, unclear if pathologists blinded.	**Some concerns** No statistical analysis plan identified but no major concerns about selective reporting.	**Some concerns**	Risk of bias mostly due to lack of endoscopist blinding (although unavoidable for this type of intervention).
Zimmermann-Fraedrich 2025[14]	**Low** Described as randomised with sealed envelopes for concealment. No concerns about baseline differences.	**Low** Unclear if patients blinded, endoscopists not blinded but deviations from intended intervention unlikely. ITT analysis.	**Low** All randomised participants were analysed.	**Some concerns** Endoscopists aware of intervention possibly introducing some operator bias, unclear if pathologists blinded.	**Some concerns** No statistical analysis plan identified but no major concerns about selective reporting.	**Some concerns**	Risk of bias mostly due to lack of endoscopist blinding (although unavoidable for this type of intervention).
**Discovery™**							
Maas 2024 - Discovery™[15] – DISCOVERY II trial	**Low** Online randomisation, allocation likely concealed by system. No concerns about baseline differences.	**Low** Patient and endoscopists aware of treatment. No concerns about deviations from intended interventions. ITT/mITT analyses.	**Some concerns** ~15% excluded from mITT analyses, similar in both groups	**Some concerns** Endoscopists aware of intervention possibly introducing some operator bias, unclear if pathologists blinded.	**Some concerns** No statistical analysis plan identified but no major concerns about selective reporting.	**Some concerns**	Risk of bias mostly due to concerns about missing data and lack of endoscopist blinding (although unavoidable for this type of intervention).
**ENDO-AID™**							

Gimeno-Garcia 2023[16]	**Low** Computerised randomisation, sealed envelopes for concealment. No concerns about baseline differences.	**Low** Patients blinded, endoscopists aware of treatment. No concerns about deviations from intended interventions. ITT analysis for primary outcome.	**Low** All randomised participants analysed in ITT analysis.	**Some concerns** More advanced processor used in AI group. Endoscopists aware of intervention possibly introducing some operator bias, but pathologists blinded.	**Some concerns** No statistical analysis plan identified but no major concerns about selective reporting.	**Some concerns**	Risk of bias mostly due to concerns about difference in processor used between groups and lack of endoscopist blinding (although unavoidable for this type of intervention).
Lau 2024[17] – ENDOAIDTRAIN trial	**Low** Computerised randomisation, unclear method of concealment. No concerns about baseline differences.	**Low** Patients blinded, endoscopists aware of treatment. No concerns about deviations from intended interventions. mITT analysis.	**Some concerns** ~11% randomised participants excluded from mITT analyses, similar in both groups	**Some concerns** Endoscopists aware of intervention possibly introducing some operator bias, but pathologists blinded.	**Some concerns** No statistical analysis plan identified but no major concerns about selective reporting.	**Some concerns**	Risk of bias mostly due to concerns about lack of endoscopist blinding (although unavoidable for this type of intervention).
Lui 2024[18]	**Some concerns** Computer randomisation, research assistant not involved in other trial procedures maintained schedule. Sealed envelopes used. Small imbalance in endoscopist experience between groups.	**Low** Patients blinded, endoscopists aware of treatment. No concerns about deviations from intended interventions. ITT analysis.	**Low** All randomised participants analysed	**Some concerns** Endoscopists aware of intervention possibly introducing some operator bias, but pathologists blinded.	**Some concerns** No statistical analysis plan identified but no major concerns about selective reporting.	**Some concerns**	Risk of bias mostly due to concerns about lack of endoscopist blinding (although unavoidable for this type of intervention).
Spada 2025[19]	**Low**	**Low**	**Low**	**Some concerns**	**Some concerns**	**Some concerns**	Risk of bias mostly due to concerns

	Computer-based randomisation, no mention of concealment. No concerns about baseline differences.	Patients and endoscopists aware of intervention but no concerns about any deviations from interventions. ITT.	All randomised participants analysed	Endoscopists aware of intervention possibly introducing some operator bias, but pathologists blinded.	No statistical analysis plan identified but no major concerns about selective reporting.		about lack of endoscopist blinding (although unavoidable for this type of intervention).
Vilkoite 2023[20]	**Some concerns** No information on methods of randomisation provided. Very limited number of baseline characteristics reported to assess similarity between groups.	**Low** Patients blinded, endoscopists aware of treatment. No information on deviations from intended interventions, possibly mITT analysis.	**High** No information provided on numbers excluded from analyses and missing data.	**Some concerns** Endoscopists aware of intervention possibly introducing some operator bias, but pathologists blinded.	**Some concerns** No statistical analysis plan identified. Very few outcomes reported.	**High**	Risk of bias mostly due to very limited reporting of methodological details, missing data and patient characteristics, and lack of endoscopist blinding (although unavoidable for this type of intervention).
**ENDOANGEL®**							
Gong 2020[21]	**Some concerns** Computerised randomisation, method of concealment unclear. Some imbalance noted for sex.	**Low** Patients blinded, endoscopists aware of treatment. No concerns about deviations from intended interventions. ITT analysis.	**Low** All randomised participants analysed.	**High** Concerns about outcome measurement – only suspected adenomas removed and sent for histology rather than all/most polyps, difference vs other studies in the area. Endoscopists aware of intervention possibly introducing some	**Low** Trial protocol available and no major concerns about selective reporting.	**High**	Risk of bias mostly due to concerns about outcome measurement and lack of endoscopist blinding (although unavoidable for this type of intervention).

				operator bias, but pathologists blinded.			
Yao 2022[22]	**Low** Computerised randomisation, unclear method of concealment. No concerns about baseline differences.	**Low** Patients blinded, endoscopists aware of treatment. No concerns about deviations from intended interventions. mITT analysis.	**Low** Only ~4% excluded from mITT analyses, similar in both groups	**Some concerns** Endoscopists aware of intervention possibly introducing some operator bias, unclear if pathologists blinded.	**Some concerns** No statistical analysis plan identified. No major concerns about selective reporting.	**Some concerns**	Risk of bias mostly due to lack of endoscopist blinding (although unavoidable for this type of intervention).
Yao 2024[23]	**Low** Computerised randomisation, unclear method of concealment. No concerns about baseline differences.	**Low** Patients blinded, endoscopists aware of treatment. No concerns about deviations from intended interventions. mITT analysis.	**Low** Only ~2% excluded from mITT analyses, similar across groups	**Some concerns** Endoscopists aware of intervention possibly introducing some operator bias, unclear if pathologists blinded.	**Low** Trial protocol reviewed and no major concerns about selective reporting.	**Some concerns**	Risk of bias mostly due to lack of endoscopist blinding (although unavoidable for this type of intervention).
Zhang 2023[24]	**Some concerns** Computerised randomisation, unclear method of concealment. Limited reporting of baseline characteristics for assessment of similarity.	**Low** Patients blinded, endoscopists aware of treatment. No concerns about deviations from intended interventions. mITT analysis.	**Low** Only ~5% excluded from mITT analyses, similar in both groups	**High** Outcome confirmed by expert endoscopists whereas histology would be more accurate. Endoscopists aware of intervention possibly introducing some operator bias. No mention of pathologist involvement.	**Some concerns** No statistical analysis plan identified. Some concerns about missing overall PDR but may just be an omission.	**High**	Risk of bias mostly due to concerns about outcome measurement and lack of endoscopist blinding (although unavoidable for this type of intervention).
**EndoScreener®**							
Glissen Brown 2022[25]	**Some concerns** Computerised randomisation, sealed envelopes	**Low** Patients blinded, endoscopists aware of treatment. No concerns	**Low** Only ~4% excluded from mITT	**Some concerns** Endoscopists aware of intervention possibly introducing some	**Some concerns** No statistical analysis plan identified. No major	**Some concerns**	Risk of bias mostly due to some imbalances in baseline

	used for concealment. Large imbalance in sex and smaller for ethnicity.	about deviations from intended interventions. mITT analysis.	analyses, similar in both groups	operator bias, but pathologists blinded.	concerns about selective reporting.		characteristics and lack of endoscopist blinding (although unavoidable for this type of intervention).
Liu 2020[26]	**Some concerns** Digital random number generator just prior to colonoscopy procedure, unclear method of concealment. No concerns about baseline differences.	**Low** Patients blinded, endoscopists aware of treatment. No concerns about deviations from intended interventions. mITT analysis.	**Low** Only ~6% excluded from mITT analyses, similar in both groups	**Some concerns** Endoscopists aware of intervention possibly introducing some operator bias, but pathologists blinded.	**Low** Trial protocol available and no concerns about selective reporting.	**Some concerns**	Risk of bias mostly due to lack of endoscopist blinding (although unavoidable for this type of intervention).
Wang 2019[27]	**Some concerns** Digital random number generator just prior to colonoscopy procedure, unclear method of concealment. No concerns about baseline differences.	**Low** Unclear if patients blinded, endoscopists aware of treatment. No concerns about deviations from intended interventions. mITT analysis.	**Low** Only ~6% excluded from mITT analyses, similar in both groups	**Some concerns** Endoscopists aware of intervention possibly introducing some operator bias, unclear if pathologists blinded.	**Some concerns** No statistical analysis plan identified. No major concerns about selective reporting.	**Some concerns**	Risk of bias mostly due to lack of endoscopist blinding (although unavoidable for this type of intervention).
Wang 2020 (effect of a deep…)[28] - CADe-DB trial	**Low** Computerised randomisation, unclear method of concealment. No concerns about baseline differences.	**Low** Patients blinded, endoscopists aware of treatment. No concerns about deviations from intended interventions. Described as per protocol analysis but	**Low** Only ~5% excluded from mITT analyses, similar in both groups	**Some concerns** Endoscopists aware of intervention possibly introducing some operator bias, unclear if pathologists blinded.	**Some concerns** No statistical analysis plan identified. No major concerns about selective reporting.	**Some concerns**	Risk of bias mostly due to lack of endoscopist blinding (although unavoidable for this type of intervention).

		similar exclusions to others described as mITT.					
Wang 2020 (lower adenoma miss…)[29]	**Low** Computerised randomisation, unclear method of concealment. No concerns about baseline differences.	**Low** Patients blinded, endoscopists aware of treatment. No information about deviations from intended interventions. mITT analysis.	**Low** Only ~5% excluded from mITT analyses, similar in both groups	**Some concerns** Endoscopists aware of intervention possibly introducing some operator bias, unclear if pathologists blinded.	**Some concerns** No statistical analysis plan identified. No major concerns about selective reporting.	**Some concerns**	Risk of bias mostly due to lack of endoscopist blinding (although unavoidable for this type of intervention).
Wang 2023[30]	**Some concerns** Digital random number generator just prior to colonoscopy procedure, unclear method of concealment. No concerns about baseline differences.	**Low** Patients blinded, endoscopists aware of treatment. No information about deviations from intended interventions. mITT analysis.	**Low** Only ~4% excluded from mITT analyses, similar in both groups	**Some concerns** Endoscopists aware of intervention possibly introducing some operator bias, unclear if pathologists blinded.	**Some concerns** No statistical analysis plan identified. No major concerns about selective reporting.	**Some concerns**	Risk of bias mostly due to lack of endoscopist blinding (although unavoidable for this type of intervention).
**GI Genius™**							
Ahmad 2023[31] – AI-DETECT trial	**Some concerns** Computerised randomisation, unclear method of concealment. Only limited baseline characteristics reported for assessment of comparability.	**Low** Patients and endoscopists aware of treatment. No information about deviations from intended interventions. ITT analysis.	**Low** All randomised participants analysed	**Some concerns** Endoscopists aware of intervention possibly introducing some operator bias, unclear if pathologists blinded.	**Some concerns** No statistical analysis plan identified. No major concerns about selective reporting.	**Some concerns**	Risk of bias mostly due to lack of endoscopist blinding (although unavoidable for this type of intervention).

Engelke 2023[32]	**High**<br>Randomisation based on alternation not a random sequence. Unclear method of concealment. Concerns about imbalances as medians reported and some imbalances noted (experience of endoscopists and completion of colonoscopies).	**Some concerns**<br>Unclear if patients blinded, endoscopists aware of treatment. No information about deviations from intended interventions. Possibly ITT but limited reporting.	**Some concerns**<br>Possibly all randomised participants analysed but poor reporting of dropouts and exclusions	**Some concerns**<br>Endoscopists aware of intervention possibly introducing some operator bias, unclear if pathologists blinded.	**Some concerns**<br>No statistical analysis plan identified. No major concerns about selective reporting.	**High**	Risk of bias mostly due to concerns about randomisation methods and imbalances, limited reporting of missing data and lack of endoscopist blinding (although unavoidable for this type of intervention).
Karsenti 2023[33] – COLO-Genius trial	**Some concerns**<br>Computerised random number system, method of concealment unclear. Randomisation not performed until colon had been visualised. No concerns about baseline differences.	**Low**<br>Patients blinded, endoscopists aware of treatment. No concerns about deviations from intended interventions. mITT analysis.	**Low**<br>Only ~1% excluded from mITT analyses, similar in both groups	**Some concerns**<br>Endoscopists aware of intervention possibly introducing some operator bias, but pathologists blinded.	**Some concerns**<br>No statistical analysis plan identified. No major concerns about selective reporting.	**Some concerns**	Risk of bias mostly due to lack of endoscopist blinding (although unavoidable for this type of intervention).
Lagstrom 2025[34]	**High**<br>Quasi-randomisation with alternation every 2 weeks, no mention of concealment. Some larger differences for diagnostic indication, gender, comorbidity,	**Some concerns**<br>Unclear if patients blinded, endoscopists not blinded but deviations from intended intervention unlikely. Per protocol analysis only.	**Some concerns**<br>~10% of randomised participants excluded from per protocol analyses, similar in both groups.	**Some concerns**<br>Endoscopists aware of intervention possibly introducing some operator bias, unclear if pathologists blinded.	**Some concerns**<br>No statistical analysis plan identified. No major concerns about selective reporting.	**High**	Risk of bias mostly due to concerns about randomisation methods and imbalances, patients excluded from per protocol analyses and lack of endoscopist blinding (although

	sedation and diverticulosis).						unavoidable for this type of intervention).
Mangas-Sanjuan 2023[36] – CADILLAC trial	**Low** List of random numbers used for randomisation; electronic system used for concealment. No concerns about baseline differences.	**Low** Patients blinded, endoscopists aware of treatment. No information about deviations from intended interventions. mITT analysis.	**Low** Only ~5% excluded from mITT analyses, similar in both groups	**Some concerns** Endoscopists aware of intervention possibly introducing some operator bias, but pathologists blinded.	**Some concerns** Some possible inconsistencies compared with statistical analysis plan but no major concerns.	**Some concerns**	Risk of bias mostly due to possible inconsistencies with statistical analysis plan and lack of endoscopist blinding (although unavoidable for this type of intervention).
Ortiz 2024[37] – TIMELY trial	**Low** List of random numbers used for randomisation; electronic system used for concealment. No concerns about baseline differences.	**Low** Patients blinded, endoscopists aware of treatment. No information about deviations from intended interventions. ITT analysis.	**Low** All randomised participants analysed.	**Some concerns** Endoscopists aware of intervention possibly introducing some operator bias, but pathologists blinded.	**Low** No major concerns compared to protocol outlined in supplementary materials.	**Some concerns**	Risk of bias mostly due to lack of endoscopist blinding (although unavoidable for this type of intervention).
Repici 2020[38] – AID trial	**Low** List of random numbers used for randomisation; unclear method of concealment. No concerns about baseline differences.	**Low** Patients blinded, endoscopists aware of treatment. No information about deviations from intended interventions. mITT/ITT analysis.	**Low** Only ~2% excluded from analyses, similar in both groups	**Some concerns** Endoscopists aware of intervention possibly introducing some operator bias, but pathologists blinded.	**Some concerns** No statistical analysis plan identified. Some minor concerns given inconsistencies within paper in terms of whether those with inadequate bowel preparation	**Some concerns**	Risk of bias mostly due to uncertainty about exclusions from analysis and whether prespecified, and lack of endoscopist blinding (although unavoidable for this type of intervention).

					excluded from analysis or not.		
Repici 2022[39] – AID2 trial	**Low** List of random numbers used for randomisation; unclear method of concealment. No concerns about baseline differences.	**Low** Unclear if patients blinded, endoscopists aware of treatment. No concerns about deviations from intended interventions. mITT analysis.	**Low** Only ~3% excluded from analyses, similar in both groups	**Some concerns** Endoscopists aware of intervention possibly introducing some operator bias, unclear if pathologists blinded.	**Some concerns** No statistical analysis plan identified. Some minor concerns about whether mITT analysis with exclusion of inadequate bowel preparation prespecified given description as ITT analysis in the paper.	**Some concerns**	Risk of bias mostly due to uncertainty about exclusions from analysis and whether prespecified, and lack of endoscopist blinding (although unavoidable for this type of intervention).
Scholer 2024[11b]	**Some concerns** Sealed envelopes used for randomisation. Imbalance in endoscopist experience is a concern (70.0 vs 85.0% experienced)	**Low** Patients and endoscopists aware of treatment. No concerns about deviations from intended interventions. Possibly a mITT analysis.	**High** ~16% those randomised excluded, including some before starting the procedure. Similar in both groups, but unclear breakdown for CAD EYE® specific groups.	**Some concerns** Use of other lighting options encouraged in the AI group. Endoscopists aware of intervention possibly introducing some operator bias, but pathologists blinded.	**Some concerns** No statistical analysis plan identified. Limited outcomes reported for the group specifically receiving GI Genius™	**High**	Risk of bias mostly due to imbalance in endoscopist experience, missing data, and lack of endoscopist blinding (although unavoidable for this type of intervention).
Seager 2024[40] – COLO-DETECT trial	**Low** Web-based randomisation system which likely retained concealment. Randomisation just before procedure. No	**Low** Patients and endoscopists aware of treatment. No concerns information about deviations from intended interventions.	**Some concerns** Only 7% with missing data required imputation. Differs slightly between groups. Sensitivity	**Some concerns** Endoscopists aware of intervention possibly introducing some operator bias, but pathologists blinded.	**Low** Statistical analysis plan available and no major concerns about selective reporting.	**Some concerns**	Risk of bias mostly due to concerns about missing data and lack of endoscopist blinding (although unavoidable for

	concerns about baseline differences.	ITT analysis with some imputation.	analyses performed to explore limits to some concerns.				this type of intervention).
Thiruvengadam 2024[41]	**Low**<br>Online randomisation system, unclear method of concealment. No concerns about baseline differences.	**Low**<br>Patients blinded, endoscopists aware of treatment. No concerns about deviations from intended interventions. ITT analysis.	**Low**<br>All randomised participants analysed.	**Some concerns**<br>Endoscopists aware of intervention possibly introducing some operator bias, but pathologists blinded.	**Some concerns**<br>No statistical analysis plan identified. No major concerns about selective reporting.	**Some concerns**	Risk of bias mostly due to lack of endoscopist blinding (although unavoidable for this type of intervention).
Wallace 2022[42]	**Low**<br>Online randomisation system, which likely maintained allocation concealment. No concerns about baseline differences.	**Low**<br>Patients blinded, endoscopists aware of treatment. No concerns about deviations from intended interventions. FAS/mITT analysis.	**Some concerns**<br>~8% randomised excluded from analysis, not reported separately to compare between groups	**Some concerns**<br>Endoscopists aware of intervention possibly introducing some operator bias, but pathologists blinded.	**Some concerns**<br>No statistical analysis plan identified. No major concerns about selective reporting.	**Some concerns**	Risk of bias mostly due to lack of endoscopist blinding (although unavoidable for this type of intervention).
**MAGENTIQ-COLO™**							
Maas 2024 – MAGENTIQ-COLO™[43]	**Low**<br>Online randomisation system, which likely maintained allocation concealment. No concerns about baseline differences.	**Low**<br>Unclear if patients blinded, endoscopists aware of treatment. No concerns about deviations from intended interventions. mITT analysis.	**Low**<br>Only ~4% excluded from analyses, similar in both groups	**Some concerns**<br>Endoscopists aware of intervention possibly introducing some operator bias, but pathologists blinded.	**Some concerns**<br>No statistical analysis plan identified. No major concerns about selective reporting	**Some concerns**	Risk of bias mostly due to lack of endoscopist blinding (although unavoidable for this type of intervention).

[a]Risk of bias assessment based on whole trial population rather than just those receiving CAD EYE® for colonoscopy, but similar concerns likely to apply;

[b]Risk of bias assessment based on whole trial population rather than just those receiving GI GENIUS™ for colonoscopy, but similar concerns likely to apply.

Abbreviations: AI, artificial intelligence; ITT, intention to treat; mITT, modified intention to treat; PDR, polyp detection rate; RCT, randomised controlled trial; SLR, systematic literature review.

3.3 QUADAS-2

Supplementary Table 34. Risk of bias assessment conducted at the study level for diagnostic accuracy studies included in the SLR – QUADAS-2

Study	Domain 1 – patient selection					Domain 2 – index test				Domain 3 – reference standard				Domain 4 – flow and timing				
	SQ 1[a]	SQ2[b]	SQ3[c]	RoB[d]	Applicability[e]	SQ 1[f]	SQ2[g]	RoB[d]	Applicability[e]	SQ 1[h]	SQ2[i]	RoB[d]	Applicability[e]	SQ 1[j]	SQ2[k]	SQ3[l]	SQ4[m]	RoB[d]
**CAD EYE®**																		
Djinbachian 2024 – CAD EYE®-assisted optical daignosis[45]	Yes	Yes	Yes	LOW	LOW	Yes	NA	LOW	LOW	Yes	Yes	LOW	LOW	Unclear	Yes	Yes	No	LOW
Rondonotti 2023 – CAD EYE®-assisted optical diagnosis[46] – ABC trial	Yes	Yes	Yes	LOW	LOW	Yes	NA	LOW	LOW	Unclear	Yes	LOW	HIGH[n]	Unclear	Yes	Yes	No	HIGH[o]
Rondonotti 2023 – endoscopist optical diagnosis alone[46] – ABC trial	Yes	Yes	Yes	LOW	LOW	Yes	NA	LOW	LOW	Unclear	Yes	LOW	HIGH[n]	Unclear	Yes	Yes	No	HIGH[o]
Sato 2024 – CAD EYE®-assisted optical diagnosis[47]	Yes	Yes	Yes	LOW	LOW	Yes	NA	LOW	LOW	Unclear	Yes	LOW	HIGH[n]	Unclear	Yes	Yes	No	LOW

Rondonotti 2024 – GI Genius™-assisted optical diagnosis[52]	Hassan 2022 – GI Genius™-assisted optical diagnosis[51] – CHANGE trial	Bernhofer 2025 – GI Genius™-assisted optical diagnosis[50]	**GI Genius™**	Lopez-Serrano 2024 – VCE-assisted classification[49] – CUDISIA trial	Lopez-Serrano 2024 – Discovery™-assisted classification[49] – CUDISIA trial	**Discovery™**	Taghiakbari 2025 – CAD EYE®-assisted optical diagnosis[48]	Sato 2024 – endoscopist optical diagnosis alone[47]
Yes	Unclear	Yes		Yes	Yes		Unclear	Yes
Yes	Yes	Yes		Yes	Yes		Yes	Yes
Yes	Yes	Yes		Yes	Yes		Yes	Yes
LOW	LOW	LOW		LOW	LOW		LOW	LOW
LOW	LOW	LOW		LOW	LOW		LOW	LOW
Yes	Yes	Yes		Yes	Yes		Yes	Yes
NA	NA	NA		NA	NA		NA	NA
LOW	LOW	LOW		LOW	LOW		LOW	LOW
LOW	LOW	LOW		UNCLEAR	HIGH[q]		LOW	LOW
Unclear	Unclear	Unclear		Yes	Yes		Unclear	Unclear
Yes	Yes	Unclear		Unclear	Unclear		Unclear	Yes
LOW	LOW	LOW		LOW	LOW		HIGH[p]	LOW
HIGH[n]	HIGH[n]	Unclear[r]		LOW	LOW		LOW	HIGH[n]
Unclear	Unclear	Unclear		Unclear	Unclear		Unclear	Unclear
Yes	Yes	Yes		Yes	Yes		Yes	Yes
Yes	Yes	Yes		Yes	Yes		Yes	Yes
No	No	No		No	No		No	No
HIGH[o]	HIGH[o]	LOW		HIGH[o]	HIGH[o]		LOW	LOW

[a]Was a consecutive or random sample of patients enrolled?

[b]Was a case-control design avoided?

[c]Did the study avoid inappropriate exclusions?

[d]RoB rated as low, high or unclear

[e]Applicability rated low if no concerns about applicability, high if concerns about applicability and unclear if limited information available

[f]Were the index test results interpreted without knowledge of the results of the reference standard?

[g]If a threshold was used, was it pre-specified?

[h]Is the reference standard likely to correctly classify the target condition?

[i]Were the reference standard results interpreted without knowledge of the results of the index test?

[j]Was there an appropriate interval between index test(s) and reference standard?

[k]Did all patients receive a reference standard?

[l]Did patients receive the same reference standard?

[m]Were all patients included in the analysis?

[n]Rated high for applicability of reference standard as classification of SSLs as hyperplastic polyps/non-adenomatous may not be appropriate or may be a limitation of the analysis

[o]Concerns about exclusions or limited information on exclusions

[p]Reference standard was expert review by video rather than histopathology, which is likely to be less robust though required when implementing a resect-and-discard or diagnose-and-leave strategy

[q]Potentially interpreted detections on Discovery™ as neoplasias, which is not how the technology works, but information is unclear. Uncertainty with regards to applicability of this study given Discovery™ not outlined as having a CADx function in manufacturer submission

[r]Rated unclear for applicability as it is unclear how SSLs were treated in the analysis

Abbreviations: CADx, computer-aided characterisation; RoB, risk of bias; SLR, systematic literature review; SSL, sessile serrated lesion; SQ, signalling question; VCE, virtual chromoendoscopy.

References

1. Strapko A, Syed T, Baratta A, Strapko AM, Alexander K. P3030 - Artificial Intelligence (CAD-E)-Assisted Colonoscopy Helps Increase Adenoma Detection Rate (ADR) in the Afternoon Session. *ACG 2023 Annual Scientific Meeting Abstracts*. 2023. p. ACG 2023 Annual Scientific Meeting; 24 October 2023; Vancouver, BC, Canada: American College of Gastroenterology.

2. Alali AA, Alhashmi A, Alotaibi N, Ali N, Alali M, Alfadhli A. Artificial Intelligence for Adenoma and Polyp Detection During Screening and Surveillance Colonoscopy: A Randomized-Controlled Trial. *Journal of clinical medicine* 2025; **14**: 581.

3. Aniwan S, Mekritthikrai K, Kerr SJ, Tiankanon K, Vandaungden K, Sritunyarat Y, et al. Computer-aided detection, mucosal exposure device, their combination, and standard colonoscopy for adenoma detection: a randomized controlled trial. *Gastrointestinal Endoscopy* 2023; **97**: 507-16.

4. Desai M, Ausk K, Brannan D, Chhabra R, Chan W, Chiorean M, et al. Use of a Novel Artificial Intelligence System Leads to the Detection of Significantly Higher Number of Adenomas During Screening and Surveillance Colonoscopy: Results From a Large, Prospective, US Multicenter, Randomized Clinical Trial. *The American journal of gastroenterology* 2024; **119**: 1383-91.

5. Djinbachian R, Taghiakbari M, Barkun A, Medawar E, Alj A, Sidani S, et al. Optimized computer-assisted technique for increasing adenoma detection during colonoscopy: a randomized controlled trial. *Surgical Endoscopy* 2024; **39**: 1120-7.

6. Hiratsuka Y, Hisabe T, Ohtsu K, Yasaka T, Takeda K, Miyaoka M, et al. Evaluation of Artificial Intelligence: Computer-aided Detection of Colorectal Polyps. *Journal of the anus, rectum and colon* 2025; **9**: 79-87.

7. Huneburg R, Bucksch K, Schmeiser F, Heling D, Marwitz T, Aretz S, et al. Real-time use of artificial intelligence (CADEYE) in colorectal cancer surveillance of patients with Lynch syndrome-A randomized controlled pilot trial (CADLY). *United European gastroenterology journal* 2023; **11**: 60-8.

8. Miyaguchi K, Tsuzuki Y, Hirooka N, Matsumoto H, Ohgo H, Nakamoto H, et al. Linked-color imaging with or without artificial intelligence for adenoma detection: a randomized trial. *Endoscopy* 2024; **56**: 376-83.

9. Nakashima H, Kitazawa N, Fukuyama C, Kawachi H, Kawahira H, Momma K, et al. Clinical Evaluation of Computer-Aided Colorectal Neoplasia Detection Using a Novel Endoscopic Artificial Intelligence: A Single-Center Randomized Controlled Trial. *Digestion* 2023; **104**: 193-201.

10. Rondonotti E, Di Paolo D, Rizzotto ER, Alvisi C, Buscarini E, Spadaccini M, et al. Efficacy of a computer-aided detection system in a fecal immunochemical test-based organized colorectal cancer screening program: a randomized controlled trial (AIFIT study). *Endoscopy* 2022; **54**: 1171-9.

11. Scholer J, Alavanja M, de Lange T, Yamamoto S, Hedenstrom P, Varkey J. Impact of AI-aided colonoscopy in clinical practice: a prospective randomised controlled trial. *BMJ open gastroenterology* 2024; **11**: e001247.

12. Tiankanon K, Aniwan S, Kerr SJ, Mekritthikrai K, Kongtab N, Wisedopas N, et al. Improvement of adenoma detection rate by two computer-aided colonic polyp detection systems in high adenoma detectors: a randomized multicenter trial. *Endoscopy* 2024; **56**: 273-82.

13. Yamaguchi D, Shimoda R, Miyahara K, Yukimoto T, Sakata Y, Takamori A, et al. Impact of an artificial intelligence-aided endoscopic diagnosis system on improving endoscopy quality for trainees in colonoscopy: Prospective, randomized, multicenter study. *Digestive endoscopy : official journal of the Japan Gastroenterological Endoscopy Society* 2024; **36**: 40-8.

14. Zimmermann-Fraedrich K, Sehner S, Rosch T, Aschenbeck J, Schubert S, Liceni T, et al. No Effect of Computer Aided Diagnosis on Colonoscopic Adenoma Detection in a Large Pragmatic Multicenter Randomized Study. *American Journal of Gastroenterology* 2025: 10.14309/ajg.0000000000003500.

15. Maas MHJ, Rath T, Spada C, Soons E, Forbes N, Kashin S, et al. A computer-aided detection system in the everyday setting of diagnostic, screening, and surveillance colonoscopy: an international, randomized trial. *Endoscopy* 2024; **56**: 843-50.

16. Gimeno-Garcia AZ, Hernandez Negrin D, Hernandez A, Nicolas-Perez D, Rodriguez E, Montesdeoca C, et al. Usefulness of a novel computer-aided detection system for colorectal neoplasia: a randomized controlled trial. *Gastrointestinal endoscopy* 2023; **97**: 528-36.e1.

17. Lau LHS, Ho JCL, Lai JCT, Ho AHY, Wu CWK, Lo VWH, et al. Effect of Real-Time Computer-Aided Polyp Detection System (ENDO-AID) on Adenoma Detection in Endoscopists-in-Training: A Randomized Trial. *Clinical gastroenterology and hepatology : the official clinical practice journal of the American Gastroenterological Association* 2024; **22**: 630-41.e4.

18. Lui TK-L, Lam CP-M, To EW-P, Ko MK-L, Tsui VWM, Liu KS-H, et al. Endocuff With or Without Artificial Intelligence-Assisted Colonoscopy in Detection of Colorectal Adenoma: A Randomized Colonoscopy Trial. *Official journal of the American College of Gastroenterology | ACG* 2024; **119**: 1318-25.

19. Spada C, Salvi D, Ferrari C, Hassan C, Barbaro F, Belluardo N, et al. A comprehensive RCT in screening, surveillance, and diagnostic AI-assisted colonoscopies (ACCENDO-Colo study). *Digestive and Liver Disease* 2025; **57**: 762-9.

20. Vilkoite I, Tolmanis I, Meri HA, Polaka I, Mezmale L, Anarkulova L, et al. The Role of an Artificial Intelligence Method of Improving the Diagnosis of Neoplasms by Colonoscopy. *Diagnostics (Basel, Switzerland)* 2023; **13**: 701.

21. Gong D, Wu L, Zhang J, Mu G, Shen L, Liu J, et al. Detection of colorectal adenomas with a real-time computer-aided system (ENDOANGEL): a randomised controlled study. *The lancet Gastroenterology & hepatology* 2020; **5**: 352-61.

22. Yao L, Zhang L, Liu J, Zhou W, He C, Zhang J, et al. Effect of an artificial intelligence-based quality improvement system on efficacy of a computer-aided detection system in colonoscopy: a four-group parallel study. *Endoscopy* 2022; **54**: 757-68.

23. Yao L, Li X, Wu Z, Wang J, Luo C, Chen B, et al. Effect of artificial intelligence on novice-performed colonoscopy: a multicenter randomized controlled tandem study. *Gastrointestinal endoscopy* 2024; **99**: 91-9.e9.

24. Zhang H, Wu Q, Sun J, Wang J, Zhou L, Cai W, et al. A computer-aided system improves the performance of endoscopists in detecting colorectal polyps: a multi-center, randomized controlled trial. *Frontiers in medicine* 2023; **10**: 1341259.

25. Glissen Brown JR, Mansour NM, Wang P, Chuchuca MA, Minchenberg SB, Chandnani M, et al. Deep Learning Computer-aided Polyp Detection Reduces Adenoma Miss Rate: A United States Multi-center Randomized Tandem Colonoscopy Study (CADeT-CS Trial). *Clinical gastroenterology and hepatology : the official clinical practice journal of the American Gastroenterological Association* 2022; **20**: 1499-507.e4.

26. Liu P, Wang P, Glissen Brown JR, Berzin TM, Zhou G, Liu W, et al. The single-monitor trial: an embedded CADe system increased adenoma detection during colonoscopy: a prospective randomized study. *Therapeutic advances in gastroenterology* 2020; **13**: 1756284820979165.

27. Wang P, Berzin TM, Glissen Brown JR, Bharadwaj S, Becq A, Xiao X, et al. Real-time automatic detection system increases colonoscopic polyp and adenoma detection rates: a prospective randomised controlled study. *Gut* 2019; **68**: 1813-9.

28. Wang P, Liu X, Berzin TM, Glissen Brown JR, Liu P, Zhou C, et al. Effect of a deep-learning computer-aided detection system on adenoma detection during colonoscopy (CADe-DB trial): a double-blind randomised study. *The lancet Gastroenterology & hepatology* 2020; **5**: 343-51.

29. Wang P, Liu P, Glissen Brown JR, Berzin TM, Zhou G, Lei S, et al. Lower Adenoma Miss Rate of Computer-Aided Detection-Assisted Colonoscopy vs Routine White-Light Colonoscopy in a Prospective Tandem Study. *Gastroenterology* 2020; **159**: 1252-61.e5.

30. Wang P, Liu X-G, Kang M, Peng X, Shu M-L, Zhou G-Y, et al. Artificial intelligence empowers the second-observer strategy for colonoscopy: a randomized clinical trial. *Gastroenterology report* 2023; **11**: goac081.

31. Ahmad A, Wilson A, Haycock A, Humphries A, Monahan K, Suzuki N, et al. Evaluation of a real-time computer-aided polyp detection system during screening colonoscopy: AI-DETECT study. *Endoscopy* 2023; **55**: 313-9.

32. Engelke C, Graf M, Maass C, Tews HC, Kraus M, Ewers T, et al. Prospective study of computer-aided detection of colorectal adenomas in hospitalized patients. *Scandinavian journal of gastroenterology* 2023; **58**: 1194-9.

33. Karsenti D, Tharsis G, Perrot B, Cattan P, Percie du Sert A, Venezia F, et al. Effect of real-time computer-aided detection of colorectal adenoma in routine colonoscopy (COLO-GENIUS): a single-centre randomised controlled trial. *The lancet Gastroenterology & hepatology* 2023; **8**: 726-34.

34. Lagstrom RMB, Brauner KB, Bielik J, Rosen AW, Crone JG, Gogenur I, et al. Improvement in adenoma detection rate by artificial intelligence-assisted colonoscopy: Multicenter quasi-randomized controlled trial. *Endoscopy International Open* 2025; **13**: a25215169.

35. Levartovsky A, Levy I, Bruckmayer L, Klang E, Ben-Horin S, Kopylov U. Real-world artificial intelligence-aided colonoscopy does not improve adenoma detection rates in patients with Inflammatory Bowel Disease. *Journal of Crohn's and Colitis* 2023; **17**: i415-i6.

36. Mangas-Sanjuan C, de-Castro L, Cubiella J, Diez-Redondo P, Suarez A, Pellise M, et al. Role of Artificial Intelligence in Colonoscopy Detection of Advanced Neoplasias. *Annals of Internal Medicine* 2023; **176**: 1145-52.

37. Ortiz O, Daca-Alvarez M, Rivero-Sanchez L, Gimeno-Garcia AZ, Carrillo-Palau M, Alvarez V, et al. An artificial intelligence-assisted system versus white light endoscopy alone for adenoma detection in individuals with Lynch syndrome (TIMELY): an international, multicentre, randomised controlled trial. *The lancet Gastroenterology & hepatology* 2024; **9**: 802-10.

38. Repici A, Badalamenti M, Maselli R, Correale L, Radaelli F, Rondonotti E, et al. Efficacy of Real-Time Computer-Aided Detection of Colorectal Neoplasia in a Randomized Trial. *Gastroenterology* 2020; **159**: 512-20.e7.

39. Repici A, Spadaccini M, Antonelli G, Correale L, Maselli R, Galtieri PA, et al. Artificial intelligence and colonoscopy experience: lessons from two randomised trials. *Gut* 2022; **71**: 757-65.

40. Seager A, Sharp L, Neilson LJ, Brand A, Hampton JS, Lee TJW, et al. Polyp detection with colonoscopy assisted by the GI Genius artificial intelligence endoscopy module compared with standard colonoscopy in routine colonoscopy practice (COLO-DETECT): a multicentre, open-label, parallel-arm, pragmatic randomised controlled trial. *The lancet Gastroenterology & hepatology* 2024; **9**: 911-23.

41. Thiruvengadam NR, Solaimani P, Shrestha M, Buller S, Carson R, Reyes-Garcia B, et al. The Efficacy of Real-time Computer-aided Detection of Colonic Neoplasia in Community Practice: A Pragmatic Randomized Controlled Trial. *Clinical gastroenterology and hepatology : the official clinical practice journal of the American Gastroenterological Association* 2024; **22**: 2221-30.e15.

42. Wallace MB, Sharma P, Bhandari P, East J, Antonelli G, Lorenzetti R, et al. Impact of Artificial Intelligence on Miss Rate of Colorectal Neoplasia. *Gastroenterology* 2022; **163**: 295-304.e5.

43. Maas MHJ, Neumann H, Shirin H, Katz LH, Benson AA, Kahloon A, et al. A computer-aided polyp detection system in screening and surveillance colonoscopy: an international, multicentre, randomised, tandem trial. *The Lancet Digital health* 2024; **6**: e157-e65.

44. Cassinotti A, Zadro V, Parravicini M, Ferraris M, Balzarini M, Sessa F, et al. LCI/BLI chromoendoscopy plus CAD-EYE artificial intelligence for the detection and characterization of endoscopic visible lesions in ulcerative colitis. *Journal of Crohn's and Colitis* 2023; **17**: i291.

45. Djinbachian R, Haumesser C, Taghiakbari M, Pohl H, Barkun A, Sidani S, et al. Autonomous Artificial Intelligence vs Artificial Intelligence-Assisted Human Optical Diagnosis of Colorectal Polyps: A Randomized Controlled Trial. *Gastroenterology* 2024; **167**: 392-9.e2.

46. Rondonotti E, Hassan C, Tamanini G, Antonelli G, Andrisani G, Leonetti G, et al. Artificial intelligence-assisted optical diagnosis for the resect-and-discard strategy in clinical practice: the Artificial intelligence BLI Characterization (ABC) study. *Endoscopy* 2023; **55**: 14-22.

47. Sato K, Kuramochi M, Tsuchiya A, Yamaguchi A, Hosoda Y, Yamaguchi N, et al. Multicentre study to assess the performance of an artificial intelligence instrument to support qualitative diagnosis of colorectal polyps. *BMJ Open Gastroenterology* 2024; **11**: e001553.

48. Taghiakbari M, Rex DK, Pohl H, Djinbachian R, Huang F, Hassan C, et al. Pragmatic Resect and Discard Implementation Using Computer-Assisted Optical Polyp Diagnosis. *Gastroenterology* 2025; **168**: 154-6.e2.

49. Lopez-Serrano A, Voces A, Lorente JR, Santonja FJ, Algarra A, Latorre P, et al. Artificial intelligence for dysplasia detection during surveillance colonoscopy in patients with ulcerative colitis: A cross-sectional, non-inferiority, diagnostic test comparison study. *Gastroenterologia y hepatologia* 2024; **48**: 502210.

50. Bernhofer S, Prosenz J, Duller C, Venturi D, Maieron A. The Augmented Colonoscopy with Computer-Aided polyp Characterization (AC-CADx) study - prospective study comparing the diagnostic reliability of optical diagnosis of trainees with experts without AI. *American Journal of Gastroenterology* 2025: 10.14309/ajg.0000000000003558.

51. Hassan C, Balsamo G, Lorenzetti R, Zullo A, Antonelli G. Artificial Intelligence Allows Leaving-In-Situ Colorectal Polyps. *Clinical gastroenterology and hepatology : the official clinical practice journal of the American Gastroenterological Association* 2022; **20**: 2505-13.e4.

52. Rondonotti E, Bergna IMB, Paggi S, Amato A, Andrealli A, Scardino G, et al. White light computer-aided optical diagnosis of diminutive colorectal polyps in routine clinical practice. *Endoscopy international open* 2024; **12**: E676-E83.

53. Abdelrahim M, Hossain E, Subramaniam S, Bhandari P. Validation of a novel AI system (CADEYE) for in vivo characterization of colorectalpolyps. *Endoscopy* 2021; **53**: S50-S1.

54. Abdelrahim M, Siggens K, Iwadate Y, Maeda N, Htet H, Bhandari P. New AI model for neoplasia detection and characterisation in inflammatory bowel disease. *Gut* 2024; **73**: 725-8.

55. Australian New Zealand Clinical Trials Registry (ANZCTR). Does Artificial Intelligence Improve Polyp Detection at Colonoscopy?, 2022. Available from: https://www.cochranelibrary.com/central/doi/10.1002/central/CN-02428837/full. Date accessed: Jun 25.

56. Adiwinata R, Tandarto K, Arifputra J, Waleleng BJ, Gosal F, Rotty L, et al. The Impact of Artificial Intelligence in Improving Polyp and Adenoma Detection Rate During Colonoscopy: Systematic-Review and Meta-Analysis. *Asian Pacific journal of cancer prevention : APJCP* 2023; **24**: 3655-63.

57. Agazzi S, Chicco F, Scudeller L, Marzo V, Rosa C, Rossi G, et al. Real-time artificial intelligence-aided colonoscopy experience: The impact on routine clinical practice in a high-volume center-preliminary data. *United European Gastroenterology Journal* 2021; **9**: 813-4.

58. Ahmad A, Dhillon A, Wilson A, Suzuki N, Thomas-Gibson S, Humphries A, et al. Early evaluation of a computer assisted polyp detection system in bowel cancer screening. *Gut* 2021; **70**: A42.

59. Ahsan M, Anderson Z, Bakr M, Phillips RW. IS THERE A BENEFIT FOR COMPUTER-AIDED DETECTION OF POLYPS DURING SCREENING COLONOSCOPY AMONG EXPERIENCED ENDOSCOPISTS IN A COMMUNITY HOSPITAL SETTING? *Gastroenterology* 2024; **166**: S-977.

60. Ahsan M, Anderson Z, Jarbath M, Bakr M, Phillips RW. The Impact of Computer-aided Detection Technology in Adenoma Detection Rate Among Experienced Endoscopists in the Community Setting. *Journal of community hospital internal medicine perspectives* 2024; **14**: 42-8.

61. Alahmad M, Ghoris Y, Thomas C, Atieh MI, Abualkas H, Ahmed I, et al. REAL WORLD EVIDENCE ON THE EFFICIENCY OF AI- ENHANCED COLONOSCOPY: A CASE-CONTROL STUDY. *Gastrointestinal Endoscopy* 2024; **99**: AB37.

62. Ali F, Holzwanger E, Berzin T. ADHERENCE OF COMPUTER-AIDED POLYP DETECTION (CADE) CLINICAL TRIALS TO THE CONSOLIDATED STANDARDS OF REPORTING TRIALS-ARTIFICIAL INTELLIGENCE (CONSORT-AI) EXTENSION GUIDELINE - EVIDENCE REPORT FROM A SYSTEMATIC REVIEW AND META-ANALYSIS. *Gastrointestinal Endoscopy* 2024; **99**: AB15.

63. Ali H, Pamarthy R, Lambert K, Regan K. Adenoma and polyp detection rates by implementing artificial intelligence systems: A metaanalysis. *American Journal of Gastroenterology* 2021; **116**: S139.

64. Aljabiri M, Warshow U, Saadi R, Hassan A, Deduchova A, Tomagan J, et al. THE USE OF ARTIFICAL INTELLIGENCE IN COLONOSCOPY IMPROVES ADENOMA DETECTION RATES AND INVERSELY REDUCES THE RISK OF INTERVAL COLORECTAL CANCER; FIRST COMPARATIVE STUDY IN UAE. *Gastrointestinal Endoscopy* 2023; **97**: AB781.

65. Akram U, Fatima E, Ahmad MH, Ahmed S, Ahmad E, Bharadwaj HR. EFFECTIVENESS OF THE COMBINATION OF ENDOCUFF-ASSISTED AND COMPUTER-AIDED COLONOSCOPY: A META-ANALYSIS OF RANDOMIZED CONTROLLED TRIALS. *Gastrointestinal Endoscopy* 2025; **101**: S45.

66. Alahmad M, Ghoris YA, Ball CT, Atieh MI, Abualkas H, Nagaraju D, et al. Impact of Artificial Intelligence Enhanced Colonoscopy in a Clinical Practice Setting. *American Journal of Gastroenterology* 2023; **118**: S266 EP - S7.

67. Anand J, Gandhi S, Patel K. Artificial Intelligence-Aided Colonoscopy in a Real World Setting. *American Journal of Gastroenterology* 2023; **118**: S260.

68. Anderer S. Meta-Analysis: AI-Assisted Colonoscopy Increases Detection of Polyps, Adenomas. *Jama* 2024; **332**: 1968.

69. Aniwan S, Mekritthikrai K, Kerr SJ, Tiankanon K, Kongtub N, Piyachaturawat P, et al. THE DIFFERENCES IN ADENOMA DETECTION RATES AND OTHER INDICES BETWEEN STANDARD SCREENING COLONOSCOPY VS. COMPUTER-AIDED DETECTION VS. MUCOSAL EXPOSURE DEVICE VS. THE COMBINATION: A RANDOMIZED TRIAL. *Gastrointestinal Endoscopy* 2022; **95**: AB211.

70. Canadian Agency for Drugs and Technologies in Health. Artificial Intelligence-Assisted Colonoscopy for Detecting Polyps, Adenomas, Precancerous Lesions, and Colorectal Cancer: Health Technologies, 2024. Available from: https://www.ncbi.nlm.nih.gov/books/NBK611312/. Date accessed: Jun 25.

71. Antonelli G, Desideri F, Scarozza P, Andrisani G, Zerboni G, Furnari M, et al. ARTIFICIAL INTELLIGENCE FOR LEAVING IN SITU COLORECTAL POLYPS: RESULTS FROM A RANDOMISED TRIAL. *Gastrointestinal Endoscopy* 2025; **101**: S23 EP - S4.

72. Areia M, Mori Y, Correale L, Repici A, Bretthauer M, Sharma P, et al. Cost-effectiveness of artificial intelligence for screening colonoscopy: a modelling study. *The Lancet Digital health* 2022; **4**: e436-e44.

73. Areia M, Mori Y, Correale L, Repici A, Bretthauer M, Sharma P, et al. Cost-effectiveness of artificial intelligence for screening colonoscopy: a modelling study. *The Lancet Digital Health* 2022; **4**: e436-e44.

74. Armonis P, Vraka M, Petraki K, Mountaki A, Koumentakis N, Armonis A. COMPUTER-AIDED DETECTION IMPROVES ADENOMA DETECTION RATE FOR SCREENING COLONOSCOPY: A PROSPECTIVE TANDEM STUDY. *Gastroenterology* 2024; **166**: S-1498.

75. Ashat M, Klair JS, Singh D, Murali AR, Krishnamoorthi R. Impact of real-time use of artificial intelligence in improving adenoma detection during colonoscopy: A systematic review and meta-analysis. *Endoscopy international open* 2021; **9**: E513-E21.

76. Aslam MF, Bano S, Khalid M, Sarfraz Z, Sarfraz A, Sarfraz M, et al. The effectiveness of real-time computer-aided and quality control systems in colorectal adenoma and polyp detection during colonoscopies: a meta-analysis. *Annals of medicine and surgery (2012)* 2023; **85**: 80-91.

77. Aziz M, Haghbin H, Sayeh W, Alfatlawi H, Sharma S, Weissman S, et al. COMPARISON OF ARTIFICIAL INTELLIGENCE WITH OTHER INTERVENTIONS TO IMPROVE ADENOMA DETECTION RATE FOR COLONOSCOPY: A NETWORK META-ANALYSIS OF RANDOMIZED CONTROLLED TRIALS. *Gastroenterology* 2022; **162**: S-139.

78. Aziz M, Fatima R, Dong C, Lee-Smith W, Nawras A. The impact of deep convolutional neural network-based artificial intelligence on colonoscopy outcomes: A systematic review with meta-analysis. *Journal of gastroenterology and hepatology* 2020; **35**: 1676-83.

79. Aziz M, Haghbin H, Sayeh W, Alfatlawi H, Gangwani MK, Sohail AH, et al. Comparison of Artificial Intelligence With Other Interventions to Improve Adenoma Detection Rate for Colonoscopy: A Network Meta-analysis. *Journal of clinical gastroenterology* 2024; **58**: 143-55.

80. Bai Y, Zhao SB. Establishment and real-world validation of a computer-assisted polyp identification and localization system based on deep learning. *Journal of Digestive Diseases* 2019; **20**: 76-7.

81. Bai S, Singh B, Ethakota J, Ogedegbe OJ, Ntukidem OL, Chitkara A, et al. The role of artificial intelligence in colorectal cancer and polyp detection: A systematic review. *Journal of Clinical Oncology* 2025; **43**.

82. Bang CS, Lee JJ, Baik GH. Computer-Aided Diagnosis of Diminutive Colorectal Polyps in Endoscopic Images: Systematic Review and Meta-analysis of Diagnostic Test Accuracy. *Journal of medical Internet research* 2021; **23**: e29682.

83. Barkun AN, Sadri H. COST-EFFECTIVENESS ANALYSIS OF ARTIFICIAL INTELIGENCE-AIDED COLONOSCOPY FOR ADENOMA DETECTION IN COLORECTAL CANCER - A CANADIAN PERSPECTIVE. *Gastrointestinal Endoscopy* 2022; **95**: AB263-AB4.

84. Barkun AN, von Renteln D, Sadri H. Cost-effectiveness of Artificial Intelligence-Aided Colonoscopy for Adenoma Detection in Colon Cancer Screening. *Journal of the Canadian Association of Gastroenterology* 2023; **6**: 97-105.

85. Barua I, Vinsard DG, Jodal HC, Loberg M, Kalager M, Holme O, et al. Artificial intelligence for polyp detection during colonoscopy: a systematic review and meta-analysis. *Endoscopy* 2021; **53**: 277-84.

86. Barua I, Wieszczy P, Kudo S, Misawa M, Holme O, Gulati S, et al. ARTIFICIAL INTELLIGENCE FOR REAL-TIME OPTICAL DIAGNOSIS OF NEOPLASTIC POLYPS DURING COLONOSCOPY. *Gastrointestinal Endoscopy* 2022; **95**: AB267.

87. Barua I, Wieszczy P, Kudo SE, Misawa M, Holme O, Gulati S, et al. ARTIFICIAL INTELLIGENCE FOR REAL-TIME OPTICAL DIAGNOSIS OF NEOPLASTIC POLYPS DURING COLONOSCOPY. *Gut* 2022; **71**: A177.

88. Barua I, Wieszczy P, Kudo S-E, Misawa M, Holme O, Gulati S, et al. Real-Time Artificial Intelligence-Based Optical Diagnosis of Neoplastic Polyps during Colonoscopy. *NEJM evidence* 2022; **1**: EVIDoa2200003.

89. Behncke J, Kelmendi D, Mewes J, Gibson S, Popham G. Does Current Reimbursement Drive the Adoption of Computer-Aided Applications to Increase the Adenoma Detection in Colonoscopies - a Provider-Based Impact Model for Germany, France, and Italy. *Value in Health* 2023; **26**: S436.

90. Beran A, Nayfeh T, Ramai D, Spadaccini M, Hassan C, Repici A, et al. ENDOCUFF WITH OR WITHOUT ARTIFICIAL INTELLIGENCE-ASSISTED COLONOSCOPY FOR DETECTION OF COLORECTAL ADENOMA: A SYSTEMATIC REVIEW AND META-ANALYSIS OF RANDOMIZED CONTROLLED TRIALS. *Gastrointestinal Endoscopy* 2025; **101**: S275 EP - S6.

91. Bergagnini I, Buckley MC, Bhandari P, Ma M, Liu Y, Barraza L, et al. COMPUTER-AIDED DETECTION WITH ENDOCUFF IMPROVES ADENOMA DETECTION RATE: A QUALITY IMPROVEMENT INITIATIVE. *Gastroenterology* 2023; **164**: S-154.

92. Bergna IMB, Rondonotti E, Paggi S, Amato A, Andrealli A, Scardino G, et al. ARTIFICIAL INTELLIGENCE SYSTEM USING WHITE LIGHT FOR REAL-TIME OPTICAL CHARACTERIZATION OF COLONIC POLYPS. *Digestive and Liver Disease* 2023; **55**: S170-S1.

93. Bernhofer S, Maieron A. The impact of artificial intelligence on the adenoma detection rate (ADR): A comparison between experienced and trainee endoscopists' adr. *Endoscopy* 2021; **53**: S255-S6.

94. Bernhofer S, Prosenz J, Venturi D, Maieron A. Augmented Colonoscopy with Computer-Aided polyp characterization - evaluation of the performance of an artificial intelligence application in the classification of colorectal polyps. *Zeitschrift fur Gastroenterologie* 2024; **62**: e482.

95. Bilal M, Glissen Brown JR, Berzin TM. Using Computer-Aided Polyp Detection During Colonoscopy. *The American journal of gastroenterology* 2020; **115**: 963-6.

96. Bin Goh WW, Chia KYA, Cheung FK, Kee K, Lwin M, Schulz P, et al. SENIOR ENDOSCOPISTS ARE MORE LIKELY TO TRUST AND ACCEPT AI-ASSISTED COLONOSCOPY FOR DETECTION AND TREATMENT OF POLYPS COMPARED TO JUNIOR ENDOSCOPISTS. *Gastrointestinal Endoscopy* 2024; **99**: AB4.

97. Biscaglia G, Cocomazzi F, Loconte I, Mileti A, Paolillo R, Marra A, et al. REAL-TIME ARTIFICIAL INTELLIGENCE-AIDED COLONOSCOPY ELIMINATES DIFFERENCES IN ADENOMA

DETECTION RATE BETWEEN TRAINEES AND EXPERIENCED ENDOSCOPISTS IN TANDEM-COLONOSCOPIES. *Digestive and Liver Disease* 2022; **54**: S135-S6.

98. Biscaglia G, Cocomazzi F, Gentile M, Loconte I, Mileti A, Paolillo R, et al. Real-time, computer-aided, detection-assisted colonoscopy eliminates differences in adenoma detection rate between trainee and experienced endoscopists. *Endoscopy international open* 2022; **10**: E616-E21.

99. Brand M, Troya J, Krenzer A, De Maria C, Mehlhase N, Walter B, et al. Influence of artificial intelligence on polyp detection in a real life scenario. *United European Gastroenterology Journal* 2020; **8**: 783.

100. Brand M, Troya J, Krenzer A, Mehlhase N, Walter B, Meining A, et al. Artificial intelligence for polyp detection during colonoscopy-an in-depth analysis of a commercially available system. *United European Gastroenterology Journal* 2021; **9**: 805-6.

101. Bretthauer M, Ahmed J, Antonelli G, Beaumont H, Beg S, Benson A, et al. Use of computer-assisted detection (CADe) colonoscopy in colorectal cancer screening and surveillance: European Society of Gastrointestinal Endoscopy (ESGE) Position Statement. *Endoscopy* 2025; **57**: 667-73.

102. Budzyn K, Romanczyk M, Kitala D, Kolodziej P, Bugajski M, Adami HO, et al. Endoscopist De-Skilling after Exposure to Artificial Intelligence in Colonoscopy: A Multicenter Observational Study. *SSRN* 2024.

103. Burke CA, Macaron C, Singh A. Artificial intelligence-assisted adenoma detection in people with Lynch syndrome. *The Lancet Gastroenterology and Hepatology* 2024; **9**: 776-7.

104. Burton SJ, Shung D, Aslanian HR. PATIENT PERSPECTIVE OF USE OF ARTIFICIAL INTELLIGENCE DURING COLONOSCOPY. *Gastroenterology* 2024; **166**: S-889.

105. Bustamante-Balen M, Merino Rodriguez B, Barranco L, Monje J, Alvarez M, De Pedro S, et al. Cost-effectiveness analysis of artificial intelligence-aided colonoscopy for adenoma detection and characterization in Spain. *Endoscopy International Open* 2025; **13**.

106. Bustamante-Balen M, Merino Rodriguez B, Barranco Priego L, Monje J, Alvarez M, de Pedro S, et al. EE600 Cost-Effectiveness and Budget Impact Analysis of Introducing Artificial Intelligence-Aided Colonoscopy for Adenoma Detection and Characterization in Spain. *Value in Health* 2024; **27**: S173.

107. Caillo L, Delliot C, Chevallier T, Bourgaux JF, Prost A, Brunaud-Gagniard B, et al. COLODETECT 1: comparative evaluation of endocuff with computer-aided detection versus computer-aided detection alone versus standard colonoscopy for enhancing adenoma detection rates during screening colonoscopy-a pilot study. *Therapeutic Advances in Gastroenterology* 2024; **17**.

108. Calce SI, Djinbachian R, Michal V, Gefflot C, Bouchard S, Bouin M, et al. ENDOSCOPISTS AND COMPUTER-AIDED DECISION SUPPORT INTERACTION FOR DETECTION OF COLORECTAL POLYPS. *Gastrointestinal Endoscopy* 2025; **101**: S36 EP - S7.

109. Carlini L, Massimi D, Mori Y, Antonelli G, Rizkala T, Spadaccini M, et al. Large language models for detecting colorectal polyps in endoscopic images. *Gut* 2025: gutjnl-2025-335091.

110. Chadha N, Healey M, Puri P, Gilles HC, Fuchs M, Spataro J, et al. Artificial Intelligence Improves Adenoma Detection Amongst Gastroenterology Fellows. *American Journal of Gastroenterology* 2024; **119**: S271 EP - S2.

111. Chang P, Kong N, Nguyen D, Wang SJ, Sharma N, Amini M, et al. AI-ASSISTED COLONOSCOPY IMPROVES ADR IN GASTROENTEROLOGY FELLOWS: AN INTERIM ANALYSIS OF A RANDOMIZED CONTROL TRIAL. *Gastroenterology* 2024; **166**: S-931.

112. Chaudhary MYN, Jawwad M, Ismail M, Chaudhary MHN, Hasan F, Rai R, et al. Novel Artificial Intelligence (AI) Systems in Detecting Adenomas in Colonoscopy: A Systematic Review and Network Meta-Analysis. *American Journal of Gastroenterology* 2024; **119**: S1204 EP - S5.

113. Cheng CL, Cadoni S, Cheng WY, Gallittu P, Mura D, Utzeri E, et al. EVALUATION OF ARTIFICIAL INTELLIGENCE FOR ADENOMA DETECTION IN WATER EXCHANGE COLONOSCOPY: INTERIM ANALYSIS OF THE WEAID RANDOMIZED CONTROLLED TRIAL. *Gastrointestinal Endoscopy* 2025; **101**: S43 EP - S4.

114. Cheng CL, Kuo YL, Su IC, Cadoni S, Zou KY, Lee YS, et al. Evaluation of a Computer-Aided Detection Device During Water Exchange Colonoscopy: A Pragmatic Implementation Performance Improvement Study. *American Journal of Gastroenterology* 2023; **118**: S189 EP - S90.

115. Cheng W, Kuo YL, Su IC, Lee BP, Tsui YN, Lin HT, et al. Evaluating the Performance of a Computer-Aided Diagnosis System in Implementing Diagnose-and-Leave and Resect-and-Discard Strategies for Diminutive Colorectal Polyps: A Real-World Pragmatic Study. *American Journal of Gastroenterology* 2024; **119**: S263 EP - S4.

116. Cheng CL, Cadoni S, Kuo YL, Su IC, Tsui YN, Lee BP, et al. PERFORMANCE OF REAL-TIME COMPUTER-AIDED POLYP DETECTION USING WATER EXCHANGE COLONOSCOPY: A PRELIMINARY PILOT STUDY. *Gastrointestinal Endoscopy* 2024; **99**: AB17-AB8.

117. Cheong SH, Bomman S, Witten B, Ho S, Nhat Pham H, Trieu R, et al. Colon AI-scopy: Artificial Intelligence Influence on Detection and Removal of Non-Neoplastic Polyps, Diminutive Hyperplastic Polyps, and Sessile Serrated Polyps in Screening Colonoscopy. *American Journal of Gastroenterology* 2024; **119**: S618 EP - S9.

118. Chinese Clinical Trial Register (ChiCTR). The effect of artificial intelligence-assisted shortening of colonoscopy withdrawal time on adenoma detection, 2024. Available from: https://www.cochranelibrary.com/central/doi/10.1002/central/CN-02743739/full. Date accessed: Jun 25.

119. Chinese Clinical Trial Register (ChiCTR). Study on the detection value of colorectal adenomas by different artificial intelligence-assisted diagnostic devices, 2024. Available from: https://www.cochranelibrary.com/central/doi/10.1002/central/CN-02782598/full. Date accessed: Jun 25.

120. Chinese Clinical Trial Register (ChiCTR). Comparison between AI+Magic-Cap-assisted colonoscopy and traditional colonoscopy, 2025. Available from: https://www.cochranelibrary.com/central/doi/10.1002/central/CN-02813113/full. Date accessed: Jun 25.

121. Chinese Clinical Trial Register (ChiCTR). The impact of a computer aided diagnosis system based on deep learning on incresing polyp detection rate during colonoscopy, a

prospective double blind study (ChiCTR1800017675), 2018. Available from: https://www.cochranelibrary.com/central/doi/10.1002/central/CN-01908117/full. Date accessed: Feb 25.

122. Chinese Clinical Trial Register (ChiCTR). A multicenter randomized controlled study for evaluating the effectiveness of artificial intelligence in improving colonoscopy quality (ChiCTR1900021984), 2019. Available from: https://www.cochranelibrary.com/central/doi/10.1002/central/CN-01972896/full. Date accessed: Feb 25.

123. Chinese Clinical Trial Register (ChiCTR). The impact of a colon polyp detection CAD system based on deep learning on colon adenoma miss rate: a randomized prospective tandem study (ChiCTR1900023086), 2019. Available from: https://www.cochranelibrary.com/central/doi/10.1002/central/CN-01974376/full. Date accessed: Feb 25.

124. Chinese Clinical Trial Register (ChiCTR). A multicenter prospective randomized controlled trial for the impact of a computer-aided colon polyp detection system based on deep learning on colon adenoma detection during colonoscopy in comparison with junior endoscopist second observer (ChiCTR1900025235), 2019. Available from: https://www.cochranelibrary.com/central/doi/10.1002/central/CN-01975304/full. Date accessed: Feb 25.

125. Chinese Clinical Trial Register (ChiCTR). The comparison of AI-assisted colonoscopy and conventional colonoscopy for the detection of polyps in colorectal cancer screening in White Light and LCI mode (ChiCTR1900026726), 2019. Available from: https://www.cochranelibrary.com/central/doi/10.1002/central/CN-02445925/full. Date accessed: Feb 25.

126. Chinese Clinical Trial Register (ChiCTR). Artificial Intelligence-assisted Colonoscopy for Detection of Colon Polyps: a Prospective Randomized Cohort Study (ChiCTR1900027307), 2019. Available from: https://www.cochranelibrary.com/central/doi/10.1002/central/CN-02065374/full. Date accessed: Feb 25.

127. Chinese Clinical Trial Register (ChiCTR). Difference Analysis of Colonoscopy Detection Rate of Adenoma Assisted by Artificial Intelligence (ChiCTR2000034887), 2020. Available from: https://www.cochranelibrary.com/central/doi/10.1002/central/CN-02436580/full. Date accessed: Feb 25.

128. Chinese Clinical Trial Register (ChiCTR). Comparing adenoma detection rate of cap-assisted colonoscopy and conventional colonoscopy with and without artificial intelligence: a prospective, randomized, single-center trial (ChiCTR2000034889), 2020. Available from: https://www.cochranelibrary.com/central/doi/10.1002/central/CN-02184623/full. Date accessed: Feb 25.

129. Chinese Clinical Trial Register (ChiCTR). Effects of phased application of artificial intelligence-assisted polyp diagnosis system on independent colonoscopy performance of endoscopists: a multicenter randomized controlled trial (ChiCTR2100045262), 2021. Available from: https://www.cochranelibrary.com/central/doi/10.1002/central/CN-02349265/full. Date accessed: Feb 25.

130. Chinese Clinical Trial Register (ChiCTR). A prospective, multicenter, randomized control trial of a real-time quality-control system for the colonoscopy examination of outpatient (ChiCTR2200063455), 2022. Available from: https://www.cochranelibrary.com/central/doi/10.1002/central/CN-02545593/full. Date accessed: Feb 25.

131. Chinese Clinical Trial Register (ChiCTR). Research on Artificial Intelligence Disease Identification System Based on Dynamic Big Data of Digestive Endoscope (ChiCTR2200063891), 2022. Available from: https://www.cochranelibrary.com/central/doi/10.1002/central/CN-02560604/full. Date accessed: Feb 25.

132. Chinese Clinical Trial Register (ChiCTR). Effect of an Artificial Intelligence Computer-Aided Detection System on Adenoma Detection: a Multicenter Randomized Controlled Trial (ChiCTR2200064399), 2022. Available from: https://www.cochranelibrary.com/central/doi/10.1002/central/CN-02552942/full. Date accessed: Feb 25.

133. Chinese Clinical Trial Register (ChiCTR). A multi-center study to observe the adenoma detection rate for different colonoscopy withdrawal time with AI-assisted detection system or not (ChiCTR2300067573), 2023. Available from: https://www.cochranelibrary.com/central/doi/10.1002/central/CN-02561626/full. Date accessed: Feb 25.

134. Chinese Clinical Trial Register (ChiCTR). A Clinical Trial of the Effectiveness and Safety of Software Assisting Diagnose the Intestinal Polyp Digestive Endoscopy by Analysis of Colonoscopy Medical Images From Electronic Digestive Endoscopy Equipment (ChiCTR2300071120), 2023. Available from: https://www.cochranelibrary.com/central/doi/10.1002/central/CN-02583391/full. Date accessed: Feb 25.

135. Chinese Clinical Trial Register (ChiCTR). A clinical trial to validate the effectiveness and safety of AI-assisted colonoscopy (ChiCTR2300073421), 2023. Available from: https://www.cochranelibrary.com/central/doi/10.1002/central/CN-02589137/full. Date accessed: Feb 25.

136. Chinese Clinical Trial Register (ChiCTR). To verify the effect of artificial intelligence-assisted diagnosis combined with water exchange technology on the diagnostic efficiency of colonoscopy (ChiCTR2400082293), 2024. Available from: https://www.cochranelibrary.com/central/doi/10.1002/central/CN-02685507/full. Date accessed: Feb 25.

137. Chinese Clinical Trial Register (ChiCTR). The effect of artificial intelligence on the adenoma detection rates and missed rates in water-assisted colonoscopy (ChiCTR2400082752), 2024. Available from: https://www.cochranelibrary.com/central/doi/10.1002/central/CN-02690912/full. Date accessed: Feb 25.

138. Chieng M, Schauer C, Wang M, Neave M, Watson S, Van Rijnsoever M, et al. Effect of artificial intelligence on adenoma detection during colonoscopy: The first New Zealand experience. *Journal of Gastroenterology and Hepatology* 2022; **37**: 231-2.

139. Chikatimalla R, Kumar S, Panjiyar BK, Suresh SB, Rehman OA, kumari A, et al. REAL-TIME ARTIFICIAL INTELLIGENCE VS STANDARD COLONOSCOPY IN THE EARLY DETECTION OF COLORECTAL CANCER: A META-ANALYSIS. *Gastrointestinal Endoscopy* 2025; **101**: S48 EP - S9.

140. Chilakapati A, Pokala VC, Khan SM, Khan A, Venkatareddy A, Maddineni G, et al. Enhancing Colorectal Cancer Detection by Using Artificial Intelligence-Driven Colonoscopy to Detect Polyp and Adenoma Rates. *American Journal of Gastroenterology* 2024; **119**: S316 EP - S7.

141. Chin S-E, Wan F-T, Ladlad J, Chue K-M, Teo E-K, Lin C-L, et al. One-year review of real-time artificial intelligence (AI)-aided endoscopy performance. *Surgical endoscopy* 2023; **37**: 6402-7.

142. Cho H, Sakamoto T, Nakamura K, Mizuguchi Y, Hisada I, Makiguchi M, et al. THE PERFORMANCE OF CAD-EYETM FOR DIFFERENTIAL DIAGNOSIS OF COLORECTAL POLYPS. *Digestive Endoscopy* 2022; **34**: 83.

143. Chow K, Bell M, Cumpian N, Amour M, Hsu R, Eysselein V, et al. LONG-TERM IMPACT OF ARTIFICIAL INTELLIGENCE ON COLORECTAL ADENOMA DETECTION IN A SAFETY-NET HOSPITAL: A ONE-YEAR FOLLOW-UP. *Gastrointestinal Endoscopy* 2024; **99**: AB36-AB7.

144. Chow KW, Bell MT, Cumpian N, Amour M, Hsu RH, Eysselein VE, et al. Long-term impact of artificial intelligence on colorectal adenoma detection in high-risk colonoscopy. *World journal of gastrointestinal endoscopy* 2024; **16**: 335-42.

145. Chow K, Bell M, Amour M, Eysselein V, Srivastava N, Fleischman MW, et al. Impact of Artificial Intelligence on Colorectal Adenoma Detection in High-Risk Colonoscopy: Initial Experience at a Safety-Net Hospital. *American Journal of Gastroenterology* 2023; **118**: S317 EP - S8.

146. Chowdary P, Kmiotek EK, Mirnezami R. Application of machine learning approaches in the diagnosis and management of colorectal cancer. *British Journal of Surgery* 2023; **110**: iii34.

147. Chung GE, Lee J, Lim SH, Kang HY, Kim J, Song JH, et al. A prospective comparison of two computer aided detection systems with different false positive rates in colonoscopy. *npj Digital Medicine* 2024; **7**: 366.

148. Claassen PL, Johnston DL, Adkins BJ, Panko NB, Visger JS. IMPROVING ADENOMA AND POLYP DETECTION RATES WITH COMPUTER-AIDED DETECTION: LESSENING DISPARITIES IN COLORECTAL CANCER SCREENING IN RURAL AMERICA. *Gastrointestinal Endoscopy* 2025; **101**: S285.

149. Contreras C, Ortiz C, Pena P, Finke A, Herrera S, Alonso M, et al. The Impact of CADe on Sessile Serrated Adenomas Detection Rate During Colonoscopy. *American Journal of Gastroenterology* 2023; **118**: S243 EP - S4.

150. Contreras C, Ortiz C, Pena P, Finke A, Herrera S, Pena N, et al. The Evaluation of Artificial Intelligence on Adenoma Detection Rate in a Latin American Community Setting. *American Journal of Gastroenterology* 2023; **118**: S244.

151. Contreras CP, Contreras F, Abreu D, Mella I, Martinez M, Polanco K, et al. IMPACT OF CADE ON ADENOMA DETECTION RATES DURING LINKED COLOR IMAGING-ENHANCED

COLONOSCOPIES IN A NON-ACADEMIC OUTPATIENT FACILITY IN THE DOMINICAN REPUBLIC. *Gastrointestinal Endoscopy* 2025; **101**: S44 EP - S5.

152. Contreras F, Contreras C, Abbott AF, Herrera S, Matos E, Nunez M, et al. Adenoma Detection Rate Using LCI vs White Light Colonoscopy Both With and Without the Use of Artificial Intelligence: A Prospective Study in a Non-Academic Center in Latin America. *American Journal of Gastroenterology* 2022; **117**: S435.

153. Cooper J, Norwood DA, Evers CD, Mulki R, Sanchez-Luna S, Sarkis F, et al. Head-to-Head Comparison of Two Computer Aided Detection (CAD-e) Systems on Colonoscopy Performance Metrics. *American Journal of Gastroenterology* 2024; **119**: S362 EP - S3.

154. Coron E, Vanbiervliet G, Prouvost V, Medlej C, Musquer N, Le Rhun M, et al. Is artifical intelligence (CAD EYE) useful to not only detect but also to characterizesmall colorectal polyps? First results from a prospective french multicenter study. *Endoscopy* 2021; **53**: S50.

155. Coronel E, Barringer D, Ross WA, Shafi M, Ge PS. PHYSICIAN AND STAFF ATTITUDES TOWARDS IMPLEMENTATION OF ARTIFICIAL INTELLIGENCE-ASSISTED COLONOSCOPY. *Gastroenterology* 2022; **162**: S-841.

156. Correia C, Gravito-Soares E, Gravito-Soares M, Amaro P, Macedo C, Ferreira M, et al. Artificial intelligence in the characterization of colorectal polyps: A prospective study ina clinical setting using cadeye. *Endoscopy* 2021; **53**: S156-S7.

157. de Castro EG, Argenal LFA, de la Osa DR, Urra CP, Molleda LC, Fernandez JS, et al.?EL EMPLEO DE UN DISPOSITIVO DE INTELIGENCIA ARTIFICIAL EN COLONOSCOPIAS DE CRIBADO DE CCR MEJORA LOS ESTANDARES DE CALIDAD DE LAS COLONOSCOPIAS? EXPERIENCIA EN UN HOSPITAL DE SEGUNDO NIVEL. *Gastroenterologia y Hepatologia* 2023; **46**: 90238.

158. De Lange G, Prouvost V, Rahmi G, Vanbiervliet G, Le Berre C, Mack S, et al. Artificial intelligence for characterization of colorectal polyps: Prospective multicenter study. *Endoscopy international open* 2024; **12**: E413-E8.

159. Deliwala S, Hamid K, Barbarawi M, Zayed Y, Kandel P, Lakshman H, et al. Artificial Intelligence (AI)-Guided vs Routine Colonoscopy for Colorectal Polyps: A Meta-Analysis of Recent Randomized Controlled Trials. *American Journal of Gastroenterology* 2020; **115**: S263-S4.

160. Deliwala SS, Hamid K, Barbarawi M, Lakshman H, Zayed Y, Kandel P, et al. Artificial intelligence (AI) real-time detection vs. routine colonoscopy for colorectal neoplasia: a meta-analysis and trial sequential analysis. *International journal of colorectal disease* 2021; **36**: 2291-303.

161. Desai P, Giordano N, Wasser T, Whitebloom D, Shah N. COMPUTER-AIDED POLYP DETECTION INCREASES ADENOMA DETECTION RATE IN A HIGH ADENOMA DETECTING GROUP: A MULTI-SITE COMMUNITY PRACTICE EXPERIENCE. *Gastrointestinal Endoscopy* 2024; **99**: AB33.

162. Djinbachian R, Haumesser C, Taghiakbari M, Alj A, Barkun A, Liu J, et al. COMPARING ENDOSCOPIST DIAGNOSIS OF COLORECTAL POLYPS ASSISTED BY ARTIFICIAL INTELLIGENCE (CADX) VS CADX WITHOUT ENDOSCOPIST INPUT: A RANDOMIZED CONTROLLED TRIAL. *Journal of the Canadian Association of Gastroenterology* 2024; **7**: 9-10.

163. Djinbachian R, Haumesser C, Taghiakbari M, Pohl H, Barkun A, Sidani S, et al. AUTONOMOUS ARTIFICIAL INTELLIGENCE VERSUS AI ASSISTED HUMAN OPTICAL DIAGNOSIS OF COLORECTAL POLYPS: A RANDOMIZED CONTROLLED TRIAL. *Gastrointestinal Endoscopy* 2024; **99**: AB11.

164. Djinbachian R, Taghiakbari M, Barkun A, Medawar E, Panzini B, Sidani S, et al. OPTIMIZED COMPUTER ASSISTED TECHNIQUE FOR INCREASING ADENOMA DETECTION DURING COLONOSCOPY: A RANDOMIZED CONTROLLED TRIAL. *Journal of the Canadian Association of Gastroenterology* 2024; **7**: 97-8.

165. Djinbachian R, Pohl H, Rex DK, Barkun A, Hassan C, Soucy G, et al. Accuracy of histopathology evaluation in diminutive colonic polyps diagnosed as neoplastic by computer-aided characterisation. *Gut* 2025; **74**: 703 EP - 5.

166. Djinbachian R, Haumesser C, Taghiakbari M, Pohl H, Barkun A, Sidani S, et al. Autonomous Artificial Intelligence versus AI Assisted Human optical diagnosis of colorectal polyps: a randomized controlled trial. 2024; **56**: S146.

167. Dominitz JA, Gawron A, McKee G, Hoggatt K, Kaltenbach TR. DURABILITY OF THE IMPACT OF ARTIFICIAL INTELLIGENCE COMPUTER-AIDED DETECTION (CADE) ON THE ALL-INDICATION ADENOMA DETECTION RATE (ADR) AND OTHER COLONOSCOPY QUALITY INDICATORS: A RANDOMIZED PRAGMATIC STUDY. *Gastrointestinal Endoscopy* 2025; **101**: S25 EP - S6.

168. Dominitz JA, Gawron A, McKee G, Hoggatt K, Kaltenbach TR. IMPACT OF ARTIFICIAL INTELLIGENCE COMPUTER-AIDED DETECTION (CADE) ON THE ALL-INDICATION ADENOMA DETECTION RATE (ADR): PRAGMATIC QUALITY IMPROVEMENT (QI) STUDY IN A NATIONAL HEALTHCARE SYSTEM. *Gastroenterology* 2024; **166**: S-975.

169. Doring S, Hann A, Zoller W. Artificial intelligence in endoscopic screening for colorectal cancer-expensive add-on or cost saving. *United European Gastroenterology Journal* 2021; **9**: 812.

170. Dos Santos CEO, Malaman D, Sanmartin IDA, Leao ABS, Leao GS, Pereira-Lima JC. Performance of artificial intelligence in the characterization of colorectal lesions. *Saudi journal of gastroenterology : official journal of the Saudi Gastroenterology Association* 2023; **29**: 219-24.

171. German Clinical Trials Register (DRKS). Real-time use of artificial intelligence (CADEYE) in the colo-rectal cancer surveillance of Lynch syndrome patients (CADLY), 2020. Available from: https://www.cochranelibrary.com/central/doi/10.1002/central/CN-02240016/full. Date accessed: Jun 25.

172. German Clinical Trials Register (DRKS). Computer-aided detection of polyps during colonoscopy - a prospective, controlled study, 2021. Available from: https://www.cochranelibrary.com/central/doi/10.1002/central/CN-02350362/full. Date accessed: Jun 25.

173. German Clinical Trials Register (DRKS). Artificial Intelligence in screening colonoscopy, 2021. Available from: https://www.cochranelibrary.com/central/doi/10.1002/central/CN-02350433/full. Date accessed: Jun 25.

174. German Clinical Trials Register (DRKS). Real-time use of artificial intelligence (CAD EYE) in the colorectal cancer surveillance of Lynch syndrome patients (CADLYII) – an international multicenter trial, 2023. Available from: https://www.cochranelibrary.com/central/doi/10.1002/central/CN-02572871/full. Date accessed: Jun 25.

175. Eelbode T, Hassan C, Neumann H, Demedts I, Sinonquel P, Roelandt P, et al. 879 A PROSPECTIVE MULTI-CENTER VALIDATION STUDY FOR AUTOMATED POLYP DETECTION AS A SECOND OBSERVER. *Gastrointestinal Endoscopy* 2020; **91**: AB72.

176. El Zoghbi M, Shaukat A, Hassan C, Anderson JC, Repici A, Gross SA. Artificial Intelligence-Assisted Optical Diagnosis: A Comprehensive Review of Its Role in Leave-In-Situ and Resect-and-Discard Strategies in Colonoscopy. *Clinical and translational gastroenterology* 2023; **14**: e00640.

177. Elhadi M, Haas EM. Using Artificial Intelligence-Enhanced White-Light Colonoscopy for Predicting Deeply Invasive Colorectal Cancer: A Diagnostic Accuracy Meta-Analysis. *Journal of the American College of Surgeons* 2024; **239**: S120 EP - S1.

178. Ellison AC, Dougherty CE, George PS, Conway TG, Dougherty SG, Atiyeh L, et al. Effect of Artificial Intelligence Polyp Detection in an Office-Based Endoscopy Practice. *American Journal of Gastroenterology* 2023; **118**: S316.

179. Ellrichmann M, Conrad C, Loose J, Jessen P, Heits N, Schulte B, et al. THE USE OF ARTIFICIAL INTELLIGENCE IMPROVES QUALITY CRITERIA IN SCREENING COLONOSCOPY. *Gastrointestinal Endoscopy* 2023; **97**: AB729-AB30.

180. England C, Washington J. Artificial Intelligence And Health Technology Assessment: Playing Catch-Up. *International Journal of Technology Assessment in Health Care* 2024; **40**: S91.

181. Foroutan F, Vandvik PO, Helsingen LM, Kalager M, Rutter M, Selby K, et al. Computer aided detection and diagnosis of polyps in adult patients undergoing colonoscopy: a living clinical practice guideline. *BMJ* 2025: e082656.

182. Fitting D, Krenzer A, Troya J, Bock W, Meining A, Hann A. Development and comparison of the polyp detection system endomind with a commercially available cade system. *United European Gastroenterology Journal* 2021; **9**: 824-5.

183. Gach T, Orzeszko Z, Bogacki P, Krzak JM, Markowska B, Szura M, et al. Artificial Intelligence During Colonoscopy: The First Thousand Procedures. *Surgical Endoscopy* 2023; **37**: S294.

184. Gallagher S, Davis L, Pajovich H, Kjelstrom S, Wong P. AI in Action: Augmenting Adenoma Detection Rates in a Community-Based Gastroenterology Practice. *American Journal of Gastroenterology* 2024; **119**: S299.

185. Gangwani MK, Haghbin H, Ishtiaq R, Dahiya DS, Hayat U, Lee-Smith WM, et al. Comparing Adenoma Detection Rate (ADR) in Single vs Dual Observer vs Artificial Intelligence-Assisted Colonoscopy: A Network Analysis of Randomized Controlled Trials. *American Journal of Gastroenterology* 2023; **118**: S292 EP - S3.

186. Gangwani MK, Haghbin H, Ishtiaq R, Hasan F, Dillard J, Jaber F, et al. Single Versus Second Observer vs Artificial Intelligence to Increase the ADENOMA Detection Rate of Colonoscopy-A Network Analysis. *Digestive diseases and sciences* 2024; **69**: 1380-8.

187. Gross S, Trautwein C, Behrens A, Winograd R, Palm S, Lutz HH, et al. Computer-based classification of small colorectal polyps by using narrow-band imaging with optical magnification. *Gastrointestinal endoscopy* 2011; **74**: 1354-9.

188. Gu G, Seacor T, Hyder SM, Marshall C. Disconnect Between Perceptions of Artificial Intelligence and Adenoma Detection Rate at a Tertiary Center: Survey and Retrospective. *American Journal of Gastroenterology* 2024; **119**: S380.

189. Guerrero MGR, Ho S, Bhardwaj M. Computer-Aided Detection (CADe) and Its Effect on Adenoma Detection Rate (ADR) in a Single Tertiary Center. *American Journal of Gastroenterology* 2022; **117**: S243.

190. Guerrero Vinsard D, Barua I, Jodal HC, Loberg M, Kalager M, Holme O, et al. Artificial intelligence for polyp detection during colonoscopy: A systematic review and meta-analysis. *United European Gastroenterology Journal* 2020; **8**: 754-5.

191. Halvorsen N, Hassan C, Correale L, Pilonis N, Helsingen LM, Spadaccini M, et al. Benefits, burden, and harms of computer aided polyp detection with artificial intelligence in colorectal cancer screening: microsimulation modelling study. *BMJ medicine* 2025; **4**: e001446.

192. Halvorsen N, Mori Y. Cost-Effectiveness for Artificial Intelligence in Colonoscopy. *Gastrointestinal endoscopy clinics of North America* 2025; **35**: 401-5.

193. Hann A, Troya J, Fitting D. Current status and limitations of artificial intelligence in colonoscopy. *United European gastroenterology journal* 2021; **9**: 527-33.

194. Hardy NP, MacAonghusa P, Dalli J, Epperlein JP, Huxel P, Khan MF, et al. Explainable endoscopic artificial intelligence method for real-time in situ significant rectal lesion characterization: a prospective cohort study. *International journal of surgery (London, England)* 2025; **111**: 2313 EP - 6.

195. Hassan C, Rizkala T, Mori Y, Spadaccini M, Misawa M, Antonelli G, et al. Computer-aided diagnosis for the resect-and-discard strategy for colorectal polyps: a systematic review and meta-analysis. *The Lancet Gastroenterology and Hepatology* 2024; **9**: 1010 EP - 9.

196. Hassan C, Balsamo G, Lorenzetti R, Zullo A, Antonelli G. ARTIFICIAL INTELLIGENCE FOR LEAVING-IN-SITU COLORECTAL POLYPS: RESULTS OF A REAL TIME CLINICAL TRIAL. *Digestive and Liver Disease* 2022; **54**: S81.

197. Hassan C, Lorenzetti R, Zullo A, Antonelli G. ARTIFICIAL INTELLIGENCE FOR LEAVING-IN-SITU COLORECTAL POLYPS: RESULTS OF A CLINICAL TRIAL. *Gastrointestinal Endoscopy* 2022; **95**: AB219-AB20.

198. Hassan C, Spadaccini M, Alfarone L, Da Rio L, Solitano V, Ferretti S, et al. CHARACTERIZATION COMPARISON BETWEEN TWO CAD SYSTEMS (COMBO CAD STUDY) IN REAL-LIFE ENDOSCOPY: AN INTERIM ANALYSIS. *Digestive and Liver Disease* 2022; **54**: S77-S8.

199. Hassan C, Spadaccini M, Alfarone L, Da Rio L, Solitano V, Ferretti S, et al. CHARACTERIZATION COMPARISON BETWEEN TWO CAD SYSTEMS (COMBO CAD STUDY) IN

REAL- LIFE ENDOSCOPY: AN INTERIM ANALYSIS. *Gastrointestinal Endoscopy* 2022; **95**: AB228-AB9.

200. Hassan C, Badalamenti M, Maselli R, Correale L, Iannone A, Radaelli F, et al. Computer-aided detection-assisted colonoscopy: classification and relevance of false positives. *Gastrointestinal endoscopy* 2020; **92**: 900-4.e4.

201. Hassan C, Bhandari P, Antonelli G, Repici A. Artificial intelligence for non-polypoid colorectal neoplasms. *Digestive endoscopy : official journal of the Japan Gastroenterological Endoscopy Society* 2021; **33**: 285-9.

202. Hassan C, Misawa M, Rizkala T, Mori Y, Sultan S, Facciorusso A, et al. Computer-Aided Diagnosis for Leaving Colorectal Polyps In Situ : A Systematic Review and Meta-analysis. *Annals of internal medicine* 2024; **177**: 919-28.

203. Hassan C, Povero M, Pradelli L, Spadaccini M, Repici A. Cost-utility analysis of real-time artificial intelligence-assisted colonoscopy in Italy. *Endoscopy international open* 2023; **11**: E1046-E55.

204. Hassan C, Sharma P, Mori Y, Bretthauer M, Rex DK, Repici A, et al. Comparative Performance of Artificial Intelligence Optical Diagnosis Systems for Leaving in Situ Colorectal Polyps. *Gastroenterology* 2023; **164**: 467-9.e4.

205. Hassan C, Spadaccini M, Iannone A, Maselli R, Jovani M, Chandrasekar VT, et al. Performance of artificial intelligence in colonoscopy for adenoma and polyp detection: a systematic review and meta-analysis. *Gastrointestinal endoscopy* 2021; **93**: 77-85.e6.

206. Hassan C, Spadaccini M, Mori Y, Foroutan F, Facciorusso A, Gkolfakis P, et al. Real-Time Computer-Aided Detection of Colorectal Neoplasia During Colonoscopy : A Systematic Review and Meta-analysis. *Annals of internal medicine* 2023; **176**: 1209-20.

207. Hassan C, Wallace MB, Sharma P, Maselli R, Craviotto V, Spadaccini M, et al. New artificial intelligence system: first validation study versus experienced endoscopists for colorectal polyp detection. *Gut* 2020; **69**: 799-800.

208. Haviland B, Sarvepalli S, Welch S, Nunn D, Clark M, Botti C. A RETROSPECTIVE ANALYSIS OF ADENOMA DETECTION WITH GI GENIUS, ENDOCUFF, THEIR COMBINATION, AND STANDARD COLONOSCOPY. *Gastrointestinal Endoscopy* 2025; **101**: S54.

209. Herman T, Mohamed K, Vinsard DG, Freeman M, Gravely A, Westanmo A, et al. Time and Experience Do Not Lead to Improved Adenoma Detection Rate With Artificial Intelligence-Assisted Colonoscopy: An 11-Month Implementation Trial. *American Journal of Gastroenterology* 2024; **119**: S623 EP - S4.

210. Herman T, Vinsard DG, Freeman M, Gravely A, Westanmo A, Bilal M, et al. Artificial Intelligence-Assisted Colonoscopy Is Associated With Higher Conversions From Screening to Therapeutic Exams. *American Journal of Gastroenterology* 2024; **119**: S575 EP - S6.

211. Herman T, Vinsard DG, Freeman M, Westanmo A, Gravely A, Bilal M, et al. Head-to-Head Real World Comparative Analysis of Two Artificial Intelligence-Assisted Colonoscopy Systems. *American Journal of Gastroenterology* 2024; **119**: S334 EP - S5.

212. Herman T, Vinsard DG, Freeman M, Westanmo A, Gravely A, Bilal M, et al. 699 ARTIFICIAL INTELLIGENCE-ASSISTED COLONOSCOPY FAILED TO INCREASE ADENOMA DETECTION RATE: AN IMPLEMENTATION STUDY. *Gastroenterology* 2024; **166**: S-164.

213. Hocke M, Frieling T, Eckardt AJ, Teubner D, Kiesslich R. DISCOVERY AI COMBINATION WITH G-EYE COLONOSCOPY SIGNIFICANTLY INCREASES ADENOMA DETECTION RATE - RESULTS OF A MULTICENTER STUDY. *Gastrointestinal Endoscopy* 2022; **95**: AB217.

214. Holanda EU, Meine GC, Barbosa EC, Santo P, Nau AL, Moore KM, et al. COMPUTER-AIDED DETECTION WITH OR WITHOUT MUCOSAL-EXPOSURE DEVICES IN COLONOSCOPY: A SYSTEMATIC REVIEW AND META-ANALYSIS WITH TRIAL SEQUENTIAL ANALYSIS. *Gastrointestinal Endoscopy* 2025; **101**: S51 EP - S2.

215. Horiuchi H, Tamai N, Kamba S, Inomata H, Ohya TR, Sumiyama K. REAL-TIME COMPUTER-AIDED DIAGNOSIS OF DIMINUTIVE COLORECTAL POLYPS USING AN AUTOFLUORESCENCE IMAGING SYSTEM. *Gastrointestinal Endoscopy* 2019; **89**: AB387.

216. Horiuchi H, Tamai N, Kamba S, Inomata H, Ohya TR, Sumiyama K. Real-time computer-aided diagnosis of diminutive rectosigmoid polyps using an auto-fluorescence imaging system and novel color intensity analysis software. *Scandinavian journal of gastroenterology* 2019; **54**: 800-5.

217. Hsu WF, Chang LC, Chang WY, Lin HH, Kuo CY, Wu MS, et al. IMPLEMENTATION OF THE NOVEL AI-BASED CECAL RECOGNITION SYSTEM IMPROVED THE ADENOMA DETECTION RATE IN SCREENING COLONOSCOPY. *Gastroenterology* 2023; **164**: S-312.

218. Htet H, Siggens K, Suthan H, Longcroft-Wheaton G, Hamson J, Alkandari A, et al. O14 Time to implement resect & discard service into practice: two novel ways of polyp sizing and optical diagnosis with CADx. *Gut* 2023; **72**: A7.

219. Htet H, Siggens K, Longcroft-Wheaton G, Suthan H, Popoola V, Bombeo L, et al. REAL-TIME COMPARATIVE STUDY OF CADX AND SIZING DEVICES FOR COLORECTAL POLYPS DURING COLONOSCOPY: A SOLUTION TO IMPLEMENT RESECT & DISCARD? *Gut* 2024; **73**: A148 EP - A9.

220. Htet HM, Hassan C, Maselli R, Neumann H, Mangiavillano B, Sharma P, et al. CADE RESULTS FROM A LARGE INTERNATIONAL, MULTI-CENTRE, RANDOMISED-CONTROLLED TRIAL: MORE ADENOMAS DETECTED, NO INCREASE IN UNNECESSARY POLYPECTOMIES. *Gastrointestinal Endoscopy* 2025; **101**: S12 EP - S3.

221. Huang L, Chang P, Wang S, Nguyen D, Chang FW, Lee H, et al. Artificial Intelligence-Assisted Colonoscopy Improves Adenoma Detection Rate (ADR) in Both Low and High ADR Endoscopists: A Meta-Analysis. *American Journal of Gastroenterology* 2023; **118**: S518 EP - S9.

222. Huang D, Shen J, Hong J, Zhang Y, Dai S, Du N, et al. Effect of artificial intelligence-aided colonoscopy for adenoma and polyp detection: a meta-analysis of randomized clinical trials. *International journal of colorectal disease* 2022; **37**: 495-506.

223. Huneburg R, Bucksch K, Schmeir F, Heling D, Marwitz T, Kaczmarek DJ, et al. Real-time use of artificial intelligence in colorectal cancer surveillance of patients with Lynch syndrome - a randomized controlled trial. *Familial Cancer* 2022; **21**: 592-4.

224. Ishiyama M, Kudo S-E, Misawa M, Mori Y, Maeda Y, Ichimasa K, et al. Impact of the clinical use of artificial intelligence-assisted neoplasia detection for colonoscopy: a large-scale prospective, propensity score-matched study (with video). *Gastrointestinal endoscopy* 2022; **95**: 155-63.

225. ISRCTN. Future of real time endoscopy, artificial intelligence, 2024. Available from: https://www.cochranelibrary.com/central/doi/10.1002/central/CN-02674880/full. Date accessed: Jun 2025.

226. ISRCTN. ColoVision: using computers to instantly find and describe colorectal polyps, 2023. Available from: https://www.cochranelibrary.com/central/doi/10.1002/central/CN-02595307/full. Date accessed: Jun 25.

227. Jabbal I, Patel R, Ferretti F, Avalos M, Patel P. Utilization of Endocuff-Assisted Colonoscopy and Computer-Aided Detection in Optimizing Colonoscopies in the Elderly. *American Journal of Gastroenterology* 2023; **118**: S249 EP - S50.

228. Jaber F, Alsakarneh S, Madi MY, Numan L, Beran A, Abboud Y, et al. COMPARISON OF COLONOSCOPY QUALITY METRICS USING ARTIFICIAL INTELLIGENCE ASSISTED COLONOSCOPY VERSUS STANDARD COLONOSCOPY: A SYSTEMATIC REVIEW AND META-ANALYSIS OF RANDOMIZED CONTROLLED TRIALS. *Gastroenterology* 2024; **166**: S-1498.

229. James LW, Jie Lee JW, Wu C, Liang R, Lin K, Thian MY, et al. REAL-WORLD VALIDATION OF A COMPUTER-AIDED DIAGNOSIS SYSTEM FOR CHARACTERIZATION OF POLYP HISTOLOGY IN COLONOSCOPY: A PROSPECTIVE MULTICENTER STUDY. *Gastrointestinal Endoscopy* 2023; **97**: AB732-AB3.

230. Jawwad M, Maheshwari M, Aleem S, Batool Z, Alsubaie N, Syed S, et al. Novel Artificial Intelligence Systems in Detecting Adenomas in Colonoscopy: A Systemic Review and Network Meta-Analysis. *SSRN* 2025.

231. Jimenez BS, Solis MR, Hernandez-Guerrero A. ARTIFICIAL INTELLIGENCE ENHANCES ADENOMA DETECTION RATE IN LYNCH SYNDROME DURING COLONOSCOPY. *Gastrointestinal Endoscopy* 2024; **99**: AB31-AB2.

232. Jin B, Huang L, Liu S, Lyu B, Hu Y. Effect of an artificial intelligence-assisted recognition system on colonoscopy quality. *Zhonghua nei ke za zhi* 2024; **63**: 1111 EP - 5.

233. Jin XF, Ma HY, Shi JW, Cai JT. Efficacy of artificial intelligence in reducing miss rates of GI adenomas, polyps, and sessile serrated lesions: a meta-analysis of randomized controlled trials. *Gastrointestinal Endoscopy* 2024; **99**: 667-75.e1.

234. Jootun M, Bretos-Azcona P, Wallace M, Agirrezabal I. PCN115 Economic Evaluation of Artificial Intelligence-Assisted Colonoscopy for Routine Screening of Low- to High-Risk Colorectal Cancer Patients in the United Kingdom. *Value in Health* 2020; **23**: S443.

235. Jootun M, Bretos-Azcona P, Wallace M, Agirrezabal I. PCN99 Economic Evaluation of Artificial Intelligence-Assisted Colonoscopy for Routine Screening of High-Risk Colorectal Cancer Patients in Spain. *Value in Health* 2020; **23**: S440.

236. Japan Primary Registries Network (JPRN) - Japan Registry for Clinical Trials (jRCT). Clinical trial of colonoscopy using computer-aided detection systems in colorectal cancer screening (JPRN-jRCT1032230396), 2023. Available from:

https://www.cochranelibrary.com/central/doi/10.1002/central/CN-02629188/full. Date accessed: Feb 25.

237. Japan Primary Registries Network (JPRN) - University Hospital Medical Information Network (UMIN) Clinical Trials Registry. The usefulness of computer-aided detection technologies based on artificial intelligence with image enhanced endoscopy for detecting colon adenoma; single center, randomized controlled trial (JPRN-UMIN000050685), 2023. Available from: https://www.cochranelibrary.com/central/doi/10.1002/central/CN-02594194/full. Date accessed: Feb 25.

238. Japan Primary Registries Network (JPRN) - University Hospital Medical Information Network (UMIN) Clinical Trials Registry. A multicenter prospective randomized controlled trial evaluating the usefulness of a real-time colonoscopy diagnostic support system (JPRN-UMIN000051437), 2023. Available from: https://www.cochranelibrary.com/central/doi/10.1002/central/CN-02605573/full. Date accessed: Feb 25.

239. Japan Primary Registries Network (JPRN) - University Hospital Medical Information Network (UMIN) Clinical Trials Registry. Influence of fatigue and stress on endoscopy accuracy and its relationship to AI colonoscopy: a multicenter clinical trial (JPRN-UMIN000053693), 2024. Available from: https://www.cochranelibrary.com/central/doi/10.1002/central/CN-02725962/full. Date accessed: Feb 25.

240. Japan Primary Registries Network (JPRN) - University Hospital Medical Information Network (UMIN) Clinical Trials Registry. Randomised trial examining the sessile serrated lesion detection rate using linked color imaging in combination with an artificial intelligence assisted colonoscopy (JPRN-UMIN000053777), 2024. Available from: https://www.cochranelibrary.com/central/doi/10.1002/central/CN-02725977/full. Date accessed: Feb 25.

241. Japan Primary Registries Network (JPRN) - Japan Registry for Clinical Trials (jRCT). CAD for Polyp Detection Trial (JPRN-jRCTs022190014), 2019. Available from: https://www.cochranelibrary.com/central/doi/10.1002/central/CN-02070300/full. Date accessed: Feb 25.

242. Japan Primary Registries Network (JPRN) - Japan Registry for Clinical Trials (jRCT). The validation study of detectability and diagnostic accuracy of AI-aided endoscopic diagnosis system for colonoscopy (JPRN-jRCTs032190061), 2019. Available from: https://www.cochranelibrary.com/central/doi/10.1002/central/CN-02070320/full. Date accessed: Feb 25.

243. Kader R, Bassett P, Kabir Y, Cheung S, Macabodbod LG, Jayanthi A, et al. RANDOMIZED CONTROLLED TRIAL OF A CLOUD-BASED ARTIFICIAL INTELLIGENCE (AI) COMPUTER-AIDED DIAGNOSIS (CADX) SYSTEM IN NON-EXPERT ENDOSCOPISTS. *Gastrointestinal Endoscopy* 2024; **99**: AB12-AB3.

244. Kader R, Bassett P, Kabir Y, Cheung S, Macabodbod LG, Jayanthi A, et al. RANDOMIZED CONTROLLED TRIAL OF A CLOUD-BASED ARTIFICIAL INTELLIGENCE POLYP DETECTION SYSTEM (CADDIE). *Gastrointestinal Endoscopy* 2024; **99**: AB23.

245. Kader R, Hassan C, Lanas A, Romanczyk M, Romanczyk T, Kotowski B, et al. CLOUD-BASED ARTIFICIAL INTELLIGENCE FOR DETECTION OF COLORECTAL NEOPLASIA - A RANDOMIZED CONTROLLED TRIAL (EAGLE TRIAL). *Gastrointestinal Endoscopy* 2025; **101**: S25.

246. Kader R, Kabir Y, Cheung S, Chand M, Lovat L. RANDOMIZED CONTROLLED TRIAL (RCT) OF A CLOUDBASED ARTIFICIAL INTELLIGENCE (AI) COMPUTERAIDED DIAGNOSIS (CADX) SYSTEM INNON-EXPERT ENDOSCOPISTS. *Gut* 2024; **73**: A46 EP - A7.

247. Kader R, Bassett P, Kabir Y, Cheung S, Macabodbod L, Jayanthi A, et al. Randomized controlled trial of a cloud-based artificial intelligence (AI) computer-aided diagnosis (CADx) system in non-expert endoscopists (CADDIE). 2024; **56**: S55.

248. Kader R, Bassett P, Kabir Y, Cheung S, Macabodbod L, Jayanthi A, et al. Randomized controlled trial of a cloud-based artificial intelligence polyp detection system (CADDIE). 2024; **56**: S57.

249. Kader R, Kabir Y, Cheung S, Jayanthi A, Chand M, Lovat LB. RANDOMISED CONTROLLED TRIAL OF A CLOUD-BASED ARTIFICIAL INTELLIGENCE POLYP DETECTION SYSTEM (CADDIE). 2024; **73**: A31-A2.

250. Kamba S, Matsui H, Horiuchi H, Tamai N, Ohya T, Tonouchi A, et al. The real-time detection and differential diagnosis of colorectal polyps in colonoscop y with an artificial intelligence algorithm; a prospective observational study. *United European Gastroenterology Journal* 2019; **7**: 50.

251. Kamba S, Tamai N, Horiuchi H, Matsui H, Kobayashi M, Ego M, et al. ID: 3519580 A MULTICENTRE RANDOMIZED CONTROLLED TRIAL TO VERIFY THE REDUCIBILITY OF ADENOMA MISS RATE OF COLONOSCOPY ASSISTED WITH ARTIFICIAL INTELLIGENCE BASED SOFTWARE. *Gastrointestinal Endoscopy* 2021; **93**: AB195.

252. Kamba S, Tamai N, Saitoh I, Matsui H, Horiuchi H, Kobayashi M, et al. Reducing adenoma miss rate of colonoscopy assisted by artificial intelligence: a multicenter randomized controlled trial. *Journal of gastroenterology* 2021; **56**: 746-57.

253. Kandel P, Mupparaju V, Mathur S, Patel V, Shinde T, Chandrupatla S. ARTIFICIAL INTELLIGENE AIDED COLONOSOCPY DOES NOT IMPROVE ENDOSCOPIST PERFORMACE IN COMMUNITY SETTINGS. *Gastroenterology* 2024; **166**: S-1490.

254. Kandel PN, Mupparaju V, Mathur K, Patel V, Shinde T, Chandrupatla S. Artificial Intelligence-Aided Colonoscopy Does Not Improve Endoscopist Performance in Community Settings. *American Journal of Gastroenterology* 2024; **119**: S248 EP - S9.

255. Karthikeyan R, McClain B, Delfino K, Thuppal S, Poola V. COMPUTER-AIDED DETECTION OF POLYPS WITH ARTIFICIAL INTELLIGENCE DURING COLONOSCOPY IN THE HANDS OF AN EXPERIENCED COLORECTAL SURGEON. *Diseases of the Colon and Rectum* 2024; **67**: e632 EP - e3.

256. Kawamoto A, Takenaka K, Okamoto R, Watanabe M, Ohtsuka K. Ao - Takenaka K. Systematic review of artificial intelligence-based image diagnosis for inflammatory bowel disease. *Digestive Endoscopy* 2022; **34**: 1311-9.

257. Keshtkar K, Safarpour AR, Heshmat R, Sotoudehmanesh R, Keshtkar A. A Systematic Review and Meta-analysis of Convolutional Neural Network in the Diagnosis of Colorectal

Polyps and Cancer. *The Turkish journal of gastroenterology : the official journal of Turkish Society of Gastroenterology* 2023; **34**: 985-97.

258. Keswani R, Thakkar U, Sals A, Pandolfino J. ADOPTION OF A COMPUTER-AIDED DETECTION SYSTEM SIGNIFICANTLY IMPROVES POLYP DETECTION IN ROUTINE CLINICAL PRACTICE. *Gastrointestinal Endoscopy* 2023; **97**: AB468-AB9.

259. Keswani RN, Thakkar U, Sals A, Pandolfino JE. A Computer-Aided Detection (CADe) System Significantly Improves Polyp Detection in Routine Practice. *Clinical gastroenterology and hepatology : the official clinical practice journal of the American Gastroenterological Association* 2024; **22**: 893-5.e1.

260. Keswani R, Pandolfino JE. Adoption of a Computer-Aided Detection System May Improve Polyp Detection in Patients With Positive Stool-Based Testing. *American Journal of Gastroenterology* 2024; **119**: S268.

261. Khatri V, Sachdeva A, Verma P, Jagannath S. The Use of Artificial Intelligence to Improve Adenoma Detection Rate in a Community Practice. *American Journal of Gastroenterology* 2023; **118**: S253.

262. Khouri A, Dickson HC, Green A, Sonnier WP, Hanjar A, Ray GJ, et al. Effect of Computer-Aided Detection Device on the Adenoma Detection Rate and Serrated Detection Rate Among Trainee Fellows. *American Journal of Gastroenterology* 2024; **119**: S370.

263. Kim JH, Park SC, Kim HS. Role of Artificial Intelligence in Improving Quality of Colonoscopy. *The Korean journal of gastroenterology = Taehan Sohwagi Hakhoe chi* 2025; **85**: 137 EP - 45.

264. Kim JH, Wang J, Pence C, Magahis P, Dawod E, Schnoll-Sussman F, et al. GI Genius increases small and right-sided adenoma and sessile serrated lesion detection rate when used with EndoCuff in a real-world setting: a retrospective United States study. *Clinical endoscopy* 2025; **58**: 438-47.

265. Kim HJ, Parsa N, Byrne MF. The role of artificial intelligence in colonoscopy. *Seminars in Colon and Rectal Surgery* 2024; **35**: 101007.

266. Kim JH, Wang J, Pence C, Magahis P, Dawod E, Schnoll-Sussman F, et al. EFFICACY OF COMPUTER-AIDED POLYP DETECTION WHEN USED ALONE AND IN CONJUNCTION WITH A MUCOSA-EXPOSURE DEVICE DURING COLONOSCOPY IN A REAL-WORLD SETTING. *Gastrointestinal Endoscopy* 2024; **99**: AB34-AB5.

267. Klare P, Sander C, Prinzen M, Munzenmayer C, Nowack S, Von Delius S, et al. Computer assisted detection of polyps during colonoscopy-results from an initial technical study. *Biomedizinische Technik* 2017; **62**: S15.

268. Kliegis L, Obst W, Bruns J, Weigt J. Can a Polyp Detection and Characterization System Predict Complete Resection? *Digestive diseases (Basel, Switzerland)* 2022; **40**: 115-8.

269. Kobayashi R, Yoshida N, Tomita Y, Hashimoto H, Inoue K, Hirose R, et al. Detailed Superiority of the CAD EYE Artificial Intelligence System over Endoscopists for Lesion Detection and Characterization Using Unique Movie Sets. *Journal of the anus, rectum and colon* 2024; **8**: 61-9.

270. Kode V, Czech T, Levy P, Lee F, Chen T. IMPACT OF ARTIFICIAL INTELLIGENCE ON ADENOMA DETECTION RATE OF GASTROENTEROLOGISTS AT A TERTIARY CARE ENDOSCOPY SUITE: A QUALITY IMPROVEMENT STUDY AND STATISTICAL ANALYSIS. *Gastroenterology* 2024; **166**: S-1496.

271. Koh FH, Ladlad J, Teo E-K, Lin C-L, Foo F-J, Foo Fj TWJSSSHLMLNJLKFHCCADKJ, et al. Real-time artificial intelligence (AI)-aided endoscopy improves adenoma detection rates even in experienced endoscopists: a cohort study in Singapore. *Surgical endoscopy* 2023; **37**: 165-71.

272. Koleth G, Emmanue J, Spadaccini M, Mascagni P, Khalaf K, Mori Y, et al. Artificial intelligence in gastroenterology: Where are we heading? *Endoscopy international open* 2022; **10**: E1474-E80.

273. Kominami Y, Yoshida S, Tanaka S, Sanomura Y, Hirakawa T, Raytchev B, et al. Computer-aided diagnosis of colorectal polyp histology by using a real-time image recognition system and narrow-band imaging magnifying colonoscopy. *Gastrointestinal endoscopy* 2016; **83**: 643-9.

274. Kudaravalli P, Peng H, Adu-Gyamfi KO, Sobani ZA, Chandrasekar V, Sifuentes H, et al. Artificial Intelligence-Assisted Colon Polyp Detection: Initial Experience by Gastroenterology Fellows. *American Journal of Gastroenterology* 2022; **117**: S207-S9.

275. Kumar A, Aravind N, Gillani T, Kumar D. Artificial intelligence breakthrough in diagnosis, treatment, and prevention of colorectal cancer - A comprehensive review. *Biomedical Signal Processing and Control* 2025; **101**: 107205.

276. Kuo J, Shao PP, Romero T, Leung FW. META-ANALYSIS COMPARING ADVANCED ADENOMA DETECTION RATE OF WATER EXCHANGE AND COMPUTER-AIDED DETECTION COLONOSCOPY. *Gastroenterology* 2024; **166**: S-976.

277. Labaki C, Uche-Anya EN, Berzin TM. Artificial Intelligence in Gastrointestinal Endoscopy. *Gastroenterology Clinics of North America* 2024; **53**: 773 EP - 86.

278. Ladabaum U, Shepard J, Weng Y, Desai M, Singer S, Mannalithara A. COMPUTER-AIDED DETECTION OF POLYPS DOES NOT IMPROVE COLONOSCOPIST PERFORMANCE IN A PRAGMATIC IMPLEMENTATION TRIAL. *Gastroenterology* 2023; **164**: S-153.

279. Lam TY, Yi Y, Cheung FK, Goh WWB, Sung JJ. LEVEL OF ACCEPTANCE AND TRUST OF ARTIFICIAL INTELLIGENCE AMONG GASTROENTEROLOGY NURSES. *Gastroenterology* 2024; **166**: S-888.

280. Lambin T, Pioche M, Jacques J. Artificial intelligence for improving screening colonoscopies. *Hepato-Gastro et Oncologie Digestive* 2021; **28**: 656-61.

281. Lau HSL, Ho CLJ, Lai CTJ, Ho AHY, Wu CWK, Lo VWH, et al. EFFECT of REAL-TIME COMPUTER-AIDED POLYP DETECTION SYSTEM (ENDOAID) on ADENOMA DETECTION in ENDOSCOPIST-IN-TRAINING: A SINGLE-BLIND RANDOMIZED CONTROLLED TRIAL (ENDOAID-TRAIN STUDY). *United European Gastroenterology Journal* 2023; **11**: 251-3.

282. Lau HSL, Ho CLJ, Lai CTJ, Tang RSY, Chiu PWY. New computer-aided polyp detection system (Endo-aid) increased the 5-10mm adenoma detection rate in junior endoscopists during colonoscopies-a pilot study. *United European Gastroenterology Journal* 2021; **9**: 809-10.

283. Lee C, Parker CH, Liu LW, Salim M, Jeyalingam T. EXPLORING ENDOSCOPIST PERCEPTIONS OF ARTIFICIAL INTELLIGENCE-AIDED COLONOSCOPY: A QUALITATIVE ANALYSIS. *Journal of the Canadian Association of Gastroenterology* 2024; **7**: 104-5.

284. Lee MC, Jeyalingam T, Parker CH, Liu LW. TANDEM STUDY DESIGN IS LESS LIKELY TO DEMONSTRATE IMPROVED ADENOMA DETECTION RATE THAN PARALLEL STUDY DESIGN IN THE ASSESSMENT OF ARTIFICIAL INTELLIGENCE-ASSISTED COLONOSCOPY. *Journal of the Canadian Association of Gastroenterology* 2023; **6**: 68.

285. Lee MCM, Parker CH, Liu LWC, Farahvash A, Jeyalingam T. Impact of study design on adenoma detection in the evaluation of artificial intelligence-aided colonoscopy: a systematic review and meta-analysis. *Gastrointestinal endoscopy* 2024; **99**: 676-87.e16.

286. Lee F, Kode V, Farah K, Chen T. Impact of Artificial Intelligence on Adenoma Detection Rate of Gastroenterologists at a Tertiary Care Endoscopy Suite: A Quality Improvement Study and Descriptive Analysis. *American Journal of Gastroenterology* 2023; **118**: S146 EP - S7.

287. Lei S, Wang Z, Tu M, Liu P, Lei L, Xiao X, et al. Adenoma detection rate is not influenced by the time of day in computer-aided detection colonoscopy. *Medicine* 2020; **99**: e23685.

288. Levy I, Bruckmayer L, Klang E, Ben-Horin S, Kopylov U. ARTIFICIAL INTELLIGENCE-AIDED COLONOSCOPY DOES NOT INCREASE ADENOMA DETECTION RATE IN ROUTINE CLINICAL PRACTICE. *United European Gastroenterology Journal* 2022; **10**: 56.

289. Levy I, Bruckmayer L, Klang E, Ben-Horin S, Kopylov U. Artificial Intelligence-Aided Colonoscopy Does Not Increase Adenoma Detection Rate in Routine Clinical Practice. *The American journal of gastroenterology* 2022; **117**: 1871-3.

290. Li J, Wu L, Du D, Liu J, Wang Q, Luo Z, et al. Cost-effectiveness analysis of an artificial intelligence-assisted diagnosis and treatment system for gastrointestinal endoscopy. *Chinese Journal of Digestive Endoscopy* 2023; **40**: 206-11.

291. Li WJ, Wu C, Lock Khor CJ, Liang RFH, Lee JWJ, So J, et al. Real World Validation of an Artificial Intelligence Characterization Support System for Prediction of Polyp Histology in Colonoscopy: Interim Analysis of a Prospective Multicenter Study. *American Journal of Gastroenterology* 2022; **117**: S99-S100.

292. Li J, Lu J, Yan J, Tan Y, Liu D. Artificial intelligence can increase the detection rate of colorectal polyps and adenomas: a systematic review and meta-analysis. *European journal of gastroenterology & hepatology* 2021; **33**: 1041-8.

293. Li M-D, Huang Z-R, Shan Q-Y, Chen S-L, Zhang N, Hu H-T, et al. Performance and comparison of artificial intelligence and human experts in the detection and classification of colonic polyps. *BMC gastroenterology* 2022; **22**: 517.

294. Li TY, Mansour NM. CAN ARTIFICIAL INTELLIGENCE ASSISTED COLONOSCOPY COMPENSATE FOR INADEQUATE BOWEL PREPARATION? *Gastrointestinal Endoscopy* 2025; **101**: S21.

295. Lin K, Galati JS, Popov V, Rex DK, Shaukat A, Pochapin MB, et al. COMPARISON OF COMPUTER-AIDED POLYP DETECTION (CADE) AND ENDOSCOPIC MECHANICAL ATTACHMENT ON ADENOMA DETECTION RATE: A SYSTEMATIC REVIEW AND META-ANALYSIS. *Gastrointestinal Endoscopy* 2022; **95**: AB172.

296. Linlawan S, Tiankanon K, Aniwan S, Kongtub N, Vateekul P, Nupairoj N, et al. USING COMPUTER-AIDED POLYP DETECTION SYSTEM(CADE) TO MAINTAIN THE HIGH QUALITY IN ADENOMA RATE DURING COMMUNITY-BASED COLORECTAL CANCER SCREENING IN THAILAND: A RANDOMIZED TRIAL. *Gastrointestinal Endoscopy* 2024; **99**: AB14.

297. Liu P, Wu J, He C, Wang W. ENDOANGEL versus water exchange for the detection of colorectal adenomas. *Therapeutic advances in gastroenterology* 2023; **16**: 17562848231218570.

298. Liu W-N, Zhang Y-Y, Bian X-Q, Wang L-J, Yang Q, Zhang X-D, et al. Study on detection rate of polyps and adenomas in artificial-intelligence-aided colonoscopy. *Saudi journal of gastroenterology : official journal of the Saudi Gastroenterology Association* 2020; **26**: 13-9.

299. Lou S, Du F, Song W, Xia Y, Yue X, Yang D, et al. Artificial intelligence for colorectal neoplasia detection during colonoscopy: a systematic review and meta-analysis of randomized clinical trials. *EClinicalMedicine* 2023; **66**: 102341.

300. Lu Z, Huang L, Wu L, Yu H. LONG-TERM EFFECTIVENESS OF ARTIFICIAL INTELLIGENCE SYSTEM IN IMPROVING ADENOMA DETECTION RATE: A MULTICENTER SELF-CONTROLLED STUDY. *United European Gastroenterology Journal* 2022; **10**: 1029.

301. Lu Z, Yao L, Zhang L, Yu H. ARTIFICIAL INTELLIGENCE ASSISTANCE IMPROVES AS ENDOSCOPIST FATIGUE INCREASES: SECONDARY ANALYSIS OF TWO RANDOMIZED TRIALS. *United European Gastroenterology Journal* 2022; **10**: 1028-9.

302. Lu Z, Zhang L, Yao L, Gong D, Wu L, Xia M, et al. Assessment of the Role of Artificial Intelligence in the Association Between Time of Day and Colonoscopy Quality. *JAMA network open* 2023; **6**: e2253840.

303. Lui TKL, Guo C-G, Leung WK. Accuracy of artificial intelligence on histology prediction and detection of colorectal polyps: a systematic review and meta-analysis. *Gastrointestinal endoscopy* 2020; **92**: 11-22.e6.

304. Lui TKL, Leung WK. 1246 FINDINGS AT SURVEILLANCE COLONOSCOPY IN HIGH-RISK PATIENTS WITH PRIOR COMPUTED-ASSISTED DETECTION DURING INDEX COLONOSCOPY: A PROPENSITY SCORE MATCHING ANALYSIS. *Gastroenterology* 2024; **166**: S-293.

305. Lui TKL, Liu SHK, Leung K, Wu JT, Zauber AG, Leung WK. POTENTIAL IMPACTS OF COMPUTER-AIDED DETECTION OF COLORECTAL POLYPS ON COLONOSCOPY SURVEILLANCE INTERVALS. *Gastroenterology* 2022; **162**: S-1109.

306. Lui TKL, Dao H, Tsao SKK, Lam C, Mak LLY, Ko MKL, et al. COMPUTER-ASSISTED DETECTION VERSUS CONVENTIONAL COLONOSCOPY ON DETECTION AND MISS RATES OF PROXIMAL COLONIC LESIONS: A MULTI-CENTRE, RANDOMIZED, TANDEM COLONOSCOPY STUDY. *United European Gastroenterology Journal* 2022; **10**: 55.

307. Lui KLT, Liu SHK, Leung K, Wu JT, Zauber AG, Leung WK. The Impacts of Computer-Aided Detection of Colorectal Polyps on Subsequent Colonoscopy Surveillance Intervals: Simulation Study. *Journal of medical Internet research* 2023; **25**: e42665.

308. Lui TKL, Hang DV, Tsao SKK, Hui CKY, Mak LLY, Ko MKL, et al. Computer-assisted detection versus conventional colonoscopy for proximal colonic lesions: a multicenter, randomized, tandem-colonoscopy study. *Gastrointestinal endoscopy* 2023; **97**: 325-34.e1.

309. Ka-Luen Lui T, Hui C, Tsui VW, Cheung MK, Michael Ko KL, Yeung CK, et al. ARTIFICIAL INTELLIGENCE-ASSISTED REAL-TIME DETECTION REDUCES MISSED LESIONS DURING COLONOSCOPY: A RETROSPECTIVE AND PROSPECTIVE STUDY. *Gastrointestinal Endoscopy* 2020; **91**: AB234.

310. Ka-Luen Lui T, Lam C, Tsui V, Michael Ko KL, Wong SY, Loey Mak LY, et al. ARTIFICIAL INTELLIGENCE ASSISTED MONITORING OF EFFECTIVE WITHDRAWAL TIME AND ADENOMA DETECTION RATE OF INDIVIDUAL ENDOSCOPIST. *Gastrointestinal Endoscopy* 2024; **99**: AB2-AB3.

311. Ka-Luen Lui T, Lam C, Tsui V, Wong SY, Kevin Liu SH, Hui C, et al. COMPUTER-ASSISTED DETECTION WITH OR WITHOUR ENDOCUFF ON DETECTION OF COLORECTAL ADENOMA: A RANDOMIZED CONTROLLED TRIAL (INTERIM ANALYSIS). *Gastrointestinal Endoscopy* 2023; **97**: AB730-AB1.

312. Ka-Luen Lui T, Lam C, Tsui VW, Wan-Hin Hui R, Wong SY, To WPE, et al. PROSPECTIVE EVALUATION OF ARTIFICIAL INTELLIGENCE-ASSISTED MONITORING OF EFFECTIVE WITHDRAWAL TIME VERSUS STANDARD WITHDRAWAL TIME ON ADENOMA PER COLONOSCOPY. *Gastrointestinal Endoscopy* 2025; **101**: S31 EP - S2.

313. Lui TKL, Ko MKL, To EWP, Leung WK. Surveillance findings in high-risk patients after baseline computer-assisted detection colonoscopy: a propensity score matching analysis. *Gastrointestinal Endoscopy* 2025.

314. Luo Y, Zhang Y, Liu M, Lai Y, Liu P, Wang Z, et al. Artificial Intelligence-Assisted Colonoscopy for Detection of Colon Polyps: a Prospective, Randomized Cohort Study. *Journal of gastrointestinal surgery : official journal of the Society for Surgery of the Alimentary Tract* 2021; **25**: 2011-8.

315. Lwin WP, Ichimasa K, Kudo S-E, Kouyama Y, Okumura T, Maeda Y, et al. Clinical significance of computer-aided quality assessment systems in colonoscopy: a comprehensive review. *Clinical endoscopy* 2025.

316. Ma Y, Wen Y, Lu C, Liao J. Efficacy and safety of computer-aided detection(CADe) in colonoscopy for colorectal neoplasia detection: A meta-analysis. *Chinese Journal of Evidence-Based Medicine* 2024; **24**: 1270 EP - 7.

317. Maan S, Agrawal R, Singh S, Thakkar S. Artificial Intelligence in Endoscopy Quality Measures. *Gastrointestinal Endoscopy Clinics of North America* 2025; **35**: 431 EP - 44.

318. Maas MHJ, Neumann H, Shirin H, Jacob H, Katz LH, Benson A, et al. A NOVEL COMPUTER-AIDED POLYP DETECTION SYSTEM in DAILY CLINICAL CARE: AN INTERNATIONAL MULTICENTER, RANDOMIZED, TANDEM TRIAL. *United European Gastroenterology Journal* 2023; **11**: 248-9.

319. Maida M, Marasco G, Spadaccini M, Facciorusso A, Sinagra E, Hassan C. EFFECTIVENESS OF ARTIFICIAL INTELLIGENCE FOR COLONOSCOPY ON ADENOMA AND POLYP MISS RATE: A METAANALYSIS OF TANDEM RCTS. *Digestive and Liver Disease* 2023; **55**: S151-S2.

320. Maida M, Marasco G, Maas MHJ, Ramai D, Spadaccini M, Sinagra E, et al. Effectiveness of artificial intelligence assisted colonoscopy on adenoma and polyp miss rate: A meta-analysis of tandem RCTs. *Digestive and Liver Disease* 2025; **57**: 169 EP - 75.

321. Makar J, Abdelmalak J, Con D, Hafeez B, Garg M. Use of Artificial Intelligence Improves Colonoscopy Performance in Adenoma Detection: A Systematic Review and Meta-Analysis. *Gastrointestinal endoscopy* 2024.

322. Malik S, Loganathan P, Jason G, Mohan B, Kothari S. Is Adenoma Detection Any Different From Polyp Detection With Artificial Intelligence in Colonoscopy? A Meta-Analysis of Randomized Controlled Data. *American Journal of Gastroenterology* 2024; **119**: S186.

323. Mangas-Sanjuan C, De Castro L, Cubiella J, Diez-Redondo P, Suarez A, Pellise M, et al. ROLE OF ARTIFICIAL INTELLIGENCE IN COLONOSCOPY DETECTION OF ADVANCED LESIONS: A RANDOMIZED TRIAL. *Gastrointestinal Endoscopy* 2023; **97**: AB727-AB8.

324. Mangas-Sanjuan C, De-Castro L, Cubiella J, Diez Redondo P, Suarez Gonzalez A, Pellise Urquiza M, et al. ROLE of ARTIFICIAL INTELLIGENCE in COLONOSCOPY DETECTION of ADVANCED LESIONS. *United European Gastroenterology Journal* 2023; **11**: 250-1.

325. Mansour NM. Artificial Intelligence in Colonoscopy. *Current gastroenterology reports* 2023; **25**: 122-9.

326. McGrath BS, Borgaonkar M, McGrath GS. DOES AI INFLUENCE ADENOMA DETECTION RATES IN FIT-POSITIVE PATIENTS. *Journal of the Canadian Association of Gastroenterology* 2025; **8**: i48 EP - i9.

327. Mehta A, Kumar H, Yazji K, Wireko AA, Sivanandan Nagarajan J, Ghosh B, et al. Effectiveness of artificial intelligence-assisted colonoscopy in early diagnosis of colorectal cancer: a systematic review. *International journal of surgery (London, England)* 2023; **109**: 946-52.

328. Mekritthikrai K, Aniwan S, Tiankanon K, Kongtub N, Wisedopas N, Piyachaturawat P, et al. THE PERFORMANCE OF REAL-TIME COMPUTER-AIDED CHARACTERIZATION OF COLORECTAL NEOPLASIA IN SCREENING COLONOSCOPY: A PROSPECTIVE STUDY. *Gastrointestinal Endoscopy* 2022; **95**: AB212.

329. Melquist SJ, Meyer C, Kennedy KF, Desai M, Srinivasan S, Repici A, et al. CAUSES OF FALSE POSITIVE DETECTIONS BY CADE DURING REAL-TIME COLONOSCOPY AND ITS CLINICAL IMPACT: A SYSTEMATIC REVIEW AND META-ANALYSIS. *Gastroenterology* 2022; **162**: S-843.

330. Milluzzo SM, Cesaro P, Hassan C, Pesatori EV, Piccirelli S, Catino F, et al. ID: 3522041 INCREMENTAL YIELD OF ARTIFICIAL INTELLIGENCE IN FOLLOW-UP SCREENING COLONOSCOPIES - AN INTERIM ANALYSIS. *Gastrointestinal Endoscopy* 2021; **93**: AB187-AB8.

331. Milluzzo SM, Cesaro P, Hassan C, Pesatori EV, Piccirelli S, Catino F, et al. Incremental Yield Of Artificial Intelligence In Followup Screening Colonoscopies: An Interim Analysis. *Digestive and Liver Disease* 2021; **53**: S119.

332. Milluzzo SM, Cesaro P, Hassan C, Pesatori EV, Piccirelli S, Catino F, et al. Incremental yield of artificial intelligence in follow-up screening colonoscopies-an interim analysis on 432 patients. *United European Gastroenterology Journal* 2021; **9**: 796-7.

333. Milluzzo SM, Cesaro P, Hassan C, Pesatori EV, Piccirelli S, Catino F, et al. Incremental yield of artificial intelligence in follow-up screening colonoscopies-aninterim analysis. *Endoscopy* 2021; **53**: S51.

334. Minegishi Y, Kudo S-E, Miyata Y, Nemoto T, Mori K, Misawa M, et al. Comprehensive Diagnostic Performance of Real-Time Characterization of Colorectal Lesions Using an Artificial Intelligence-Assisted System: A Prospective Study. *Gastroenterology* 2022; **163**: 323-5.e3.

335. Ming Yen Koh GE, Ng B, Khoo N, Ladlad J, Tan C, Aw D, et al. REAL-WORLD ASSESSMENT OF THE EFFICACY OF COMPUTER-ASSISTED DIAGNOSIS (CADX) IN COLONOSCOPY - A COHORT STUDY IN SINGAPORE. *Gastrointestinal Endoscopy* 2024; **99**: AB38.

336. Misawa M, Kudo S, Miyata Y, Minegishi Y, Mori Y, Maeda Y, et al. A PROSPECTIVE STUDY OF REAL-TIME USE OF COMPUTER-AIDED CHARACTERIZATION FOR COLORECTAL LESIONS. *United European Gastroenterology Journal* 2022; **10**: 843-4.

337. Misawa M, Kudo S, Miyata Y, Minegishi Y, Mori Y, Takashina Y, et al. A PROSPECTIVE STUDY OF REAL-TIME COMPUTER-AIDED CHARACTERIZATION OF COLORECTAL LESIONS: DIAGNOSTIC PERFORMANCE AND IMPACT ON HUMAN DIAGNOSIS. *Gastrointestinal Endoscopy* 2022; **95**: AB230.

338. Miyaguchi K, Tsuzuki Y, Hirooka N, Matsumoto H, Ohgo H, Nakamoto H, et al. Artificial intelligence-assisted linked color imaging versus linked color imaging for colorectal adenoma detection: the first randomized controlled trial. 2024.

339. Mizukami K, Fushimi E, Sagami R, Abe T, Sato T, Terashi S, et al. Usefulness of AI-Equipped Endoscopy for Detecting Colorectal Adenoma during Colonoscopy Screening: Confirm That Colon Neoplasm Finely Can Be Identified by AI without Overlooking Study (Confidential Study). *Journal of clinical medicine* 2023; **12**.

340. Mohan BP, Facciorusso A, Khan SR, Chandan S, Kassab LL, Gkolfakis P, et al. Real-time computer aided colonoscopy versus standard colonoscopy for improving adenoma detection rate: A meta-analysis of randomized-controlled trials. *EClinicalMedicine* 2020; **29**: 100622.

341. Mohan BP, Khan SR, Kassab LL, Ponnada S, Dulai PS, Kochhar GS. Accuracy of convolutional neural network-based artificial intelligence in diagnosis of gastrointestinal lesions based on endoscopic images: A systematic review and meta-analysis. *Endoscopy international open* 2020; **8**: E1584-E94.

342. Moosvi Z, Shah S, Ortizo R, Samarasena J. Computer-Aided Polyp Detection during Colonoscopy: A Systematic Review and Meta-Analysis. *American Journal of Gastroenterology* 2020; **115**: S148.

343. Mori Y, Areia M, Correale L, Repici A, Bretthauer M, Sharma P, et al. COST-EFFECTIVENESS OF ARTIFICIAL INTELLIGENCE FOR SCREENING COLONOSCOPY. *Gastrointestinal Endoscopy* 2022; **95**: AB137.

344. Mori Y, Kudo S, East JE, Rastogi A, Bretthauer M, Misawa M, et al. Cost savings in colonoscopy with artificial intelligence-aided polyp diagnosis: An add-on analysis of a clinical trial. *United European Gastroenterology Journal* 2020; **8**: 735.

345. Mori Y, Kudo S, Misawa M, Kataoka S, Takeda K, Suzuki K, et al. 482 PERFORMANCE OF NON-EXPERT ENDOSCOPISTS IN OPTICAL BIOPSY OF DIMINUTIVE COLORECTAL POLYPS WITH REAL-TIME USE OF ARTIFICIAL INTELLIGENCE. *Gastrointestinal Endoscopy* 2019; **89**: AB89.

346. Mori Y, Kudo S, Misawa M, Saito Y, Ikematsu H, Hotta K, et al. Optical biopsy of diminutive colorectal polyps with real-time use of "artificial intelligence"-assisted endoscopy. *United European Gastroenterology Journal* 2018; **6**: A188-A9.

347. Mori Y, Kudo S, Misawa M, Takeda K, Ichimasa K, Ogawa Y, et al. Diagnostic yield of "artificial intelligence"-assisted endocytoscopy for colorectal polyps: A prospective study. *United European Gastroenterology Journal* 2017; **5**: A1.

348. Mori Y, Kudo SE, Misawa M, Hotta K, Ohtsuka K, Saito S, et al. Artificial intelligence-assisted colonoscopy for cancer recognition: A multicenter study designed to obtain regulatory approval. *Endoscopy* 2021; **53**: S58-S9.

349. Mori Y, Kudo S-E, East JE, Rastogi A, Bretthauer M, Misawa M, et al. Cost savings in colonoscopy with artificial intelligence-aided polyp diagnosis: an add-on analysis of a clinical trial (with video). *Gastrointestinal endoscopy* 2020; **92**: 905-11.e1.

350. Mori Y, Kudo S-E, Misawa M, Saito Y, Ikematsu H, Hotta K, et al. Real-Time Use of Artificial Intelligence in Identification of Diminutive Polyps During Colonoscopy: A Prospective Study. *Annals of internal medicine* 2018; **169**: 357-66.

351. Mori Y, Wang P, Loberg M, Misawa M, Repici A, Spadaccini M, et al. Impact of Artificial Intelligence on Colonoscopy Surveillance After Polyp Removal: A Pooled Analysis of Randomized Trials. *Clinical gastroenterology and hepatology : the official clinical practice journal of the American Gastroenterological Association* 2023; **21**: 949-59.e2.

352. Morimoto S, Yoshida S, Yongfei W, Koide T, Tamaki T, Saino M, et al. DEVELOPMENT OF COMPUTER-AIDED DIAGNOSTIC SYSTEMS FOCUSED ON THE JNET CLASSIFICATIONS FOR COLORECTAL LESIONS. *Gastrointestinal Endoscopy* 2025; **101**: S11 EP - S2.

353. Morimoto S, Tanaka H, Takehara Y, Yamamoto N, Tanino F, Kamigaichi Y, et al. Efficiency of Real-time Computer-aided Polyp Detection during Surveillance Colonoscopy: A Pilot Study. *Journal of the anus, rectum and colon* 2025; **9**: 127-33.

354. Mwango A, Akhtar TS, Abbas S, Abbasi DS, Khan A. Effect of artificial intelligence-aided colonoscopy on the adenoma detection rate: A systematic review. *International Journal of Gastrointestinal Intervention* 2024; **13**: 65 EP - 73.

355. Narimiti A, Mohamed S, Lee L. AI Assisted Colonoscopy Does Not Affect Mental Workload in Gastroenterologists. *American Journal of Gastroenterology* 2022; **117**: S184-S5.

356. Nayar KD, Yakout A, Nader B, Sanchez PS, Yan Y, Huang Y, et al. The Impact of Real Time Artificial Intelligence Enhanced Colonoscopy: A One Year Performance Review. *American Journal of Gastroenterology* 2024; **119**: S297 EP - S8.

357. Nazarian S, Glover B, Ashrafian H, Darzi A, Teare J. Diagnostic Accuracy of Artificial Intelligence and Computer-Aided Diagnosis for the Detection and Characterization of Colorectal Polyps: Systematic Review and Meta-analysis. *Journal of medical Internet research* 2021; **23**: e27370.

358. ClinicalTrials.gov. Quality Improvement Intervention in Colonoscopy Using Artificial Intelligence (NCT03622281), 2018. Available from: https://www.cochranelibrary.com/central/doi/10.1002/central/CN-01626126/full. Date accessed: Feb 25.

359. ClinicalTrials.gov. Computer Aided Detection of Polyps in the Colon (NCT03925337), 2019. Available from: https://www.cochranelibrary.com/central/doi/10.1002/central/CN-01931561/full. Date accessed: Feb 25.

360. ClinicalTrials.gov. Impact of Automatic Polyp Detection System on Adenoma Detection Rate (NCT03967756), 2019. Available from: https://www.cochranelibrary.com/central/doi/10.1002/central/CN-01933143/full. Date accessed: Feb 25.

361. ClinicalTrials.gov. A Randomized Controlled Multicenter Study of Artificial Intelligence Assisted Digestive Endoscopy (NCT04071678), 2019. Available from: https://www.cochranelibrary.com/central/doi/10.1002/central/CN-01983832/full. Date accessed: Feb 25.

362. ClinicalTrials.gov. Computer Aided Detection, Tandem Colonoscopy Study (NCT04074577), 2019. Available from: https://www.cochranelibrary.com/central/doi/10.1002/central/CN-01983913/full. Date accessed: Feb 25.

363. ClinicalTrials.gov. A Multicenter Study Evaluating the Effectiveness of Endo.Angel in Improving the Quality of Colonoscopy (NCT04102631), 2019. Available from: https://www.cochranelibrary.com/central/doi/10.1002/central/CN-01992195/full. Date accessed: Feb 25.

364. ClinicalTrials.gov. Artificial Intelligence-assisted Colonoscopy for Detection of Colon Polyps (NCT04126265), 2019. Available from: https://www.cochranelibrary.com/central/doi/10.1002/central/CN-01992842/full. Date accessed: Feb 25.

365. ClinicalTrials.gov. Artificial Intelligence-assisted Colonoscopy on Detection of Missed Proximal Lesions (NCT04294355), 2020. Available from: https://www.cochranelibrary.com/central/doi/10.1002/central/CN-02088736/full. Date accessed: Feb 25.

366. ClinicalTrials.gov. CADDIE Trial - Computer Aided Diagnosis and Detection for Intelligent Endoscopy (NCT04325815), 2020. Available from: https://www.cochranelibrary.com/central/doi/10.1002/central/CN-02091157/full. Date accessed: Feb 25.

367. ClinicalTrials.gov. Does AI-assisted Colonoscopy Improve Adenoma Detection in Screening Colonoscopy? (NCT04422548), 2020. Available from: https://www.cochranelibrary.com/central/doi/10.1002/central/CN-02125347/full. Date accessed: Feb 25.

368. ClinicalTrials.gov. Impact of Artificial Intelligence Genius® System-assisted Colonoscopy vs. Standard Colonoscopy (COLO-GENIUS; NCT04440865), 2020. Available from: https://www.cochranelibrary.com/central/doi/10.1002/central/CN-02133774/full. Date accessed: Feb 25.

369. ClinicalTrials.gov. Assessing the Additional Neoplasia Yield of Computer-aided Colonoscopy in a Screening Setting (NCT04441580), 2020. Available from: https://www.cochranelibrary.com/central/doi/10.1002/central/CN-02180815/full. Date accessed: Feb 25.

370. ClinicalTrials.gov. A Single Center Study on Comparing the Different Function of EndoAngel in Improving the Quality of Colonscopy (NCT04453956), 2020. Available from: https://www.cochranelibrary.com/central/doi/10.1002/central/CN-02134061/full. Date accessed: Feb 25.

371. ClinicalTrials.gov. Application of AI in Polypectomy (NCT04485715), 2020. Available from: https://www.cochranelibrary.com/central/doi/10.1002/central/CN-02137408/full. Date accessed: Feb 25.

372. ClinicalTrials.gov. A Study to Evaluate the Safety and Efficacy of the Use of ME-APDS During Colonoscopy (NCT04640792), 2020. Available from: https://www.cochranelibrary.com/central/doi/10.1002/central/CN-02205850/full. Date accessed: Feb 25.

373. ClinicalTrials.gov. Usefulness of GI-GENIUS in FIT-based Colorectal Cancer Screening Program (NCT04673136), 2020. Available from: https://www.cochranelibrary.com/central/doi/10.1002/central/CN-02209431/full. Date accessed: Feb 25.

374. ClinicalTrials.gov. Impact of Artificial Intelligence (AI) on Adenoma Detection During Colonoscopy in FIT+ Patients (NCT04691401), 2020. Available from: https://www.cochranelibrary.com/central/doi/10.1002/central/CN-02209808/full. Date accessed: Feb 25.

375. ClinicalTrials.gov. COLO-DETECT: can an Artificial Intelligence Device Increase Detection of Polyps During Colonoscopy? (NCT04723758), 2021. Available from: https://www.cochranelibrary.com/central/doi/10.1002/central/CN-02234398/full. Date accessed: Feb 25.

376. ClinicalTrials.gov. Comparison of Polyp Detection and False Alarm Rates in Water Exchange and Air Insufflation Colonoscopy (NCT04727814), 2021. Available from: https://www.cochranelibrary.com/central/doi/10.1002/central/CN-02234495/full. Date accessed: Feb 25.

377. ClinicalTrials.gov. Artificial Intelligence Performance in Colonoscopy in Daily Practice (NCT04837599), 2021. Available from: https://www.cochranelibrary.com/central/doi/10.1002/central/CN-02253653/full. Date accessed: Feb 25.

378. ClinicalTrials.gov. Effect of Real-time Computer-aided System (ENDO-AID) on Adenoma Detection in Endoscopist-in-training (NCT04838951), 2021. Available from: https://www.cochranelibrary.com/central/doi/10.1002/central/CN-02269473/full. Date accessed: Feb 25.

379. ClinicalTrials.gov. Study on the Use of Artificial Intelligence (Fujifilm) for Polyp Detection in Colonoscopy (NCT04894708), 2020. Available from: https://www.cochranelibrary.com/central/doi/10.1002/central/CN-02277730/full. Date accessed: Feb 25.

380. ClinicalTrials.gov. Evaluation of ArTificial Intelligence System (Gi-Genius) for adenoMa dEtection in Lynch Syndrome (NCT04909671), 2021. Available from: https://www.cochranelibrary.com/central/doi/10.1002/central/CN-02278073/full. Date accessed: Feb 25.

381. ClinicalTrials.gov. A Study on the Effectiveness of AI-assisted Colonoscopy in Improving the Effect of Colonoscopy Training for Trainees (NCT04912037), 2021. Available from: https://www.cochranelibrary.com/central/doi/10.1002/central/CN-02278121/full. Date accessed: Feb 25.

382. ClinicalTrials.gov. Artificial Intelligence Aid Systems in Colorectal Adenoma Detection (NCT04945044), 2021. Available from: https://www.cochranelibrary.com/central/doi/10.1002/central/CN-02290004/full. Date accessed: Feb 25.

383. ClinicalTrials.gov. ENDO-AID Assisted Tandem Colonoscopy RCT (NCT05013125), 2021. Available from: https://www.cochranelibrary.com/central/doi/10.1002/central/CN-02307130/full. Date accessed: Feb 25.

384. ClinicalTrials.gov. Early diAgnosis Real-Time Healthcare System for CANcer Trial (NCT05064124), 2021. Available from: https://www.cochranelibrary.com/central/doi/10.1002/central/CN-02340941/full. Date accessed: Feb 25.

385. ClinicalTrials.gov. Endocuff With or Without AI-assisted Colonoscopy (NCT05133544), 2021. Available from: https://www.cochranelibrary.com/central/doi/10.1002/central/CN-02345258/full. Date accessed: Feb 25.

386. ClinicalTrials.gov. The EYE Study Enhancing the Diagnostic Yield of Standard Colonoscopy by Artificial Intelligence Aided Endoscopy (NCT05139186), 2021. Available from: https://www.cochranelibrary.com/central/doi/10.1002/central/CN-02353346/full. Date accessed: Feb 25.

387. ClinicalTrials.gov. Artificial Intelligence Aid Systems and Endocuff in Colorectal Adenoma Detection (NCT05141773), 2021. Available from: https://www.cochranelibrary.com/central/doi/10.1002/central/CN-02353406/full. Date accessed: Feb 25.

388. ClinicalTrials.gov. Comparison of Colonoscopy Adenoma Detection Yield (NCT05158725), 2021. Available from: https://www.cochranelibrary.com/central/doi/10.1002/central/CN-02348934/full. Date accessed: Feb 25.

389. ClinicalTrials.gov. Artificial Intelligence in Colonic Polyp Detection (NCT05178095), 2021. Available from: https://www.cochranelibrary.com/central/doi/10.1002/central/CN-02355285/full. Date accessed: Feb 25.

390. ClinicalTrials.gov. Comparing Detection of Standard Colonoscopy, CAD-EYE and Combined CAD-EYE and G-EYE® Aided Colonoscopy (NCT05237310), 2022. Available from: https://www.cochranelibrary.com/central/doi/10.1002/central/CN-02367681/full. Date accessed: Feb 25.

391. ClinicalTrials.gov. Clinical Efficacy Evaluation of a Computer-aided Colonoscopy as Compared With the Standard Colonoscopy (NCT05240625), 2022. Available from: https://www.cochranelibrary.com/central/doi/10.1002/central/CN-02381531/full. Date accessed: Feb 25.

392. ClinicalTrials.gov. Gastroenterology Artificial INtelligence System for Detecting Colorectal Polyps (The GAIN Study; NCT05275556), 2022. Available from: https://www.cochranelibrary.com/central/doi/10.1002/central/CN-02374844/full. Date accessed: Feb 25.

393. ClinicalTrials.gov. A Prospective Randomized Study Comparing the Adenoma Detection Yield of SC, AI and Combined AI and G-EYE® (NCT05317351), 2022. Available from: https://www.cochranelibrary.com/central/doi/10.1002/central/CN-02388158/full. Date accessed: Feb 25.

394. ClinicalTrials.gov. A Dual Tandem Study - SC vs. CAD-EYE vs. CAD-EYE With G-EYE (NCT05318495), 2022. Available from: https://www.cochranelibrary.com/central/doi/10.1002/central/CN-02392298/full. Date accessed: Feb 25.

395. ClinicalTrials.gov. Evaluate the Effects of An AI System on Colonoscopy Quality of Novice Endoscopists (NCT05323279), 2022. Available from: https://www.cochranelibrary.com/central/doi/10.1002/central/CN-02392426/full. Date accessed: Feb 25.

396. ClinicalTrials.gov. Artificial Intelligence for Diminutive Polyp Characterization (NCT05391477), 2022. Available from: https://www.cochranelibrary.com/central/doi/10.1002/central/CN-02405334/full. Date accessed: Feb 25.

397. ClinicalTrials.gov. Combination of Artificial Intelligence and Mucosal Exposure Device to Enhance Colorectal Neoplasia Detection (NCT05414448), 2022. Available from: https://www.cochranelibrary.com/central/doi/10.1002/central/CN-02405903/full. Date accessed: Feb 25.

398. ClinicalTrials.gov. Impact of AI on Trainee ADR (NCT05423964), 2022. Available from: https://www.cochranelibrary.com/central/doi/10.1002/central/CN-02423057/full. Date accessed: Feb 25.

399. ClinicalTrials.gov. Adenoma Detection Rate in Water and Air Colonoscopy Using Computer-aided System (NCT05448300), 2022. Available from: https://www.cochranelibrary.com/central/doi/10.1002/central/CN-02423633/full. Date accessed: Feb 25.

400. ClinicalTrials.gov. Validating the Safety and Effectiveness of ENDOANGEL Lower Gastrointestinal Endoscope Image Auxiliary Diagnostic Software (NCT05481632), 2022. Available from: https://www.cochranelibrary.com/central/doi/10.1002/central/CN-02431453/full. Date accessed: Feb 25.

401. ClinicalTrials.gov. Artificial Intelligence for Leaving in Situ Colorectal Polyps (NCT05500248), 2022. Available from: https://www.cochranelibrary.com/central/doi/10.1002/central/CN-02456560/full. Date accessed: Feb 25.

402. ClinicalTrials.gov. A Multi Center Study Comparing the Efficacy of CAD EYE and the Standard of Care (White Light; NCT05523271), 2022. Available from: https://www.cochranelibrary.com/central/doi/10.1002/central/CN-02457075/full. Date accessed: Feb 25.

403. ClinicalTrials.gov. Prospective, Randomized Controlled Study to Evaluate the Effect of Artificial Intelligence Assisted Optical Diagnosis of Advanced Adenomas (NCT05568992), 2022. Available from: https://www.cochranelibrary.com/central/doi/10.1002/central/CN-02468948/full. Date accessed: Feb 25.

404. ClinicalTrials.gov. Comparison of the ENDOCUFF VISION® Endoscopy Cap Coupled With GI GENIUS™ Artificial Intelligence Compared to Each Device Alone in Improving Colonic Adenoma Detection Rate During Colonoscopy (NCT05594576), 2022. Available from: https://www.cochranelibrary.com/central/doi/10.1002/central/CN-02488091/full. Date accessed: Feb 25.

405. ClinicalTrials.gov. Real-time Computer-Aided Detection of Colonic Adenomas With NEC WISE VISION Endoscopy (NCT05611151), 2022. Available from: https://www.cochranelibrary.com/central/doi/10.1002/central/CN-02495311/full. Date accessed: Feb 25.

406. ClinicalTrials.gov. Real-Time Artificial Intelligence Assissted Colonoscopy to Identify and Classify Polyps (NCT05718193), 2023. Available from: https://www.cochranelibrary.com/central/doi/10.1002/central/CN-02520333/full. Date accessed: Feb 25.

407. ClinicalTrials.gov. EAGLE Trial CADDIE Artificial Intelligence Endoscopy (NCT05730192), 2023. Available from: https://www.cochranelibrary.com/central/doi/10.1002/central/CN-02523168/full. Date accessed: Feb 25.

408. ClinicalTrials.gov. Adenoma Detection Rate in Artificial Intelligence-assisted Colonoscopy (NCT05740137), 2023. Available from: https://www.cochranelibrary.com/central/doi/10.1002/central/CN-02526902/full. Date accessed: Feb 25.

409. ClinicalTrials.gov. AI-assisted Colonoscopy Report System In Improving Reporting Quality (NCT05829590), 2023. Available from: https://www.cochranelibrary.com/central/doi/10.1002/central/CN-02555589/full. Date accessed: Feb 25.

410. ClinicalTrials.gov. Accuracy of Endo-aid in Consecutive Patients Referred for Colonoscopy (NCT05862948), 2023. Available from: https://www.cochranelibrary.com/central/doi/10.1002/central/CN-02561490/full. Date accessed: Feb 25.

411. ClinicalTrials.gov. Nationwide Study of Artificial Intelligence in Adenoma Detection for Colonoscopy (NCT05870332), 2023. Available from: https://www.cochranelibrary.com/central/doi/10.1002/central/CN-02564727/full. Date accessed: Feb 25.

412. ClinicalTrials.gov. Efficacy of Artificial Intelligence-assisted Colonic Polyp Detection System (NCT05941689), 2023. Available from: https://www.cochranelibrary.com/central/doi/10.1002/central/CN-02580020/full. Date accessed: Feb 25.

413. ClinicalTrials.gov. Safety and Efficacy of the Olympus CADe System in Real-time Colonoscopy (NCT05943288), 2023. Available from:

https://www.cochranelibrary.com/central/doi/10.1002/central/CN-02582700/full. Date accessed: Feb 25.

414. ClinicalTrials.gov. CAD-EYE System for the Detection of Neoplastic Lesions in Patients With Lynch Syndrome (NCT05963191), 2023. Available from: https://www.cochranelibrary.com/central/doi/10.1002/central/CN-02588593/full. Date accessed: Feb 25.

415. ClinicalTrials.gov. Real-Time CAD for Colonic Neoplasia: a RCT (NCT05963724), 2023. Available from: https://www.cochranelibrary.com/central/doi/10.1002/central/CN-02585159/full. Date accessed: Feb 25.

416. ClinicalTrials.gov. Artificial Intelligence in the Detection of Right Sided Colonic Polyp in Different Operator Experience (NCT05990218), 2023. Available from: https://www.cochranelibrary.com/central/doi/10.1002/central/CN-02588536/full. Date accessed: Feb 25.

417. ClinicalTrials.gov. Artificial Intelligence to Implement Cost-saving Strategies for Colonoscopy Screening Based on in Vivo Prediction of Polyp Histology (NCT06041945), 2023. Available from: https://www.cochranelibrary.com/central/doi/10.1002/central/CN-02602116/full. Date accessed: Feb 25.

418. ClinicalTrials.gov. Computer Aided Diagnosis (CADx) for Colorectal Polyps Resect-and-Discard Strategy (NCT06062095), 2023. Available from: https://www.cochranelibrary.com/central/doi/10.1002/central/CN-02602213/full. Date accessed: Feb 25.

419. ClinicalTrials.gov. Comparing CADe Software for Enhanced Polyp Detection (NCT06077435), 2023. Available from: https://www.cochranelibrary.com/central/doi/10.1002/central/CN-02603492/full. Date accessed: Feb 25.

420. ClinicalTrials.gov. Assessing the Additional Neoplasia Yield of Computer-aided Colonoscopy in Follow-up Patients in a Screening Setting (NCT06160466), 2023. Available from: https://www.cochranelibrary.com/central/doi/10.1002/central/CN-02651903/full. Date accessed: Feb 25.

421. ClinicalTrials.gov. Water Exchange Colonoscopy With Artificial Intelligence-assisted Detection (NCT06173258), 2023. Available from: https://www.cochranelibrary.com/central/doi/10.1002/central/CN-02633814/full. Date accessed: Feb 25.

422. ClinicalTrials.gov. Performance of Artificial Intelligence in Colonoscopy for Right Colon Polyp Detection (NCT06216405), 2024. Available from: https://www.cochranelibrary.com/central/doi/10.1002/central/CN-02676717/full. Date accessed: Feb 25.

423. ClinicalTrials.gov. Longterm Effectiveness of Artificial Intelligence-assisted Colonoscopy on Adenoma Recurrence (NCT06251700), 2024. Available from: https://www.cochranelibrary.com/central/doi/10.1002/central/CN-02680097/full. Date accessed: Feb 25.

424. ClinicalTrials.gov. Artificial Intelligence and Dysplasia Detection in Inflammatory Bowel Disease (EIIDISIA Study; NCT06281392), 2023. Available from: https://www.cochranelibrary.com/central/doi/10.1002/central/CN-02672635/full. Date accessed: Feb 25.

425. ClinicalTrials.gov. Effectiveness of Artificial Intelligence - Assisted Colonoscopy in Colorectal Neoplasms (NCT06469671), 2024. Available from: https://www.cochranelibrary.com/central/doi/10.1002/central/CN-02720252/full. Date accessed: Feb 25.

426. ClinicalTrials.gov. Autonomous Artificial Intelligence Versus AI Assisted Human Optical Diagnosis, 2024. Available from: https://www.cochranelibrary.com/central/doi/10.1002/central/CN-02741776/full. Date accessed: Jun 25.

427. ClinicalTrials.gov. Effect of the Computer Aided Diagnosis with Explainable Artificial Intelligence for Colon Polyp on Optical Diagnosis and Acceptance of Technology, 2024. Available from: https://www.cochranelibrary.com/central/doi/10.1002/central/CN-02781988/full. Date accessed: Jun 25.

428. ClinicalTrials.gov. Artificial Intelligence in Colonoscopy, 2024. Available from: https://www.cochranelibrary.com/central/doi/10.1002/central/CN-02766658/full. Date accessed: Jun 25.

429. ClinicalTrials.gov. The Yield of Artificial Intelligence (GI Genius) in Lynch Syndrome - a Randomized Tandem-colonoscopy Trial, 2024. Available from: https://www.cochranelibrary.com/central/doi/10.1002/central/CN-02779059/full. Date accessed: Jun 25.

430. ClinicalTrials.gov. Impact of Artificial Intelligence on Trainee Polyp Miss Rates, 2024. Available from: https://www.cochranelibrary.com/central/doi/10.1002/central/CN-02779920/full. Date accessed: Jun 25.

431. ClinicalTrials.gov. Evaluation of a CAM System for Colorectal Polyp Size Measurement, 2024. Available from: https://www.cochranelibrary.com/central/doi/10.1002/central/CN-02790933/full. Date accessed: Jun 25.

432. ClinicalTrials.gov. Artificial Intelligence in Colonoscopy, 2025. Available from: https://www.cochranelibrary.com/central/doi/10.1002/central/CN-02803716/full. Date accessed: Jun 25.

433. ClinicalTrials.gov. Artificial Intelligence-Assisted Colonoscopy in Colorectal Cancer Screening in a General Hospital, 2025. Available from: https://www.cochranelibrary.com/central/doi/10.1002/central/CN-02803846/full. Date accessed: Jun 25.

434. ClinicalTrials.gov. Artificial Intelligence-based Screening Models for Prevention and Early Detection of Colorectal Cancer, 2024. Available from: https://www.cochranelibrary.com/central/doi/10.1002/central/CN-02810437/full. Date accessed: Jun 25.

435. ClinicalTrials.gov. Artificial Intelligence in Endoscopic Diagnosis of Colorectal Polyps: A Prospective Randomized Study, 2025. Available from: https://clinicaltrials.gov/study/NCT06786793 Date accessed: Jun 25.

436. ClinialTrials.gov. Artificial Intelligence Predicts the Pathology and Endoscopic Classification of Colorectal Polyps During Colonoscopy, 2025. Available from: https://clinicaltrials.gov/study/NCT06773832 Date accessed: Jun 25.

437. ClinialTrials.gov. Impact of Computer Aided Detection on Trainee Polyp Miss Rates Using a Tandem Colonoscopy Design 2024. Available from: https://clinicaltrials.gov/study/NCT06676930. Date accessed: Jun 25.

438. ClinicalTrials.gov. A Prospective Evaluation of the Correlation Between Real Time Artificial Intelligence-derived Effective Withdrawal Time and Adenoma Detection Rate 2024. Available from: https://clinicaltrials.gov/study/NCT06345105. Date accessed: Jun 25.

439. ClinicalTrials.gov. Artificial Intelligence - Assisted Colonoscopy in Diagnosis of Colorectal Neoplasms: Russian Multicenter Randomised Open - Label Trial 2024. Available from: https://clinicaltrials.gov/study/NCT06469671 Date accessed: Jun 25.

440. ClinicalTrials.gov. Real-time Artificial Intelligence-based Endocytoscopic Diagnosis of Colorectal Neoplasms: a Single Center, Prospective Clinical Study 2024. Available from: https://clinicaltrials.gov/study/NCT06335654 Date accessed: Jun 25.

441. ClinicalTrials.gov. Evaluation of Artificial Intelligence for Adenoma Detection in Water Exchange Colonoscopy: the WEAID Randomized Controlled Trial (Water Exchange With Artificial Intelligence-assisted Detection) 2023. Available from: https://clinicaltrials.gov/study/NCT06173258 Date accessed: Jun 25.

442. ClinicalTrials.gov. Evaluation of the CAD-EYE System for the Detection of Colorectal Neoplastic Lesions in Patients With Lynch Syndrome 2023. Available from: https://clinicaltrials.gov/study/NCT05963191. Date accessed: Jun 25.

443. ClinicalTrials.gov. Research on the Auxiliary Diagnosis and Treatment System of Digestive Endoscopy Based on Artificial Intelligence: An Efficacy Study of Artificial Intelligence-assisted Colonic Polyp Detection System 2023. Available from: https://clinicaltrials.gov/study/NCT05941689 Date accessed: Jun 25.

444. ClinicalTrials.gov. Saving by Artificial Intelligence for Virtual Endoscopy Biopsy Artificial Intelligence to Implement Cost-saving Strategies for Colonoscopy Screening Based on in Vivo Prediction of Polyp Histology 2023. Available from: https://clinicaltrials.gov/study/NCT06041945 Date accessed: Jun 25.

445. ClinicalTrials.gov. Comparing CADe Software for Enhanced Polyp Detection: A Randomized Controlled Trial 2023. Available from: https://clinicaltrials.gov/study/NCT06077435. Date accessed: Jun 25.

446. ClinicalTrials.gov. Enhancing Polyp Detection: A Randomized Controlled Trial Comparing Combined Computer-Aid Detection and Polyp-Detecting Colonoscope Attachment to Computer-Aid Detection Alone in Patients Undergoing Colonoscopy 2023. Available from: https://clinicaltrials.gov/study/NCT06116864 Date accessed: Jun 25.

447. ClinicalTrials.gov. Computer Assisted Detection of Neoplasia During Colonoscopy Evaluation (CADeNCE) 2023. Available from: https://clinicaltrials.gov/study/NCT05888623 Date accessed: Jun 25.

448. ClinicalTrials.gov. Speech and Image Recognition Based System in Improving Reporting Quality During Colonoscopy 2023. Available from: https://clinicaltrials.gov/study/NCT05829590 Date accessed: Jun 25.

449. ClinicalTrials.gov. A Comprehensive Review of the Impact of a COmPuter-aIded reaL-time pOlyp deTection System on Adult Colonoscopy (COPILOT Study) - a Single Institution Adoption Experience 2023. Available from: https://clinicaltrials.gov/study/NCT05822895 Date accessed: Jun 25.

450. ClinicalTrials.gov. Evaluation of Artificial Intelligence for Detection of Gastrointestinal Lesions in Endoscopy (EAGLE) 2023. Available from: https://clinicaltrials.gov/study/NCT05730192 Date accessed: Jun 25.

451. ClinicalTrials.gov. Impact on Polyp Detection of a Computer Aided Detection System (CADEYE) Combined With a Balloon Mucosal Exposure Device (G-EYE 760R) in Individuals Participating in a Organized Colorectal Cancer Screening Program 2023. Available from: https://clinicaltrials.gov/study/NCT05829447. Date accessed: Jun 25.

452. ClinicalTrials.gov. A Clinical Trial of the Effectiveness and Safety of Software Assisting Diagnose the Intestinal Polyp Digestive Endoscopy by Analysis of Colonoscopy Medical Images From Electronic Digestive Endoscopy Equipment, a Prospective, Multicenter, Randomized Stratified Block, Incomplete Blind Setting, Para, 2023. Available from: https://clinicaltrials.gov/study/NCT05687318 Date accessed: Jun 25.

453. ClinicalTrials.gov. Efficacy of Artificial Intelligence in the Detection of Right Sided Colonic Polyp in Operators with Different Endoscopic Experience: a Randomized Control Trial 2023. Available from: https://clinicaltrials.gov/study/NCT05990218 Date accessed: Jun 25.

454. ClinicalTrials.gov. Real-time Computer-aided Polyp/Adenoma Detection During Screening Colonoscopy: a Single-center Crossover Trial 2023. Available from: https://clinicaltrials.gov/study/NCT05734820 Date accessed: Jun 25.

455. ClinicalTrials.gov. Using Artificial Intelligence-assisted Optical Polyp Diagnosis for Diminutive Colorectal Polyps, 2023. Available from: https://clinicaltrials.gov/study/NCT06059378 Date accessed: Jun 25.

456. ClinicalTrials.gov. Accuracy of Real Time Characterization in Artificial Intelligence-assisted Colonoscopy - A Prospective Quality Assurance Study 2023. Available from: https://clinicaltrials.gov/study/NCT05754229 Date accessed: Jun 25.

457. ClinicalTrials.gov. Comparison of Flat Colorectal Lesion Detection by Artificial Intelligence-assisted Colonoscopy Versus Endoscopists: AIChallenge - Medtronic 2023. Available from: https://clinicaltrials.gov/study/NCT05942677 Date accessed: Jun 25.

458. ClinicalTrials.gov. Efficacy of Real-Time Computer Aided-Detected of Colonic Neoplasia in an Underserved Population, A Randomized Controlled Trial 2023. Available from: https://clinicaltrials.gov/study/NCT05963724 Date accessed: Jun 25.

459. ClinicalTrials.gov. Safety and Efficacy of the Olympus Endoscopy Computer-Aided Detection (CADe) System in Detection of Colorectal Neoplasia's During Real-time Colonoscopy: A European Prospective, Multicenter, Randomized Controlled Trial (EuroCADe) 2023. Available from: https://clinicaltrials.gov/study/NCT05943288 Date accessed: Jun 25.

460. ClinicalTrials.gov. A Prospective Evaluation of the Correlation Between Artificial Intelligence-derived Effective Withdrawal Time and Adenoma Detection Rate 2023. Available from: https://clinicaltrials.gov/study/NCT06063720 Date accessed: Jun 25.

461. ClinicalTrials.gov. The Influence of Artificial Intelligence (AI) Assisted Polyp Detection (Discovery System) on Visual Gaze Patterns During Real-time Colonoscopy 2022. Available from: https://clinicaltrials.gov/study/NCT05619614 Date accessed: Jun 25.

462. ClinicalTrials.gov. Accuracy of CAD Eye in the Detection of Colonic Remaining Lesions After Endoscopic Mucosal Resection: a Pilot Study 2022. Available from: https://clinicaltrials.gov/study/NCT05542030 Date accessed: Jun 25.

463. ClinicalTrials.gov. Comparison of the ENDOCUFF VISION Endoscopy Cap Coupled with GI GENIUSTM Artificial Intelligence Compared to Each Device Alone in Improving Colonic Adenoma Detection Rate During Colonoscopy 2022. Available from: https://clinicaltrials.gov/study/NCT05594576 Date accessed: Jun 25.

464. ClinicalTrials.gov. A Multi-center Control Study to Determine the Efficacy of CADEYE in Detecting Colon Polyps in Comparison to Standard of Care 2022. Available from: https://clinicaltrials.gov/study/NCT05523271. Date accessed: Jun 25.

465. ClinicalTrials.gov. A Prospective Study to Evaluate the Diagnostic Accuracy of Computer-aided Diagnosis (CADx) System in Real-time Characterization of Colorectal Neoplasia 2022. Available from: https://clinicaltrials.gov/study/NCT05414383. Date accessed: Jun 25.

466. ClinicalTrials.gov. Real Time Computer-aided Diagnosis (CADx) of Diminutive Colorectal Polyps Using Artificial Intelligence 2022. Available from: https://clinicaltrials.gov/study/NCT05349110. Date accessed: Jun 25.

467. ClinicalTrials.gov. A Dual Tandem Study Comparing the Adenoma Detection and Miss-rate of SC to That of Artificial Intelligence (CAD-EYE) Aided Colonoscopy and to That of Artificial Intelligence (CAD-EYE) and G-EYE Aided Colonoscopy 2022. Available from: https://clinicaltrials.gov/study/NCT05318495 Date accessed: Jun 25.

468. ClinicalTrials.gov. Impact of Artificial Intelligence on Trainee Adenoma Detection Rate 2022. Available from: https://clinicaltrials.gov/study/NCT05423964 Date accessed: Jun 25.

469. ClinicalTrials.gov. Evaluate the Effects of An Artificial Intelligence System on Colonoscopy Quality of Novice Endoscopists: A Randomized Controlled Trial 2022. Available from: https://clinicaltrials.gov/study/NCT05323279 Date accessed: Jun 25.

470. ClinicalTrials.gov. Impact of Computer-aided Optical Diagnosis (CADx) in Predicting Histology of Diminutive Rectosigmoid Polyps: a Multicenter Prospective Trial 2022. Available from: https://clinicaltrials.gov/study/NCT05500248 Date accessed: Jun 25.

471. ClinicalTrials.gov. Performance Evaluation of CAD-EYE and SCALE-EYE for Detection, Classification, and Measurement of Colorectal Polyps: a Prospective Study 2022. Available from: https://clinicaltrials.gov/study/NCT05236790 Date accessed: Jun 25.

472. ClinicalTrials.gov. Artificial Intelligence (AI) Assisted Real-time Adenoma Detection and Classification During Colonoscopies 2022. Available from: https://clinicaltrials.gov/study/NCT05244278. Date accessed: Jun 25.

473. ClinicalTrials.gov. Dual Tandem Study Comparing the Adenoma Detection and Miss-rate of Standard Colonoscopy to That of Artificial Intelligence (CAD-EYE) and to That of Artificial Intelligence (CAD-EYE) and G-EYE Aided Colonoscopy *clinicaltrialsgov* 2022.

474. ClinicalTrials.gov. Synergistic Effect of G-Eye Balloon for Behind the Folds Visualization With Artificial Intelligence Assisted Polyp Detection (Discovery System) on Adenoma Detection Rate. 'Discovery III Study' 2022. Available from: https://clinicaltrials.gov/study/NCT05220345 Date accessed: Jun 25.

475. ClinicalTrials.gov. Efficacy and Cost-effectiveness of an Artificial Intelligence System (GI-Genius) on the Characterization of Diminutive Colorectal Polyps Within a Colorectal Cancer Screening Program: a Multicenter Randomized Controlled Trial (ODDITY Trial) 2022. Available from: https://clinicaltrials.gov/study/NCT05391477 Date accessed: Jun 25.

476. ClinicalTrials.gov. Questionnaire Study Concerning Artificial Intelligence and Its Application in (Gastrointestinal) Healthcare - Patients' and Physicians' Perspectives, 2022. Available from: https://clinicaltrials.gov/study/NCT05214625. Date accessed: Jun 25.

477. ClinicalTrials.gov. Real-time Computer-Aided Detection of Colonic Adenomas With NEC WISE VISION Endoscopy: Prospective, Randomized Clinical Performance Evaluation 2022. Available from: https://clinicaltrials.gov/study/NCT05611151 Date accessed: Jun 25.

478. ClinicalTrials.gov. Accuracy and Feasibility of CADx System for White Light Colonic Polyp Characterization 2022. Available from: https://clinicaltrials.gov/study/NCT05492656 Date accessed: Jun 25.

479. ClinicalTrials.gov. Adenoma Detection Using Real-Time Computer-Aided Colon Polyp Detection System to Compare Water Exchange and Air Insufflation - A Randomized Controlled Trial 2022. Available from: https://clinicaltrials.gov/study/NCT05448300 Date accessed: Jun 25.

480. ClinicalTrials.gov. Combination of Artificial Intelligence (ENDOAID) and Mucosal Exposure Device (ENDOCUFF) to Enhance Colorectal Neoplasia Detection: a Randomized Controlled Trial 2022. Available from: https://clinicaltrials.gov/study/NCT05414448 Date accessed: Jun 25.

481. ClinicalTrials.gov. Improving Polyp Detection Rate by Artificial Intelligence in Colonoscopy 2022. Available from: https://clinicaltrials.gov/study/NCT05322993 Date accessed: Jun 25.

482. ClinicalTrials.gov. Real-World Validation of an Artificial Intelligence Characterization Support (CADx) System for Prediction of Polyp Histology in Colonoscopy: A Prospective Multicentre Study 2021. Available from: https://clinicaltrials.gov/study/NCT05034185 Date accessed: Jun 25.

483. ClinicalTrials.gov. Computer-assisted Adenoma Detection Coloscopy With Endo-AID Artificial Intelligence System and Endocuff Versus Endocuff Assisted Colonoscopy: a Randomized Controlled Trial 2021. Available from: https://clinicaltrials.gov/study/NCT05141773 Date accessed: Jun 25.

484. ClinicalTrials.gov. Impact of Artificial Intelligence in Dysplasia Detection During Colonoscopy in Patients With Long-data Ulcerative Colitis: a Crossover Study 2021. Available from: https://clinicaltrials.gov/study/NCT05171634. Date accessed: Jun 25.

485. ClinicalTrials.gov. Artificial Intelligence in Colonic Polyp Detection 2021. Available from: https://clinicaltrials.gov/study/NCT05178095 Date accessed: Jun 25.

486. ClinicalTrials.gov. Endocuff With or Without Artificial Intelligence-assisted Colonoscopy in Detection of Colorectal Adenoma: a Randomized Colonoscopy Trial 2021. Available from: https://clinicaltrials.gov/study/NCT05133544 Date accessed: Jun 25.

487. ClinicalTrials.gov. The COMBO CAD Study: Characterization cOMparison Between twO CAD Systems 2021. Available from: https://clinicaltrials.gov/study/NCT05141409 Date accessed: Jun 25.

488. ClinicalTrials.gov. Early DiAgnosis Real-Time Healthcare System for CANcer Trial 2021. Available from: https://clinicaltrials.gov/study/NCT05064124 Date accessed: Jun 25.

489. ClinicalTrials.gov. Real-time Computer-aided Polyp Detection During Screening Colonoscopy Performed by Expert Endoscopists 2021. Available from: https://clinicaltrials.gov/study/NCT04915833 Date accessed: Jun 25.

490. ClinicalTrials.gov. Usefulness of the Endo-AID Artificial Intelligence System in the Detection of Colorectal Adenomas. a Randomized Controlled Trial 2021. Available from: https://clinicaltrials.gov/study/NCT04945044 Date accessed: Jun 25.

491. ClinicalTrials.gov. Colorectal Polyp Detection Comparing Computer Assisted Colonoscopy With Conventional Colonoscopy 2021. Available from: https://clinicaltrials.gov/study/NCT04979962. Date accessed: Jun 25.

492. ClinicalTrials.gov. Cross-sectional, Multi-center Study Comparing Diagnostic Performance Between the CAD EYE System and the Physician on Histological Prediction of Colonic Polyps in Screening of Colorectal Cancer by Colonoscopy 2021. Available from: https://clinicaltrials.gov/study/NCT04921488 Date accessed: Jun 25.

493. ClinicalTrials.gov. The CHANGE Study: Characterization Helping the Assessment of Colorectal Neoplasia in Gastrointestinal Endoscopy 2021. Available from: https://clinicaltrials.gov/study/NCT04884581 Date accessed: Jun 25.

494. ClinicalTrials.gov. Artificial Intelligence in the Characterization of Small and Diminutive Colorectal Polyps: A Prospective Study in a Clinical Setting Using CAD EYE 2021. Available from: https://clinicaltrials.gov/study/NCT04749277 Date accessed: Jun 25.

495. ClinicalTrials.gov. Computer Aided Polyp Detection (C3PO) Trial: A Multicenter International Trial Evaluating Diagnostic Accuracy of Artificial Intelligence System in Detecting Colon Polyps 2021. Available from: https://clinicaltrials.gov/study/NCT04777019. Date accessed: Jun 25.

496. ClinicalTrials.gov. COLO-DETECT: A Randomised Controlled Trial of Lesion Detection at Colonoscopy Using the GI Genius Artificial Intelligence Platform 2021. Available from: https://clinicaltrials.gov/study/NCT04723758 Date accessed: Jun 25.

497. ClinicalTrials.gov. Patient and Endoscopists' Experiences and Perceptions of Colonoscopy and New Technologies in Colonoscopy 2021. Available from: https://clinicaltrials.gov/study/NCT04747665 Date accessed: Jun 25.

498. ClinicalTrials.gov. Polyp Detection and False Alarm Rates by Computer-Aided Analysis of Videos of Withdrawal Phase of Colonoscopy in a Randomized Controlled Trial of Water Exchange Versus Air Insufflation, 2021. Available from: https://clinicaltrials.gov/study/NCT04727814 Date accessed: Jun 25.

499. ClinicalTrials.gov. A Pilot Study: Retrospective Evaluation of 3 Colonic Adenoma Detection Strategies During a Colonoscopy: Endoscopy Cap Associated With the Artificial Intelligence GI GENIUS TM System, the Artificial Intelligence GI GENIUS TM Alone and Colonoscopy Alone 2021. Available from: https://clinicaltrials.gov/study/NCT05080088 Date accessed: Jun 25.

500. ClinicalTrials.gov. Effect of Two Colonoscopy AI Systems for Colon Polyp Detection According to the False Positive Rates of the Systems: A Single-center Prospective Study 2021. Available from: https://clinicaltrials.gov/study/NCT05089071 Date accessed: Jun 25.

501. ClinicalTrials.gov. Comparison of Colonoscopy Adenoma Detection Yield of Standard Colonoscopy, Discovery Aided Colonoscopy, and Discovery and G-EYE Aided Colonoscopy 2021. Available from: https://clinicaltrials.gov/study/NCT05158725. Date accessed: Jun 25.

502. ClinicalTrials.gov. Assessment of Efficacy of ENDO-AID Assisted Colonoscopy in Adenoma Detection: a Single Centre Randomised Controlled Trial 2021. Available from: https://clinicaltrials.gov/study/NCT05013125 Date accessed: Jun 25.

503. ClinicalTrials.gov. A Study on the Effectiveness of Artificial Intelligence-assisted Colonoscopy in Improving the Effect of Colonoscopy Training for Trainees 2021. Available from: https://clinicaltrials.gov/study/NCT04912037 Date accessed: Jun 25.

504. ClinicalTrials.gov. Clinical vAliDation of ARTificial Intelligence in POlyp Detection 2020. Available from: https://clinicaltrials.gov/study/NCT04442607 Date accessed: Jun 25.

505. ClinicalTrials.gov. The AID Study 2: Artificial Intelligence for Colorectal Adenoma Detection 2 2020. Available from: https://clinicaltrials.gov/study/NCT04260321 Date accessed: Jun 25.

506. ClinicalTrials.gov. Impact of Computer-aided Optical Diagnosis (CAD) in Predicting Histology of Diminutive Rectosigmoid Polyps: a Multicenter Prospective Trial (Artificial Intelligence BLI Characterization - ABC Study) 2020. Available from: https://clinicaltrials.gov/study/NCT04607083 Date accessed: Jun 25.

507. ClinicalTrials.gov. A Prospective, Randomized, Single-blind, 2 x 2 Factorial Design Single Center Study Evaluating the Polyp Detection and Quality Monitoring Function of EndoAngel in Improving the Quality of Colonoscopy 2020. Available from: https://clinicaltrials.gov/study/NCT04453956 Date accessed: Jun 25.

508. ClinicalTrials.gov. Artificial Intelligence-assisted Colonoscopy Versus Conventional Colonoscopy for Missed Lesions in the Proximal Colon: A Prospective Multi-center Randomized Study in Asia 2020. Available from: https://clinicaltrials.gov/study/NCT04294355 Date accessed: Jun 25.

509. ClinicalTrials.gov. Prospective Randomized Study on the Use of Artificial Intelligence (Fujifilm) for Polyp Detection in Colonoscopy 2020. Available from: https://clinicaltrials.gov/study/NCT04894708 Date accessed: Jun 25.

510. ClinicalTrials.gov. Application of Artificial Intelligence in Colorectal Polypectomy, 2020. Available from: https://clinicaltrials.gov/study/NCT04485715 Date accessed: Jun 25.

511. ClinicalTrials.gov. Prospective Multicenter Study of Artificial Intelligence-assisted Quality Evaluation System for Colonoscopy 2020. Available from: https://clinicaltrials.gov/study/NCT04610177 Date accessed: Jun 25.

512. ClinicalTrials.gov. The CERTAIN Study: Combining Endo-cuff in a Randomized Trial for Artificial Intelligence Navigation 2020. Available from: https://clinicaltrials.gov/study/NCT04676308 Date accessed: Jun 25.

513. ClinicalTrials.gov. A Randomized Two Arm Multi-Center Study to Evaluate the Safety and Efficacy of the Use of Magentiq Eye's Automatic Polyp Detection System (ME-APDS) During Colonoscopy 2020. Available from: https://clinicaltrials.gov/study/NCT04640792 Date accessed: Jun 25.

514. ClinicalTrials.gov. Artificial Intelligence for Real-time Detection and Monitoring of Colorectal Polyps 2020. Available from: https://clinicaltrials.gov/study/NCT04586556. Date accessed: Jun 25.

515. ClinicalTrials.gov. Impact of Artificial Intelligence Genius System-assisted Colonoscopy vs. Standard Colonoscopy on Adenoma Detection Rate in Routine Practice: a Prospective Randomized Controlled Trial 2020. Available from: https://clinicaltrials.gov/study/NCT04440865 Date accessed: Jun 25.

516. ClinicalTrials.gov. Comparing the Number of False Activations Between Two Artificial Intelligence CADe Systems: the NOISE Study 2020. Available from: https://clinicaltrials.gov/study/NCT04399590 Date accessed: Jun 25.

517. ClinicalTrials.gov. Does AI-assisted Colonoscopy Improve Adenoma Detection in Screening Colonoscopy? A Multi-center Randomized Controlled 2020. Available from: https://clinicaltrials.gov/study/NCT04422548 Date accessed: Jun 25.

518. ClinicalTrials.gov. Development and Validation of a New Artificial Intelligence System for Automated Detection of Colorectal Polyps During Colonoscopy 2020. Available from: https://clinicaltrials.gov/study/NCT04359355 Date accessed: Jun 25.

519. ClinicalTrials.gov. Real Life AI in Polyp Detection 2020. Available from: https://clinicaltrials.gov/study/NCT04335318 Date accessed: Jun 25.

520. ClinicalTrials.gov. Improving Optical Diagnosis of Colorectal Polyps Using Computer-aided Diagnosis (CADx) and the BLI Adenoma Serrated International Classification (BASIC) 2020. Available from: https://clinicaltrials.gov/study/NCT04349787 Date accessed: Jun 25.

521. ClinicalTrials.gov. Artificial Intelligence Validation Trial for Polyp Detection: Pilot Study, 2020. Available from: https://clinicaltrials.gov/study/NCT04378660 Date accessed: Jun 25.

522. ClinicalTrials.gov. Artificial Intelligence-Assisted Real-time Detection of Missed Lesions During Colonoscopy: A Prospective Study 2020. Available from: https://clinicaltrials.gov/study/NCT04227795 Date accessed: Jun 25.

523. ClinicalTrials.gov. Resa Diagnostica Aggiuntiva Dell'Intelligenza Artificiale Nella Colonscopia (GENIAL COLONOSCOPY), Per lo Screening Del Carcinoma Colorettale 2020. Available from: https://clinicaltrials.gov/study/NCT04441580 Date accessed: Jun 25.

524. ClinicalTrials.gov. Polyp REcognition Assisted by a Device Interactive Characterization Tool - The PREDICT Study 2020. Available from: https://clinicaltrials.gov/study/NCT04589078 Date accessed: Jun 25.

525. ClinicalTrials.gov. Multi-Centre, Open-label, Randomised, Prospective Trial to Assess Efficacy and Safety of the CADDIE Artificial Intelligence System for Improving Endoscopic Detection of Colonic Polyps in Real-time 2020. Available from: https://clinicaltrials.gov/study/NCT04325815. Date accessed: Jun 25.

526. ClinicalTrials.gov. A Single Center Study on the Effectiveness and Safety of Polyp Detection and Polyp Classification With Artificial Intelligence 2019. Available from: https://clinicaltrials.gov/study/NCT04216901 Date accessed: Jun 25.

527. ClinicalTrials.gov. The AID Study: Artificial Intelligence for Colorectal Adenoma Detection 2019. Available from: https://clinicaltrials.gov/study/NCT04079478 Date accessed: Jun 25.

528. ClinicalTrials.gov. A Prospective, Randomized, Single-blind, Parallel-controlled Multicenter Study Evaluating the Effectiveness of Endo.Angel in Improving the Quality of Colonoscopy 2019. Available from: https://clinicaltrials.gov/study/NCT04102631. Date accessed: Jun 25.

529. ClinicalTrials.gov. Computer Aided Detection of Polyps in the Colon 2019. Available from: https://clinicaltrials.gov/study/NCT03925337. Date accessed: Jun 25.

530. ClinicalTrials.gov. Computer Aided Detection, Tandem Colonoscopy Study 2019. Available from: https://clinicaltrials.gov/study/NCT04074577 Date accessed: Jun 25.

531. ClinicalTrials.gov. Prospective, Randomized, Multicenter, Tandem Study Evaluating the Safety and Effectiveness of the CB-17-08 Augmented Endoscopy System for the Detection of Mucosal Colorectal Polyps in Adult Patients Undergoing Screening or Surveillance Colonoscopy for CRC 2019. Available from: https://clinicaltrials.gov/study/NCT03954548 Date accessed: Jun 25.

532. ClinicalTrials.gov. Computer-aided Detection With Deep Learning for Colorectal Adenoma During Colonoscopic Examination 2019. Available from: https://clinicaltrials.gov/study/NCT03842059 Date accessed: Jun 25.

533. ClinicalTrials.gov. In Vivo Computer-aided Prediction of Polyp Histology on White Light Colonoscopy 2018. Available from: https://clinicaltrials.gov/study/NCT03775811. Date accessed: Jun 25.

534. ClinicalTrials.gov. Deep-Learning for Automatic Polyp Detection During Colonoscopy 2018. Available from: https://clinicaltrials.gov/study/NCT03637712 Date accessed: Jun 25.

535. ClinicalTrials.gov. Validating the Performance of Artificial Intelligence in Identifying Polyps in Real-world Colonoscopy 2018. Available from: https://clinicaltrials.gov/study/NCT03761771 Date accessed: Jun 25.

536. ClinicalTrials.gov. Computer-aided Classification of Colorectal Polyp by Using Linked Colour Imaging 2017. Available from: https://clinicaltrials.gov/study/NCT03359343 Date accessed: Jun 25.

537. ClinicalTrials.gov. Computer Aided Diagnosis of Colorectal Neoplasms During Colonoscopic Examination 2017. Available from: https://clinicaltrials.gov/study/NCT03069833 Date accessed: Jun 25.

538. ClinicalTrials.gov. Computer-assisted Detection of Colonic Polyps 2016. Available from: https://clinicaltrials.gov/study/NCT02838888 Date accessed: Jun 25.

539. Nguyen D, Chang P, Kong N, Wang SJ, Sharma N, Ong J, et al. AI-Assisted Colonoscopy May Increase ADR in Right Side of Colon in Gastroenterology Fellows: A Randomized Control Trial. *American Journal of Gastroenterology* 2024; **119**: S210.

540. Nguyen D, Chang P, Wang S, Sharma N, Bui A, Kong N, et al. Artificial Intelligence-Assisted Colonoscopy Improves ADR of Gastroenterology Fellows: Results of a Prospective Cohort Study. *American Journal of Gastroenterology* 2023; **118**: S266.

541. Overview of Medical Research in the Netherlands (NL-OMON). Discovery II Study (NL-OMON22821), 2020. Available from: https://www.cochranelibrary.com/central/doi/10.1002/central/CN-02240692/full. Date accessed: Feb 25.

542. Overview of Medical Research in the Netherlands (NL-OMON). Discovery: pentax* Computer-aided Detection to Improve Adenoma Detection in a Real-time Setting - The Discovery II Study. A randomized clinical trial (NL-OMON49600), 2020. Available from: https://www.cochranelibrary.com/central/doi/10.1002/central/CN-02719119/full. Date accessed: Feb 25.

543. Overview of Medical Research in the Netherlands (NL-OMON). Randomized Two Arm Multi-Center Study to Evaluate the Safety and Efficacy of the Use of Magentiq Eye's Automatic Polyp Detection System (ME-APDS) During Colonoscopy (NL-OMON50965), 2021. Available from: https://www.cochranelibrary.com/central/doi/10.1002/central/CN-02719399/full. Date accessed: Feb 25.

544. Overview of Medical Research in the Netherlands (NL-OMON). Effect of a computer-aided detection system (CAD EYE) on adenoma detection in patients with Lynch syndrome: an international, multicenter parallel randomized controlled trial (NL-OMON51017), 2021. Available from: https://www.cochranelibrary.com/central/doi/10.1002/central/CN-02719700/full. Date accessed: Feb 25.

545. Norwood DA, Cartee AK, Herman TT, Russ KB, Sarkis F, Sanchez-Luna SA, et al. LEAVING NO STONE UNTURNED - ROLE OF ARTIFICIAL INTELLIGENCE IN DETECTION OF SMALLER LESIONS DURING COLONOSCOPY. *Gastroenterology* 2024; **166**: S-889.

546. Norwood DA, Thakkar S, Cartee A, Sarkis F, Torres-Herman T, Montalvan-Sanchez EE, et al. Performance of Computer-Aided Detection and Quality of Bowel Preparation: A Comprehensive Analysis of Colonoscopy Outcomes. *Digestive Diseases and Sciences* 2024; **69**: 3681 EP - 9.

547. Okiye P, Soudan R, Soudan O, Shahsavari D, Adu-Gyamfi KO, Alkaddour A. A Comparison of Distal Attachment Devices, Artificial Intelligence, and Standard Colonoscopy for Adenoma Detection Rate and Withdrawal Times: Advantage or Hindrance. *American Journal of Gastroenterology* 2024; **119**: S647.

548. Oleksiw M, Djinbachian R, Rex DK, Hassan C, Pohl H, Von Renteln D. ARTIFICIAL INTELLIGENCE AND ENDOSCOPIST DIAGNOSTIC AGREEMENT AS A FRAMEWORK FOR COLORECTAL POLYP OPTICAL DIAGNOSIS IMPLEMENTATION. *Gastrointestinal Endoscopy* 2025; **101**: S47 EP - S8.

549. Oleksiw M, Djinbachian R, Von Renteln D. IMPLEMENTATION STRATEGIES TO OPTIMIZE DIAGNOSTIC ACCURACY OF COMPUTER-ASSISTED OPTICAL POLYP DIAGNOSIS. *Journal of the Canadian Association of Gastroenterology* 2025; **8**: i5.

550. O'Mara M, Galati J, Gross S, Pochapin M, Gross SA. Comparing the Adenoma Detection Rate of Endocuff-Assisted Colonoscopy (EAC) Against Combined Artificial Intelligence and Endocuff-Assisted Colonoscopy (AEAC). *American Journal of Gastroenterology* 2022; **117**: S225-S6.

551. Okumura T, Imai K, Misawa M, Kudo S-E, Hotta K, Ito S, et al. Evaluating false-positive detection in a computer-aided detection system for colonoscopy. *Journal of gastroenterology and hepatology* 2024; **39**: 927-34.

552. Olabintan O, Halvorsen N, Baddeley R, Kudo SE, Barua I, Misawa M, et al. ENVIRONMENTAL IMPACT OF OPTICAL DIAGNOSIS BY ARTIFICIAL INTELLIGENCE IN COLONOSCOPY: A PROSPECTIVE TRIAL. *Gastrointestinal Endoscopy* 2024; **99**: AB25.

553. Ortiz O, Daca-Alvarez M, Rivero L, Huneburg R, Sanchez VA, Nattermann J, et al. EVALUATION OF ARTIFICIAL INTELLIGENCE-ASSISTED COLONOSCOPY FOR ADENOMA DETECTION IN LYNCH SYNDROME: A MULTICENTRE RANDOMIZED CONTROLLED TRIAL (TIMELY STUDY). *Gastrointestinal Endoscopy* 2024; **99**: AB22.

554. Ortiz O, Rivero-Sánchez L, Gimeno-Garcia A, Vicente JL, Martínez RJ, Ricciardiello L, et al. Evaluation of Artificial Intelligence-Assisted Colonoscopy for Adenoma Detection in Lynch Syndrome: a multicentre randomized controlled trial (Timely study). 2024; **56**: S56.

555. Orzeszko Z, Gach T, Bogacki P, Markowska B, Solecki R, Szura M. Effect of artificial intelligence implementation to the latest generation 4K colonoscopy. *Polski przeglad chirurgiczny* 2024; **96**: 24 EP - 30.

556. Pagador JB, Peralta LFS, Del Nozal JB, Fernandez HL, Rodriguez AN, Pinon PD, et al. Identification of clinical needs for the improvement of ai-assisted colonoscopy cad systems. *British Journal of Surgery* 2025; **112**: ii6.

557. Pal P, Pooja K, Nabi Z, Gupta R, Tandan M, Rao GV, et al. Artificial intelligence in endoscopy related to inflammatory bowel disease: A systematic review. *Indian journal of gastroenterology : official journal of the Indian Society of Gastroenterology* 2024; **43**: 172-87.

558. Pan H, Cai M, Liao Q, Jiang Y, Liu Y, Zhuang X, et al. Artificial Intelligence-Aid Colonoscopy Vs. Conventional Colonoscopy for Polyp and Adenoma Detection: A Systematic Review of 7 Discordant Meta-Analyses. *Frontiers in medicine* 2021; **8**: 775604.

559. Pannala R, Krishnan K, Melson J, Parsi MA, Schulman AR, Sullivan S, et al. Artificial intelligence in gastrointestinal endoscopy. *VideoGIE : an official video journal of the American Society for Gastrointestinal Endoscopy* 2020; **5**: 598-613.

560. Park DK, Kim EJ, Im JP, Lim H, Lim YJ, Byeon JS, et al. A prospective multicenter randomized controlled trial on artificial intelligence assisted colonoscopy for enhanced polyp detection. *Scientific reports* 2024; **14**: 25453.

561. Pasam RT, Mohan B, Chava S, Adler D. ADENOMA DETECTION RATES WITH COMPUTER AIDED COLONOSCOPY AND DISTAL ATTACHMENT MUCOSAL EXPOSURE DEVICES - A SYSTEMATIC REVIEW AND NETWORK META-ANALYSIS. *Gastrointestinal Endoscopy* 2023; **97**: AB718-AB9.

562. Patel H, Mori Y, Radadiya D, Spadaccini M, Repici A, Rex D, et al. AI IN COLONOSCOPY FOR THE DETECTION OF COLORECTAL NEOPLASIA: A META-ANALYSIS OF RANDOMIZED AND NON-RANDOMIZED STUDIES. *Gastrointestinal Endoscopy* 2024; **99**: AB30-AB1.

563. Patel H, Radadiya D, Spadaccini M, Rizkala T, Nathani P, Velji-Ibrahim J, et al. BENEFITS AND HARMS OF INCORPORATING AI DURING COLONOSCOPY FOR TRAINEES: A SYSTEMATIC REVIEW AND META-ANALYSIS OF PUBLISHED LITERATURE. *Gastrointestinal Endoscopy* 2024; **99**: AB24-AB5.

564. Patel H, Radadiya D, Srinivasan S, Chandrasekar VT, Desai M, Repici A, et al. COMPARISON OF MEAN ADENOMA PER COLONOSCOPY USING ARTIFICIAL INTELLIGENCE SYSTEMS VS STANDARD COLONOSCOPY: A SYSTEMATIC REVIEW AND META-ANALYSIS OF RANDOMIZED CONTROLLED STUDIES. *Gastrointestinal Endoscopy* 2023; **97**: AB719-AB20.

565. Patel H, Radadiya D, Srinivasan S, Nathani P, Chandrasekar VT, Desai M, et al. EXISTING ARTIFICIAL INTELLIGENCE SYSTEMS DO NOT IMPROVE SESSILE SERRATED LESION DETECTION: A META-ANALYSIS OF RANDOMIZED CONTROLLED STUDIES. *Gastrointestinal Endoscopy* 2023; **97**: AB714.

566. Patel HK, Radadiya D, Nathani P, Spadaccini M, Velji-Ibrahim J, Rizkala T, et al. AI IN COLONOSCOPY BENEFITS THOSE ENDOSCOPISTS WITH LOW BASELINE ADR: AN AGGREGATE META-ANALYSIS AND META-REGRESSION OF ENDOSCOPIST-LEVEL DATA. *Gastroenterology* 2024; **166**: S-1487.

567. Patel HK, Radadiya D, Srinivasan S, Nathani P, Desai M, Chandrasekar VT, et al. COMPARISON OF ADENOMA DETECTION RATE IN SCREENING OR SURVEILLANCE COLONOSCOPY USING ARTIFICIAL INTELLIGENCE VS STANDARD COLONOSCOPY: A META-ANALYSIS OF RANDOMIZED CONTROLLED STUDIES. *Gastroenterology* 2023; **164**: S-1180.

568. Patel M, Gulati S, O'Neil S, Wilson N, Williams S, Charles-Nurse S, et al. Artificial intelligence increases adenoma detection even in 'high-detector' colonoscopy: Early evidence for human: machine interaction. *Gut* 2021; **70**: A70-A1.

569. Patel HK, Mori Y, Hassan C, Rizkala T, Radadiya DK, Nathani P, et al. Lack of Effectiveness of Computer Aided Detection for Colorectal Neoplasia: A Systematic Review and Meta-Analysis of Nonrandomized Studies. *Clinical gastroenterology and hepatology : the*

official clinical practice journal of the American Gastroenterological Association 2024; **22**: 971-80.e15.

570. Patel HK, Chudasama J, Radadiya D, Khalaf K, Li H, Spadaccini M, et al. ROLE OF ENDOSCOPIST ON PERFORMANCE OF ARTIFICIAL INTELLIGENCE IN NEOPLASIA DETECTION DURING COLONOSCOPY: METAANALYSIS AND METAREGRESSION OF ENDOSCOPIST LEVEL DATA FROM 25 STUDIES. *Gastrointestinal Endoscopy* 2025; **101**: S52.

571. Patel K, VanLeer-Greenberg B. Artificial Intelligence in Colonoscopy in the Community Setting. *American Journal of Gastroenterology* 2023; **118**: S256.

572. Patel H, Yuichi M, Hassan C, rizkala T, Radadiya D, Nathani P, et al. Lack of Effectiveness of Computer Aided Detection for Colorectal Neoplasia: a Systematic Review and Meta-analysis of Non-Randomized Studies. 2024; **56**: S208.

573. Pecere S, Antonelli G, Dinis-Ribeiro M, Mori Y, Hassan C, Fuccio L, et al. Endoscopists performance in optical diagnosis of colorectal polyps in artificial intelligence studies. *United European gastroenterology journal* 2022; **10**: 817-26.

574. Pfeifer L, Neufert C, Leppkes M, Waldner MJ, Hafner M, Beyer A, et al. Computer-aided detection of colorectal polyps using a newly generated deep convolutional neural network: from development to first clinical experience. *European journal of gastroenterology & hepatology* 2021; **33**: e662-e9.

575. Prijic R, Grubelic Ravic K, Jelakovic M, Domislovic V, Brinar M, Kalauz M, et al. ARTIFICIAL INTELLIGENCE: A NEW TOOL IN ENDOSCOPIC SURVEILLANCE IN INFLAMMATORY BOWEL DISEASE PATIENTS. *United European Gastroenterology Journal* 2022; **10**: 329.

576. Putera M, Kwan C, Lin CL. REAL-WORLD STUDY OF SEQUENTIAL IMPLEMENTATION OF PHYSICIAN ADR REPORTING AND CAD-E SYSTEM IN IMPROVING PRAGMATIC ADENOMA DETECTION RATE (ADR) IN ELECTIVE COLONOSCOPY IN A TERTIARY HOSPITAL IN SINGAPORE. *Gastrointestinal Endoscopy* 2024; **99**: AB38-AB9.

577. Quan SY, Friedland S, Pirsiavash H, Kompella R, Sachdev V. Artificial intelligence based computer aided detection system reliably detects polyps earlier than physicians during colonoscopy. *American Journal of Gastroenterology* 2019; **114**: S159-S60.

578. Quan SY, Wei M, Lee J, Mohi-Ud-Din R, Mostaghim R, Sachdev RM, et al. Increased Polyp Detection In A Western Population Using A Real-Time Artificial Intelligence-Based System During Colonoscopy: A Pilot Study. *Gastroenterology* 2021; **160**: S-137.

579. Quan SY, Wei MT, Lee J, Mohi-Ud-Din R, Mostaghim R, Sachdev R, et al. Clinical evaluation of a real-time artificial intelligence-based polyp detection system: a US multi-center pilot study. *Scientific reports* 2022; **12**: 6598.

580. Qaqish F, Tawfik M, Abureesh M, Yetiskul E, Aslam T, Sleiman J, et al. The Impact of Artificial Intelligence-Assisted Colonoscopy on Key Colonoscopy Quality Indicators in the Underserved Population. *American Journal of Gastroenterology* 2024; **119**: S241.

581. Radadiya D, Patel HK, Srinivasan S, Devani K, Nathani P, Desai M, et al. ARE ALL COMPUTER-AIDED DETECTION SYSTEMS (CADE) CREATED EQUAL? - COMPARING ADENOMA DETECTION RATE OF DIFFERENT CADE SYSTEMS: NETWORK META-ANALYSIS OF RANDOMIZED CONTROLLED TRIALS. *Gastroenterology* 2023; **164**: S-1175.

582. Rath T, Pfeifer L, Seibt H, Eggert C, Huber H, Neufert C, et al. Computer-aided detection of colorectal polyps using a newly generated deep convolutional neural network. *United European Gastroenterology Journal* 2020; **8**: 772.

583. Renelus BD, Panchal T, Cole CA, Beazer JK, Joseph A, Tobun T, et al. AI ASSISTED COLONOSCOPY INCREASES ADENOMA DETECTION IN AVERAGE RISK SCREENING POPULATION. *Gastroenterology* 2023; **164**: S-316.

584. Renelus B, Cole C, Onyekaba J, Tobun T, Ortega D, Agbim C, et al. Computer-Aided Detection Device Increase Adenoma Detection in Anemic Patients Undergoing Diagnostic Colonoscopy. *American Journal of Gastroenterology* 2023; **118**: S248 EP - S9.

585. Repici A, Badalamenti M, Maselli R, Radaelli F, Rondonotti E, Pellegatta G, et al. 876 REAL-TIME COMPUTER AIDED DIAGNOSIS FOR DETECTION OF COLORECTAL NEOPLASIA AT COLONOSCOPY. *Gastrointestinal Endoscopy* 2020; **91**: AB71.

586. Repici A, Spadaccini M, Antonelli G, Maselli R, Galtieri PA, Pellegatta G, et al. Efficacy of real-time computer-aided detection of colorectal neoplasia in a non-expertsetting: A randomized controlled trial. *Endoscopy* 2021; **53**: S6.

587. Rex DK, Bhavsar-Burke I, Buckles D, Burton J, Cartee A, Comar K, et al. Artificial Intelligence for Real-Time Prediction of the Histology of Colorectal Polyps by General Endoscopists. *Annals of internal medicine* 2024; **177**: 911-8.

588. Rex DK, Mori Y, Sharma P, Lahr RE, Vemulapalli KC, Hassan C. Strengths and Weaknesses of an Artificial Intelligence Polyp Detection Program as Assessed by a High-Detecting Endoscopist. *Gastroenterology* 2022; **163**: 354-8.e1.

589. Rex DK, Guardiola JJ, von Renteln D, Mori Y, Sharma P, Hassan C. Detection of large flat colorectal lesions by artificial intelligence: a persistent weakness and blind spot. *Gut* 2025.

590. Richter R, Bruns J, Obst W, Keitel-Anselmino V, Weigt J. INFLUENCE OF ARTIFICIAL INTELLIGENCE ON THE ADENOMA DETECTION RATE THROUGHOUT THE DAY. *United European Gastroenterology Journal* 2022; **10**: 382.

591. Richter R, Bruns J, Obst W, Keitel-Anselmino V, Weigt J. Influence of Artificial Intelligence on the Adenoma Detection Rate throughout the Day. *Digestive diseases (Basel, Switzerland)* 2023; **41**: 615-9.

592. Rizkala T, Hassan C, Mori Y, Spadaccini M, Antonelli G, Dekker E, et al. Accuracy of Computer-aided Diagnosis in Colonoscopy Varies according to Polyp Location. A Systematic Review and Meta-analysis. *Clinical gastroenterology and hepatology : the official clinical practice journal of the American Gastroenterological Association* 2024.

593. Rizkala T, Menini M, Massimi D, Repici A. Role of Artificial Intelligence for Colon Polyp Detection and Diagnosis and Colon Cancer. *Gastrointestinal endoscopy clinics of North America* 2025; **35**: 389-400.

594. Robles-Medranda C, Cifuentes-Gordillo C, Arevalo-Mora M, Egas-Izquierdo M, Puga-Tejada M, Cunto D, et al. REAL-TIME COMPUTER-AIDED POLYP AND ADENOMA DETECTION DURING SCREENING COLONOSCOPY IN EXPERT AND NON-EXPERT ENDOSCOPISTS: A SINGLE CENTER STUDY. *Gastrointestinal Endoscopy* 2023; **97**: AB746.

595. Robles-Medranda C, Cifuentes-Gordillo C, Arevalo-Mora M, Mendez JC, Puga-Tejada M, Del Valle R, et al. REAL-TIME COMPUTER-AIDED POLYP/ ADENOMA DETECTION DURING SCREENING COLONOSCOPY: A SINGLE-CENTER DIAGNOSTIC TRIAL. *Digestive Endoscopy* 2022; **34**: 202.

596. Robles-Medranda C, Cifuentes-Gordillo C, Arevalo-Mora M, Puga-Tejada M, Egas-Izquierdo M, Baquerizo-Burgos J, et al. REAL-TIME COMPUTER-AIDED POLYP/ADENOMA DETECTION DURING SCREENING COLONOSCOPY: A SINGLE-CENTER DIAGNOSTIC TRIAL. *United European Gastroenterology Journal* 2022; **10**: 1027.

597. Roccato MK, Dao T, Berry R, Karnes W, Rael E. Artificial intelligence-aided colonoscopy: A retrospective analysis of effect on procedure time. *American Journal of Gastroenterology* 2019; **114**: S189-S90.

598. Rocchetto S, Segatta F, Piagnani A, Paggi S, Bina N, Di Paolo D, et al. Combining a Computer Aided Detection system (CADe) and G-EYE balloon for adenoma detection in a FIT-based organized colorectal cancer screening program: preliminary results of a randomized controlled trial. 2024; **56**: S456.

599. Rodriguez-Diaz E, Jepeal L, Baffy G, Lo WK, Bigio IJ, Singh SK. ARTIFICIAL INTELLIGENCE AUGMENTS REAL-TIME HISTOLOGY OF COLON POLYPS USING COMBINED-MODALITY NARROW BAND IMAGE CLASSIFICATION AND ELASTIC-SCATTERING SPECTROSCOPY. *Gastroenterology* 2019; **156**: S-933.

600. Rodriguez-Diaz E, Ravikumar V, Lo WK, Mashimo H, Repaka A, Xu H, et al. REAL TIME CLINICAL VALIDATION OF COMPUTER AIDED DIAGNOSIS WITH AUGMENTED REALITY FOR HISTOLOGY ASSESSMENT OF COLORECTAL POLYPS. *Gastrointestinal Endoscopy* 2023; **97**: AB745-AB6.

601. Rodriguez-Diaz E, Ravikumar VA, Lo WK, Mashimo H, Shahab O, Shah T, et al. EVALUATION OF REAL-TIME COLORECTAL POLYP HISTOLOGY ASSESSMENT USING COMPUTER AIDED DIAGNOSIS WITH AUGMENTED REALITY VISUALIZATION: PRELIMINARY RESULTS FROM PILOT STUDY. *Gastrointestinal Endoscopy* 2022; **95**: AB264-AB5.

602. Ronborg SN, Ujjal S, Ploug M, Kroijer R. Can artificial intelligence improve the quality of colonoscopy investigations? Evaluation of the GI genius endoscopy module in daily clinical practice. *Colorectal Disease* 2022; **24**: 227-8.

603. Ronborg SN, Ujjal S, Kroijer R, Ploug M. Assessing the potential of artificial intelligence to enhance colonoscopy adenoma detection in clinical practice: a prospective observational trial. *Clinical endoscopy* 2024.

604. Rondonotti E, Hassan C, Tamanini G, Antonelli G, Andrisani G, Leonetti G, et al. COMPUTER-AIDED OPTICAL DIAGNOSIS OF DIMINUTIVE RECTOSIGMOID POLYPS IN CLINICAL PRACTICE: A MULTICENTER PROSPECTIVE STUDY. *Digestive and Liver Disease* 2021; **53**: S92.

605. Sabbagh H, Tariq T, Wilson TJ, Kanaan Z, Lilley K, Rifkin S, et al. Ceiling Effect of CADe in Improving Detection Rates in FIT Positive Cases Among Gastroenterologists With High Baseline ADR. *American Journal of Gastroenterology* 2024; **119**: S309.

606. Sabran MZ, Sutanto R, Rusbiyanto MC, Sukendro GM, Huang I, Kurniawan A. Artificial intelligence-assisted colonoscopy detection rate on colorectal neoplasia patient: a systematic review of RCTs. *Annals of Oncology* 2024; **35**: S1331 EP - S2.

607. Saleepol A, Aniwan S, Tiankanon K, Kongtub N, Kulpatcharapong S, Vateekul P, et al. IMPACT OF A REAL-TIME COMPUTER-AIDED POLYP CHARACTERIZATION IN SCREENING COLONOSCOPY PERFORMED BY TRAINEES VERSUS EXPERIENCED ENDOSCOPISTS: A RANDOMIZED CONTROLLED TRIAL. *Gastrointestinal Endoscopy* 2024; **99**: AB22-AB3.

608. Salvi D, Pecere S, Cesaro P, Ciuffini C, Milluzzo SM, Quadarella A, et al. BENEFITS FROM A COMPUTER-AIDED DETECTION DEVICE IN COLONOSCOPY (ACCENDO-COLO) - AN INTERIM ANALYSIS OF AN ITALIAN MULTICENTER RANDOMIZED CLINICAL TRIAL. *Digestive and Liver Disease* 2023; **55**: S102-S3.

609. Samuel PI, Samuel P, Giday S. Impact of AI-Assisted Colonoscopy on Sessile Serrated Adenoma Detection in a Community GI Practice Setting. *American Journal of Gastroenterology* 2023; **118**: S274.

610. Sanchez-Peralta LF, Bote-Curiel L, Picon A, Sanchez-Margallo FM, Pagador JB. Deep learning to find colorectal polyps in colonoscopy: A systematic literature review. *Artificial intelligence in medicine* 2020; **108**: 101923.

611. Satiya J, Dammeyer K, Ahmad O, Stoyanov D, Lovat L, Popov V. Is Artificial Intelligence for Colonoscopy Ready for Prime-Time: A Systematic Review and Meta-Analysis of Randomized Controlled Trials. *American Journal of Gastroenterology* 2020; **115**: S165.

612. Sato K, Kuramochi M, Yamaguchi A, Hosoda Y, Tsuchiya A, Yamaguchi N, et al. A MULTICENTER SINGLE-ARM PROSPECTIVE STUDY TO ASSESS THE PERFORMANCE OF AN ARTIFICIAL INTELLIGENCE TO SUPPORT CHARACTERIAZTION OF COLORECTAL POLYPS. *Gastrointestinal Endoscopy* 2024; **99**: AB492-AB3.

613. Scalvini D, Agazzi S, Maimaris S, Rovedatti L, Brinch D, Cappellini A, et al. Strategies to Enhance the Adenoma Detection Rate (ADR) and the Serrated Polyp Detection Rate (SPDR) in Colonoscopy: A Comprehensive Review. *Gastroenterology Insights* 2025; **16**: 9.

614. Schacher F, da Rocha C, Borba S, Wolff F, Grillo L, Pinto R, et al. ARTIFICIAL INTELLIGENCE IN COLONOSCOPY: A REAL-WORLD EVALUATION. *Gastrointestinal Endoscopy* 2024; **99**: AB39-AB40.

615. Schauer C, Chieng M, Wang M, Neave M, Watson S, Van Rijnsoever M, et al. Artificial intelligence improves adenoma detection rate during colonoscopy. *The New Zealand medical journal* 2022; **135**: 22-30.

616. Schauer C, Chieng M, Wang M, Neave M, Watson S, Jafer A. Artificial intelligence for polyp detection during colonoscopy: A win for humans? *Gastroenterology and Hepatology from Bed to Bench* 2021; **14**: S128.

617. Schmidt K, Sood S, Dilmaghani S, Goyal M, Barry B, Zhu X, et al. PATIENT PERSPECTIVES AND ACCEPTABILITY OF ARTIFICIAL INTELLIGENCE USED DURING SCREENING COLONOSCOPY. *Gastroenterology* 2024; **166**: S-1493.

618. Schrader C, Wallstabe I, Schiefke I. Artificial intelligence in screening colonoscopy. *Coloproctology* 2022; **44**: 110-5.

619. Seager A, Sharp L, Hampton JS, Neilson LJ, Lee TJW, Brand A, et al. Trial protocol for COLO-DETECT: A randomized controlled trial of lesion detection comparing colonoscopy assisted by the GI Genius TM artificial intelligence endoscopy module with standard

colonoscopy. *Colorectal disease : the official journal of the Association of Coloproctology of Great Britain and Ireland* 2022; **24**: 1227-37.

620. Seager A, Sharp L, Neilson LJ, Brand A, Hampton J, Lee TJW, et al. THE EFFECT OF ENDOCUFF VISION ON POLYP DETECTION DURING COLONOSCOPY WITH THE GI GENIUS COMPUTER-AIDED DETECTION DEVICE. *Gut* 2024; **73**: A48 EP - A9.

621. Seager A, Sharp L, Neilson LJ, Brand A, Hampton J, Lee TJW, et al. BMJ GUT BEST LABORATORY SCIENCE ABSTRACT: COLO-DETECT: A RANDOMISED CONTROLLED TRIAL OF THE GI GENIUS AI DEVICE FOR POLYP DETECTION DURING ROUTINE COLONOSCOPY. *Gut* 2024; **73**: A11.

622. Sekiguchi M, Shinmura K, Sumiyama K, Matsuda T, Han KS, Kim HS, et al. Protocol for a multicenter randomized controlled trial to assess the usefulness of computer-aided detection systems for colonoscopy in colorectal cancer screening in the Asia-Pacific region (project CAD/NCCH2217). *Japanese journal of clinical oncology* 2025.

623. Sekiguchi M, Igarashi A, Toyoshima N, Takamaru H, Yamada M, Esaki M, et al. Cost-effectiveness analysis of computer-aided detection systems for colonoscopy in Japan. *Digestive endoscopy : official journal of the Japan Gastroenterological Endoscopy Society* 2023; **35**: 891-9.

624. Shah S, Park N, El Hage Chehade N, Monachese M, Ji SS, Nguyen PH, et al. ARTIFICIAL INTELLIGENCE AND COMPUTER AIDED DETECTION (CADE) SYSTEMS IMPROVE ADENOMA MISS RATES, ADENOMA DETECTION RATES AND POLYP DETECTION RATES: A SYSTEMATIC REVIEW AND META-ANALYSIS. *Gastrointestinal Endoscopy* 2022; **95**: AB231-AB2.

625. Shah S, Park N, Chehade NEH, Chahine A, Monachese M, Tiritilli A, et al. Effect of computer-aided colonoscopy on adenoma miss rates and polyp detection: A systematic review and meta-analysis. *Journal of gastroenterology and hepatology* 2023; **38**: 162-76.

626. Shah YR, Dahiya DS, Sebastian SA, Chandan S, Ramai D, Al Ta'ani O, et al. ASSESSING THE ECONOMIC IMPACT AND CLINICAL BENEFITS OF ARTIFICAL INTELLIGENCE (AI)-ASSISTED COLONOSCOPY USING SIMULATION MODELS: IS AI WORTH THE HYPE? - A SYSTEMATIC REVIEW AND META-ANALYSIS. *Gastrointestinal Endoscopy* 2025; **101**: S39 EP - S40.

627. Shao PP, Kuo J, Shao CR, Leung FW. Comparing Sessile Serrated Adenoma/Polyp Detection Rate Between Water Exchange and Computer-Aided Detection Colonoscopy Using Pooled Data From Randomized Controlled Trials. *American Journal of Gastroenterology* 2023; **118**: S286 EP - S7.

628. Shao PP, Kuo J, Shao CR, Leung FW. Comparing Advanced Adenoma Detection Rate Between Water Exchange and Computer-Aided Colonoscopy Using Pooled Data From Randomized Controlled Trials. *American Journal of Gastroenterology* 2023; **118**: S187 EP - S8.

629. Shao PP, Kuo J, Shao CR, Leung FW. Comparing Adenoma Detection Rate Between Computer-Aided Detection and Water Exchange Colonoscopy Using Pooled RCTs Data - An Interim Report. *American Journal of Gastroenterology* 2023; **118**: S298 EP - S300.

630. Shao P, Kuo J, Romero T, Leung F. WATER EXCHANGE AND COMPUTER-AIDED DETECTION IMPROVED ADENOMA DETECTION RATE - A META-ANALYSIS OF POOLED DATA FROM RANDOMIZED CONTROLLED TRIALS. *Gastrointestinal Endoscopy* 2024; **99**: AB626.

631. Shao P, Kuo J, Romero T, Leung F. WATER EXCHANGE IS SUPERIOR TO COMPUTER-AIDED DETECTION IN DETECTING SESSILE SERRATED ADENOMA/POLYP. *Gastrointestinal Endoscopy* 2024; **99**: AB626-AB7.

632. Shao L, Yan X, Liu C, Guo C, Cai B. Effects of ai-assisted colonoscopy on adenoma miss rate/adenoma detection rate: A protocol for systematic review and meta-analysis. *Medicine* 2022; **101**: e31945.

633. Sharma N, Chang P, Wang SJ, Nguyen D, Ong J, Amini M, et al. 393 ARTIFICIAL INTELLIGENCE ASSISTED COLONOSCOPY IMPROVES PDR AND ADR IN RIGHT COLON IN PATIENTS WITH IBD: A COHORT STUDY IN A HIGH-VOLUME CENTER. *Gastroenterology* 2024; **166**: S-89.

634. Shaukat A, Phillips SA, Chavali N, Colucci D, Erisson L, Ng J, et al. ID: 3526633 THE EFFECT OF CHANGING THE SPECIFICITY OF AN ARTIFICIAL INTELLIGENCE-AIDED POLYP DETECTION DEVICE AND ITS IMPACT ON CLINICAL PERFORMANCE. *Gastrointestinal Endoscopy* 2021; **93**: AB203.

635. Shaukat A, Lichtenstein DR, Somers SC, Chung DC, Perdue DG, Gopal M, et al. Computer-Aided Detection Improves Adenomas per Colonoscopy for Screening and Surveillance Colonoscopy: A Randomized Trial. *Gastroenterology* 2022; **163**: 732-41.

636. Shen P, Li WZ, Li JX, Pei ZC, Luo YX, Mu JB, et al. Real-time use of a computer-aided system for polyp detection during colonoscopy, an ambispective study. *Journal of digestive diseases* 2021; **22**: 256-62.

637. Shinozaki S, Watanabe J, Kanno T, Yuan Y, Yano T, Yamamoto H. Computer-aided diagnosis for colorectal polyp in comparison with endoscopists: Systematic review and meta-analysis. *Digestive Endoscopy* 2025.

638. Siggens K, Htet H, Abdelrahim M, Subramaniam S, Longcroft-Wheaton G, Bhandari P. THE FIRST EVER REAL-TIME EVALUATION OF A NOVEL CADE-IBD ALGORITHM FOR DETECTION OF NEOPLASIA DURING SURVEILLANCE COLONOSCOPY IN COLITIS PATIENTS. *Gut* 2024; **73**: A18 EP - A9.

639. Siggens K, Htet HM, Naoto M, Namiki S, Longcroft-Wheaton G, Aslam SP, et al. A VALIDATION STUDY EVALUATING THE ADDED VALUE OF A NOVEL COMPUTER AIDED DETECTION SYSTEM FOR NEOPLASIA DETECTION IN INFLAMMATORY BOWEL DISEASE (CADE-IBD). *Gastrointestinal Endoscopy* 2025; **101**: S13.

640. Siggens K, Htet H, Abdelrahim M, Marugame A, Alkandari A, Repici A, et al. VALIDATION AND REAL-TIME PERFORMANCE OF A NOVEL CAD-X ALGORITHM FOR CHARACTERISATION AND SIZING OF COLORECTAL POLYPS. *Gastrointestinal Endoscopy* 2023; **97**: AB730.

641. Siggens K, Htet H, Iwadate Y, Namiki S, Suthan H, Abdelrahim M, et al. VALIDATION OF A NOVEL CADE ALGORITHM FOR DETECTION OF NEOPLASIA IN IBD: DATA FROM IMAGE BASED AND REAL-TIME EVALUATION. *Gut* 2023; **72**: A38-A9.

642. Siggens K, Htet H, Marugame A, Saiga H, Alkandari A, Abdelrahim M, et al. P178 A NOVEL CADX ALGORITHM FOR CHARACTERISATION AND SIZING OF COLORECTAL POLYPS MEETS PIVI 1 AND PIVI 2 THRESHOLD. *Gut* 2023; **72**: A147-A8.

643. Singh A, Atkinson N, Schauer C, Jafer A, Walmsley R, Vanrijnsoever M. Artificial Intelligence for Colon Polyp Detection: Get a Machine for Your Unit Now! *Saudi Journal of Gastroenterology* 2019; **25**: 6.

644. Sinonquel P, De Wulf D, Tate DJ, Wyffels D, Eelbode T, Callaerts B, et al. THE CLINICAL VALIDATION OF A COMPUTER-ADIDE POLYP DETECTION MODEL INTEGRATED AS A PLUG-AND-PLAY ENDOSCOPY DEVICE (ALTER-EGO TRIAL). *Gastrointestinal Endoscopy* 2025; **101**: S33.

645. Sinonquel P, Eelbode T, Pech O, De Wulf D, Dewint P, Neumann H, et al. CLINICAL IMPACT OF THE MISMATCH BETWEEN OPTICAL DIAGNOSIS AND HISTOLOGY: A POST- HOC SUB-ANALYSIS OF THE CAD-ARTIPOD TRIAL. *Gastrointestinal Endoscopy* 2025; **101**: S19.

646. Sinonquel P, Eelbode T, Hassan C, Neumann H, Demedts I, Roelandt P, et al. Artificial intelligence for colorectal polyp detection: A validation trial with real-time unblinding. *United European Gastroenterology Journal* 2020; **8**: 749.

647. Sinonquel P, Eelbode T, Pech O, De Wulf D, Dewint P, Neumann H, et al. CLINICAL VALIDATION OF A COMPUTER-AIDED DETECTION MODEL FOR COLORECTAL POLYP DETECTION (CAD-ARTIPOD) TRIAL USING A SECOND OBSERVER AND REAL-TIME UNBLINDING. *Gastrointestinal Endoscopy* 2023; **97**: AB712.

648. Sinonquel P, Eelbode T, Hassan C, Antonelli G, Filosofi F, Neumann H, et al. Real-time unblinding for validation of a new CADe tool for colorectal polyp detection. *Gut* 2021; **70**: 641-3.

649. Sinonquel P, Eelbode T, Pech O, De Wulf D, Dewint P, Neumann H, et al. Clinical consequences of computer-aided colorectal polyp detection. *Gut* 2024.

650. Sivananthan A, Nazarian S, Ayaru L, Patel K, Ashrafian H, Darzi A, et al. ID: 3525827 PERFORMANCE OF COMPUTER AIDED DETECTION SYSTEMS IN FLAT, SESSILE AND DIMINUITIVE ADENOMAS: A META-ANALYSIS. *Gastrointestinal Endoscopy* 2021; **93**: AB197.

651. Sivananthan A, Nazarian S, Ayaru L, Patel K, Ashrafian H, Darzi A, et al. Does computer-aided diagnostic endoscopy improve the detection of commonly missed polyps? A meta-analysis. *Clinical endoscopy* 2022; **55**: 355-64.

652. Soleymanjahi S, Elmansy L, Rajashekar N, Mahmoudi-Rouhani R, Shung D. PERFORMANCE OF ARTIFICIAL INTELLIGENCE ASSISTED COLONOSCOPY VS CONVENTIONAL COLONOSCOPY: A SYSTEMATIC REVIEW AND META-ANALYSIS. *Gastroenterology* 2023; **164**: S-1177.

653. Soleymanjahi S, Huebner J, Elmansy L, Rajashekar N, Paracha R, Shung D. DOES COMPUTER-AIDED DETECTION (CADE) OFFER ANY ADVANTAGE TO CONVENTIONAL COLONOSCOPY: A COMPREHENSIVE COMPARISON OF EFFICACY AND SAFETY MEASURES? *Gastroenterology* 2024; **166**: S-1499.

654. Soleymanjahi S, Huebner J, Elmansy L, Rajashekar N, Paracha R, Shung D. PERFORMANCE OF DIFFERENT COMPUTER-AIDED DETECTION (CADE) PLATFORMS COMPARED TO CONVENTIONAL COLONOSCOPY: A SYSTEMATIC REVIEW AND META-ANALYSIS. *Gastroenterology* 2024; **166**: S-1501.

655. Soleymanjahi S, Kolb JM, Chung S, Foroutan F, Sultan S, Shung D. PROVIDER TRUST TOWARDS ADOPTING REAL TIME ARTIFICIAL INTELLIGENCE IN COLONOSCOPY: A SYSTEMATIC REVIEW. *Gastroenterology* 2024; **166**: S-1491.

656. Soleymanjahi S, Huebner J, Elmansy L, Rajashekar N, Ludtke N, Paracha R, et al. Artificial Intelligence-Assisted Colonoscopy for Polyp Detection. *Annals of Internal Medicine* 2024; **177**: 1652 EP - 63.

657. Soleymanjahi S, Kolb J, Foroutan F, Sultan S, Shung D. Perceived Advantages and Disadvantages of Adopting Real Time Artificial Intelligence in Colonoscopy by Providers: A Systematic Review. *American Journal of Gastroenterology* 2024; **119**: S292 EP - S3.

658. Soo JM-P, Koh FH-X. Detection of sessile serrated adenoma using artificial intelligence-enhanced endoscopy: an Asian perspective. *ANZ journal of surgery* 2024; **94**: 362-5.

659. Soons E, Rath T, Hazewinkel Y, van Dop WA, Esposito D, Testoni PA, et al. Real-time colorectal polyp detection using a novel computer-aided detection system (CADe): a feasibility study. *International journal of colorectal disease* 2022; **37**: 2219-28.

660. Spadaccini M, Alfarone L, Da Rio L, Maselli R, Carrara S, Galtieri PA, et al. COMPARING NUMBER AND RELEVANCE OF FALSE ACTIVATIONS BETWEEN TWO ARTIFICIAL INTELLIGENCE CADE SYSTEMS: THE NOISE STUDY. *Gastrointestinal Endoscopy* 2022; **95**: AB255.

661. Spadaccini M, Hassan C, Alfarone L, Da Rio L, Maselli R, Carrara S, et al. COMPARING NUMBER AND RELEVANCE OF FALSE ACTIVATIONS BETWEEN TWO ARTIFICIAL INTELLIGENCE CADE SYSTEMS: THE NOISE STUDY. *Digestive and Liver Disease* 2022; **54**: S81-S2.

662. Spadaccini M, Hassan C, De Marco A, Mori Y, Facciorusso A, Gkolfakis P, et al. REAL-TIME COMPUTER-AIDED DETECTION OF COLORECTAL NEOPLASIA DURING COLONOSCOPY: SYSTEMATIC REVIEW AND META-ANALYSIS. *Digestive and Liver Disease* 2023; **55**: S188-S9.

663. Spadaccini M, Hassan C, Mori Y, Facciorusso A, Maselli R, Khalaf K, et al. PERFORMANCE OF AI-AIDED COLONOSCOPY FOR THE DETECTION OF HIGH-RISK COLORECTAL CANCER PRECURSORS: A SYSTEMATIC REVIEW AND META-ANALYSIS. *Digestive and Liver Disease* 2022; **54**: S80.

664. Spadaccini M, Hassan C, Mori Y, Facciorusso A, Rizkala T, Massimi D, et al. VARIABILITY IN COMPUTER-AIDED DETECTION EFFECT ON ADENOMA DETECTION RATE IN RANDOMIZED CONTROLLED TRIALS: A META-REGRESSION ANALYSIS. *Digestive and Liver Disease* 2024; **56**: S229.

665. Spadaccini M, Hassan C, Mori Y, Halvorsen N, Gimeno-Garcia AZ, Nakashima H, et al. ARTIFICIAL INTELLIGENCE AND COLORECTAL NEOPLASIA DETECTION PERFORMANCES IN FIT+ PATIENTS: A META-ANALYSIS AND SYSTEMATIC REVIEW. *Digestive and Liver Disease* 2024; **56**: S143.

666. Spadaccini M, Hassan C, Selvaggio C, Antonelli G, Khalaf K, Rizkala T, et al. IN VIVO CONCORDANCE BETWEEN TWO ARTIFICIAL INTELLIGENCE SYSTEMS FOR LEAVING IN SITU COLORECTAL POLYPS. *Digestive and Liver Disease* 2023; **55**: S187.

667. Spadaccini M, Iannone A, Maselli R, Badalamenti M, Desai M, Chandrasekar VT, et al. ARTIFICIAL INTELLIGENCE VERSUS ADVANCED IMAGING FOR DETECTION OF COLORECTAL NEOPLASIA: A NETWORK METAANALYSIS. *Digestive and Liver Disease* 2021; **53**: S99-S100.

668. Spadaccini M, Hassan C, Alfarone L, Da Rio L, Maselli R, Carrara S, et al. Comparing the number and relevance of false activations between 2 artificial intelligence computer-aided detection systems: the NOISE study. *Gastrointestinal endoscopy* 2022; **95**: 975-81.e1.

669. Spadaccini M, Hassan C, Rondonotti E, Antonelli G, Andrisani G, Lollo G, et al. Combination of Mucosa-Exposure Device and Computer-Aided Detection for Adenoma Detection During Colonoscopy: A Randomized Trial. *Gastroenterology* 2023; **165**: 244-51.e3.

670. Spadaccini M, Iannone A, Maselli R, Badalamenti M, Desai M, Chandrasekar VT, et al. Computer-aided detection versus advanced imaging for detection of colorectal neoplasia: a systematic review and network meta-analysis. *The lancet Gastroenterology & hepatology* 2021; **6**: 793-802.

671. Spadaccini M, Hassan C, Mori Y, Halvorsen N, Gimeno-Garcia AZ, Nakashima H, et al. Artificial intelligence and colorectal neoplasia detection performances in patients with positive fecal immunochemical test: Meta-analysis and systematic review. *Digestive Endoscopy* 2025.

672. Spadaccini M, Hassan C, Mori Y, Massimi D, Correale L, Facciorusso A, et al. Variability in computer-aided detection effect on adenoma detection rate in randomized controlled trials: A meta-regression analysis. *Digestive and Liver Disease* 2025; **57**: 1141 EP - 8.

673. Su J-R, Li Z, Shao X-J, Ji C-R, Ji R, Zhou R-C, et al. Impact of a real-time automatic quality control system on colorectal polyp and adenoma detection: a prospective randomized controlled study (with videos). *Gastrointestinal endoscopy* 2020; **91**: 415-24.e4.

674. Sultan S, Shung DL, Kolb JM, Foroutan F, Hassan C, Kahi CJ, et al. AGA Living Clinical Practice Guideline on Computer-Aided Detection-Assisted Colonoscopy. *Gastroenterology* 2025; **168**: 691 EP - 700.

675. Syed T, Baratta A, Strapko A, Malik AK. Artificial Intelligence (CAD-E)-Assisted Colonoscopy Helps Increase Adenoma Detection Rate (ADR) in the Afternoon Session. *American Journal of Gastroenterology* 2023; **118**: S219 EP - S20.

676. Taghiakbari M, Rex DK, Pohl H, Hassan C, Djinbachian R, Huang F, et al. PRAGMATIC IMPLEMENTATION OF RESECT AND DISCARD AND DIAGNOSE AND LEAVE STRATEGIES USING AUTONOMOUS COMPUTER-ASSISTED OPTICAL POLYP DIAGNOSIS. *Gastrointestinal Endoscopy* 2025; **101**: S50.

677. Takasu A, Gotoda T, Dai Z, Yamada Y, Nakayama M, Miura Y, et al. IMPACT OF INTRODUCING ARTIFICIAL INTELLIGENCE ON COLONOSCOPY IN REAL-WORLD CLINICAL PRACTICE. *Gastrointestinal Endoscopy* 2025; **101**: S55 EP - S6.

678. Tanaka I, Ono S, Nishimura Y, Kubo M, Shimoda Y, Inoue M, et al. Evaluation of cad eyeTM based on artificial intelligence technology for detection and characterization of colorectal neoplasia in a clinical setting. *United European Gastroenterology Journal* 2021; **9**: 799-800.

679. Tang CP, Hsieh YH, Lin TL, Tseng CW, Leung F. Comparing polyp detection rate between water exchange and air insufflation by a computeraided detection algorithm: An analysis of withdrawal phase videos from a randomized controlled trial. *American Journal of Gastroenterology* 2021; **116**: S94.

680. Tang CP, Lin TL, Hsieh YH, Hsieh CH, Leung FW. ADENOMA DETECTION USING REAL-TIME COMPUTER-AIDED COLON POLYP DETECTION SYSTEM TO COMPARE WATER EXCHANGE AND AIR INSUFFLATION - A PILOT CONTROLLED STUDY. *Gastrointestinal Endoscopy* 2022; **95**: AB141-AB2.

681. Tang C-P, Lin T-L, Hsieh Y-H, Hsieh C-H, Tseng C-W, Leung FW. Polyp detection and false-positive rates by computer-aided analysis of withdrawal-phase videos of colonoscopy of the right-sided colon segment in a randomized controlled trial comparing water exchange and air insufflation. *Gastrointestinal endoscopy* 2022; **95**: 1198-206.e6.

682. Tang C-P, Shao PP, Hsieh Y-H, Leung FW. A review of water exchange and artificial intelligence in improving adenoma detection. *Tzu chi medical journal* 2021; **33**: 108-14.

683. Tang CP, Lin TL, Leung FW, Tseng CW. REAL-TIME COMPUTER-AIDED SYSTEM TO COMPARE RIGHT COLON ADENOMA DETECTION AND ADENOMA PER COLONOSCOPY RATES IN WATER EXCHANGE AND AIR INSUFFLATION - A RANDOMIZED CONTROLLED STUDY. *Gastrointestinal Endoscopy* 2025; **101**: S287 EP - S8.

684. Tariq R, Dilmaghani S, Advani R, Soroush A, Berzin T, Khanna S. Perception and Understanding of Artificial Intelligence Among Gastroenterology Fellows and Early Career Gastroenterologists: A Nationwide Cross-Sectional Survey Study. *Digestive Diseases and Sciences* 2025.

685. Thai Clinical Trials Registry (TCTR). Comparison of computer-aided diagnosis colonoscopy, Endocuff-Assisted Colonoscopy, combination of computer-aided diagnosis colonoscopy and Endocuff-Assisted Colonoscopy and High-definition Colonoscopy for Adenomas Detection in Colorectal Cancer Screening (TCTR20200929003), 2020. Available from: https://www.cochranelibrary.com/central/doi/10.1002/central/CN-02190086/full. Date accessed: Feb 25.

686. Thai Clinical Trials Registry (TCTR). Comparison between two computer-aided diagnosis colonoscopy systems (Deep GI system and CAD EYE system) and High-definition Colonoscopy for Adenomas Detection in Colorectal Cancer Screening: a randomized control Trial (TCTR20220826004), 2022. Available from: https://www.cochranelibrary.com/central/doi/10.1002/central/CN-02473959/full. Date accessed: Feb 25.

687. Thai Clinical Trials Registry (TCTR). Adenoma Miss rate in Artificial Intelligence-Based versus Conventional Colonoscopy, A Prospective Randomized Trial (TCTR20230504002), 2023. Available from: https://www.cochranelibrary.com/central/doi/10.1002/central/CN-02566635/full. Date accessed: Feb 25.

688. Thai Clinical Trials Registry (TCTR). Comparison between two computer-aided polyp detection colonoscopy systems and High-definition Colonoscopy for Adenomas Detection in Colorectal Cancer Screening: a multi-center randomized control Trial (TCTR20230706006), 2023. Available from: https://www.cochranelibrary.com/central/doi/10.1002/central/CN-02586609/full. Date accessed: Feb 25.

689. Thai Clinical Trials Registry (TCTR). Using Computer-Aided polyp detection system (CADe) to maintain the high quality in adenoma detection rate during community-based colorectal cancer screening in Thailand: a randomized trial (TCTR20240710001), 2024.

Available from: https://www.cochranelibrary.com/central/doi/10.1002/central/CN-02732746/full. Date accessed: Feb 25.

690. Tham S, Koh FH, Teo E-K, Lin C-L, Foo F-J, Tan Wj SSSHLMLNJLKFHCCADKJHTA, et al. Knowledge, perceptions and behaviours of endoscopists towards the use of artificial intelligence-aided colonoscopy. *Surgical endoscopy* 2023; **37**: 7395-400.

691. Thijssen A, Schreuder RM, Dehghani N, Schor M, De With PHN, Van Der Sommen F, et al. Improving the endoscopic recognition of early colorectal carcinoma using artificial intelligence: current evidence and future directions. *Endoscopy International Open* 2024; **12**: E1102 EP - E17.

692. Thiruvengadam NR, Cote GA, Gupta S, Rodrigues M, Schneider Y, Arain MA, et al. THE IMPACT OF COMPUTER-AIDED DETECTION ON PATIENTS UNDERGOING SCREENING COLONOSCOPY PERFORMED BY LOW- AND HIGH-DETECTOR ENDOSCOPISTS: A MICROSIMULATION ANALYSIS. *Gastroenterology* 2023; **164**: S-314.

693. Thiruvengadam NR, Schneider Y, Kochman ML, Saumoy M. COST-EFFECTIVENESS OF THE ADDITION OF REAL-TIME COMPUTER-AIDED DETECTION OF ADENOMAS TO SCREENING COLONOSCOPY IN COLORECTAL CANCER SCREENING OF AVERAGE-RISK PERSONS AT 45 YEARS OF AGE. *Gastrointestinal Endoscopy* 2022; **95**: AB178.

694. Thiruvengadam NR, Solaimani P, Shrestha M, Carson R, Reyes-Garcia B, Buller S, et al. EFFICACY OF REAL-TIME COMPUTER AIDED-DETECTED OF COLONIC NEOPLASIA IN AN UNDERSERVED POPULATION, A RANDOMIZED CONTROLLED TRIAL. *Gastroenterology* 2023; **164**: S-155.

695. Thiruvengadam NR, Cote GA, Gupta S, Rodrigues M, Schneider Y, Arain MA, et al. An Evaluation of Critical Factors for the Cost-Effectiveness of Real-Time Computer-Aided Detection: Sensitivity and Threshold Analyses Using a Microsimulation Model. *Gastroenterology* 2023; **164**: 906-20.

696. Thomas J, Ravichandran R, Nag A, Gupta L, Singh M, Panjiyar BK. Advancing Colorectal Cancer Screening: A Comprehensive Systematic Review of Artificial Intelligence (AI)-Assisted Versus Routine Colonoscopy. *Cureus* 2023; **15**: e45278.

697. Tiankanon K, Aniwan S, Mekritthikrai K, Kongtub N, Wisedopas N, Piyachaturawat P, et al. THE IMPROVEMENT ON ADENOMA DETECTION RATE AND OTHER SECONDARY INDICATORS OF THE TWO REAL-TIME ARTIFICIAL INTELLIGENCES IN HIGH ADENOMA DETECTORS: A RANDOMIZED MUTI-CENTER TRIAL. *Gastrointestinal Endoscopy* 2023; **97**: AB733.

698. Tolosa C, Arayakarnkul S, Inal E, White J, Al Ghamdi S, Ngamruengphong S. REDUCTION OF ADENOMA MISS RATE WITH ARTIFICIAL INTELLIGENCE (AI): A META-ANALYSIS OF RANDOMIZED TANDEM TRIALS OF AI-ASSISTED COLONOSCOPY. *Gastrointestinal Endoscopy* 2023; **97**: AB727.

699. Japan Primary Registries Network (JPRN) - University Hospital Medical Information Network (UMIN) Clinical Trials Registry. Prospective study for endocytoscopy-based computer-aided diagnosis system for small colorectal lesions (UMIN000013917), 2014. Available from: https://www.cochranelibrary.com/central/doi/10.1002/central/CN-01824364/full. Date accessed: Feb 25.

700. Japan Primary Registries Network (JPRN) - University Hospital Medical Information Network (UMIN) Clinical Trials Registry. Evaluation of the impact of CAD EYE on the quality of colonoscopy and the learning curve of gastroenterology fellows (UMIN000044031), 2021. Available from: https://www.cochranelibrary.com/central/doi/10.1002/central/CN-02411986/full. Date accessed: Feb 25.

701. Japan Primary Registries Network (JPRN) - University Hospital Medical Information Network (UMIN) Clinical Trials Registry. Artificial Intelligence in Colonoscopy for Cancer Prevention -a Randomized Health Service Implementation Trial (UMIN000044748), 2021. Available from: https://www.cochranelibrary.com/central/doi/10.1002/central/CN-02330310/full. Date accessed: Feb 25.

702. Japan Primary Registries Network (JPRN) - University Hospital Medical Information Network (UMIN) Clinical Trials Registry. A randomized control trial of adenoma detection rate in artificial intelligence-assisted colonoscopy using linked color imaging (UMIN000046361), 2021. Available from: https://www.cochranelibrary.com/central/doi/10.1002/central/CN-02412245/full. Date accessed: Feb 25.

703. Japan Primary Registries Network (JPRN) - University Hospital Medical Information Network (UMIN) Clinical Trials Registry. Examination of detection ability of colorectal polyps with AI colonoscopy (UMIN000046502), 2022. Available from: https://www.cochranelibrary.com/central/doi/10.1002/central/CN-02412286/full. Date accessed: Feb 25.

704. Japan Primary Registries Network (JPRN) - University Hospital Medical Information Network (UMIN) Clinical Trials Registry. Veridation research of colonoscopy using artificial intelligence (UMIN000047666), 2022. Available from: https://www.cochranelibrary.com/central/doi/10.1002/central/CN-02430063/full. Date accessed: Feb 25.

705. Vadhwana B, Tarazi M, Patel V. The Role of Artificial Intelligence in Prospective Real-Time Histological Prediction of Colorectal Lesions during Colonoscopy: A Systematic Review and Meta-Analysis. *Diagnostics (Basel, Switzerland)* 2023; **13**.

706. van der Zander QE, Schreuder RM, Thijssen A, Kusters KC, Dehghani N, Scheeve T, et al. REAL-TIME CLASSIFICATION OF COLORECTAL POLYPS USING ARTIFICIAL INTELLIGENCE - A PROSPECTIVE PILOT STUDY COMPARING TWO COMPUTER-AIDED DIAGNOSIS SYSTEMS AND ONE EXPERT ENDOSCOPIST. *Gastrointestinal Endoscopy* 2022; **95**: AB250-AB1.

707. Van Langendonc S, Corens P, Stragier E, Vanderstraeten E, Walgraeve D, Bouhadan S, et al. Computer aided detection (CADE) in colonoscopy: an end-user experience using two systems. *Endoscopy* 2021; **53**: S254.

708. Vinsard DG, Herman T, Smith LA, Gravely A, Reinink A, Lou S, et al. IMPACT OF ARTIFICIAL INTELLIGENCE ON RIGHT COLONIC ADENOMA MISS RATE: A PRAGMATIC QUASI-RANDOMIZED TRIAL. *Gastrointestinal Endoscopy* 2025; **101**: S20 EP - S1.

709. Wadhwa RP, Ravindranath A, Sengupta K, Shahane S, Naik NR. Diagnostic Accuracy of Artificial Intelligence in Classification of Colonic Polyps - A Real World Prospective Study. *American Journal of Gastroenterology* 2023; **118**: S535.

710. Wallace MB, Sharma P, Bhandari P, East JE, Antonelli G, Lorenzetti R, et al. IMPACT OF ARTIFICIAL INTELLIGENCE ON MISS RATE OF COLORECTAL NEOPLASIA: A RANDOMIZED TANDEM CLINICAL TRIAL. *Gastrointestinal Endoscopy* 2022; **95**: AB180.

711. Wang A, Mo J, Zhong C, Wu S, Wei S, Tu B, et al. Artificial intelligence-assisted detection and classification of colorectal polyps under colonoscopy: A systematic review and meta-analysis. *Ann Transl Med* 2021; **9**: A28.

712. Wang L, Feng H, Chen W, Luan F. Artificial Intelligence - based Colorectal Polyp Diagnostic System Can Increase the Detection Rate of Polyps: A Prospective Randomized Controlled Study. *Chinese Journal of Gastroenterology* 2022; **27**: 163-7.

713. Wang P, Bharadwaj S, Berzin TM, Becq A, Li L, Liu P, et al. Assistance of a real-time automatic colon polyp detection system increases polyp and adenoma detection during colonoscopy: A prospective randomized controlled study. *United European Gastroenterology Journal* 2018; **6**: A2-A3.

714. Wang P, Li L, Liu P, Song Y, Zhang D, Li Y, et al. Automatic polyp detection during colonoscopy increases adenoma detection: An interim analysis of a prospective randomized control study. *Gastrointestinal Endoscopy* 2018; **87**: AB490-AB1.

715. Wang P, Li L, Zhang B, Cheng Y, Meng F, Xiao W, et al. A retrospective study of computer-aided detection system for detection improvement of adenomas. *Chinese Journal of Digestive Endoscopy* 2024; **41**: 443-8.

716. Wang P, Zhou G, Glissen Brown JR, Berzin TM, Liu X, Li L, et al. Colonoscopy with embedded deep learning computer-aided detection system improves adenoma detection without increasing physician fatigue: A prospective randomized study. *United European Gastroenterology Journal* 2019; **7**: 1418-9.

717. Wang P, Zhou G, Liu P, Liu X, Li L, Xiao X. 859 COMPUTER-AIDED-DETECTION EMBEDDED COLONOSCOPY VERSUS ROUTINE COLONOSCOPY: A PROSPECTIVE, RANDOMIZED TANDEM TRIAL. *Gastroenterology* 2020; **158**: S-176.

718. Wang A, Mo J, Zhong C, Wu S, Wei S, Tu B, et al. Artificial intelligence-assisted detection and classification of colorectal polyps under colonoscopy: a systematic review and meta-analysis. *Ann Transl Med* 2021; **9**: 1662.

719. Wang Y, He C. ENDOANGEL improves detection of missed colorectal adenomas in second colonoscopy: A retrospective study. *Medicine* 2024; **103**: e38938.

720. Wang SJ, Wang D, Chang P, Bakr O, Sharma N, Nguyen D, et al. AI Assisted Colonoscopy Improves Polyp Detection in Obese Patients. *American Journal of Gastroenterology* 2024; **119**: S1499.

721. Wang SJ, Wang D, Sharma N, Chang P, Nguyen D, Ong J, et al. Artificial Intelligence-Assisted Colonoscopy Improves PDR in Patients With IBD: A Cohort Study in a High Volume Center. *American Journal of Gastroenterology* 2024; **119**: S929.

722. Warman R, Singh T, Lezama J, Gallagher JE, Borkowski A, Vidyarthi G. Non-Neoplastic Polyp Detection Using AI, GI Genius, With Experienced Endoscopist - Pilot Study. *American Journal of Gastroenterology* 2024; **119**: S189 EP - S90.

723. Wei M, Fay S, Yung D, Ladabaum U, Kopylov U. Artificial Intelligence-Assisted Colonoscopy in Real World Clinical Practice: A Systematic Review and Meta-Analysis. *American Journal of Gastroenterology* 2023; **118**: S264 EP - S5.

724. Wei M, Shankar U, Friedlander Y, Friedland S. EVALUATION OF ARTIFICIAL INTELLIGENCE ENABLED COMPUTER AIDED DETECTION ASSISTANCE IN DETECTING COLON POLYPS IN THE COMMUNITY (AI-SEE): A MULTICENTER RANDOMIZED CLINICAL TRIAL. *Gastroenterology* 2023; **164**: S-112.

725. Wei MT, Fay S, Yung D, Ladabaum U, Kopylov U. Artificial Intelligence-Assisted Colonoscopy in Real-World Clinical Practice: A Systematic Review and Meta-Analysis. *Clinical and translational gastroenterology* 2024; **15**: e00671.

726. Wenderott K, Krups J, Zaruchas F, Weigl M. Effects of artificial intelligence implementation on efficiency in medical imaging-a systematic literature review and meta-analysis. *npj Digital Medicine* 2024; **7**: 265.

727. Wong AYT, Wong KF. The study on artificial intelligence (AI) colonoscopy in affecting the rate of polyp detection in colonoscopy - A single center retrospective study. *Surgical Practice* 2021; **25**: 6.

728. Wong YT, Tai TF, Wong KF, Leung SK, Lam SM, Wong SY, et al. The study on artificial intelligence (AI) colonoscopy in affecting the rate of polyp detection in colonoscopy: A single centre retrospective study. *Surgical Practice* 2022; **26**: 115-9.

729. Wong YT, Wong KF. The study on artificial intelligence (AI) colonoscopy in affecting the rate of polyp detection in colonoscopy - a single center retrospective study. *Surgical Endoscopy* 2022; **36**: S340-S1.

730. Wong YT, Wong KF. The study on artificial intelligence (ai) colonoscopy in affecting the rate of polyp detection in colonoscopy - a single-center retrospective study. *Gut* 2021; **70**: A101-A2.

731. Wu Y, Wang J, Ma Y, Zeng R, Sha W, Chen H, et al. Efficacy of Water Exchange vs Computer-Aided Detection: A Bayesian Network Meta-Analysis of Randomized Controlled Trials. *American Journal of Gastroenterology* 2023; **118**: S537.

732. Xu A, Catania VV, Nguyen TBP, Kastuar S, Ho L, Sparkman J, et al. Implementation of Artificial Intelligence Device for Polyp Detection During Colonoscopy at an Academic County Hospital System. *American Journal of Gastroenterology* 2023; **118**: S220 EP - S1.

733. Xu Z, Li Y, Su P, Zhong Z, Zeng Z, Chen M, et al. Artificial intelligence system improves the quality of digestive endoscopy: A prospective pretest and post-test single-center clinical trial. *Digestive and Liver Disease* 2025.

734. Xu L, He X, Zhou J, Zhang J, Mao X, Ye G, et al. Artificial intelligence-assisted colonoscopy: A prospective, multicenter, randomized controlled trial of polyp detection. *Cancer medicine* 2021; **10**: 7184-93.

735. Xu Y, Ding W, Wang Y, Tan Y, Xi C, Ye N, et al. Comparison of diagnostic performance between convolutional neural networks and human endoscopists for diagnosis of colorectal polyp: A systematic review and meta-analysis. *PloS one* 2021; **16**: e0246892.

736. Yamada M, Saito Y, Imaoka H, Saiko M, Yamada S, Kondo H, et al. Development of a real-time endoscopic image diagnosis support system using deep learning technology in colonoscopy. *Scientific reports* 2019; **9**: 14465.

737. Yamada M, Saito Y, Yamada S, Kondo H, Hamamoto R. Detection of flat colorectal neoplasia by artificial intelligence: A systematic review. *Best practice & research Clinical gastroenterology* 2021; **52**: 101745.

738. Yamaguchi D, Miyahara K, Yukimoto T, Takamori A, Mizuta Y, Fujimura Y, et al. EVALUATION OF THE IMPACT OF AN AI-AIDED ENDOSCOPIC DIAGNOSIS SYSTEM ON IMPROVING ENDOSCOPY QUALITY AND INCREASING THE LEARNING CURVE FOR BEGINNING COLONOSCOPY TRAINEES: A PROSPECTIVE RANDOMIZED MULTI-CENTER STUDY. *Gastrointestinal Endoscopy* 2023; **97**: AB176.

739. Yang LS, Perry E, Shan L, Wilding H, Connell W, Thompson AJ, et al. Clinical application and diagnostic accuracy of artificial intelligence in colonoscopy for inflammatory bowel disease: systematic review. *Endoscopy international open* 2022; **10**: E1004-E13.

740. Yi Y, Guo Y, Lam T. REAL-TIME USE OF ARTIFICIAL INTELLIGENCE IN CHARACTERIZATION OF DIMINUTIVE POLYPS DURING COLONOSCOPY: A SYSTEMATIC REVIEW AND META-ANALYSIS. *Gastrointestinal Endoscopy* 2024; **99**: AB41-AB2.

741. Yu H, Wu L, Hu S. Improved adenoma detection with ENDOANGEL: A randomized controlled trial. *United European Gastroenterology Journal* 2019; **7**: 173-4.

742. Yu H, Zhang C, Yao L. ASSESSMENT OF THE ROLE OF FALSE-POSITIVE ALERTS IN COMPUTER-AIDED POLYP DETECTION FOR ASSISTANCE CAPABILITIES: A SECONDARY ANALYSIS OF RANDOMIZED CLINICAL TRIAL. *Gastroenterology* 2024; **166**: S-603.

743. Zha B, Cai A, Wang G. Diagnostic Accuracy of Artificial Intelligence in Endoscopy: Umbrella Review. *JMIR medical informatics* 2024; **12**: e56361.

744. Zhang L, Yao L, Yu H. An artificial intelligence-based quality improvement system significantly improved the efficacy of computer-aided detection system in colonoscopy: A 2*2 factorial analysis. *United European Gastroenterology Journal* 2021; **9**: 808-9.

745. Zhang C, Yao L, Jiang R, Wang J, Wu H, Li X, et al. Assessment of the role of false-positive alerts in computer-aided polyp detection for assistance capabilities. *Journal of gastroenterology and hepatology* 2024; **39**: 1623-35.

746. Zhang Y, Zhang X, Wu Q, Gu C, Wang Z. Artificial Intelligence-Aided Colonoscopy for Polyp Detection: A Systematic Review and Meta-Analysis of Randomized Clinical Trials. *Journal of laparoendoscopic & advanced surgical techniques Part A* 2021; **31**: 1143-9.

747. Zhang C, Tao X, Pan J, Huang L, Dong Z, Lin J, et al. The Effect of Computer-Aided Device on Adenoma Detection Rate in Different Implement Scenarios: A Real-World Study. *Journal of Gastroenterology and Hepatology (Australia)* 2025; **40**: 692 EP - 705.

748. Zhao Y, Wei J, Zhang S, Li Z, Zhao S, Bai Y. Artificial intelligence in colorectal sessile serrated lesion: recent progress. *Academic Journal of Naval Medical University* 2025; **46**: 24 EP - 31.

749. Zhao G, Li X, Liang X, Zhu H, Jia M, Wang B, et al. Influence of artificial intelligence on colonoscopy in different examination periods. *Gut* 2021; **70**: A138.

750. Zhao G, Wang X, Bai Y, Li H, Liang X, Li X, et al. CLINICAL STUDY OF ARTIFICIAL INTELLIGENCE ASSISTED COLONOSCOPY IN DETECTING COLORECTAL POLYPS. *Gut* 2022; **71**: A119.

751. Zhao S-B, Yang W, Wang S-L, Pan P, Wang R-D, Chang X, et al. Establishment and validation of a computer-assisted colonic polyp localization system based on deep learning. *World journal of gastroenterology* 2021; **27**: 5232-46.

752. Zhou G, Liu X, Berzin TM, Brown JRG, Li L, Zhou C, et al. A REAL-TIME AUTOMATIC DEEP LEARNING POLYP DETECTION SYSTEM INCREASES POLYP AND ADENOMA DETECTION DURING COLONOSCOPY: A PROSPECTIVE DOUBLE-BLIND RANDOMIZED STUDY. *Gastroenterology* 2019; **156**: S-1511.

753. Zippelius C, Schedel J, Brookman-Amissah D, Muehlenberg K, Schorr W, Salzberger A, et al. Prospective evaluation of a new artificial intelligence system for detection of colonpolyps. *Endoscopy* 2021; **53**: S51.

754. Zippelius C, Alqahtani SA, Schedel J, Brookman-Amissah D, Muehlenberg K, Federle C, et al. Diagnostic accuracy of a novel artificial intelligence system for adenoma detection in daily practice: a prospective nonrandomized comparative study. *Endoscopy* 2022; **54**: 465-72.

755. Li JW, Wu CCH, Lee JWJ, Liang R, Soon GST, Wang LM, et al. Real-World Validation of a Computer-Aided Diagnosis System for Prediction of Polyp Histology in Colonoscopy: A Prospective Multicenter Study. *The American journal of gastroenterology* 2023; **118**: 1353-64.

756. Picardo S, Menon S, So K, Venugopal K, Cheng W, Ragunath K. PP-495 Evaluation of the artificial intelligencesystem CAD-EYE to characterize lesions ininflammatory bowel disease surveillance. *Journal of Gastroenterology and Hepatology*. 2023. p. 280. Asian Pacific Digestive Week; Bangkok, Thailand.

757. Zavyalov DV, Kashin SV, Guseinova SRAOZDV, Ka Ohoo---A-. CAD EYE for real-time detection and differentiation of colorectal lesions. *Russian Journal of Evidence-Based Gastroenterology* 2024; **13**: 50-4.

758. Odin Vision. Clinical Investigation Report - CADDIE Trial_CONFIDENTIAL. 2023.

759. Odin Medical Ltd. Clinical Investigation Report - EAGLE Trial_CONFIDENTIAL. 2024.

760. EndoPerv LLC. Preliminary Results EMIS NIH study (CONFIDENTIAL). 2025.

761. Tavanapong W, Pratt J, Oh J, Khaleel M, Wong JS, de Groen P.C. Ao - Pratt J, et al. Development and deployment of Computer-aided Real-Time feedback for improving quality of colonoscopy in a Multi-Center clinical trial. *Biomedical Signal Processing and Control* 2023; **83**: 104609.

762. Baumer S, Streicher K, Alqahtani SA, Brookman-Amissah D, Brunner M, Federle C, et al. Accuracy of polyp characterization by artificial intelligence and endoscopists: a prospective, non-randomized study in a tertiary endoscopy center. *Endoscopy international open* 2023; **11**: E818-E28.

763. Koh GE, Ng B, Lagstrom RMB, Foo F-J, Chin S-E, Wan F-T, et al. Real-World Assessment of the Efficacy of Computer-Assisted Diagnosis in Colonoscopy: A Single Institution Cohort Study in Singapore. *Mayo Clinic proceedings Digital health* 2024; **2**: 647-55.

764. Ladabaum U, Mannalithara A, Weng Y, Shaw B, Olsen E, Watkins K, et al. BELIEFS AND ATTITUDES ABOUT ARTIFICIAL INTELLIGENCE (AI) AMONG COLONOSCOPIST PARTICIPANTS IN A PRAGMATIC IMPLEMENTATION TRIAL OF COMPUTER-AIDED DETECTION (CADE) OF POLYPS THAT DID NOT REPLICATE THE POSITIVE RESULTS OF RANDOMIZED TRIALS. *Gastrointestinal Endoscopy* 2023; **97**: AB763-AB4.

765. Clinicaltrials.gov. Nationwide Study of Artificial Intelligence in Adenoma Detection for Colonoscopy (NAIAD), 2024. Available from: https://www.clinicaltrials.gov/study/NCT05870332. Date accessed: Aug 2025.

766. Nehme F, Coronel E, Barringer DA, Romero LG, Shafi MA, Ross WA, et al. Performance and attitudes toward real-time computer-aided polyp detection during colonoscopy in a large tertiary referral center in the United States. *Gastrointestinal endoscopy* 2023; **98**: 100-9.e6.

767. Olabintan O, Iniesta R, Siwoku S, Eqbal A, Ayubi H, Naeem N, et al. UK ENDOSCOPISTS' PERSPECTIVES ON ARTIFICIAL INTELLIGENCE IN ENHANCING POLYP MANAGEMENT AND ENDOSCOPIC PRACTICE. *Gastrointestinal Endoscopy* 2025; **101**: S52 EP - S3.

768. Pinto C, Ortigao R, Chaves J, Ramos Silva D, Dinis-Ribeiro M, Lopes Brandao C. ACUITY OF ARTIFICIAL INTELLIGENCE ASSISTED COLONOSCOPY IN LYNCH SYNDROME PATIENTS. *United European Gastroenterology Journal* 2022; **10**: 1025.

769. Seager A, Dobson C, Sharp L, Rees C. USERS' OPINIONS & EXPERIENCES OF A COMPUTERAIDED DETECTION DEVICE FOR COLONOSCOPY AND POTENTIAL EFFECTS ON ADOPTION AND IMPLEMENTATION. *Gut* 2024; **73**: A189.

770. Anderson R, Materacki L, Zeino Z, Dharmasiri S. ARTIFICIAL INTELLIGENCE IN COLONOSCOPY: REAL WORLD EXPERIENCE FROM THE SOUTHWEST ENDOSCOPY GROUP. *Gut* 2024; **73**: A162.

771. Burton SJ, Shung D, Chung S, Aslanian H. Patient Perspective of Use of Artificial Intelligence During Colonoscopy. *Gastro hep advances* 2025; **4**: 100543.

772. Magahis PT, Pence CJ, Wan D. Impact of Artificial Intelligence on Gastroenterology Training and Education: A Survey of Fellows' Perspectives. *American Journal of Gastroenterology* 2023; **118**: S555 EP - S6.

773. Schmidt KA, Sood S, Dilmaghani S, Leggett C, Dierkhising R, Goyal M, et al. Understanding Patients' Current Acceptability of Artificial Intelligence During Colonoscopy for Polyp Detection: A Single-Center Study. *Techniques and Innovations in Gastrointestinal Endoscopy* 2025; **27**: 250905.

774. Higgins J, Thomas J, Chandler J, Cumpston M, Li T, Page M, et al. Cochrane Handbook for Systematic Reviews of Interventions version 6.5 (updated August 2024). Cochrane, 2024. Available from: www.training.cochrane.org/handbook. Date accessed: Feb 26.
